# In-Cage Recombination Facilitates the Enantioselective Organocatalytic [1,2]-Rearrangement of Allylic Ammonium Ylides

**DOI:** 10.1021/jacs.4c14516

**Published:** 2024-12-23

**Authors:** Will C. Hartley, Kevin Kasten, Mark D. Greenhalgh, Taisiia Feoktistova, Henry R. Wise, Jacqueline M. Laddusaw, Aileen B. Frost, Sean Ng, Alexandra M. Z. Slawin, Bela E. Bode, Paul Ha-Yeon Cheong, Andrew D. Smith

**Affiliations:** a EaStCHEM, School of Chemistry, University of St Andrews, North Haugh, St. Andrews, KY16 9ST, U.K.; b Department of Chemistry, University of Warwick, Coventry CV4 7AL, U.K.; c Department of Chemistry, Oregon State University, 153 Gilbert Hall, Corvallis, Oregon 97331, United States; d Syngenta, Jealott’s Hill International Research Centre, Bracknell, Berkshire RG42 6EY, U.K.

## Abstract

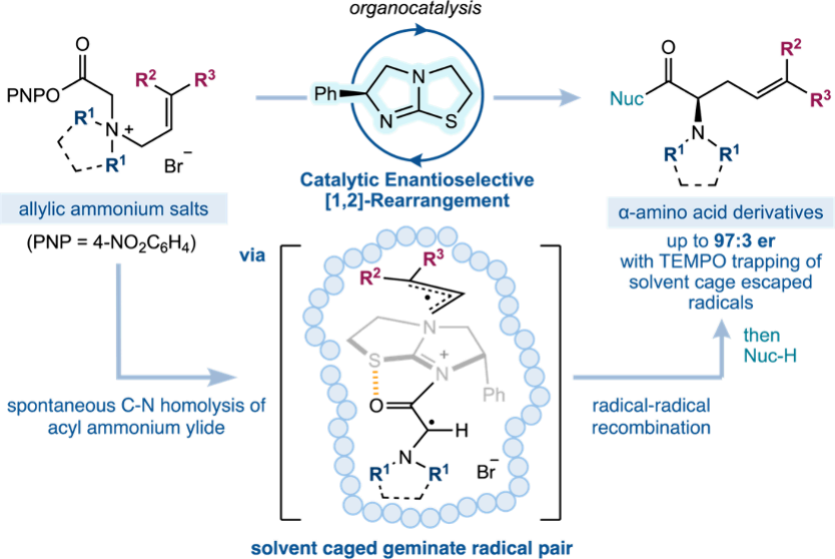

The [1,2]-rearrangement of allylic ammonium ylides is traditionally observed as a competitive minor pathway alongside the thermally allowed [2,3]-sigmatropic rearrangement. Concerted [1,2]-rearrangements are formally forbidden, with these processes believed to proceed through homolytic C–N bond fission of the ylide, followed by radical–radical recombination. The challenges associated with developing a catalytic enantioselective [1,2]-rearrangement of allylic ammonium ylides therefore lie in biasing the reaction pathway to favor the [1,2]-reaction product, alongside controlling a stereoselective radical–radical recombination event. Herein, a Lewis basic chiral isothiourea facilitates catalytic [1,2]-rearrangement of prochiral aryl ester ammonium salts to generate unnatural α-amino acid derivatives with up to complete selectivity over the [2,3]-rearrangement and with good to excellent enantiocontrol. Key factors in favoring the [1,2]-rearrangement include exploitation of disubstituted terminal allylic substituents, cyclic N-substituted ammonium salts, and elevated reaction temperatures. Mechanistic studies involving ^13^C-labeling and crossover reactions, combined with radical trapping experiments and observed changes in product enantioselectivity, are consistent with a radical solvent cage effect, with maximum product enantioselectivity observed through promotion of “in-cage” radical–radical recombination. Computational analysis indicates that the distribution between [1,2]- and [2,3]-rearrangement products arises predominantly from C–N bond homolysis of an intermediate ammonium ylide, followed by recombination of the α-amino radical at either the primary or tertiary site of an intermediate allylic radical. Electrostatic interactions involving the bromide counterion control the facial selectivity of the [1,2]- and [2,3]-rearrangements, while the sterically hindered tertiary position of the allylic substituent disfavors the formation of the [2,3]-product. These results will impact further investigations and understanding of enantioselective radical–radical reactions.

## Introduction

**Figure 1:**
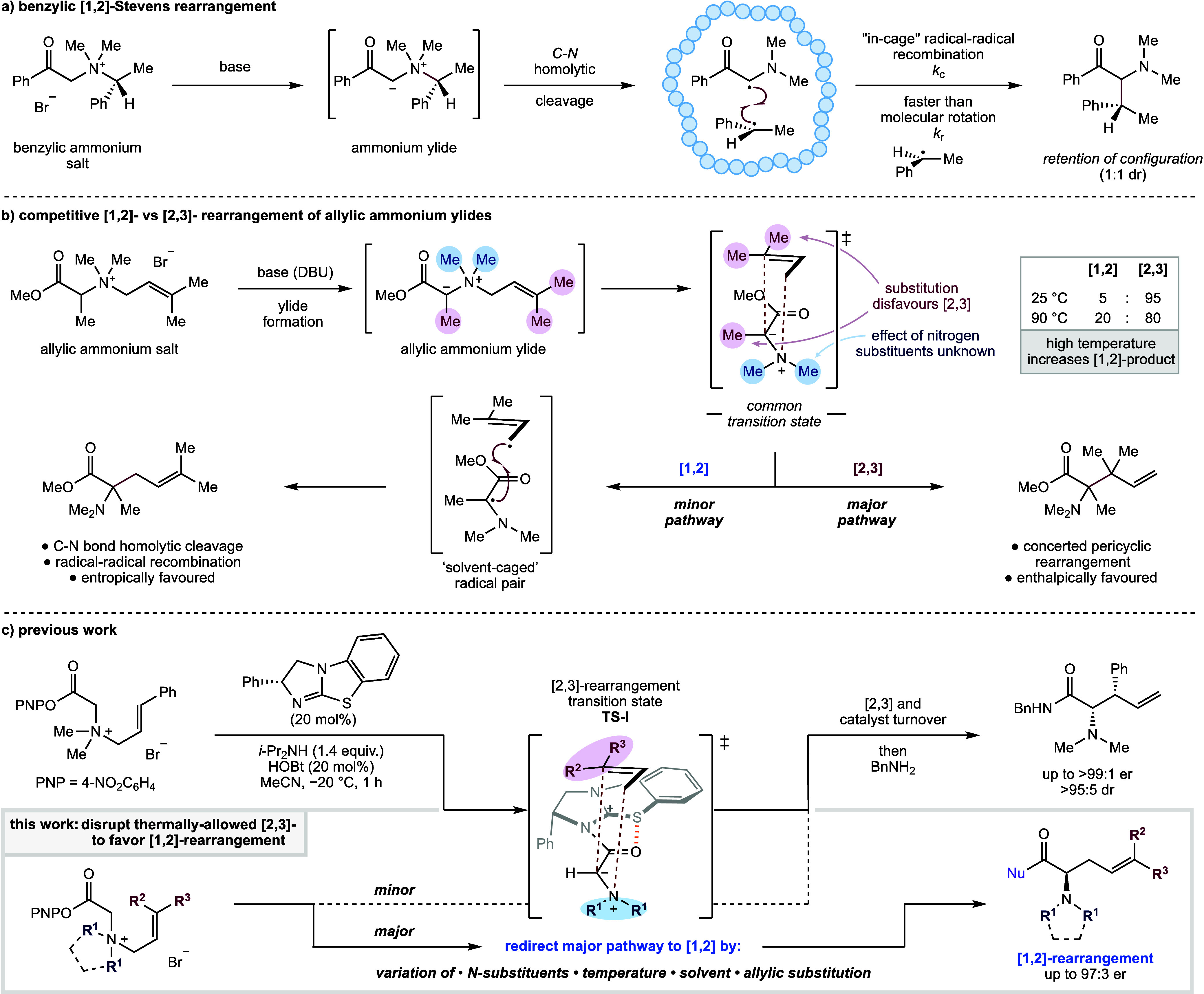
a) [1,2]-Stevens rearrangement of optically active benzylic ammonium ylides which proceeds with retention of configuration at the migrating carbon stereocenter. b) Singleton’s proposed common transition state for the competitive [1,2]- and [2,3]-rearrangements of allylic ammonium ylides (ref (9)). c) Previously disclosed enantio- and diastereoselective [2,3]-rearrangement of allylic ammonium ylides catalyzed by a chiral isothiourea and this work: the selective [1,2]-rearrangement of allylic ammonium ylides.

The dichotomy between competitive [1,2]- and [2,3]-rearrangements of onium ylides has been of long-standing interest to the synthetic community. Stevens reported the first [1,2]-rearrangement of a benzylic ammonium ylide in 1928,^1^ with the mechanism of this rearrangement thoroughly investigated and of widespread interest, as concerted [1,2]-rearrangements are forbidden by the Woodward–Hoffmann rules. Wittig^2^ and Hauser^3^ demonstrated that [1,2]-rearrangement proceeds with retention of configuration at the migrating carbon.^4^ The most widely accepted mechanistic explanation comes from detailed experiments by Ollis,^5−7^ which indicate homolytic C–N bond cleavage of the ammonium ylide generates a geminate radical pair that undergoes preferential “intramolecular” radical–radical recombination (Figure 1a). Experiments using an enantiopure benzylic ammonium salt **I** showed up to 99% enantiospecificity in viscous solvents, leading to the suggestion that radical–radical recombination takes place within the solvent cage at a faster rate (estimated at >10^11^ s^–1^)^7^ than molecular rotation or cage escape/diffusion of either of the radical partners (Figure 1a). The [1,2]-rearrangement of benzylic ammonium ylides has been most widely studied, as the competing [2,3]-rearrangement process (Sommelet–Hauser) is significantly disfavored due to the requirement for transient dearomatization. In contrast, allylic ammonium ylides typically give product mixtures that are ascribed to competitive [1,2]- and [2,3]-rearrangement, with the [1,2]-product usually being a minor reaction pathway. Biasing the product distribution to favor the [1,2]-rearrangement product is a recognized unmet challenge in this area, with previous studies indicating that a range of factors influence the ratio of [1,2]- and [2,3]-products. For example, strong bases and aprotic solvents,^8^ higher reaction temperatures,^5,6,9^ and the use of sterically congested and unstabilized ylides typically lead to a greater proportion of [1,2]-rearrangement.^5,6,10^ The current state-of-the-art understanding of these processes has been provided by Singleton and co-workers, with [1,2]- and [2,3]-rearrangement product mixtures shown to arise from a common transition state in which homolytic C–N bond cleavage is promoted by a loose [2,3]-transition state, leading to a maximum of 20% of the [1,2]-product at 90 °C (Figure 1b).^8,9^

Current asymmetric approaches to [1,2]-rearrangements of ammonium ylides are limited and generally rely upon either stoichiometric chiral auxiliaries^11,12^ or *stereospecific* rearrangements through N to C chirality transfer.^13−15^*Catalytic enantioselective* approaches are rare, with the difficulty in controlling radical–radical recombination leading to this being a recognized challenge in enantioselective synthesis. The current state-of-the-art is represented by the work of Arnold and co-workers, who recently established a biocatalytic [1,2]-Stevens type ring expansion of *N*-benzyl aziridines.^16^ Single examples of a phosphite-catalyzed enantioselective [1,2]-rearrangement of an N-benzylic isoquinoline derivative (94:6 er)^17^ and a copper-catalyzed enantioselective [1,2]-rearrangement of an allylic sulfonium ylide (69:31 er) have been demonstrated.^18^ During the finalization of this work, enantioselective variants involving ring expansion of conformationally restricted azetidine-derived allylic ammonium ylides that proceed through a stepwise intramolecular ion-pair pathway have been reported.^19,20^

In previous work, we developed the isothiourea-catalyzed enantioselective [2,3]-rearrangement of allylic ammonium ylides (Figure 1c).^21^ Experimental and computational studies led to the proposal of a highly stereodefined transition state **TS-I**, in which stereocontrol is governed by (i) the (*Z*)-configuration of the enolate-like ylide; (ii) a 1,5-O···S chalcogen interaction that locks the conformation of the acylated catalyst; (iii) the phenyl substituent of the catalyst promoting facial selectivity for rearrangement; and (iv) a π···isothiouronium interaction between the cinnamyl Ph substituent and the acylated catalyst.^22^ Building upon Singleton’s proposition of common [1,2]- and [2,3]-transition states,^8,9^ we rationalized that access to preorganized [2,3]-transition state **TS-I** could be exploited to develop a catalytic enantioselective [1,2]-rearrangement through incorporating steric hindrance at the terminal allylic substituent (to disfavor concerted [2,3]-rearrangement) while exploiting solvent polarity and increasing reaction temperature (Figure 1c, gray box). Herein is described a catalytic protocol using a commercially available isothiourea catalyst, tetramisole hydrochloride, that favors the formation of the [1,2]-rearrangement product with up to complete selectivity over the [2,3]-rearrangement product. Key factors that favor the [1,2]-rearrangement include disubstituted terminal allylic substituents and carbocyclic N-substituted ammonium salts, providing unnatural α-amino acid derivatives with good to excellent enantiocontrol. Detailed mechanistic studies indicate that maximizing pseudointramolecular “in-cage” radical–radical recombination is a requirement to generate the [1,2]-product with high enantiocontrol. Computational analysis supports the hypothesis of common [1,2]- and [2,3]-transition states and indicates that the [1,2]-product is formed via preferential C–N bond homolysis of an ylide intermediate, followed by stereoselective recombination of an allylic radical with a catalyst-bound captodative radical.

## Results and Discussion

### Initial Optimization

Optimization of a protocol to maximize the formation of the desired [1,2]-rearrangement product was conducted by using ammonium salt **1a**, bearing a terminal disubstituted allyl substituent that was predicted to disfavor formation of the [2,3]-rearrangement product (Table 1). MeCN was initially chosen as the reaction solvent due to its high polarity and resultant solubility of the substrate and isothiourea catalysts. Using tetramisole·HCl (TM·HCl) **2a** as the Lewis base catalyst at 40 °C with *i*-PrNEt_2_ as base, followed by amidation through addition of benzylamine (5 equiv), gave an 85:15 mixture of the desired [1,2]- and [2,3]-rearrangement products **3a** and **4a** (Table 1, entry 1). Purification gave amide **3a** in 46% yield with 97:3 er, validating the feasibility of a catalytic enantioselective protocol. Use of the alternative isothiourea catalysts **2b** and **2c** led to reduced selectivity for the [1,2]-product, giving an 80:20 and 25:75 mixture of **3a** and **4a** in 5:95 and 40:60 er, respectively (entries 2 and 3). For entries 1–3, in each case, only a moderate yield of product **3a** was observed as part of a complex product distribution. Further optimization aimed to maximize the formation and yield of the [1,2]-rearrangement product while maintaining good enantiocontrol by using catalyst **2a**. Changing the base to NEt_3_ led to higher product yields, while reducing the amount of benzylamine (to 1.5 equiv) led to selective amidation of the [1,2]-rearrangement product to give amide **3a** and simplified chromatographic separation of [1,2]-product amide **3a** and [2,3]-product ester **4a′** (entries 4–14). The effect of increasing the reaction temperature (at 10 °C increments from 30 to 70 °C) was probed (entries 4–8) with increasing selectivity of **3a:4a′** (from 67:33 to 88:12) and yield (from 57% to 79%) for the [1,2]-rearrangement product **3a** observed. This correlated with reduced enantioselectivity of [1,2]-rearrangement product **3a** (from 93:7 er to 85:15er). A reaction temperature of 50 °C (entry 6) was considered an optimal compromise between maximizing product yield and enantioselectivity, providing the [1,2]-rearrangement product **3a** in 72% isolated yield and 91:9 er. Reduced catalyst loading of **2a** (10 and 5 mol %, entries 9 and 10) led to diminished selectivity for [1,2]-product **3a** over [2,3]-product **4a′** and with reduced enantiocontrol. The iodide salt of **1a** was also subjected to rearrangement at 50 °C (entry 11), giving [1,2]-amide **3a** in 92:8 er. The use of alternative solvents of varying dielectric constant and viscosity was also investigated. Dichloromethane as a solvent gave significantly lower [1,2]:[2,3]-rearrangement selectivity (entry 12), while dimethylacetamide (DMA) and ethylene carbonate (EC) provided good [1,2]:[2,3]-rearrangement selectivity and marginally improved enantioselectivity relative to MeCN (entries 13 and 14). However, reactions in DMA led to significantly lower product yield (36%), while EC complicated product isolation due to competitive reaction with benzylamine to give a carbamate side product. Control reactions confirmed that [2,3]-rearrangement product ester **4a** does not isomerize to the [1,2]-rearrangement product under the standard reaction conditions. *In situ* reaction monitoring revealed a consistent ratio of [1,2]- to [2,3]-reaction products throughout the course of the reaction, again consistent with no interchange of [1,2]- and [2,3]-intermediates or products under the reaction conditions (see SI Section F for details). The absolute configuration of (*R,E*)-**3a** was proven by crystallographic analysis, consistent with no isomerization of the allylic fragment being observed in the generation of the [1,2]-product. The relative configuration of the [2,3]-rearrangement product **4a** was assigned by comparison to X-ray analysis of analogue **4j** (see SI Section E).

**Table 1:**
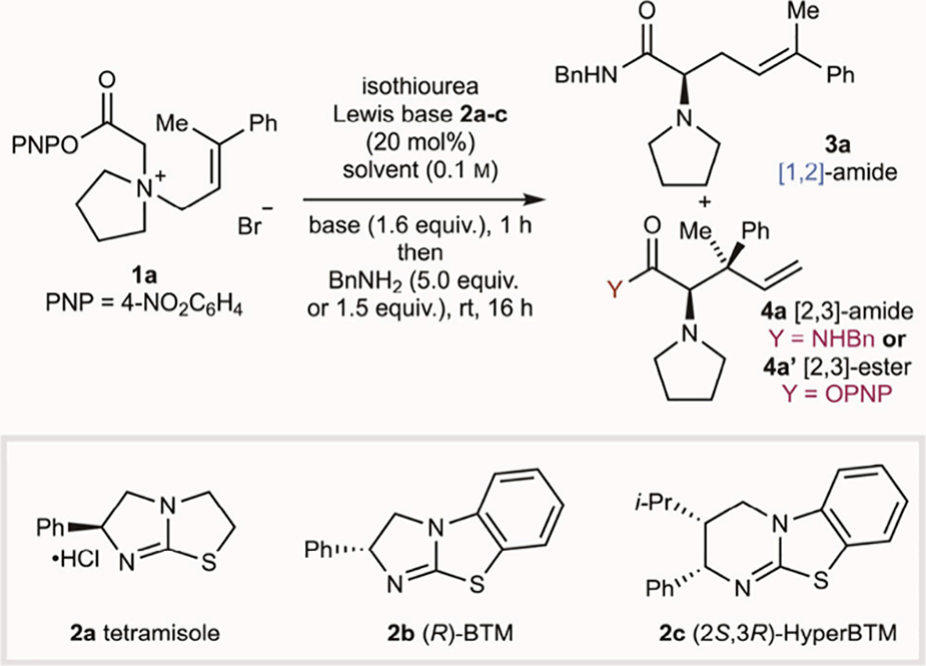
Reaction Optimization^a^

entry	solvent/catalyst	*T*/°C	ratio **3a**:**4**	yield **3a** [%]	er **3a**	yield **4** [%]
1^b^	MeCN/**2a**	40	85:15	46	97:3	8^c^
2^b^	MeCN/**2b**	40	80:20	47	5:95	12^c^
3^b^	MeCN/**2c**	40	25:75	13	40:60	38^c^
4^d^	MeCN/**2a**	30	67:33	57	93:7	29^e^
5^d^	MeCN/**2a**	40	80:20	70	92:8	17^e^
6^d^	MeCN/**2a**	50	84:16	75 (72)	91:9	14 (11)^e^,^f^
7^d^	MeCN/**2a**	60	86:14	76	84:16	12^e^
8^d^	MeCN/**2a**	70	88:12	79	85:15	11^e^
9^d^^,^^g^	MeCN/**2a**	50	71:29	50	90:10	27^e^
10^c^^,^^g^	MeCN/**2a**	50	65:35	53	88:12	22^d^
11^d^^,^^i^	MeCN/**2a**	50	81:19	52	92:8	12^e^
12^d^^,^^j^	CH_2_Cl_2_/**2a**	50	67:33	32	89:11	17^e^
13^d^	DMA/**2a**	50	84:16	36	93:7	7^e^
14^d^	EC/**2a**	50	75:25	68	94:6	21^e^

a Yields and [1,2]:[2,3]-rearrangement product ratios (**3a**:**4a** or **4a′**) calculated by ^1^H NMR analysis of the crude reaction product using 1,4-dinitrobenzene as internal standard; numbers in parentheses correspond to isolated yields. DMA = dimethylacetamide. EC = ethylene carbonate.

b Using *i*-Pr_2_NEt as base and BnNH_2_ (5 equiv).

c Product **4a**.

d Using NEt_3_ as base and BnNH_2_ (1.5 equiv).

e Product **4a′**.

f 80:20 dr; major diastereoisomer obtained in 78:22 er.

g Using **2a** (10 mol %).

h Using **2a** (5 mol %).

i Using the iodide salt of ammonium salt **1a**.

j Sealed tube.

### Scope and Limitations

**Figure 2:**
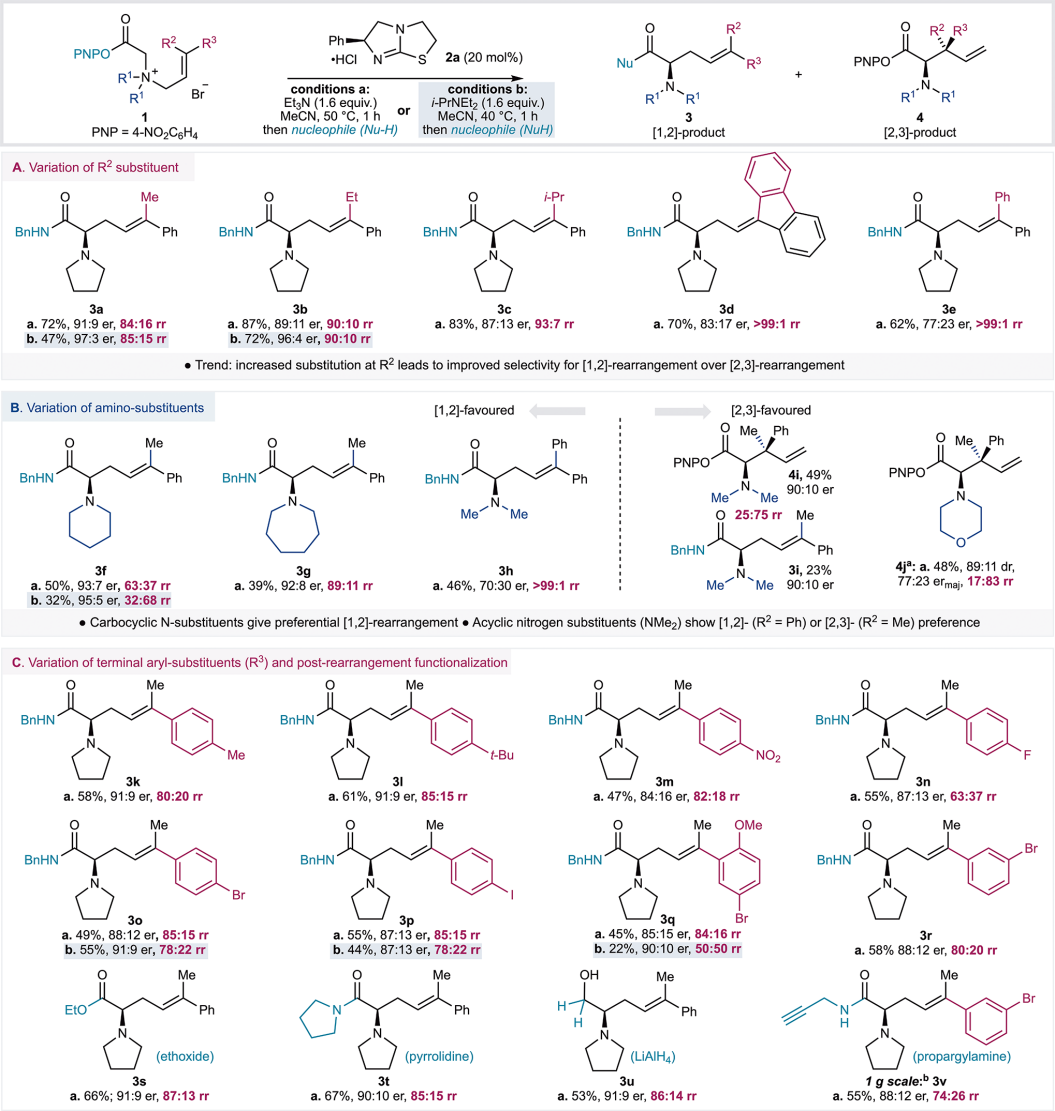
Scope of the reaction. Isolated yields are given. [1,2]:[2,3] product ratio calculated by ^1^H NMR analysis of the crude reaction product. ^*a*^[1,2]-rearrangement product was not isolated. ^*b*^Reaction was carried out on a 1 g scale. **A.** Variation of R^2^. **B.** Variation of R^1^_._**C.** Variation of R^3^ and nucleophile added at the end of the reaction. rr = rearrangement product ratio ([1,2]:[2,3] products).

With optimized conditions developed that maximized selectivity for the [1,2]-product over the [2,3]-product while compromising but maintaining satisfactory enantioselectivity, the scope and limitations of this process were investigated (Figure 2, reaction conditions **a**, 50 °C, NEt_3_). In a small number of selected cases, the alternative reaction conditions that in the optimization studies led to enhanced enantioselectivity of the [1,2]-product, while compromising yield, were also investigated to probe the generality of this process (reaction conditions **b**, 40 °C, *i*-PrNEt_2_). The effect of structural variation within the allylic substituents was first probed (Figure 2A).

Sequentially changing the R^2^ substituent from methyl **3a** through ethyl **3b** and isopropyl **3c** increased the ratio of [1,2]:[2,3]-rearrangement products from 84:16 to 90:10 to 93:7 (Figure 2A, conditions **a**), alongside a small reduction in enantioselectivity. Under reaction conditions **b**, **3a** and **3b** were generated in higher enantioselectivity while compromising product yield (97:3 er, 47% yield; 96:4 er, 72% yield). Both fluorenyl and diphenyl substitution gave exclusive [1,2]-rearrangement products **3d** and **3e** in 83:17 and 77:23 er, respectively. *In situ* reaction monitoring of the rearrangement to give **3e** showed exclusive formation of the [1,2]-reaction product throughout the course of the reaction, again consistent with no interchange of [1,2]- and [2,3]-intermediates or products under the reaction conditions. The sequential increase in [1,2]-selectivity observed across this series is consistent with a combination of both increasing steric hindrance at the alkene terminus disfavoring [2,3]-rearrangement and increasing stability of an assumed allylic radical formed following C–N bond homolysis. Control experiments showed that prenyl substitution within the allylic fragment provided no rearrangement products, while consistent with previous work under the conditions developed herein, cinnamyl substitution gave exclusive [2,3]-rearrangement (95%, >95:5 dr, 93:7 er) (see SI for details).^21^ These experiments demonstrate the importance of (i) a terminal aryl substituent to promote reactivity and (ii) terminal disubstitution to provide selectivity for the [1,2]-rearrangement.

Changing the nature of the N-substituents (R^1^) was next investigated and shown to have a profound effect on the ratio of rearrangement products (Figure 2B). Variation of the ring size showed that both piperidinyl and azepanyl derivatives provided preferential [1,2]-rearrangement at 50 °C (conditions **a**), giving products **3f** and **3g** with 93:7 and 92:8 er, respectively. Notably, rearrangement to give **3f** under conditions **b** led to improved product enantioselectivity (95:5 er), but gave **3f** as the minor product (32:68 rr). With acyclic *N*,*N*-dimethylamino substitution, [2,3]- or [1,2]-selectivity was dependent upon the nature of the terminal allylic substituents. Exclusive [1,2]-rearrangement was observed with diphenyl substitution (R^2^, R^3^ = Ph) to give **3h** in 70:30 er. However, when R^2^ = Me, the [2,3]-rearrangement product was preferred ([1,2]:[2,3] = 25:75 rr), with the major diastereoisomer of the [2,3]-rearrangement product **4i** and the [1,2]-rearrangement product **3i** both isolable in high enantiopurity (90:10 er). Morpholinyl substitution resulted in highly selective [2,3]-rearrangement ([1,2]:[2,3] = 17:83 rr), and while the [1,2]-rearrangement product could not be isolated, the [2,3]-rearrangement product **4j** was obtained in 48% yield and 77:23 er (major diastereoisomer). The relative and absolute configuration within **4j** was confirmed by X-ray analysis (see SI Section E for details).

Next, variation of the terminal aryl substituent R^3^ was investigated (Figure 2C). Introduction of methyl and *tert*-butyl groups in the *para*-position gave the desired rearrangement products **3k** and **3l** in 58% and 61% yield, respectively, and 91:9 er in both cases. With a *para*-NO_2_ electron-withdrawing group, the rearrangement was less efficient, leading to 47% of [1,2]-product **3m** in 84:16 er. *Para*-halogen substitution with fluorine, bromine, and iodine gave the [1,2]-products **3n**–**3p** in good yield, with progressively greater preference for [1,2]-rearrangement, while enantioselectivity remained high (≈90:10 er). *Ortho*- and *meta*-substitution was also well-tolerated, with **3q** and **3r** obtained in moderate yields but with high [1,2]-rearrangement selectivity. Finally, a range of nucleophiles can be used following the catalytic reaction to access different functional groups. Addition of sodium ethoxide gave ethyl ester **3s** in good yield and 91:9 er, while addition of pyrrolidine gave tertiary amide **3t** in excellent yield and 90:10 er. Pleasingly, addition of lithium aluminum hydride to the crude reaction product mixture after solvent exchange gave aminoalcohol **3u** in good yield with no erosion of enantiopurity. The reaction can also be carried out on a gram scale, with propargylic amide **3v** obtained in good yield and 88:12 er. Consistent with previous observations, rearrangement to give **3o**, **3p**, and **3q** using conditions **b** led to equivalent or improved product enantioselectivity (91:9, 97:13, and 90:10 er, respectively) but with reduced selectivity for the [1,2]-rearrangement products.

### Mechanistic Investigations

**Figure 3:**
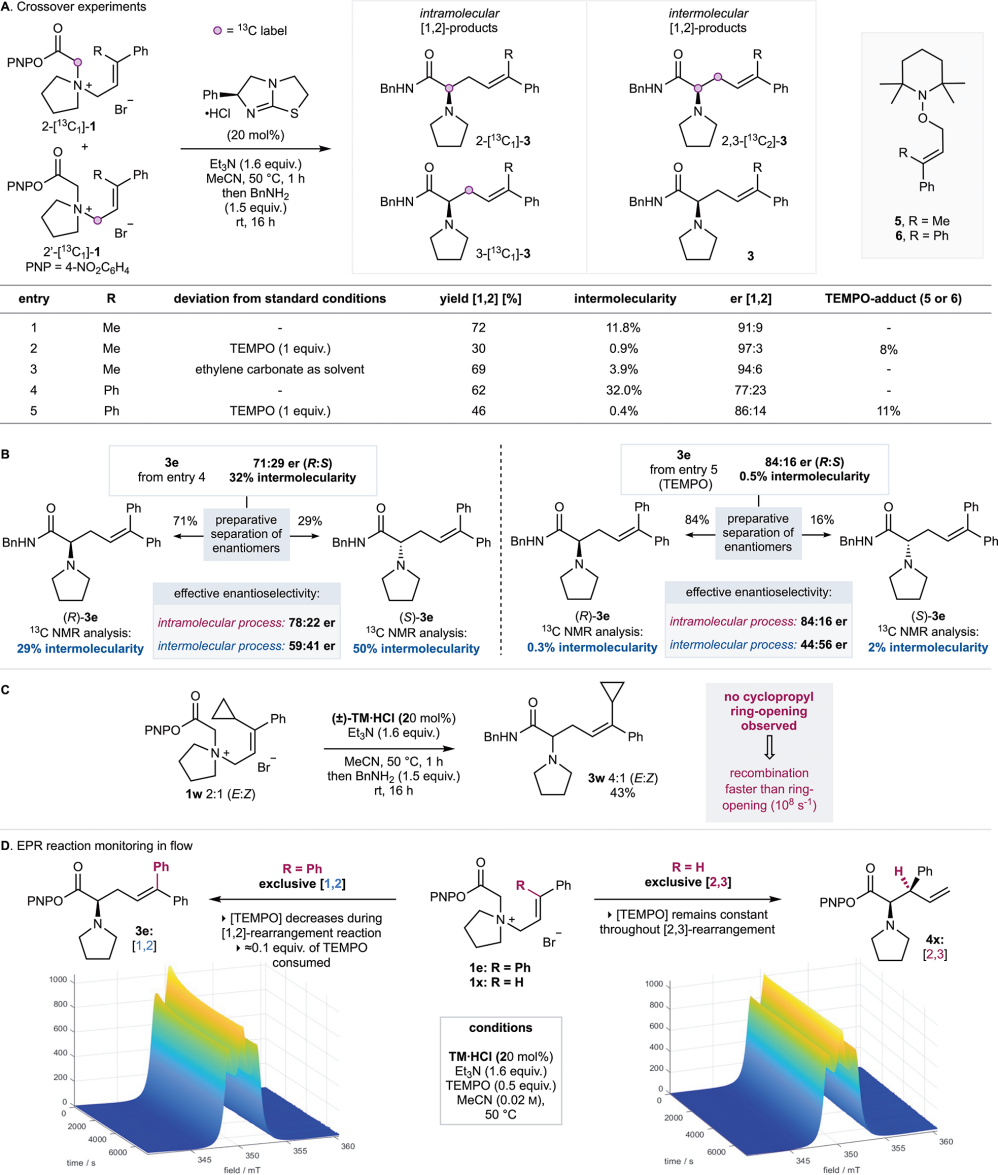
**A**. Crossover experiments were performed to determine intermolecularity of [1,2]-rearrangement. Isolated yields given. Intermolecularity calculated through quantitative ^13^C{^1^H} NMR analysis of purified [1,2]-rearrangement product, with extended delay (d1 = 43 s) to allow full relaxation of active nuclei (see SI for full details). **B**. Preparative separation of enantiomers from crossover experiments and subsequent analysis were performed by quantitative ^13^C{^1^H} NMR. **C**. Reaction of cyclopropyl-containing substrate **2w**. **D**. EPR spectroscopic monitoring of the TEMPO concentration in the rearrangement of **2e** (left) and **2x** (right).

Mechanistic investigations were then carried out to provide an in-depth understanding of the fundamental principles that underpin this catalytic enantioselective [1,2]-rearrangement. Crossover experiments were used to assess the intra- and intermolecularity of the [1,2]- and [2,3]-rearrangement products. As the rearrangement product distributions are highly sensitive to structural variation, minimizing the use of chemically differentiated substrates was deemed to be of paramount importance for experimental design. For this purpose, a series of allylic ammonium salts containing a single ^13^C-enriched isotopic label were prepared. In these reactions, the observation of crossover products containing two ^13^C-labels was taken as a measure of the intermolecularity of the [1,2]- and [2,3]-rearrangement processes. In the [1,2]-rearrangement case, this is assumed to be representative of competition between “in-cage” radical–radical recombination (considered as “intramolecular”) and diffusion of the radicals followed by recombination (considered as “intermolecular”). Treatment of a 1:1 mixture of salts 2-[^13^C_1_]-**1a** and 2′-[^13^C_1_]-**1a** under the optimized conditions and analysis of the resultant isolated [1,2]-rearrangement product indicated significant intermolecularity (12% crossover, Figure 3A, entry 1). Analogous treatment of a 1:1 mixture of salts 2-[^13^C_1_]-**1a** and 4′-[^13^C_1_]-**1a** indicated that the [2,3]-rearrangement product contained significantly reduced intermolecularity (2.6% crossover) (see SI Figure S12 for details). This is consistent with observations by Singleton, who also observed increased crossover in [1,2]-products compared to [2,3]-products.^9^ While Lehn and Kitching have shown that simple allylic and benzylic ammonium salts readily undergo salt exchange and metathesis,^23,24^ control studies indicate that salt metathesis processes (that could potentially allow ammonium substituent exchange and therefore crossover within starting materials) are not observed in this system under typical reaction conditions (see SI Figure S10 for details). A radical trap experiment using 1 equiv of TEMPO did not fully inhibit the reaction but gave the [1,2]-rearrangement product in a lower product yield (30%) with drastically reduced product intermolecularity (0.9%) and a substantial increase in enantiopurity (97:3 er). TEMPO adduct **5** was isolated in 8% yield, consistent with TEMPO trapping of an allylic radical intermediate (Figure 3A). The use of a viscous and polar solvent,^25,26^ ethylene carbonate, resulted in reduced intermolecularity (3.9%) with the [1,2]-rearrangement product again obtained with enhanced enantiopurity (94:6 er). Rearrangement of the corresponding ^13^C-labeled diphenyl-substituted salts 2-[^13^C_1_]-**1e** and 2′-[^13^C_1_]-**1e** gave [1,2]-rearrangement product **3e** in 77:23 er with significant levels of intermolecularity (32%). Performed in the presence of TEMPO, this rearrangement proceeded with remarkably reduced intermolecularity (0.4%) and increased product enantioselectivity (86:14 er) as well as giving 11% of the TEMPO-adduct **6**. To further probe the consistent trend in higher product enantioselectivity with reduced intermolecularity, the [1,2]-rearrangement products arising from the ^13^C-labeled diphenyl-substituted salts 2-[^13^C_1_]-**1e** and 2′-[^13^C_1_]-**1e** (in both the absence and presence of TEMPO) were further analyzed through preparative separation of the product enantiomers (from samples of 71:29 er and 84:16 er, respectively, Figure 3B). In both instances, higher intermolecularity was observed in the (*S*)-enantiomer (50% intermolecularity without TEMPO, 2% with TEMPO) than in the (*R*)-enantiomer (29% intermolecularity without TEMPO, 0.3% with TEMPO). Using these data, the enantioselectivity of the inter- and pseudo-intramolecular processes can be calculated. In both the absence and presence of TEMPO, the pseudo-intramolecular process is highly enantioselective (calculated as 78:22 and 84:16 er, respectively), while the intermolecular process leads to significantly reduced product enantioselectivity, giving close to racemic product (59:41 and 44:56 er, respectively). These experiments are consistent with “in-cage” radical–radical recombination being essential for high enantioselectivity, with the addition of TEMPO increasing product enantioselectivity by efficiently trapping the diffused radicals to minimize the intermolecular reaction that proceeds with reduced enantioselectivity.^27^

A cyclopropyl-substituted allyl substrate **1w** was next prepared as a control to probe the rate of recombination within the radical cage (Figure 3C). Despite an assumed rate of ≈10^8^ s^–1^ for the ring opening of an intermediate cyclopropyl methyl radical, only the [1,2]-product **3w** was observed, consistent with the rate of radical recombination (previously estimated as >10^11^ s^–1^)^7^ outcompeting ring-opening of the cyclopropyl fragment.^28^ The possibility of *in situ* identification of diffused radical intermediates was next sought. Given the significant 32% intermolecularity measured for the [1,2]-rearrangement of diphenyl-substituted salt **1e**, this substrate was selected for EPR studies, as it was considered that this level of intermolecularity may relate to the lifetime of a diffused radical (Figure 3D). However, attempts to directly detect radical intermediates proved unsuccessful, largely hampered by strong microwave absorption of the MeCN reaction solvent. To overcome this, an alternative approach was undertaken by monitoring the consumption of TEMPO by EPR in two distinct rearrangement reactions. A flow setup was used so the reaction solution could be passed directly through the EPR spectrometer and allow *in situ* reaction monitoring. First, the rearrangement of diphenyl-substituted salt **1e** (R = Ph), which proceeds with complete [1,2]-selectivity, and second the rearrangement of cinnamyl-substituted salt **1x** (R = H), which proceeds with complete [2,3]-selectivity, were studied. Applying 0.5 equiv of TEMPO in each reaction resulted in ≈0.1 equiv being consumed during the [1,2]-rearrangement of **1e**, whereas the concentration of TEMPO remained constant during the [2,3]-rearrangement of **1x**. These results are consistent with direct trapping of the allylic radical as TEMPO adduct **6** during the [1,2]-rearrangement, while no analogous product, or consumption of TEMPO, was identified from the [2,3]-rearrangement of **1x**.

### Computational Investigations

**Figure 4:**
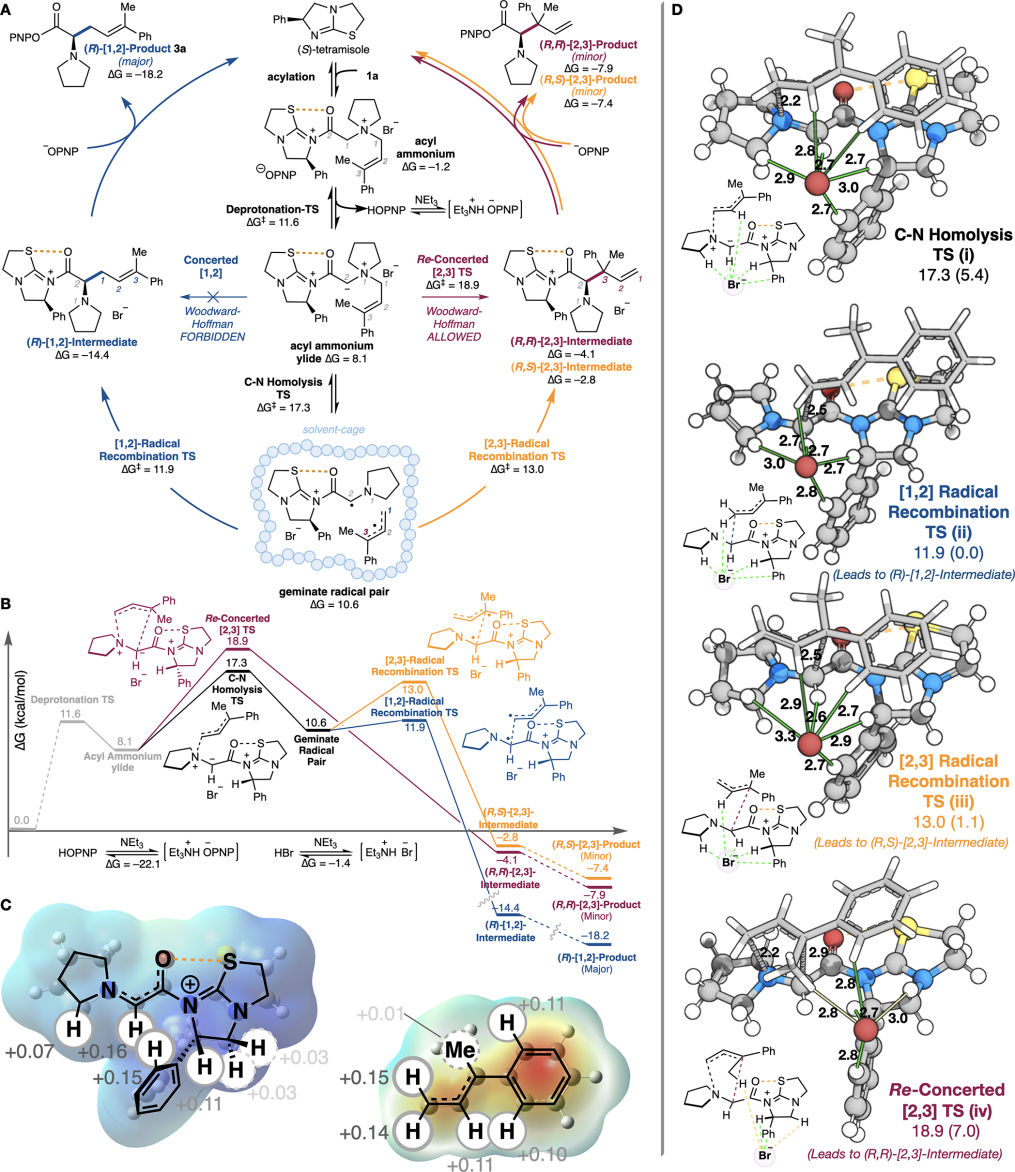
**A**. Proposed catalytic cycle and computed energies. [1,2]-Product forming processes are shown on the left (blue) and the minor [2,3]-product forming processes are shown on the right (red and orange). **B**. Computed reaction progress profile. **C**. Electrostatic potential maps and ChelpG charges of the acylated catalyst cation radical (left) and allyl radical (right). **D**. Computed transition states (TS) for the C–N homolysis, concerted [2,3]-sigmatropic rearrangement, and radical recombination processes that lead to [1,2]- and [2,3]-products. Green and yellow lines denote strong and weak electrostatic interactions, respectively.

DFT was used to further investigate the mechanism and selectivity of this reaction using tetramisole and allylic ammonium salt substrate **1a**, using M06/6-31G*/PCM(acetonitrile) as implemented in Gaussian16 with thermal corrections at 50 °C and Grimme’s D3 empirical dispersion corrections computed at the same geometries (see SI). All radical and transition state structures were computed by using unrestricted M06. The proposed catalytic cycle is shown in Figure 4. Notably, the inclusion and location of the bromide counterion that participates in multiple noncovalent stabilizing C–H/N–CH···Br^–^ interactions^29−38^ within all structures on the catalytic cycle were critical for attaining relative transition state energies that reproduce the experimentally observed preferential formation of the [1,2]-rearrangement product. In addition, exploration of the rearrangement for all transition state structures involving both facial approaches of the reactive allylic group for [1,2]- and [2,3]-processes was established, with the lowest energy structures used to construct the catalytic cycle (Figure 4A).

Initial acylation of isothiourea catalyst tetramisole by the allylic ammonium salt **1a** liberates 4-nitrophenoxide to give an acyl ammonium complex (Δ*G* = −1.2 kcal/mol). Deprotonation by 4-nitrophenoxide (deprotonation-TS, Δ*G*^⧧^ = 11.6 kcal/mol) results in formation of the reactive acyl ammonium ylide at 8.1 kcal/mol, with onward competitive reaction pathways feasible to generate the observed [1,2]- and [2,3]-products (see SI Section H for full details). While the acyl ammonium ylide can undergo a concerted-[2,3] sigmatropic rearrangement process (Δ*G*^⧧^ = 18.9 kcal/mol) to generate the (*R,R***)**-[2,3]-intermediate (Δ*G* = −4.1 kcal/mol), extensive efforts to find a concerted [1,2]-sigmatropic rearrangement from the acyl ammonium ylide failed to locate this transition state, consistent with this being a Woodward–Hoffmann thermally forbidden process. Instead, homolytic C–N bond cleavage (C–N homolysis-TS, Δ*G*^⧧^ = 17.3 kcal/mol) is preferred to concerted rearrangement and generates a solvent-caged geminate radical pair (Δ*G* = 10.6 kcal/mol), consisting of the catalyst-bound captodative radical and a phenyl-conjugated allylic radical. Radical recombination preferentially occurs at the least hindered primary (C1) site of the allylic radical ([1,2]-radical recombination TS, Δ*G*^⧧^ = 11.9 kcal/mol) to generate postrearrangement acyl ammonium ([1,2]-intermediate, Δ*G* = −14.4 kcal/mol) as the favored reaction pathway. Alternatively, recombination at the more hindered tertiary (C3) carbon of the allylic radical is feasible but higher in energy ([2,3]-radical recombination TS, Δ*G*^⧧^ = 13.0 kcal/mol) leading to the (*R,S*)**-**[2,3]-intermediate (Δ*G* = −2.8 kcal/mol). Subsequent turnover of catalyst–product complexes with 4-nitrophenoxide releases the observed ester products ([1,2]-product **3a** and diastereoisomeric [2,3]-products) and regenerates the tetramisole isothiourea catalyst.

There are four factors that govern the selectivities observed in this reaction (Figure 4):

### 1 Planarity of the Acyl Ammonium Intermediate Structures

All structures involving the acylated isothiourea are planar due to an O···S chalcogen bonding interaction (n_O_ to σ*_S–C_).^39^ This preorganization forces the radical–radical recombination event to preferentially occur *anti* to the stereodirecting catalyst phenyl substituent and explains the observed absolute configuration of the [1,2]-rearrangement product.

### 2 Location of the Bromide Counterion in [1,2]-Rearrangement Structures

The location of the bromide counterion to allow maximum stabilizing C–H/N–CH···Br^–^ electrostatic interactions was critical to reproduce the experimentally observed preference for the [1,2]-rearrangement product. Atomic charge analyses using ChelpG (Figure 4C) of the proposed intermediates reveal that (i) the catalyst-bound captodative radical carries substantial positive charge on a number of H atoms: C(2)H (+0.16), ^+^N–CH (+0.11), and ^+^N–CPh–H (+0.15) substituents; (ii) the substrate pyrrolidine N–CH bears more partial positive charge than the distal catalyst C*sp*^3^–H (+0.07 vs +0.03, respectively); and (iii) the C*sp*^2^–H at C(1) and C(2) of the allylic substituent bears more partial positive charge than the C*sp*^3^–H of the C(3)-methyl substituent (+0.11–0.15 vs 0.01, respectively). Considering the transition state structures (Figure 4D), the position of bromide being proximal to the pyrrolidine N–CH and leading to maximum stabilization through noncovalent C–H/N–CH···Br^–^ interactions (green dotted lines) is conserved in the C–N homolysis, as well as [1,2]- and [2,3]-radical recombination TS (Figure 4D i–iii, respectively). The lowest energy C–N homolysis and [1,2]-rearrangement transition state structures (Figure 4D i,ii place the allylic substituent in a conformation with the C(3)Me-substituent *syn* to the C=O of the acylated catalyst, maximizing C–H/N–CH···Br^–^ interactions (Figure 4D; green and yellow lines denote strong and weak electrostatic interactions, respectively). Alternative orientations of the allylic group lead to higher energy transition structures (see Supporting Information Section H). The bromide position being proximal to the pyrrolidine N–CH is not observed in the *Re*-concerted-[2,3]-TS (Figure 4D iv, where the allylic C(3)Me-substituent is *anti* to C=O of the acylated catalyst.

### 3 Electrostatic Interactions between the Acyl Ammonium Ylide and the Bromide Counterion Control the Stepwise vs Concerted Selectivity

From the acyl ammonium ylide, a concerted [1,2]-transition structure could not be found, consistent with this being a Woodward–Hoffmann thermally forbidden transformation. The concerted [2,3]-rearrangement can in principle form the new C–C bond on either the *Re*- or the *Si*-face of the allylic fragment. [2,3]-Rearrangement involving the *Si*-face was not found as a stationary point on the potential energy surface; exhaustive efforts to locate this structure decomposed into the C–N homolysis transition structure, suggesting that C–N homolysis is the favored process from this conformation. The only concerted [2,3]-rearrangement transition state found involves rearrangement through the *Re*-face of the allylic substituent (*Re*-concerted-[2,3]-TS), which corresponds to the configuration of the major diastereoisomer of the [2,3]-product obtained experimentally (Figure 4C). In a comparison of these two pathways, the C–N homolysis transition state is energetically preferred over the *Re*-concerted-[2,3]-TS due to greater stabilizing electrostatic interactions with the bromide counterion.

### 4 Steric Effects of the Allyl Substituents

The regioselectivity between stepwise [1,2]- and stepwise [2,3]-recombination is determined by steric occlusion of C1 versus C3 of the allylic fragment. In the stepwise [1,2]-process ([1,2]-radical recombination-TS), the C–C bond forms at the primary C1 allylic carbon, which experiences minimal steric repulsion. However, in the stepwise [2,3]-process the C–C bond forms at the more substituted tertiary C(3)-allylic carbon ([2,3]-radical recombination-TS). Distortion–interaction analysis reveals that indeed the [2,3]-radical recombination-TS is more distorted than the [1,2]-radical recombination-TS, consistent with the hypothesis that forming a bond to the tertiary carbon of the allylic fragment is energetically unfavorable (see Supporting Information Section H). As expected, the *Re*-concerted-[2,3]-TS, which involves bond forming and breaking at both the primary and the tertiary carbons of the allylic group, experiences the greatest distortion, further explaining why the [1,2]-process is favored over both the concerted and stepwise [2,3]-processes. Computational analysis of a model system in which the allylic group bears just a single phenyl substituent predicts a switch in selectivity to now favor the concerted [2,3]-process, in keeping with experimental results.

## Conclusions

In summary, the first catalytic enantioselective protocol that favors the [1,2]-rearrangement of allylic ammonium ylides has been developed. Using a commercially available isothiourea catalyst, tetramisole hydrochloride, up to complete selectivity for the [1,2]-product over the [2,3]-product was observed, with the [1,2]-products obtained in good yield with good to excellent enantioselectivity. Key factors that favor the [1,2]-rearrangement include disubstituted terminal allylic substituents, carbocyclic N-substituted ammonium salts, and higher reaction temperatures. The use of ^13^C-isotopic labeling in crossover and radical-trapping experiments has revealed an intriguing interplay between intermolecularity and product enantioselectivity, consistent with “in-cage” radical recombination leading to high enantioselectivity. Computational analysis indicates that C–N bond homolysis from an acyl ammonium ylide intermediate, followed by regio- and enantioselective radical–radical recombination at the primary site of the intermediate allylic radical, leads to the [1,2]-product. The [2,3]-product can be formed through either a concerted [2,3]-sigmatropic rearrangement or radical–radical recombination at the tertiary site of the allylic radical. The fundamental mechanistic insight provided from this study, particularly the relationship between intermolecularity and product enantioselectivity, will have important consequences for stereoselective synthetic methods in general and for future investigations of solvent-caged enantioselective radical–radical recombination reactions.

## Data Availability

The data underpinning this manuscript are available from the University of St Andrews Research Portal, Pure ID: 298800968, “In-Cage Recombination Facilitates the Enantioselective Organocatalytic [1,2]-Rearrangement of Allylic Ammonium Ylides”.

## Supporting Information Available

Experimental procedures, characterization data, NMR spectra, HPLC traces, DFT calculations (PDF)
