# Supplementary material for: In-Cage Recombination Facilitates the Enantioselective Organocatalytic [1,2]-Rearrangement of Allylic Ammonium Ylides
Source: J Am Chem Soc. 2024 Dec 23;147(1):1101–11. doi: 10.1021/jacs.4c14516 (PMC11726568; doi:10.1021/jacs.4c14516)
Supplement: Supplementary file 1 — ja4c14516_si_001.pdf [file ja4c14516_si_001.pdf]

Supporting Information

In-cage Radical Recombination In The Enantioselective Organocatalytic [1,2]-Rearrangement of Allylic Ammonium Ylides

Will C. Hartley,[1] Kevin Kasten,[1] Mark D. Greenhalgh,[1,2] Taisiia Feoktistova,[3,‡] Henry B. Wise,[3,‡] Jacqueline M. Laddusaw,[3,‡], Aileen B. Frost,[1] Sean Ng,[4] Alexandra M. Z. Slawin,[1] Bela E. Bode,[1] Paul Ha-Yeon Chong[3*] & Andrew D. Smith[1*]

[1] EaStCHEM, School of Chemistry, University of St Andrews, North Haugh, St Andrews, KY16 9ST, UK.

[2] Department of Chemistry, University of Warwick, CV4 7AL, UK

[3] Department of Chemistry, Oregon State University, 153 Gilbert Hall, Corvallis, OR 97331, USA.

[4] Syngenta, Jealott's Hill International Research Centre, Bracknell, Berkshire, RG42 6EY, UK.

*Correspondence to:

Prof. Paul Ha-Yeon Cheong: cheongh@oregonstate.edu

Prof. Andrew D. Smith: ads10@st-andrews.ac.uk

Table of Contents

A. General Information ... 3

A. 1. Purification of Reagents ... 3

A. 2. Experimental Details ... 3

A. 3. Purification of Products ... 3

A. 4. Analysis of Products ... 3

B. Reaction optimization and control experiments ... 5

B. 1. Solvent, base and temperature ... 5

B. 2. Control and additive experiments ... 6

B. 3. Effect of radical trap additives ... 7

C. Substrate synthesis ... 8

C. 1. General Procedures ... 8

C. 2. Synthesis and characterisation of substrates ... 10

D. Characterisation of rearrangement products ... 39

D. 1. Scope ... 39

D. 2. Synthesis and characterisation of ^{13}C-labelled compounds ... 58

D. 3. TEMPO adducts ... 70

E. Crystallographic data ... 71

F. Mechanistic Studies ... 73

F. 1. Crossover experiments using ^{13}C-isotopic labelling ... 73

F. 2. EPR spectroscopy ... 86

F. 3. Product Isomerisation ... 88

G. Solvent properties ... 90

H. Computational Details ... 91

H. 1. Computed Geometries, Dispersion & Solvation Corrections, and Energies ... 91

H. 2. Transition States ... 227

H. 3. Juxtaposition to Acyclic N,N-Dimethylamino Substrate ... 231

I. References ... 233

J. NMR spectra ... 237

K. HPLC traces ... 418

A. General Information

All reagents and solvents were obtained from commercial suppliers and were used without further purification unless otherwise stated. Purification was carried out according to standard laboratory methods. (±)-Tetramisole•HCl and (*S*)-tetramisole•HCl was obtained from Sigma-Aldrich, benzotetramisole (BTM)[1] and HyperBTM[2] were synthesised in house. Racemic products were obtained using (±)-tetramisole•HCl.

Purification of Solvents

Anhydrous solvents (Et_2O, CH_2Cl_2, THF and PhMe) were obtained after passing through an alumina column (Mbraun SPS-800). Anhydrous DMA, DMF and MeCN were obtained from Sigma-Aldrich and used without further purification. Petrol is defined as petroleum ether 40-60 °C. All other solvents and commercial reagents were used as received without further purification unless otherwise stated. EtOAc, Et_2O, CH_2Cl_2 and Petrol for purification purposes were used as obtained from suppliers without further purification.

A. 1. Purification of Reagents

Dry *i*-Pr_2NEt and Et_3N was obtained by distillation over KOH and transferred to and stored in a screw-top vial over KOH and purged with and stored under argon.

A. 2. Experimental Details

Reactions were carried out in flame-dried glassware under an inert atmosphere (N_2) using standard vacuum line techniques. Purging refers to a vacuum/nitrogen-refilling procedure. Room temperature (rt) refers to 15–25 °C. Temperatures of 0 °C and −78 °C were obtained using an ice/water and CO_2(s)/acetone baths, respectively. Reactions involving heating were performed using DrySyn blocks and a contact thermocouple. Under reduced pressure refers to the use of either a Büchi Rotavapor R-200 with a Büchi V-491 heating bath and Büchi V-800 vacuum controller, a Büchi Rotavapor R-210 with a Büchi V-491 heating bath and Büchi V-850 vacuum controller, a Heidolph Laborota 4001 with vacuum controller, an IKA RV10 rotary evaporator with a IKA HB10 heating bath and ILMVAC vacuum controller, or an IKA RV10 rotary evaporator with a IKA HB10 heating bath and Vacuubrand CVC3000 vacuum controller. Rotary evaporator condensers are fitted to Julabo FL601 Recirculating Coolers filled with ethylene glycol and set to –6 °C.

A. 3. Purification of Products

Analytical thin layer chromatography was performed on pre-coated aluminium plates (Kieselgel 60 F254 silica) and visualisation was achieved using ultraviolet light (254 nm) and/or staining with either aqueous $KMnO_4$ solution or ethanolic Vanillin solution followed by heating. Manual column chromatography was performed in glass columns fitted with porosity 3 sintered discs over Kieselgel 60 silica using the solvent system stated. Automated chromatography was performed on a Biotage Isolera Four running Biotage OS578 with a UV/Vis detector using the method stated and cartridges filled with Kieselgel 60 silica.

A. 4. Analysis of Products

Melting points (mp) were recorded on an Electrothermal 9100 melting point apparatus, (dec) refers to decomposition.

Optical rotations $[\alpha]_D^{20}$ were measured on a Perkin Elmer Precisely/Model-341 polarimeter operating at the sodium D line with a 100 mm path cell at 20 °C.

HPLC analyses were obtained on either a Shimadzu HPLC consisting of a DGU-20A5 degassing unit, LC-20AT liquid chromatography pump, SIL-20AHT autosampler, CMB-20A communications bus module, SPD-M20A diode array detector and a CTO-20A column oven or a Shimadzu HPLC consisting of a DGU-20A5R degassing unit, LC-20AD liquid chromatography pump, SIL-20AHT autosampler, SPD-20A UV/Vis detector and a CTO-20A column oven. Separation was achieved using either DAICEL CHIRALCEL OD-H and OJ-H columns or DAICEL CHIRALPAK AD-H, AS-H, IA, IB, IC and ID columns using the method stated. HPLC traces of enantiomerically enriched compounds were compared with authentic racemic spectra.

Infrared spectra (ν_{max}) were recorded on a Shimadzu IRAffinity-1 Fourier transform IR spectrophotometer fitted with a Specac Quest ATR accessory (diamond puck). Spectra were recorded of either thin films or solids, with characteristic absorption wavenumbers (ν_{max}) reported in cm^{-1}.

^{1}H, $^{13}C\{^{1}H\}$, and ^{19}F NMR spectra were acquired on either a Bruker AV400 with a BBFO probe, a Bruker AVII 400 with a BBFO probe, a Bruker AVIII-HD 500 with a SmartProbe BBFO+ probe, or a Bruker AVIII 500 with a CryoProbe Prodigy BBO probe, in the deuterated solvent stated. All chemical shifts are quoted in parts per million (ppm) relative to the residual solvent peak.[3] All coupling constants, J, are quoted in Hz. Multiplicities are indicated as s (singlet), d (doublet), t (triplet), q (quartet), m (multiplet), and multiples thereof. The abbreviation Ar denotes aromatic and app denotes apparent.

Mass spectrometry (HRMS) data were acquired by either electrospray ionisation (ESI), electron impact (EI), atmospheric solids analysis probe (ASAP), or nanospray ionisation (NSI) at either the University of St Andrews Mass Spectrometry Facility ([A] quoted) or at the EPSRC UK National Mass Spectrometry Facility at Swansea University ($[A]^{+}$ or $[A]^{-}$ quoted).

B. Reaction optimization and control experiments

B. 1. Solvent, base and temperature

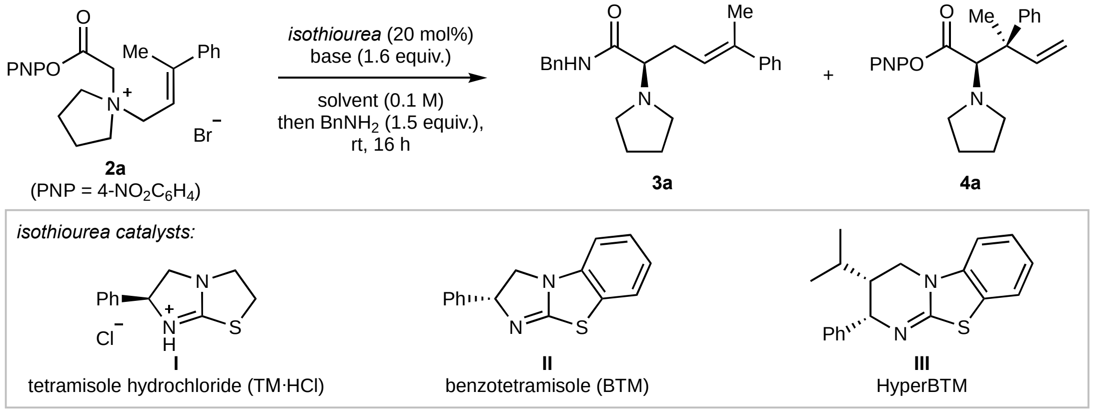

entry	catalyst	solvent	base	temp.	time (h)	yield [1,2] [%]	er	yield [2,3] [%] (dr)	ratio [1,2]:[2,3]
1	**I**	MeCN	*i*-Pr_2NEt	40 °C	1	46	97:3	8 (n/d)	5.8 : 1
2	**II**	MeCN	*i*-Pr_2NEt	40 °C	1	47	95:5	12 (95:5)	3.9 : 1
3	**III**	MeCN	*i*-Pr_2NEt	40 °C	1	13	60:40	38 (79:21)	1 : 2.9
4	**I**	MeCN	*i*-Pr_2NEt	rt	24	26	85:15	28 (90:10)	1 : 1.1
6	**I**	MeCN	*i*-Pr_2NEt	50 °C	1	49	94:6	2 (n/d)	25 : 1
7	**I**	DMSO	*i*-Pr_2NEt	40 °C	1	39	92:8	13 (86:14)	3 : 1
8	**I**	DMF	*i*-Pr_2NEt	40 °C	1	13	96:4	14 (79:21)	1 : 1.1
9	**I**	EC	*i*-Pr_2NEt	40 °C	1	40	98:2	17 (>95:5)	2.4 : 1
10	**I**	EC	Et_3N	40 °C	1	63	96:4	25 (84:16)	2.5 : 1
12	**I**	EC	Et_3N	50 °C	1	69	94:6	21 (80:20)	3.2 : 1
13	**I**	MeCN	Et_3N	30 °C	1	57	93:7	29	2 : 1
14	**I**	MeCN	Et_3N	40 °C	1	70	92:8	17	4.1 : 1
**15**	**I**	**MeCN**	**Et_3N**	**50 °C**	**1**	**75 (72)[a]**	**91:9**	**14**	**5.4 : 1**
16	**I**	MeCN	Et_3N	60 °C	1	80	85:15	3	27 : 1
17	**I**	MeCN	Et_3N	70 °C	1	66	84:16	4	17 : 1

Table S1. Yield of products determined by ^1H NMR analysis of the crude reaction residue using 1,4-dinitrobenzene as internal standard. [a] isolated yield. For [2,3]-rearrangement, the ratio in parentheses refers to the dr obtained from analysis of the ^1H NMR spectrum of the crude reaction product.

B. 2. Control and additive experiments

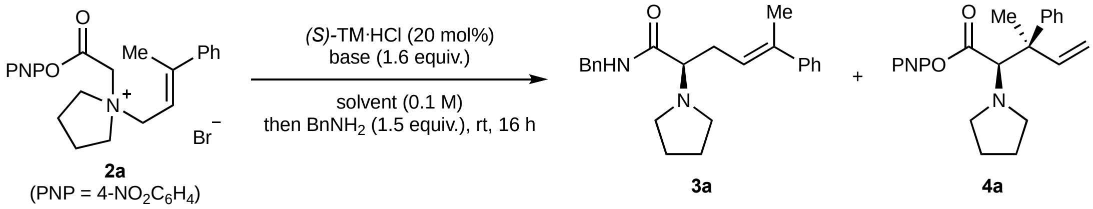

entry	deviation	temp.	yield **3a** [1,2] [%]	e.r. of **3a**	yield **4a** [2,3] [%]	ratio [1,2]:[2,3]
1	No catalyst	50 °C	<5	-	<5	-
2	Exclusion of light	50 °C	68	91:9	12	5.7 : 1
3	Degassed solvent	50 °C	63	91:9	9	7 : 1
4	Irradiation with black light	40 °C	23	n.d	0	[1,2] only
5	UV light + 9,10-diphenylanthracene (1 equiv.)	40 °C	33	n.d	0	[1,2] only
6	Bu_4N(OPNP) (1 equiv.)	40 °C	33	n.d	12	2.8 : 1
7	4Å MS	40 °C	64	88:12	21	3 : 1
8	No $BnNH_2$ quench	50 °C	26	91:9	8	3.3 : 1
9	No Et_3N	50 °C	0	-	-	-

Table S2. n.d = not determined. NB Entry 9: free based (*S*)-tetramisole was used as catalyst, which was prepared by dissolving (*S*)-tetramisole·HCl in dichloromethane, washed three times with aqueous NaOH (1M), dried over $MgSO_4$, filtered and concentrated *in vacuo*. Yields determined by ^{1}H-NMR analysis of the crude reaction product. Black light refers to a light source with λ_{max} = 365 nm.

B. 3. Effect of radical trap additives

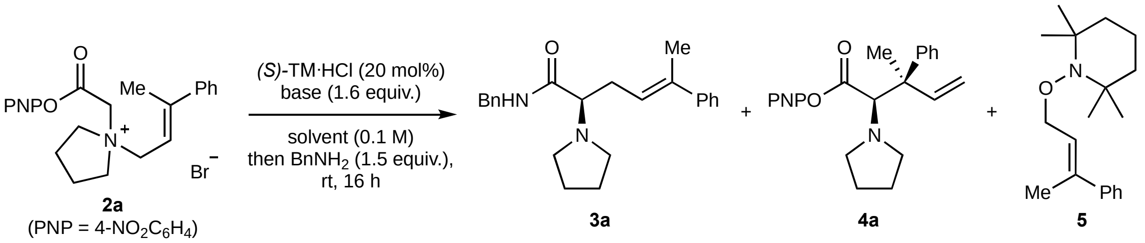

entry	solvent	additive	yield **3a** [1,2] [%]	e.r. of **3a**	yield **4a** [2,3] [%]	yield **5** [%]
1	MeCN	-	75	91:9	14	n/a
2	MeCN	TEMPO (1 equiv.)	30	97:3	13	8
3	MeCN	TEMPO (10 equiv.)	23	98:2	-*	16
4	EC	-	69	91:9	21	n/a
5	EC	TEMPO (5 equiv.)	43	93:7	-*	5
6	CH_2Cl_2	-	32	89:11	17	n/a
7	CH_2Cl_2	TEMPO (1 equiv.)	27	96:4	17	12
8	DMA	-	36	93:7	7	n/a
9	DMA	TEMPO (1 equiv.)	34	93:7	8	2
10	MeCN	1,4-cyclohexadiene (1 equiv.)	72	94:6	15	n/a
11	MeCN	1,1-diphenylethylene (1 equiv.)	66	91:9	13	n/a

Table S3. * [2,3] product not isolated due to co-elution with various side-products. When TEMPO was used, the yields refer to isolated compounds since crude ^{1}H NMR analysis was hampered by the paramagnetic properties of TEMPO. EC = ethylene carbonate. n/a = not applicable.

C. Substrate synthesis

C. 1. General Procedures

*General Procedure **A**: Wadsworth-Emmons olefination*

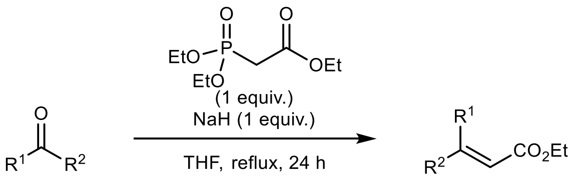

To a flame-dried round bottomed flask under an atmosphere of nitrogen was added anhydrous THF (0.3 M) and NaH (1 equiv.). The requisite phosphonoacetate ester (1 equiv.) was added dropwise at 0 °C, which induced effervescence. After addition was complete, the reaction was stirred at room temperature for 20 minutes, before addition of the requisite ketone/aldehyde (1 equiv.). The reaction was then heated at reflux for 16 hours before cooling to room temperature. Water (20 mL) was added carefully to quench excess NaH. The layers were separated, and the organic layer washed with aqueous HCl (3 × 25 mL) and then brine (25 mL), which was dried over anhydrous magnesium sulfate, filtered and concentrated *in vacuo*. The crude residue was purified by flash chromatography on silica gel (typically petroleum ether 40/60 : Et_2O) to afford the (*E*)-α,β-unsaturated esters.

*General Procedure **B**: Reduction of α,β-unsaturated esters to allylic alcohols*

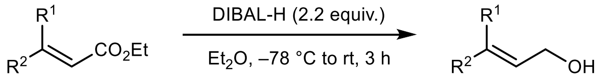

The requisite α,β-unsaturated ester (1.0 equiv.) was dissolved in anhydrous Et_2O and cooled to −78 °C. DIBAL-H (2.2 equiv., 1.0 M in hexanes) was added dropwise and the reaction was stirred at −78 °C for 3 hours. The reaction was warmed to room temperature for 1 hour before saturated aqueous NH_4Cl was added (~ 1/10 vol.) dropwise at 0 °C until the reaction mixture turned cloudy. Saturated aqueous Rochelle's salt (potassium sodium tartrate) was added (equal vol.) and the mixture stirred vigorously for 1 hour until the emulsion homogenized. The layers were separated and the organic layer washed with aqueous saturated NH_4Cl (2 × 1/2 vol.), brine (1/2 vol.) and then dried over magnesium sulfate, filtered and concentrated *in vacuo*. The crude product was used without further purification unless stated otherwise.

*General Procedure **C**: Bromination of allylic alcohols*

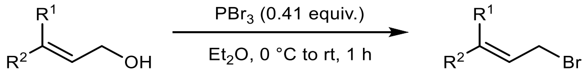

The requisite allylic alcohol (1.0 equiv.) was dissolved in anhydrous Et_2O and cooled to 0 °C. Phosphorous tribromide (0.41 equiv.) was added dropwise and the reaction was stirred at room temperature for 1 hour. The reaction was quenched with saturated aqueous $NaHCO_3$ (1/4 vol.), and the layers separated. The organic layer was washed with saturated $NaHCO_3$ (2 × 1/4 vol.) and a mixture of saturated aqueous $Na_2S_2O_3$ and brine (1:1, 1/4 vol.), dried over magnesium sulfate, filtered and concentrated *in vacuo*. The crude product was used without further purification.

*General Procedure **D**: Synthesis of allylic tertiary amines from allylic bromides*

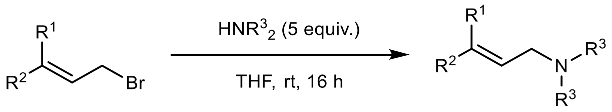

The requisite allylic bromide (1.0 equiv.) was dissolved in anhydrous THF, and the requisite secondary amine (5 equiv.) was added dropwise. The reaction was stirred at room temperature for 16 hours. Aqueous NaOH (equal vol., 1 M) was added and the mixture stirred for 1 hour. The layers were separated, and the aqueous layer washed with Et_2O (3 × equal vol.). The combined organic layers were washed with brine (1/2 vol.), dried over magnesium sulfate, filtered and concentrated *in vacuo*. The crude residue was dissolved in a small amount of diethyl ether and filtered to remove any solids and then concentrated *in vacuo*. The crude product was used without further purification unless stated otherwise.

*General Procedure **E**: Synthesis of Allylic Ammonium Salts*

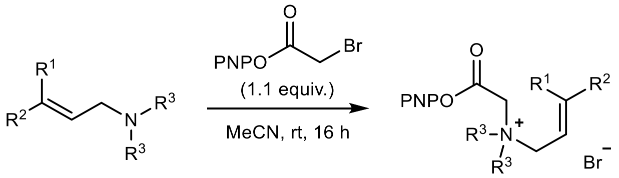

The requisite allylic tertiary amine (1.0 equiv.) was dissolved in anhydrous MeCN (1.1 M) before addition of 4-nitrophenyl 2-bromoacetate (1.1 equiv.). The desired ammonium salt often precipitated from solution after 5 minutes, but the reaction was stirred at room temperature for a further 16 hours to ensure full alkylation of the tertiary amine. Et_2O (10 × vol.) was added slowly, and the precipitate was filtered and washed with cold Et_2O ether to give the crude product which was dried *in vacuo* and used without further purification unless stated otherwise.

*General Procedure **F**: Isothiourea-catalysed enantioselective [1,2]-rearrangement of allylic ammonium ylides*

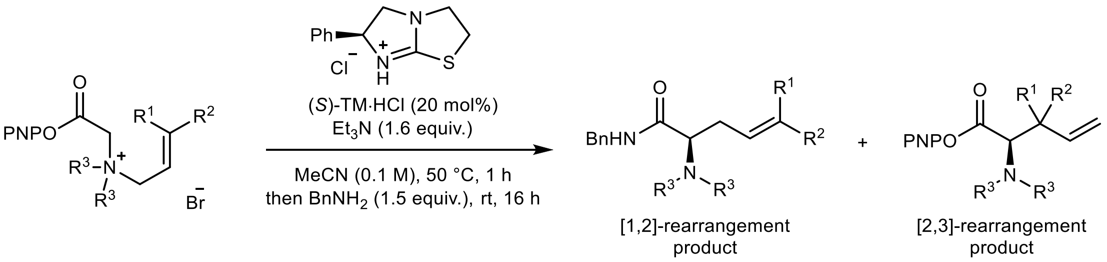

To a septum-capped screw top vial was added allylic ammonium salt (1 equiv.), isothiourea catalyst (20 mol%) and anhydrous MeCN (0.1 M). The solution was heated to 50 °C, and triethylamine (1.6 equiv.) was added and the reaction stirred for 1 hour at 50 °C. The reaction was then cooled to room temperature before addition of the requisite nucleophile (1.5 equiv.), and the reaction stirred for 16 hours at room temperature. The reaction mixture was concentrated *in vacuo* and crude ^{1}H-NMR analysis was used to determine the product distribution. The crude residue was purified by flash chromatography on silica gel to afford the [1,2]-rearrangement product (the [2,3]-rearrangement was usually obtained as an impure mixture of diastereoisomers with PNPOH contamination, unless stated otherwise).

C. 2. Synthesis and characterisation of substrates

C. 2. i. α,β-Unsaturated esters

Ethyl (*E*)-3-phenylbut-2-enoate S1

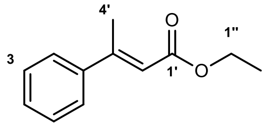

Following General Procedure **A**, ethyl 2-(diethoxyphosphoryl)acetate (10.0 mL, 50.4 mmol), sodium hydride (2.02 g, 50.4 mmol, 60% in mineral oil), acetophenone (5,88 mL, 50.4 mmol) in THF gave the crude product, which was purified by column chromatography (Petroleum ether 40/60 : diethyl ether (97:3)) to give the title compound as a colourless oil (7.38 g, 77%) with spectroscopic data in accordance with the literature;[4]

^{1}H-NMR (400 MHz, $CDCl_3$) δ_H: **1.32** (3H, t, $^3J_{HH}$ = 7.1, OCH_2CH_3), **2.58** (3H, d, $^4J_{HH}$ = 1.3, C(4`)$H_3$), **4.22** (2H, q, $^3J_{HH}$ = 7.1, $OCH_2CH_3$), **6.14** (1H, q, $^4J_{HH}$ = 1.3, C(2`)*H*), **7.34 – 7.41** (3H, m, ArC(2,4,6)*H*), **7.46 – 7.50** (2H, m, ArC(3,5)*H*).

Ethyl (*E*)-3-phenylpent-2-enoate S2

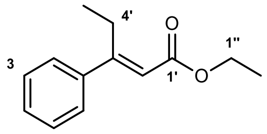

Following General Procedure **A**, ethyl 2-(diethoxyphosphoryl)acetate (2.98 mL, 15.0 mmol), sodium hydride (600 mg, 15.0 mmol, 60% in mineral oil), propiophenone (2 mL, 15.0 mmol) in THF gave the crude product, which was purified by column chromatography (Petroleum ether 40/60 : diethyl ether (97:3)) to give the title compound as a colourless oil (1.18 g, 39%) with spectroscopic data in accordance with the literature;[5]

^{1}H-NMR (400 MHz, $CDCl_3$) δ_H: **1.08** (3H, t, $^3J_{HH}$ = 7.5, C(5`)$H_3$), **1.32** (3H, t, $^3J_{HH}$ = 7.1, $OCH_2CH_3$), **3.11** (2H, q, $^3J_{HH}$ = 7.5, C(4`)H_2), **4.21** (2H, q, $^3J_{HH}$ = 7.1, OCH_2CH_3), **6.02** (1H, s, C(2`)*H*), **7.34 – 7.39** (3H, m, ArC(2,4,6)*H*), **7.43 – 7.47** (2H, m, ArC(3,5)*H*).

Ethyl (*E*)-4-methyl-3-phenylpent-2-enoate S3

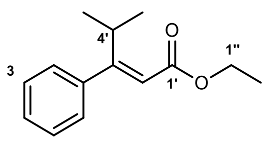

Following General Procedure **A**, isobutyrophenone (4.50 mL, 30.0 mmol), sodium hydride (1.20 g, 30.0 mmol, 60% in mineral oil), ethyl 2-(diethoxyphosphoryl)acetate (5.95 ml, 30.0 mmol) in THF gave the crude product, which was purified by column chromatography (petroleum ether 40/60 : diethyl ether (98:2)) to give the title compound as a colourless oil (2.20 g, 34%) with spectroscopic data in accordance with the literature;[6]

^{1}H NMR (400 MHz, $CDCl_3$) δ_H: **1.09** (6H, d, $^3J_{HH}$ = 7.0, 2 × C(5`)$H_3$), **1.30** (3H, t, $^3J_{HH}$ = 7.1, $OCH_2H_3$), **4.11** (1H, hept, $^3J_{HH}$ = 7.1, C(4`)*H*), **4.20** (2H, q, $^3J_{HH}$ = 7.2 , OCH_2CH_3), **5.70** (1H, s, C(2`)*H*), **7.18 − 7.22** (2H, m, ArC(2,6)*H*), **7.28 − 7.35** (3H, m, ArC(3,4,5)*H*).

Methyl 2-(9*H*-fluoren-9-ylidene)acetate S4

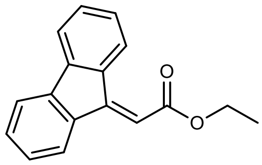

Following General Procedure **A**, 9-fluorenone (3.60 g, 20.0 mmol), sodium hydride (800 mg, 20 mmol, 60% in mineral oil), methyl 2-(dimethoxyphosphoryl)acetate (3.27 mL, 20 mmol) in THF gave the crude product, which was purified by column chromatography (petroleum ether 40/60 : diethyl ether (95:5)) to give the title compound as a colourless oil (3.61 g, 77%) with spectroscopic data in accordance with the literature;[7]

^{1}H NMR (500 MHz, $CDCl_3$) δ_H: **3.88** (3H, s, OC*H*$_3$), **6.75** (1H, s, C=C*H*CO_2Me), **7.25 – 7.27** (1H, m, ArC*H*), **7.29 – 7.35** (1H, m, ArC*H*), **7.38 – 7.41** (2H, m, ArC*H*), **7.59 – 7.68** (3H, m, ArC*H*), **8.89** (1H, appd, *J* = 7.8, ArC*H*); ^{13}C{^{1}H} NMR (126 MHz, $CDCl_3$) δ_C: **52.0** (O*C*H$_3$), **113.5** (C=*C*HCO_2Me), **119.7** (Ar*C*H), **119.9** (Ar*C*H), **121.4** (Ar*C*H), **127.6** (Ar*C*H), **128.2** (Ar*C*H), **129.3** (Ar*C*H), **130.8** (Ar*C*H), **131.1** (Ar*C*H), **135.2** (Ar*C*), **138.9** (Ar*C*), **140.9** (Ar*C*), **142.6** (Ar*C*), **148.7** (*C*=CHCO_2Me), **166.9** (*C*O_2Me).

Ethyl 3,3-diphenylacrylate S5

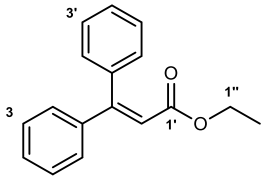

Following General Procedure **A**, benzophenone (5.47 g, 30.0 mmol), sodium hydride (1.20 g, 30 mmol, 60% in mineral oil), ethyl 2-(diethoxyphosphoryl)acetate (5.95 mL, 30 mmol) in THF gave the crude product, which was purified by column chromatography (petroleum ether 40/60 : diethyl ether (95:5)) to give the title compound as a colourless oil (6.63 g, 88%) with spectroscopic data in accordance with the literature;[8]

^{1}H NMR (400 MHz, $CDCl_3$) δ_H: **1.10** (3H, t, $^3J_{HH}$ = 7.2, C*H*$_3$), **4.04** (2H, q, $^3J_{HH}$ = 7.2, C*H*$_2$), **6.36** (1H, s, C(2``)*H*), **7.20 − 7.24** (2H, m, ArC*H*), **7.27 − 7.34** (5H, m, ArC*H*), **7.36 − 7.39** (3H, m, ArC*H*).

Ethyl (*E*)-3-(*p*-tolyl)but-2-enoate S6

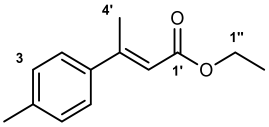

Following General Procedure **A**, ethyl 2-(diethoxyphosphoryl)acetate (3.96 mL, 20.0 mmol), sodium hydride (800 mg, 20.0 mmol, 60% in mineral oil), 1-(*p*-tolyl)ethan-1-one (2.68 mL, 20.0 mmol) in THF gave the crude product, which was purified by column chromatography (Petroleum ether 40/60 : diethyl ether (97:3)) to give the title compound as a colourless oil (2.45 g, 60%) with spectroscopic data in accordance with the literature;[5]

^{1}H-NMR (400 MHz, $CDCl_3$) δ_H: **1.32** (3H, t, $^3J_{HH}$ = 7.1, C(2``)$H_3$), **2.37** (3H, s, ArCC$H_3$), **2.57** (3H, d, $^4J_{HH}$ = 1.3, C(4`)$H_3$), **4.21** (2H, q, $^3J_{HH}$ = 7.1, C(1``)H_2), **6.13** (1H, q, $^3J_{HH}$ = 1.3, C(2`)*H*), **7.18** (2H, d, $^3J_{HH}$ = 7.5, ArC(2,6)*H*), **7.39** (2H, d, $^3J_{HH}$ = 7.5, ArC(3,5)*H*).

Ethyl (*E*)-3-(4-(tert-butyl)phenyl)but-2-enoate S7

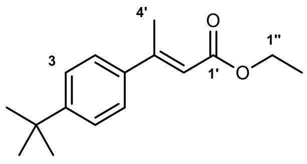

Following General Procedure **A**, ethyl 2-(diethoxyphosphoryl)acetate (1.12 mL, 5.67 mmol), sodium hydride (227 mg, 5.67 mmol, 60% in mineral oil), 1-(4-(tert-butyl)phenyl)ethan-1-one (1.00 g, 5.67 mmol) in THF gave the crude product, which was purified by column chromatography (petroleum ether 40/60 : diethyl ether (98:2)) to give the title compound as a colourless oil (839 mg, 60%);

ν_{max} (film): 2963 (C-H), 1713 (C=O), 1626 (C=C); ^{1}H-NMR (400 MHz, $CDCl_3$) δ_H: 1.32 (3H, t, $^3J_{HH}$ = 7.1, OCH_2CH_3) **1.33** (9H, s, ArCC(CH_3)$_3$), **2.58** (3H, d, $^4J_{HH}$ = 1.3, C(4`)$H_3$), **4.22** (2H, q, $^3J_{HH}$ = 7.1, OC$H_2$), **6.15** (1H, m, C(2`)*H*), **7.38 – 7.46** (4H, m, ArC*H*); ^{13}C{^{1}H}-NMR (126 MHz, $CDCl_3$) δ_C: **14.5** (ArC(*C*H_3)$_3$), **17.9** (OCH_2*C*H_3), **31.4** (*C*(4`)$H_3$), **34.8** (Ar*C*($CH_3$)$_3$), **59.9** (O*C*$H_2$), **116.5** (*C*(2`)H), **125.6** (Ar*C*(2,6)H), **126.2** (Ar*C*(3,5)H), **139.3** (Ar*C*(1)), **152.5** (ArC(4)(*C*H_3)$_3$), **155.4** (*C*(3`)), **167.2** (*C*O_2Et); HRMS (ESI$^+$) $C_{16}H_{22}O_2$ [M+Na]$^+$: found 269.1512, required 269.1507 (+1.9 ppm).

Ethyl (*E*)-3-(4-nitrophenyl)but-2-enoate S8

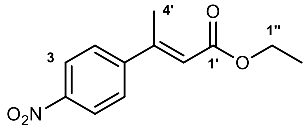

Following General Procedure **A**, ethyl 2-(diethoxyphosphoryl)acetate (6.00 ml, 30.2 mmol), sodium hydride (1.21 g, 30.2 mmol, 60% in mineral oil), 4-nitroacetophenone (4.99 g, 30.2 mol) in THF gave the crude product, which was purified by column chromatography (petroleum ether 40/60 : ethyl acetate (95:5)) to give the title compound as a colourless oil (3.00 g, 42%) with spectroscopic data in accordance with the literature;[9]

^{1}H-NMR (400 MHz, $CDCl_3$) δ_H: **1.33** (3H, t, $^3J_{HH}$ = 7.1, OCH_2CH_3), **2.59** (3H, d, $^4J_{HH}$ = 1.4 , C(4`)$H_3$), **4.24** (2H, q, $^3J$ = 7.1, OC$H_2$), **6.18** (1H, q, $^4J_{HH}$ = 1.4, C(2`)*H*), **7.59 – 7.63** (2H, m, ArC(2,6)*H*), **8.21 – 8.25** (2H, m, ArC(3,5)*H*).

Ethyl (*E*)-3-(4-fluorophenyl)but-2-enoate S9

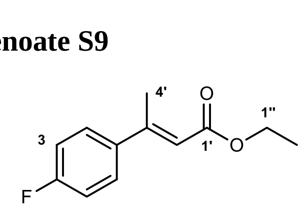

Following General Procedure **A**, ethyl 2-(diethoxyphosphoryl)acetate (1.98 mL, 10.0 mmol), sodium hydride (400 mg, 10.0 mmol, 60% in mineral oil), 4-fluoroacetophenone (1.21 mL, 10.0 mmol) in THF gave the crude product, which was purified by column chromatography (petroleum ether 40/60 : diethyl ether (97:3)) gave the title compound as a colourless oil (966 mg, 46%) with spectroscopic data in accordance with the literature;[10]

^{1}H-NMR (400 MHz, $CDCl_3$) δ_H: **1.32** (3H, t, $^{3}J_{HH}$ = 7.1 , C(2``)$H_3$), **2.56** (2H, d, $^{4}J_{HH}$ = 1.3 , C(4`)$H_3$), **4.21** (2H, q, $^{3}J_{HH}$ = 7.1 , C(1``)H_2), **6.09** (1H, q, $^{4}J_{HH}$ = 1.3 , C(2`)*H*), **7.03 – 7.09** (2H, m, C(2, 6)*H*), **7.43 – 7.48** (2H, m, C(3, 5)*H*); ^{19}F{^{1}H}-NMR (376 MHz, $CDCl_3$) δ_F: **–113.0** (s, ArC*F*).

Ethyl (*E)*-3-(4-bromophenyl)but-2-enoate S10

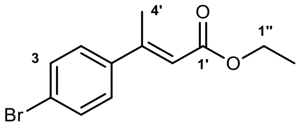

Following General Procedure **A**, ethyl 2-(diethoxyphosphoryl)acetate (1.98 mL, 10.0 mmol), sodium hydride (400 MHzg, 10.0 mmol, 60% in mineral oil), 4-bromoacetophenone (1.99 g, 10.0 mmol) in THF gave the crude product, which was purified by column chromatography (petroleum ether 40/60 : diethyl ether (97:3)) gave the title compound as a colourless oil (1.07 g, 40%) with spectroscopic data in accordance with the literature;[5]

^{1}H NMR ($CDCl_3$, 400 MHz) δ_H: **1.31** (3H, t, $^{3}J_{HH}$ = 7.1, OCH_2H_3), **2.54** (2H, d, $^{4}J_{HH}$ = 1.3, C(4`)$H_3$), **4.21** (2H, q, $^{3}J_{HH}$ = 7.1, OC$H_2$), **6.11** (1H, q, $^{4}J_{HH}$ = 1.3, C(2`)*H*), **7.32 – 7.36** (2H, m, ArC(2,6)*H*), **7.48 – 7.52** (2H, m, ArC(3,5)*H*).

Ethyl (*E*)-3-(4-iodophenyl)but-2-enoate S11

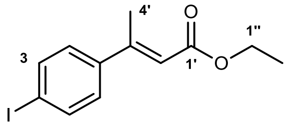

Following General Procedure **A**, ethyl 2-(diethoxyphosphoryl)acetate (2.42 mL, 12.2 mmol), sodium hydride (488 mg, 12.2 mmol, 60% in mineral oil), 4-iodoacetophenone (3.00 g, 12.2 mmol) in THF gave the crude product, which was purified by column chromatography (petroleum ether 40/60 : diethyl ether (98:2)) to give the title compound as a colourless oil (2.18 g, 57%);

ν_{max} (film): 2976 (C-H), 1717 (C=O), 1638 (C=C); ^{1}H-NMR (400 MHz, $CDCl_3$) δ_H: **1.31** (3H, t, $^{3}J_{HH}$ = 7.1, OCH_2H_3), **2.53** (3H, d, $^{4}J_{HH}$ = 1.4, C(4`)$H_3$), **4.21** (2H, q, $^{3}J_{HH}$ = 7.1, OC$H_2$$CH_3$), **6.11** (1H, q, $^{4}J_{HH}$ = 1.4, C(2`)*H*), **7.19 – 7.22** (2H, m, ArC(2,6)*H*), **7.68 – 7.72** (2H, m, ArC(3,5)*H*); ^{13}C{^{1}H}-NMR (126 MHz, $CDCl_3$) δ_C: **14.4** (OCH_2*C*H_3), **17.8** (*C*(4`)$H_3$), **60.1** (O*C*$H_2$), **95.1** (Ar*C*(4)I), **117.7** (*C*(2`)H), **128.2** (Ar*C*(2,6)H), **137.7** (Ar*C*(3,5)H), **141.7** (Ar*C*(1)), **154.3** (*C*(3`)), **166.7** (*C*O_2Et); HRMS (ESI$^+$) $C_{12}H_{13}IO_2$ $[M+Na]^+$: found 338.9843, required 338.9852 (–2.7 ppm).

Ethyl (*E*)-3-(5-bromo-2-methoxyphenyl)but-2-enoate S12

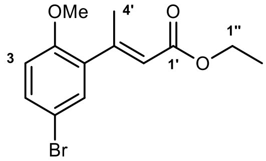

Following General Procedure **A**, ethyl 2-(diethoxyphosphoryl)acetate (2.08 mL, 10.5 mmol), sodium hydride (419 mg, 20.0 mmol, 60% in mineral oil), 1-(5-bromo-2-methoxyphenyl)ethan-1-one (2.40 g, 10.5 mmol) in THF gave the crude product, which was purified by column chromatography (petroleum ether 40/60 : diethyl ether (97:3)) gave the title compound as a colourless oil (1.87 g, 60%);

ν_{max} (film): 2978 (C-H), 2938 (C-H), 2841 (C-H), 1713 (C=O), 1634 (C=C); ^{1}H NMR (400 MHz, $CDCl_3$) δ_H: **1.30** (3H, t, $^{3}J_{HH}$ = 7.1, $OCH_2C$$H_3$), **2.45** (3H, d, $^{4}J_{HH}$ = 1.4, C(4`)H_3), **3.80** (3H, s, ArCOCH_3),

4.20 (2H, q, $^3J_{HH}$ = 7.1, OCH_2CH$_3$), **5.87** (1H, q, $^4J_{HH}$ = 1.4, C(2`)$H$), **6.77** (1H, d, $^3J_{HH}$ = 8.7, ArC(3)$H$), **7.25** (1H, t, $^4J_{HH}$ = 2.5, ArC(6)$H$), **7.38** (1H, dd, $^3J_{HH}$ = 8.8, $^4J_{HH}$ = 2.5, ArC(4)$H$); $^{13}$C NMR (126 MHz, CDCl$_3$) $\delta_C$: **14.5** (OCH$_2$$C$H$_3$), **19.7** ($C$(4`)H$_3$), **55.9** (ArCOCH$_3$), **60.0** (OCH$_2$CH$_3$), **112.8** (C(2`)H), **112.8** (Ar$C$(3)H), **120.1** (Ar$C$(5)Br), **131.5** (Ar$C$(4)H), **132.0** (Ar$C$(1)), **135.1** (Ar$C$(6)H), **155.0** ($C$(3`)), **155.7** (ArC(2)OCH$_3$), **166.6** (CO$_2$Et); HRMS (ASAP) $C_{13}H_{15}BrO_3$ [M+H]$^+$: found 299.0282, required 299.0283 (–0.3 ppm).

Ethyl (*E*)-3-(3-bromophenyl)but-2-enoate S13

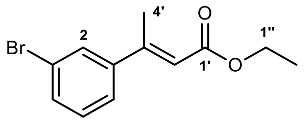

Following General Procedure **A**, ethyl 2-(diethoxyphosphoryl)acetate (3.97 mL, 20.0 mmol), sodium hydride (800 mg, 20.0 mmol, 60% in mineral oil), 3-bromoacetophenone (2.65 mL, 20.0 mmol) in THF gave the crude product, which was purified by column chromatography (petroleum ether 40/60 : diethyl ether (97:3)) gave the title compound as a colourless oil (3.19 g, 59%);

ν_{max} (film): 2980 (C-H), 2928 (C-H), 1715 (C=O), 1632 (C=C); ^{1}H-NMR (400 MHz, CDCl$_3$) δ_H: **1.32** (3H, t, $^3J_{HH}$ = 7.1, OCH$_2$CH_3), **2.54** (3H, d, $^4J_{HH}$ = 1.3, C(4`)$H_3$), **4.22** (2H, q, $^3J_{HH}$ = 7.1, OC$H_2$), **6.11** (1H, q, $^4J_{HH}$ = 1.3, C(2`)H), **7.24** (1H, t, $^3J_{HH}$ = 7.9, ArC(5)H), **7.39** (1H, ddd, $^3J_{HH}$ = 7.8, $^4J_{HH}$ = 1.8, 1.0, ArC(6)H), **7.48** (1H, ddd, $^3J_{HH}$ = 7.9 , $^4J_{HH}$ = 2.0 , 1.0 , ArC(4)H), **7.48** (1H, t, $^4J_{HH}$ = 1.9, ArC(2)H); ^{13}C{^{1}H}-NMR (126 MHz, CDCl$_3$) δ_C: **14.5** (OCH$_2$$CH_3$), **18.0** ($C$(4`)H$_3$), **60.2** (O$C$H$_2$), **118.4** ($C$(2`)H), **122.8** (Ar$C$(3)Br), **125.1** (Ar$C$(6)H), **129.6** (Ar$C$(2)H), **130.2** (Ar$C$(5)H), **132.0** (Ar$C$(4)H), **144.5** (ArC(1)), **153.9** (C(3`)), **166.7** (CO$_2$Et); HRMS (ASAP) $C_{12}H_{13}BrO_2$ [M+H]$^+$: found 269.0174, required 269.0177 (–1.1 ppm).

Ethyl (*E*)-3-cyclopropyl-3-phenylacrylate S14

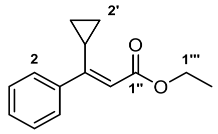

Following General Procedure **A**, ethyl 2-(diethoxyphosphoryl)acetate (5.95 mL, 30.0 mmol), sodium hydride (1.20 g, 30.0 mmol, 60% in mineral oil), cyclopropyl(phenyl)methanone (4.15 mL, 30.0 mmol) in THF gave the crude product, which was purified by column chromatography (Petroleum ether 40/60 : diethyl ether (99.5:0.5)) gave the title compound as a colourless oil (785 g, 12%) with spectroscopic data in accordance with the literature;[5]

^{1}H-NMR (400 MHz, CDCl$_3$) δ_H: **0.48** (2H, m, C(2`, 3`)H_AH$_B$), **0.89** (2H, m, C(2`, 3`)H$_A$$H_B$), **1.31** (3H, t, $^3J_{HH}$ = 7.1 , C(2```)H_3), **3.14** (1H, ttd, $^3J_{HH}$ = 8.5 , 5.4 , $^4J_{HH}$ = 0.9 , C(1`)H), **4.22** (2H, q, $^3J_{HH}$ = 7.1 , C(1```)H_2), **5.79** (1H, d, $^3J_{HH}$ = 0.9 , C(2``)H), **7.11 – 7.16** (2H, m, C(2, 6)H), **7.28 – 7.33** (3H, m, C(3, 4, 5)H).

C. 2. ii. Allylic alcohols

(*E*)-3-phenylbut-2-en-1-ol S15

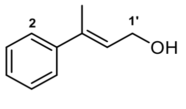

Following General Procedure **B**, ethyl (*E*)-3-phenylbut-2-enoate **S1** (2.52 g, 13.2 mmol), DIBAL-H (29.1 mL, 29.1 mmol, 1 M in hexanes) in Et_2O gave the title compound as a colourless oil (1.71 g, 87%) with spectroscopic data in accordance with the literature;[11]

^{1}H-NMR (500 MHz, $CDCl_3$) δ_H: **1.33** (1H, t, $^3J_{HH}$ = 5.6, CH_2OH), **2.09** (3H, d, $^4J_{HH}$ = 1.5, CH_3), **4.38** (2H, tq, $^3J_{HH}$ = 6.7, $^4J_{HH}$ = 1.4, CH_2OH), **5.98** (1H, tq, $^3J_{HH}$ = 6.7, 1.4, C(2`)*H*), **7.24 − 7.29** (1H, m, ArC(4)*H*), **7.31 − 7.36** (2H, m, ArC(2,6)*H*), **7.40 − 7.43** (2H, m, ArC(3,5)*H*).

(*E*)-3-phenylpent-2-en-1-ol S16

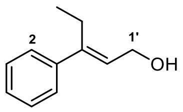

Following General Procedure M, ethyl (*E*)-3-phenylpent-2-enoate **S2** (1.11 g, 5.41 mmol), DIBAL-H (11.9 mL, 11.9 mmol, 1 M in hexanes) in Et_2O gave the title compound as a colourless oil (636 mg, 72%) with spectroscopic data in accordance with the literature;[5]

^{1}H-NMR (500 MHz, $CDCl_3$) δ_H: **1.00** (3H, t, $^3J_{HH}$ = 7.6, CH_3), **1.53** (1H, brs, O*H*), **2.55** (2H, q, $^3J_{HH}$ = 7.6, C(4`)$H_2$), **4.36** (2H, d, $^3J_{HH}$ = 6.8, C(1`)H_2), **5.84** (1H, t, $^3J_{HH}$ = 6.8, C(2`)*H*), **7.25 – 7.29** (1H, m, ArC(2,6)*H*), **7.31 – 7.35** (2H, m, ArC(3,5)*H*), **7.36 – 7.40** (1H, m, ArC(4)*H*).

(*E*)-4-methyl-3-phenylpent-2-en-1-ol S17

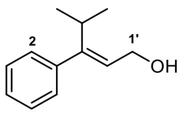

Following General Procedure **B**, ethyl (*E*)-4-methyl-3-phenylpent-2-enoate **S3** (918 mg, 4.21 mmol), DIBAL-H (9.25 mL, 9.25 mmol, 1 M in hexanes) in Et_2O gave the title compound as a colourless oil (617 mg, 82%) with spectroscopic data in accordance with the literature;[12]

^{1}H-NMR (400 MHz, $CDCl_3$) δ_H: **1.06** (6H, d, $^3J_{HH}$ = 7.0, C(5`)$H_3$), **1.47** (1H, brs, O*H*), **3.03** (2H, hpt, $^3J_{HH}$ = 7.0, C(4`)*H*), **4.37** (2H, d, $^3J_{HH}$ = 6.7, CH_2), **5.49** (1H, t, $^3J_{HH}$ = 6.7, C(2`)*H*), **7.16 – 7.20** (2H, m, ArC(2,6)*H*), **7.25 – 7.33** (3H, m, ArC(3,4,5)*H*).

2-(9H-Fluoren-9-ylidene)ethan-1-ol S18

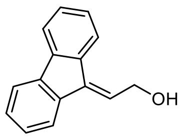

Following General Procedure **B**, methyl 2-(9H-fluoren-9-ylidene)acetate **S4** (2.89 g, 11.5 mmol), DIBAL-H (46.6 mL, 46.6 mmol, 1 M in hexanes) in Et_2O gave the title compound as a colourless oil (2.12 g, 88%) with spectroscopic data in accordance with the literature;[13]

^{1}H NMR (500 MHz, $CDCl_3$): **1.77** (1H, brs, CH_2OH), **4.99** (2H, d, $^3J_{HH}$ = 5.9, CH_2OH), **6.82** (1H, t, $^3J_{HH}$ = 6.8, C=C*H*CH_2OH), **7.27 – 7.39** (4H, m, ArC*H*), **7.26 – 7.30** (5H, m, ArC*H*), **7.58 – 7.62** (1H, m, ArC*H*), **7.66 – 7.71** (2H, m, ArC*H*), **7.72 – 7.77** (1H, m, ArC*H*); ^{13}C{^{1}H} NMR (126 MHz, $CDCl_3$) δ_C: **60.7** (O*C*H_2OH), **119.8** (C=*C*H), **120.1** (Ar*C*H), **120.3** (Ar*C*H), **125.3** (Ar*C*H), **127.3** (Ar*C*H), **127.3**

(Ar*C*H), **128.3** (Ar*C*H), **128.5** (Ar*C*H), **128.6** (Ar*C*H), **136.1** (Ar*CC*), **136.8** (Ar*C*), **138.9** (Ar*C*), **139.3** (Ar*C*), **141.2** (Ar*C*).

3,3-Diphenylprop-2-en-1-ol S19

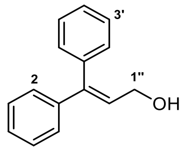

Following General Procedure **B**, ethyl 3,3-diphenylacrylate **S5** (2.89 g, 11.5 mmol), DIBAL-H (46.6 mL, 46.6 mmol, 1 M in hexanes) in Et_2O gave the title compound as a colourless oil (2.12 g, 88%) with spectroscopic data in accordance with the literature;[14]

^{1}H NMR (500 MHz, $CDCl_3$): **1.68** (1H, brs, CH_2OH), **4.23** (2H, d, $^3J_{HH}$ = 6.8, CH_2OH), **6.27** (1H, t, $^3J_{HH}$ = 6.8, C(2``)*H*CH_2OH), **7.16 – 7.20** (2H, m, ArC*H*), **7.26 – 7.30** (5H, m, ArC*H*), **7.34 – 7.42** (3H, m, ArC*H*).

(*E*)-3-(*p*-Tolyl)but-2-en-1-ol S20

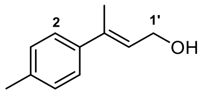

Following General Procedure **B**, ethyl (*E*)-3-(p-tolyl)but-2-enoate **S6** (2.44 g, 11.9 mmol), DIBAL-H (26.3 mL, 26.3 mmol, 1 M in hexanes) in Et_2O gave the title compound as a colourless oil (1.16 g, 60%) with spectroscopic data in accordance with the literature;[15]

^{1}H-NMR (500 MHz, $CDCl_3$) δ_H: **1.39** (1H, m, O*H*), **2.07** (3H, s, C(4`)$H_3$), **2.35** (3H, s, ArC$H_3$), **4.36** (2H, t, $^3J_{HH}$ = 6.1, OC$H_2$), **5.96** (1H, tq, $^3J_{HH}$ = 6.8, $^4J_{HH}$ = 1.4, C(2`)*H*), **7.14** (2H, d, $^3J_{HH}$ = 8.0, ArC(2,6)*H*), **7.32** (2H, d, $^3J_{HH}$ = 8.0, ArC(3,5)*H*).

(*E*)-3-(4-(Tert-butyl)phenyl)but-2-en-1-ol S21

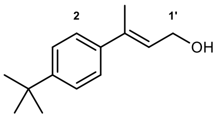

Following General Procedure **B**, ethyl (*E*)-3-(4-(tert-butyl)phenyl)but-2-enoate **S7** (789 mg, 3.21 mmol), DIBAL-H (7.06 mL, 7.06 mmol, 1 M in hexanes) in Et_2O gave the title compound as a colourless solid (596 mg, 98%);

ν_{max} (film): 3312 (OH), 2961, 2903, 2877, 1645, 1508, 1462, 1447, 1395, 1362, 1272, 1202, 1119, 1063, 999, 824; ^{1}H-NMR (500 MHz, $CDCl_3$) δ_H: **1.33** (9H, s, ArC(CH_3)$_3$), **1.34** (1H, brs, O*H*), **2.08** (3H, q, $^4J_{HH}$ = 1.4, C(4`)$H_3$), **4.37** (2H, t, $^3J_{HH}$ = 5.9, OC$H_2$OH), **5.98** (1H, tq, $^3J_{HH}$ = 5.9, $^4J_{HH}$ = 1.4 , C(2`)*H*), **7.35 – 7.37** (4H, m, ArC*H*); ^{13}C{^{1}H}-NMR (126 MHz, $CDCl_3$) δ_C: **16.1** (*C*(4`)$H_3$), **31.5** (ArC(*C*$H_3$)$_3$), **34.6** (Ar*C*($CH_3$)$_3$), **60.1** (*C*$H_2$OH), **125.3** (Ar*C*(3,5)H), **125.6** (Ar*C*(2,6)H), **125.9** (*C*(2`)H), **137.8** (*C*(3`)), **140.0** (Ar*C*(1)), **150.5** (Ar*C*(4)C(CH_3)$_3$); HRMS (ASAP) $C_{14}H_{20}O$ $[M-H]^-$: found 203.1433, required 203.1436 (–1.5 ppm).

(*E*)-3-(4-Nitrophenyl)but-2-en-1-ol S22

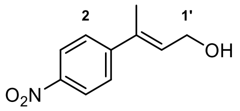

Following General Procedure **B**, ethyl (*E*)-3-(4-nitrophenyl)but-2-enoate **S8** (3.00 g, 12.7 mmol), DIBAL-H (28.0 mL, 28.0 mmol, 1 M in hexanes) in Et_2O gave the title compound as a dark red oil (2.30 g, 94%) with spectroscopic data in accordance with the literature;[16]

^{1}H-NMR (500 MHz, $CDCl_3$) δ_H: **1.47** (1H, s, CH_2OH), **2.11** (3H, d, $^4J_{HH}$ = 1.2, C*H*$_3$), **4.42** (2H, d, $^3J_{HH}$ = 6.6, C*H*$_2$), **6.11** (1H, tq, $^3J_{HH}$ = 6.4, $^4J_{HH}$ = 1.4, C(2`)*H*), **7.54** (2H, m, ArC(2,6)*H*), **8.19** (2H, m, ArC(3,5)*H*).

(*E*)-3-(4-Fluorophenyl)but-2-en-1-ol S23

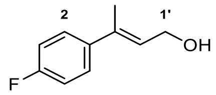

Following General Procedure **B**, ethyl (*E*)-3-(4-fluorophenyl)but-2-enoate **S9** (953 mg, 4.58 mmol), DIBAL-H (10.1 mL, 10.1 mmol, 1 M in hexanes) in Et_2O gave the title compound as a colourless oil (577 mg, 76%) with spectroscopic data in accordance with the literature;[15]

^{1}H-NMR (500 MHz, $CDCl_3$) δ_H: **1.34** (1H, brs, CH_2OH), **2.07** (3H, d, $^4J_{HH}$ = 1.4, C*H*$_3$), **4.36** (2H, t, $^3J_{HH}$ = 6.7, C*H*$_2$OH), **5.92** (1H, tq, $^3J_{HH}$ = 6.7, $^4J_{HH}$ = 1.4, C(2`)*H*CH$_2$OH), **7.01** (2H, d, $^3J_{HH}$ = 8.9, ArC(2,6)*H*), **7.36** (2H, d, $^3J_{HH}$ = 8.9, ArC(3,5)*H*).

(*E*)-3-(4-Bromophenyl)but-2-en-1-ol S24

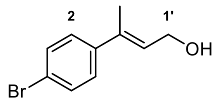

Following General Procedure **B**, ethyl (*E*)-3-(4-bromophenyl)but-2-enoate **S10** (959 mg, 3.56 mmol), DIBAL-H (7.84 mL, 7.84 mmol, 1 M in hexanes) in Et_2O gave the title compound as a colourless oil (440 mg, 54%);

ν_{max} (film): 3292, 3211, 3196, 2916, 2855, 1481, 1447; ^{1}H-NMR (500 MHz, $CDCl_3$) δ_H: **1.42** (1H, brs, CH_2OH), **2.05** (3H, d, $^4J_{HH}$ = 1.4, C*H*$_3$), **4.36** (2H, t, $^3J_{HH}$ = 5.6, C*H*$_2$OH), **5.96** (1H, tq, $^3J_{HH}$ = 6.7, $^4J_{HH}$ = 1.4, C(2`)*H*), **7.27** (2H, d, $^3J_{HH}$ = 8.8, ArC(2,6)*H*), **7.36** (2H, d, $^3J_{HH}$ = 8.8, ArC(3,5)*H*); $^{13}$C{$^1$H}-NMR (126 MHz, $CDCl_3$) $\delta_C$: **16.1** (*C*H$_3$), **60.1** (*C*(1`)H$_2$OH), **121.3** (Ar*C*(4)Br), **127.2** (*C*(2`)H), **127.6** (Ar*C*(3,5)H), **131.5** (Ar*C*(2,6)H), **136.9** (*C*(3`)), **141.8** (Ar*C*(1)); HRMS (ESI$^+$) $C_{10}H_{11}BrO$ $[M-H_2O]^+$: found 208.9961, required 208.9960.

(*E*)-3-(4-Iodophenyl)but-2-en-1-ol S25

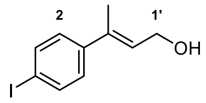

Following General Procedure **B**, ethyl (*E*)-3-(4-iodophenyl)but-2-enoate **S11** (1.73 mg, 5.48 mmol), DIBAL-H (12.1 mL, 12.1 mmol, 1 M in hexanes) in Et_2O gave the title compound as a white solid (1.07 g, 72%);

mp. 173 – 175 °C ($CHCl_3$); ν_{max} (film): 3422, 3009, 2922, 1722, 1647, 1493, 1447; ^{1}H-NMR (500 MHz, $CDCl_3$): δ_H **1.42** (1H, brs, O*H*), **2.04** (3H, q, $^4J_{HH}$ = 1.4, C*H*$_3$), **4.35** (2H, t, $^3J_{HH}$ = 6.6, C*H*$_2$OH), **5.97**

(1H, tq, $^3J_{HH}$ = 6.6, $^4J_{HH}$ = 1.4, C(2`)*H*), **7.14** (2H, d, $^3J_{HH}$ = 8.6, ArC(2,6)*H*), **7.64** (2H, d, $^3J_{HH}$ = 8.6, ArC(3,5)*H*); $^{13}$C{$^1$H} NMR (126 MHz, $CDCl_3$): **16.0** ($CH_3$), **60.1** (*C*(1`)H_2OH), **92.9** (Ar*C*(4)I), **127.2** (*C*(2`)H), **127.8** (Ar*C*(2,6)H), **136.9** (*C*(3`)), **137.5** (Ar*C*(3,5)H), **142.4** (Ar*C*(1)); HRMS (ESI$^+$) $C_{10}H_{11}IO$ [M−H_2O]$^+$: found 256.9823, required 256.9822 (+0.4 ppm).

(*E*)-3-(5-Bromo-2-methoxyphenyl)but-2-en-1-ol S26

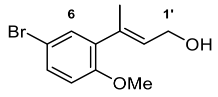

Following General Procedure **B**, ethyl (*E*)-3-(5-bromo-2-methoxyphenyl)but-2-enoate **S12** (1.49 g, 4.98 mmol), DIBAL-H (11.0 mL, 11.0 mmol, 1 M in hexanes) in Et_2O gave the title compound as a colourless oil (1.00 g, 78%);

ν_{max} (film): 3314, 2936, 2839, 1587, 1483, 1460, 1439, 1287, 1236, 1179, 1140, 1105, 1063, 1024; ^{1}H-NMR (500 MHz, $CDCl_3$) δ_H: **1.35** (1H, brs, CH_2O*H*), **1.99** (3H, d, $^4J_{HH}$ = 1.5, C(4`)*H*$_3$), **3.79** (3H, s, ArCOC*H*$_3$), **4.32** (2H, ddd, $^3J_{HH}$ = 6.7, 5.7, C*H*$_2$OH), **5.68** (1H, tq, $^3J_{HH}$ = 6.7, $^4J_{HH}$ = 1.5, C(2`)*H*CH_2OH), **6.73** (1H, d, $^3J_{HH}$ = 8.7, ArC(3)*H*), **7.25** (1H, d, $^4J_{HH}$ = 2.6, C(6)*H*), **7.33** (1H, dd, $^3J_{HH}$ = 8.7 , $^4J_{HH}$ = 2.6 , C(4)*H*); ^{13}C{^{1}H}-NMR (126 MHz, $CDCl_3$) δ_C: **17.2** (*C*(4`)H), **55.8** (ArCO*C*$H_3$), **59.7** (*C*(1`)H_2OH), **112.5** (Ar*C*(5)Br), **122.8** (Ar*C*(3)H), **129.3** (Ar*C*(1)), **131.0** (*C*(2`)H), **132.2** (Ar*C*(6)H), **134.0** (Ar*C*(4)H), **138.6** (*C*(3`)), **156.7** (Ar*C*(2)OCH_3); HRMS (ASAP) $C_{11}H_{12}BrO_2$ [M–H]$^+$: found 255.0015, found 255.0015 (+0.0 ppm).

(*E*)-3-(3-Bromophenyl)but-2-en-1-ol S27

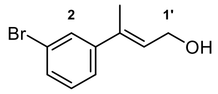

Following General Procedure **B**, ethyl (*E*)-3-(3-bromophenyl)but-2-enoate **S13** (2.65 g, 9.85 mmol), DIBAL-H (21.7 mL, 21.7 mmol, 1 M in hexanes) in Et_2O gave the title compound as a yellow oil (2.02 g, 90%);

ν_{max} (film): 3420, 2922, 2855, 1722, 1686, 1657, 1591, 1558, 1474, 1418, 1375, 1248, 1157, 1063; ^{1}H-NMR (500 MHz, $CDCl_3$) δ_H: **1.45** (1H, brs, CH_2O*H*), **2.05** (3H, d, $^4J_{HH}$ = 1.3, C*H*$_3$), **4.37** (2H, t, $^3J_{HH}$ = 6.6, C*H*$_2$), **5.97** (1H, tq, $^3J_{HH}$ = 6.6, $^4J_{HH}$ = 1.3, C(2`)*H*), **7.19** (1H, t, $^3J_{HH}$ = 7.8, ArC(5)*H*), **7.32** (1H, ddd, $^3J_{HH}$ = 7.8, $^4J_{HH}$ = 1.8, 1.0, ArC(6)*H*), **7.39** (1H, ddd, $^3J_{HH}$ = 7.8, $^4J_{HH}$ = 1.8, 1.0, ArC(4)*H*), **7.54** (1H, t, $^4J_{HH}$ = 1.8, ArC(2)*H*); ^{13}C{^{1}H}-NMR (126 MHz, $CDCl_3$) δ_C: **16.1** (*C*H_3), **60.0** (*C*(1`)$H_2$), **122.7** (*C*(2`)H), **124.5** (Ar*C*(3)Br), **127.9** (Ar*C*(6)H), **129.1** (Ar*C*(5)H), **129.9** (Ar*C*(4)H), **130.3** (Ar*C*(2)H), **136.6** (*C*(3`)), **145.1** (ArC(1)); HRMS (ASAP) $C_{10}H_{10}BrO$ [M-H]$^+$: found 224.9908, required 224.9910 (–0.9 ppm).

(*E*)-3-cyclopropyl-3-phenylprop-2-en-1-ol S28

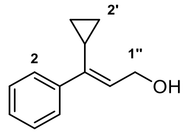

Following General Procedure M, ethyl (*E*)-3-cyclopropyl-3-phenylacrylate **S14** (1.00 g, 4.62 mmol), DIBAL-H (10.2 mL, 10.2 mmol, 1 M in hexanes) in Et_2O gave the title compound as a colourless oil (314 mg, 39%) with spectroscopic data in accordance with the literature;[17]

^{1}H-NMR (500 MHz, $CDCl_3$) δ_H: **0.32 – 0.37** (2H, m, C(5`)$H_AH_B$, **0.79 – 0.85** (2H, m, C(5`)H_AH_B), **1.42** (1H, brs, O*H*), **1.77** (1H, m, C(6)*H*), **4.54** (2H, m, C(1`)$H_2$), **5.87** (1H, td, $^{3}J_{HH}$ = 6.6 , $^{4}J_{HH}$ = 1.7 , C(2`)*H*), **7.21 – 7.26** (1H, m, C(2, 6)*H*), **7.27 – 7.32** (2H, m, C(3, 5)*H*), **7.32 – 7.35** (1H, m, C(4)*H*).

C. 2. iii. Allylic bromides

(*E*)-(4-Bromobut-2-en-2-yl)benzene S29

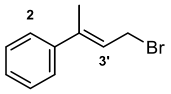

Following General Procedure **C**, (*E*)-3-phenylbut-2-en-1-ol **S15** (1.37 g, 9.25 mmol), phosphorous tribromide (357 µl, 3.80 mmol) in Et_2O gave the title compound as a pale yellow liquid (1.61 g, 7.65 mmol, 83%) with spectroscopic data in accordance with the literature.[18] The product was used immediately without further purification as it is prone to decomposition;

^{1}H-NMR (400 MHz, $CDCl_3$) δ_H: **2.15** (3H, d, $^{3}J_{HH}$ = 1.4, CH_3), **4.21** (2H, d, $^{3}J_{HH}$ = 8.5, CH_2), **6.10** (1H, tq, $^{3}J_{HH}$ = 8.5, $^{4}J_{HH}$ = 1.4, C(3`)*H*), **7.27 – 7.38** (3H, m, ArC(2,4,6)*H*), **7.38 – 7.45** (2H, m, ArC(3,5)*H*).

(*E*)-(1-Bromopent-2-en-3-yl)benzene S30

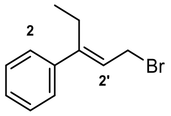

Following General Procedure **C**, (*E*)-3-ethylcinnamyl alcohol **S16** (630 mg, 3.88 mmol), phosphorous tribromide (150 µl, 1.59 mmol) in Et_2O gave the title compound as a pale yellow liquid (748 mg, 86%) with spectroscopic data in accordance with the literature.[18] The product was used immediately without further purification as it is prone to decomposition;

ν_{max} (film): 2955, 2922, 2853, 1632, 1495, 1456, 1377, 1200, 1032; ^{1}H-NMR (400 MHz, $CDCl_3$) δ_H: **1.05** (3H, t, $^{3}J_{HH}$ = 7.6, CH_3), **2.63** (2H, q, $^{3}J_{HH}$ = 7.6, C(4`)$H_2$), **4.20** (2H, d, $^{3}J_{HH}$ = 8.6, C(1`)H_2), **5.96** (1H, t, $^{3}J_{HH}$ = 8.5, C(2`)*H*), **7.27 – 7.31** (1H, m, ArC(4)*H*), **7.32 – 7.36** (2H, m, ArC(2,6)*H*), **7.37 – 7.40** (2H, m, ArC(3,5)*H*); $^{13}$C{$^{1}$H}-NMR (126 MHz, $CDCl_3$) $\delta_C$: **13.5** (*C*$H_3$), **23.0** (*C*(4`)H_2), **29.2** (*C*(1`)$H_2$Br), **122.7** (*C*(2`)H), **126.6** (Ar*C*(2,6)H), **127.9** (Ar*C*(4)H), **128.5** (Ar*C*(3,5)H), **141.4** (*C*(3`)), **148.1** (Ar*C*(1)); HRMS: product decomposed before further analysis was performed.

(*E*)-(1-Bromo-4-methylpent-2-en-3-yl)benzene S31

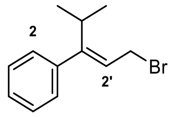

Following General Procedure **C**, (*E*)-4-methyl-3-phenylpent-2-en-1-ol **S17** (1.58 g, 8.96 mmol), phosphorous tribromide (345 µl, 3.68 mmol) in Et_2O gave the title compound as a brown liquid (1.93 g, 90%) with spectroscopic data in accordance with the literature.[18] The product was used immediately without further purification as it is prone to decomposition;

ν_{max} (film) 2963, 2928, 2872, 1636, 1599, 1464, 1441, 1362, 1105, 1074, 1030; ^{1}H-NMR (400 MHz, $CDCl_3$) δ_H: **1.10** (6H, d, $^3J_{HH}$ = 7.0, 2 × CH_3), **3.14** (1H, hpt, $^3J_{HH}$ = 7.0, C(4`)*H*($CH_3$)$_2$), **4.18** (2H, d, $^3J_{HH}$ = 8.7, C$H_2$Br), **5.63** (1H, t, $^3J_{HH}$ = 8.7, C(2`)*H*CH_2Br), **7.15 – 7.20** (2H, m, ArC(2,6)*H*), **7.27 – 7.34** (3H, m, ArC(3,4,5)*H*); ^{13}C{^{1}H}-NMR (126 MHz, $CDCl_3$) δ_C: **21.7** (2 × *C*H_3), **28.2** (*C*H(CH_3)$_2$), **29.4** (*C*(1`)$H_2$Br), **124.0** (*C*(2`)HCH_2Br), **127.1** (Ar*C*(4)H), **127.8** (Ar*C*(2,6)H), **128.4** (Ar*C*(3,5)H), **141.6** (*C*(3`)), **153.1** (Ar*C*(1)); HRMS: product decomposed before further analysis was performed.

(3-Bromoprop-1-ene-1,1-diyl)dibenzene S33

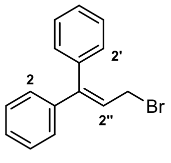

Following General Procedure **C**, 3,3-diphenylprop-2-en-1-ol l **S19** (1.03 g, 4.90 mmol), phosphorous tribromide (189 µl, 2.01 mmol) in Et_2O gave the title compound as a brown liquid (1.08 g, 81%) with spectroscopic data in accordance with the literature.[19] The product was used immediately without further purification as it is prone to decomposition;

^{1}H NMR (500 MHz, $CDCl_3$): **4.06** (1H, d, $^3J_{HH}$ = 8.6, C=CHCH_2Br), **6.34** (1H, t, $^3J_{HH}$ = 8.6, C=C*H*CH_2Br), **7.23 – 7.48** (10H, m, ArC*H*).

(*E*)-1-(4-Bromobut-2-en-2-yl)-4-methylbenzene S34

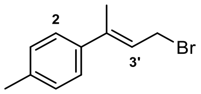

Following General Procedure **C**, (*E*)-3-(p-tolyl)but-2-en-1-ol **S20** (1.16 g, 7.13 mmol), phosphorous tribromide (275 µl, 2.92 mmol) in Et_2O gave the title compound as a pale yellow liquid (1.58 g, 99%) with spectroscopic data in accordance with the literature.[20] The product was used immediately without further purification as it is prone to decomposition;

^{1}H-NMR (400 MHz, $CDCl_3$) δ_H: **2.14** (3H, d, $^4J_{HH}$ = 1.4, C(1`)$H_3$), **2.36** (3H, s, ArC$H_3$), **4.21** (2H, d, $^3J_{HH}$ = 8.6, C$H_2$), **6.08** (1H, td, $^3J_{HH}$ = 8.6, $^4J_{HH}$ = 1.4, C(3`)*H*), **7.13 – 7.17** (2H, m, ArC(2,6)*H*), **7.30 – 7.33** (2H, m, ArC(3,5)*H*).

(*E*)-1-(4-Bromobut-2-en-2-yl)-4-(tert-butyl)benzene S35

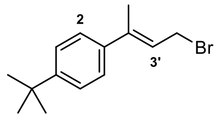

Following General Procedure **C**, (*E*)-3-(4-(tert-butyl)phenyl)but-2-en-1-ol **S21** (596 mg, 2.92 mmol), phosphorous tribromide (115 µl, 1.20 mmol) in Et_2O gave the title compound as a pale yellow liquid (732 mg, 94%). The product was used immediately without further purification as it is prone to decomposition;

ν_{max} (film): 2961, 2905, 2866, 1508, 1460, 1433, 1400, 1362, 1269, 1202, 1113, 1015; ^{1}H-NMR (400 MHz, $CDCl_3$) δ_H: **1.32** (9H, s, ArC(CH_3)$_3$), **2.14** (3H, d, $^4J_{HH}$ = 1.4, C(1`)$H_3$), **4.21** (2H, d, $^3J_{HH}$ = 8.6, C$H_2$), **6.09** (1H, tq, $^3J_{HH}$ = 8.6, $^4J_{HH}$ = 1.4, C(3`)*H*), **7.36** (4H, apps, ArC*H*); ^{13}C{^{1}H}-NMR (101 MHz, $CDCl_3$) δ_C: **15.6** (*C*(1`)$H_3$), **29.9** (*C*(4`)H_2Br), **31.4** (ArC(*C*H_3)$_3$), **34.7** (Ar*C*(CH_3)$_3$), **122.2** (*C*(3`)H), **125.4** (Ar*C*(3,5)H), **125.7** (Ar*C*(2,6)H), **139.3** (*C*(2`)), **141.4** (Ar*C*(1)), **151.1** (Ar*C*(4)); HRMS (NSI$^+$) $C_{14}H_{19}Br$ [M+H]$^+$: found 269.0721, required 269.0729 (–3.0 ppm).

(*E*)-1-(4-Bromobut-2-en-2-yl)-4-nitrobenzene S36

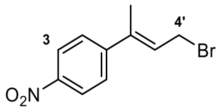

Following General Procedure **C**, (*E*)-3-(4-nitrophenyl)but-2-en-1-ol **S22** (2.32 g, 11.2 mmol), phosphorous tribromide (430 µl, 4.58 mmol) in Et_2O gave the title compound as a dark purple liquid (2.10 g, 74%) with spectroscopic data in accordance with the literature.[16] The product was used immediately without further purification as it is prone to decomposition;

^{1}H-NMR (400 MHz, $CDCl_3$) δ_H: **2.18** (3H, d, $^4J_{HH}$ = 1.4, C*H*$_3$), **4.18** (2H, d, $^3J_{HH}$ = 8.5, C*H*$_2$), **6.22** (1H, td, $^3J_{HH}$ = 8.5, $^4J_{HH}$ = 1.4, C(3`)*H*), **7.55** (2H, d, $^3J_{HH}$ = 9.0, ArC(2,6)*H*), **8.20** (2H, d, $^3J_{HH}$ = 9.0, ArC(3, 5)*H*).

(*E*)-1-(4-bromobut-2-en-2-yl)-4-fluorobenzene S37

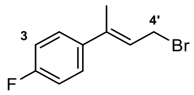

Following General Procedure **C**, (*E*)-3-(4-fluorophenyl)but-2-en-1-ol **S23** (577 mg, 3.47 mmol), phosphorous tribromide (135 µl, 1.42 mmol) in Et_2O gave the title compound as a yellow-brown oil (712 mg, 90%). The product was used immediately without further purification as it is prone to decomposition;

^{1}H-NMR (400 MHz, $CDCl_3$) δ_H: **2.13** (3H, d, $^4J_{HH}$ = 1.3, C*H*$_3$), **4.19** (2H, d, $^3J_{HH}$ = 8.5, C*H*$_2$), **6.04** (1H, td, $^3J_{HH}$ = 8.5, $^4J_{HH}$ = 1.4, C(3`)*H*), **7.00 – 7.05** (2H, m, ArC(2,6)*H*), **7.35 – 7.39** (2H, m, ArC(3,5)*H*); $^{13}$C{$^1$H}-NMR (126 MHz, $CDCl_3$) $\delta_C$: **15.9** (*C*H$_3$), **29.4** (*C*H$_2$Br), **115.3** (2C, d, $^2J_{CF}$ = 21.4, Ar*C*(3,5)H, **122.8** (*C*(3`)H), **127.7** (2C, d, $^3J_{CF}$ = 8.0, Ar*C*(2,6)H), **138.3** (Ar*C*(4)), **140.6** (*C*(2`)), **162.6** (d, $^1J_{CF}$ = 247.0, Ar*C*(4)F); HRMS: product decomposed before analysis was performed.

(*E*)-1-(4-bromobut-2-en-2-yl)-4-bromobenzene S38

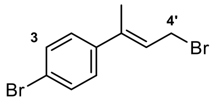

Following General Procedure **C**, (*E*)-3-(4-bromophenyl)but-2-en-1-ol **S24** (440 mg, 1.94 mmol), phosphorous tribromide (75 µl, 0.80 mmol) in Et_2O gave the title compound as a brown oil (487 mg, 87%) with spectroscopic data in accordance with the literature.[18] The product was used immediately without further purification as it is prone to decomposition;

^{1}H-NMR (500 MHz, $CDCl_3$) δ_H: **2.12** (3H, d, $^4J_{HH}$ = 1.4, C*H*$_3$), **4.17** (2H, d, $^3J_{HH}$ = 8.5, C*H*$_2$Br), **6.08** (1H, td, $^3J_{HH}$ = 8.5, $^4J_{HH}$ = 1.4, C(3`)*H*), **7.27** (2H, d, $^3J_{HH}$ = 8.5 , ArC(2,6)*H*), **7.45** (2H, d, $^3J_{HH}$ = 8.5, ArC(3,5)*H*); $^{13}$C{$^1$H}-NMR (126 MHz, $CDCl_3$) $\delta_C$: **15.7** (*C*H$_3$), **29.1** (*C*(4`)H$_2$Br), **122.0** (Ar*C*(4)Br), **123.5** (*C*(3`)H), **127.7** (Ar*C*(2,6)H), **131.6** (Ar*C*(3,5)H), **140.4** (Ar*C*(1)), **141.1** (*C*(2`)).

(*E*)-1-(4-Iodobut-2-en-2-yl)-4-bromobenzene S39

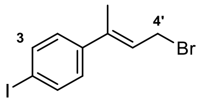

Following General Procedure **C**, (*E*)-3-(4-iodophenyl)but-2-en-1-ol **S25** (1.07 g, 3.92 mmol), phosphorous tribromide (151 µl, 1.61 mmol) in Et_2O gave the title compound as a brown oil (1.13 g, 85%). The product was used immediately without further purification as it is prone to decomposition;

ν_{max} (film): 3028, 2961, 2926, 2868, 2808, 1674, 1634, 1582, 1508, 1481, 1454, 1396, 1200, 1070, 1003; ^{1}H-NMR (400 MHz, $CDCl_3$) δ_H: **2.11** (3H, d, $^4J_{HH}$ = 1.4, C*H*$_3$), **4.17** (2H, d, $^3J_{HH}$ = 8.5, C*H*$_2$Br), **6.08** (1H, tq, $^3J_{HH}$ = 8.5, $^4J_{HH}$ = 1.4, C(3`)*H*CH$_2$Br), **7.14** (2H, d, $^3J_{HH}$ = 8.6, ArC(2,6)*H*), **7.66** (2H, d, $^3J_{HH}$ = 8.6, ArC(3,5)*H*); $^{13}$C{$^1$H}-NMR (126 MHz, $CDCl_3$) $\delta_C$: **15.6** (*C*H$_3$), **29.0** (*C*(4`)H$_2$Br), **93.5** (Ar*C*(4)I), **123.5** (*C*(3`)H), **127.9** (Ar*C*(2,6)H), **137.6** (Ar*C*(3,5)H), **140.5** (Ar*C*(1)), **141.8** (*C*(2`)); HRMS (ASAP) $C_{10}H_{10}BrI$ [M]$^+$: found 335.9004, required 335.9905 (–0.3 ppm).

(*E*)-4-Bromo-2-(4-bromobut-2-en-2-yl)-1-methoxybenzene S40

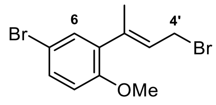

Following General Procedure **C**, (*E*)-3-(5-bromo-2-methoxyphenyl)but-2-en-1-ol **S26** (1.00 g, 3.89 mmol), phosphorous tribromide (150 µl, 1.59 mmol) in Et_2O gave the title compound as a pale yellow liquid (955 mg, 77%). The product was used immediately without further purification as it is prone to decomposition;

ν_{max} (film): 3001, 2959, 2936, 2839, 1589, 1485, 1460, 1439, 1396, 1288, 1261, 1233, 1202, 1180, 1134, 1061, 1026; ^{1}H-NMR (400 MHz, $CDCl_3$) δ_H: **2.05** (3H, d, $^4J_{HH}$ = 1.5, C(1`)*H*$_3$), **3.80** (3H, s, ArC*H*$_3$), **4.13** (2H, d, $^3J_{HH}$ = 8.5, C*H*$_2$Br), **5.81** (1H, tq, $^3J_{HH}$ = 8.5, 1.5, C(3`)*H*), **6.74** (1H, d, $^3J_{HH}$ = 8.7, C(3)*H*), **7.24** (1H, d, $^4J_{HH}$ = 2.6, ArC(6)*H*), **7.34** (1H, dd, $^3J_{HH}$ = 8.7, 2.6, Ar*C*(4)*H*); ^{13}C{^{1}H}-NMR (126 MHz, $CDCl_3$) δ_C: **16.8** (*C*(1`)H$_3$), **28.7** (*C*(4`)H$_2$Br), **55.8** (O*C*H$_3$), **112.6** (Ar*C*(3)H), **112.8** (Ar*C*(5)H), **125.8** (*C*(3`)H), **131.4** (Ar*C*(4)H), **132.0** (Ar*C*(6)H), **135.1** (*C*(2`)), **140.5** (Ar*C*(1)), **156.0** (Ar*C*(2)OCH$_3$); HRMS: product decomposed before analysis was performed.

(*E*)-1-Bromo-3-(4-bromobut-2-en-2-yl)benzene S41

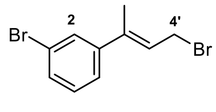

Following General Procedure **C**, (*E*)-3-(3-bromophenyl)but-2-en-1-ol **S27** (1.94 g, 8.56 mmol), phosphorous tribromide (330 µl, 3.51 mmol) in Et_2O gave the title compound as a pale yellow liquid (2.05 g, 83%). The product was used immediately without further purification as it is prone to decomposition;

ν_{max} (film): 2951, 2922, 2853, 1688, 1634, 1591, 1558, 1474, 1408, 1377, 1275, 1260, 1200, 1078, 1059; ^{1}H-NMR (400 MHz, $CDCl_3$) δ_H: **2.12** (3H, d, $^4J_{HH}$ = 1.4, C*H*$_3$), **4.17** (2H, d, $^3J_{HH}$ = 8.5, C*H*$_2$Br), **6.08** (1H, tq, $^3J_{HH}$ = 8.5, $^4J_{HH}$ = 1.4, C(3`)*H*CH$_2$Br), **7.21** (1H, appt, $^3J_{HH}$ = 7.8, ArC(5)*H*), **7.32** (1H, ddd, $^3J_{HH}$ = 7.8, $^4J_{HH}$ = 1.8, $^4J_{HH}$ = 1.1, ArC(6)*H*), **7.41** (1H, ddd, $^3J_{HH}$ = 7.8, $^4J_{HH}$ = 1.8 , $^4J_{HH}$ = 1.1, ArC(4)*H*), **7.54** (1H, t, $^4J_{HH}$ = 1.9, ArC(2)*H*); ^{13}C{^{1}H}-NMR (126 MHz, $CDCl_3$) δ_C: **15.7** (*C*H$_3$), **28.9** (*C*(4`)H$_2$Br), **122.7** (*C*(3`)H), **124.1** (Ar*C*(3)Br), **124.7** (Ar*C*(6)H), **129.2** (Ar*C*(2)H), **130.0** (Ar*C*(5)H), **130.8** (Ar*C*(4)H), **140.1** (*C*(2`)), **144.5** (Ar*C*(1)); HRMS: product decomposed before analysis was performed.

C. 2. iv. Allylic tertiary amines

(*E*)-1-(3-Phenylbut-2-en-1-yl)pyrrolidine S42

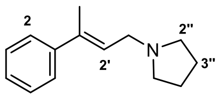

Following General Procedure **D**, pyrrolidine (1.85 mL, 22.2 mmol), (*E*)-(4-bromobut-2-en-2-yl)benzene **S29** (931 mg, 4.41 mmol) in THF gave the title compound as a yellow oil (784 mg, 88%);

ν_{max} (film): 2959, 2926, 2906, 2874, 2776, 1599, 1493, 1445, 1375, 1314, 1240, 1200, 1138, 1057, 1026; ^{1}H-NMR (400 MHz, $CDCl_3$) δ_H: **1.79 – 1.83** (4H, m, C(3``,4``)H_2), **2.07** (3H, d, $^4J_{HH}$ = 1.4, CH_3), **2.56 – 2.62** (4H, m, C(2``,5``)H_2), **3.31** (2H, d, $^3J_{HH}$ = 6.7, C(1`)$H_2$), **5.95** (1H, tq, $^3J_{HH}$ = 6.7, $^4J_{HH}$ = 1.4, C(2`)*H*), **7.21 – 7.26** (1H, m, ArC(4)*H*), **7.28 – 7.34** (2H, m, ArC(2,6)*H*), **7.39 – 7.43** (2H, m, ArC(3,5)*H*); ^{13}C{^{1}H}-NMR (126 MHz, $CDCl_3$) δ_C: **16.3** (*C*H_3), **23.6** (*C*(3``,4``)H_2), **54.3** (*C*(1`)H_2), **54.5** (*C*(2``,5``)H_2), **125.7** (*C*(2`)H), **125.9** (Ar*C*(4)H), **127.0** (Ar*C*(2,6)H), **128.3** (Ar*C*(3,5)H), **136.5** (*C*(3`)), **143.6** (Ar*C*(1)); HRMS (NSI$^+$) $C_{14}H_{19}N$ [M+H]$^+$: found 202.1589, required 202.1590 (–0.4 ppm).

(*E*)-1-(3-Phenylpent-2-en-1-yl)pyrrolidine S43

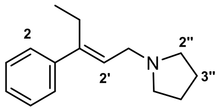

Following General Procedure **D**, pyrrolidine (682 μL, 8.31 mmol), (*E*)-(1-bromopent-2-en-3-yl)benzene **S30** (748 mg, 3.32 mmol) in THF gave the title compound as a yellow oil (498 mg, 70%);

ν_{max} (film): 2963, 2926, 2872, 2776, 1493, 1458, 1445, 1373, 1346, 1140, 1063, 1032; ^{1}H-NMR ($CDCl_3$, 400 MHz) δ_H: **0.98** (3H, t, $^3J_{HH}$ = 7.5, CH_3), **1.81** (4H, p, $^3J_{HH}$ = 3.1, C(3``,4``)H_2), **2.55** (2H, q, $^3J_{HH}$ = 7.5, C(4`)$H_2$$CH_3$), **2.56 – 2.59** (4H, m, C(2``,5``)H_2), **3.29** (2H, d, $^3J_{HH}$ = 6.7, C(1`)$H_2$), **5.80** (1H, t, $^3J_{HH}$ = 6.7 , C(2`)*H*), **7.23** (1H, tt, $^3J_{HH}$ = 7.4, $^4J_{HH}$ = 2.2, ArC(4)*H*), **7.29 – 7.33** (2H, t, $^3J_{HH}$ = 7.4, ArC(3,5)*H*), **7.36 – 7.39** (2H, m, ArC(2,6)*H*); ^{13}C{^{1}H}-NMR (126 MHz, $CDCl_3$) δ_C: **13.6** (*C*H_3), **23.3** (C(4`)H_2CH_3), **23.6** (*C*(3``,4``)H_2), **54.0** (*C*(1`)H_2), **54.4** (C(2``,5``)H_2), **125.6** (*C*(2`)H), **126.5** (Ar*C*(3,5)H), **126.9** (Ar*C*(4)H), **128.3** (Ar*C*(2,6)H), **142.6** (*C*(3`)), **143.1** (Ar*C*(1)); HRMS (ESI$^+$) $C_{15}H_{21}N$ [M+H]$^+$: found 216.1740, required 216.1747 (–3.2 ppm).

(*E*)-1-(4-Methyl-3-phenylpent-2-en-1-yl)pyrrolidine S44

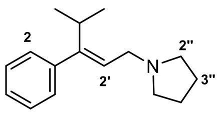

Following General Procedure **D**, pyrrolidine (1.16 mL, 14.1 mmol), (*E*)-(1-bromo-4-methylpent-2-en-3-yl)benzene **S31** (676 mg, 2.83 mmol) in THF gave the title compound as a brown oil (561 mg, 86%);

ν_{max} (film) 2961, 2872, 2778, 1491, 1458, 1441, 1362, 1346, 1140, 1119, 1072, 1032; ^{1}H-NMR (500 MHz, $CDCl_3$) δ_H: **1.04** (6H, d, $^3J_{HH}$ = 7.0, 2 × CH_3), **1.78 – 1.82** (4H, m, C(3``,4``)H_2), **2.54 – 2.61** (4H, m, C(2``,5``)H_2), **3.06** (1H, hpt, $^3J_{HH}$ = 7.0, C*H*(CH_3)$_2$), **3.30** (2H, d, $^3J_{HH}$ = 6.6 , C(1`)$H_2$), **5.43** (1H, t, $^3J_{HH}$ = 6.6 , C(2`)*H*), **7.16 – 7.19** (2H, m, ArC(2,6)*H*), **7.20 – 7.25** (1H, m, ArC(4)*H*), **7.25 – 7.29** (2H, m, ArC(3,5)*H*); ^{13}C{^{1}H}-NMR (126 MHz, $CDCl_3$) δ_C: **21.8** (2 × *C*H_3), **23.6** (*C*(3``,4``)H_2), **29.6** (*C*H(CH_3)$_2$), **53.2** (*C*(1`)H_2), **54.3** (*C*(2``,5``)H_2), **126.3** (*C*(2`)H), **126.4** (Ar*C*(4)H), **127.6** (Ar*C*(2,6)H),

128.7 (Ar*C*(3,5)H), **142.9** (C(3`)), **148.2** (ArC(1)); HRMS (ESI$^+$) $C_{16}H_{23}N$ [M+H]$^+$: found 230.1900, required 230.1903 (–1.3 ppm).

1-(3,3-Diphenylallyl)pyrrolidine S46

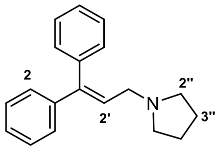

Following General Procedure **D**, pyrrolidine (3.01 mL, 36.6 mmol), (3-bromoprop-1-ene-1,1-diyl)dibenzene **S33** (2.00 g, 7.32 mmol) in THF gave the title compound as a brown oil (1.60 g, 83%) with spectroscopic data in accordance with the literature;[21]

^{1}H-NMR (400 MHz, CDCl3) δ_H: **1.72 – 1.83** (4H, m, C(3``,4``)H_2), **2.46 – 2.55** (4H, m, C(2``,5``)H_2), **3.19** (2H, d, $^3J_{HH}$ = 6.8, C(1`)$H_2$), **6.28** (1H, t, $^3J_{HH}$ = 6.8, C(2`)*H*), **7.14 – 7.40** (10H, m, ArC*H*).

(*E*)-1-(3-Phenylbut-2-en-1-yl)piperidine S47

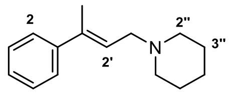

Following General Procedure **D**, piperidine (467 µL, 4.73 mmol), (*E*)-(4-bromobut-2-en-2-yl)benzene **S29** (400 mg, 1.89 mmol) in THF gave the title compound as a brown oil (314 mg, 77%);

ν_{max} (film): 2978, 2857, 2810, 2790, 1503, 1444, 1323, 1324, 1201, 1081; ^{1}H-NMR (400 MHz, $CDCl_3$) δ_H: **1.44** (2H, brs, C(4``)$H_2$), **1.57 – 1.64** (4H, m, C(3``,5``)$H_2$), **2.06** (3H, d, $^4J_{HH}$ = 1.4, C$H_3$), **2.40 – 2.49** (4H, m, C(2``,6``)H_2), **3.15** (2H, appdd, $^3J_{HH}$ = 6.8, $^4J_{HH}$ = 1.1, C(1`)$H_2$), **5.93** (1H, tq, $^3J_{HH}$ = 6.8, $^4J_{HH}$ = 1.4, C(2`)*H*), **7.24** (1H, td, $^3J_{HH}$ = 7.4, $^4J_{HH}$ = 1.3, ArC(4)*H*), **7.29 – 7.33** (2H, m, ArC(2,6)*H*), **7.40 – 7.43** (2H, m, ArC(3,5)*H*); ^{13}C-NMR (126 MHz, $CDCl_3$) δ_C: **16.3** (*C*H_3), **24.5** (*C*(6)H_2), **26.2** (*C*(3,5)H_2), **54.9** (*C*(2,6)H_2), **57.7** (*C*(1`)$H_2$), **125.5** (*C*(2`)H), **125.8** (Ar*C*(2,6)H), **127.0** (Ar*C*(4)H), **128.3** (Ar*C*(3,5)H), **137.0** (*C*(3`)), **143.5** (Ar*C*(1)); HRMS (ESI$^+$) $C_{15}H_{21}N$ [M+H]$^+$: found 216.1742, required 216.1747 (−2.3 ppm).

(*E*)-1-(3-Phenylbut-2-en-1-yl)azepane S48

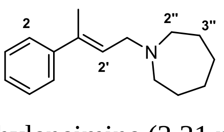

Following General Procedure **D**, hexamethyleneimine (3.31 mL, 29.4 mmol), (*E*)-(4-bromobut-2-en-2-yl)benzene **S29** (1.24 g, 5.87 mmol) in THF gave the title compound as a brown oil (1.18 g, 87%);

ν_{max} (film): 2920, 2853, 2812, 2768, 1493, 1356, 1321, 1152, 1078, 1026; ^{1}H-NMR (500 MHz, $CDCl_3$) δ_H: **1.60 – 1.65** (4H, m, C(4``,5``)H_2), **1.65 – 1.71** (4H, m, C(3``,6``)H_2), **2.05 – 2.06** (3H, m, CH_3), **2.66** (4H, t, $^3J_{HH}$ = 5.7 , C(2``,6``)H_2), **3.29** (2H, d, $^3J_{HH}$ = 6.7, C(1``)$H_2$), **5.93** (1H, tq, $^3J_{HH}$ = 6.7, $^4J_{HH}$ = 1.4, C(2`)*H*), **7.21 – 7.25** (1H, m, ArC(4)*H*), **7.29 – 7.34** (2H, m, ArC(2,6)*H*), **7.39 – 7.43** (2H, m, ArC(3,5)*H*); $^{13}$C{$^1$H}-NMR (126 MHz, $CDCl_3$) $\delta_C$: **16.3** (*C*$H_3$), **27.0** (*C*(4``,5``)$H_2$), **28.3** (*C*(3``,6``)$H_2$), **56.1** (*C*(2``,7``)H_2), **57.1** (*C*(1`)$H_2$), **125.8** (Ar*C*(2,6)H), **126.4** (*C*(2`)H), **126.9** (Ar*C*(4)H), **128.3** (Ar*C*(3,5)H), **136.6** (*C*(3`)), **143.7** (Ar*C*(1)); HRMS (ESI$^+$) $C_{16}H_{23}N$ [M+H]$^+$: found 230.1898, required 230.1903 (–2.2 ppm).

***N,N*-Dimethyl-3,3-diphenylprop-2-en-1-amine S49**

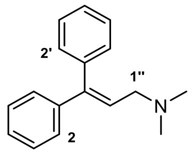

Following General Procedure **D**, dimethylamine (9.85 mL, 19.7 mmol, 2 M in THF), (3-bromoprop-1-ene-1,1-diyl)dibenzene **S33** (1.08 g, 3.94 mmol) in THF gave the title compound as a brown oil (533 mg, 57%);

ν_{max} (film): 3055, 3024, 2970, 2940, 2853, 2814, 2764, 1668, 1597, 1493, 1443, 1366, 1022; ^{1}H-NMR (500 MHz, $CDCl_3$) δ_H: **2.23** (6H, s, 2 × NCH_3), **3.00** (2H, d, $^{3}J_{HH}$ = 6.8, C(1``)$H_2$NMe$_2$), **6.20** (1H, t, $^{3}J_{HH}$ = 6.8, C(2``)*H*), **7.14 – 7.18** (2H, m, ArC*H*), **7.21 – 7.26** (3H, m, ArC*H*), **7.26 – 7.30** (2H, m, ArC*H*), **7.30 – 7.35** (1H, m, ArC*H*), **7.35 – 7.40** (2H, m, ArC*H*); ^{13}C{^{1}H}-NMR (126 MHz, $CDCl_3$) δ_C: **45.6** (N(*C*H$_3$)$_2$), **58.5** (C1``), **127.1** (*C*(2`)H), **127.3** (Ar*C*H), **127.4** (Ar*C*H), **127.5** (Ar*C*H), **128.3** (Ar*C*H), **128.3** (Ar*C*H), **130.0** (Ar*C*H), **139.9** (C(3``)Ph$_2$), **142.3** (ArC(1`)), **143.7** (ArC(1)); HRMS (ESI$^+$) $C_{17}H_{19}N$ [M+H]$^+$: found 238.1583, required 238.1590 (−2.9 ppm).

(*E*)-*N,N*-Dimethyl-3-phenylbut-2-en-1-amine S50

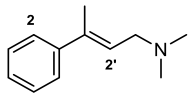

Following General Procedure **D**, dimethylamine (12.5 mL, 25.0 mmol, 2 M in THF), (*E*)-(4-bromobut-2-en-2-yl)benzene **S29** (1.06 g, 5.00 mmol) in THF gave the title compound as a brown oil (787 mg, 90%) with spectroscopic data in accordance with the literature;[22]

^{1}H-NMR (400 MHz, $CDCl_3$) δ_H: **2.07** (3H, s, C(4`)$H_3$), **2.29** (6H, s, 2 × NC$H_3$), **3.10** (2H, d, $^{3}J_{HH}$ = 6.8, C(1`)H_2NMe$_2$), **5.88** (1H, t, $^{3}J_{HH}$ = 6.8, C(2`)*H*), **7.21 – 7.26** (1H, m, ArC(4)*H*), **7.29 – 7.36** (2H, m, ArC(2,6)*H*), **7.38 – 7.44** (2H, m, ArC(3,5)*H*).

(*E*)-4-(3-Phenylbut-2-en-1-yl)morpholine S51

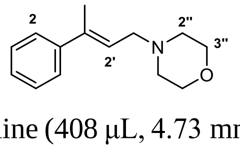

Following General Procedure **D**, morpholine (408 µL, 4.73 mmol), (*E*)-(4-bromobut-2-en-2-yl)benzene **S29** (400 mg, 1.89 mmol) in THF gave the title compound as a brown oil (297 mg, 72%);

ν_{max} (film): 2955, 2853, 2805, 1495, 1445, 1354, 1294, 1273, 1115, 1003; ^{1}H-NMR (400 MHz, $CDCl_3$) δ_H: **2.08** (3H, d, $^{4}J_{HH}$ = 1.3, CH_3), **2.52** (4H, brs, C(2``,6``)H_2), **3.19** (2H, d, $^{3}J_{HH}$ = 6.8, C(1`)H_2), **3.74** (4H, t, $^{3}J_{HH}$ = 4.7, C(3``,5``)H_2), **5.88** (1H, tq, $^{3}J_{HH}$ = 6.8, $^{4}J_{HH}$ = 1.4, C(2`)*H*), **7.22 – 7.25** (1H, m, ArC(4)*H*), **7.30 – 7.34** (2H, m, ArC(2,6)*H*), **7.39 – 7.42** (2H, m, ArC(3,5)*H*); ^{13}C{^{1}H}-NMR (126 MHz, $CDCl_3$) δ_C: **16.4** (*C*H$_3$), **53.9** (*C*(2``,6``)H$_2$), **57.3** (*C*(1`)H$_2$), **67.2** (*C*(3``,5``)H$_2$), **124.2** (*C*(2`)H), **125.8** (Ar*C*(2,6)H), **127.2** (Ar*C*(4)H), **128.4** (Ar*C*(3,5)H), **138.1** (*C*(3`)), **143.3** (Ar*C*(1)); HRMS (ESI$^+$) $C_{14}H_{19}NO$ [M+H]$^+$: found 218.1535, required 218.1539 (−1.8 ppm).

(*E*)-1-(3-(*p*-Tolyl)but-2-en-1-yl)pyrrolidine S52

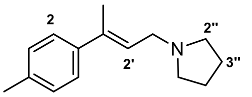

Following General Procedure **D**, pyrrolidine (887 μL, 10.8 mmol), (*E*)-1-(4-bromobut-2-en-2-yl)-4-methylbenzene **S34** (973 mg, 4.32 mmol) in THF gave the title compound as a yellow oil (462 mg, 50%);

ν_{max} (film): 2963, 2922, 2874, 2778, 1684, 1653, 1512, 1458, 1445, 1375, 1346, 1314, 1128, 1057, 1032, 1018; ^{1}H-NMR (400 MHz, $CDCl_3$) δ_H: **1.78 – 1.83** (4H, m, C(3``,4``)H_2), **2.05** (3H, s, C(4`)H_3), **2.33** (3H, s, ArCH_3), **2.55 – 2.59** (4H, m, C(2``,5``)H_2), **3.28** (2H, d, $^3J_{HH}$ = 6.7, C(1`)$H_2$), **5.91** (1H, t, $^3J_{HH}$ = 6.7, C(2`)*H*), **7.12** (2H, d, $^3J_{HH}$ = 8.0, ArC(3,5)*H*), **7.31** (2H, t, $^3J_{HH}$ = 8.0, ArC(2,6)*H*); ^{13}C{^{1}H}-NMR (126 MHz, $CDCl_3$) δ_C: **16.3** (*C*(4`)H_3), **21.2** (Ar*C*H_3), **23.6** (C(3``,4``)H_2), **54.3** (*C*(1`)H_2), **54.4** (*C*(2``,5``)H_2), **125.0** (*C*(2`)H), **125.7** (Ar*C*(3,5)H), **126.0** (Ar*C*(4)$CH_3$), **129.0** (Ar*C*(2,6)H), **136.6** (*C*(3`)), **140.7** (Ar*C*(1)); HRMS (ESI$^+$) $C_{15}H_{21}N^+$ [M+H]$^+$: found 216.1741, required 216.1747 (–2.8 ppm).

(*E*)-1-(3-(4-(Tert-butyl)phenyl)but-2-en-1-yl)pyrrolidine S53

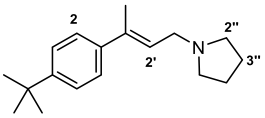

Following General Procedure **D**, pyrrolidine (1.12 mL, 13.7 mmol), (*E*)-1-(4-bromobut-2-en-2-yl)-4-(*tert*-butyl)benzene **S35** (732 mg, 2.74 mmol) in THF gave the title compound as a yellow oil (667 mg, 95%);

ν_{max} (film): 2961, 2872, 2779, 1674, 1645, 1508, 1479, 1458, 1396, 1362, 1346, 1271, 1138, 1117, 1003; ^{1}H-NMR (400 MHz, $CDCl_3$) δ_H: **1.32** (9H, s, ArC(CH_3)$_3$), **1.77 – 1.85** (4H, m, C(3``,4``)H_2), **2.06** (3H, q, $^4J_{HH}$ = 1.0, C(4`)H_3), **2.53 – 2.62** (4H, m, C(2``,5``)H_2), **3.29** (2H, d, $^3J_{HH}$ = 6.7, C(1`)$H_2$), **5.95** (1H, tq, $^3J_{HH}$ = 6.7, $^4J_{HH}$ = 1.0, C(2`)*H*), **7.31 – 7.39** (4H, m, ArC*H*); ^{13}C{^{1}H}-NMR (126 MHz, $CDCl_3$) δ_C: **16.2** (*C*(4`)H_3), **23.6** (C(3``,4``)H_2), **31.5** (ArC(*C*H_3)$_3$), **34.6** (Ar*C*(CH_3)$_3$), **54.3** (C(2``,5``)H_2), **54.4** (*C*(1`)$H_2$), **125.2** (*C*(2`)H), **125.5** (Ar*C*(3,5)H), **127.8** (Ar*C*(2,6)H), **137.4** (*C*(3`)), **140.6** (Ar*C*(4)C(CH_3)$_3$), **149.9** (Ar*C*(1)); HRMS (ESI$^+$) $C_{18}H_{27}N$ [M+H]$^+$: found 258.2212, required 258.2216 (–1.6 ppm).

(*E*)-1-(3-(4-Nitrophenyl)but-2-en-1-yl)pyrrolidine S54

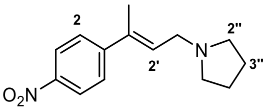

Following General Procedure **D**, pyrrolidine (3.37 mL, 41.0 mmol), (*E*)-1-(4-bromobut-2-en-2-yl)-4-nitrobenzene **S36** (2.10 g, 8.20 mmol) in THF gave the title compound as a brown oil (1.60 g, 79%);

ν_{max} (film): 2961, 2785, 1593, 1514, 1342, 1111; ^{1}H-NMR (400 MHz, $CDCl_3$) δ_H: **1.79 – 1.86** (4H, m, C(3``,4``)H_2), **2.10** (3H, d, $^4J_{HH}$ = 1.3, CH_3), **2.58 – 2.62** (4H, m, C(2``,5``)H_2), **3.33** (3H, d, $^3J_{HH}$ = 6.6, C(1`)$H_2$), **6.10** (1H, td, $^3J_{HH}$ = 6.6, $^4J_{HH}$ = 1.3, C(2`)*H*), **7.53** (2H, d, $^3J_{HH}$ = 8.8, ArC(2,6)*H*), **8.16** (2H, d, $^3J_{HH}$ = 8.8, ArC(3,5)*H*); ^{13}C{^{1}H}-NMR (126 MHz, $CDCl_3$) δ_C: **16.1** (*C*H_3), **23.7** (C(3``,4``)H_2), **54.3** (*C*(1`)H_2), **54.5** (*C*(2``,5``)H_2), **123.8** (Ar*C*(2,6)H), **126.5** (Ar*C*(3,5)H), **129.7** (*C*(2`)H), **135.0** (*C*(3`)),

146.8 (Ar*C*(4)NO_2), **149.9** (Ar*C*(1)); HRMS (ESI$^+$) $C_{14}H_{18}N_2O_2$ [M+H]$^+$: found 247.1437, required 247.1441 (–1.6 ppm).

(*E*)-1-(3-(4-Fluorophenyl)but-2-en-1-yl)pyrrolidine S55

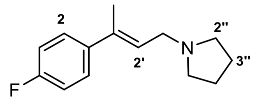

Following General Procedure **D**, pyrrolidine (1.28 mL, 15.5 mmol), (*E*)-1-(4-bromobut-2-en-2-yl)-4-fluorobenzene **S37** (712 mg, 3.10 mmol) in THF gave the title compound as a yellow oil (522 mg, 77%);

ν_{max} (film): 2963, 2779, 1684, 1636, 1483, 1458, 1399, 1136, 1074; ^{1}H-NMR (400 MHz, $CDCl_3$) δ_H: **1.78 –1.86** (4H, m, C(3``,4``)*H*$_2$), **2.04** (3H, d, $^4J_{HH}$ = 1.4, C*H*$_3$), **2.57** (4H, m, C(2``,5``)*H*$_2$), **3.27** (2H, d, $^3J_{HH}$ = 6.7, C(1`)*H*$_2$), **5.88** (1H, td, $^3J_{HH}$ = 6.7, $^4J_{HH}$ = 1.4, C(2`)*H*), **6.96 – 7.01** (2H, m, Ar*C*(3,5)*H*), **7.33 – 7.38** (2H, m, Ar*C*(2,6)*H*); ^{13}C{^{1}H}-NMR (126 MHz, $CDCl_3$) δ_C: **16.4** (*C*H$_3$), **23.6** (*C*(3``,4``)H$_2$), **54.3** (*C*(1`)H$_2$), **54.4** (*C*(2``,5``)H$_2$), **115.1** (d, $^2J_{CF}$ = 21.3, Ar*C*(3,5)H), **125.8** (*C*(2`)H), **127.3** (d, $^3J_{CF}$ = 7.9, Ar*C*(2,6)H), **135.4** (*C*(3`)), **139.7** (Ar*C*(1), **162.1** (d, $^1J_{CF}$ = 245.4, Ar*C*(4)F); HRMS (ESI$^+$) $C_{14}H_{18}FN$ [M+H]$^+$: found 220.1488, required 220.1496 (–3.6 ppm).

(*E*)-1-(3-(4-Bromophenyl)but-2-en-1-yl)pyrrolidine S56

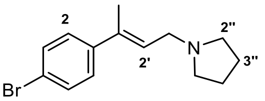

Following General Procedure **D**, pyrrolidine (690 µL, 8.40 mmol), (*E*)-1-(4-bromobut-2-en-2-yl)-4-bromobenzene **S38** (487 mg, 1.68 mmol) in THF gave the title compound as a brown oil (424 mg, 90%);

ν_{max} (film): 2963, 2780, 1684, 1641, 1480, 1459, 1399, 1136, 1077, 1002; ^{1}H-NMR (400 MHz, $CDCl_3$) δ_H: **1.78 – 1.83** (4H, m, C(3``,4``)*H*$_2$), **2.03** (3H, d, $^4J_{HH}$ = 1.3, C*H*$_3$), **2.53 – 2.59** (4H, m, C(2``,5``)*H*$_2$), **3.27** (2H, d, $^3J_{HH}$ = 6.7, C(1`)*H*$_2$), **5.93** (1H, tq, $^3J_{HH}$ = 6.7, $^4J_{HH}$ = 1.3, C(2`)*H*), **7.26** (2H, dd, $^3J_{HH}$ = 8.6, $^4J_{HH}$ = 2.1, ArC(2,6)*H*), **7.42** (2H, dd, $^3J_{HH}$ = 8.6, $^4J_{HH}$ = 2.1, ArC(3,5)*H*); ^{13}C{^{1}H}-NMR (126 MHz, $CDCl_3$) δ_C: **16.2** (*C*H$_3$), **23.6** (*C*(3``,4``)), **54.3** (*C*(1`)H$_2$), **54.4** (*C*(2``,5``)H$_2$), **120.8** (*C*(2`)H), **126.6** (Ar*C*(4)Br), **127.5** (Ar*C*(2,6)H), **131.4** (Ar*C*(3,5)H), **135.3** (*C*(3`)H), **142.5** (Ar*C*(1)); HRMS (ESI$^+$) $C_{14}H_{19}BrN$ [M+H]$^+$: found 280.0690, required 280.0695 (–1.8 ppm).

(*E*)-1-(3-(4-Iodophenyl)but-2-en-1-yl)pyrrolidine S57

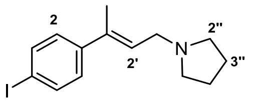

Following General Procedure **D**, pyrrolidine (1.60 mL, 19.6 mmol), (*E*)-1-bromo-3-(4-iodobut-2-en-2-yl)benzene **S39** (1.32 g, 3.92 mmol) in THF gave the title compound as a brown oil (1.08 g, 84%);

ν_{max} (film): 2961, 2872, 2779, 1682, 1645, 1479, 1458, 1396, 1371, 1346, 1138, 1069, 1003; ^{1}H-NMR (400 MHz, $CDCl_3$) δ_H: **1.77 – 1.85** (4H, m, C(3``,4``)*H*$_2$), **2.03** (3H, q, $^4J_{HH}$ = 1.4, C*H*$_3$), **2.51 – 2.62** (4H, m, C(2``,5``)*H*$_2$), **3.26** (2H, d, $^3J_{HH}$ = 6.7, C(1`)*H*$_2$), **5.93** (1H, tq, $^3J_{HH}$ = 6.7, $^4J_{HH}$ = 1.4, C(2`)*H*), **7.11 – 7.17** (2H, m, ArC(2,6)*H*), **7.58 – 7.65** (2H, m, ArC(3,5)*H*); ^{13}C{^{1}H}-NMR (126 MHz, $CDCl_3$) δ_C: **16.1** (*C*H$_3$), **23.6** (*C*(3``,4``)H$_2$), **54.3** (*C*(1`)H$_2$), **54.4** (*C*(2``,5``)H$_2$), **92.3** (Ar*C*(4)I), **126.6** (*C*(2`)H),

127.8 (Ar*C*(2,6)H), **135.4** (*C*(3`)), **137.3** (Ar*C*(3,5)H), **143.1** (ArC(1)); HRMS (ESI$^+$) $C_{14}H_{18}IN$ [M+H]$^+$: found 328.0551, required 328.0557 (–1.8 ppm).

(*E*)-1-(3-(5-Bromo-2-methoxyphenyl)but-2-en-1-yl)pyrrolidine S58

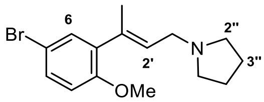

Following General Procedure **D**, pyrrolidine (1.50 mL, 18.6 mmol), (*E*)-1-bromo-2-(4-bromobut-2-en-2-yl)-4-methoxybenzene **S40** (955 mg, 3.71 mmol) in THF gave the title compound as a brown oil (826 mg, 72%);

ν_{max} (film): 2959, 2779, 1587, 1485, 1460, 1439, 1396, 1371, 1346, 1288, 1248, 1223, 1180, 1140, 1057, 1028; ^{1}H-NMR (400 MHz, $CDCl_3$) δ_H: **1.77 – 1.83** (4H, m, C(3``,4``)*H*$_2$), **1.97** (3H, d, $^4J_{HH}$ = 1.5, C(4`)*H*$_3$), **2.54 – 2.60** (4H, m, C(2``,5``)*H*$_2$), **3.24** (2H, d, $^3J_{HH}$ = 6.8, C(1`)*H*$_2$), **3.78** (3H, s, OC*H*$_3$), **5.63** (1H, tq, $^3J_{HH}$ = 6.8, $^4J_{HH}$ = 1.5, C(2`)*H*), **6.71** (1H, d, $^3J_{HH}$ = 8.7, ArC(3)*H*), **7.26** (1H, d, $^4J_{HH}$ = 2.5, ArC(6)*H*), **7.30** (1H, t, $^3J_{HH}$ = 8.7, $^4J_{HH}$ = 2.5, ArC(4)*H*); ^{13}C{^{1}H}-NMR (126 MHz, $CDCl_3$) δ_C: **17.3** (*C*(4`)H$_3$), **23.6** (*C*(3``,4``)H$_2$), **53.6** (*C*(1`)H$_2$), **54.2** (*C*(2``,5``)H$_2$), **55.8** (O*C*H$_3$), **112.5** (Ar*C*(5)Br), **112.8** (Ar*C*(3)H), **127.9** (Ar*C*(1)), **130.6** (*C*(2`)), **132.3** (Ar*C*(6)H), **135.8** (Ar*C*(4)H), **136.6** (*C*(3`)), **155.9** (Ar*C*(2)OCH$_3$); HRMS (NSI$^+$) $C_{15}H_{21}BrNO$ [M+H]$^+$: found 310.0806, required 310.0801 (+1.6 ppm).

(*E*)-1-(3-(3-Bromophenyl)but-2-en-1-yl)pyrrolidine S59

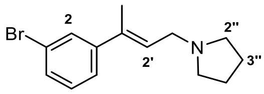

Following General Procedure **D**, pyrrolidine (3.62 mL, 44.1 mmol), (*E*)-1-bromo-3-(4-bromobut-2-en-2-yl)benzene **S41** (2.00 g, 8.81 mmol) in THF gave the title compound as a brown oil (1.70 g, 69%);

ν_{max} (film): 3383, 2957, 2924, 2779, 1661, 1591, 1557, 1476, 1458, 1412, 1373, 1346, 1246, 1140; ^{1}H-NMR (400 MHz, $CDCl_3$) δ_H: **1.78 – 1.84** (4H, m, C(3``,4``)*H*$_2$), **2.04** (3H, d, $^4J_{HH}$ = 1.4, C*H*$_3$), **2.53 – 2.60** (4H, m, C(2``,5``)*H*$_2$), **3.28** (2H, d, $^3J_{HH}$ = 6.7, C(1`)*H*$_2$), **5.94** (1H, td, $^3J_{HH}$ = 6.6, $^4J_{HH}$ = 1.4, C(2`)*H*), **7.17** (1H, t, $^3J_{HH}$ = 7.9, ArC(5)*H*), **7.31** (1H, appdt, $^3J_{HH}$ = 7.9, $^4J_{HH}$ = 1.4, ArC(6)*H*), **7.35** (1H, dt, $^3J_{HH}$ = 7.8, $^4J_{HH}$ = 1.5, C(4)*H*), **7.53** (1H, appt, $^4J_{HH}$ = 1.9, C(2)*H*); ^{13}C{^{1}H}-NMR (126 MHz, $CDCl_3$) δ_C: **16.2** (*C*H$_3$), **23.6** (*C*(3``,4``)H$_2$), **54.1** (*C*(1`)H$_2$), **54.3** (*C*(2``,5``)H$_2$), **122.6** (*C*(2`)H), **124.5** (Ar*C*(3)Br), **126.7** (Ar*C*(6)H), **129.0** (Ar*C*(4)H), **129.8** (Ar*C*(2)H), **129.9** (Ar*C*(5)H), **135.5** (*C*(3`)H), **145.7** (Ar*C*(1)); HRMS (NSI$^+$) $C_{14}H_{19}BrN$ [M+H]$^+$: found 280.0698, required 280.0695 (–1.1 ppm).

1-(3-cyclopropyl-3-phenylallyl)pyrrolidine S60

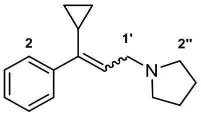

To a solution of (*E*)-3-cyclopropyl-3-phenylprop-2-en-1-ol **S28** (314 mg, 1.80 mmol) in Et_2O (5 mL) was added PBr_3 (69 µL, 739 µmol) dropwise at –20 °C. The reaction was stirred at 0 °C for 1 hour, before saturated aqueous $NaHCO_3$ was added dropwise at 0 °C until effervescence stopped. The mixture was extracted with Et_2O (3 × 5 mL), and the combined organic layers were washed with brine and saturated aqueous $Na_2S_2O_3$ (10 mL, 1:1), dried over anhydrous magnesium sulfate, filtered and concentrated *in vacuo* at room temperature. The crude residue was dissolved in anhydrous THF (5 mL)

and pyrrolidine (648 µL, 7.89 mmol) added dropwise at 0 °C. The reaction mixture was stirred at room temperature for 16 h, before addition of aqueous NaOH (10 mL, 1 M). After 30 minutes of stirring, Et_2O (20 mL) was added, and the layers separated. The aqueous layer was extracted with Et_2O (3 × 10 mL), and the combined organic layers washed with brine (10 mL), dried over anhydrous magnesium sulfate, filtered and concentrated *in vacuo*. The crude residue was redissolved in EtOAc (20 mL) and filtered to remove solid impurities. Concentration *in vacuo* gave the title compound as a yellow oil (162 mg, 40%, (*E*)/(*Z*) = 2:1);

ν_{max} (film): 3420, 2963, 2778, 1670, 1599, 1491, 1443, 1344, 1140, 1023; ^{1}H-NMR (400 MHz, $CDCl_3$) δ_H: **0.29 – 0.34** (1.2H, m, C(5`,6`)$H_A H_B$ (*major*)), **0.42 – 0.47** (0.8H, m, C(5`,6`)$H_A H_B$ (*minor*)), **0.61 – 0.67** (0.8H, m, C(5`,6`)$H_A H_B$ (*minor*)), **0.77 – 0.83** (1 H, m C(5`,6`)$H_A H_B$ (*major*)), **1.59** (0.4H, ttd, $^3J_{HH}$ = 8.3, 5.3, $^4J_{HH}$ = 0.9, C(4`)*H* (*minor*)), **1.69 – 1.77** (2.2H, m, C(3``,4``)H_2 (*minor*) and C(4`)*H* (*major*), **1.79 – 1.84** (2.4H, m, C(3``,4``)H_2 (*major*)), **2.39 – 2.44** (1.6H, m, C(2``,5``)H_2 (*minor*)), **2.56 –2.64** (2.4H, m, C(2``,5``)H_2 (*major*), **2.97** (0.8H, d, $^3J_{HH}$ = 6.9, C(1`)$H_2$ (*minor*)), **3.47** (1H, d, $^3J_{HH}$ 6.6, C(1`)H_2 (*major*)), **5.60** (0.4H, td, $^3J_{HH}$ = 6.9, $^4J_{HH}$ = 1.0, C(2`)*H* (*minor*)), **5.84** (0.6H, td, $^3J_{HH}$ = 6.6, C(2`)*H* (*major*)), **7.10 – 7.14** (0.6H, m, ArC(4)*H* (*major*)), 7.17 – 7.34 (4.4H, m, ArC*H*); 13C{1H}-NMR (500 MHz, $CDCl_3$) δ_C: **5.5** (*C*(5`,6`)H_2 (*major*)), **6.9** (*C*(5`,6`)H_2 (*minor*)), **11.8** (*C*H$(CH_2)_2$ (*minor*)), **18.6** (*C*H$(CH_2)_2$ (*major*)), **23.5** (*C*(3``,4``)H_2 (*major*)), **23.7** (*C*(3``,4``)H_2 (*minor*)), **54.2** (*C*(3``,4``)H_2 (*major*)), **54.3** (*C*(3``,4``)H_2 (*minor*)), **122.4** (*C*(2`)H (*major+minor*)), **126.6** (Ar*C*(4)H (*minor*)), **126.9** (Ar*C*(2,6)H (*minor*)), **127.5** (Ar*C*(3,5)H (*minor*)), **127.8** (Ar*C*(4)H (*major*)), **128.0** (Ar*C*(2,6)H (*major*)), **128.9** (Ar*C*(3,5)H (*major*)), **139.9** (Ar*C*(1) (*major*)), **142.1** (Ar*C*(1) (*minor*)), **142.3** (*C*(3`) (*minor*)), **144.3** (*C*(3`) (major)); HRMS (ESI$^+$) $C_{16}H_{21}N$ $[M+H]^+$: found 228.1741, required 228.1746 (–2.2 ppm).

C. 2. v. Allylic ammonium salts

(*E*)-1-(2-(4-Nitrophenoxy)-2-oxoethyl)-1-(3-phenylbut-2-en-1-yl)pyrrolidin-1-ium bromide 2a

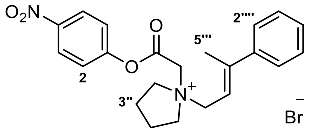

Following General Procedure **E**, (*E*)-1-(3-phenylbut-2-en-1-yl)pyrrolidine **S42** (1.20 g, 5.96 mmol), 4-nitrophenyl 2-bromoacetate (3.12 g, 12.0 mmol) in MeCN gave the title compound as a white solid (2.53 g, 92%);

mp.: 144 °C (MeCN/Et_2O); ν_{max} (film): 3035, 1775, 1614, 1587, 1518, 1483, 1402, 1346, 1296, 1206, 1165, 1155, 1136, 1088; ^{1}H-NMR (500 MHz, d^6-DMSO) δ_H: **2.13** (3H, s, CH_3), **2.15 – 2.19** (4H, m, C(3``,4``)H_2), **3.79 – 3.99** (4H, m, C(2``,5``)H_2), **4.48** (2H, d, $^3J_{HH}$ = 6.6, C(2```)H_2), **4.90** (2H, s, ArOC(O)CH_2), **6.08** (1H, t, $^3J_{HH}$ = 7.6 Hz, C(3```)*H*), **7.34 – 7.48** (3H, m, ArC(3````,4````,5````)*H*), **7.51** (2H, d, $^3J_{HH}$ = 9.0, ArC(2,6)*H*), **7.55 – 7.65** (2H, m, ArC(2````,6````)*H*), **8.34** (2H, d, $^3J_{HH}$ = 9.0, ArC(3,5)*H*); ^{13}C{^{1}H}-NMR (126 MHz, d^6-DMSO) δ_C: **16.4** (*C*H_3), **22.3** (*C*(3``,4``)H_2), **58.6** (ArOC(O)*C*H_2), **59.7** (*C*(2```)H_2), **63.3** (*C*(2``,5``)H_2), **114.6** (*C*(2`)H), **123.1** (Ar*C*(2,6)H), **125.6** (Ar*C*(3,5)H), **126.3** (Ar*C*(2````,6````)H), **128.4** (Ar*C*(4````)H), **128.5** (Ar*C*(3````,5````)H), **141.4** (*C*(4```)), **145.6** (Ar*C*(1````)), **146.1** (ArC(4)NO_2), **153.9** (Ar*C*(1)), **163.9** (ArO_2CCH_2); HRMS (NSI$^+$) $C_{22}H_{25}N_2O_4$ $[M]^+$: found 381.1802, required 381.1809 (–1.8 ppm).

(*E*)-1-(2-(4-Nitrophenoxy)-2-oxoethyl)-1-(3-phenylpent-2-en-1-yl)pyrrolidin-1-ium bromide 2b

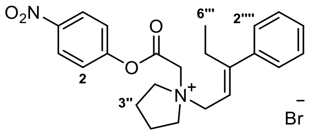

Following General Procedure **E**, (*E*)-1-(3-phenylpent-2-en-1-yl)pyrrolidine **S43** (263 mg, 1.22 mmol), 4-nitrophenyl 2-bromoacetate (635 mg, 2.44 mmol) in MeCN gave the title compound as white solid (372 mg, 64%);

mp: 159 °C (MeCN/Et_2O); ν_{max} (film): 2967, 2878, 1769, 1616, 1589, 1528, 1485, 1589, 1528, 1485, 1462, 1412, 1350, 1204, 1171, 1138, 1088; ^{1}H-NMR (500 MHz, d^6-DMSO) δ_H: **0.88** (3H, t, $^3J_{HH}$ = 7.4, C*H*$_3$), **2.13 – 2.22** (4H, m, C(3``,4``)*H*$_2$), **2.59** (2H, q, $^3J_{HH}$ = 7.6, C(5```)*H*$_2$), **3.78 – 3.90** (4H, m, C(2``,5``)*H*$_2$), **4.45** (2H, d, $^3J_{HH}$ = 7.6, C(2```)*H*$_2$), **4.88** (2H, s, ArOC(O)C*H*$_2$), **5.94** (1H, t, $^3J_{HH}$ = 7.6, C(3```)*H*), **7.33 – 7.43** (3H, m, ArC(3````,4````,5````)*H*), **7.49 – 7.55** (4H, m, ArC(2,6)*H* and ArC(2````,6````)*H*), **8.35** (2H, d, $^3J_{HH}$ = 9.0, ArC(3,5)*H*); ^{13}C{^{1}H}-NMR (126 MHz, d^6-DMSO) δ_C: **13.4** (*C*(6```)H$_3$), **22.3** (*C*(3``,4``)H$_2$), **22.4** (*C*(5```)H$_2$), **58.5** (*C*(2```)H$_2$), **59.4** (ArOC(O)*C*H$_2$), **63.4** (*C*(2``,5``)H$_2$), **114.3** (*C*(3```)H), **123.0** (Ar*C*(2,6)H), **125.6** (Ar*C*(3,5)H), **126.7** (Ar*C*(3````,5````)H), **128.3** (Ar*C*(4````)H), **128.5** (Ar*C*(2````,6````)H), **140.3** (*C*(4```)), **145.6** (Ar*C*(1````)), **152.1** (Ar*C*(4)NO$_2$), **153.9** (Ar*C*(1)), **163.8** (ArO*C*(O)CH$_2$); HRMS (NSI$^+$) $C_{23}H_{27}N_2O_4^+$ [M]$^+$: found 395.1960, required 395.1965 (–1.3 ppm).

(*E*)-1-(4-Methyl-3-phenylpent-2-en-1-yl)-1-(2-(4-nitrophenoxy)-2-oxoethyl)pyrrolidin-1-ium 2c

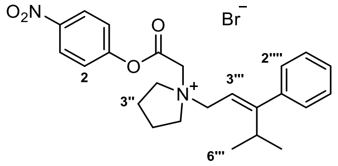

Following General Procedure **E**, (*E*)-1-(4-methyl-3-phenylpent-2-en-1-yl)pyrrolidine **S44** (550 mg, 2.40 mmol), 4-nitrophenyl 2-bromoacetate (1.25 g, 4.80 mmol) in MeCN gave the title compound as a white solid (1.16 g, 99%);

mp: 151 °C (MeCN/Et_2O); ν_{max} (film): 2968, 2922, 2891, 1775, 1533, 1489, 100, 1344, 1202, 1150, 1038; ^{1}H-NMR (400 MHz, d^6-DMSO) δ_H: **0.96** (6H, d, $^3J_{HH}$ = 6.9, (C(6```)*H*$_3$)$_2$), **2.10 – 2.22** (4H, m, C(3``,4``)*H*$_2$), **3.06** (1H, hpt, $^3J_{HH}$ = 6.9, C(5```)*H*), **3.76 – 3.91** (4H, m, C(2``,5``)*H*$_2$), **4.46** (2H, d, $^3J_{HH}$ = 7.5, C(2```)*H*$_2$), **4.87** (2H, s, ArOC(O)C*H*$_2$), **5.58** (1H, t, $^3J_{HH}$ = 7.4, C(3```)*H*), **7.22 – 7.29** (2H, m, ArC(2````,6````)*H*), **7.31 – 7.42** (3H, m, ArC(3````,4````,5````)*H*), **7.56** (2H, d, $^3J_{HH}$ = 9.2, ArC(2,6)*H*), **8.39** (2H, d, $^3J_{HH}$ = 9.2, ArC(3,5)*H*); ^{13}C{^{1}H}-NMR (126 MHz, d^6-DMSO) δ_C: **21.4** (2 × *C*6```), **22.4** (*C*(5```)H), **28.7** (*C*(3``,4``)H$_2$), **58.3** (*C*(2```)H$_2$), **58.6** (*C*(2``,5``)H$_2$), **63.4** (ArOC(O)*C*H$_2$), **115.6** (*C*(3```)H), **123.0** (Ar*C*(2,6)H), **125.6** (Ar*C*(3,5)H), **127.4** (Ar*C*(4````)H), **127.9** (Ar*C*(2````,6````)H), **128.2** (Ar*C*(3````,5````)H), **140.5** (*C*(4```)), **145.6** (Ar*C*(1````)), **153.9** (Ar*C*(4)NO$_2$), **157.1** (Ar*C*(1)), **163.9** (ArO*C*(O)CH$_2$); HRMS (ESI$^+$) $C_{24}H_{29}BrN_2O_4^+$ [M]$^+$: found 488.1317, required 488.1311 (+1.2 ppm).

1-(2-(9*H*-fluoren-9-ylidene)ethyl)-1-(2-(4-nitrophenoxy)-2-oxoethyl)pyrrolidin-1-ium bromide 2d

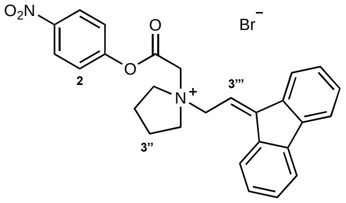

Following General Procedure **E**, 1-(2-(9*H*-fluoren-9-ylidene)ethyl)pyrrolidine **S45** (205 mg, 0.78 mmol), 4-nitrophenyl 2-bromoacetate (612 mg, 2.35 mmol) in MeCN gave the title compound as a beige solid (255 mg, 62%);

mp.: 140 – 142 °C (MeCN/Et_2O); ^{1}H-NMR (500 MHz, d^6-DMSO) δ_H: **2.22 – 2.26** (4H, m, C(3``)$H_2$), **4.06 – 4.10** (4H, m, C(2``)H_2), **5.13** (2H, d, $^3J_{HH}$ = 6.9 Hz, C(2```)H_2), 5.17 (2H, s, C(2`)H_2), **7.19** (1H, t, $^3J_{HH}$ = 6.9 Hz, C(3```)*H*), **7.29 – 7.57** (6H, Ar*H*), **7.85** (1H, d, $^3J_{HH}$ = 7.5 Hz, Ar*H*), **7.92** (1H, d, $^3J_{HH}$ = 7.6 Hz, Ar*H*), **7.99** (1H, d, $^3J_{HH}$ = 7.8 Hz, Ar*H*), **8.14** (1H, d, $^3J_{HH}$ = 7.5 Hz, Ar*H*), **8.25** (2H, d, $^3J_{HH}$ = 8.7 Hz, 2H); ^{13}C NMR (126 MHz, DMSO) δ_C = **22.5** (*C*(3``)$H_2$), **59.2** (*C*$H_2$), **59.8** (*C*$H_2$), **64.3** (*C*(2``)H_2), **116.2** (*C*(3```)H), **119.9** (Ar*C*(2,6)H), **120.5** (Ar*C*), **121.9** (Ar*C*), **122.9** (Ar*C*), **125.3** (Ar*C*), **125.8** (Ar*C*), **127.5** (Ar*C*), **127.6** (Ar*C*), **129.3** (Ar*C*), **129.7** (Ar*C*), **134.7** (Ar*C*), **137.9** (Ar*C*), **138.6** (Ar*C*), **140.5** (Ar*C*), **141.2** (*C*(4```)), **145.5** (Ar*C*(4)NO_2), **153.8** (Ar*C*(1)), **163.6** (*C*O_2Ar); HRMS (NSI$^+$) $C_{27}H_{25}N_2O_4$ [M]$^+$: found 441.1793, required 441.1809 (–3.6 ppm).

1-(3,3-diphenylallyl)-1-(3-(4-nitrophenyl)-2-oxopropyl)pyrrolidin-1-ium bromide 2e

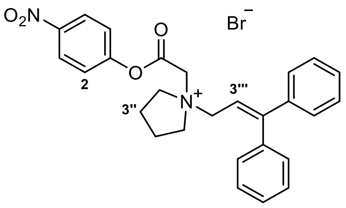

Following General Procedure **E**, 1-(3,3-diphenylallyl)pyrrolidine **S46** (548 mg, 2.08 mmol), 4-nitrophenyl 2-bromoacetate (649 mg, 2.50 mmol) in MeCN gave the title compound as a white solid (853 mg, 78%);

mp.: 155 – 157 °C (MeCN/Et_2O); ν_{max} (film): 3036, 2982, 2963, 1767, 1634, 1616, 1591, 1520, 1485, 1445, 1416, 1385, 1344 1190, 1157; ^{1}H-NMR (500 MHz, d^6-DMSO) δ_H: **1.90 – 2.19** (4H, m, C(3``,4``)H_2), **3.74 – 3.85** (4H, m, C(2``,5``)H_2), **4.31** (2H, d, $^3J_{HH}$ = 7.3, C(2```)H_2), **4.89** (2H, s, ArOC(O)CH_2), **6.45** (1H, t, $^3J_{HH}$ = 7.3, C(3```)*H*), **7.32 – 7.43** (8H, m, ArC*H*), **7.43 – 7.48** (2H, m, ArC(2,6)*H*), **8.33 – 8.39** (2H, m, ArC(3,5)*H*); ^{13}C{^{1}H} NMR (126 MHz, d^6-DMSO) δ_C: **21.9** (*C*(3``,4``)H_2), **58.2** (ArOC(O)*C*H_2), **59.1** (*C*(2```)H_2), **63.5** (*C*(2``,5``)H_2), **115.4** (*C*(3```)H), **122.9** (Ar*C*(2,6)H), **125.5** (Ar*C*(3,5)H), **126.2** (Ar*C*(4````)H), **127.8** (Ar*C*(2````,6````)H), **128.4** (ArC(3````,5````)H), **128.7** (Ar*C*(4`````)H), **128.7** Ar*C*(2`````,6`````)H), **129.3** (Ar*C*(3`````,5`````)H), **140.4** (*C*(4```)), **145.5** (Ar*C*(1````)), **150.7** (ArC(4)NO_2), **153.8** (Ar*C*(1)), **163.2** (*C*O_2Ar); HRMS (NSI$^+$) $C_{27}H_{27}N_2O_4$ [M]$^+$: found 443.1959, required 443.1965 (–1.4 ppm).

(*E*)-1-(2-(4-Nitrophenoxy)-2-oxoethyl)-1-(3-phenylbut-2-en-1-yl)piperidin-1-ium bromide 2f

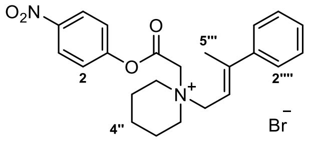

Following General Procedure **E**, (*E*)-1-(3-phenylbut-2-en-1-yl)piperidine **S47** (259 mg, 1.20 mmol), 4-nitrophenyl 2-bromoacetate (624 mg, 2.40 mmol) in MeCN gave the title compound as a white solid (269 mg, 47%);

mp: 114 − 116 °C (MeCN/Et_2O); ν_{max} (film): 3076, 1765, 1528, 1344, 1288, 1193, 1151, 1041, 1011; ^{1}H NMR (500 MHz, d^6-DMSO) δ_H: **1.55 – 1.73** (2H, m, C(4``)*H*$_2$), **1.89 – 2.03** (4H, m, C(3``,5``)*H*$_2$), **2.16** (3H, s, C(5```)*H*$_3$), **3.61** – **3.82** (4H, m, C(2``,6``)*H*$_2$), **4.49** (2H, d, $^3J_{HH}$ = 7.8, C(2```)*H*$_2$, **4.92** (2H, s, ArOC(O)C*H*$_2$), **6.06** (1H, t, $^3J_{HH}$ = 7.7 Hz, C(3```)*H*), **7.32 – 7.44** (3H, m, ArC(3````,4````,5````)*H*), **7.46 – 7.50** (2H, m, ArC(2,6)*H*), **7.61** (2H, d, $^3J_{HH}$ = 6.9 Hz, ArC(2````,6````)*H*), **8.35** (2H, d, $^3J_{HH}$ = 9.1 Hz, ArC(3,5)*H*); ^{13}C{^{1}H} NMR (126 MHz, d^6-DMSO) δ_C: **16.5** (*C*(5```)H$_3$), **19.3** (*C*(4``)H$_2$), **20.5** (*C*(3``,5``)H), **59.5** (*C*(2```,6```)H$_2$), **59.7** (*C*(2```)H$_2$), **64.9** (ArOC(O)*C*H$_2$), **113.2** (*C*(3```)H), **123.0** (Ar*C*(2,6)H), **125.5** (Ar*C*(3,5)H), **125.8** (Ar*C*(4````)H), **126.2** (Ar*C*(2````,6````)H), **128.4** (Ar*C*(3````,5````)H), **141.4** (C(4```)), **145.6** (Ar*C*(1````)), **146.6** (Ar*C*(4)NO$_2$), **153.8** (Ar*C*(1)), **163.4** (ArO*C*(O)CH$_2$); HRMS (NSI$^+$) $C_{23}H_{27}N_2O_4^+$ [M]$^+$: found 395.1958, required 395.1965 (–1.8 ppm).

(*E*)-1-(2-(4-Nitrophenoxy)-2-oxoethyl)-1-(3-phenylbut-2-en-1-yl)azepan-1-ium bromide 2g

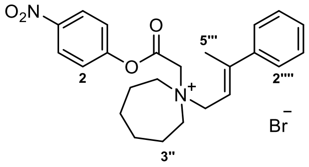

Following General Procedure **E**, (*E*)-1-(3-phenylbut-2-en-1-yl)azepane **S58** (617 mg, 2.69 mmol), 4-nitrophenyl 2-bromoacetate (839 mg, 3.23 mmol) in MeCN gave the title compound as a beige solid (1.02 g, 78%) which was contaminated with remaining (*E*)-1-(3-phenylbut-2-en-1-yl)azepane **S58** (~1:1).

mp: 88 − 90 °C (MeCN/Et_2O); ν_{max} (film): 2934, 2689, 2604, 1769, 1589, 1524, 1344, 1200, 1161; ^{1}H NMR (500 MHz, d^6-DMSO) δ_H: **1.76 – 1.93** (4H, m, C(4``,5``)*H*$_2$), **1.95 – 2.03** (4H, m, C(3`,6``)*H*$_2$), **2.15** (3H, s, C*H*$_3$), **3.75 – 3.86** (4H, m, C(2``,7``)*H*$_2$), **4.48** (2H, d, $^3J_{HH}$ = 7.7, C(1```)*H*$_2$), **4.84** (2H, s, ArOC(O)C*H*$_2$), **6.08** (1H, t, $^3J_{HH}$ = 7.7, C(2```)*H*), **7.38 – 7.45** (2H, m, ArC*H*), **7.48 – 7.54** (2H, m, ArC*H*), **7.58 – 7.65** (1H, m, ArC*H*), **8.34 – 8.43** (2H, m, ArC(3,5)*H*; ^{13}C{^{1}H} NMR (126 MHz, d^6-DMSO) δ_C: **16.5** (*C*H$_3$), **21.4** (*C*(4``,5``)H$_2$), **27.2** (*C*(3``,6``)H$_2$), **58.3** (*C*(2```)H$_2$), **60.9** (ArOC(O)*C*H$_2$), **63.1** (*C*(2``,7``)H$_2$), **113.9** (*C*(3```)H), **123.1** (Ar*C*(2,6)H), **125.7** (Ar*C*(3,5)H), **126.3** (Ar*C*(2````,6````)H), **128.5** (Ar*C*(4````)H), **128.5** (Ar*C*(3````,5````)H), **141.5** (*C*(4```)), **145.7** (Ar*C*(1````)), **146.7** (Ar*C*(4)NO$_2$), **153.9** (Ar*C*(1)), **163.6** (ArOC(*O*)*CH$_2$*); HRMS (NSI$^+$) $C_{24}H_{29}N_2O_4^+$ [M]$^+$: found 409.2113, required 409.2122 (–2.2 ppm).

***N,N*-Dimethyl-*N*-(3-(4-nitrophenyl)-2-oxopropyl)-3,3-diphenylprop-2-en-1-aminium bromide 2h**

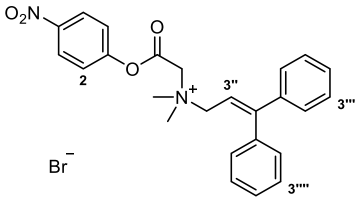

Following General Procedure **E**, *N,N*-dimethyl-3,3-diphenylprop-2-en-1-amine **S49** (529 mg, 2.23 mmol), 4-nitrophenyl 2-bromoacetate (869 mg, 3.34 mmol) in MeCN gave the title compound as a white solid (1.05 g, 94%);

mp.: 160 – 162 °C (MeCN/Et_2O); ^{1}H NMR (500 MHz, d^6-DMSO) δ_H: **3.30** (6H, s, N(C*H*$_3$)$_2$), **4.30** (2H, d, $^3J_{HH}$ = 7.3, C(2``)*H*$_2$), **4.77 – 4.87** (2H, m, ArOC(O)C*H*$_2$), **6.47** (1H, t, $^3J_{HH}$ = 7.3, C(3``)*H*), **7.15 – 7.19** (2H, m, ArC(2,6)*H*), **7.37 – 7.39** (5H, m, ArC*H*), **7.41 – 7.52** (5H, m, ArC*H*), **8.33 – 8.41** (2H, m, ArC(3,5)*H*); ^{13}C{^{1}H}-NMR (126 MHz, d^6-DMSO) δ_C: **51.1** (N(*C*H$_3$)$_2$), **60.7** (*C*(2``)H$_2$), **62.9** (ArOC(O)*C*H$_2$), **114.5** (*C*(3``)H), **123.0** (Ar*C*(2,6)H), **125.5** (Ar*C*(3,5)H), **127.8** (Ar*C*(2```,6```)H), **128.3** (Ar*C*(4```)H), **128.4** (Ar*C*(2````,6````)H), **128.7** (Ar*C*(4````)H), **128.8** (Ar*C*(3```,5```)H), **129.2** (Ar*C*(3````,5````)H), **137.3** (Ar*C*(1```)), **140.4** (Ar*C*(1````)), **145.6** (C(4``)), **151.0** (Ar*C*(4)NO$_2$), **153.8** (Ar*C*(1)), **163.0** (ArO*C*(O)CH$_2$); HRMS (ESI$^+$) $C_{25}H_{25}N_2O_4^+$ [M]$^+$: found 417.1798, required 417.1809 (−2.6 ppm).

(*E*)-*N,N*-Dimethyl-*N*-(2-(4-nitrophenoxy)-2-oxoethyl)-3-phenylbut-2-en-1-aminium bromide 2i

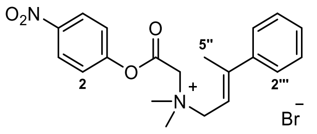

Following General Procedure **E**, (*E*)-*N,N*-dimethyl-3-phenylbut-2-en-1-amine **S50** (155 mg, 0.88 mmol), 4-nitrophenyl 2-bromoacetate (460 mg, 1.77 mmol) in MeCN gave the title compound as a white solid (258 mg, 67%);

mp: 140 °C (MeCN/Et_2O); ν_{max} (film): 2886, 1761, 1526, 1346, 1194, 1172, 1150, 1132; ^{1}H-NMR (500 MHz, d^6-DMSO) δ_H: **2.20** (3H, s, C(4``)*H*$_3$), **3.33** (6H, s, N(C*H*$_3$)$_2$), **4.43** (2H, d, $^3J_{HH}$ = 8.0, C(2``)*H*$_2$), **4.84** (2H, s, ArO$_2$CC*H*$_2$), **6.06** (1H, t, $^3J_{HH}$ = 8.0, C(3``)*H*), **7.34 – 7.42** (3H, m, ArC(3```,4```,5```)*H*), **7.57** (2H, d, $^3J_{HH}$ = 9.0, ArC(2,6)*H*), **7.62** (2H, d, $^3J_{HH}$ = 7.6, ArC(2```,6```)*H*), **8.37** (2H, d, $^3J_{HH}$ = 9.0, ArC(3,5)*H*); ^{13}C{^{1}H}-NMR (126 MHz, d^6-DMSO) δ_C: **16.5** (*C*H$_3$), **50.6** (N(*C*H$_3$)$_2$), **60.5** (*C*(2``)H$_2$), **63.1** (ArOC(O)*C*H$_2$), **113.5** (*C*(3``)H), **123.1** (Ar*C*(2,6)H), **125.6** (Ar*C*(3,5)H), **126.3** (Ar*C*(3```,5```)H), **128.4** (Ar*C*(2```,6```)H), **128.5** (Ar*C*(4```)H), **141.4** (*C*(4``)), **145.6** (Ar*C*(1```)), **147.5** (Ar*C*(4)NO$_2$), **153.9** (Ar*C*(1)), **163.3** (ArOC(*O*)CH$_2$); HRMS (NSI$^+$) $C_{20}H_{23}N_2O_4$ [M]$^+$: found 355.1650, required 355.1652 (–0.6 ppm).

(*E*)-4-(2-(4-Nitrophenoxy)-2-oxoethyl)-4-(3-phenylbut-2-en-1-yl)morpholin-4-ium bromide 2j

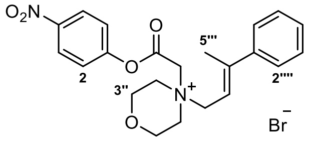

Following General Procedure **E**, (*E*)-1-(3-phenylbut-2-en-1-yl)morpholine **S51** (241 mg, 1.11 mmol), 4-nitrophenyl 2-bromoacetate (577 mg, 2.22 mmol) in MeCN gave the title compound as a white solid (268 mg, 51%);

mp: 132 – 136 °C (MeCN/Et_2O); ν_{max} (film): 2984, 2911, 2592, 1694, 1454, 1435, 1414, 1362, 1285, 1254, 1173, 1155, 1069; ^{1}H-NMR (500 MHz, d^6-DMSO) δ_H: **2.16** (3H, d, $^4J_{HH}$ = 1.3, C*H*$_3$), **3.77 – 3.93** (4H, m, C(2``,6``)*H*$_2$), **4.05 – 4.15** (4H, m, C(3``,5``)*H*$_2$), **4.65** (2H, d, $^3J_{HH}$ = 7.8, C(2```)*H*$_2$), **5.07** (2H, s, ArOC(O)C*H*$_2$), **6.04 – 6.15** (1H, m, C(3```)*H*), **7.38 – 7.45** (3H, m, ArC(2````,4````,6````)*H*), **7.47 – 7.52** (2H, m, ArC(2,6)*H*), **7.60 – 7.65** (2H, m, ArC(3````, 5````)*H*), **8.34 – 8.41** (2H, m, ArC(3,5)*H*); ^{13}C{^{1}H}-NMR (126 MHz, d^6-DMSO) δ_C: **16.8** (*C*H$_3$), **51.1** (*C*(2```)H$_2$), **58.8** (*C*(2``,6``)H$_2$), **60.3** (*C*(3``,5``)H$_2$), **64.0** (ArO$_2$C*C*(1`)H$_2$), **113.3** (*C*(3```)H), **123.4** (Ar*C*(2,6)H), **126.1** (Ar*C*(3,5)H), **126.3** (Ar*C*(4````)H), **126.8** (Ar*C*(2````,6````)H), **128.8** (Ar*C*(3````,C5````)), **141.8** (*C*(4```)), **146.1** (*C*(1````)), **147.5** (Ar*C*(4)NO$_2$), **154.2** (Ar*C*(1)), **163.7** (ArO*C*(O)CH$_2$); HRMS (NSI$^+$) $C_{22}H_{25}N_2O_5^+$ [M]$^+$: found 397.1752, required 397.1758 (–1.5 ppm).

(*E*)-1-(2-(4-nitrophenoxy)-2-oxoethyl)-1-(3-(p-tolyl)but-2-en-1-yl)pyrrolidin-1-ium bromide 2k

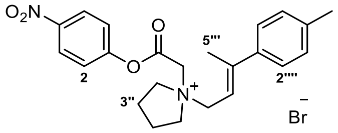

Following General Procedure **E**, (*E*)-1-(3-(p-tolyl)but-2-en-1-yl)pyrrolidine **S52** (206 mg, 0.957 mmol), 4-nitrophenyl 2-bromoacetate (547 mg, 2.10 mmol) in MeCN gave the title compound as a white solid (318 mg, 70%);

mp: 136 °C (MeCN/Et_2O); ν_{max} (film): 3044, 2967, 1767, 1614, 1589, 1528, 1387, 1344, 1310, 1194, 1157, 1123, 1042, 1009; ^{1}H-NMR (500 MHz, d^6-DMSO) δ_H: **2.09** (3H, s, C(5```)*H*$_3$), **2.12 – 2.17** (4H, m, C(3``,4``)*H*$_2$), **2.31** (3H, s, ArC*H*$_3$), **3.82** (4H, m, C(2``,5``)*H*$_2$), **4.41** (2H, d, $^3J_{HH}$ = 7.6, C(2```)*H*$_2$), **4.83** (2H, s, ArOC(O)C*H*$_2$), **6.06** (1H, t, $^3J_{HH}$ = 7.6, C(3```)*H*), **7.19 – 7.23** (2H, d, $^3J_{HH}$ = 7.9, C(3````, 5````)*H*), **7.46 – 7.53** (4H, m, C(2,6)*H* and C(2````,6````)*H*), **8.36** (2H, d, $^3J_{HH}$ = 9.1, C(3,5)*H*); ^{13}C{^{1}H}-NMR (126 MHz, d^6-DMSO) δ_C: **16.2** (*C*(5```)H$_3$), **20.7** (Ar*C*H$_3$), **22.2** (*C*(3``,4``)), **58.5** (*C*(2```)H$_2$), **59.7** (ArOC(O)*C*H$_2$), **63.3** (*C*(2``,5``)H$_2$), **113.6** (*C*(3```)H), **123.0** (Ar*C*(2,6)H), **125.6** (Ar*C*(3,5)H), **126.7** (Ar*C*(3````,5````)H), **128.3** (ArC(4````)CH$_3$), **129.0** (Ar*C*(2````,6````)H), **138.4** (*C*(4``)), **145.6** (Ar*C*(1````)), **145.9** (Ar*C*(4)NO$_2$), **153.9** (Ar*C*(1)), **163.8** (ArO*C*(O)CH$_2$); HRMS (NSI$^+$) $C_{23}H_{27}N_2O_4^+$ [M]$^+$: found 395.1957, required 395.1965 (–2.0 ppm).

(*E*)-1-(3-(4-(Tert-butyl)phenyl)but-2-en-1-yl)-1-(2-(4-nitrophenoxy)-2-oxoethyl)pyrrolidin-1-ium bromide 2l

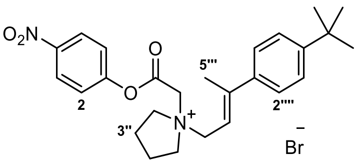

Following General Procedure **E**, (*E*)-1-(3-(4-(*tert*-butyl)phenyl)but-2-en-1-yl)pyrrolidine **S53** (661 mg, 2.59 mmol), 4-nitrophenyl 2-bromoacetate (1.01 g, 3.89 mmol) in MeCN gave the title compound as a white solid (940 mg, 70%);

mp: 127 °C (MeCN/Et_2O); ν_{max} (film): 2949, 2866, 1771, 1634, 1616, 1589, 1530, 1350, 1209, 1167; ^{1}H-NMR (400 MHz, d^6-DMSO) δ_H: **1.29** (9H, s, ArC(C*H*$_3$)$_3$), **2.12** (3H, d, $^4J_{HH}$ = 1.3, C(5```)*H*$_3$), **2.14 – 2.21** (4H, m, C(3``,4``)*H*$_2$), **3.77 – 3.91** (4H, m, C(2``,5``)*H*$_2$), **4.44** (2H, d, $^3J_{HH}$ = 7.8, C(2```)*H*$_2$), **4.85** (2H, s, ArOC(O)C*H*$_2$), **6.07** (1H, t, $^3J_{HH}$ = 7.8, C(3```)*H*), **7.38 – 7.47** (2H, m, C(2,6)*H*), **7.48 – 7.58** (4H, m, ArC(2````,3````,5````,6````)*H*), **8.33 – 8.43** (2H, m, ArC(3,5)*H*); ^{13}C{^{1}H}-NMR (101 MHz, d^6-DMSO) δ_C: **16.2** (*C*(5```)H$_3$), **22.2** (*C*(3``,4``)H$_2$), **31.0** (ArC(*C*H$_3$)$_3$), **34.3** (Ar*C*(CH$_3$)$_3$), **58.5**

(*C*(2```)H_2), **59.8** (ArOC(O)*C*H_2), **63.3** (*C*(2``,5``)H_2), **113.8** (*C*(3```)H), **123.0** (ArC(2,6)H), **125.1** (Ar*C*(3,5)H), **125.6** (Ar*C*(3````,5````)H), **125.9** (Ar*C*(2````,6````)H), **138.5** (*C*(4```)), **145.6** (ArC(1````)), **146.0** (Ar*C*(4)NO_2), **151.0** (Ar*C*(1)), **163.8** (ArO*C*(O)CH_2); HRMS (NSI^+) $C_{23}H_{33}N_2O_4^+$ $[M]^+$: found 437.2423, required 437.2435 (–2.7 ppm).

(*E*)-1-(2-(4-Nitrophenoxy)-2-oxoethyl)-1-(3-(4-nitrophenyl)but-2-en-1-yl)pyrrolidin-1-ium bromide 2m

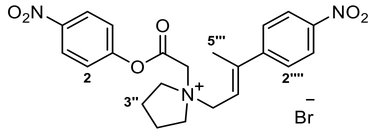

Following General Procedure **E**, (*E*)-1-(3-(4-nitrophenyl)but-2-en-1-yl)pyrrolidine **S54** (300 mg, 1.20 mmol), 4-nitrophenyl 2-bromoacetate (635 mg, 2.40 mmol) in MeCN gave the title compound as a yellow solid (328 mg, 54%);

mp: 157 °C (MeCN/Et_2O); ν_{max} (film): 3056, 1778, 1589, 1520, 1337, 1207, 1172; ^{1}H-NMR (500 MHz, d^6-DMSO) δ_H: **2.15 – 2.22** (7H, m, C(5```)*H*$_3$ and C(3``,4``)*H*$_2$), **3.81 – 3.88** (4H, m, C(2``,5``)*H*$_2$), **4.49** (2H, d, $^3J_{HH}$ = 6.6, C(2```)*H*$_2$), **4.90** (2H, s, ArO_2CC*H*$_2$), **6.30** (1H, t, $^3J_{HH}$ = 7.6, C(3```)*H*), **7.51** (2H, d, $^3J_{HH}$ = 9.2, ArC(2,6)*H*), **7.88** (2H, d, $^3J_{HH}$ = 9.0, ArC(2````,6````)*H*), **8.26** (2H, d, $^3J_{HH}$ = 9.0 Hz, ArC(3````,5````)*H*), **8.36** (2H, d, $^3J_{HH}$ = 9.2, ArC(3,5)*H*); ^{13}C{^{1}H}-NMR (126 MHz, d^6-DMSO) δ_C: **16.2** (*C*(5```)H_3), **22.2** (*C*(3``4``)H_2), **58.6** (*C*(2```)*H*$_2$), **59.4** (ArO_2C*C*H_2)¸ **63.6** (*C*(2``,5``)H_2), **118.4** (*C*(3```)H), **123.1** (Ar*C*(2,6)H), **123.6** (Ar*C*(2````,6````)H), **125.6** (Ar*C*(3````,5````)H), **127.6** (Ar*C*(3,5)H), **143.9** (*C*(4```)), **145.6** (Ar*C*(1````)), **147.1** (Ar*C*(4````)H), **147.9** (Ar*C*(4)NO_2), **153.9** (Ar*C*(1)), **163.9** (ArO*C*(O)CH_2); HRMS (NSI^+) $C_{22}H_{24}N_3O_6$ $[M]^+$: found 426.1654, required 426.1660 (–1.4 ppm).

(*E*)-1-(3-(4-Fluorophenyl)but-2-en-1-yl)-1-(2-(4-nitrophenoxy)-2-oxoethyl)pyrrolidin-1-ium bromide 2n

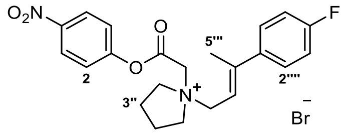

Following General Procedure **E**, (*E*)-1-(3-(4-fluorophenyl)but-2-en-1-yl)pyrrolidine **S55** (202 mg, 0.920 mmol), 4-nitrophenyl 2-bromoacetate (479 mg, 1.84 mmol) in MeCN gave the title compound as a white solid (284 mg, 64%);

mp: 140 °C (MeCN/Et_2O); ν_{max} (film): 3035, 1767, 1510, 1344, 1229, 1196, 1157, 1008; ^{1}H-NMR (500 MHz, d^6-DMSO) δ_H: **2.12** (3H, s, C*H*$_3$), **2.15 – 2.20** (4H, m, C(3``,4``)*H*$_2$), **3.79 – 3.89** (4H, m, C(2``,5``)*H*$_2$), **4.44** (2H, d, $^3J_{HH}$ = 7.6, C(2```)*H*$_2$), **4.89** (2H, s, ArO_2CC*H*$_2$), **6.09** (1H, t, $^3J_{HH}$ = 7.6, C(3```)*H*), **7.21 – 7.28** (2H, m, ArC(2````,6````)*H*), **7.53** (2H, d, $^3J_{HH}$ = 9.1, ArC(2,6)*H*), **7.64 – 7.69** (2H, m, ArC(3````,5````)*H*), **8.37** (2H, d, $^3J_{HH}$ = 9.1, ArC(3,5)*H*); ^{13}C{^{1}H}-NMR (126 MHz, d^6-DMSO) δ_C: **16.4** (*C*H_3), **22.2** (*C*(3``,4``)H_2), **58.5** (*C*(2```)H_2), **59.6** (ArO_2C*C*H_2), **63.3** (*C*(2``,5``)H_2), **114.7** (*C*(3```)H), **115.2** (Ar*C*(3````,5````)H), d, $^2J_{CF}$ = 21.3), **123.1** (Ar*C*(2,6)H), **125.6** (Ar*C*(3,5)H), **128.4** (Ar*C*(2````,6````), d, $^3J_{CF}$ = 8.3), **137.8** (*C*(4```)), **144.9** (Ar*C*(1````)), **145.6** (Ar*C*(4)NO_2), **153.9** (Ar*C*(1)), **162.2** (Ar*C*(4````). d, $^1J_{CF}$ = 245.5), **163.9** (ArO*C*(O)CH_2); ^{19}F{^{1}H}-NMR (470 MHz, d^6-DMSO) δ_F:

–113.9 – −114.0 (m, ArC(4````)*F*); HRMS (NSI$^+$) $C_{22}H_{24}FN_2O_4^+$ [M]$^+$: found 399.1716, required 399.1715 (+0.3 ppm).

(*E*)-1-(3-(4-Bromophenyl)but-2-en-1-yl)-1-(2-(4-nitrophenoxy)-2-oxoethyl)pyrrolidin-1-ium bromide 2o

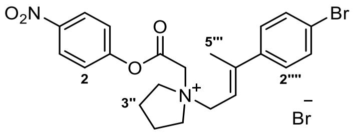

Following General Procedure **E**, (*E*)-1-(3-(4-bromophenyl)but-2-en-1-yl)pyrrolidine **S56** (160 mg, 0.570 mmol), 4-nitrophenyl 2-bromoacetate (297 mg, 1.14 mmol) in MeCN gave the title compound as an off-white solid (235 mg, 76%);

mp: 148 °C (MeCN/Et$_2$O); ν_{max} (film): 3031, 1767, 1528, 1344, 1194, 1155, 1009; ^{1}H-NMR (500 MHz, d^6-DMSO) δ_H: **2.10** (3H, s, C*H*$_3$), **2.13 – 2.19** (4H, m, C(3``,4``)*H*$_2$), **3.77 – 3.88** (4H, m, C(2``,5``)*H*$_2$), **4.43** (2H, d, $^3J_{HH}$ = 7.6, C(2```)*H*$_2$), **4.87** (2H, s, ArO$_2$CC*H*$_2$), **6.13** (1H, t, $^3J_{HH}$ = 7.6, C(3```)*H*), **7.51** (2H, d, $^3J_{HH}$ = 9.2, ArC(2,6)*H*), **7.55 – 7.63** (4H, m, ArC(2````,3````,5````,6````)*H*), **8.37** (2H, d, $^3J_{HH}$ = 9.2, ArC(3,5)*H*); ^{13}C{^{1}H}-NMR (126 MHz, d^6-DMSO) δ_C: **16.2** (*C*H$_3$), **22.2** (*C*(3``,4``)H$_2$), **58.5** (*C*(2```)H$_2$), **59.5** (ArO$_2$CCH$_2$), **63.4** (*C*(2``,5``)H$_2$), **115.4** (*C*(3```)H), **121.7** (Ar*C*(4````)Br), **123.1** (Ar*C*(2,6)H), **125.6** (Ar*C*(3,5)H), **128.4** (Ar*C*(2````,6````)H), **131.3** (Ar*C*(3````,5````)H), **140.5** (*C*4```), **144.8** (Ar*C*(1````)), **145.6** (Ar*C*(4)NO$_2$), **153.9** (Ar*C*(1)), **163.9** (ArO*C*(O)CH$_2$); HRMS (NSI$^+$) $C_{22}H_{24}BrN_2O_4^+$ [M]$^+$: found 461.0888, required 461.0897 (–2.0 ppm).

(*E*)-1-(3-(4-Iodophenyl)but-2-en-1-yl)-1-(2-(4-nitrophenoxy)-2-oxoethyl)pyrrolidin-1-ium bromide 2p

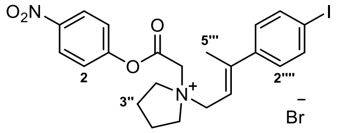

Following General Procedure **E**, (*E*)-1-(3-(4-iodophenyl)but-2-en-1-yl)pyrrolidine **S57** (1.06 g, 3.25 mmol), 4-nitrophenyl 2-bromoacetate (1.27 g, 4.88 mmol) in MeCN gave the title compound as an off-white solid (1.48 g, 77%);

mp: 143 °C (MeCN/Et$_2$O); ν_{max} (film): 3017, 2949, 2845, 1773, 1508, 1340, 1202, 1159, 1109; ^{1}H-NMR (500 MHz, d^6-DMSO) δ_H: **2.10** (3H, s, C*H*$_3$), **2.13 – 2.21** (4H, m, C(3``,4``)*H*$_2$), **3.76 – 3.92** (4H, m, C(2``,5``)*H*$_2$), **4.44** (2H, d, $^3J_{HH}$ = 7.5, C(2```)*H*$_2$), **4.88** (2H, s, ArO$_2$CC*H*$_2$), **6.13** (1H, t, $^3J_{HH}$ = 7.5, C(3```)*H*), **7.42** (2H, d, $^3J_{HH}$ = 8.5, C(2````,6````)*H*), **7.52** (2H, d, $^3J_{HH}$ = 9.2, C(2,6)*H*), **7.77** (2H, d, $^3J_{HH}$ = 8.5 Hz, C(3````,5````)*H*), **8.37** (2H, d, $^3J_{HH}$ = 9.2, C(3,5)*H*); ^{13}C{^{1}H}-NMR (101 MHz, d^6-DMSO) δ_C: **16.1** (*C*H$_3$), **22.2** (*C*(3``,4``)H$_2$), **58.5** (*C*(2```)H$_2$), **59.5** (ArOC(O)*C*H$_2$), **63.4** (*C*(2``,5``)H$_2$), **94.8** (Ar*C*(4````)I), **115.2** (*C*(3```)H), **123.0** (Ar*C*(2,6)H), **125.6** (Ar*C*(3,5)), **128.4** (Ar*C*(2````,6````)H), **137.1**

(Ar*C*(3````,5````)H), **140.8** (*C*(4```)), **145.0** (Ar*C*(1````)H), **145.6** (Ar*C*(4)NO_2), **153.9** (Ar*C*(1)), **163.8** (ArO*C*(O)CH_2); HRMS (NSI^+) $C_{22}H_{24}IN_2O_4^+$ $[M]^+$: found 507.0772, required 507.0775 (–0.6 ppm).

(*E*)-1-(3-(5-Bromo-2-methoxyphenyl)but-2-en-1-yl)-1-(2-(4-nitrophenoxy)-2-oxoethyl)pyrrolidin-1-iumbromide 2q

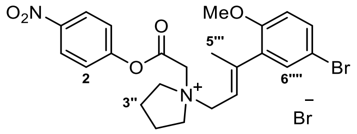

Following General Procedure **E**, (*E*)-1-(3-(5-bromo-2-methoxyphenyl)but-2-en-1-yl)pyrrolidine **S58** (826 mg, 2.66 mmol), 4-nitrophenyl 2-bromoacetate (1.39 g, 5.33 mmol) in MeCN gave the title compound as a white solid (1.24 g, 81%);

mp: 162 °C (MeCN/Et_2O); ν_{max} (film): 3048, 1771, 1616, 1591, 1528, 1483, 1396, 1348, 1196, 1177, 1032, 1016; ^{1}H-NMR (400 MHz, d^6-DMSO) δ_H: **2.00** (3H, d, $^4J_{HH}$ = 1.4, C(5```)H_3), **2.13 – 2.22** (4H, m, C(3``,4``)H_2), **3.77** (3H, s, OCH_3), **3.78 – 3.90** (4H, m, C(2``,5``)H_2), **4.38** (2H, d, $^3J_{HH}$ = 7.6, C(2```)H_2), **4.84** (2H, s, ArOC(O)CH_2), **5.77** (1H, tq, $^3J_{HH}$ = 7.6, $^4J_{HH}$ = 1.4, C(3```)*H*), **7.03** (1H, d, $^3J_{HH}$ = 8.9, ArC(3````)*H*), **7.42** (1H, d, $^4J_{HH}$ = 2.6, ArC(6````)*H*), **7.50** (1H, dd, $^3J_{HH}$ = 8.9, $^4J_{HH}$ = 2.6, ArC(4````)*H*), **7.52 – 7.56** (2H, m, ArC(2,6)*H*), **8.37 – 8.42** (2H, m, ArC(3,5)*H*); $^{13}C\{^1H\}$-NMR (101 MHz, d^6-DMSO) δ_C: **17.6** (*C*(5```)H_3), **22.1** (*C*(3``,4``)H_2)**,** **55.9** (O*C*H_3), **58.4** (*C*(2```)H_2), **58.9** (ArOC(O)*C*H_2), **63.5** (*C*(2``,5``)H_2), **111.9** (Ar*C*(3````)H), **113.7** (*C*(3```)H), **115.8** (Ar*C*(5````)Br), **123.0** (Ar*C*(2,6)H), **125.6** (Ar*C*(3,5)H), **131.2** (Ar*C*(1````)), **131.6** (Ar*C*(6````)H), **134.4** (Ar*C*(4````)H), **144.9** (*C*(4```)), **145.6** (Ar*C*(4)NO_2), **153.9** (Ar*C*(1)), **155.4** (Ar*C*(2````)OCH_3), **163.9** (ArO*C*(O)CH_2); HRMS (ESI^+) $C_{23}H_{26}N_2O_5^+$ $[M]^+$: found 489.1009, required 489.1020 (−2.2 ppm).

(*E*)-1-(3-(3-Bromophenyl)but-2-en-1-yl)-1-(2-(4-nitrophenoxy)-2-oxoethyl)pyrrolidin-1-ium bromide 2r

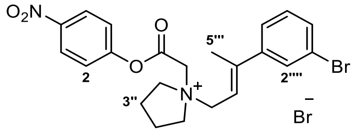

Following General Procedure **E**, (*E*)-1-(3-(3-bromophenyl)but-2-en-1-yl)pyrrolidine **S59** (1.65 g, 5.89 mmol), 4-nitrophenyl 2-bromoacetate (3.06 g, 11.8 mmol) in MeCN gave the title compound as a white solid (2.60 g, 82%);

mp: 151 °C (MeCN/Et_2O); ν_{max} (film): 3038, 2855, 1767, 1589, 1558, 1522, 1341, 1192, 1167, 1157, 1109; ^{1}H-NMR (400 MHz, d^6-DMSO) δ_H: **2.12** (3H, d, $^4J_{HH}$ = 1.3, CH_3), **2.14 – 2.22** (4H, m, C(3``,4``)H_2), **3.78 – 3.91** (4H, m, C(2``,5``)H_2), **4.44** (2H, d, $^3J_{HH}$ = 7.6, C(2```)H_2), **4.88** (2H, s, ArOC(O)CH_2), **6.15** (1H, tq, $^3J_{HH}$ = 7.6, $^4J_{HH}$ = 1.3, C(3```)*H*, **7.38** (1H, t, $^3J_{HH}$ = 7.9, ArC(5````)*H*), **7.51 – 7.55** (2H, m, ArC(2,6)*H*), **7.55 – 7.62** (2H, m, ArC(4````,6````)*H*), **7.82** (1H, t, $^4J_{HH}$ = 1.8, ArC(2````)*H*), **8.35 – 8.41** (2H, m, ArC(3,5)*H*); $^{13}C\{^1H\}$-NMR (126 MHz, d^6-DMSO) δ_C: **16.3** (*C*H_3), **22.2** (*C*(3``,4``)H_2), **58.5** (*C*(2```)H_2), **59.4** (ArOC(O)*C*H_2), **63.4** (*C*(2``,5``)H_2), **115.8** (Ar*C*(3````)Br), **116.1** (*C*(3```)H), **122.0** (Ar*C*(6````)H), **123.1** (Ar*C*(2,6)H), **125.6** (Ar*C*(3,5)H), **128.9** (Ar*C*(5````)H), **130.5** (Ar*C*(2````)H), **131.1** (Ar*C*(4````)H), **143.8** (*C*(4```)), **144.6** (Ar*C*(1````)), **145.6** (Ar*C*(4)H), **153.9** (Ar*C*(1)), **163.9** (ArO*C*(O)CH_2); HRMS (ESI^+) $C_{22}H_{24}BrN_2O_4^+$ $[M]^+$: found 459.0905, required 459.0914 (−2.0 ppm).

1-(3-cyclopropyl-3-phenylallyl)-1-(2-(4-nitrophenoxy)-2-oxoethyl)pyrrolidin-1-ium bromide 2w

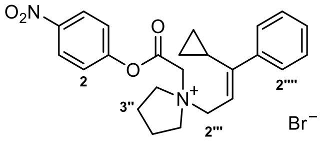

Following General Procedure **E**, 1-(3-cyclopropyl-3-phenylallyl)pyrrolidine **S60** (243 mg, 1.07 mmol) and 4-nitrophenyl 2-bromoacetate (556 mg, 2.14 mmol) in MeCN gave the title compound as a brown solid (450 mg, 86%, 2.3:1 ratio of (*E*)/(*Z*));

ν_{max} (film): 2808, 1742, 1641, 1454, 1439, 1414, 1335, 1177, 1024; ^{1}H-NMR (400 MHz, d^6-DMSO) δ_H: **0.27 – 0.32** (0.6H, m, C(5`,6`)H_AH$_B$ (*minor*)), **0.53 – 0.60** (1.4H, m, C(5`,6`)H_AH$_B$ (*major*)), **0.77 – 0.82** (1.4H, m, C(5`,6`)H$_A$$H_B$ (*major*)), **0.82 – 0.88** (0.6H, m, C(5`,6`)H$_A$$H_B$ (*minor*)), **1.76 – 1.80** (0.7H, m, C(4```)*H* (*major*)), 1.85 – 2.22 (4.3H, m, C(3``,4``)H_2 (*major* + *minor*) and C(4```)H (*minor*)), **3.63 – 3.71** (2.8H, m, C(2``,5``)H_2 (*major*)), **3.83 – 3.89** (1.2H, m, C(2``,5``)H_2 (*minor*)), **4.04** (1.4H, d, $^3J_{HH}$ = 7.4, C(1```)H_2 (*major*)), **4.58** (0.6H, d, $^3J_{HH}$ = 7.6, C(1```)H_2 (*minor*)), **4.69** (1.4H, s, CH_2CO$_2$Ar (*major*)), **4.88** (0.6H, s, CH_2CO$_2$Ar (*minor*)), **5.82** (0.7H, t, $^3J_{HH}$ = 7.4, C(2```)*H* (*major*)), **5.90** (0.3H, t, $^3J_{HH}$ = 7.6, C(2```)*H* (*minor*)), **7.09 – 7.17** (1H, m, ArC(4````)*H*), **7.28 – 7.43** (4H, m, ArC*H*), **7.46 – 7.52** (1.4H, m, ArC(2,6)*H* (*major*)), **7.53 – 7.58** (2H, m, ArC(2,6)*H* (*minor*)), **8.36 – 8.43** (2H, m, ArC(3,5)*H* (*major* + *minor*)); HRMS (ESI$^+$) $C_{24}H_{27}N_2O_4^+$ [M]$^+$: found 407.1957, required 407.1965 (–2.0 ppm).

D. Characterisation of rearrangement products

D. 1. Scope

(*R*,*E*)-*N*-Benzyl-5-phenyl-2-(pyrrolidin-1-yl)hex-4-enamide 3a

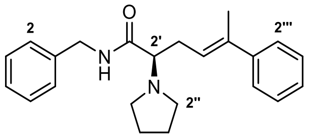

Following General Procedure **F**, (*S*)-tetramisole hydrochloride (9.60 mg, 0.04 mmol), triethylamine (45 µL, 0.32 mmol), (*E*)-1-(2-(4-nitrophenoxy)-2-oxoethyl)-1-(3-phenylbut-2-en-1-yl)pyrrolidin-1-ium bromide **2a** and benzylamine (33 µL, 0.30 mmol) in MeCN gave the crude product, which was purified by silica flash chromatography (CH_2Cl_2 : Et_2O 95:5 to 70:30) to give the title compound as a pale yellow solid (50 mg, 72%);

mp 154−156 °C; $[\alpha]_D^{20}$ = −45.3 (*c* 0.75, $CHCl_3$); Chiral HPLC analysis, Chiralcel OJ-H (98:2 hexane : IPA, flow rate 2 mLmin^{-1}, 254 nm, 40 °C) t_R (major): 14.3 min, t_R (minor): 24.2 min, 91:9 er; ν_{max} **(**film): 3292, 3061, 3030, 2965, 2803, 1653, 1522, 1495, 1454, 1244, 1138, 1028; ^{1}H-NMR (500 MHz, $CDCl_3$) δ_H: **1.71 – 1.78** (4H, m, C(3``,4``)H_2), **2.02** (3H, s, CH_3), **2.56 – 2.68** (5H, m, C(3`)H_AH$_B$ and C(2``, 5``)H_2), **2.76** (1H, ddd, $^2J_{HH}$ = 15.6, $^3J_{HH}$ = 7.70, 4.4, C(3`)H$_A$$H_B$, **3.04** (1H, dd, $^3J_{HH}$ = 6.6, 4.4, C(2`)*H*), **4.39** (1H, dd, $^2J_{HH}$ = 14.8, $^3J_{HH}$ = 5.6, NHCH_AH$_B$, **4.53** (1H, dd, $^2J_{HH}$ = 14.8, $^3J_{HH}$ = 6.3, NHCH$_A$$H_B$), **5.85** (1H, t, $^3J_{HH}$ = 7.2, C(4`)*H*), **7.17** (1H, s, N*H*), **7.21 – 7.25** (5H, m, ArC*H*), **7.27 – 7.34** (5H, m, ArC*H*); ^{13}C{^{1}H}-NMR (126 MHz, $CDCl_3$) δ_C: **16.2** (*C*H_3), **23.5** (C(3``,4``)H_2), **31.3** (*C*(3`)H_2), **43.3** (NH*C*H_2), **52.0** (*C*(2``,5``)H_2), **69.1** (*C*(2`)H), **123.4** (*C*(4`)H), **125.9** (Ar*C*(4)H), **126.9** (C4```), **127.4** (Ar*C*(2```,6```)H), **127.9** (Ar*C*(2,6)H), **128.3** (Ar*C*(3```,5```)H), **128.8** (Ar*C*(3,5)H), **136.9** (*C*(5`)), **138.5** (Ar*C*(1)), **143.7** (Ar*C*(1```)), **173.1** (*C*(1`)ONHBn); HRMS (NSI$^+$) $C_{23}H_{29}N_2O^+$ [M+H]$^+$: found 349.2277, required 349.2274 (+0.9 ppm).

4-Nitrophenyl (2*R*,3*R*)-3-methyl-3-phenyl-2-(pyrrolidin-1-yl)pent-4-enoate (*syn*-4a) and 4-nitrophenyl (2*R*,3*S*)-3-methyl-3-phenyl-2-(pyrrolidin-1-yl)pent-4-enoate (*anti*-4a)

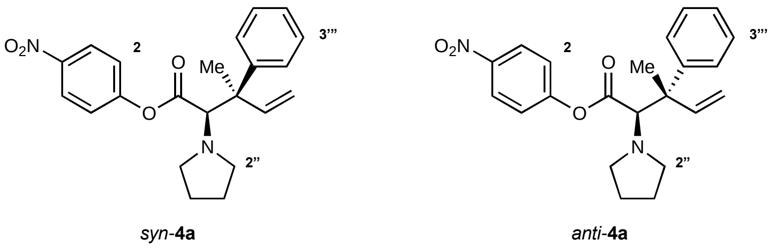

syn-**4a** *anti*-**4a**

Selected data for [2,3]-rearrangement product (crude ^{1}H NMR: 4:1 dr);

Major diastereoisomer syn-**4a**, Isolated as a pale-yellow solid (8 mg, 11%); mp 122−124 °C; $[\alpha]_D^{20}$ = −56.8 (*c* 1, $CHCl_3$); Chiral HPLC analysis, Chiralcel OJ-H (99.6:0.4 hexane : IPA, flow rate 2 mLmin^{-1}, 254 nm, 40 °C) t_R (2`*S*): 17.8 min, $t_R$ (2`*R*): 23.5 min, 22:78 er; ν_{max} (film): 2970, 1761, 1614, 1593, 1524, 1489, 1346, 1206, 1161, 1092, 1007; ^{1}H-NMR (500 MHz, $CDCl_3$) δ_H: **1.69** (3H, s, CH_3), **1.71 – 1.74** (4H, m, C(3``,4``)H_2), **2.70 – 2.85** (4H, m, C(2``,5``)H_2), **4.10** (1H, s, C(2`)*H*), **5.25** (1H, dd, $^3J_{HH}$ = 17.5, $^2J_{HH}$ = 1.2, C(5`)H_AH$_B$), **5.31** (1H, dd, $^3J_{HH}$ = 10.9, $^2J_{HH}$ = 1.2, C(5`)H$_A$$H_B$), **6.73** (1H, dd, $^3J_{HH}$ = 17.5, $^3J_{HH}$ = 10.9, C(4`)*H*), **6.84 – 6.89** (2H, m, ArC(2,6)*H*), **7.22 – 7.26** (1H, m, ArC(4```)*H*), **7.33** (2H, dd, $^3J_{HH}$ = 8.5, $^3J_{HH}$ = 7.0, ArC(2```,6```)*H*), **7.43 – 7.47** (2H, m, ArC(3```,5```)*H*), **8.14 – 8.19** (2H, m, ArC(3,5)*H*); ^{13}C{^{1}H}-NMR (126 MHz, $CDCl_3$) δ_C: **22.8** (*C*H_3), **23.9** (*C*(3``,4``)H_2), **48.0** (*C*(3`)),

52.3 (C(2``,5``)H_2), **74.1** (C(2`)H), **115.1** ($C$(5`)H_2), **122.7** (ArC(2,6)H), **125.2** (ArC(3,5)H), **126.7** (ArC(4```)H), **126.9** (ArC(2```,6```)H), **128.4** (ArC(3```,5```)H), **142.6** (C(4`)H), **145.4** (ArC(1```)), **146.3** (ArC(4)NO_2), **155.1** (ArC(1)O), **168.7** (C(1`)O_2Ar); HRMS (ESI$^+$) $C_{22}H_{24}N_2O_4^+$ $[M+H]^+$: found 381.1801, required 381.1808 (–1.8 ppm).

Minor diastereoisomer anti-**4a** (not isolated): ^{1}H-NMR (500 MHz, $CDCl_3$) δ_H: **1.71** (3H, s, CH_3), **1.73 – 1.79** (4H, m, C(3``,4``)H_2), **2.68 – 2.86** (4H, m, C(2``,5``)H_2), **4.03** (1H, s, C(2`)$H$), **5.09** (1H, dd, $^3J_{HH(trans)}$ = 17.5, $^2J_{HH}$ = 1.0, C(5`)$H_A$$H_B$), **5.21** (1H, dd, $^3J_{HH(cis)}$ = 10.8, $^2J_{HH}$ = 1.0, C(5`)$H_A$$H_B$), **6.52** (1H, dd, $^3J_{HH(trans)}$ = 17.5, $^3J_{HH(cis)}$ = 10.8), **6.73 – 6.77** (2H, m, ArC(2,6)H), **7.20 – 7.25** (1H, m, ArC(4```)H), **7.30 – 7.37** (2H, m, ArC(2```,6```)H), **7.42 – 7.47** (2H, m, ArC(3```,5```)H), **8.11 – 8.17** (2H, m, ArC(3,5)H).

(*R*,*E*)-*N*-Benzyl-5-phenyl-2-(pyrrolidin-1-yl)hept-4-enamide 3b

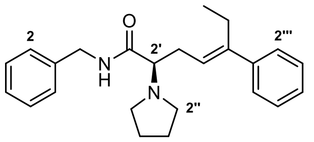

Following General Procedure **F**, (*S*)-tetramisole hydrochloride (9.6 mg, 40.0 µmol), triethylamine (45 µL, 0.32 mmol), (*E*)-1-(2-(4-nitrophenoxy)-2-oxoethyl)-1-(3-phenylpent-2-en-1-yl)pyrrolidin-1-ium bromide **2b** (95.0 mg, 0.200 mmol), benzylamine (33 µL, 0.300 mmol) in MeCN gave the crude product, which was purified by silica flash chromatography (CH_2Cl_2 : Et_2O 95:5 to 70:30) to give the title compound as a pale yellow solid (63 mg, 87%);

mp 89 – 91 °C ($CHCl_3$); $[\alpha]_D^{20}$ = −61.2 (*c* 1, $CHCl_3$); Chiral HPLC analysis, Chiralcel OJ-H (98.5:1.5 hexane : IPA, flow rate 2 mLmin^{-1}, 254 nm, 40 °C) t_R (*R*): 11.4 min, t_R (*S*): 16.5 min, 89:11 er; ν_{max} (film) 3291, 2965, 2930, 2803, 1653, 1516, 1495, 1454, 1333, 1292, 1238, 1136, 1030; ^{1}H-NMR (500 MHz, $CDCl_3$) δ_H: **0.95** (3H, t, $^3J_{HH}$ = 7.5, C(7`)H_3), **1.75** (4H, m, C(3``,5``)H_2), **2.51** (2H, s, C(6`)H_2), **2.57 – 2.67** (5H, m, C(2``,5``)H_2 and C(3`)$H_A$$H_B$), **2.77** (1H, ddd, $^2J_{HH}$ = 15.5, $^3J_{HH}$ = 8.0, 4.3, C(3`)$H_A$$H_B$), **3.02** (1H, dd, $^3J_{HH}$ = 6.5, 4.3, C(2`)$H$), **4.41** (1H, dd, $^2J_{HH}$ = 14.8, $^3J_{HH}$ = 5.7, NHC$H_A$$H_B$), **4.50** (1H, dd, $^2J_{HH}$ = 14.8, $^3J_{HH}$ = 6.2, NHC$H_A$$H_B$), **5.71** (1H, t, $^3J_{HH}$ = 7.1, C(4`)H), **7.08** (1H, s, NH), **7.22 – 7.25** (6H, m, ArCH), **7.27 – 7.30** (4H, m, ArCH); ^{13}C{^{1}H}-NMR (126 MHz, $CDCl_3$) δ_C: **13.6** (CH_3), **23.2** (C(6`)H_2), **23.5** (C(3``,4``)H_2), **30.9** (C(3`)H_2) **43.3** (NH$C$$H_2$Ph), **52.0** ($C$(2``,5``)), **69.2** ($C$(2`)H), **123.0** ($C$(4`)H), **126.5** (ArC(2```,6```)H), **126.8** (ArC(4```)H), **127.5** (ArC(4)H), **127.9** (ArC(2,6)H), **128.3** (ArC(3,5)H), **128.8** (ArC(3```,5```)H), **138.5** (C(5`)), **142.8** (ArC(1)), **143.8** (ArC(1```)), **173.2** (C(1`)ONHBn); HRMS (NSI$^+$) $C_{24}H_{30}N_2O$ $[M+H]^+$: found 363.2421, required 363.2431 (−2.8 ppm).

4-Nitrophenyl (2*R*,3*R*)-3-ethyl-3-phenyl-2-(pyrrolidin-1-yl)pent-4-enoate 4b

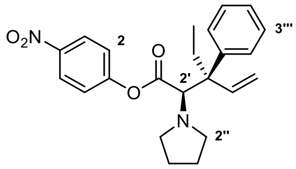

The [2,3]-rearrangement product **4b** was not isolated, but was adjudged to have formed in 9% by ^{1}H-NMR analysis of the crude reaction mixture using the following diagnostic signals (NB only one diastereoisomer was detected):

^{1}H-NMR (500 MHz, $CDCl_3$) δ_H: **6.63** (1H, dd, $^3J_{HH}$ = 17.8, 11.0, C(4`)H), **6.82** (2H, d, $^3J_{HH}$ = 9.1, ArC(2,6)H), **8.13** (2H, d, $^3J_{HH}$ = 9.1, ArC(3,5)H).

(*R*,*E*)-*N*-Benzyl-6-methyl-5-phenyl-2-(pyrrolidin-1-yl)hept-4-enamide 3c

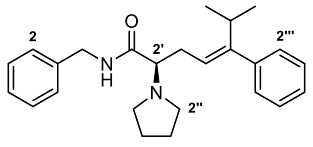

Following General Procedure **F**, (*S*)-tetramisole hydrochloride (14.4 mg, 60.0 μmol), triethylamine (67 μL, 0.48 mmol), (*E*)-1-(4-methyl-3-phenylpent-2-en-1-yl)-1-(2-(4-nitrophenoxy)-2-oxoethyl) pyrrolidin-1-ium bromide **2c** (147 mg, 0.30 mmol), benzylamine (49 μL, 0.45 mmol) in MeCN gave the crude product, which was purified by silica flash chromatography (CH_2Cl_2 : Et_2O 95:5 to 70:30) to give the title compound as a pale yellow oil (94 mg, 83%);

mp 82–84 °C; $[\alpha]_D^{20} = -57.7$ (*c* 1, $CHCl_3$); Chiral HPLC analysis, Chiralcel IC (98:2 hexane : IPA, flow rate 2 mLmin^{-1}, 254 nm, 40 °C) t_R (*R*): 26.4 min, t_R (*S*): 30.8 min, 87:13 er; ν_{max} (film): 2963, 2928, 2874, 1659, 1524, 1493, 1261; ^{1}H-NMR (400 MHz, CD_2Cl_2) δ_H: **1.01$_1$** (3H, d, $^3J_{HH}$ = 6.9, CH(C*H*$_3$)$_A$(CH$_3$)$_B$), **1.01$_4$** (3H, d, $^3J_{HH}$ = 7.1, CH(CH$_3$)$_A$(C*H*$_3$)$_B$), **1.69 – 1.78** (4H, m, C(3``,4``)*H*$_2$), **2.54 – 2.68** (5H, m, C(2``,5``)*H*$_2$ and C(3`)*H*$_A$H$_B$), **2.74** (1H, ddd, $^2J_{HH}$ = 15.6, $^3J_{HH}$ = 7.7, 4.5, C(3`)H$_A$*H*$_B$), **2.96** (1H, dd, $^3J_{HH}$ = 6.8, 4.5, C(2`)*H*), **3.02** (1H, app hept, $^3J_{HH}$ = 7.0, C*H*(CH$_3$)$_2$), **4.42** (2H, app d, $^3J_{HH}$ = 6.0, NHC*H*$_2$), **5.33** (1H, dd, $^3J_{HH}$ = 7.7, 6.5, C(4`)*H*), **7.08 – 7.12** (2H, m, C(2```,6```)*H*), 7.15 (1H, s (br), N*H*), **7.20 – 7.33** (8H, m, Ar*H*); ^{13}C{^{1}H}-NMR (101 MHz, CD_2Cl_2) δ_C: **22.0** (CH(*C*H$_3$)$_A$(CH$_3$)$_B$), **22.1** (CH(CH$_3$)$_A$(*C*H$_3$)$_B$), **23.9** (*C*(3``,4``)H$_2$), **29.9** (*C*H(CH$_3$)$_2$), **30.5** (*C*H$_2$NH), **43.5** (*C*(3`)H$_2$), **52.2** (*C*(2``,5``)H$_2$), **69.5** (*C*(2`)H), **124.6** (*C*(4`)H), **126.8** (Ar*C*H), **127.7** (Ar*C*H), **128.1** (Ar*C*(2,6)H), **128.2** (Ar*C*(2```,6```)H), **129.1** (Ar*C*(3,5)H), **129.2** (Ar*C*(3```,5```)H), **139.5** (ArC(1)), **143.7** (Ar*C*(1```)), **148.9** (*C*(5`)*i*-PrPh), **173.4** (*C*ONHBn); HRMS (ESI$^+$) $C_{25}H_{33}ON_2$ [M+H]$^+$: found 377.2578, required 377.2587 (−2.4 ppm).

4-Nitrophenyl (2*R*,3*R*)-3-isopropyl-3-phenyl-2-(pyrrolidin-1-yl)pent-4-enoate 4c

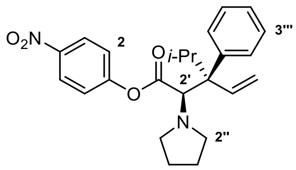

The [2,3]-rearrangement product **4c** was not isolated from the crude reaction residue, but was adjudged to have formed in 5% by ^{1}H-NMR analysis using the following diagnostic signals:

^{1}H-NMR (500 MHz, $CDCl_3$) δ_H: **6.33** (1H, dd, $^3J_{HH}$ = 18.0, 11.4, C(4`)*H*), **8.16** (2H, d, $^3J_{HH}$ = 9.0, ArC(3,5)*H*).

(*R*)-*N*-benzyl-4-(9*H*-fluoren-9-ylidene)-2-(pyrrolidin-1-yl)butanamide 3d

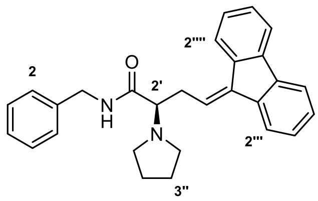

Following General Procedure **F**, (*S*)-tetramisole hydrochloride (9.6 mg, 40.0 μmol), triethylamine (45 μL, 0.32 mmol), 1-(2-(9*H*-fluoren-9-ylidene)ethyl)-1-(2-(4-nitrophenoxy)-2-oxoethyl)pyrrolidin-1-ium bromide **2d** (95.0 mg, 0.200 mmol), benzylamine (33 μL, 0.30 mmol) in MeCN gave the crude

product, which was purified by silica flash chromatography (CH_2Cl_2 : Et_2O 95:5 to 70:30) to give the title compound as a pale yellow solid (58 mg, 70%);

mp 156 °C (dec., $CHCl_3$); $[\alpha]_D^{20}$ = −13.8 (*c* 1, $CHCl_3$); Chiral HPLC analysis, Chiralcel ID (95:5 hexane : IPA, flow rate 1.5 $mLmin^{-1}$, 254 nm, 40 °C) tR (*S*): 26.5 min, tR (*R*): 30.4 min, 83:17 er; ^1H NMR (500 MHz, $CDCl_3$) δ_H: **1.72 – 1.80** (4H, m, C(3``,4``)H_2), **2.61 – 2.75** (4H, m NC(2``,5``)H_2), **3.28 – 3.43** (3H, m, C(2`)*H* and C(3`)H_2), **4.35** (1H, dd, $^3J_{HH}$ = 14.7, 5.2 Hz, NHCH_AH_B), **4.58** (1H, dd, $^3J_{HH}$ = 14.7, 6.7 Hz, NHCH_AH_B), **6.80 – 6.84** (1H, m, C(4`)*H*), **7.17 – 7.23** (5H, m, Ar*H*), **7.24** (1H, dd, *J* = 7.5, 1.1 Hz, Ar*H*), **7.27 – 7.30** (1H, m, Ar*H*), **7.33** (2H, tdd, *J* = 7.3, 5.6, 1.2 Hz, Ar*H*), **7.38** (1H, td, *J* = 7.5, 1.1 Hz, Ar*H*), **7.54** (1H, dt, *J* = 7.7, 0.9 Hz, Ar*H*), **7.71** (1H, dt, *J* = 7.5, 1.0 Hz, Ar*H*), **7.76** (1H, dt, *J* = 7.5, 1.0 Hz, Ar*H*), **7.89** (1H, d, *J* = 7.7 Hz, Ar*H*); $^{13}C\{^1H\}$ NMR (126 MHz, $CDCl_3$) δ_C: **23.5** (*C*(3``, 4``)H_2), **31.6** (*C*(3`)H_2),, **43.3** (NH*C*H_2), **51.9** (*C*(2``,5``)H_2), **68.4** (*C*(2`)H), **119.6** (*C*(4`)H), **120.0** (Ar*C*(5```)H), **120.1** (Ar*C*(5````)H), **125.2** (Ar*C*(2````)H), **125.6** (Ar*C*(2```)H), **127.0** (Ar*C*(3```)H), **127.2** (Ar*C*(3````)H), **127.5** (Ar*C*(4)H), **127.8** (Ar*C*(4````)H), **127.9** (Ar*C*(2,6)H)), **128.1** (Ar*C*(4```)H), **128.8** (Ar*C*(3,5)H), **136.7** (Ar*C*(6````)), **137.2** (Ar*C*(6```)), **138.4** (Ar*C*(1)), **138.8** (C(5`)), **139.3** (Ar*C*(1````)), **141.1** (Ar*C*(1```)), **172.8**, (*C*(1`)ONHBn); HRMS (ESI^+) $C_{28}H_{28}N_2O$ $[M+H]^+$: found 409.2266, required 409.2274.

(*R*)-*N*-benzyl-5,5-diphenyl-2-(pyrrolidin-1-yl)pent-4-enamide 3e

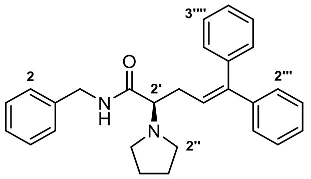

Following General Procedure **F**, (*S*)-tetramisole hydrochloride (12.0 mg, 50.0 μmol), triethylamine (56 μL, 0.400 mmol), 1-(3,3-diphenylallyl)-1-(2-(4-nitrophenoxy)-2-oxoethyl)pyrrolidin-1-ium bromide **2e** (131 mg, 0.250 mmol), benzylamine (41 μL, 0.375 mmol) in MeCN gave the crude product, which was purified by silica flash chromatography (CH_2Cl_2 : Et_2O 95:5 to 70:30) to give the title compound as an off-white solid (69 mg, 67%);

mp 117 – 119 °C ($CHCl_3$); $[\alpha]_D^{20}$ = −36.0 (*c* 1, $CHCl_3$); Chiral HPLC analysis, Chiralcel AD-H (98:2 hexane : IPA, flow rate 2 $mLmin^{-1}$, 254 nm, 40 °C) tR (*S*): 27.0 min, tR (*R*): 33.8 min, 77:23 er; ν_{max} (film): 3298, 3055, 3028, 2965, 2930, 2805, 1651, 1518, 1495, 1445, 1333, 1244, 1134, 1074, 1030; ^1H-NMR (500 MHz, $CDCl_3$) δ_H: **1.66 – 1.73** (4H, m, C(3``,4``)H_2), **2.46 – 2.59** (5H, m, C(2``,5``)H_2 and C(3`)$H_AH_B$), **2.66** (1H, ddd, $^2J_{HH}$ = 15.4, $^3J_{HH}$ = 7.7, 4.7, C(3`)H_AH_B), **2.98** (1H, dd, $^3J_{HH}$ = 7.7, 4.7, C(2`)*H*), **4.41** (1H, dd, $^2J_{HH}$ = 14.7, $^3J_{HH}$ = 5.6, NHC$H_AH_B$), **4.53** (1H, dd, $^2J_{HH}$ = 14.7, $^3J_{HH}$ = 6.3, NHC$H_AH_B$), **6.19** (1H, dd, $^3J_{HH}$ = 7.6, 6.4, C(4`)*H*), **7.11** (1H, brs, N*H*), **7.11 – 7.15** (2H, m, ArC*H*), **7.16 – 7.20** (2H, m, ArC*H*), **7.22 – 7.27** (8H, m, ArC*H*), **7.28 – 7.32** (1H, m, ArC(4)*H*), **7.33 – 7.38** (2H, m, ArC*H*); $^{13}C\{^1H\}$-NMR (126 MHz, $CDCl_3$) δ_C: **23.4** (*C*(3``,4``)H_2), **32.1** (*C*(3`)H_2), **43.3** (NH*C*H_2), **51.7** (*C*(2``,5``)H_2), **69.1** (*C*(2`)H), **125.1** (*C*(4`)H), **127.1** (*C*(4```)H), **127.2** (Ar*C*(4````)H), **127.4** (Ar*C*(2,6)H), **127.5** (Ar*C*(4)H), **128.0** (Ar*C*(2```,6```)H), **128.2** (Ar*C*(2````,6````)H), **128.4** (Ar*C*(3,5)H), **128.8** (Ar*C*(3````,5````)H), **130.0** (Ar*C*(3```,5```)H), **138.6** (Ar*C*(1)), **139.9** (C(5`)), **142.6** (Ar*C*(1````)), **143.2** (Ar*C*(1```)), **173.1** (*C*(1`)ONHBn); HRMS: (NSI^+) $C_{28}H_{31}N_2O$ $[M+H]^+$: found 411.2427, required 411.2431 (–1.0 ppm).

(*R*,*E*)-*N*-benzyl-5-phenyl-2-(piperidin-1-yl)hex-4-enamide 3f

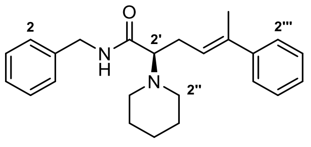

Following a modified General Procedure **F**, (*S*)-tetramisole hydrochloride (9.6 mg, 0.040 mmol), triethylamine (45 µL, 0.32 mmol), (*E*)-1-(2-(4-nitrophenoxy)-2-oxoethyl)-1-(3-phenylbut-2-en-1-yl)piperidin-1-ium bromide **2f** (95.1 mg, 0.20 mmol) and then benzylamine (33 µL, 0.30 mmol) in MeCN gave the crude product, which was purified by silica flash chromatography (CH_2Cl_2 : Et_2O 95:5 to 70:30) to give the title compound as an off-white solid (36 mg, 50%);

mp 95 – 97 °C ($CHCl_3$); Chiral HPLC analysis, $[\alpha]_D^{20}$ = −49.9 (*c* 1, $CHCl_3$); Chiralcel OJ-H (98:2 hexane : IPA, flow rate 2 $mLmin^{-1}$, 254 nm, 40 °C) t_R (*R*): 10.2 min, t_R (*S*): 14.5 min, 93:6 er; ^1H-NMR (500 MHz, $CDCl_3$) δ_H: **1.41 – 1.55** (6H, m, C(3``,4``,5``)$H_2$), **2.05** (3H, s, C$H_3$), **2.49** (2H, brs, C(2``,6``)$H_AH_B$), **2.57 – 2.66** (3H, m, C(2``,6``)H_AH_B and C(3`)$H_AH_B$), **2.77** (1H, dt, $^2J_{HH}$ = 13.2, $^3J_{HH}$ = 6.2, C(3`)H_AH_B), **3.15** (1H, t, $^3J_{HH}$ = 6.1, C(2`)), **4.40** (1H, dd, $^2J_{HH}$ = 14.9, $^3J_{HH}$ = 5.7, NHC$H_AH_B$), **4.52** (1H, dd, $^2J_{HH}$ = 14.9, $^3J_{HH}$ = 6.2, NHC$H_AH_B$), **5.89** (1H, t, $^3J_{HH}$ = 7.1, C(4`)*H*), **7.20 – 7.25** (5H, m, ArC*H*), **7.28 – 7.35** (5H, m, ArC*H*); $^{13}C\{^1H\}$-NMR (101 MHz, $CDCl_3$) δ_C: **16.1** (CH_3), **24.3** (*C*(4``)$H_2$), **26.6** (*C*(3``,5``)$H_2$), **27.1** (*C*(3`)$H_2$), **43.4** (NH$CH_2$Ph), **51.8** (*C*(2``,6``)H_2), **69.7** (*C*(2`)H), **125.4** (*C*(4`)), **125.8** (Ar*C*(2```6```)H), **126.8** (Ar*C*(4)H), **127.4** (Ar*C*(4```)H), **127.7** (Ar*C*(2,6)H), **128.0** (*C*(5`)) , **128.3** (Ar*C*(3,5)H), **128.8** (Ar*C*(3```,5```)H), **138.8** (Ar*C*(1)), **143.8** (Ar*C*(1```)), **173.1** (*C*(1`)ONHBn); HRMS (NSI^+) $C_{24}H_{31}N_2O$ $[M+H]^+$: found 363.2433, required 363.2431 (+0.6 ppm).

4-Nitrophenyl (2*R*,3*R*)-3-methyl-3-phenyl-2-(piperidin-1-yl)pent-4-enoate *syn*-4f and 4-nitrophenyl (2*R*,3*S*)-3-methyl-3-phenyl-2-(piperidin-1-yl)pent-4-enoate *anti*-4f

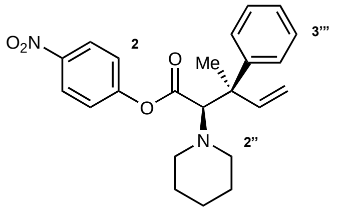

syn-**4f**

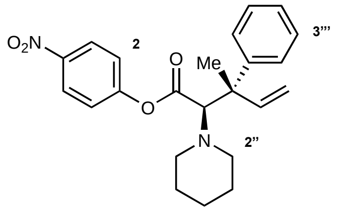

anti-**4f**

Selected data for the [2,3]-rearrangement product (NMR yield = 22%, 1.5:1 dr), which decomposed upon subjection to silica gel column chromatography:

Major diastereoisomer syn-**4f**: ^1H NMR (400 MHz, $CDCl_3$) δ_H: **1.68** (3H, s, CH_3), **5.30** (1H, dd, $^3J_{HH}$ = 17.7, $^2J_{HH(trans)}$ = 1.3, C(5`)$H_AH_B$), **5.35** (1H, dd, $^3J_{HH}$ = 11.1, $^2J_{HH}$ = 1.3, C(5`)H_AH_B) **6.77** (1H, dd, $^3J_{HH}$ = 17.7, 11.1, C(4`)*H*), **6.92 – 6.98** (2H, m, ArC(2,6)*H*), **8.13 – 8.19** (2H, m, ArC(3,5)*H*).

Minor diastereoisomer anti-**4f**: ^1H NMR (400 MHz, $CDCl_3$) δ_H: **6.44** (1H, dd, $^3J_{HH}$ = 17.6, $^3J_{HH}$ = 10.8, C(4`)*H*), **6.85** (2H, d, $^3J_{HH}$ = 9.1, ArC(2,6)*H*).

(*R*,*E*)-2-(Azepan-1-yl)-*N*-benzyl-5-phenylhex-4-enamide 3g

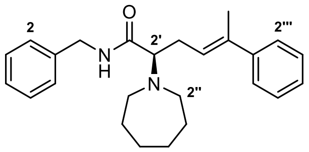

Following a modified General Procedure **F**, (*S*)-tetramisole hydrochloride (9.6 mg, 0.040 mmol), triethylamine (45 µL, 0.32 mmol), (*E*)-1-(2-(4-nitrophenoxy)-2-oxoethyl)-1-(3-phenylbut-2-en-1-yl)azepan-1-ium bromide **2g** (97.9 mg, 0.20 mmol) and benzylamine (33 µL, 0.30 mmol) in MeCN at gave the crude product, which was purified by silica flash chromatography (CH_2Cl_2 : Et_2O 95:5 to 60:40) to give the title compound as an off-white solid (29 mg, 39%);

mp 96 – 98 °C ($CHCl_3$); $[\alpha]_D^{20}$ = −66.2 (*c* 1, $CHCl_3$); Chiral HPLC analysis, Chiralcel OJ-H (98:2 hexane : IPA, flow rate 2 mLmin^{-1}, 254 nm, 40 °C, t_R (*R*): 9.4 min, t_R (*S*): 14.7 min, 92:8 er; ν_{max} (film): 3298, 3028, 2920, 2851, 1651, 1514, 1495, 1452, 1445, 1358, 1263, 1240, 1153, 1130, 1078, 1028; ^{1}H-NMR (500 MHz, $CDCl_3$) δ_H: **1.50 – 1.62** (8H, m, C(3``,4``,5``,6``)H_2), **2.07** (3H, d, $^4J_{HH}$ = 1.3, CH_3), **2.62** (1H, dt, $^2J_{HH}$ = 15.3, $^3J_{HH}$ = 7.6, C(3`)H_AH$_B$), **2.66 – 2.80** (4H, m, C(2``,7``)H_2), **2.88** (1H, dt, $^2J_{HH}$ = 15.3, $^3J_{HH}$ = 6.3, C(3`)H$_A$$H_B$), **3.39** (1H, appt, $^3J_{HH}$ = 6.5, C(2`)*H*), **4.40** (1H, dd, $^2J_{HH}$ = 14.8, $^3J_{HH}$ = 5.6, NHCH_AH$_B$Ph), **4.53** (1H, dd, $^2J_{HH}$ = 14.8, $^3J_{HH}$ = 6.4, NHCH$_A$$H_B$Ph), **5.92** (1H, ddq, $^3J_{HH}$ = 7.7, $^3J_{HH}$ = 6.3, $^4J_{HH}$ = 1.3, C(4`)*H*), **7.20 – 7.25** (1H, m, ArC*H*), **7.26 – 7.34** (7H, m, ArC*H*), **7.34 – 7.38** (2H, m, ArC*H*), **7.61** (1H, t, $^3J_{HH}$ = 6.1, N*H*); ^{13}C{^{1}H}-NMR (126 MHz, $CDCl_3$) δ_C: **16.0** (*C*H$_3$), **26.8** (*C*(4``,5``)H$_2$), **26.9** (*C*(3`)H$_2$), **29.6** (*C*(3``,6``)H_2), **43.5** (NH*C*H$_2$Ph), **53.6** (*C*(2``,7``)), **70.5** (*C*(2`)H), **125.8** (Ar*C*(2```,6```)H), **126.1** (*C*(4`)H), **126.7** (Ar*C*(4)H), **127.5** (Ar*C*(4```)H), **127.8** (Ar*C*(2,6)), **128.3** (Ar*C*(3,5)H), **128.8** (Ar*C*(3```,5```)H), **135.3** (*C*(5`)), **138.8** (Ar*C*(1)), **143.9** (ArC(1```)), **173.4** (*C*(1`)ONHBn); HRMS (ESI$^+$) $C_{25}H_{32}N_2O$ $[M+H]^+$: found 377.2579, required 377.2587 (–2.1 ppm).

4-Nitrophenyl (2*R*,3*R*)-2-(azepan-1-yl)-3-methyl-3-phenylpent-4-enoate *syn*-4g and 4-nitrophenyl (2*R*,3*S*)-2-(azepan-1-yl)-3-methyl-3-phenylpent-4-enoate *anti*-4g

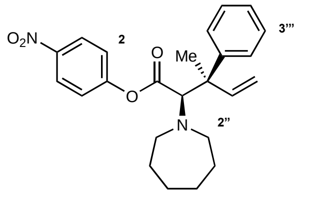

syn-**4g**

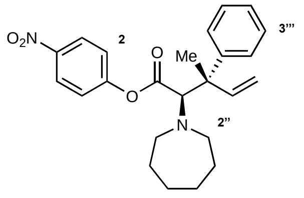

anti-**4g**

Selected data for the [2,3]-rearrangement product (NMR yield = 4%, 70:30 dr):

Major diastereoisomer syn-**4g**: ^{1}H-NMR (500 MHz, $CDCl_3$) δ_H: **4.01** (1H, s, C(2`)*H*), **5.30** (1H, d, $^3J_{HH(trans)}$ = 17.6, C(5`)H_AH$_B$), **5.35** (1H, d, $^3J_{HH(cis)}$ = 11.0, C(5`)H$_A$$H_B$), **5.92** (1H, dd, $^3J_{HH(trans)}$ = 17.6, $^3J_{HH(cis)}$ = 11.0, C(4`)*H*);

Minor diastereoisomer anti-**4g**: ^{1}H-NMR (500 MHz, $CDCl_3$) δ_H: **3.98** (1H, s, C(2`)*H*), **5.17** (1H, d, $^3J_{HH(cis)}$ = 11.3, C(5`)H$_A$$H_B$), **6.57** (1H, dd, $^3J_{HH(trans)}$ = 17.4, $^3J_{HH(cis)}$ = 11.3, C(4`)*H*), **6.86 – 6.88** (1H, m, ArC(2,6)*H*).

(*R*)-*N*-Benzyl-2-(dimethylamino)-5,5-diphenylpent-4-enamide 3h

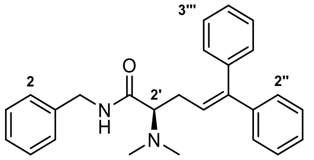

Following General Procedure **F**, (*S*)-tetramisole hydrochloride (9.6 mg, 0.040 mmol), triethylamine (45 μL, 0.32 mmol), *N*,*N*-dimethyl-*N*-(2-(4-nitrophenoxy)-2-oxoethyl)-3,3-diphenylprop-2-en-1-aminium bromide **2h** (99.5 mg, 0.200 mmol) and benzylamine (33 μL, 0.30 mmol) in MeCN gave the crude product, which was purified by silica flash chromatography (CH_2Cl_2 : Et_2O 95:5 to 70:30) to give the title compound as an off-white solid (35 mg, 46%);

mp 131 – 133 °C ($CHCl_3$); $[\alpha]_D^{20}$ = −32.2 (*c* 1, $CHCl_3$); Chiral HPLC analysis, Chiralcel ID (98:2 hexane : IPA, flow rate 2 mLmin^{-1}, 254 nm, 40 °C) t_R (*R*): 23.3 min, t_R (*S*): 26.8 min, 71:29 er; ^{1}H-NMR (500 MHz, $CDCl_3$) δ_H: **2.18** (6H, s, N(CH_3)$_2$), **2.56** (1H, ddd, $^2J_{HH}$ = 15.5, $^3J_{HH}$ = 8.2, 5.7, C(3`)$H_AH_B$), **2.63** (1H, dd, $^2J_{HH}$ = 15.5, $^3J_{HH}$ = 6.5, C(3`)H_AH_B), **3.03** (1H, t, $^3J_{HH}$ = 6.5, C(2`)*H*), **4.41** (1H, dd, $^2J_{HH}$ = 14.7, $^3J_{HH}$ = 5.7, NHC$H_AH_B$), **4.50** (1H, dd, $^2J_{HH}$ = 14.7, $^3J_{HH}$ = 6.2, NHC$H_AH_B$), **6.26** (1H, dd, $^3J_{HH}$ = 8.1, 6.3, C(4`)*H*), **7.16 – 7.21** (4H, m, ArC*H*), **7.22 – 7.26** (3H, m, ArC*H*), **7.27 – 7.28** (3H, m, ArC*H*), **7.28 – 7.33** (2H, m, ArC*H*), **7.34 – 7.40** (3H, m, ArC*H*); ^{13}C{^{1}H}-NMR (126 MHz, $CDCl_3$) δ_C: **28.1** (*C*(3`)$H_2$), **42.7** (N(*C*$H_3$)$_2$), **43.4** (NH*C*$H_2$Ph), **69.9** (*C*(2`)H), **126.5** (*C*(4`)H), **127.1** (Ar*C*(4)H), **127.2** (Ar*C*(4```)H), **127.3** (Ar*C*(2,6)H), **127.5** (ArC(4``)H), **128.0** (Ar*C*(2```,6```)H), **128.2** (Ar*C*(2``,6``)H), **128.4** (Ar*C*(3,5)H), **128.8** (Ar*C*(3```,5```)H), **130.0** (Ar*C*(3``,5``)H), **138.6** (*C*(5`)), **140.0** (Ar*C*(1)H), **142.5** (Ar*C*(1```)H), **142.7** (Ar*C*(1``)), **172.7** (*C*(1`)ONHBn); HRMS (ESI$^+$) $C_{26}H_{28}N_2O$ [M+H]$^+$: found 385.2270, required 385.2274 (–1.0 ppm).

(*R*,*E*)-*N*-Benzyl-2-(dimethylamino)-5-phenylhex-4-enamide 3i

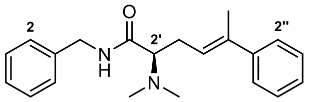

Following General Procedure **F**, (*S*)-tetramisole hydrochloride (9.6 mg, 0.04 mmol), triethylamine (45 μL, 0.32 mmol), (*E*)-*N*,*N*-dimethyl-*N*-(2-(4-nitrophenoxy)-2-oxoethyl)-3-phenylbut-2-en-1-aminium bromide **2i** (87 mg, 0.2 mmol) and benzylamine (33 μL, 0.15 mmol) in MeCN gave the crude product, which was purified by silica flash chromatography (CH_2Cl_2 : Et_2O 95:5 to 70:30) to give the title compound as an off-white solid (16 mg, 25%);

mp 109 – 112 °C ($CHCl_3$); $[\alpha]_D^{20}$ = −40.7 (*c* 1, $CHCl_3$); Chiral HPLC analysis, Chiralcel OJ-H (98:2 hexane : IPA, flow rate 2 mLmin^{-1}, 254 nm, 40 °C) t_R (*R*): 19.3 min, t_R (*S*): 31.1 min, 90:10 er; ν_{max} (film): 3300, 3292, 2924, 2854, 2783, 1643, 1518, 1495, 1454, 1445, 1379, 1360, 1244, 1153, 1028; ^{1}H-NMR (500 MHz, $CDCl_3$) δ_H: **2.05** (3H, s, C(6`)$H_3$), **2.32** (6H, s, N(C$H_3$)$_2$), **2.60 – 2.77** (2H, m, C(3`)H_2), **3.06** (1H, t, $^3J_{HH}$ = 5.9, C(2`)*H*), **4.41** (1H, dd, $^2J_{HH}$ = 14.7, $^3J_{HH}$ = 5.6, NHC$H_AH_B$), **4.50** (1H, dd, $^2J_{HH}$ = 14.7, $^3J_{HH}$ = 6.2, NHC$H_AH_B$), **5.87** (1H, t, $^3J_{HH}$ = 7.8, C(4`)*H*), **7.20 – 7.25** (5H, m, Ar*H*), **7.28 – 7.34** (4H, m, Ar*H*), **7.35** (1H, brs, N*H*); ^{13}C{^{1}H}-NMR (126 MHz, $CDCl_3$) δ_C: **16.1** (*C*(6`)$H_3$), **27.9** (*C*(3`)H_2), **43.0** (N(*C*H_3)$_2$), **43.4** (NH*C*H_2), **69.9** (*C*(2`)H), **124.6** (*C*(4`)H), **125.8** (Ar*C*(2``,6``)H), **126.8** (Ar*C*(4)H), **127.5** (Ar*C*(4``)H), **128.0** (Ar*C*(2,6)H), **128.3** (Ar*C*(3,5)H, **128.8** (Ar*C*(3``,5``)H), **136.2** (*C*(5`)), **138.6** (Ar*C*(1)), **143.8** (Ar*C*(1``)), **172.9** (C(1`)ONHBn); HRMS (ESI$^+$) $C_{21}H_{27}N_2O$ [M+H]$^+$: found 323.2112, required 323.2118 (–1.9 ppm).

4-Nitrophenyl (2*R*,3*R*)-2-(dimethylamino)-3-methyl-3-phenylpent-4-enoate *syn*-4i and 4-nitrophenyl (2*R*,3*S*)-2-(dimethylamino)-3-methyl-3-phenylpent-4-enoate *anti*-4i

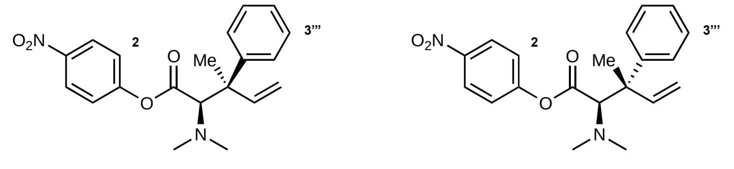

syn-**4i** *anti*-**4i**

Data for [2,3]-rearrangement product (76:24 dr):

Major diastereoisomer syn-**4i**: Isolated as a yellow oil (35 mg, 49%); Chiral HPLC analysis, Chiralcel OJ-H (98:2 hexane : IPA, flow rate 2 mLmin^{-1}, 254 nm, 40 °C) t_R (2`*R*): 19.3 min, $t_R$ (2`*S*): 31.1 min, 90:10 er; ν_{max} (film): 3318, 3082, 2982, 1763, 1694, 1589, 1516, 1487, 1443, 1335, 1288, 1238, 1165, 1107, 1045, 1007; ^{1}H-NMR (500 MHz, $CDCl_3$) δ_H: **1.63** (3H, s, C(3`)C*H*$_3$), **2.52** (6H, s, N(C*H*$_3$)$_2$), **3.92** (1H, s, C(2`)*H*), **5.30** (1H, dd, $^3J_{HH}$ = 17.7, $^2J_{HH}$ = 1.3, C(5`)*H*$_A$H$_B$), **5.38** (1H, dd, $^3J_{HH}$ = 10.9 , $^2J_{HH}$ = 1.2 , C(5`) H$_A$*H*$_B$), **6.74 – 6.82** (1H, m, C(4`)*H*), **6.84 – 6.88** (2H, m, ArC(2,6)*H*), **7.22 – 7.25** (1H, m, ArC(4``)*H*), **7.31 – 7.37** (2H, m, ArC(2``,6``)*H*), **7.40** (2H, m, ArC(3``,5``)*H*), **8.15 – 8.19** (2H, m, ArC(3,5)*H*); $^{13}$C{$^1$H}-NMR (101 MHz, $CDCl_3$) $\delta_C$: **23.9** (*C*H$_3$), **45.0** (*C*(3`)), **48.0** (N(*C*H$_3$)$_2$), **76.2** (*C*(2`)HN(CH$_3$)$_2$), **115.9** (*C*(5`)H$_2$), **122.7** (Ar*C*(2,6)H), **125.2** (Ar*C*(3,5)H), **126.7** (Ar*C*(4``)H), **126.8** (Ar*C*(2``,6``)H), **128.5** (Ar*C*(3``,5``)H), **141.8** (*C*(4`)H), **145.4** (Ar*C*(4)NO$_2$), **146.3** (Ar*C*(1``)), **155.0** (Ar*C*(1)), **167.9** (*C*(1`)O$_2$Ar); HRMS (ESI$^+$) $C_{20}H_{22}N_2O_4$ [M+H]$^+$: found 355.1640, required 355.1652 (–3.6 ppm).

Minor diastereoisomer *anti*-**4i** (not isolated): ^{1}H NMR (400 MHz, $CDCl_3$) δ_H: **1.72** (3H, s, C(4`)*H*$_3$), **2.52** (6H, s, N(C*H*$_3$)$_2$), **3.90** (1H, s, C(2`)*H*), **5.01** (1H, dd, $^3J_{HH(trans)}$ = 17.6, $^2J_{HH}$ = 1.0 Hz, C(5`)*H*$_A$H$_B$), **5.19** (1H, dd, $^3J_{HH(cis)}$ = 10.9, $^2J_{HH(trans)}$ = 1.0 Hz, C(5`)H$_A$*H*$_B$), **6.45** (1H, dd, $^3J_{HH(trans)}$ = 17.5, $^3J_{HH(cis)}$ = 10.9 Hz, C(4`)*H*).

4-Nitrophenyl (2*R*,3*R*)-3-methyl-2-morpholino-3-phenylpent-4-enoate *syn*-4j and 4-nitrophenyl (2*R*,3*S*)-3-methyl-2-morpholino-3-phenylpent-4-enoate *anti*-4j

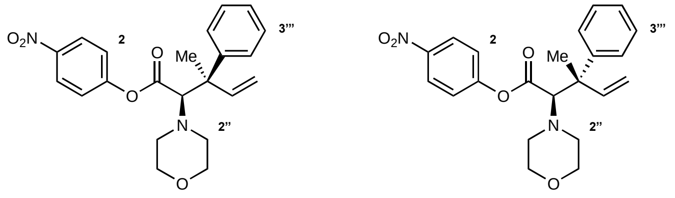

syn-**4j** *anti*-**4j**

Following General Procedure **F**, (*S*)-tetramisole hydrochloride (9.6 mg, 0.04 mmol), triethylamine (45 µL, 0.32 mmol), (*E*)-4-(2-(4-Nitrophenoxy)-2-oxoethyl)-4-(3-phenylbut-2-en-1-yl)morpholin-4-ium bromide **2j** and benzylamine (33 µL, 0.30 mmol) in MeCN at gave the crude product (89:11 *syn*:*anti*), which was purified by silica flash chromatography (CH_2Cl_2 : Et_2O 95:5 to 70:30) to give:

Syn-**4j** (38 mg, 48%) as an off-white solid; mp,: 130 – 132 °C ($CHCl_3$); $[\alpha]_D^{20}$ = −23.1° (*c* 1, $CHCl_3$); Chiral HPLC analysis, Chiralcel OJ-H (99:1 hexane : IPA, flow rate 2 mLmin^{-1}, 254 nm, 40 °C, t_R (*R*): 33.7 min, t_R (*S*): 52.3 min, 77:23 er; ν_{max} (film): 2965, 2855, 1763, 1616, 1591, 1524, 1489, 1447, 1346, 1206, 1161, 1115, 1096, 1009; ^{1}H-NMR (500 MHz, $CDCl_3$) δ_H: **1.61** (3H, s, C*H*$_3$), **2.53 – 2.59** (2H, m, NC(2``,6``)*H*$_A$H$_B$), **3.04 – 3.08** (2H, m, NC(2``,6``)H$_A$*H*$_B$), **3.65 – 3.75** (4H, m, OC(3``,5``)*H*$_2$), **3.85** (1H, s, C(2`)*H*), **5.31** (1H, dd, $^3J_{HH(trans)}$ = 17.7 , $^2J_{HH}$ = 1.3, C(5`)*H*$_A$H$_B$), **5.37** (1H, dd, $^3J_{HH(cis)}$ = 10.9, $^2J_{HH}$ = 1.3, C(5`)*H*$_A$H$_B$), **6.76** (1H, dd, $^3J_{HH}$ = 17.7, 10.9, C(4`)*H*), **6.88 – 6.92** (2H, m, ArC(2,6)*H*), **7.23**

– **7.27** (1H, m, ArC(4```)*H*), **7.34** (2H, dd, $^3J_{HH}$ = 8.6, 6.9, ArC(3```,5```)*H*), **7.39 – 7.43** (2H, m, ArC(2```,6```)*H*), **8.15 – 8.20** (2H, m, ArC(3,5)*H*); ^{13}C{^{1}H}-NMR (126 MHz, $CDCl_3$) δ_C: **24.2** (*C*H_3), **48.2** (*C*(3`)), **53.3** (N*C*(2``,6``)H_2), **67.6** (O*C*(3``,5``)H_2), **76.3** (*C*(2`)), **116.1** (*C*(5`)H_2), **122.8** (Ar*C*(2,6)H), **125.3** (Ar*C*(3,5)H), **126.7** (Ar*C*(2```,6```)H), **126.8** (Ar*C*(4```)H), **128.6** (Ar*C*(3```,5```)H), **141.4** (Ar*C*(4)NO_2), **145.6** (Ar*C*(1```)), **145.9** (*C*(4`)H), **154.9** (Ar*C*(1)O), **167.4** (*C*(1`)O_2Ar); HRMS (ESI$^+$) $C_{22}H_{24}N_2O_5$ [M+H]$^+$: found 397.1751, required 397.1758 (−1.8 ppm).

Selected data for the minor diastereoisomer (not isolated), *anti*-**4j**: ^{1}H-NMR (400 MHz, $CDCl_3$) δ_H: **1.70** (3H, s, C*H*$_3$), **2.61 – 2.67** (2H, m, NC(2``,6``)*H*$_A$H$_B$), **2.88 – 2.97** (2H, m, NC(2``,6``)H$_A$*H*$_B$), **3.68 – 3.78** (4H, m, OC(3``,5``)*H*$_2$), **3.83** (1H, s, C(2`*H*)), **4.99** (1H, dd, $^3J_{HH(trans)}$ = 17.5, $^2J_{HH}$ = 1.0, C(5`)*H*$_A$H$_B$), **5.18** (1H, dd, $^3J_{HH(cis)}$ = 10.8, $^2J_{HH}$ = 1.0, C(5`)H$_A$*H*$_B$), **6.45** (1H, dd, $^3J_{HH}$ = 17.5, 10.8, C(4`)*H*), **6.82 – 6.87** (2H, m, ArC(2,6)*H*), **7.25 – 7.28** (1H, m, ArC*H*), **7.32 – 7.35** (2H, ArC*H*), **7.42 – 7.46** (2H, m, ArC*H*), **8.17 – 8.20** (2H, m, ArC(3,5)*H*).

(*R*,*E*)-*N*-Benzyl-2-morpholino-5-phenylhex-4-enamide 3j

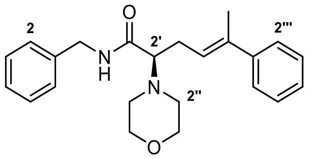

[1,2]-rearrangement product could not be isolated from the crude reaction mixture, but was assigned based on the following characteristic signals (NMR yield = 9%):

^{1}H-NMR (400 MHz, $CDCl_3$) δ_H: **2.08** (3H, q, $^4J_{HH}$ = 1.3, C*H*$_3$), **5.88** (1H, tq, $^3J_{HH}$ = 6.8, $^4J_{HH}$ = 1.3, C(4`)*H*).

(*R*,*E*)-*N*-Benzyl-2-(pyrrolidin-1-yl)-5-(*p*-tolyl)hex-4-enamide 3k

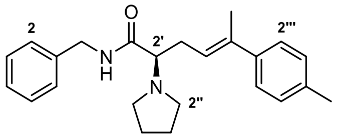

Following General Procedure **F**, (*S*)-tetramisole hydrochloride (4.8 mg, 0.020 mmol), triethylamine (22 µL, 0.16 mmol), (*E*)-1-(2-(4-nitrophenoxy)-2-oxoethyl)-1-(3-(p-tolyl)but-2-en-1-yl)pyrrolidin-1-ium bromide **2k** (47.5 mg, 0.020 mmol), benzylamine (55 µL, 0.50 mmol) in MeCN gave the crude product, which was purified by silica flash chromatography (CH_2Cl_2 : Et_2O 95:5 to 70:30) to give the title compound as a pale yellow solid (42 mg, 58%);

mp 98 − 100 °C; $[\alpha]_D^{20}$ = −58.5 (*c* 1.1, $CHCl_3$); Chiral HPLC analysis, Chiralcel OJ-H (97:3 hexane : IPA, flow rate 2 mLmin^{-1}, 40 °C) t_R (*R*): 9.1 min, t_R (*S*): 11.2 min, 91:9 er; ν_{max} (film): 3291, 2965, 2920, 2799, 1651, 1512, 1454, 1244, 1136, 1028; ^{1}H-NMR (500 MHz, $CDCl_3$) δ_H: **1.72 – 1.81** (4H, m, C(3``,4``)*H*$_2$), **2.00** (3H, d, $^4J_{HH}$ = 1.2, C(5`)*H*$_3$), **2.34** (3H, s, ArC*H*$_3$), **2.57 - 2.69** (5H, m, C(2``,5``)*H*$_2$ and C(3`)*H*$_A$H$_B$), **2.71 – 2.79** (1H, ddd, $^2J_{HH}$ = 15.3, $^3J_{HH}$ = 7.7, $^4J_{HH}$ = 4.4, C(3`)H$_A$*H*$_B$), **3.04** (1H, dd, $^3J_{HH}$ = 6.6, $^3J_{HH}$ = 4.4, C(2`)*H*), **4.40** (1H, dd, $^2J_{HH}$ = 14.8, $^3J_{HH}$ = 5.7, NHC*H*$_A$H$_B$), **4.51** (1H, dd, $^2J_{HH}$ = 14.8, $^3J_{HH}$ = 6.3, NHCH$_A$*H*$_B$), **5.81** (1H, t, $^3J_{HH}$ = 6.5, C(4`)*H*), **7.08 – 7.12** (3H, m, N*H* and ArC(3```,5```)*H*), **7.21** (2H, d, $^3J_{HH}$ = 8.2, ArC(2```,6```)*H*), **7.23 – 7.25** (5H, m, ArC*H*); ^{13}C{^{1}H}-NMR (126 MHz, $CDCl_3$) δ_C: **16.2** (*C*(5`)H_3), **21.2** (ArC*C*H_3), **23.5** (*C*(3``,4``)H_2), **31.3** (*C*(3`)H_2), **43.3** (NH*C*H_2Ph), **51.9** (*C*(2``,5``)H_2), **69.1** (*C*(2`)H), **122.7** (*C*(4`)H), **125.7** (Ar*C*(2```,6```)H), **127.4** (Ar*C*(3```,5```)H), **127.9** (Ar*C*(4)H), **128.8** (Ar*C*(3,5)H), **129.0** (Ar*C*(2,6)H), **136.5** (Ar*C*(4```)H), **136.7**

(C(5`)), **138.6** (ArC(1)), **140.9** (ArC(1```)), **173.3** (C(1`)ONHBn); HRMS (NSI$^+$) $C_{24}H_{31}N_2O$ [M+H]$^+$: found 363.2433, required 363.2431 (+0.6 ppm).

4-Nitrophenyl (2*R*,2*R*)-3-methyl-2-(pyrrolidin-1-yl)-3-(*p*-tolyl)pent-4-enoate *syn*-4k and 4-Nitrophenyl (2*R*,2*S*)-3-methyl-2-(pyrrolidin-1-yl)-3-(*p*-tolyl)pent-4-enoate *anti*-4k

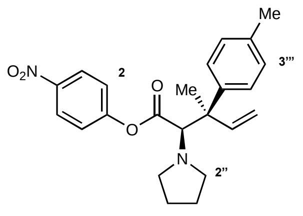

syn-**4k**

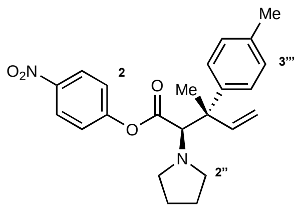

anti-**4k**

Selected data for the [2,3]-rearrangement product (NMR yield = 14%, 4:1 dr):

Major diastereoisomer syn-**4k**: ^{1}H NMR (400 MHz, $CDCl_3$) δ_H: **1.54 – 1.60** (4H, s, C(3``,4``)H_2), **1.61** (3H, s, C(3`)CH_3), **2.24 – 2.52** (4H, m, C(2``,5``)H_2), **2.30** (3H, s, ArC(4```)CH_3) **3.44** (1H, s, C(2`)*H*), **4.83** (1H, dd, $^3J_{HH(trans)}$ = 17.3, $^2J_{HH}$ = 1.1, C(5`)H_AH$_B$), **4.97** (1H, dd, $^3J_{HH(cis)}$ = 10.7, $^2J_{HH}$ = 1.0, C(5`)H$_A$$H_B$, **6.42** (1H, dd, $^3J_{HH}$ = 17.3, $^2J_{HH}$ = 10.7, C(4`)*H*), **6.88 – 6.93** (2H, m, ArC(2,6)*H*), **7.27 – 7.36** (4H, m, C(3`)ArC*H*), **8.11 – 8.16** (2H, m, ArC(3,5)*H*).

Minor diastereoisomer anti-**4k**: ^{1}H NMR (400 MHz, $CDCl_3$) δ_H: **5.19** (1H, dd, $^3J_{HH(trans)}$ = 17.5, $^2J_{HH}$ = 1.0, C(5`)$H_A$H$_B$), **5.41** (1H, dd, $^3J_{HH(cis)}$ = 10.9, $^2J_{HH}$ = 1.0, C(5`)H$_A$$H_B$), **6.51** (1H, dd, $^3J_{HH}$ = 17.5, $^2J_{HH}$ = 10.9, C(4`)*H*).

(*R*, *E*)-*N*-Benzyl-5-(4-(tert-butyl)phenyl)-2-(pyrrolidin-1-yl)hex-4-enamide 3l

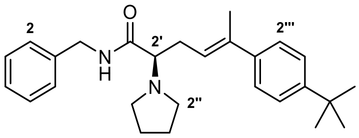

Following General Procedure **F**, (*S*)-tetramisole hydrochloride (9.3 mg, 38.7 μmol), triethylamine (43 μL, 0.309 mmol), (*E*)-1-(3-(4-(tert-butyl)phenyl)but-2-en-1-yl)-1-(2-(4-nitrophenoxy)-2-oxoethyl)pyrrolidin-1-ium bromide **2l** (100 mg, 0.193 mmol) and then benzylamine (32 μL, 0.290 mmol) in MeCN gave the crude product, which was purified by silica flash chromatography (CH_2Cl_2 : Et_2O 95:5 to 70:30) to give the title compound as an off-white solid (48 mg, 61%);

mp 94 – 96 °C ($CHCl_3$); $[\alpha]_D^{20}$ = −20.9 (*c* 1.1, $CHCl_3$); Chiral HPLC analysis, Chiralcel OJ-H (98:2 hexane : IPA, flow rate 2 mLmin^{-1}, 254 nm, 40 °C) t_R (*R*): 11.7 min, t_R (*S*): 15.9 min, 91:9 er; ν_{max} (film): 3287, 2961, 2870, 2803, 1651, 1508, 1454, 1362, 1269, 1244, 1138, 1115; ^{1}H-NMR (500 MHz, $CDCl_3$): δ_H **1.33** (9H, s, ArC(CH_3)$_3$, **1.71 – 1.79** (4H, m, C(3``,4``)H_2), **2.01** (3H, s, C(6`)H_3), **2.63 – 2.69** (5H, m, C(2``,5``)*H* and C(3`)$H_A$H$_B$), **2.76** (1H, ddd, $^2J_{HH}$ = 15.6, $^3J_{HH}$ = 7.8, 4.4, C(3`)H$_A$$H_B$), **3.04** (1H, dd, $^3J_{HH}$ = 6.8, 4.4, C(2`)*H*), **4.40** (1H, dd, $^2J_{HH}$ = 14.7, $^3J_{HH}$ = 5.5, NHC$H_A$H$_B$), **4.52** (1H, dd, $^2J_{HH}$ = 14.7, $^3J_{HH}$ = 6.5, NHCH$_A$$H_B$), **5.84** (1H, t, $^3J_{HH}$ = 7.8, C(4`)*H*), **7.10** (1H, s, N*H*), **7.20 – 7.25** (5H, m, ArC*H*), **7.26 – 7.34** (4H, m, C(2```,3```,5```,6```)*H*); ^{13}C{^{1}H}-NMR (126 MHz, $CDCl_3$) δ_C: **16.1** (C(6`)H$_3$), **23.5** (C(3``,4``)H$_2$), **31.3** (C(3`)H$_2$), **31.4** (ArC(CH$_3$)$_3$), **34.6** (NHCH$_2$Ph), **43.3** (ArC(CH$_3$)$_3$), **52.0** (C(2``,5``)H$_2$), **69.1** (C(2`)H), **122.8** ($C$(4`)H), **125.2** (ArC(3```,5```)H), **125.5** (ArC(2```,6```)H), **127.4** (ArC(4)H), **127.9** (ArC(2,6)H), **128.8** (ArC(3,5)H), **136.6** (C(5`)), **138.6** (ArC(1)), **140.8** (ArC(1```)H), **149.8** (ArC(4```)CH(CH$_3$)$_3$), **173.3** (C(1`)ONHBn); HRMS (NSI$^+$) $C_{27}H_{36}N_2O$ [M+H]$^+$: found 405.2898, required 405.2900 (−0.5 ppm).

4-Nitrophenyl (2*R*,3*R*)-3-(4-(*tert*-butyl)phenyl)-3-methyl-2-(pyrrolidin-1-yl)pent-4-enoate *syn*-4l and 4-Nitrophenyl (2*R*,3*S*)-3-(4-(*tert*-butyl)phenyl)-3-methyl-2-(pyrrolidin-1-yl)pent-4-enoate *anti*-4l

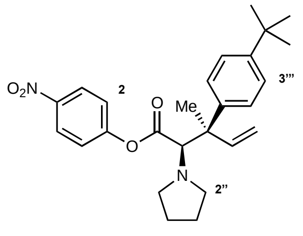

syn-**4l**

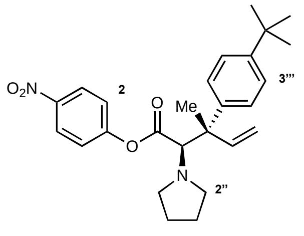

anti-**4l**

Selected data for the [2,3]-rearrangement product (NMR yield = 10%, 17:1 dr):

Major diastereoisomer syn-**4l**: ^{1}H NMR (400 MHz, $CDCl_3$) δ_H: **1.32** (9H, s, ArC(4```)CH(C*H*$_3$)$_3$), **1.68** (3H, s, C(3`)C*H*$_3$), 1.74 – 1.79 (4H, m, C(3``,4``)*H*$_2$), **2.75 – 2.86** (4H, m, C(2``,5``)*H*$_2$), **4.06** (1H, s, C(2`)*H*), **5.27** (1H, dd, $^3J_{HH(trans)}$ = 17.6, $^2J_{HH}$ = 1.3, C(5`)*H*$_A$H$_B$), **5.31** (1H, dd, $^3J_{HH(cis)}$ = 10.9, $^2J_{HH}$ = 1.3, C(5`)H$_A$*H*$_B$), **6.72 – 6.75** (2H, m, ArC(2,6)*H*), **6.79** (1H, dd, $^3J_{HH(trans)}$ = 17.6, $^3J_{HH(cis)}$ =10.9, C(4`)*H*), **7.34 – 7.37** (4H, m, C(3`)ArC*H*), **8.11 – 8.15** (2H, m, ArC(3,5)*H*);

Minor diastereoisomer anti-**4l**: ^{1}H NMR (400 MHz, $CDCl_3$) δ_H: **3.97** (1H, s, C(2`)*H*), **5.10** (1H, d, $^3J_{HH(trans)}$ = 17.6, C(5`)*H*$_A$H$_B$), **5.17** (1H, d, $^3J_{HH}$ = 10.9, C(5`)H$_A$*H*$_B$), **6.49** (1H, dd, $^3J_{HH(trans)}$ = 17.5, $^3J_{HH(cis)}$ =10.9, C(4`)*H*), **6.56 – 6.58** (2H, m, ArC(2,6)*H*), **7.49 – 7.54** (4H, m, C(3`)ArC*H*), **7.90 – 7.93** (2H, m, ArC(3,5)*H*).

(*R*,*E*)-*N*-Benzyl-5-(4-nitrophenyl)-2-(pyrrolidin-1-yl)hex-4-enamide 3m

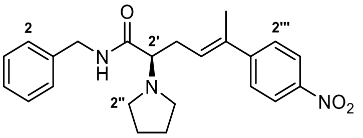

Following General Procedure **F**, (*S*)-tetramisole hydrochloride (11.1 mg, 46.2 μmol), triethylamine (52 μL, 0.370 mmol), (*E*)-1-(2-(4-nitrophenoxy)-2-oxoethyl)-1-(3-phenylbut-2-en-1-yl)pyrrolidin-1-ium bromide **2m** (117 mg, 0.231 mmol), benzylamine (38 μL, 0.347 mmol) in MeCN gave the crude product, which was purified by silica flash chromatography (CH_2Cl_2 : Et_2O 95:5 to 50:50) to give the title compound as an orange solid (43 mg, 47%);

mp 131-134 °C ($CHCl_3$); $[\alpha]_D^{20} = -43.2$ (*c* 1.6, $CHCl_3$); Chiral HPLC analysis, Chiralcel OJ-H (97.5:2.5 hexane : IPA, flow rate 2 mLmin^{-1}, 254 nm, 40 °C) t_R (*R*): 41.9 min, t_R (*S*): 46.8 min, 84:16 er; ν_{max} (film): 3296, 2959, 2924, 2853, 2361, 2340, 1653, 1516, 1497, 1454, 1344, 1111, 1080, 1007; ^{1}H NMR (500 MHz, $CDCl_3$) δ_H: **1.74 – 1.80** (4H, m, C(3``,4``)*H*$_2$), **2.03** (3H, s, C*H*$_3$), **2.58 – 2.68** (5H, m, C(2``,5``)*H*$_2$ and C(3`)*H*$_A$H$_B$), **2.77** (1H, ddd, $^2J_{HH}$ = 15.5, $^3J_{HH}$ = 7.1, 4.5, C(3`)H$_A$*H*$_B$), **3.07** (1H, dd, $^3J_{HH}$ = 7.1, 4.4, C(2`)*H*), **4.35** (1H, dd, $^2J_{HH}$ = 14.7, $^3J_{HH}$ = 5.5, NC*H*$_A$H$_B$), **4.56** (1H, dd, $^2J_{HH}$ = 14.7, $^3J_{HH}$ = 6.6, NHCH$_A$*H*$_B$), **6.00** (1H, t, $^3J_{HH}$ = 7.1, C(4`)*H*), **7.17** (1H, s, N*H*), **7.22 – 7.25** (5H, m, ArC*H*), **7.40** (2H, d, $^3J_{HH}$ = 8.9, ArC(2```,6```)*H*), **8.12** (2H, d, $^3J_{HH}$ = 8.9, ArC(3```,5```)*H*); ^{13}C{^{1}H}-NMR (126 MHz, $CDCl_3$) δ_C: **15.9** (*C*H$_3$), **23.5** (*C*(3``,4``)H$_2$), **31.3** (*C*(3`)H$_2$), **43.3** (N*C*H$_2$Ph), **51.9** (*C*(2``,5``)H$_2$), **68.6** (*C*(2`)H), **123.7** (Ar*C*(2```,6```)H), **126.4** (Ar*C*(3```,5```)H), **127.6** (*C*(4`)H), **127.9** (Ar*C*(4)H), **128.0** (Ar*C*(2,6)H), **128.8** (Ar*C*(3,5)H), **135.2** (Ar*C*(1)), **138.6** (*C*(5`)), **146.6** (Ar*C*(1```)), **150.1** (Ar*C*(4```)NO$_2$), **172.9** (*C*(1`)ONHBn); HRMS (NSI$^+$) $C_{23}H_{27}N_3O_3$ [M+H]$^+$: found 394.2119, required 394.2125 (−1.5 ppm).

4-Nitrophenyl (2*R*,3*R*)-3-methyl-3-(4-nitrophenyl)-2-(pyrrolidin-1-yl)pent-4-enoate *syn*-4m and 4-nitrophenyl (2*R*,3*S*)-3-methyl-3-(4-nitrophenyl)-2-(pyrrolidin-1-yl)pent-4-enoate *anti*-4m

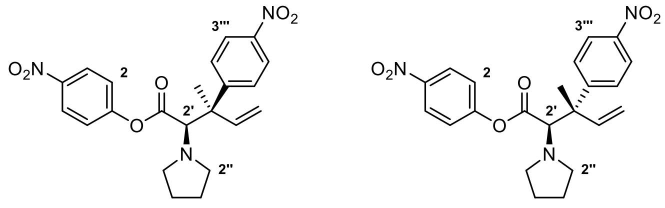

syn-**XX** *anti*-**XX**

Selected data for the [2,3]-rearrangement product (NMR yield = 9%, 5:1 dr):

Major diastereoisomer syn-**4m**: ^{1}H-NMR (500 MHz, $CDCl_3$) δ_H: **1.70** (3H, s, CH_3), **1.72 – 1.77** (4H, m, C(3``,4``)H_2), **2.68 – 2.89** (4H, m, C(2``,5``)H_2), **4.17** (1H, s, C(2`)*H*), **5.28** (1H, d, $^3J_{HH(trans)}$ = 17.5, C(5`) H_AH$_B$), **5.38** (1H, d, $^3J_{HH(cis)}$ = 10.9, C(5`) H$_A$$H_B$), **6.58** (1H, dd, $^3J_{HH}$ = 17.5, 10.9, C(4`)*H*), **7.06** (2H, dd, $^3J_{HH}$ = 9.1, $^4J_{HH}$ = 2.4, ArC(2,6)*H*), **7.64** (2H, dd, $^3J_{HH}$ = 8.9, $^4J_{HH}$ = 2.7, C(2```,6```)*H*), **8.18** (2H, dd, $^3J_{HH}$ = 8.9, $^4J_{HH}$ = 2.7, C(3```,5```)*H*), **8.20 – 8.24** (2 H, dd, $^3J_{HH}$ = 9.1, $^4J_{HH}$ = 2.4, ArC(3,5)*H*); ^{13}C{^{1}H}-NMR (126 MHz, $CDCl_3$) δ_C: **22.5** (CH_3), **24.2** (*C*3``,4``), **48.7** (*C*3`), **52.4** (*C*2``,5``), **72.8** (*C*(2`)H), **116.4** (*C*(5`)H_2), **122.6** (Ar*C*(2,6)H), **123.5** (Ar*C*(3```,5```)H), **125.4** (Ar*C*(3,5)H), **127.9** (ArC(2```,6```)), **141.7** (Ar*C*(4)NO_2), **145.6** (Ar*C*(4```)NO_2), **146.6** (*C*(4`)H), **153.9** (Ar*C*(1```)H), **154.8** (Ar*C*(1)H), **168.1** (*C*(1`)O_2Ar).

Minor diastereoisomer anti-**4m**: ^{1}H-NMR (500 MHz, $CDCl_3$) δ_H: **4.11** (1H, s, C(2`)*H*), **5.10** (1H, d, $^3J_{HH(trans)}$ = 17.6, C(5`) H_AH$_B$), **5.34** (1H, d, $^3J_{HH(cis)}$ = 10.9, C(5`) H$_A$$H_B$), **6.54** (1H, dd, $^3J_{HH}$ = 17.6, 10.9, C(4`)*H*).

(*R*,*E*)-*N*-benzyl-5-(4-fluorophenyl)-2-(pyrrolidin-1-yl)hex-4-enamide 3n

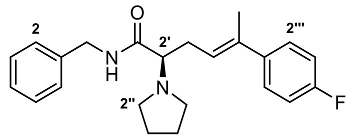

Following General Procedure **F**, (*S*)-tetramisole hydrochloride (9.8 mg, 40.9 μmol), triethylamine (46 μL, 0.327 mmol), (*E*)-1-(2-(4-fluorophenoxy)-2-oxoethyl)-1-(3-phenylbut-2-en-1-yl)pyrrolidin-1-ium bromide **2n** (98 mg, 0.204 mmol), benzylamine (34 μL, 0.307 mmol) in MeCN gave the crude product, which was purified by silica flash chromatography (CH_2Cl_2 : Et_2O 95:5 to 70:30) to give the title compound as an off-white solid (41 mg, 55%);

mp. 111-113 °C ($CHCl_3$); $[\alpha]_D^{20}$ = −28.1 (*c* 1.1, $CHCl_3$); Chiral HPLC analysis, Chiralcel OJ-H (99:1 hexane : IPA, flow rate 2 mLmin^{-1}, 211 nm, 40 °C) t_R (*R*): 31.8 min, t_R (*S*): 39.9 min, 87:13 er; ν_{max} (film): 3289, 3063, 2967, 2803, 1651, 1601, 1508, 1454, 1435, 1360, 1229, 1161, 1138, 1030, 1013; ^{1}H-NMR (500 MHz, $CDCl_3$) δ_H: **1.73 – 1.78** (4H, m, C(3``,4``)H_2), **1.98** (3H, d, $^4J_{HH}$ = 1.3 , CH_3), **2.57 – 2.67** (5H, m, C(2``,5``)H_2 and C(3`)$H_A$H$_B$), **2.74** (1H, ddd, $^2J_{HH}$ = 15.4, $^3J_{HH}$ = 7.6, 4.4, C(3`)H$_A$$H_B$), **3.02** (1H, dd, $^3J_{HH}$ = 6.6, 4.4, C(2`)*H*), **4.37** (1H, dd, $^2J_{HH}$ = 14.7, $^3J_{HH}$ = 5.5, NHC$H_A$H$_B$), **4.54** (1H, dd, $^2J_{HH}$ = 14.7, $^3J_{HH}$ = 6.6, NHCH$_A$$H_B$), **5.77** (1H, t, $^3J_{HH}$ = 7.2, C(4`)*H*), **6.93 – 6.99** (2H, m, C(2```,6```)*H*), **7.10** (1H, s, N*H*), **7.22 – 7.24** (5H, m, ArC*H*), **7.24 – 7.27** (2H, m, C(3```,5```)*H*); ^{13}C{^{1}H}-NMR (126 MHz, $CDCl_3$) δ_C: **16.3** (CH_3), **23.5** (*C*(3``,4``)H_2), **31.3** (*C*(3`)H_2), **43.3** (NCH_2Ph), **52.0** (*C*(2``,5``)), **69.1** (*C*(2`)), **115.0** (Ar*C*(3```,5```)H, d, $^3J_{CF}$ = 21.2)), **123.5** (*C*(4`)H), **127.4** (Ar*C*(2```,6```)H, d, $^4J_{CF}$ = 7.9), **127.5** (Ar*C*(4)H), **127.9** (Ar*C*(3,5)H), **128.8** (Ar*C*(2,6)H), **135.8** (*C*(5`)), **138.6** (Ar*C*(1)H), **139.8** (Ar*C*(1```)), **150.1** (Ar*C*(4```)F, d, $^2J_{CF}$ = 245.2), **172.9** (*C*(1`)ONHBn); ^{19}F-NMR (377 MHz, $CDCl_3$)

δ_F: −116.4 – −116.5 (m, ArC(4```)*F*); HRMS (NSI$^+$) $C_{23}H_{29}FN_2O$ [M+H]$^+$: found 367.2182, required 367.2180 (+0.5 ppm).

4-Nitrophenyl (2*R*,3*R*)-3-(4-fluorophenyl)-3-methyl-2-(pyrrolidin-1-yl)pent-4-enoate *syn*-4n and 4-nitrophenyl (2*R*,3*S*)-3-(4-fluorophenyl)-3-methyl-2-(pyrrolidin-1-yl)pent-4-enoate *anti*-4n

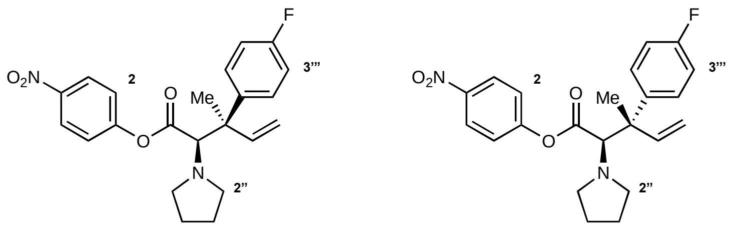

syn-**4n** *anti*-**4n**

Selected data for the [2,3]-rearrangement product (NMR yield = 11%, 4.5:1 dr):

Major diastereoisomer syn-**4n**: ^{1}H NMR (400 MHz, $CDCl_3$) δ_H: **1.66** (3H, s, C*H*$_3$), **1.71 – 1.76** (4H, m, C(3``,4``)*H*$_2$), **2.68 – 2.85** (4H, m, C(2``,5``)*H*$_2$), **4.06** (1H, s, C(2`)*H*), **5.24** (1H, dd, $^3J_{HH(trans)}$ = 17.5, $^2J_{HH}$ = 1.1, C(5`)*H*$_A$H$_B$), **5.31** (1H, d, $^3J_{HH(cis)}$ = 10.9, $^2J_{HH}$ = 1.1, C(5`)H$_A$*H*$_B$), **6.66** (1H, dd, $^3J_{HH(trans)}$ = 17.5, $^3J_{HH(cis)}$ = 10.9, C(4`)*H*), **6.93 – 6.97** (2H, m, ArC(2,6)*H*), **6.98 – 7.06** (2H, m, ArC(3```,5```)*H*), **7.38 – 7.45** (2H, m, ArC(2```,6```)*H*), **8.19 – 8.22** (2H, m, ArC(3,5)*H*); 13{1H}-NMR (101 MHz, $CDCl_3$) δ_C: **22.9** (*C*H$_3$), **24.0** (*C*(3``,4``)H$_2$), **47.6** (*C*(3`)), **52.3** (*C*(2``,5``)H$_2$), **73.8** (*C*(2`)H), **115.2** (Ar*C*(3``,5```)H), **122.6** (Ar*C*(2,6)H), **125.3** (Ar*C*(3,5)H), **128.5** (Ar*C*(2```,6```)H, d, $^3J_{CF}$ = 7.6), **141.9** (Ar*C*(1```), d, $^4J_{CF}$ = 3.3), **142.5** (*C*(4`)H), **145.5** (Ar*C*(1)O), **155.0** (Ar*C*(4```)F, d, $^1J_{CF}$ = 245.8), **168.5** (*C*(1`)O$_2$Ar); ^{19}F-NMR (377MHz, $CDCl_3$) δ_F: −116.3 – −116.4 (m, ArC(4````)*F*).

Minor diastereoisomer anti-**4n**: ^{1}H NMR (400 MHz, $CDCl_3$) δ_H: **1.69** (3H, s, C*H*$_3$), **1.72 – 1.78** (4H, m, C(3``,4``)*H*$_2$), **2.70 – 2.83** (4H, m, C(2``,5``)*H*$_2$), **4.00** (1H, s, C(2`)*H*), **5.07** (1H, dd, $^3J_{HH(trans)}$ = 17.6, $^2J_{HH}$ = 1.0, C(5`)*H*$_A$H$_B$), **5.22** (1H, dd, $^3J_{HH(cis)}$ = 10.8, $^2J_{HH}$ = 1.0, C(5`)H$_A$*H*$_B$), **6.50** (1H, dd, $^3J_{HH(trans)}$ 17.6, $^3J_{HH(cis)}$ = 10.8, C(4`)*H*), **6.85 – 6.89** (2H, m, ArC(2,6)*H*), **6.99 – 7.06** (2H, m, ArC(3```,5```)*H*), **7.40 – 7.46** (2H, m, ArC(2```,6```)*H*), **8.15 – 8.19** (2H, m, ArC(3,5)*H*); ^{19}F-NMR (377MHz, $CDCl_3$) δ_F: −116.1 – −116.2 (m, ArC(4````)*F*).

(*R*,*E*)-*N*-Benzyl-5-(4-bromophenyl)-2-(pyrrolidin-1-yl)hex-4-enamide 3o

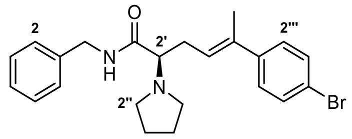

Following General Procedure **F**, (*S*)-tetramisole hydrochloride (9.6 mg, 40.0 µmol), triethylamine (44 µL, 0.320 mmol), (*E*)-1-(2-(4-bromophenoxy)-2-oxoethyl)-1-(3-phenylbut-2-en-1-yl)pyrrolidin-1-ium bromide **2o** (56 mg, 0.104 mmol), then benzylamine (17 µL, 0.155 mmol) in MeCN gave the crude product, which was purified by silica flash chromatography (CH_2Cl_2 : Et_2O 95:5 to 70:30) to give the title compound as an off-white solid (22 mg, 49%);

mp 138-140 °C ($CHCl_3$); $[\alpha]_D^{20}$ = −63.0 (*c* 1, $CHCl_3$); Chiral HPLC analysis, Chiralcel OJ-H (98.5:1.5 hexane : IPA, flow rate 2 mLmin^{-1}, 254 nm, 40 °C) t_R (*R*): 27.4 min, t_R (*S*): 35.1 min, 88:12 er; ν_{max} (film): 3283, 3063, 3030, 2965, 2930, 2801, 1651, 1520, 1489, 1454, 1433, 1400, 1360, 1244, 1138, 1076, 1030, 1007; ^{1}H-NMR (500 MHz, $CDCl_3$) δ_H: **1.73 – 1.78** (4H, m, C(3``,4``)*H*$_2$), **1.97** (3H, d, $^4J_{HH}$ = 1.1 , C*H*$_3$), **2.56 – 2.68** (5H, m, C(2``,5``)*H*$_2$ and C(3`)*H*$_A$H$_B$), **2.73** (1H, ddd, $^2J_{HH}$ = 15.4, $^3J_{HH}$ = 7.6, 4.4, C(3`)H$_A$*H*$_B$), **3.04** (1H, t, $^3J_{HH}$ = 5.8, C(2`)*H*), **4.36** (1H, dd, $^2J_{HH}$ = 14.7, $^3J_{HH}$ = 5.5, NC*H*$_A$H$_B$), **4.53**

(1H, dd, $^2J_{HH}$ = 14.7, $^3J_{HH}$ = 6.4, NCH$_A$*H*$_B$), **5.82** (1H, t, $^3J_{HH}$ = 7.9, C(4`)*H*), **7.10 – 7.16** (1H, m, N*H*), **7.15** (2H, d, $^3J_{HH}$ = 8.6 , ArC(2```,6```)*H*), **7.21 – 7.25** (5H, m, ArC*H*), **7.39** (2H, d, $^3J_{HH}$ = 8.7 , ArC(3``,5``)*H*); ^{13}C{^{1}H}-NMR (126 MHz, CDCl$_3$) δ$_C$: **16.0** (*C*H$_3$), **23.5** (*C*(3``,4``)H$_2$), **31.3** (*C*(3`)H), **43.3** (NH*C*H$_2$Ar), **51.9** (*C*(2``,5``)H$_2$), **68.9** (*C*(2`)H), **120.7** (*C*(4`)H), **124.2** (Ar*C*(4```)Br), **127.5** (Ar*C*(2,6)H), **127.5** (Ar*C*(4)H), **127.9** (Ar*C*(2```,6```)H), **128.8** (Ar*C*(3,5)H), **131.3** (Ar*C*(3```,5```)H), **135.8** (*C*(5`)), **138.5** (Ar*C*(1)), **142.6** (Ar*C*(1```)), **173.1** (*C*(1`)ONHBn); HRMS: (NSI$^+$) $C_{23}H_{27}BrN_2O$ $[M+H]^+$: found 429.1355, required 429.1359 (−0.9 ppm).

4-Nitrophenyl (2*R*,3*R*)-3-(4-bromophenyl)-3-methyl-2-(pyrrolidin-1-yl)pent-4-enoate *syn*-4o and 4-Nitrophenyl (2*R*,3*S*)-3-(4-bromophenyl)-3-methyl-2-(pyrrolidin-1-yl)pent-4-enoate *anti*-4o

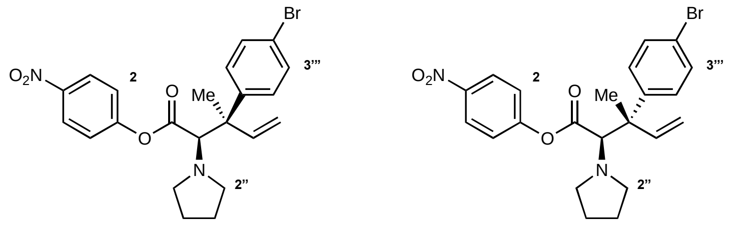

syn-**4o** *anti*-**4o**

Selected data for the [2,3]-rearrangement product (NMR yield = 7%, 6:1 dr):

Major diastereoisomer syn-**4o**: ^{1}H NMR (400 MHz, CDCl$_3$) δ$_H$: **1.66** (3H, s, C*H*$_3$), **1.71 – 1.77** (4H, m, C(3``,4``)*H*$_2$), **2.67 – 2.85** (4H, m, C(3``,4``)*H*$_2$), **4.06** (1H, s, C(2`)*H*), **5.24** (1H, dd, $^3J_{HH(trans)}$ = 17.5, $^2J_{HH}$ = 1.1, C(5`)*H*$_A$H$_B$), **5.31** (1H, dd, $^3J_{HH(cis)}$ = 10.9, $^2J_{HH}$ = 1.1, C(5`)H$_A$*H*$_B$), **6.62** (1H, dd, $^3J_{HH}$ = 17.5, 10.9, C(4`)*H*), **6.94 – 6.99** (2H, m, ArC(2,6)*H*), **7.30 – 7.36** (2H, m, ArC(2```,6```)*H*), **7.42 – 7.47** (2H, m, ArC(3```,5```)*H*), **8.18 – 8.24** (2H, m, ArC(3,5)*H*).

Minor diastereoisomer anti-**4o**: ^{1}H NMR (400 MHz, CDCl$_3$) δ$_H$: **3.99** (1H, s, C(2`)*H*), **5.07** (1H, d, $^3J_{HH(trans)}$ = 17.5, C(5`)*H*$_A$H$_B$), **5.24** (1H, d, $^3J_{HH(cis)}$ = 10.8, C(5`)H$_A$*H*$_B$), **6.49** (1H, dd, $^3J_{HH(trans)}$ 17.5, $^3J_{HH(cis)}$ = 10.8, C(4`)*H*).

(*R*,*E*)-*N*-Benzyl-5-(4-iodophenyl)-2-(pyrrolidin-1-yl)hex-4-enamide 3p

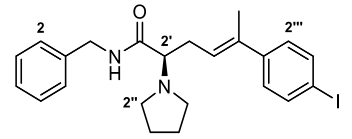

Following General Procedure **F**, (*S*)-tetramisole hydrochloride (9.6 mg, 40.0 µmol), triethylamine (44 µL, 320 µmol), (*E*)-1-(2-(4-iodophenoxy)-2-oxoethyl)-1-(3-phenylbut-2-en-1-yl)pyrrolidin-1-ium bromide **2p** (117.5 mg, 200 µmol), benzylamine (33 µL, 300 µmol) in MeCN gave the crude product, which was purified by silica flash chromatography (CH_2Cl_2 : Et_2O 95:5 to 70:30) to give the title compound as an off-white solid (59 mg, 62%);

mp 115-117 °C (CHCl$_3$); $[\alpha]_D^{20}$ = –27.3° (*c* 1, CHCl$_3$); Chiral HPLC analysis, Chiralcel OJ-H (98.5:1.5 hexane : IPA, flow rate 2 mLmin^{-1}, 254 nm, 40 °C) t$_R$ (*R*): 27.4 min, t$_R$ (*S*): 35.1 min, 91:9 er; ν_{max} (film): 3287, 2965, 2930, 2805, 1653, 1518, 1454, 1396, 1360, 1244, 1138, 1070, 1003, 814, 698; ^{1}H-NMR (500 MHz, CDCl$_3$): δ$_H$ **1.73 – 1.78** (4H, m, C(3``,4``)*H*$_2$), **1.97** (3H, d, $^4J_{HH}$ = 1.1, C*H*$_3$), **2.54 – 2.66** (5H, m, C(2``,5``)*H*$_2$ and C(3`)*H*$_A$H$_B$), **2.69 – 2.77** (1H, m, C(3`)H$_A$*H*$_B$), **3.03** (1H, dd, $^3J_{HH}$ = 6.8, 4.4, C(2`)*H*), **4.36** (1H, dd, $^2J_{HH}$ = 14.7, $^3J_{HH}$ = 5.6, NHC*H*$_A$H$_B$), **4.53** (1H, dd, $^2J_{HH}$ = 14.7, $^3J_{HH}$ = 6.4, NHCH$_A$*H*$_B$), **5.82** (1H, t, $^3J_{HH}$ = 7.9, C(4`)*H*), **7.03** (2H, d, $^3J_{HH}$ = 8.4, ArC(3```,5```)*H*), **7.10** (1H, d, $^3J_{HH}$ = 6.3 , N*H*), **7.20 – 7.26** (5H, m, ArC*H*), **7.59** (2H, d, $^3J_{HH}$ = 8.4, ArC(2```,6```)*H*); ^{13}C{^{1}H}-NMR

(126 MHz, $CDCl_3$) δ_C: **16.0** (*C*H_3), **23.5** (C(3``,4``)H_2), **31.3** (*C*(3`)H_2), **43.3** (N*C*H_2Ar), **51.9** (C(2``,5``)H_2), **68.9** (*C*(2`)H), **92.1** (Ar*C*(4```)I), **124.4** (*C*(4`)H), **127.5** (Ar*C*(4)H), **127.8** (Ar*C*(2,6)H), **127.9** (Ar*C*(3,5)H), **128.8** (Ar*C*(2```,6```)H), **135.8** (*C*(5`)), **137.3** (ArC(3```,5```)H), **138.6** (Ar*C*(1)), **143.3** (Ar*C*(1```)), **173.1** (*C*(1`)ONHBn); HRMS (NSI$^+$) $C_{23}H_{27}IN_2O$ [M+H$^+$]: found 475.1230, required 475.1241 (−2.3 ppm).

4-Nitrophenyl (2*R*,3*R*)-3-(4-iodophenyl)-3-methyl-2-(pyrrolidin-1-yl)pent-4-enoate *syn*-4p and 4-Nitrophenyl (2*R*,3*S*)-3-(4-iodophenyl)-3-methyl-2-(pyrrolidin-1-yl)pent-4-enoate *anti*-4p

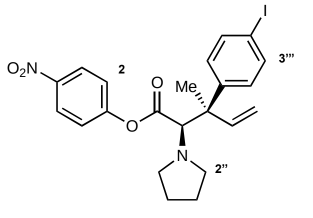

syn-**4p**

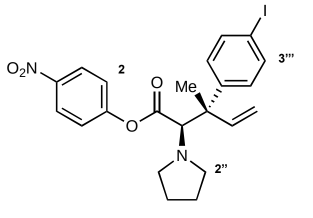

anti-**4p**

Selected data for the [2,3]-rearrangement product (NMR yield = 7%, 7:1 dr):

Major diastereoisomer *syn*-**4p**: ^{1}H NMR (400 MHz, $CDCl_3$) δ_H: **1.64** (3H, s, C*H_3*), **1.71 – 1.76** (4H, m, C(3``,4``)*H*), **2.68 – 2.84** (4H, m, C(2``,5``)*H*), **4.05** (1H, s, C(2`)*H*), **5.23** (1H, dd, $^3J_{HH(trans)}$ = 17.5, $^2J_{HH}$ = 1.0, C(5`)H_AH_B), **5.31** (1H, dd, $^3J_{HH(cis)}$ = 10.9, $^2J_{HH}$ = 1.0, C(5`)$H_AH_B$), **6.61** (1H, dd, $^3J_{HH(trans)}$ = 17.5, $^3J_{HH(cis)}$ = 10.9, C(4`)*H*), **6.93 – 6.98** (2H, m, ArC(2,6)*H*), **7.18 – 7.23** (2H, m, ArC(2```,6```)*H*), **7.62 – 7.68** (2H, m, ArC(3```,5```)*H*), **8.19 – 8.22** (2H, m, ArC(3,5)*H*);

Minor diastereoisomer *anti*-**4p**: ^{1}H NMR (400 MHz, $CDCl_3$) δ_H: **3.98** (1H, s, C(2`)*H*), **5.07** (1H, dd, $^3J_{HH(trans)}$ = 17.5, $^2J_{HH}$ = 0.9, C(5`)H_AH_B), **5.24** (1H, dd, $^3J_{HH(cis)}$ = 11.0, $^2J_{HH}$ = 0.9, C(5`)$H_AH_B$), **6.48** (1H, dd, $^3J_{HH(trans)}$ = 17.5, $^3J_{HH(cis)}$ = 11.0, C(4`)*H*).

(*R*,*E*)-*N*-Benzyl-5-(3-bromophenyl)-2-(pyrrolidin-1-yl)hex-4-enamide 3r

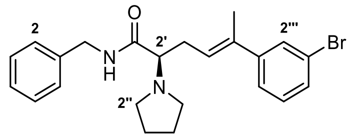

Following General Procedure **F**, (*S*)-tetramisole hydrochloride (9.6 mg, 40.0 μmol), triethylamine (45 μL, 0.320 mmol), (*E*)-1-(3-(3-bromophenyl)but-2-en-1-yl)-1-(2-(4-nitrophenoxy)-2-oxoethyl)pyrrolidin-1-ium bromide **2r** (108 mg, 0.200 mmol), then benzylamine (33 μL, 0.300 mmol) in MeCN gave the crude product, which was purified by silica flash chromatography (CH_2Cl_2 : Et_2O 95:5 to 70:30) to give the title compound as an off-white solid (50 mg, 58%);

mp 98 – 101 °C ($CHCl_3$); $[\alpha]_D^{20}$ = −75.5 (*c* 1, $CHCl_3$); Chiral HPLC analysis, Chiralcel AS-H (98.8:1.2 hexane : IPA, flow rate 1 mLmin^{-1}, 211 nm, 40 °C) t_R (*R*): 28.8 min, t_R (*S*): 32.5 min, 88:12 er; ν_{max} (film): 3285, 2965, 2928, 2805, 1651, 1557, 1518, 1454, 1360, 1327, 1246, 1138, 1090, 1028; ^{1}H-NMR (500 MHz, $CDCl_3$): δ_H **1.73 – 1.79** (4H, m, C(3``,5``)*H_2*), **1.97** (3H, d, $^4J_{HH}$ = 1.1, C(6`)*H_3*), **2.56 – 2.67** (5H, m, C(2``,5``)*H_2* and C(3`)$H_AH_B$), **2.74** (1H, ddd, $^2J_{HH}$ = 15.6, $^3J_{HH}$ = 7.8, 4.4, C(3`)H_AH_B), **3.03** (1H, dd, $^3J_{HH}$ = 6.7, 4.4, C(2`)*H*), **4.37** (1H, dd, $^2J_{HH}$ = 14.7, $^3J_{HH}$ = 5.6, NHC$H_AH_B$), **4.54** (1H, dd, $^2J_{HH}$ = 14.7, $^3J_{HH}$ = 6.7, NHCH$_AH_B$), **5.83** (1H, t, $^3J_{HH}$ = 7.9, C(4`)*H*), **7.11** (1H, t, $^3J_{HH}$ = 6.1, N*H*), **7.14** (1H, t, $^3J_{HH}$ = 7.8, C(5```)*H*), **7.19 – 7.21** (1H, m, ArC(6```)*H*), **7.22 – 7.25** (5H, m, ArC*H*), **7.35** (1H, ddd, $^3J_{HH}$ = 7.8, $^4J_{HH}$ = 2.0, 1.2, ArC(4```)*H*), **7.43** (1H, t, $^3J_{HH}$ = 1.8, ArC(2```)*H*); ^{13}C{^{1}H}-NMR (126 MHz, $CDCl_3$) δ_C: **16.1** (*C*H_3), **23.5** (*C*(3``,4``)H_2), **31.3** (*C*(3`)H_2), **43.3** (NH*C*H_2Ph), **51.9** (*C*(2``,5``)H_2), **68.9**

(*C*(2`)H), **122.5** (Ar*C*(4`)H), **124.5** (Ar*C*(3```)Br), **124.8** (Ar*C*(6```)H), **127.5** (Ar*C*(4)H), **127.9** (Ar*C*(2,6)H), **128.8** (Ar*C*(3,5)H), **128.9** (Ar*C*(2```)H), **129.7** (Ar*C*(4```)H), **129.8** (Ar*C*(5```)H), **135.7** (*C*(5`)), **138.5** (Ar*C*(1)), **145.9** (Ar*C*(1```)), **173.1** (*C*(1`)ONHBn); HRMS: (NSI$^+$) $C_{23}H_{38}BrN_2O$ [M+H]$^+$: found 427.1377, required 427.1380 (−0.7 ppm);

4-Nitrophenyl (2*R*,3*R*)-3-(3-bromophenyl)-3-methyl-2-(pyrrolidin-1-yl)pent-4-enoate *syn*-4r and 4-nitrophenyl (2*R*,3*R*)-3-(3-bromophenyl)-3-methyl-2-(pyrrolidin-1-yl)pent-4-enoate *anti*-4r

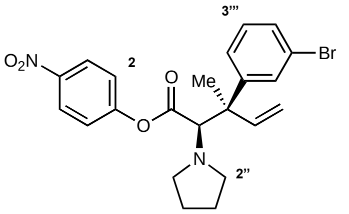

syn-**4r**

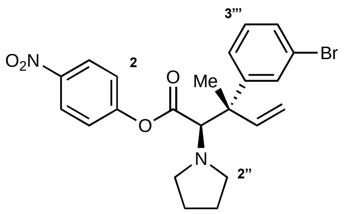

anti-**4r**

Selected data for the [2,3]-rearrangement product (NMR yield = 14%, 5.6:1 dr):

Major diastereoisomer syn-**4r**: ^{1}H NMR (400 MHz, CDCl$_3$) δ_H: **1.65** (3H, s, C*H*$_3$), **1.72 – 1.77** (4H, m, C(3``,4``)*H*$_2$), **2.70 – 2.87** (4H, m, C(2``,5``)*H*$_2$), **4.07** (1H, s, C(2`)*H*), **5.26** (1H, dd, $^3J_{HH(trans)}$ = 17.5, $^2J_{HH}$ = 1.1, C(5`)*H*$_A$H$_B$), **5.33** (1H, dd, $^3J_{HH(cis)}$ = 10.9, $^2J_{HH}$ = 1.1, C(5`)H$_A$*H*$_B$), **6.63** (1H, dd, $^3J_{HH}$ = 17.5, $^3J_{HH}$ = 10.9, C(4`)*H*), **6.96 – 7.00** (2H, m, ArC(2,6)*H*), **7.20** (1H, dd, $^3J_{HH}$ = 8.8, 7.1, ArC(5``)*H*), **7.36 – 7.41** (2H, m, ArC(4```,6```)*H*), **7.57 – 7.60** (1H, m, ArC(2```)*H*), **8.18 – 8.23** (2H, m, ArC(3,5)*H*);

Major diastereoisomer anti-**4r**: ^{1}H NMR (400 MHz, CDCl$_3$) δ_H: **1.67** (3H, s, C*H*$_3$), **4.00** (1H, s, C(2`)*H*), **5.09** (1H, dd, $^3J_{HH(trans)}$ = 17.5, C(5`)*H*$_A$H$_B$, **5.26** (1H, dd, $^3J_{HH(cis)}$ = 10.8, $^2J_{HH}$ = 1.1, C(5`)H$_A$*H*$_B$), **6.48** (1H, dd, $^3J_{HH}$ = 17.5, $^3J_{HH}$ = 10.8, C(4`)*H*), **6.89 – 6.93** (2H, m, ArC(2,6)*H*).

(*R*,*E*)-*N*-benzyl-5-(5-bromo-2-methoxyphenyl)-2-(pyrrolidin-1-yl)hex-4-enamide 3q

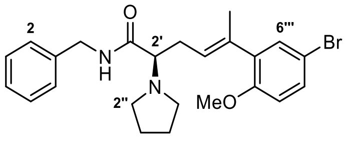

Following General Procedure **F**, (*S*)-tetramisole hydrochloride (9.6 mg, 40.0 μmol), triethylamine (45 μL, 0.320 mmol), (*E*)-1-(3-(5-bromo-2-methoxyphenyl)but-2-en-1-yl)-1-(2-(4-nitrophenoxy)-2-oxoethyl)pyrrolidin-1-ium bromide **2q** (114 mg, 0.200 mmol), benzylamine (33 μL, 0.300 mmol) in MeCN gave the crude product, which was purified by silica flash chromatography (CH_2Cl_2 : Et_2O 95:5 to 70:30) to give the title compound as an off-white solid (41 mg, 35%);

mp 122 – 124 °C (CHCl$_3$); $[\alpha]_D^{20}$ = −62.7 (*c* 1, CHCl$_3$); Chiral HPLC analysis, Chiralcel AD-H (98:2 hexane : IPA, flow rate 2 mLmin^{-1}, 254 nm, 40 °C) t_R (*S*): 21.7 min, t_R (*R*): 31.0 min, 85:15 er; ν_{max} (film): 3285, 2965, 2928, 2805, 1651, 1589, 1557, 1518, 1246, 1138, 1080; ^{1}H NMR (500 MHz, CDCl$_3$): δ_H **1.73 – 1.78** (4H, m, C(3``,4``)*H*$_2$), **1.92** (3H, s, C(6`)*H*$_3$), **2.56 – 2.67** (5H, m, C(2``,5``)*H*$_2$ and C(3`)*H*$_A$H$_B$), **2.71** (1H, ddd, $^2J_{HH}$ = 15.6, $^3J_{HH}$ = 7.8, 4.4, C(3`)H$_A$*H*$_B$), **3.01** (1H, dd, $^3J_{HH}$ = 6.7, 4.4, C(2`)*H*), **3.74** (3H, s, OC*H*$_3$), **4.44** (1H, dd, $^2J_{HH}$ = 14.8, $^3J_{HH}$ = 5.8, NHC*H*$_A$H$_B$), **4.49** (1H, dd, $^2J_{HH}$ = 14.8, $^3J_{HH}$ = 5.8, NHCH$_A$*H*$_B$), **5.83** (1H, t, $^3J_{HH}$ = 7.8, C(4`)*H*), **6.70** (1H, d, $^3J_{HH}$ = 8.7, ArC(5```)*H*), **7.08** (1H, brs, N*H*), **7.14** (1H, d, $^4J_{HH}$ = 2.6, ArC(2```)*H*), **7.23 – 7.31** (6H, m, 5 × NHCH$_2$ArC*H* and ArC(4```)*H*); ^{13}C{^{1}H}-NMR (126 MHz, CDCl$_3$) δ_C: **17.3** (*C*(6`)H$_3$), **23.5** (*C*(3``,4``)H$_2$), **30.9** (*C*(3`)H$_2$), **43.3** (NH*C*H$_2$Ph), **51.9** (C(2``,5``)H$_2$), **55.7** (O*C*H$_3$), **69.0** (*C*(2`)H), **112.5** (*C*(4`)H), **112.8** (Ar*C*(5``)H),

126.0 (Ar*C*(3```)Br), **127.5** (Ar*C*(1```)), **127.9** (Ar*C*(2,6)H), **128.8** (Ar*C*(3,5)H), **130.5** (Ar*C*(4)H), **132.2** (ArC(2```)H), **136.0** (Ar*C*(4```)H), **136.8** (C(5`)), **138.6** (Ar*C*(1)), **155.9** (Ar*C*(6```)OCH_3), **173.3** (C(1`)); HRMS (NSI^+) $C_{24}H_{30}BrN_2O$ $[M+H]^+$: found 457.1476, required 457.1485 (−2.0 ppm).

4-Nitrophenyl (2*R*,2*R*)-3-(5-bromo-2-methoxyphenyl)-3-methyl-2-(pyrrolidin-1-yl)pent-4-enoate *syn*-4q and 4-nitrophenyl (2*R*,2*S*)-3-(5-bromo-2-methoxyphenyl)-3-methyl-2-(pyrrolidin-1-yl)pent-4-enoate *anti*-4q

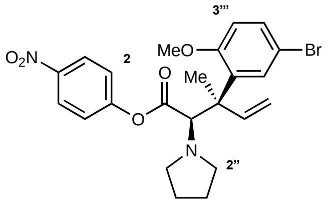

syn-**4q**

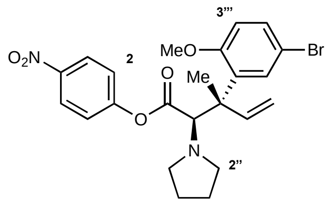

anti-**4q**

Selected data for [2,3]-rearrangement product (NMR yield = 9%, 2.5:1 dr):

Major diastereoisomer syn-**4q**: ^{1}H NMR (400 MHz, $CDCl_3$) δ_H: **1.52 – 1.64** (7H, m, C(3``,4``)H_2 and C(3`)CH_3), **2.45 – 3.01** (4H, m, C(2``,5``)H_2), **3.82** (1H, s, C(2`)*H*), **3.86** (3H, s, OC$H_3$), **5.29** (1H, dd, $^3J_{HH(trans)}$ = 17.6, $^2J_{HH}$ = 1.4, C(5`)H_AH_B), **5.35** (1H, dd, $^3J_{HH(cis)}$ = 10.9, $^2J_{HH}$ = 1.4, C(5`)$H_AH_B$), **6.75** (1H, dd, $^3J_{HH(trans)}$ = 17.6, $^3J_{HH(cis)}$ = 10.9, C(4`)*H*), **6.90 – 6.97** (2H, m, ArC(2,6)*H*), **7.20 – 7.26** (1H, m, ArC*H*), **7.30 – 7.35** (1H, m, ArC*H*), **7.39 – 7.47** (1H, m, ArC*H*), **8.15 – 8.19** (2H, m, ArC(3,5)*H*).

Minor diastereoisomer anti-**4q**: ^{1}H NMR (400 MHz, $CDCl_3$) δ_H: **1.39 – 1.47** (4H, m, C(3``,4``)H_2), **1.68** (3H, s, C(3`)C$H_3$), **4.91** (1H, dd, $^3J_{HH(trans)}$ = 17.5, $^2J_{HH}$ = 1.2, C(5`)H_AH_B), **5.13** (1H, dd, $^3J_{HH(cis)}$ = 10.8, $^2J_{HH}$ = 1.2, C(5`)$H_AH_B$), **6.45** (1H, dd, $^3J_{HH(trans)}$ = 17.5, $^3J_{HH(cis)}$ = 10.8, C(4`)*H*), **6.83 – 6.87** (2H, m, ArC(2,6)*H*).

Ethyl (*R*,*E*)-5-phenyl-2-(pyrrolidin-1-yl)hex-4-enoate 3s

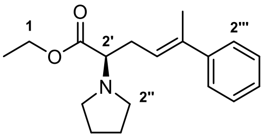

Following General Procedure **F**, (*S*)-tetramisole hydrochloride (9.6 mg, 0.04 mmol), triethylamine (44 µL, 0.32 mmol), (*E*)-1-(2-(4-nitrophenoxy)-2-oxoethyl)-1-(3-phenylbut-2-en-1-yl)pyrrolidin-1-ium bromide **2a** (92.3 mg, 0.2 mmol) in MeCN, followed by sodium ethoxide (41 mg, 0.6 mmol) in ethanol (1 mL) at room temperature for 16 hours gave the crude product, which was purified by silica flash chromatography (CH_2Cl_2 : Et_2O 95:5 to 85:15) to give the title compound as an off-white solid (38 mg, 66%);

mp 81 – 83 °C ($CHCl_3$); $[\alpha]_D^{20}$ = −63.5 (*c* 1, $CHCl_3$); Chiral HPLC analysis, Chiralpak AD-H (99.5:0.5 hexane : IPA, flow rate 2 mLmin^{-1}, 254 nm, 40 °C, t_R (*R*): 7.1 min, t_R (*S*): 7.3 min, 90:10 er; ν_{max} (film): 3057, 3030, 2968, 2932, 2874, 2806, 1727, 1682, 1597, 1495, 1445, 1377, 1292, 1273, 1173, 1157, 1096, 1026; ^{1}H-NMR (400 MHz, $CDCl_3$) δ_H: **1.24** (3H, t, $^3J_{HH}$ = 7.1, C(2)H_3), **1.78 – 1.84** (4H, m, C(3``,4``)H_2), **2.04** (3H, d, $^4J_{HH}$ = 1.4, C(6`)$H_3$), **2.60 – 2.69** (3H, m, C(3`)H_AH_B and C(2``,5``)H_AH_B), **2.70 – 2.80** (3H, m, C(3`)H_AH_B and C(2``,5``)H_AH_B), **3.23** (1H, dd, $^3J_{HH}$ = 9.3, $^4J_{HH}$ = 5.5, C(2`)*H*), **4.17** (2H, q, $^3J_{HH}$ = 7.1, C(1)$H_2$), **5.72** (1H, ddq, $^3J_{HH}$ = 8.0, $^3J_{HH}$ = 6.5, $^4J_{HH}$ = 1.4, C(4`)*H*), **7.22** (1H, tt, $^3J_{HH}$ = 7.0, $^4J_{HH}$ = 1.6, ArC(4```)*H*), **7.27 – 7.32** (2H, m, ArC(3```,5```)*H*), **7.33 – 7.37** (2H, m, ArC(2```,6```)*H*); $^{13}C\{^1H\}$-NMR (101 MHz, $CDCl_3$) δ_C: **14.5** (OCH_2CH_3), **16.2** (*C*(6`)H_3), **23.6**

(*C*(3``,4``)H_2), **31.4** (*C*(3`)H), **51.1** (*C*(2``,5``)H_2), **60.6** (O*C*(1)H_2CH_3), **67.2** (*C*(2`)H), **123.2** (*C*(4`)H), **125.9** (Ar*C*(2```,6```)H), **126.9** (Ar*C*(4```)H), **128.3** (Ar*C*(3```,5```)H), **137.3** (*C*(5`)), **143.8** (Ar*C*(1```)), **172.7** (*C*(1`)O_2Et); HRMS (NSI$^+$) $C_{18}H_{26}NO_2$ [M+H]$^+$: found 288.1958, required 288.1958. (+0.0 ppm).

(*R*,*E*)-5-Phenyl-1,2-di(pyrrolidinyl)hex-4-en-1-one 3t

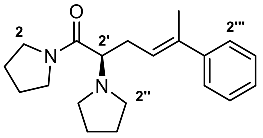

Following General Procedure **F**, (*S*)-tetramisole hydrochloride (9.6 mg, 0.04 mmol), triethylamine (44 μL, 0.32 mmol), (*E*)-1-(2-(4-nitrophenoxy)-2-oxoethyl)-1-(3-phenylbut-2-en-1-yl)pyrrolidin-1-ium bromide **2a** (92.3 mg, 0.2 mmol), pyrrolidine (25 μL, 0.3 mmol) in MeCN gave the crude product, which was purified by silica flash chromatography (CH_2Cl_2 : Et_2O 95:5 to 85:15, then CH_2Cl_2 : MeOH 90:10) to give the title compound as a yellow oil (42 mg, 67%);

$[\alpha]_D^{20}$ = −58.0 (*c* 1, $CHCl_3$); Chiral HPLC analysis, Chiralcel ID (92:8 hexane : IPA, flow rate 1.2 mLmin^{-1}, 254 nm, 40 °C, t_R (*R*): 16.4 min, t_R (*S*): 19.3 min, 91:9 er; ν_{max} (film): 2965, 2872, 2805, 1634, 1495, 1437, 1339, 1190, 1138, 1028; ^{1}H-NMR (400 MHz, $CDCl_3$) δ_H: **1.73 – 1.94** (8H, m, C(3,4)H_2 and C(3``,4``)H_2), **2.07** (3H, d, $^3J_{HH}$ = 1.4, CH_3), **2.59 – 2.84** (6H, m, C(2``,5``)H_2 and C(3')H_AH_B and C(3`)$H_AH_B$), **3.36 – 3.56** (5H, m, C(2,5)$H_2$ and C(2`)*H*), **5.74** (1H, tq, $^3J_{HH}$ = 7.6, $^4J_{HH}$ = 1.4, C(4`)*H*), **7.19 – 7.24** (1H, m, ArC(4```)*H*), **7.27 – 7.34** (4H, m, ArC*H*); ^{13}C{^{1}H}-NMR (126 MHz, $CDCl_3$) δ_C: **16.1** (*C*H_3), **23.5** (*C*(3``,4``)H_2), **24.3** (*C*(3)H_2), **26.4** (*C*(4)H_2), **30.2** (*C*(3`)H_2), **46.0** (*C*(2)H_2), **46.9** (*C*(5)H_2)**,** **50.7** (*C*(2``,5``)H_2), **64.3** (*C*(2`)H), **123.6** (*C*(4`)H), **125.7** (Ar*C*(2```,6```)), **126.9** (Ar*C*(4```)H), **128.3** (Ar*C*(3```,5```)H), **137.4** (*C*(5`)), **143.8** (Ar*C*(1```)), **170.7** (*C*(1`)O); HRMS (NSI$^+$) $C_{20}H_{29}N_2O$ [M+H]$^+$: found 313.2277, required 313.2274 (+1.0 ppm).

(*R*,*E*)-5-Phenyl-2-(pyrrolidin-1-yl)hex-4-en-1-ol 3u

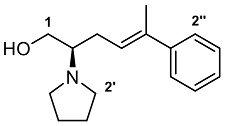

Following a modified General Procedure **F**, (*S*)-tetramisole hydrochloride (9.6 mg, 0.04 mmol), triethylamine (44 μL, 0.32 mmol), (*E*)-1-(2-(4-nitrophenoxy)-2-oxoethyl)-1-(3-phenylbut-2-en-1-yl)pyrrolidin-1-ium bromide **2a** (92.3 mg, 0.2 mmol) were stirred in MeCN at 50 °C under an atmosphere of nitrogen. After 1 hour, the solvent was removed under reduced pressure and the residue dissolved in THF (2 mL). The solution was cooled to 0 °C and $LiAlH_4$ (200 μL, 0.4 mmol, 2 M in THF) was added. After 1 hour, aqueous KOH (1 mL, 2M) was added and the solution extracted with ethyl acetate (3 x 10 mL). The organic layers were combined, dried over anhydrous magnesium sulfate, filtered and concentrated *in vacuo* to give the crude product, which was purified by silica flash chromatography (CH_2Cl_2 : Et_2O 95:5 to 85:15, then CH_2Cl_2 : MeOH 85:15) to give the title compound as a brown oil (26 mg, 53%);

$[\alpha]_D^{20}$ = −38.0 (*c* 1, $CHCl_3$); Chiral HPLC analysis, Chiralcel OJ-H (96:3.7:0.3 hexane : IPA : Et_3N, flow rate 2 mLmin^{-1}, 254 nm, 30 °C, t_R (*R*): 14.7 min, t_R (*S*): 16.8 min, 91:9 er; ^{1}H-NMR (400 MHz, $CDCl_3$) δ_H: **1.76 – 1.84** (4H, m, C(3`,4`)H_2), **2.05** (3H, s, CH_3), **2.29** (1H, dt, $^3J_{HH}$ = 14.4, 8.8, C(3)H_AH_B), **2.43 – 2.51** (1H, m, C(3)H_AH_B), **2.64 – 2.78** (5H, m, C(2`,5`)H_2 and C(2)*H*), **3.44** (1H, dd, $^3J_{HH}$ = 10.6,

6.9, C(1)H_AH_B), **3.66** (1H, dd, $^3J_{HH}$ = 10.6, 4.2, C(1)H_AH_B), **5.75** (1H, tq, $^3J_{HH}$ = 6.8, $^4J_{HH}$ = 1.4, C(4)*H*), **7.20 – 7.25** (1H, m, ArC(4``)*H*), **7.28 – 7.33** (2H, m, ArC(3``,5``)*H*), **7.34 – 7.38** (2H, m, ArC(2``,6``)*H*); $^{13}$C{$^1$H}-NMR (126 MHz, $CDCl_3$) $\delta_C$: **16.2** (*C*$H_3$), **23.6** (*C*(3`,4`)$H_2$), **26.7** (*C*(3)H), **49.7** (C(2`,5`)$H_2$), **61.9** (*C*(1)$H_2$OH), **63.6** (*C*(2)H), **124.6** (*C*(4`)H), **125.8** (Ar*C*(2``,6``)H), **126.9** (Ar*C*(4``)H), **128.4** (Ar*C*(3``,5``)H), **137.0** (Ar*C*(1``)), **143.7** (*C*(5)); HRMS (NSI$^+$) $C_{16}H_{22}NO$ [M+H]$^+$: found 246.1861, required 246.1858 (+1.2 ppm).

(*E*)-5-(3-Bromophenyl)-*N*-(prop-2-yn-1-yl)-2-(pyrrolidin-1-yl)hex-4-enamide 3v

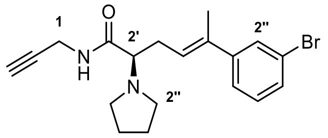

Following General Procedure **F**, (*S*)-tetramisole hydrochloride (89.0 mg, 0.370 mmol), triethylamine (413 µL, 2.96 mmol), (*E*)-1-(3-(3-bromophenyl)but-2-en-1-yl)-1-(2-(4-nitrophenoxy)-2-oxoethyl)pyrrolidin-1-ium bromide **2r** (1.00 g, 1.85 mmol), propargylamine (178 µL, 2.78 mmol) in MeCN gave the crude product, which was purified by silica flash chromatography (CH_2Cl_2 : Et_2O 95:5 to 70:30) to give the title compound as a pale yellow solid (378 mg, 55%);

mp 112−114 °C; Chiral HPLC analysis, Chiralpak AS-H (99.5:0.5 hexane : IPA, flow rate 1 mLmin^{-1}, 254 nm, 40 °C) t_R (*R*): 35.5 min, t_R (*S*): 44.8 min, 88:12 er; ν_{max} (film): 3296, 2965, 2805, 1713, 1661, 1589, 1557, 1514, 1418, 1333, 1242, 1136; ^{1}H-NMR (500 MHz, $CDCl_3$) δ_H: **1.76 – 1.84** (4H, m, C(3``,4``)H_2), **1.99** (3H, d, $^4J_{HH}$ = 1.0, CH_3), **2.19** (1H, t, $^4J_{HH}$ = 2.5, C(3)*H*), **2.55 – 2.75** (6H, m, C(2`,5`)H_2 and C(3`)$H_2$), **3.02** (1H, dd, $^3J_{HH}$ = 6.6, 4.5, C(2`)*H*), **4.02** (1H, ddd, $^2J_{HH}$ = 17.6, $^3J_{HH}$ = 5.4, $^4J_{HH}$ = 2.6, C(1)H_AH_B), **4.11** (1H, ddd, $^2J_{HH}$ = 17.6, $^3J_{HH}$ = 5.9, $^4J_{HH}$ = 2.6, C(1)H_AH_B), **5.82** (1H, t, $^3J_{HH}$ = 8.0, C(4`)*H*), **7.02** (1H, s, N*H*), **7.15** (1H, t, $^3J_{HH}$ = 7.9, ArC(5```)*H*), **7.28** (1H, ddd, $^3J_{HH}$ = 7.8 , $^4J_{HH}$ = 1.8 , 1.1 , ArC(4```)*H*), **7.34** (1H, ddd, $^3J_{HH}$ = 7.9 , $^4J_{HH}$ = 2.0 , 1.1 , ArC(6```)*H*), **7.49** (1H, t, $^4J_{HH}$ = 1.8 , ArC(2```)*H*); ^{13}C{^{1}H}-NMR (101 MHz, $CDCl_3$) δ_C; **16.2** (*C*H_3), **23.5** (*C*(3``,4``)H_2), **28.7** (*C*(3`)H_2), **31.1** (NH*C*(1)H_2), **51.8** (*C*(2``,5``)H_2), **68.7** (*C*(2`)H), **71.5** (*C*(3)H), **79.8** (*C*(2)$CH_2$NH), **122.5** (*C*(4`)H), **124.6** (Ar*C*(3```)Br, **124.7** (Ar*C*(6```)H), **129.0** (Ar*C*(5```)H), **129.8** (Ar*C*(4```)H), **135.9** (*C*(5`)), **146.0** (Ar*C*(1```)), **173.0** (*C*(1`)O); HRMS (ESI$^+$) $C_{19}H_{23}BrN_2O$ [M+H]$^+$: found 375.1055, required 375.1067 (–3.2 ppm).

***N*-benzyl-5-cyclopropyl-5-phenyl-2-(pyrrolidin-1-yl)pent-4-enamide 3w**

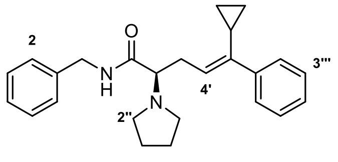

Following General Procedure **F**, (±)-tetramisole hydrochloride (9.6 mg, 0.2 mmol), triethylamine (45 µL, 0.32 mmol), 1-(3-cyclopropyl-3-phenylallyl)-1-(2-(4-nitrophenoxy)-2-oxoethyl)pyrrolidin-1-ium bromide **2w** (97.5 mg, 0.2 mmol), benzylamine (33 µL, 0.3 mmol) in MeCN gave the crude product, which was purified by silica flash chromatography (CH_2Cl_2 : Et_2O 95:5 to 70:30) to give the title compound as a pale yellow solid (16 mg, 55%, 4:1 (*E*)/(*Z*));

ν_{max} (film): 3229, 3055, 3030, 2924, 2805, 1668, 1643, 1549, 1495, 1450, 1416, 1244, 1140, 1028; ^{1}H-NMR (500 MHz, $CDCl_3$) δ_H: **0.24 – 0.32** (1.6H, m, C(7`,8`)H_AH_B (*major*)), **0.35 –0.39** (0.4H, m, C(7`,8`)H_AH_B (*minor*)), **0.57 – 0.64** (0.4H, m, C(7`,8)$H_AH_B$), **0.78 –0.82** (1.6H, m, C(7`,8`)$H_AH_B$ (*major*)), **1.53 – 1.60** (0.2H, m, C(6`)*H* (*minor*)), **1.63 – 1.87** (4H, m, C(3``,4``)H_2 (*major* + *minor*)), **2.36 – 2.49** (0.8H, m, C(6`)*H* (*major*)), **2.55 – 2.68** (3.2H, m, C(2``,5``)H_2 (major)), **2.75 – 2.84** (0.8H,

m, C(2``,5``)H_2 (*minor*)), **2.97 – 3.13** (2H, m, C(3`)$H_2$ (*major* + *minor*)), **4.39 – 4.46** (1H, m, NHC$H_A$$H_B$ (*major* + *minor*)), **4.46 – 4.55** (1H, m, NHC$H_A$$H_B$ (*major* + *minor*)), **5.51** (0.2H, appt, $^3J_{HH}$ = 7.0, C(4`)*H* (*minor*)), **5.76** (0.8H, appt, $^3J_{HH}$ = 6.7, C(4`)*H* (*major*)); $^{13}$C{$^1$H}-NMR (126 MHz, $CDCl_3$) $\delta_C$ (*major*): **6.7** (*C*(7`,8`)$H_2$), **11.7** (*C*(6`)H), **23.5** (*C*(3``,4``)H_2), **31.0** (*C*(3`)H_2), **43.3** (NH*C*H_2), **52.0** (*C*(2``,5``)H_2), **69.1** (*C*(2`)H), **126.5** (*C*(4`)H), **127.5** (Ar*C*(4)H), **127.5** (Ar*C*(2,6)H), **127.8** (Ar*C*(2```,6```)H), **127.9** (Ar*C*(3,5)H), **128.0** (Ar*C*(4```), **128.8** (Ar*C*(3```,5```)H), **138.5** (*C*(5`)), **142.3** (Ar*C*(1)), **142.7** (Ar*C*(1```)), **173.5** (*C*O_2Ar); HRMS (ESI$^+$) $C_{25}H_{30}N_2O$ [M+H]$^+$: found 375.2424, required 375.2431 (–1.9 ppm).

D. 2. Synthesis and characterisation of ^{13}C-labelled compounds

Butyl 2-bromoacetate-1-^{13}*C* S61

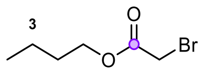

To neat bromo(1-^{13}C)acetic acid (1.00 g, 7.15 mmol) was added oxalyl chloride (665 µL, 7.86 mmol) in a flame-dried shlenk tube under an atmosphere of nitrogen, and the mixture heated at 35 °C for 24 hours. After cooling to 0 °C, *n*-butanol (785 µL, 8.58 mmol) was added dropwise and the mixture stirred for 30 minutes at room temperature. Ethyl acetate (5 mL) and water (5 mL) was added, and the mixture was extracted with ethyl acetate (3 x 10 mL). The organic layers were combined, dried with anhydrous magnesium sulfate, filtered and concentrated *in vacuo* to afford the title compound as a colourless oil (1.40 g, 100%,), which was used immediately;

^{1}H-NMR (400 MHz, $CDCl_3$) δ_H: **0.94** (3H, t, $^3J_{HH}$ = 7.4, CH_3), **1.34 – 1.45** (2H, m, C(3)H_2), **1.60 – 1.69** (2H, m, C(2)H_2), **3.83** (2H, d, $^2J_{HC}$ = 4.7, CH_2Br), **4.17** (2H, td, $^3J_{HH}$ = 6.7, $^3J_{HC}$ = 3.0, OC(1)H_2); ^{13}C{^{1}H}-NMR (101 MHz, $CDCl_3$) δ_C: **13.8** (*C*H_3), **19.1** (*C*(3)H_2), **26.1** (d, $^1J_{CC}$ = 65.2, *C*H_2Br), **30.6** (d, $^3J_{CC}$ = 2.1, *C*(2)H_2), **66.3** (d, $^2J_{CC}$ = 2.6, O*C*(1)H_2)), **167.5** (*C*O^2*n*-Bu).

butyl 2-(diethoxyphosphoryl)acetate-1-^{13}*C* S62

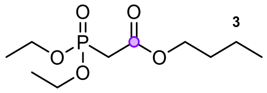

Butyl (1-^{13}C)bromoacetate **S61** (1.40 g, 7.14 mmol) and triethyl phosphite (1.35 mL, 7.85 mmol) were placed in a flame-dried shlenk flask under an atmosphere of nitrogen. The mixture was heated to 120 °C for 24 hours. Remaining triethyl phosphite was removed *in vacuo*, and the residue used without further purification (1.45 g, 80%);

ν_{max} (film): 3051, 2957, 2851, 1775, 1614, 1587, 1518, 1456, 1346, 1292, 1206, 1165, 1086; ^{1}H-NMR (400 MHz, $CDCl_3$) δ_H: **0.92** (3H, t, $^3J_{HH}$ = 7.3, C(6)H_3), **1.31 – 1.44** (8H, m, C(5)H_2 and C(8)H_3), **1.57 – 1.66** (2H, m, C(4)H_2), 2.95 (2H, dd, $^2J_{HC}$ = 21.6 Hz, $^2J_{HP}$ = 7.3 Hz, C(1)H_2, **4.06 – 4.21** (6H, m, C(3)H_2 and C(7)H_2); ^{13}C{^{1}H}-NMR (126 MHz, $CDCl_3$) δ_C: **13.8** (*C*(4)H_3), **16.4** (d, $^3J_{CP}$ = 6.2, 2 × P(O)OCH_2*C*H_3), **19.1** (*C*(3)H_2), **30.6** (*C*(2)H_2), **34.5** (dd, $^1J_{CP}$ = 134.3, $^1J_{CC}$ = 58.8, P(O)*C*$H_2$$CO_2$*n*-Bu), **62.8** (d, $^2J_{CP}$ = 6.3, 2 × P(O)O*C*$H_2$$CH_3$), **65.6** (d, $^2J_{CC}$ = 2.3, O*C*(1)H_2), **166.0** (d, $^2J_{CP}$ = 6.2, *C*=O); HRMS (ESI$^+$) $C_9{}^{13}CH_{21}O_5P$ [M+Na]$^+$: found 276.1246, required 276.1252 (–2.2 ppm).

butyl (*E*)-3-phenylbut-2-enoate-1-^{13}C S63

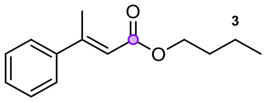

Following General Procedure **A**, butyl 2-(diethoxyphosphoryl)(1-^{13}C)acetate **S62** (1.45 g, 5.71 mmol), sodium hydride (228 mg, 5.71 mmol, 60% in mineral oil), acetophenone (666 µL, 5.71 mmol) in THF gave the crude product, which was purified by column chromatography (Petroleum ether 40/60 : diethyl ether (97:3)) gave the title compound as a colourless oil (649 mg, 52%);

ν_{max} (film): 2959, 2932, 2874, 1670, 1628, 1447, 1342, 1138; ^{1}H-NMR (400 MHz, $CDCl_3$) δ_H: **0.96** (3H, t, $^{3}J_{HH}$ = 7.4 Hz, C(4)H_3), **1.37 – 1.49** (2H, m, C(3)H_2), **1.63 – 1.71** (2H, m, C(2)H_2), **2.58** (3H, t, $^{4}J_{HH}$ = 1.3, C=C(CH_3)Ph), **4.16** (2H, td, $^{3}J_{HH}$ = 6.7, $^{3}J_{HC}$ = 2.9, OC(1)H_2), **6.14** (1H, m, C*H*), **7.33 – 7.41** (3H, m, ArC(2,4,6)*H*), **7.45 – 7.51** (2H, m, ArC(3,5)*H*); ^{13}C{^{1}H}-NMR (101 MHz, $CDCl_3$) δ_C: **13.9** (*C*(4)H_3),**18.1** (*C*(3)H_2), **19.4** (*C*(2)H_2), **30.9** (d, $^{3}J_{CC}$ = 2.3 Hz, *C*(2)H_2), **64.0** (d, $^{2}J_{CC}$ = 2.4 Hz, *C*(1)H_2), **117.4** (d, $^{1}J_{CC}$ = 76.4, *C*(2`)HCO_2*n*-Bu), **126.4** (Ar*C*(2,6)H), **128.6** (Ar*C*(3,5)H), **129.1** (Ar*C*(4)H), **142.4** (d, $^{2}J_{CC}$ = 7.3, C=*C*(CH_3)Ph), **155.6** (Ar*C*(1)), **167.2** (*C*=O); HRMS (ESI$^+$) $C_{13}{}^{13}CH_{18}O_2$ [M+H]$^+$: found 220.1406, required 220.1413 (–3.2 ppm).

(*E*)-3-phenylbut-2-en-1-ol-1-^{13}C S64

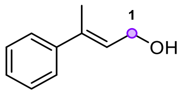

Following General Procedure **B**, butyl (*E*)-3-phenylbut-2-enoate-1-^{13}C **S63** (789 mg, 3.21 mmol), DIBAL-H (7.06 mL, 7.06 mmol, 1 M in hexanes) in Et_2O gave the title compound as a colourless solid (596 mg, 98%);

^{1}H-NMR (400 MHz, $CDCl_3$) δ_H: **1.46** (1H, brs, O*H*), **2.09** (3H, d, $^{4}J_{HH}$ = 0.9, CH_3), **4.37** (2H, dd, $^{1}J_{HC}$ = 142.4, $^{3}J_{HH}$ = 6.8, C=CH_2OH), **5.98** (1H, t, $^{3}J_{HH}$ = 6.8, C=C*H*CH_2OH), **7.24 – 7.29** (1H, m, ArC(4)*H*), **7.31 – 7.36** (2H, m, ArC(2,6)*H*), **7.39 – 7.44** (2H, m, ArC(3,5)*H*); ^{13}C{^{1}H}-NMR (101 MHz, $CDCl_3$) δ_C: **16.2** (d, $^{3}J_{CC}$ = 4.3, *C*H_3), **60.1** (*C*H_2OH), **125.9** (Ar*C*(2,6)H), **126.6** (d, $^{1}J_{CC}$ = 47.9, C=*C*HCH_2OH), **127.4** (Ar*C*(4)H), **128.4** (Ar*C*(3,5)H), **138.0** (Ar(1)*C*(CH_3)=CHCH_2OH), **143.0** (d, $^{2}J_{CC}$ = 5.0, Ar(CH_3)*C*=CHCH_2OH); HRMS (ESI$^+$) $C9^{13}CH_{12}O$ [M–H_2O]$^+$: found 132.0890, required 132.0889 (+0.8 ppm).

(*E*)-(4-bromobut-2-en-2-yl-4-^{13}C)benzene S65

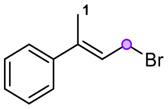

Following General Procedure **C**, (*E*)-3-phenylbut-2-en-1-ol-1-^{13}C **S64** (237 mg, 1.59 mmol), phosphorous tribromide (61 µl, 0.65 mmol) in Et_2O gave the title compound as a pale yellow liquid (304 mg, 90%), which was used immediately without further purification;

^{1}H-NMR (400 MHz, $CDCl_3$) δ_H: **2.15** (3H, d, $^{4}J_{HH}$ = 1.4, CH_3), **4.21** (2H, dd, $^{1}J_{HC}$ = 153.2, $^{2}J_{HC}$ = 8.5, CH_2Br), **6.10** (1H, tdq, $^{3}J_{HH}$ = 8.4, $^{2}J_{HC}$ = 4.0, $^{4}J_{HH}$ = 1.4, C=C*H*CH_2Br), **7.26 – 7.31** (1H, m, ArC(4)*H*), **7.32 – 7.37** (2H, m, ArC(2,6)*H*), **7.39 – 7.43** (2H, m, ArC(3,5)*H*); ^{13}C{^{1}H}-NMR (101 MHz, $CDCl_3$) δ_C: **15.8** (d, $^{3}J_{CC}$ = 4.6, *C*H_3), **29.6** (C=CH*C*H_2Br), **122.9** (d, $^{1}J_{CC}$ = 48.1, C=*C*HCH_2Br), **126.1**

(Ar*C*(2,6)H), **127.9** (Ar*C*(4)H), **128.5** (Ar*C*(3,5)H), **141.6** (Ph(CH_3)*C*=$CHCH_2Br$), **142.3** (d, $^3J_{CC}$ = 5.4, Ar*C*(1)).

(*E*)-1-(3-phenylbut-2-en-1-yl-1-^{13}C)pyrrolidine S66

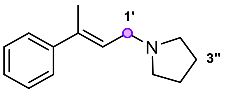

Following General Procedure **D**, pyrrolidine (583 μL, 7.10 mmol), (*E*)-(4-bromo(4-^{13}*C*)but-2-en-2-yl)benzene **S65** (301 mg, 1.42 mmol) in THF gave the title compound as a yellow oil (270 mg, 94%);

^{1}H-NMR (400 MHz, $CDCl_3$) δ_H: **1.76 – 1.86** (4H, m, C(3'',4'')*H*$_2$), **2.07** (3H, d, $^4J_{HH}$ = 1.3 Hz, C*H*$_3$), **2.55 – 2.60** (4H, m, C(2'',5'')*H*$_2$), **3.29** (2H, ddd, $^1J_{HC}$ = 131.9 Hz, $^3J_{HH}$ = 6.7 Hz, $^4J_{HH}$ = 1.0 Hz, C(1')*H*$_2$), (2H, m), **5.94** (1H, tdd, $^3J_{HH}$ = 6.7, $^2J_{HC}$ = 1.5, $^4J_{HH}$ = 1.0, C(2`)*H*), **7.21 – 7.26** (1H, m, ArC(4)*H*), **7.28 – 7.34** (2H, m, ArC(2,6)*H*), **7.38 – 7.43** (2H, m, ArC(3,5)*H*); ^{13}C{^{1}H}-NMR (126 MHz, $CDCl_3$) δ_C: **16.3** (*C*H$_3$), **23.6** (*C*(3``,4``)H$_2$), **54.3** (*C*(1`)H$_2$), **54.5** (*C*(2``,5``)H$_2$), **125.7** (C=*C*(2`)H$CH_2$), **125.9** (Ar*C*(4)H), **127.0** (Ar*C*(2,6)H), **128.3** (Ar*C*(3,5)H), **136.5** (Ph($CH_3$)*C*(3`)=$CHCH_2$), **143.6** (Ar*C*(1)); HRMS (ESI$^+$) $C_{13}{}^{13}CH_{20}N$ [M+H]$^+$: found 203.1617, required 203.1624 (–3.3 ppm).

(*E*)-1-(2-(4-nitrophenoxy)-2-oxoethyl)-1-(3-phenylbut-2-en-1-yl-1-^{13}*C*)pyrrolidin-1-ium bromide 2'-[$^{13}C_1$]-2a

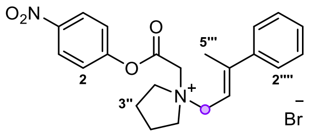

Following General Procedure **E**, (*E*)-1-(3-phenylbut-2-en-1-yl-1-^{13}*C*)pyrrolidine **S66** (123 mg, 610 μmol) and 4-nitrophenyl 2-bromoacetate (474 mg, 1.82 mmol) in MeCN gave the title compound as an off-white solid (205 mg, 73%) with data in agreement with **2a**;

mp.: 140 – 142 °C (MeCN/Et_2O); ν_{max} (film): 3042; 2961, 2884, 1771, 1614, 1587, 1526, 1346, 1203, 1167, 1084; ^{1}H-NMR (400 MHz, d^6-DMSO) δ_H: **2.14** (3H, d, $^4J_{HH}$ = 1.3, C*H*$_3$), **2.14 – 2.20** (4H, m, C(3``,4``)*H*$_2$), **3.79 – 3.93** (4H, d, $^3J_{HH}$ = 7.2, C(2``,5``)*H*$_2$), **4.44** (2H, dd, $^1J_{HC}$ = 146.0, $^3J_{HH}$ = 7.7, C(2```)*H*$_2$), **4.87** (2H, d, $^4J_{HH}$ = 2.5, ArOC(O)C*H*$_2$), **6.10** (1H, t, $^3J_{HH}$ = 7.4, C(3```)*H*), **7.34 – 7.45** (3H, m, ArC*H*), **7.49 – 7.55** (2H, m, ArC(2,6)*H*), **7.57 – 7.63** (2H, m, ArC*H*), **8.34 – 8.41** (2H, m, ArC(3,5)*H*); ^{13}C{^{1}H}-NMR (101 MHz, d^6-DMSO) δ_C: **16.3** (*C*H$_3$), **22.2** (*C*(3``,4``)H$_2$), **58.8** (ArOC(O)*C*H$_2$), **59.6** (*C*(2```)H$_2$), **63.3** (*C*(2``,5``)H$_2$), **114.5** (*C*(3```)H), **123.0** (Ar*C*(2,6)H), **125.6** (Ar*C*(3,5)H), **126.2** (Ar*C*(2```,6```)H), **128.3** (Ar*C*(4````)H), **128.4** (Ar*C*(3```,5```)H), **141.3** (*C*(4```)), **145.6** (Ar*C*(1```)), **146.1** (Ar*C*(4)NO_2), **153.9** (Ar*C*(1)O), **163.9** (ArO*C*(O)CH_2); HRMS (ESI$^+$) $C_{21}{}^{13}CH^{25}N_2O_4{}^+$ [M]$^+$: found 382.1835, required 382.1842 (–1.8 ppm).

4-nitrophenyl 2-bromoacetate-2-^{13}C S67

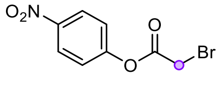

To a solution of bromo(2-^{13}*C*)acetic acid (594 mg, 4.24 mmol) in ethyl acetate (20 mL) was added 4-dimethylaminopyridine (52 mg, 0.424 mmol) and 4-nitrophenol (590 mg, 4.24 mmol). The solution was cooled to 0 °C before addition of *N*,*N'*-dicyclohexylcarbodiimide (874 mg, 4.24 mmol), which was stirred for 4 hours at room temperature. Glacial acetic acid was added (119 μL, 2.9 mmol) and the reaction mixture stirred at −20 °C for 30 minutes, before being filtered through a pad of Celite® and

concentrated *in vacuo*. The residue was dissolved in hot diethyl ether and filtered to remove solid impurities. After concentration *in vacuo*, the solid crude product was recrystallised from hexane/diethyl ether to afford the title compound as an off-white solid (65%, 723 mg);

^{1}H-NMR (500 MHz, $CDCl_3$) δ_H: **4.08** (2H, d, $^{1}J_{CH}$ = 154.4 Hz, C*H*$_2$Br), **7.34** (2H, d, $^{3}J_{HH}$ = 8.6, ArC(2,6)*H*), **8.30** (2H, d, $^{3}J_{HH}$ = 8.6, ArC(3,5)*H*); ^{13}C{^{1}H}-NMR (126 MHz, $CDCl_3$) δ_C; **25.1** (*C*H$_2$Br), **122.2** (Ar*C*(2,6)H), **125.5** (Ar*C*(3,5)H), **145.9** (ArC(4)NO_2), **155.0** (ArC(1)O), **165.1** (*C*O$_2$Ar); HRMS (CI$^+$) $C_7{}^{13}CH_7BrNO_4$ [M+H]$^+$: found 262.9582, required 262.9567 (–5.7 ppm).

(*E*)-1-(2-(4-nitrophenoxy)-2-oxoethyl-1-^{13}*C*)-1-(3-phenylbut-2-en-1-yl)pyrrolidin-1-ium bromide 2-[$^{13}C_1$]-2a

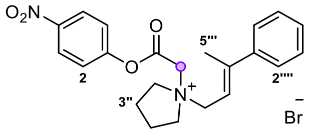

Following General Procedure **E**, (*E*)-1-(3-phenylbut-2-en-1-yl)pyrrolidine **S42** (139 mg, 690 μmol) and 4-nitrophenyl 2-bromoacetate-2-^{13}C **S67** (216 mg, 829 μmol) in MeCN gave the title compound as an off-white solid (271 mg, 85%) with data in agreement with **2a**;

mp.: 140 – 142 °C (MeCN/Et_2O); ν_{max} (film): 2993, 2880, 1769, 1589, 1526, 1348, 1202, 1167; ^{1}H-NMR (400 MHz, d^{6}-DMSO) δ_H: **2.14** (3H, d, $^{4}J_{HH}$ = 1.3, C*H*$_3$), **2.14 – 2.20** (4H, m, C(3``,4``)*H*$_2$), **3.79 – 3.93** (4H, d, $^{3}J_{HH}$ = 7.2, C(2``,5``)*H*$_2$), **4.44** (2H, dd, $^{3}J_{HH}$ = 7.7, C(2```)*H*$_2$), **4.87** (2H, d, $^{1}J_{HC}$ = 139.0, ArOC(O)C*H*$_2$), **6.10** (1H, t, $^{3}J_{HH}$ = 7.4, C(3```)*H*), **7.34 – 7.45** (3H, m, ArC*H*), **7.49 – 7.55** (2H, m, ArC(2,6)*H*), **7.57 – 7.63** (2H, m, ArC*H*), **8.34 – 8.41** (2H, m, ArC(3,5)*H*); ^{13}C{^{1}H}-NMR (101 MHz, d^{6}-DMSO) δ_C: **16.4** (*C*H$_3$), **22.3** (*C*(3``,4``)H$_2$), **58.6** (ArOC(O)*C*H$_2$), **59.7** (*C*(2```)H$_2$), **63.3** (*C*(2``,5``)H$_2$), **114.5** (*C*(3```)H), **123.1** (Ar*C*(2,6)H), **125.6** (Ar*C*(3,5)H), **126.3** (Ar*C*(2```,6```)H), **128.4** (Ar*C*(4````)H), **128.5** (Ar*C*(3```,5```)H), **141.4** (*C*(4```)), **145.6** (Ar*C*(1```)), **146.1** (Ar*C*(4)NO_2), **153.9** (Ar*C*(1)O), **163.9** (ArO*C*(O)CH$_2$, d, $^{1}J_{CC}$ = 63.9); HRMS (ESI$^+$) $C_{21}{}^{13}CH_{24}N_2O_4$ [M+H]$^+$: found 382.1835, required 382.1842 (–1.8 ppm).

(*E*)-1-(2-(4-nitrophenoxy)-2-oxoethyl-1-^{13}*C*)-1-(3-phenylbut-2-en-1-yl-1-^{13}*C*)pyrrolidin-1-ium bromide 2,2'-[$^{13}C_2$]-2a

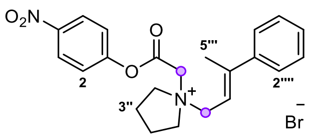

Following General Procedure **E**, (*E*)-1-(3-phenylbut-2-en-1-yl-1-^{13}C)pyrrolidine **S66** (133 mg, 657 μmol) and 4-nitrophenyl 2-bromoacetate-2-^{13}C **S67** (206 mg, 789 μmol) in MeCN gave the title compound as an off-white solid (271 mg, 82%) with data in agreement with **2a**;

mp.: 140 – 142 °C (MeCN/Et_2O); ^{1}H-NMR (400 MHz, d^{6}-DMSO) δ_H: **2.13** (3H, d, $^{4}J_{HH}$ = 1.3, C*H*$_3$), **2.14 – 2.20** (4H, m, C(3``,4``)*H*$_2$), **3.79 – 3.93** (4H, m, C(2``,5``)*H*$_2$), **4.44** (2H, dd, $^{1}J_{HC}$ = 146.0, $^{3}J_{HH}$ = 7.7, C(2```)*H*$_2$), **4.86** (2H, d, $^{1}J_{HC}$ = 147.1, ArOC(O)C*H*$_2$), **6.10** (1H, appt, $^{3}J_{HH}$ = 7.7, C(3```)*H*), **7.34 – 7.45** (3H, m, ArC*H*), **7.49 – 7.55** (2H, m, ArC(2,6)*H*), **7.57 – 7.63** (2H, m, ArC*H*), **8.34 – 8.41** (2H, m, ArC(3,5)*H*); ^{13}C{^{1}H}-NMR (101 MHz, d^{6}-DMSO) δ_C: **16.3** (*C*H$_3$), **22.2** (*C*(3``,4``)H$_2$), **58.5** (ArOC(O)*C*H$_2$), **59.6** (*C*(2```)H$_2$), **63.3** (*C*(2``,5``)H$_2$), **114.3** (*C*(3```)H), **123.0** (Ar*C*(2,6)H), **125.6** (Ar*C*(3,5)H), **126.2** (Ar*C*(2```,6```)H), **128.4** (Ar*C*(4````)H), **128.5** (Ar*C*(3```,5```)H), **141.3** (*C*(4```)),

145.6 (Ar*C*(1```)), **146.1** (Ar*C*(4)NO_2), **153.9** (Ar*C*(1)O), **163.8** (ArO*C*(O)CH_2, d, $^1J_{CC}$ = 63.2); HRMS (ESI^+) $C_{20}{}^{13}C_2H_{25}N_2O_4{}^+$ $[M]^+$: found 383.1862, required 383.1876 (–3.7 ppm).

(*R*,*E*)-*N*-benzyl-5-phenyl-2-(pyrrolidin-1-yl)hex-4-enamide-3-^{13}C 3-[^{13}C$_1$]-3a

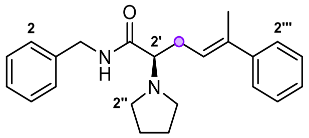

Following General Procedure **F**, (*E*)-1-(2-(4-nitrophenoxy)-2-oxoethyl)-1-(3-phenylbut-2-en-1-yl-1-^{13}*C*)pyrrolidin-1-ium bromide 2'-[^{13}C$_1$]-**2a** (37 mg, 80 µmol), (*S*)-tetramisole hydrochloride (3.9 mg, 16 µmol), triethylamine and benzylamine (13 µL, 0.12 mmol) in MeCN gave the crude product, which was purified by silica flash chromatography (CH_2Cl_2 : Et_2O 95:5 to 70:30) to give the title compound as a pale yellow solid (14 mg, 48%) with data in agreement with **3a**;

mp.: 88–91 °C (CH_2Cl_2); Chiral HPLC analysis, Chiralcel OJ-H (98:2 hexane : IPA, flow rate 2 mLmin^{-1}, 254 nm, 40 °C) t_R (major): 14.3 min, t_R (minor): 24.2 min, 91:9 er; ν_{max} (film): 3292, 3028, 2963, 2926, 2803, 1651, 1514, 1495, 1454, 1358, 1323, 1289, 1240, 1134, 1028; ^{1}H-NMR (400 MHz, $CDCl_3$) δ_H: **1.72 – 1.79** (4H, m, C(3``,4``)*H*$_2$), **2.02** (3H, d, $^4J_{HH}$ = 1.4, C*H*$_3$), **2.44 – 2.65** (1H, m, C(3`)*H*$_A$H$_B$), **2.57 – 2.70** (4H, m, C(2``,5``)*H*$_2$), **2.75 – 2.96** (1H, m, C(3`)H$_A$*H*$_B$), **3.04** (1H, dtd, $^3J_{HH}$ = 7.6 Hz, $^2J_{HC}$ = 4.0 Hz, $^3J_{HH}$ = 1.0, C(2`)*H*), **4.39** (1H, dd, $^2J_{HH}$ = 14.8, $^3J_{HH}$ = 5.6, NHC*H*$_A$H$_B$), **4.53** (1H, dd, $^2J_{HH}$ = 14.8, $^3J_{HH}$ = 6.3, NHCH$_A$*H*$_B$), **5.84** (1H, m, C(4`)*H*), **7.11** (1H, brs, N*H*), **7.23** (5H, s, ArC*H*), **7.23 – 7.28** (1H, m, ArC*H*), **7.28 – 7.33** (4H, m, ArC*H*); ^{13}C{^{1}H}-NMR (101 MHz, $CDCl_3$) δ_C: **16.2** (d, $^3J_{CC}$ = 3.6 Hz, *C*H$_3$), **23.5** (*C*(3``,4``)H$_2$), **31.4** (*C*(3`)H$_2$), **43.3** (NH*C*H$_2$Ph), **52.0** (*C*(2``,5``)H$_2$), **69.1** (*C*(2`)H, d, $^1J_{CC}$ = 34.7), **123.5** (*C*(4`)H, d, $^1J_{CC}$ = 44.4), **125.9** (Ar*C*(2```,6```)H), **126.9** (Ar*C*(4)H), **127.5** (Ar*C*(4```)H), **127.9** (Ar*C*(2,6)H), **128.3** (Ar*C*(3```,5```)H), **128.8** (Ar*C*(3,5)H), **136.9** (Ar*C*(1)), **138.6** (*C*(5`)), **143.8** (Ar*C*(1```), d, $^3J_{CC}$ = 4.6), **173.3** (*C*(1`)(O)NHBn); HRMS (ESI^+) $C_{22}{}^{13}CH_{28}N_2O$ $[M+H]^+$: found 350.2298, required 350.2308 (–2.9 ppm).

(*R*,*E*)-*N*-benzyl-5-phenyl-2-(pyrrolidin-1-yl)hex-4-enamide-2-^{13}C 2-[^{13}C$_1$]-3a

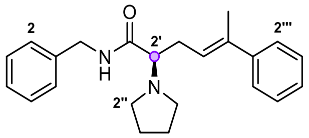

Following General Procedure **F**, (*S*)-tetramisole hydrochloride (3.9 mg, 0.016 mmol), triethylamine (18 µL, 0.128 mmol), (*E*)-1-((1-^{13}C)2-(4-nitrophenoxy)-2-oxoethyl)-1-(3-phenylbut-2-en-1-yl)pyrrolidin-1-ium bromide 2-[^{13}C$_1$]-**2a** (37 mg, 0.08 mmol), benzylamine (13 µL, 0.12 mmol) in MeCN gave the crude product, which was purified by silica flash chromatography (CH_2Cl_2 : Et_2O 95:5 to 70:30) to give the title compound as a pale yellow solid (16 mg, 55%) with data in agreement with **3a**;

mp.: 152 – 153 °C (CH_2Cl_2); ν_{max} (film) 3313, 3275, 2965, 2928, 2799, 1653, 1520, 1495, 1454, 1362, 1244, 1132, 1028; ^{1}H-NMR (400 MHz, $CDCl_3$) δ_H: **1.72 – 1.79** (4H, m, C(3``,4``)*H*$_2$), **2.01** (3H, d, $^4J_{HH}$ = 1.4, C*H*$_3$), **2.44 – 2.65** (5H, m, C(2``,5``)*H*$_2$ and C(3`)*H*$_A$H$_B$), **2.72 – 2.83** (1H, m, C(3`)H$_A$*H*$_B$), **3.04** (1H, ddd, $^1J_{HC}$ = 136.8, $^3J_{HH}$ = 6.6, $^3J_{HH}$ = 4.4, C(2`)*H*), **4.39** (1H, dd, $^2J_{HH}$ = 14.8, $^3J_{HH}$ = 5.6, NHC*H*$_A$H$_B$), **4.52** (1H, dd, $^2J_{HH}$ = 14.8, $^3J_{HH}$ = 6.3, NHCH$_A$*H*$_B$), **5.81 – 5.87** (1H, m, C(4`)*H*), **7.12** (1H, brs, N*H*), **7.20 – 7.25** (5H, s, ArC*H*), **7.27 – 7.36** (5H, m, ArC*H*); ^{13}C{^{1}H}-NMR (101 MHz, $CDCl_3$) δ_C: **16.2** (*C*H$_3$), **23.5** (*C*(3``,4``)H$_2$), **31.4** (*C*(3`)H$_2$, d, $^2J_{HC}$ = 34.7), **43.3** (NH*C*H$_2$Ph), **52.0** (*C*(2``,5``)H$_2$), **69.1** (*C*(2`)H), **123.5** (*C*(4`)H), **125.9** (Ar*C*(2```,6```)H), **126.9** (Ar*C*(4)H), **127.5** (Ar*C*(4```)H), **127.9**

(Ar*C*(2,6)H), **128.3** (Ar*C*(3```,5```)H), **128.8** (Ar*C*(3,5)H), **136.9** (Ar*C*(1)), **138.6** (*C*(5`)), **143.8** (Ar*C*(1```)), **173.3** (*C*(1`)(O)NHBn); HRMS (ESI$^+$) $C_{22}{}^{13}CH_{28}N_2O$ [M+H]$^+$: found 350.2296, required 350.2308 (–3.4 ppm).

(*R*,*E*)-*N*-benzyl-5-phenyl-2-(pyrrolidin-1-yl)hex-4-enamide-1,2-$^{13}C_2$ 2,3-[$^{13}C_2$]-3a

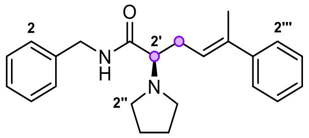

Following General Procedure **F**, (*S*)-tetramisole hydrochloride (3.9 mg, 0.016 mmol), triethylamine (18 µL, 0.128 mmol), (*E*)-1-((1-^{13}C)2-(4-nitrophenoxy)-2-oxoethyl)-1-(3-phenylbut-2-en-1-yl-1-^{13}C)pyrrolidin-1-ium bromide 2,2'-[$^{13}C_2$]-**2a** (37 mg, 0.08 mmol), benzylamine (13 µL, 0.12 mmol) in MeCN gave the crude product, which was purified by silica flash chromatography (CH_2Cl_2 : Et_2O 95:5 to 70:30) to give the title compound as a pale yellow solid (16 mg, 55%) with data in agreement with **3a**;

mp.: 151 – 153 °C (CH_2Cl_2); ν_{max} (film) 3313, 3275, 2965, 2928, 2799, 1653, 1520, 1495, 1454, 1362, 1244, 1132, 1028; ^{1}H-NMR (400 MHz, $CDCl_3$) δ_H: **1.72 – 1.79** (4H, m, C(3``,4``)*H*$_2$), **2.01** (3H, d, $^4J_{HH}$ = 1.4, C*H*$_3$), **2.44 – 2.65** (5H, m, C(2``,5``)*H*$_2$ and C(3`)*H*$_A$H$_B$), **2.72 – 2.83** (1H, m, C(3`)H$_A$*H*$_B$), **3.04** (1H, ddd, $^1J_{HC}$ = 136.8, $^3J_{HH}$ = 6.6, $^3J_{HH}$ = 4.4, C(2`)*H*), **4.39** (1H, dd, $^2J_{HH}$ = 14.8, $^3J_{HH}$ = 5.6, NHC*H*$_A$H$_B$), **4.52** (1H, dd, $^2J_{HH}$ = 14.8, $^3J_{HH}$ = 6.3, NHCH$_A$*H*$_B$), **5.81 – 5.87** (1H, m, C(4`)*H*), **7.12** (1H, brs, N*H*), **7.20 – 7.25** (5H, s, ArC*H*), **7.27 – 7.36** (5H, m, ArC*H*); ^{13}C{^{1}H}-NMR (101 MHz, $CDCl_3$) δ_C: **16.2** (*C*H$_3$), **23.5** (*C*(3``,4``)H$_2$), **31.4** (*C*(3`)H$_2$, d, $^1J_{CC}$ = 34.6), **43.3** (NH*C*H$_2$Ph), **52.0** (*C*(2``,5``)H$_2$), **69.1** (*C*(2`)H, d, $^1J_{CC}$ = 34.6), **123.5** (*C*(4`)H), **125.9** (Ar*C*(2```,6```)H), **126.9** (Ar*C*(4)H), **127.5** (Ar*C*(4```)H), **127.9** (Ar*C*(2,6)H), **128.3** (Ar*C*(3```,5```)H), **128.8** (Ar*C*(3,5)H), **136.9** (Ar*C*(1)), **138.6** (*C*(5`)), **143.8** (Ar*C*(1```)), **173.3** (*C*(1`)(O)NHBn); HRMS (ESI$^+$) $C_{21}{}^{13}C_2H_{28}N_2O_4$ [M+H]$^+$: found 351.2336, required 351.2341 (–1.4 ppm).

Ethyl (*E*)-3-phenylbut-2-enoate-3-^{13}C S68

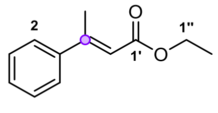

Following General Procedure **A**, triethyl phosphonoacetate (3.27 mL, 16.5 mmol), sodium hydride (660 mg, 16.5 mmol, 60% in mineral oil), acetophenone-(*carbonyl*-^{13}C) (1.94 mL, 16.5 mmol) in THF gave the crude product, which was purified by column chromatography (Petroleum ether 40/60 : diethyl ether (97:3)) to give the title compound as a colourless oil (1.20 g, 38%);

^{1}H-NMR (400 MHz, $CDCl_3$) δ_H: **1.32** (3H, t, $^3J_{HH}$ = 7.1, C(2`)*H*$_3$), **2.58** (3H, dd, $^3J_{HH}$ = 6.6, $^4J_{HH}$ = 1.3, C(4)*H*$_3$), **4.22** (2H, q, $^3J_{HH}$ = 7.1, C(1`)*H*$_2$), **6.12 – 6.14** (1H, m, C(2)*H*), **7.34 – 7.41** (3H, m, ArC*H*), **7.45 – 7.50** (2H, m, ArC*H*); ^{13}C{^{1}H}-NMR (101 MHz, $CDCl_3$) δ_C: **14.5** (*C*(2`)H$_3$), **18.1** (*C*(4`)H$_3$, d, $^1J_{CC}$ = 40.5), **60.0** (O*C*(1``)H$_2$), **117.3** (d, $^1J_{CC}$ = 73.0, *C*(2`)H), **126.4** (Ar*C*(3,5)H), **128.6** (Ar*C*(2,6)H), **129.1** (Ar*C*(4)H), **142.4** (d, $^1J_{CC}$ = 51.8, Ar*C*(1)H), **155.7** (*C*(3`)), **167.0** (*C*(1`)O$_2$Et); HRMS (ESI$^+$) $C_{11}{}^{13}CH_{14}O_2$ [M+H]$^+$: found 192.1095, required 192.1100 (−2.6 ppm).

(*E*)-3-phenylbut-2-en-1-ol-3-^{13}*C*   S69

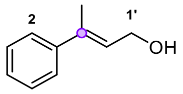

Following General Procedure **B**, (*E*)-[3-^{13}C]-3-phenylbut-2-enoate **S68** (1.19 g, 6.22 μmol) and DIBAL-H (13.7 mL, 13.7 mmol, 1 M in hexanes) in anhydrous Et_2O gave the title compound as a colourless oil (928 mg, 99%);

^{1}H-NMR (500 MHz, $CDCl_3$) δ_H: **1.37** (1H, t, $^3J_{HH}$ = 5.1, O*H*), **2.09** (3H, d, $^2J_{HC}$ = 6.2, C*H*$_3$), **4.37** (2H, q, $^3J_{HH}$ = 5.2, C*H*$_2$OH), **5.98** (1H, tp, $^3J_{HH}$ = 6.7, $^4J_{HH}$ = 1.3, C*H*CH2OH), **7.25 – 7.28** (1H, m, ArC(4)*H*), **7.31 – 7.35** (2H, m, ArC(2,6)*H*), **7.39 – 7.43** (2H, m, ArC(3,5)*H*); ^{13}C{^{1}H}-NMR (126 MHz, $CDCl_3$) δ_C: **16.2** (*C*H$_3$), **60.1** (*C*H$_2$OH), **125.9** (Ar*C*(3,5)), **126.6** (*C*(2\`)H, d, $^1J_{CC}$ = 73.4), **127.4** (Ar*C*(4)H), **128.4** (Ar*C*(2,6)H d, $^2J_{CC}$ = 3.9), **138.1** (*C*(3\`)), **143.0** (ArC(1), d, $^1J_{CC}$ = 53.5); HRMS (ESI$^-$) $C_9{}^{13}CH_{12}O$ [M-H]$^-$: found 148.0847, required 148.0849 (−1.4 ppm).

(*E*)-(4-Bromobut-2-en-2-yl-2-^{13}*C*)benzene S70

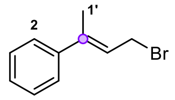

Following General Procedure **C**, (*E*)-3-phenylbut-2-en-1-ol-3-^{13}C **S69** (935 mg, 6.27 mmol), phosphorous tribromide (241 μl, 2.57 mmol) in Et_2O gave the title compound as a pale-yellow liquid (304 mg, 90%), which was used immediately without further purification;

^{1}H-NMR (500 MHz, $CDCl_3$) δ_H: **2.15** (3H, dd, $^2J_{HC}$ = 6.3, $^4J_{HH}$ = 1.4), **4.21** (2H, dd, $^3J_{HH}$ = 8.5, $^3J_{HC}$ = 5.6, CHC*H*$_2$Br), **6.09** (1H, apptq, $^3J_{HH}$ = 8.5, $^4J_{HH}$ = 1.4, C*H*CH$_2$Br), **7.26 – 7.31** (1H, m, ArC(4)*H*), **7.32 – 7.36** (2H, m, ArC(2,6)*H*), **7.38 – 7.43** (2H, m, ArC(3,5)*H*); ^{13}C{^{1}H}-NMR (126 MHz, $CDCl_3$) δ_C: **15.8** (*C*H$_3$, d, $^1J_{CC}$ = 43.0), **29.6** (*C*H$_2$Br), **122.9** (*C*(3\`)HCH$_2$Br, d, $^1J_{CC}$ = 73.8), **126.1** (Ar*C*(3,5)H), **128.0** (Ar*C*(4)H), **128.5** (Ar*C*(2,6)H, d, $^2J_{CC}$ = 3.9), **141.7** (*C*(2\`)CH$_3$), **148.4** (Ar*C*(1)); HRMS (ESI$^+$) $C_9{}^{13}CH_{11}Br$ [M–Br]$^+$: found 132.0892, required 132.0889 (+2.3 ppm).

(*E*)-1-(3-phenylbut-2-en-1-yl-3-^{13}*C*)pyrrolidine S71

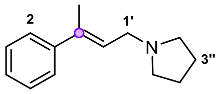

Following General Procedure **D**, pyrrolidine (583 μL, 7.10 mmol), (*E*)-(4-bromobut-2-en-2-yl-2-^{13}C)benzene **S70** (301 mg, 1.42 mmol) in THF gave the title compound as a yellow oil (270 mg, 94%);

ν_{max} (film): 2955, 2924, 2778, 1599, 1493, 1445, 1371, 1346, 1138, 1028; ^{1}H-NMR (400 MHz, $CDCl_3$) δ_H: **1.79 – 1.83** (4H, m, C(3\`\`,4\`\`)*H*$_2$), **2.07** (3H, appdq, $^3J_{HH}$ = 6.2, $^4J_{HH}$ = 1.0, C*H*$_3$), **2.54 – 2.62** (4H, m, C(2\`,5\`)*H*$_2$), **3.30** (dd, $^3J_{HH}$ = 6.6, $^3J_{HC}$ = 4.7, C(1\`)*H*$_2$), **5.91 − 5.94** (1H, m, C(2)*H*), **7.21 – 7.25** (1H, m, ArC(4)*H*), **7.28 – 7.34** (2H, m, ArC(2,6)*H*), **7.37 – 7.44** (2H, m, ArC(3,5)*H*); $^{13}$C{$^1$H}-NMR (101 MHz, $CDCl_3$) $\delta_C$: **16.3** (*C*H$_3$, d, $^1J_{CC}$ = 43.3), **23.6** (*C*(3\`\`,4\`\`)H$_2$), **54.4** (*C*(2\`\`,5\`\`)H$_3$), **74.8** (*C*(1\`)H$_2$), **125.8** (C(2), d, $^1J_{CC}$ = 73.8), **125.9** (Ar*C*(3,5)H), **127.0** (Ar*C*(4)H), **128.3** (Ar*C*(2,6)H, d, $^2J_{CC}$ = 3.9), **136.4** (*C*(3)CH$_3$), **143.6** (Ar*C*(1), d, $^1J_{CC}$ = 53.9); HRMS (ESI$^+$) $C_{13}{}^{13}CH_{18}N$ [M+H]$^+$: found 203.1617, required 203.1624 (−3.4 ppm).

(*E*)-1-(2-(4-nitrophenoxy)-2-oxoethyl)-1-(3-phenylbut-2-en-1-yl-3-^{13}*C*)pyrrolidin-1-ium bromide 4'-[$^{13}C_1$]-2a

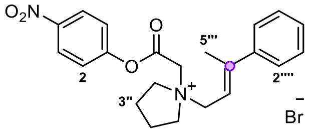

Following General Procedure **E**, (*E*)-1-(3-phenylbut-2-en-1-yl-3-^{13}*C*)pyrrolidine **S71** (405 mg, 2.00 mmol) and 4-nitrophenyl 2-bromoacetate (624 mg, 2.40 mmol) in MeCN gave the title compound as a beige powder (569 mg, 62%) with data in agreement with **2a**;

mp 143 − 145 °C (MeCN/Et_2O); ν_{max} (film): 3051, 2957, 2851, 1775, 1614, 1587, 1518, 1346, 1206, 1165; ^{1}H-NMR (500 MHz, d^6-DMSO) δ_H: **2.14** (3H, d, $^2J_{HC}$ = 6.2, C*H*$_3$), **2.15 – 2.23** (4H, m, C(3``,4``)*H*$_2$), **3.82 – 3.96** (4H, m, C(2``,5``)*H*$_2$), **4.48** (2H, d, $^3J_{HH}$ = 7.8, C(2```)*H*$_2$), **4.97** (2H, s, ArOC(O)C*H*$_2$), **6.11** (1H, t, $^3J_{HH}$ = 7.7, C(3```)*H*), **7.34 – 7.38** (1H, m, ArC(4````)*H*), **7.41** (2H, d, $^3J_{HH}$ = 8.3, ArC(2,6)*H*), **7.53 – 7.57** (2H, m, ArC(2````,6````)*H*), **7.59 – 7.66** (2H, m, ArC(3````,5````)*H*), **8.33 – 8.39** (2H, d, $^3J_{HH}$ = 8.3, ArC(3,5)*H*); ^{13}C{^{1}H}-NMR (126 MHz, DMSO-d6) δ_C: **16.4** (*C*H$_3$, d, $^1J_{CC}$ = 42.5), **22.3** (*C*(3``,4``)H$_2$), **58.6** (ArOC(O)*C*H$_2$), **59.7** (*C*(2```)H$_2$), **63.3** (*C*(2``,5``)H$_2$), **114.6** (*C*(3```)H, d, $^1J_{CC}$ = 73.7), **123.1** (Ar*C*(2,6)*H*), **125.5** (Ar*C*(3````,5````)*H*, **126.2** (Ar*C*(3,5)*H*), **128.4** (ArC(4````)*H*), **128.4** (ArC(2````,6````)*H*), **141.3** (ArC(1````), d, $^1J_{CC}$ = 52.9), **145.6** (Ar*C*(4)NO$_2$), **146.0** (*C*(4```)CH$_3$), **153.9** (Ar*C*(1)), **163.9** (ArO*C*(O)CH$_2$); HRMS (ESI$^+$) $C_{21}{}^{13}CH_{25}N_2O_4$ [M]$^+$: found 382.1832, required 382.1842 (−2.6 ppm).

(*E*)-1-(2-(4-nitrophenoxy)-2-oxoethyl)-1-(3-phenylbut-2-en-1-yl-3-^{13}*C*)pyrrolidin-1-ium bromide 2,4'-[$^{13}C_2$]-2a

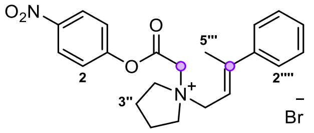

Following General Procedure **E**, (*E*)-1-(3-phenylbut-2-en-1-yl-3-^{13}*C*)pyrrolidine **S71** (405 mg, 2.00 mmol) and 4-nitrophenyl 2-bromoacetate-2-$^{13}C_1$ **S67** (624 mg, 2.40 mmol) in MeCN gave the title compound as a beige powder (569 mg, 62%) with data in agreement with **2a**;

mp 143 − 145 °C (MeCN/Et_2O); ν_{max} (film): 3051, 2957, 2851, 1775, 1614, 1587, 1518, 1346, 1206, 1165; ^{1}H-NMR (500 MHz, d^6-DMSO) δ_H: **2.14** (3H, d, $^2J_{HC}$ = 6.1, C*H*$_3$), **2.15 – 2.23** (4H, m, C(3``,4``)*H*$_2$), **3.82 – 3.96** (4H, m, C(2``,5``)*H*$_2$), **4.48** (2H, appbrs, C(2```)*H*$_2$), **4.97** (2H, d, $^1J_{HC}$ = 146.8, ArOC(O)C*H*$_2$), **6.11** (1H, t, $^3J_{HH}$ = 7.7, C(3```)*H*), **7.34 – 7.38** (1H, m, ArC(4````)*H*), **7.41** (2H, d, $^3J_{HH}$ = 8.3, ArC(2,6)*H*), **7.53 – 7.57** (2H, m, ArC(2````,6````)*H*), **7.59 – 7.66** (2H, m, ArC(3````,5````)*H*), **8.33 – 8.39** (2H, d, $^3J_{HH}$ = 8.3, ArC(3,5)*H*); ^{13}C{^{1}H}-NMR (126 MHz, DMSO-d6) δ_C: **16.4** (*C*H$_3$, d, $^1J_{CC}$ = 42.2), **22.3** (*C*(3``,4``)H$_2$), **58.6** (ArOC(O)*C*H$_2$), **59.7** (*C*(2```)H$_2$), **63.3** (*C*(2``,5``)H$_2$), **114.6** (*C*(3```)H, d, $^1J_{CC}$ = 73.7), **123.1** (Ar*C*(2,6)*H*), **125.5** (Ar*C*(3````,5````)*H*, **126.2** (Ar*C*(3,5)*H*), **128.4** (ArC(4````)*H*), **128.4** (ArC(2````,6````)*H*), **141.3** (ArC(1````), d, $^1J_{CC}$ = 52.9), **145.6** (Ar*C*(4)NO$_2$), **146.0** (*C*(4```)CH$_3$), **153.9** (Ar*C*(1)), **163.9** (d, $^1J_{CC}$ = 62.4, ArOC(O)*C*H$_2$); HRMS (ESI$^+$) $C_{20}{}^{13}C_2H_{25}N_2O_4$ [M]$^+$: found 383.1862, required 383.1876 (−3.6 ppm).

4-Nitrophenyl (2*R*,2*S*)-3-methyl-3-phenyl-2-(pyrrolidin-1-yl)pent-4-enoate-1,2-^{13}*C*$_2$

2,3-[^{13}C$_2$]-4a

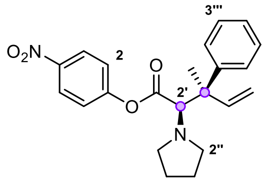

Following General Procedure **F**, (*S*)-tetramisole hydrochloride (7.2 mg, 0.03 mmol), triethylamine (33 µL, 0.24 mmol), (*E*)-1-((1-^{13}C)2-(4-nitrophenoxy)-2-oxoethyl)-1-(3-phenylbut-2-en-1-yl-3-^{13}C)pyrrolidin-1-ium bromide 2,4'-[^{13}C$_2$]-**2a** (69.5 mg, 0.15 mmol), benzylamine (25 µL, 0.225 mmol) in MeCN gave the crude product, which was purified by silica flash chromatography (CH_2Cl_2 : Et_2O 95:5 to 70:30) to give the title compound as a pale yellow solid (6.3 mg, 11%) with data in agreement with **4a**;

^{1}H-NMR (400 MHz, $CDCl_3$) δ_H: **1.66 – 1.69** (3H, m, C*H*$_3$), **1.70 – 1.77** (4H, m, C(3``,4``)*H*$_2$), **2.68 – 2.87** (4H, m, C(2``,5``)*H*$_2$), **4.10** (1H, dd, $^1J_{HC}$ = 137.9, $^2J_{HC}$ = 4.2, C(2`)*H*), **5.20 – 5.35** (2H, m, C(5`)*H*$_2$), **6.73** (1H, ddt, $^3J_{HH}$ = 17.5, 11.0, $^2J_{HC}$ = 2.3, C(4`)*H*), **6.83 – 6.89** (2H, m, ArC(2,6)*H*), **7.22 – 7.25** (1H, m, ArC(4```)*H*), **7.34** (2H, appt, $^3J_{HH}$ = 7.6, ArC(2```,6```)H), **7.43 – 7.48** (2H, m, ArC(3```,5```)*H*), **8.13 – 8.20** (2H, m, ArC(3,5)*H*); ^{13}C{^{1}H}-NMR (126 MHz, $CDCl_3$) δ_C: **22.8** (*C*H$_3$, d, $^1J_{CC}$ = 36.6), **23.9** (*C*(3``,4``)H$_2$, d, $^3J_{CC}$ = 3.7), **48.0** (*C*(3`), d, $^1J_{CC}$ = 37.1), **52.3** (*C*(2``,5``)H$_2$), **74.1** (*C*(2`)H, d, $^1J_{CC}$ = 37.1), **115.1** (*C*(5`)H$_2$), **122.7** (Ar*C*(2,6)H), **125.2** (ArC(3```,5```)H), **126.7** (Ar*C*(4```)H), **126.9** (Ar*C*(3```,5```)H), **128.4** (Ar*C*(2```,6```)H, d, $^2J_{CC}$ = 3.2), **142.7** (*C*(4`)H, d, $^2J_{CC}$ = 42.3), **145.4** (Ar*C*(4)NO$_2$), **146.3** (Ar*C*(1```), d, $^1J_{CC}$ = 42.4), **155.1** (Ar*C*(1)O), **168.6** (*C*(1`)O$_2$Ar).

Butyl 3,3-diphenylacrylate-1-^{13}C S72

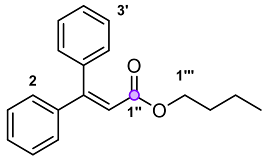

Following General Procedure **A**, butyl 2-(diethoxyphosphoryl)acetate-1-^{13}C **S62** (1.60 g, 6.30 mmol), sodium hydride (252 mg, 6.30 mmol, 60% in mineral oil), benzophenone (1.15 g, 6.30 mmol) in THF gave the crude product, which was purified by column chromatography (Petroleum ether 40/60 : diethyl ether (99:1), R$_f$ 0.10) to give the title compound as a colourless oil (1.11 g, 63%) which was used directly;

ν_{max} (film): 3057, 2959, 2934, 2872, 1682, 1663, 1614, 1445, 1356, 1258, 1134, 1032; ^{1}H-NMR (400 MHz, $CDCl_3$) δ_H: **0.86** (4H, t, $^3J_{HH}$ = 7.4, C(4`)*H*$_3$), **1.17 – 1.28** (2H, m, C(3`)*H*$_2$CH$_3$), **1.45** (2H, m, C(2`)*H*$_2$CH$_2$CH$_3$), **4.00** (2H, td, $^3J_{HH}$ = 6.6, $^4J_{HH}$ = 2.8, OC(1`)H$_2$), **6.37** (1H, d, $^2J_{HC}$ = 1.8, C(2)*H*), **7.18 – 7.23** (2H, m, ArC*H*), **7.28 – 7.36** (5H, m, ArC*H*), **7.36 – 7.40** (3H, m, ArC*H*); 13{1H}-NMR (126 MHz, $CDCl_3$) δ_C: **13.8** (*C*H$_3$), **19.2** (*C*H$_2$CH$_3$), **30.6** (OCH$_2$*C*H$_2$), **64.2** (O*C*H$_2$CH$_2$), **117.7** (d, $^1J_{CC}$ = 76.6, *C*(2`)H), **128.0** (Ar*C*H), **128.2** (Ar*C*H), **128.4** (Ar*C*H), **128.5** (Ar*C*H), **129.2** (Ar*C*H), **129.5** (Ar*C*H), **139.2** (Ar*C*H), **141.0** (Ar*C*H), 156.5 (C(3`)), **166.4** (*C*O$_2$Et) ; HRMS (ESI$^+$) $C_{16}{}^{13}CH_{16}O_2$ [M+H]$^+$: found 254.1254, required 254.1257 (−1.1 ppm).

3,3-diphenylprop-2-en-1-ol-3-^{13}C S73

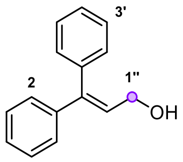

Following General Procedure **B**, butyl 3,3-diphenylacrylate-1-^{13}C **S72** (1.10 g, 3.91 mmol) and DIBAL-H (8.6 mL, 8.6 mmol, 1 M in hexanes) in anhydrous Et_2O gave the title compound as a colourless oil (672 mg, 82%) which was used directly;

ν_{max} (film): 3385, 3080, 3055, 3024, 2859, 1722, 1659, 1597, 1493, 1445, 1279, 999; ^{1}H-NMR (500 MHz, $CDCl_3$) δ_H: **1.47** (1H, s, O*H*), **4.22** (2H, dd, $^1J_{HC}$ = 143.4, $^3J_{HH}$ = 6.8, C(1``)*H*$_2$OH), **6.25** (1H, t, $^3J_{HH}$ = 6.8, C(2'')*H*CH$_2$OH), **7.15 – 7.19** (2H, m, ArC*H*), **7.25 – 7.31** (5H, m, ArC*H*), **7.32 – 7.40** (3H, m, ArC*H*); HRMS (ESI$^-$) $C_{14}{}^{13}CH_{14}O$ [M–H]$^-$: found 210.1003, required 210.1005 (–1.0 ppm)

(3-bromoprop-1-ene-1,1-diyl-3-^{13}C)dibenzene S74

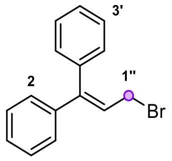

Following General Procedure **C**, 3,3-diphenylprop-2-en-1-ol-1-^{13}C **S73** (672 mg, 3.18 mmol), phosphorous tribromide (123 µl, 1.30 mmol) in Et_2O gave the title compound as a pale yellow liquid (850 mg, 98%), which was used immediately without further purification;

^{1}H-NMR (500 MHz, $CDCl_3$) δ_H: **4.06** (1H, dd, $^1J_{HC}$ = 154.2, $^3J_{HH}$ = 8.5, C(1``)*H*$_2$Br), **6.34** (1H, td, $^3J_{HH}$ = 8.5, $^2J_{HC}$ = 2.3, C(2``)*H*CH$_2$Br), **7.21 – 7.32** (4H, m, ArC*H*), **7.35 – 7.40** (1H, m, ArC*H*), **7.40 – 7.45** (1H, m, ArC*H*); ^{13}C{^{1}H}-NMR (126 MHz, $CDCl_3$) δ_C: **31.4** (*C*(1``)H$_2$Br), **123.9** (*C*(2``)HCH$_2$Br, d, $^1J_{CC}$ = 48.4), **127.9** (Ar*C*(2`,6`)H), **128.0** (Ar*C*(4`)H), **128.2** (Ar*C*(4)H), **128.4** (Ar*C*(2,6)H), **128.6** (Ar*C*(3`,5`)H), **129.6** (Ar*C*(3,5)H), **138.3** (Ar*C*(1`), d, $^3J_{CC}$ = 3.1), **141.3** (*C*(3``)Ph$_2$, d, $^2J_{CC}$ = 5.1), **146.3** (Ar*C*(1)).

1-(3,3-diphenylallyl-1-^{13}C)pyrrolidine S75

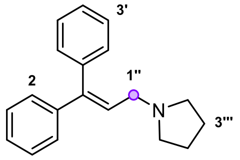

Following General Procedure **D**, pyrrolidine (1.27 mL, 15.5 mmol), (3-bromoprop-1-ene-1,1-diyl-3-^{13}C)dibenzene **S74** (847 mg, 3.09 mmol) in THF gave the title compound as a yellow oil (683 mg, 84%);

ν_{max} (film): 3055, 3026, 2963, 2781, 1684, 1628, 1597, 1495, 1445, 1277, 1126, 1074, 1030, 914; ^{1}H-NMR (500 MHz, $CDCl_3$) δ_H: **1.74 – 1.81** (4H, m, C(3```,4```)*H*$_2$), **2.47 − 2.53** (4H, m, C(2```,5```)*H*$_2$), **3.19** (2H, dd, $^1J_{HC}$ = 132.9, $^3J_{HH}$ = 6.7, CHC(1``)*H*$_2$), **6.27** (1H, t, $^3J_{HH}$ = 6.7, C(2``)*H*), **7.14 – 7.19** (2H, m, ArC*H*), **7.21 – 7.28** (5H, m, ArC*H*), **7.31 – 7.35** (1H, m, ArC*H*), **7.35 − 7.39** (2H, m, ArC*H*); ^{13}C{^{1}H}-NMR (126 MHz, $CDCl_3$) δ_C: **23.5** (*C*(3```,4```)H$_2$), **54.1** (*C*(2```,5```)H$_2$), **54.9** (*C*(1``)H$_2$), **127.0** (*C*(2``)*H*), **127.1** (Ar*C*(4`)H), **127.2** (Ar*C*(4)H) **127.4** (Ar*C*(2`,6`)H), **128.2** (Ar*C*(2,6)H), **128.2** (Ar*C*(3`,5`)H), **129.9** (Ar*C*(3,5)H), **139.8** (Ar*C*(1`), d, $^3J_{CC}$ = 2.0), **142.2** (*C*(3``)Ph$_2$, d, $^2J_{CC}$ = 4.6), **142.8** (Ar*C*(1)); HRMS: (ESI$^+$) $C_{18}{}^{13}CH_{21}N$ [M+H]$^+$: found 265.1771, required 265.1780 (–3.4 ppm).

1-(3,3-diphenylallyl)-1-(2-(4-nitrophenoxy)-2-oxoethyl-2-^{13}C)pyrrolidin-1-ium bromide 2-[$^{13}C_1$]-2e

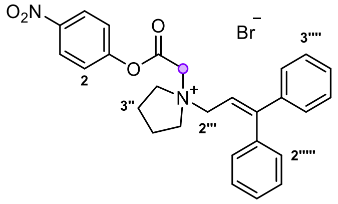

Following General Procedure **E**, 1-(3,3-diphenyl-1-allyl)pyrrolidine **S46** (140 mg, 532 µmol) and 4-nitrophenyl 2-bromoacetate-2-^{13}C (153 mg, 585 µmol) in MeCN gave the title compound as an off-white solid (226 mg, 81%) with data in agreement with **2e**;

mp 153 − 155 °C (dec.); ν_{max} (film): 3026, 2951, 1769, 1520, 1342, 1190, 1157; ^{1}H-NMR (500 MHz, DMSO-d6) δ_H: **1.90 – 2.18** (4H, m, C(3``,4``)*H*$_2$), **3.77 – 3.83** (4H, m, C(2``,5``)*H*$_2$), **4.30** (2H, d, $^3J_{HH}$ = 6.8, C(2```)*H*$_2$), **4.86** (2H, d, $^1J_{HC}$ = 147.1, C(2`)*H*$_2$), **6.45** (1H, t, $^3J_{HH}$ = 6.8, C(3```)*H*), **7.12 – 7.18** (2H, m, ArC(4````)*H* and ArC(4`````)*H*), **7.33 − 7.42** (8H, m, ArC*H*), **7.45** (2H, d, $^3J_{HH}$ = 9.0, ArC(2,6)*H*), **8.36** (2H, d, $^3J_{HH}$ = 9.0, ArC(3,5)*H*); ^{13}C{^{1}H}-NMR: (126 MHz, DMSO-d6) δ_C: **21.9** (*C*(3``,4``)H$_2$), **58.2** (ArOC(O)*C*H$_2$), **59.1** (*C*(2```)H$_2$), **63.6** (*C*(2``,5``)H$_2$), **115.5** (*C*(3```)H), **122.9** (Ar*C*(2,6)H), **125.5** (Ar*C*(3`````,5`````)H), **127.8** (Ar*C*(3````,5````)H), **128.4** (ArC(4`````)H), **128.4** (Ar*C*(3,5)H), **128.7** (Ar*C*(4````)H), **128.8** (Ar*C*(2`````,6`````)H), **129.3** (Ar*C*(2````,6````)H), **137.2** (Ar*C*(1`````)), **140.4** (Ar*C*(1````)), **145.5** (Ar*C*(4)NO$_2$), **150.7** (*C*(4```)), **153.8** (Ar*C*(1)O), **163.3** (ArO*C*(O)CH$_2$); HRMS (ESI$^+$) $C_{26}{}^{13}CH_{27}N_2O_4$ [M]$^+$: found 444.1983, required 444.1999 (−3.6 ppm).

1-(3,3-diphenylallyl-1-^{13}C)-1-(2-(4-nitrophenoxy)-2-oxoethyl)pyrrolidin-1-ium bromide 2'-[$^{13}C_1$]-2e

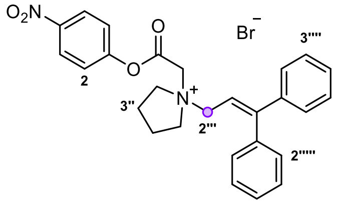

Following General Procedure **E**, 1-(3,3-diphenyl-1-allyl-1-^{13}C)pyrrolidine **S75** (233 mg, 880 µmol) and 4-nitrophenyl 2-bromoacetate (252 mg, 970 µmol) in MeCN gave the title compound as an off-white solid (333 mg, 72%) with data in agreement with **2e**;

mp 153 − 155 °C (dec.); ν_{max} (film): 3044, 2970, 1771, 1614, 1591, 1518, 1344, 1153; ^{1}H-NMR (500 MHz, d^6-DMSO) δ_H: **1.85 – 2.21** (4H, m, C(3``,4``)*H*$_2$), **3.73 – 3.90** (4H, m, C(2``,5``)*H*$_2$), **4.31** (2H, dd, $^1J_{HC}$ = 146.7, $^3J_{HH}$ = 7.2, C(2```)*H*$_2$), **4.90** (2H, s, ArOC(O)C*H*$_2$), **6.45** (1H, t, $^3J_{HH}$ = 7.2, C(3```)*H*), **7.11 – 7.17** (2H, m, ArC(4````)*H* and ArC(4`````)*H*), **7.34 − 7.42** (8H, m, ArC*H*), **7.46** (2H, d, $^3J_{HH}$ = 9.0, ArC(2,6)*H*), **8.36** (2H, d, $^3J_{HH}$ = 9.0, ArC(3,5)*H*); ^{13}C{^{1}H}-NMR (126 MHz, d^6-DMSO) δ_C: **21.9** (*C*(3``,4``)H$_2$), **58.4** (ArOC(O)*C*H$_2$), **59.1** (*C*(2```)H$_2$), **63.6** (*C*(2``,5``)H$_2$), **115.5** (*C*(3```)H, d, $^2J_{CC}$ = 47.0), **122.9** (Ar*C*(2,6)H), **125.5** (Ar*C*(2`````,6`````)H), **127.8** (Ar*C*(2````,6````)H), **128.4** (ArC(4`````)H), **128.4** (Ar*C*(3,5)H), **128.7** (Ar*C*(4````)H), **128.8** (Ar*C*(3`````,5`````)H), **129.3** (Ar*C*(3````,5````)H), **137.2** (Ar*C*(1`````)), **140.4** (Ar*C*(1````)), **145.5** (Ar*C*(4)NO$_2$), **150.7** (*C*(4```)Ph$_2$), **153.8** (Ar*C*(1)O), **163.3** (ArO*C*(O)CH$_2$); HRMS (ESI$^+$) $C_{26}{}^{13}CH_{27}N_2O_4$ [M]$^+$: found 444.2008, required 444.1999 (+2.0 ppm).

1-(3,3-diphenylallyl-1-^{13}C)-1-(2-(4-nitrophenoxy)-2-oxoethyl-2-^{13}C)pyrrolidin-1-ium bromide 2,2'-[$^{13}C_2$]-2e

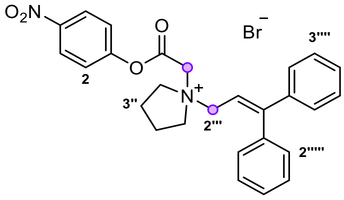

Following General Procedure **E**, 1-(3,3-diphenyl-1-allyl-1-^{13}C)pyrrolidine **S75** (78.4 mg, 300 µmol) and 4-nitrophenyl 2-bromoacetate-2-^{13}C **S67** (86.1 mg, 330 µmol) in MeCN gave the title compound as an off-white solid (119 mg, 75%) with data in agreement with **2e**;

mp 153 − 155 °C (dec.); ν_{max} (film): 3048, 2954, 1773, 1614, 1591, 1518, 1485, 1445, 1344, 1192, 1153; ^{1}H-NMR (500 MHz, DMSO-d6) δ_H: **1.91 – 2.19** (4H, m, C(3``,4``)*H*$_2$), **3.77 – 3.83** (4H, m, C(2``,5``)*H*$_2$), **4.31** (2H, dd, $^{1}J_{HC}$ = 147.0, $^{3}J_{HH}$ = 6.9, C(2```)*H*$_2$), **4.86** (2H, d, $^{1}J_{HC}$ = 147.0, ArOC(O)C*H*$_2$), **6.45** (1H, t, $^{3}J_{HH}$ = 6.9, C(3```)*H*), **7.12 – 7.18** (2H, m, ArC(4````)*H* and ArC(4`````)*H*), **7.33 − 7.42** (8H, m, ArC*H*), **7.45** (2H, d, $^{3}J_{HH}$ = 9.0, ArC(2,6)*H*), **8.36** (2H, d, $^{3}J_{HH}$ = 9.0, ArC(3,5)*H*); ^{13}C{^{1}H}-NMR (126 MHz, d^{6}-DMSO) δ_C: **21.9** (*C*(3``,4``)H$_2$), **58.2** (ArOC(O)*C*H$_2$), **59.1** (*C*(2```)H$_2$), **63.6** (*C*(2``,5``)H$_2$), **115.5** (*C*(3```)H), **122.9** (Ar*C*(2,6)H), **125.5** (Ar*C*(2`````, 6`````)H), **127.8** (Ar*C*(2````,6````)H), **128.4** (ArC(4`````)H), **128.4** (Ar*C*(3,5)H), **128.7** (Ar*C*(4````)H), **128.8** (Ar*C*(3`````,5`````)H), **129.3** (Ar*C*(3````,5````)H), **137.2** (Ar*C*(1`````)), **140.4** (Ar*C*(1````)), **145.5** (Ar*C*(4)NO$_2$), **150.7** (*C*(4```)Ph$_2$), **153.8** (Ar*C*(1)O), **163.3** (ArO*C*(O)CH$_2$, d, $^{1}J_{CC}$ = 64.0); HRMS (ESI$^+$) $C_{25}{}^{13}C_2H_{27}N_2O_4$ [M]$^+$: found 445.2020, required 445.2027 (−1.3 ppm).

(*R*)-*N*-Benzyl-5,5-diphenyl-2-(pyrrolidin-1-yl)pent-4-enamide-2,3-$^{13}C_2$ 2,3-[$^{13}C_2$]-3e

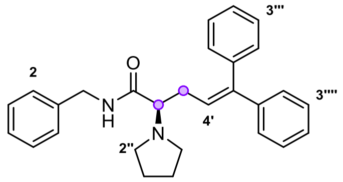

Following General Procedure **F**, (*S*)-tetramisole hydrochloride (7.2 mg, 0.03 mmol), triethylamine (33 µL, 0.24 mmol), 1-(3,3-diphenylallyl-1-^{13}C)-1-(2-(4-nitrophenoxy)-2-oxoethyl-2-^{13}C)pyrrolidin-1-ium bromide 2,2'-[$^{13}C_2$]-**2e** (78.8 mg, 0.15 mmol), benzylamine (25 µL, 0.225 mmol) in MeCN gave the crude product, which was purified by silica flash chromatography (CH_2Cl_2 : Et_2O 95:5 to 70:30) to give the title compound as a pale yellow solid (31 mg, 51%) with data in agreement with compound **3e**;

^{1}H-NMR (500 MHz, CDCl$_3$) δ_H: **1.64 – 1.76** (4H, m, C(3``,4``)*H*$_2$), **2.38 – 2.58** (5H, m, C(2``,5``)*H*$_2$ and C(3`)*H*$_A$H$_B$), **2.63 – 2.83** (1H, m, C(3`)H$_A$*H*$_B$), **2.99** (1H, appd, $^{1}J_{HC}$ = 136.7), **4.41** (1H, dd, $^{2}J_{HH}$ = 14.7, $^{3}J_{HH}$ = 5.6, NHC*H*$_A$H$_B$), **4.53** (1H, dd, $^{2}J_{HH}$ = 14.7, $^{3}J_{HH}$ = 6.2, NHC*H*$_A$H$_B$), **6.18** (1H, td, $^{3}J_{HH}$ = 6.9, $^{2}J_{HC}$ = 3.3, C(4`)H), **7.05 – 7.12** (1H, m, N*H*), **7.12 – 7.19** (4H, m, ArC*H*), **7.21 – 7.28** (8H, m, ArC*H*), **7.28 – 7.32** (1H, m, ArC*H*), **7.33 – 7.38** (2H, m, ArC*H*); ^{13}C{^{1}H}-NMR (126 MHz, CDCl$_3$) δ_C: **23.4** (*C*(3``,4``)H$_2$), **32.1** (*C*(3`)H$_2$, d, $^{1}J_{CC}$ = 34.6), **43.3** (NH*C*H$_2$Ph), **51.7** (*C*(2``,5``)H$_2$), **69.1** (*C*(2`)H, d, $^{1}J_{CC}$ = 34.5), **125.1** (*C*(4`)H), d, $^{2}J_{CC}$ = 44.6), **127.1** (Ar*C*(4```)H), **127.2** (Ar*C*(4````)H), **127.4** (Ar*C*(2,6)H), **127.5** (Ar*C*(4)H), **127.9** (Ar*C*(2```,6```)H), **128.2** (Ar*C*(2````,6````)H), **128.4** (Ar*C*(3,5)H), **128.8** (Ar*C*(3````,5````)H), **129.9** (Ar*C*(3```,5```)H) **138.6** (Ar*C*(1)), **139.9** (ArC(1````), d, $^{3}J_{CC}$ = 2.4), **142.6** (C(5`), d, $^{2}J_{CC}$ = 4.2), **143.3** (Ar*C*(1```), d, $^{3}J_{CC}$ = 3.4), **173.0** (*C*(1`)ONHBn, d, $^{1}J_{CC}$ = 53.5); HRMS (ESI$^+$) $C_{26}{}^{13}C_2H_{30}N_2O$ [M+H]$^+$: found 413.2478, required 413.2498 (–4.8 ppm).

D. 3. TEMPO adducts

(E)-2,2,6,6-tetramethyl-1-((3-phenylbut-2-en-1-yl)oxy)piperidine 5

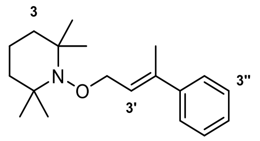

Following General Procedure **F**, (S)-tetramisole hydrochloride (9.6 mg, 0.04 mmol), triethylamine (45 µL, 0.32 mmol), (E)-1-(2-(4-nitrophenoxy)-2-oxoethyl)-1-(3-phenylbut-2-en-1-yl)pyrrolidin-1-ium bromide **2a** (92.3 mg, 0.2 mmol), TEMPO (31.3 mg, 0.2 mmol) and then benzylamine (45 µL, 0.3 mmol) in MeCN gave the crude product, which was purified by silica flash chromatography (Petroleum ether 40/60 : CH_2Cl_2 95:5 to 50:50) to give the title compound as a white solid (4.6 mg, 8%, 19:1 (E)/(Z) ratio);

mp. 86 – 87 °C (EtOAc); ν_{max} (film): 2974, 2930, 2870, 2847, 1558, 1495, 1472, 1447, 1373, 1360, 1261, 1132, 1022; ^{1}H-NMR (500 MHz, $CDCl_3$) δ_H: **1.12** (6H, s, 2 × CH_3), **1.23** (6H, s, 2 × CH_3), **1.30 – 1.37** (1H, m, C(4)H_{ax}H$_{eq}$), **1.45 – 1.51** (4H, m, C(3,5)H_2), **1.53 – 1.60** (1H, m, C(4)H$_{ax}$$H_{eq}$), **2.05** (3H, q, $^4J_{HH}$ = 1.3, C(4`)$H_3$), **4.50** (2H, d, $^3J_{HH}$ = 6.5, C(2`)H_2), **5.94** (1H, tq, $^3J_{HH}$ = 6.5, $^4J_{HH}$ = 1.3, C(3`)$H$), **7.19 – 7.26** (1H, m, ArC(4)$H$), **7.29 – 7.35** (1, m, ArC(2,6)$H$), **7.42** (1H, m, ArC(3,5)$H$); $^{13}$C{$^1$H}-NMR (126 MHz, $CDCl_3$) $\delta_C$: **16.5** ($C$(4`)H$_3$), **17.3** (C(4)H$_2$), **20.3** (2 × CH$_3$), **33.3** (2 × CH$_3$), **39.8** (C(3,5)H$_2$), **59.9** (2 × C(CH$_3$)$_2$), **75.1** (OCH$_2$), **124.1** (C(2`)H), **125.9** (ArC(2``,6``)H), **127.1** (ArC(4``)H), **128.3** (Ar$C$(3``,5``)H), **136.9** ($C$(3`)), **143.3** (ArC(1``)); HRMS (ESI$^+$) $C_{19}H_{29}NO$ [M+H]$^+$: found 288.2317, required 288.2322 (–1.7 ppm).

1-((3,3-diphenylallyl)oxy)-2,2,6,6-tetramethylpiperidine 6

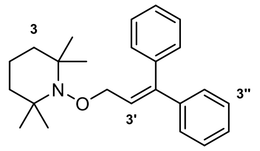

Following General Procedure **F**, (S)-tetramisole hydrochloride (9.6 mg, 0.04 mmol), triethylamine (45 µL, 0.32 mmol), 1-(3,3-diphenylallyl)-1-(2-(4-nitrophenoxy)-2-oxoethyl)pyrrolidin-1-ium bromide **2e** (105 mg, 0.2 mmol), TEMPO (31.3 mg, 0.2 mmol) and then benzylamine (45 µL, 0.3 mmol) in MeCN gave the crude product, which was purified by silica flash chromatography (Petroleum ether 40/60 : CH_2Cl_2 95:5 to 50:50) to give the title compound as a white solid (7.7 mg, 11%);

mp. 118 – 120 °C (CH_2Cl_2); ν_{max} (film): 3057, 3024, 2974, 2930, 2868, 1495, 1445, 1373, 1360, 1261, 1132, 1030; ^{1}H-NMR (400 MHz, $CDCl_3$) **1.09** (6H, s, 2 × CH_3), **1.12** (6H, s, 2 × CH_3), **1.20 – 1.35** (1H, m, C(4)H_{aq}H$_{eq}$), **1.36 – 1.45** (4H, m, C(3,5)H_2), **1.48 – 1.60** (1H, m, C(4)H$_{ax}$$H_{eq}$), **4.31** (2H, d, $^3J_{HH}$ = 6.9, C(2`)$H_2$), **6.23** (1H, t, $^3J_{HH}$ 7.0, C(3`)H), **7.18 – 7.22** (2H, m, ArCH), **7.23 – 7.29** (5H, m, ArCH), **7.28 – 7.37** (3H, m, ArCH); ^{13}C{^{1}H}-NMR (101 MHz, $CDCl_3$) δ_C: **17.3** (C(4)H$_2$), **20.3** (2 × CH$_3$), **33.1** (2 × CH$_3$), **39.8** (C(3,5)H$_2$), **59.8** (C(CH$_3$)$_2$), **75.4** (OCH$_2$), **124.8** (C(2`)H), **127.5** (Ar$C$H), **127.5** (Ar$C$H), **127.8** (Ar$C$H), **128.2** (Ar$C$H), **128.2** (Ar$C$H), **130.0** (Ar$C$H), **139.6** ($C$(3`)), **142.3** (ArC(1```)), **144.3** (ArC(1``)); HRMS (ESI$^+$) $C_{24}H_{31}NO$ [M+H]$^+$: found 350.2473, required 350.2478 (–1.4 ppm).

E. Crystallographic data

Compound **3a** (absolute configuration) – CCDC 2314746

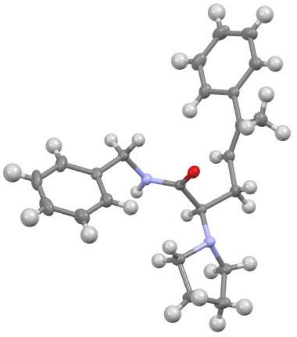

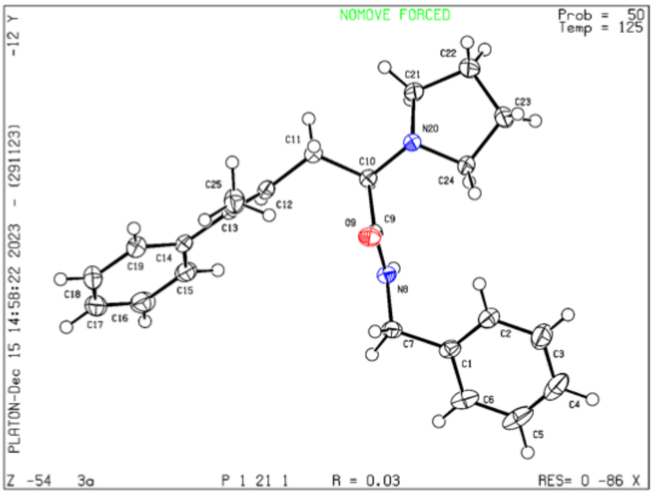

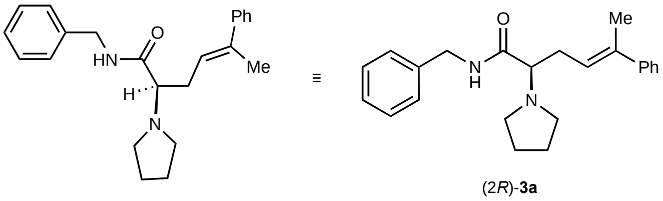

(2*R*)-**3a**

Empirical formula	$C_{23}H_{28}N_2O$
Formula weight	348.49
Temperature	125 K
Wavelength	0.71075 Å
Crystal system	monoclinic
Space group	$P2_1$
Cell lengths dimensions:	**a** 9.06740(9); **b** 8.82746(9); **c** 12.31500(13) Å
Cell angles dimensions	**α** 90.0000 **β** 93.2509(9) **γ** 90.0000
Volume:	984.132(17) $Å^3$
Z	2; Z'=1
Density (calculated)	1.176 g/cm^3
Absorption coefficient	0.094 mm^{-1}
F(000)	376
Crystal size	0.180 x 0.100 x 0.030 mm^3
Reflections collected	10857
Independent reflections	3577[R(int) = 0.0111]
Absorption correction	Multi-scan
Max. and min. transmission	0.851 and 0.983
Refinement method	Full-matrix least-squares on F^2
Goodness-of-fit on F^2	0.993
Final R indices [I>2sigma(I)]	R1 = 0.0348, wR2 = 0.0990
R indices (all data)	R1 = 0.0349, wR2 = 0.0990
Flack parameter	0.02(9)
Largest diff. peak and hole	0.34 and -0.26 $e^{-}.Å^{-3}$

Compound **4j** (major diastereoisomer – relative configuration) - CCDC 2312496

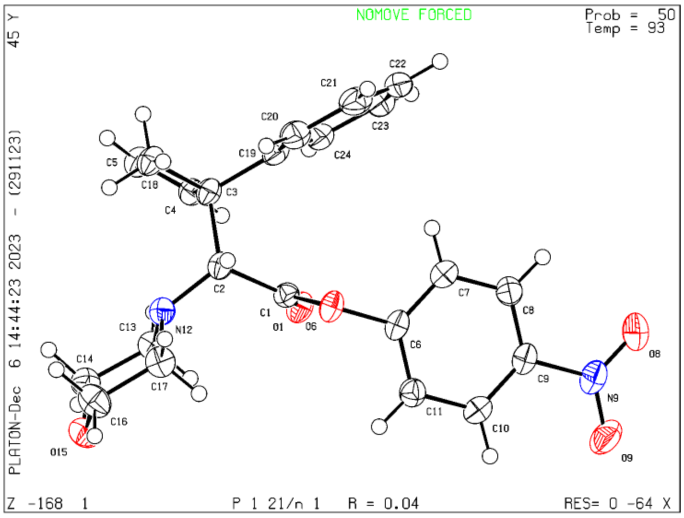

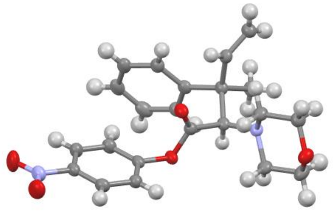

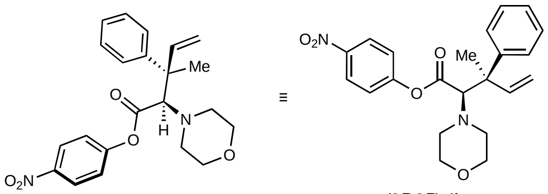

(2*R*,3*R*)-**4j**

Empirical formula	$C_{22}H_{24}N_2O_5$
Formula weight	396.44
Temperature	93K
Wavelength	0.71075 Å
Crystal system	monoclinic
Space group	P 2_1/n
Cell lengths dimensions:	**a** 6.6571(10); **b** 25.000(4); **c** 12.263(2) Å
Cell angles dimensions	**α** 90.0000 **β** 101.874(3) **γ** 90.0000
Volume:	1997.2(5) $Å^3$
Z	4; Z'=1
Density (calculated)	1.318 g/cm^3
Absorption coefficient	0.094 mm^{-1}
F(000)	840
Crystal size	0.100 x 0.050 x 0.050 mm^3
Reflections collected	21118
Independent reflections	3617[R(int) = 0.0610]
Absorption correction	Multi-scan
Max. and min. transmission	0.75 and 0.62
Refinement method	Full-matrix least-squares on F^2
Goodness-of-fit on F^2	1.062
Final R indices [I>2sigma(I)]	R1 = 0.0356, wR2 = 0.1169
R indices (all data)	R1 = 0.0426, wR2 = 0.1169
Largest diff. peak and hole	0.26 and -0.16 $e^-.Å^{-3}$

F. Mechanistic Studies

F. 1. Crossover experiments using ^{13}C-isotopic labelling

^{13}C isotopic labelling was chosen as a means of carrying out crossover experiments to quantify the intramolecularity of the reaction. The allylic ammonium salts proposed for this experiment were 2-[$^{13}C_1$]-**2a** and 2'-[$^{13}C_1$]-**2a**, which ensures a label either side of the breaking bond. In the event of intramolecular reaction, products 2-[$^{13}C_1$]-**3a** and 3-[$^{13}C_1$]-**3a** are formed, whereas intermolecular reaction would provide unlabelled product **3a** and the doubly labelled 2,3-[$^{13}C_2$]-**3a**. Quantitative ^{13}C-NMR was then used to measure the ratio of products.

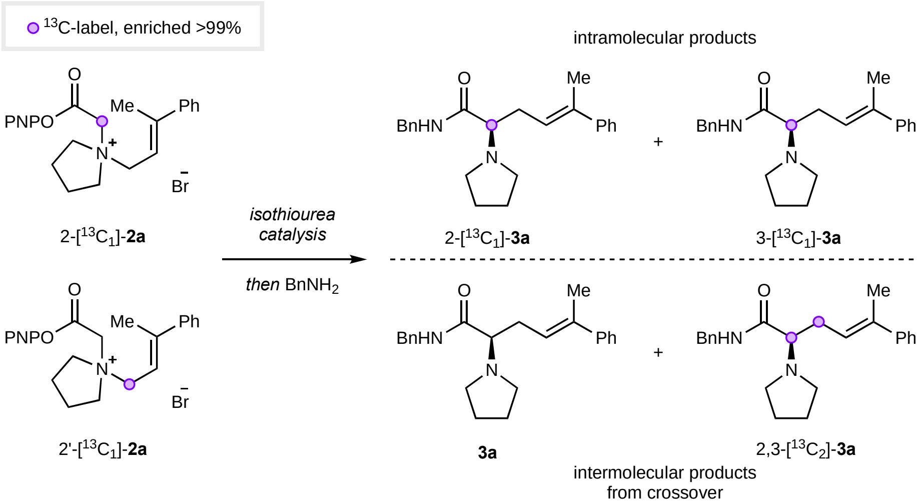

Figure S1: Crossover experiment of ^{13}C-labelled allylic ammonium salts in a [1,2]-rearrangement reaction and its possible outcomes**.**

F. 1. i. Measurement of ^{13}C NMR Relaxation Times

Integration of ^{13}C-NMR signals can be used to roughly determine the number of carbon atoms of the same hybridisation, but often gives unreliable values due to the low natural abundance of ^{13}C and varying relaxation times of carbon nuclei. Ensuring that all carbon atoms have relaxed to the ground state population distribution of spin states is important for acquiring NMR spectra that can be integrated quantitatively. Therefore, a series of NMR experiments was carried out to determine the relaxation times (T_1) of carbon nuclei in the model [1,2]-rearrangement product **3a**. Specifically, the T_1 of the C2 carbon was desired as this signal was to be used for integral analysis of crossover experiments. The T_1 of the C2 (69.0 ppm) carbon nuclei was determined to be 1.2 seconds (Table S4). The graphical solutions for which are depicted alongside Table S4, where the T_1 is the time for the value of net magnetisation to reach 63% of its maximum value (full relaxation). The longest measured T_1 (8.5 seconds) was for the quaternary C5-position. To ensure full relaxation for all quantitative ^{13}C NMR experiments, the delay time was extended to 43 seconds, over 5 times the longest T_1 measured.

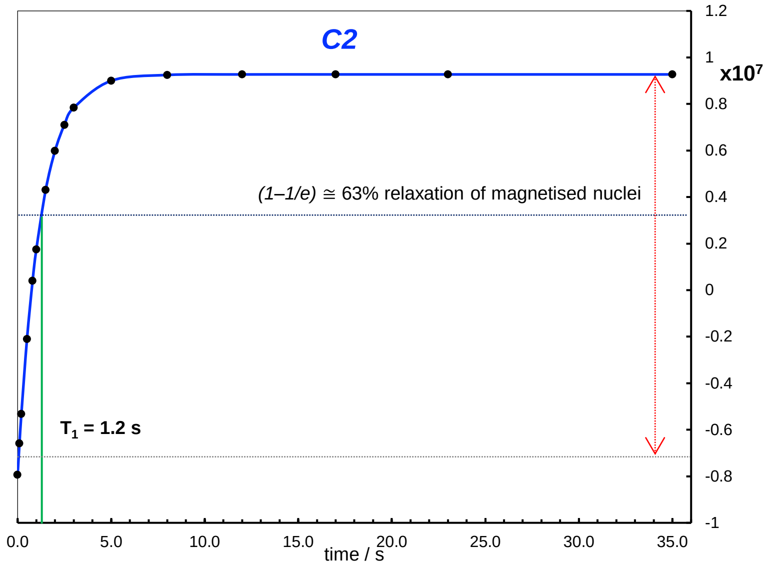

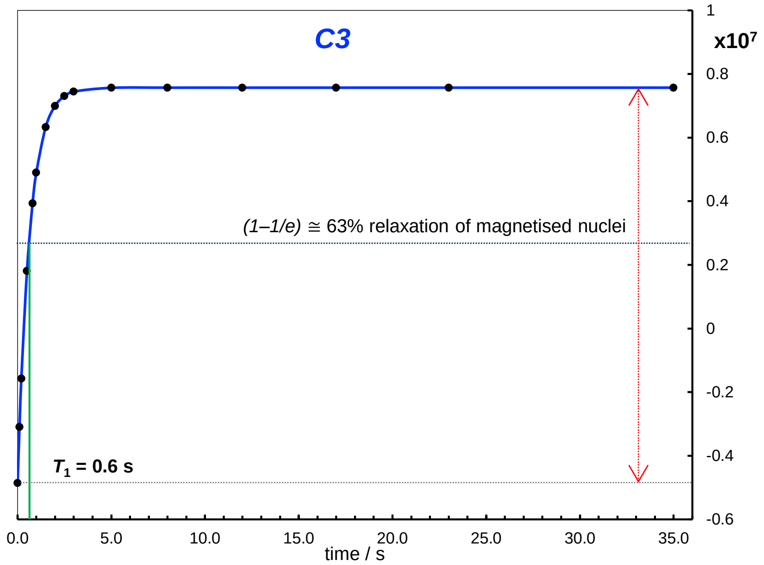

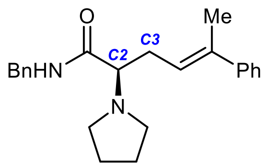

carbon	δ / ppm	T_1 / s
CO_2Ar	173.1	6.9
Ar*C* (quat.)	143.6	7.7
Ar*C* (quat.)	138.4	7.8
C (quat.)	136.8	8.5
ArCH	128.7	3.6
ArCH	128.2	2.5
ArCH	127.8	3.7
ArCH	127.3	1.9
ArCH	126.7	1.0
ArCH	125.7	2.6
*C*H	123.4	2.0
C2	**69.0**	**1.2**
CH_2	51.9	0.8
CH_2	43.2	1.3
C3	**31.2**	**0.6**
CH_2	23.4	0.9
CH_3	16.1	5.3

Table S4: ^{13}C-NMR pseudo 2D experiment to calculate the T_1 relaxation of all ^{13}C nuclei of **3a**.

F. 1. ii. Authentic Samples of Crossover Products

Authentic samples of all four possible products from the crossover experiment were synthesised and ^{13}C-NMR spectra were obtained (Figure S2). Each product was synthesised by applying the optimised reaction conditions to each of the labelled salts. The singly labelled [1,2]-rearrangement products are denoted 2-[$^{13}C_1$]-**3a** and 3-[$^{13}C_1$]-**3a**, the unlabelled [1,2]-rearrangement product is denoted **3a** and the doubly labelled product is denoted 2,3-[$^{13}C_2$]-**3a**. All four products were obtained in 72−74% yield and in 91:9 er. Each compound was characterised individually, and the C(2) signal from the ^{13}C-NMR spectra is given (Figure S2, right). Model [1,2]-rearrangement product **3a** exhibits a typical ^{13}C-NMR signal, in which there is no obvious ^{13}C doublet resulting from $^1J_{CC}$ coupling. The product arising from rearrangement of 2-[$^{13}C_1$]-**3a** displays an intense singlet C(2)-signal where there is isotopic enrichment, whereas 3-[$^{13}C_1$]-**3a** displays only a doublet due to the >99% ^{13}C-isotopic enrichment at the adjacent C(3)-position. Finally, doubly-labelled product 2,3-[$^{13}C_2$]-**3a** exhibits an intense C(2) doublet, with a measured coupling frequency ($^1J_{CC}$) of 34.6 Hz.

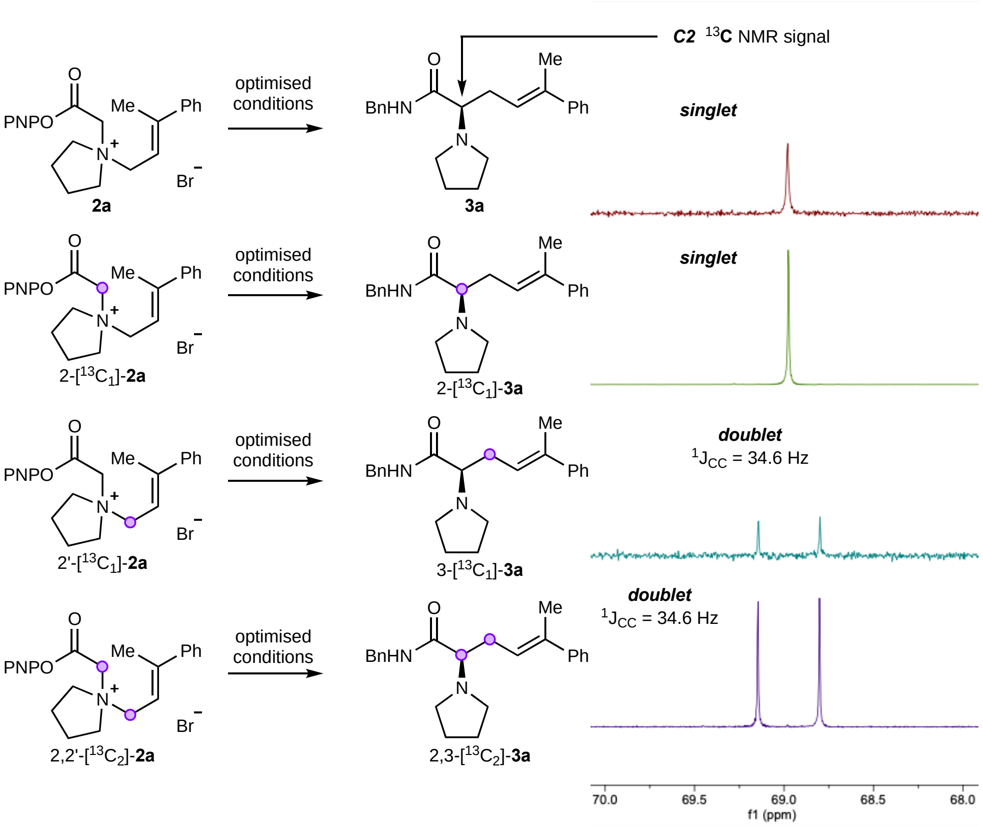

Figure S2: Intramolecular rearrangement of allylic ammonium salts **2a**, 2-[$^{13}C_1$]-**2a**, 2'-[$^{13}C_1$]-**2a** and 2,2'-[$^{13}C_1$]-**2a**, and the ^{13}C spectra of their [1,2]-rearrangment products focussed on the C(2) signal at δ_C = 69.0 ppm.

F. 1. iii. Calculation of Upper and Lower Limits of Crossover

As the C(3)-position ^{13}C-NMR signal sometimes overlapped with a minor unknown impurity, the C(2)-position signal was used for the quantitative crossover analysis. To determine crossover, the two extreme scenarios of a fully intramolecular and a fully intermolecular reaction were considered for the rearrangement of a 1:1 mixture of singly labelled ammonium salts 2-[$^{13}C_1$]-**2a** and 2`-[$^{13}C_1$]-**2a**.

Fully Intramolecular Reaction: The Lower Limit of Crossover

In a fully intramolecular reaction there will be only two [1,2]-rearrangement products, 2-[$^{13}C_1$]-**3a** and 3-[$^{13}C_1$]-**3a,** formed in a 1:1 ratio, and each arising only from one of salts 2-[$^{13}C_1$]-**2a** and 2`-[$^{13}C_1$]-**2a**. However, due to the natural abundance of ^{13}C (1.1%), there will be a proportion of the rearrangement product that has a ^{13}C-^{13}C bond, giving rise to a doublet in the NMR spectrum. If we firstly consider rearrangement of 2-[$^{13}C_1$]-**2a** the two expected products will either contain a ^{13}C at both C(2) and C(3) {2,3-[$^{13}C_2$]-**3a**} or a ^{13}C at C(2) and ^{12}C at C(3) {2-[$^{13}C_1$]-**3a**}. If we consider that salt 2-[$^{13}C_1$]-**2a** is converted to the [1,2]-rearrangement product, the predicted proportion of the [1,2]-rearrangement products that will be 2-[$^{13}C_1$]-**3a** is 98.9%. The remainder of the [1,2]-rearrangement product will be 2,3-[$^{13}C_2$]-**3a** expected in 1.1% (Figure S3). Product 2-[$^{13}C_1$]-**3a** will contribute a singlet to the NMR signal, and 2,3-[$^{13}C_2$]-**3a** will contribute a doublet (^{13}C-^{13}C).

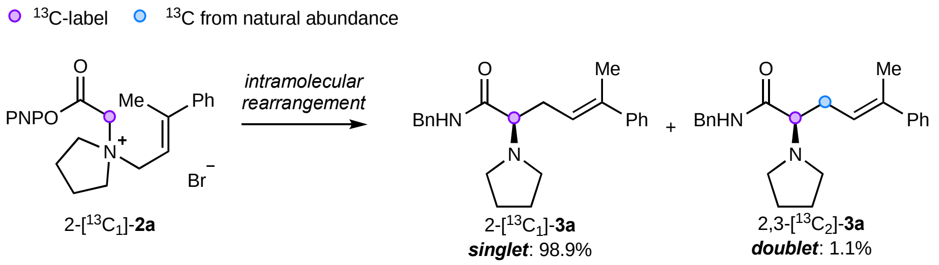

Figure S3: Hypothetical fully intramolecular [1,2]-rearrangement of 2-[$^{13}C_1$]-**2a**.

In the crossover experiment there will be a 1:1 mixture of two unique salts. Therefore, assuming parallel intramolecular rearrangements, the products arising from each salt can be separately rationalised. Rearrangement of 2-[$^{13}C_1$]-**2a** salt will contribute 50% of the total [1,2]-rearrangement product and in the ^{13}C-NMR spectrum this divides between 49.45% as the 2-[$^{13}C_1$]-**3a** singlet, and 0.55% to the doublet 2,3-[$^{13}C_2$]-**3a** NMR signal (Figure S4). As for the other salt, 2'-[$^{13}C_1$]-**2a** will react to give the same ratio of products, but in this case 2,3-[$^{13}C_2$]-**3a** contributes a doublet (0.55%) and 3-[$^{13}C_1$]-**3a** contributes nothing as there will be no ^{13}C at the C(2)-position. Overall, the signal at the C(2)-position will account for 50.55% of the total product, with the doublet constituting 2.2% of the signal. This means that when the singlet and the doublet of the C(2) signal are integrated, the ratio will be 97.8:2.2 singlet/doublet when the reaction is fully intramolecular, where 2.2% doublet represents the lower limit for intermolecularity.

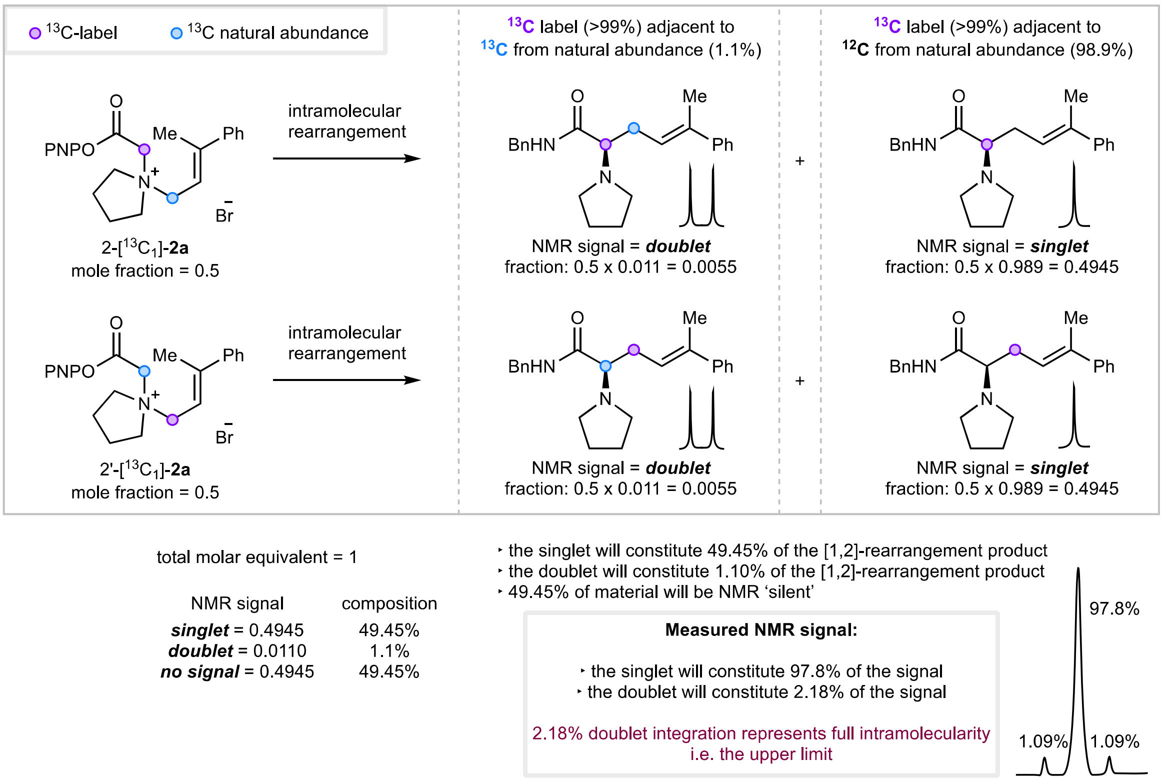

Figure S4: Expected ^{13}C NMR signal at the C2 position for a hypothetical fully intramolecular reaction.

Fully Intermolecular Reaction: The Upper Limit of Crossover

The other extreme scenario is the fully intermolecular reaction, where each ammonium ylide is assumed to fully dissociate and recombine to give a statistical mixture of products. This process is assumed to only take place after addition of isothiourea and subsequent deprotonation to form an allylic ammonium

ylide, as reaction under the optimised conditions in the absence of catalyst does not give rearrangement products. Dissociation of each intermediate will produce three fragments: for example, ammonium salt 2-[$^{13}C_1$]-**2a** will generate an equimolar amount of labelled acyl ammonium **A$_{(lab)}$**, and two distinct allylic fragments **B** (Figure S5). One will contain a ^{13}C at C(2) due to natural abundance (**B$_{(nat)}$**) and the other is NMR silent (**B$_{(0)}$**) as it will contain only ^{12}C (where '**lab**' refers to a ^{13}C-labelled component, '**nat**' refers to a ^{13}C containing component due to natural abundance, and '**0**' signifies total absence of ^{13}C). The total amount of each component in the reaction will depend on the mole fraction of salt used, which will be 0.5 for each of the two labelled salts in the crossover experiment. Therefore upon full dissociation of 2-[$^{13}C_1$]-**2a** there will be 50% of **A$_{(lab)}$**, 49.45% of **B$_{(0)}$** and 0.55% of **B$_{(nat)}$** generated (Figure S5, top). The same will be true of 2'-[$^{13}C_1$]-**2a**, which will form the allylic fragment **B$_{(lab)}$** in 50%, and two distinct acyl ammonium species **A$_{(0)}$** in 49.45% and **A$_{(nat)}$** in 0.55%.

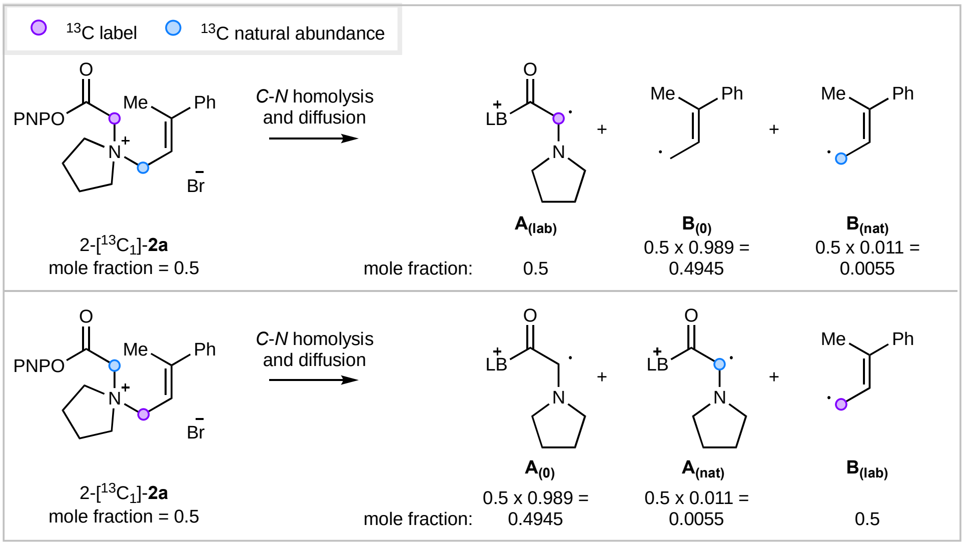

Figure S5: C-N homolytic cleavage followed by full separation of radicals through diffusion out of the solvent cage.

The six components can then form the [1,2]-rearrangement product through **A** + **B** recombination only, and the ratio of products will depend on the mole fraction of each component. The nine possible products are given in below in Figure S6, with the percentage of total [1,2]-rearrangement product calculated by multiplying the mole fractions of the corresponding components. In this scenario, the doublet 2,3-[$^{13}C_2$]-**3a**, will constitute 50.55% of the C(2) NMR signal, and the singlet 2-[$^{13}C_1$]-**3a** will constitute 49.45% of the signal. These values represent the upper limit of intermolecularity.

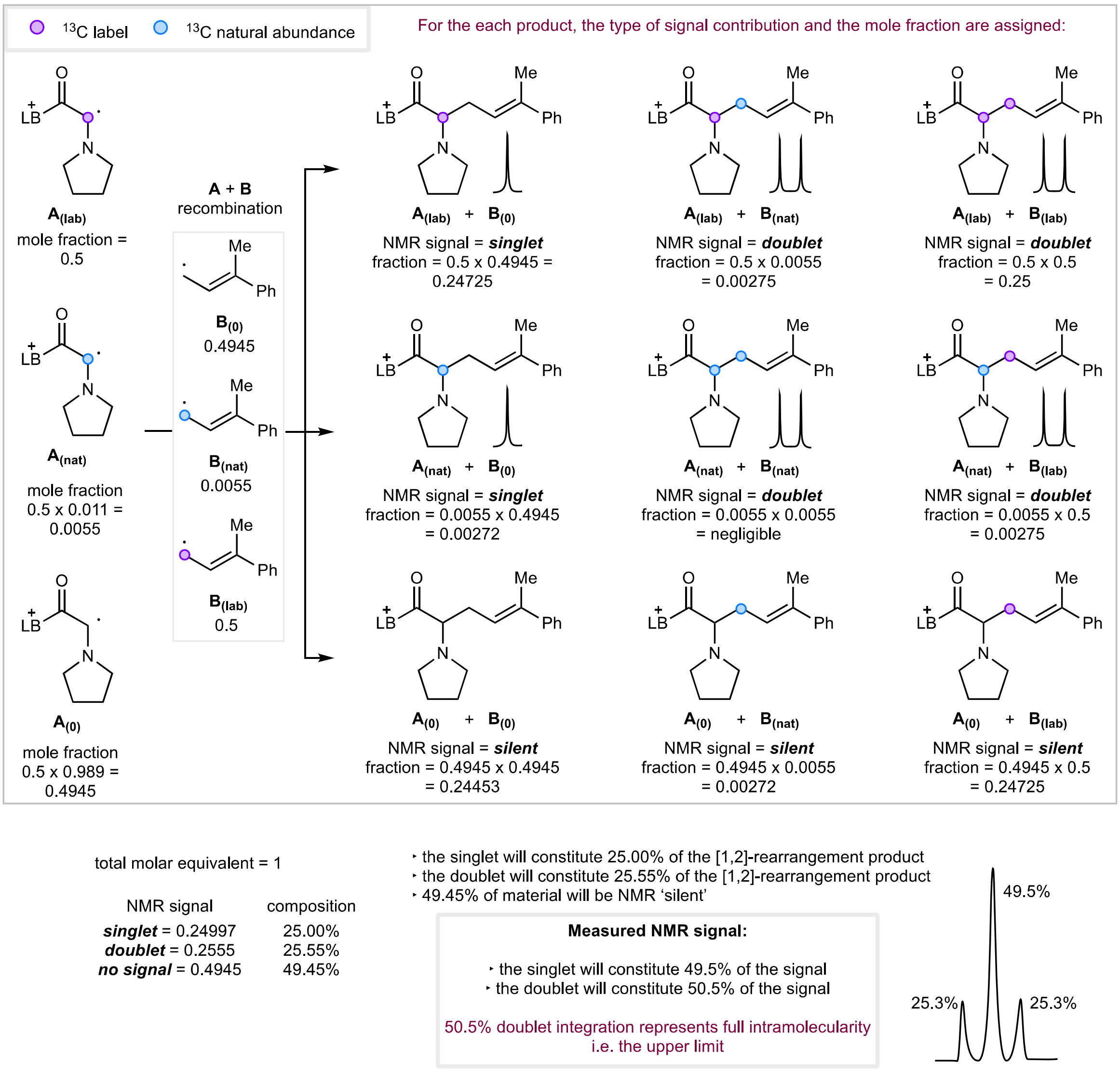

Figure S6: Breakdown of the expected ^{13}C NMR signal at the C2 position for a hypothetical fully intermolecular reaction.

With the upper and lower limits for intermolecularity established, it is possible to integrate the singlet and doublet of the C(2) NMR signal to determine the level of intermolecularity for the [1,2]-rearrangement. The values of the upper and lower limits were plotted as the percentage of the total δ_C 69.0 ppm signal integration that is doublet (upper limit = 50.55%, lower limit = 2.2%), and a straight line was fitted between these two points. The graphical solution (Figure S7) leads to the following equation:

$$y = 0.4837x + 2.18$$

where y is the percentage of the C(2) NMR signal that is doublet, and x is intermolecularity (i.e. percentage of products arising from intermolecular reaction). This equation can be used to determine intermolecularity.

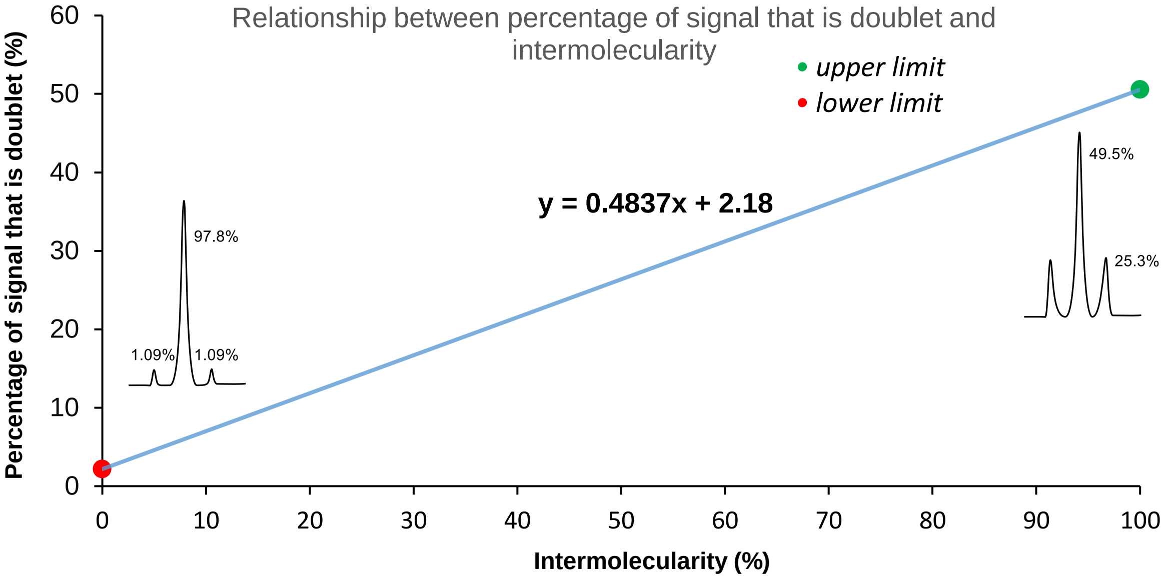

Figure S7: Graphical solution to determination of intermolecularity using the ratio of singlet to doublet of the C(2) NMR signal.

F. 1. iv. [1,2]-Rearrangement crossover experiments

The crossover experiments were conducted under the optimised conditions, using a 1:1 mixture of 2-[$^{13}C_1$]-**2a** and 2'-[$^{13}C_1$]-**2a**. Integrations were consistently measured using Mestrenova's deconvoluting 'peaks' method, providing integrals from simulated peaks independent of integration width. The values for intermolecularity are averages, as each experiment was repeated and quantitative ^{13}C-NMR analysis was carried out twice on each sample. An exemplification of the analysis is depicted in Table S5. Integral ranges were applied and the total integral value set to 100. Integral values for the doublet signal were combined, 2.18 was subtracted and the total divided by 0.4873 as set out in equation **1**. The calculated intermolecularity is given below each spectrum, with the average from four spectra being 13.5%, and the calculated standard deviation of 0.3 reflecting the reliability of these results.

a)

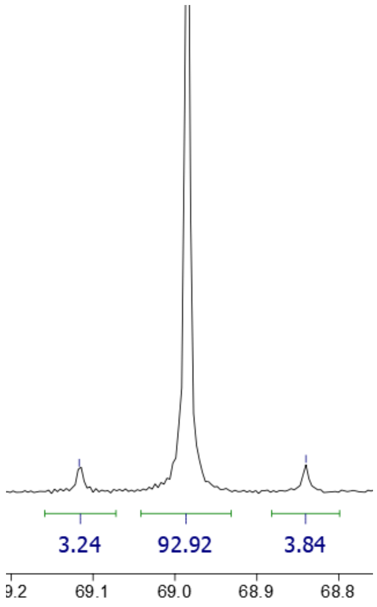

Experiment 1: 1st $^{13}C\{^{1}H\}$-NMR (d1 = 43 s)

Total integral = 100

Calculated intermolecularity = ((**3.24**+**3.84**) – 2.18) / 0.4873 = **10.1%**

b)

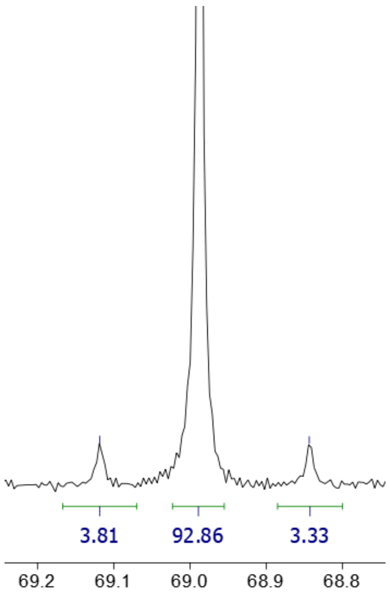

Experiment 1: 2nd $^{13}C\{^{1}H\}$-NMR (d1 = 43 s)

Total integral = 100

Calculated intermolecularity = ((**3.81**+**3.33**) – 2.18) / 0.4873 = **10.2%**

c)

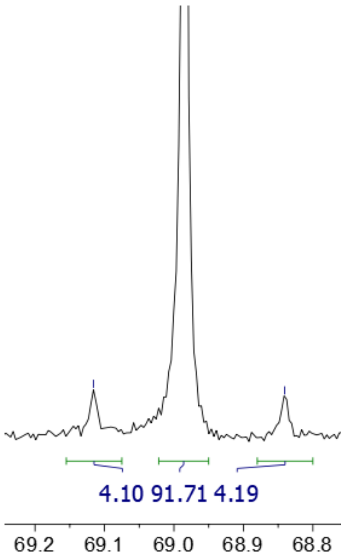

Experiment 2: 1st $^{13}C\{^{1}H\}$-NMR (d1 = 43 s)

Total integral = 100

Calculated intermolecularity = ((**4.10**+**4.19**) – 2.18) / 0.4873 = **12.5%**

d)

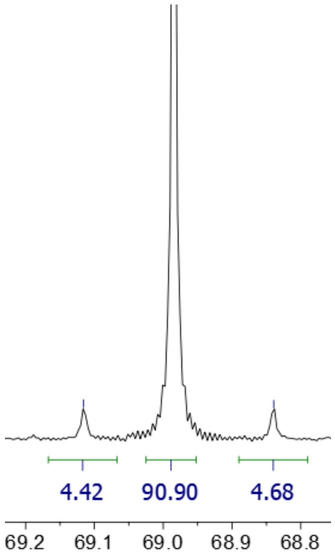

Experiment 2: 2nd $^{13}C\{^{1}H\}$-NMR (d1 = 43 s)

Total integral = 100

Calculated intermolecularity = ((**4.42**+**4.68**) – 2.18) / 0.4873 = **14.2%**

Average calculated intermolecularity = **11.8%**; margin of error (95% confidence level) = **1.7%**

Table S5: Demonstration of analyses performed on the quantitative ^{13}C-NMR spectra of the [1,2]-rearrangement product obtained from the crossover reaction. . a) and b) are spectra of two separate NMR experiments carried out on the same sample of [1,2]-rearrangement product (from a single crossover experiment). c) and d) are spectra from a repeat crossover experiment.

The [1,2]-rearrangement of ammonium salt **2a** bearing Me/Ph terminal substitution proceeded with an intermolecularity of 11.8 ± 1.7%, indicating a mostly intramolecular reaction. Using ethylene carbonate

as solvent, the [1,2]-rearrangement product was obtained with lower crossover than with MeCN (3.9% intermolecularity). Diphenyl substituted ammonium salt **2e** underwent [1,2]-rearrangement with an intermolecularity of 32.0%, and in 77:23 er. In the presence of TEMPO (1 equiv.), the [1,2]-rearrangement product was formed in an almost total intramolecular process, with enhanced ennaitoselectivity (85:15 er).

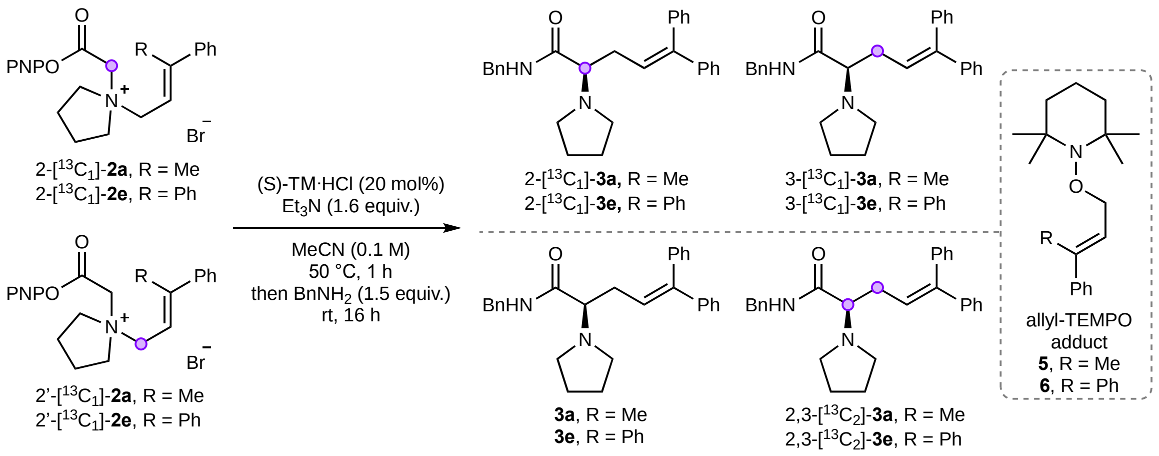

entry	R	deviation from standard conditions	yield **3** [1,2] [%]	intermolecularity	e.r. of **3**	yield **5** or **6** [%]
1	Me	-	72	11.8% ± 1.7	91:9	n/a
2	Me	Ethylene carbonate as solvent (0.1 M)	69	3.9% ± 0.4	94:6	n/a
3	Me	TEMPO (1 equiv.)	30	0.9% ± 0.2	97:3	8%
4	Ph	-	62	32.0% ± 0.6	77:23	n/a
5	Ph	TEMPO (1 equiv.)	46	0.4% ± 0.1	86:14	11%

Table S6: Crossover experiments for the [1,2]-rearrangment. Errors are one standard deviation.

F. 1. v. Determination of crossover within the enantiomeric products 3e

A sample of product **3e**, obtained from a crossover experiment between 2-[$^{13}C_1$]-**2e** and 2'-[$^{13}C_1$]-**2e**, was analysed using chiral HPLC and ^{13}C NMR. The 71:29 er sample, with 32% intermolecularity, was separated using preparative chiral HPLC (REACH chiral solutions, https://reachseparations.com/) to give both the (*R*)-**3e** and (*S*)-**3e** products in enantiopure form. The separated enantiomers were subjected to ^{13}C NMR analysis to determine the degree of intermolecularity. With the enantiomeric ratio of the reaction and the degree of intermolecularity within the individual enantiosemers, it is possible to calculate enantiomeric ratios of the two processes that lead to product formation: the intermolecular reaction and the intramolecular reaction.

For the intermolecular process (32.0%), the enantiomeric ratio is calculated to be 78:22.

For the intramolecular process (68.0%), the enantiomeric ratio is calculated to be 59:41.

This result supports our hypothesis that in-cage radical recombination (which gives the 'intramolecular product') is more enantioselective than the recombination of diffused radicals (intermolecular).

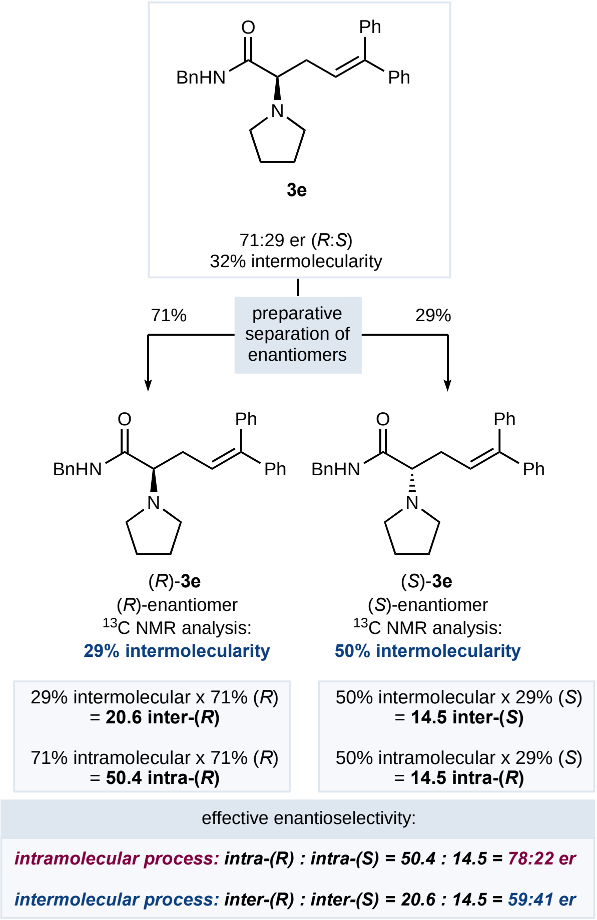

Figure S8: Calculation of enantioselectivity for both intramolecular and intermolecular formation of the [1,2]-rearrangement product **3e**.

This analysis was repeated for the crossover experiment carried out in the presence of TEMPO. The 84:16 er sample of 0.4% intermolecularity was separated into its individual enantiomers by preparative chiral HPLC.

For the intermolecular process (0.4%), the enantiomeric ratio is calculated to be 44:56.

For the intramolecular process (99.6%), the enantiomeric ratio is calculated to be 84:16.

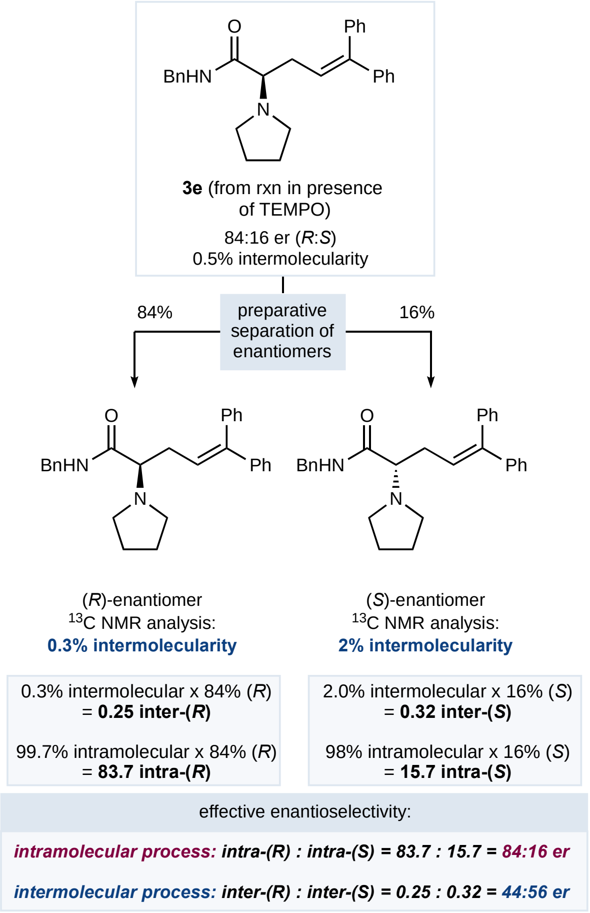

Figure S9. Calculation of enantioselectivity for both intramolecular and intermolecular formation of the [1,2]-rearrangement product **3e** in the presence of the radical scavenger TEMPO.

F. 1. vi. Ammonium salt crossover experiments

Bromide-promoted alkyl exchange was probed by heating two different ammonium salts at 50 °C in deuterated MeCN. Use of ^{1}H and ^{19}F NMR revealed no exchange took place after 16 h, indicating both thermal stability at the optimised reaction temperature and the likely innocence of bromide in promoting crossover in the absence of a base (Figure S10). This is consistent with the absence of halide-mediated exchange of allylic aliphatic ammonium salt substituents under similar reaction conditions reported in the literature (MeCN, 60 °C), where only anilinium-based salts underwent exchange.[23]

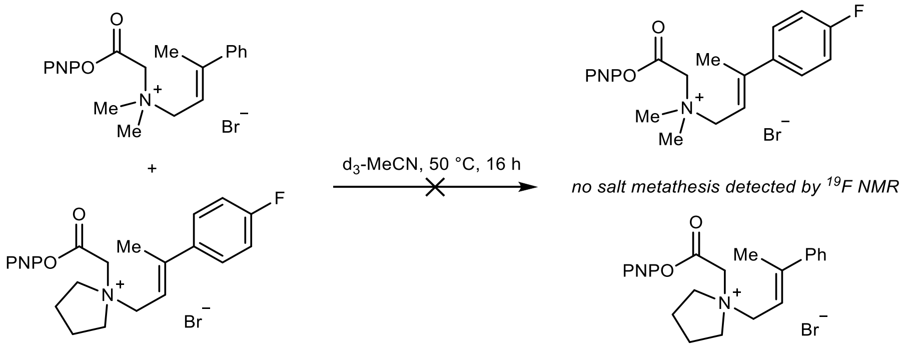

Figure S10: Salt metathesis experiment in the absence of base.

To probe the possibility of alkyl exchange under the reaction conditions, ammonium salt **2a** was subjected to 1 equivalent of a fluorine-labelled allylic tertiary amine **S55** in the presence of tetramisole hydrochloride and triethylamine at 50 °C in MeCN (Figure S11). ^{1}H and ^{19}F NMR analysis of the crude reaction mixture revealed the reaction was largely unaffected, although a small decrease in yield of rearrangement products was observed. There were a few minor unknown ^{19}F signals, but most of the tertiary amine **S55** remained (>95%). Importantly, the ^{19}F NMR signal that characterises [1,2]-rearrangement product **3n** was not detected in the crude reaction mixture. Further NMR analysis of the purified [1,2]-rearrangement product revealed no incorporation of **S55**.

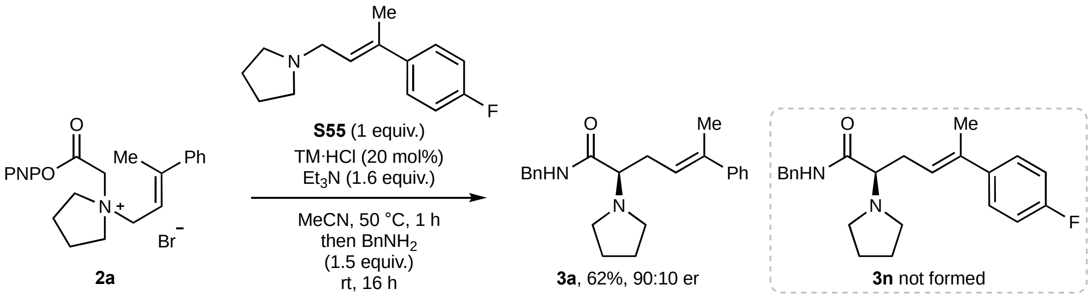

Figure S11: Salt metathesis experiment with external base.

F. 1. vii. Crossover of the Competitive [2,3]-Rearrangement

While some [2,3]-rearrangement is observed, it is not clear whether this product is formed via the thermally-allowed concerted mechanism, by the radical pair recombination mechanism, or a combination of both. To probe this, allylic ammonium salt 4'-[$^{13}C_1$]-**2a** was synthesised bearing a ^{13}C-label at the C4'-position (Figure S12). Under the optimised reaction conditions, the major diastereoisomer of the [2,3]-rearrangement product **4a** was isolated in 11% yield from treatment of a 1:1 mixture of 2-[$^{13}C_1$]-**2a** and 4'-[$^{13}C_1$]-**2a**. Analysis revealed only 2.6% intermolecular reaction had taken place, considerably less than that observed for the [1,2]-rearrangement product (11.8%).

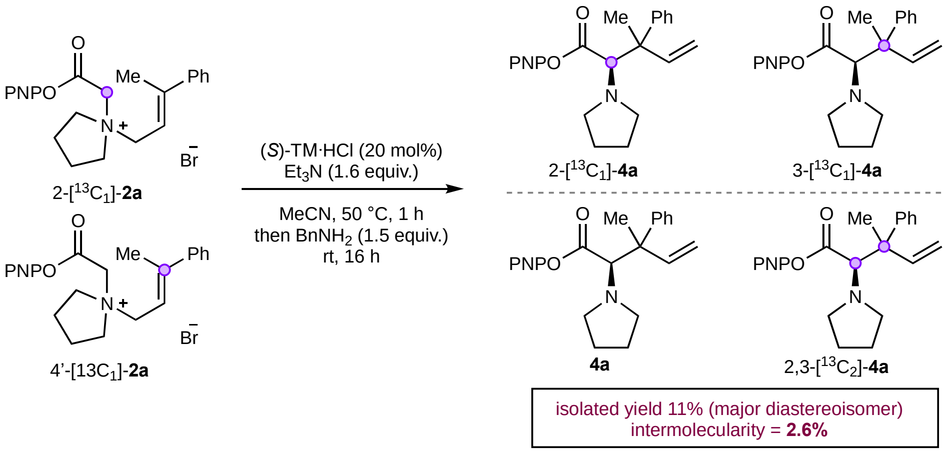

Figure S12: Crossover experiment for the [2,3]-rearrangement.

F. 1. viii. In situ reaction monitoring of 2i

A solution of **2i** (30.4 mg, 0.07 mmol) in d^3-MeCN (0.1 M) was prepared in an NMR tube and placed in an NMR spectrometer for calibration. The reaction was initiated by addition of 100 µL of a stock solution of tetramisole hydrochloride (0.014 mmol) and triethylamine (0.112 mmol) in d^3-MeCN and ^{1}H NMR measurements were taken periodically.

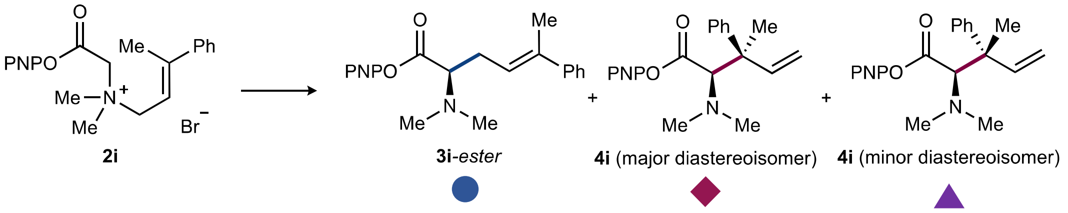

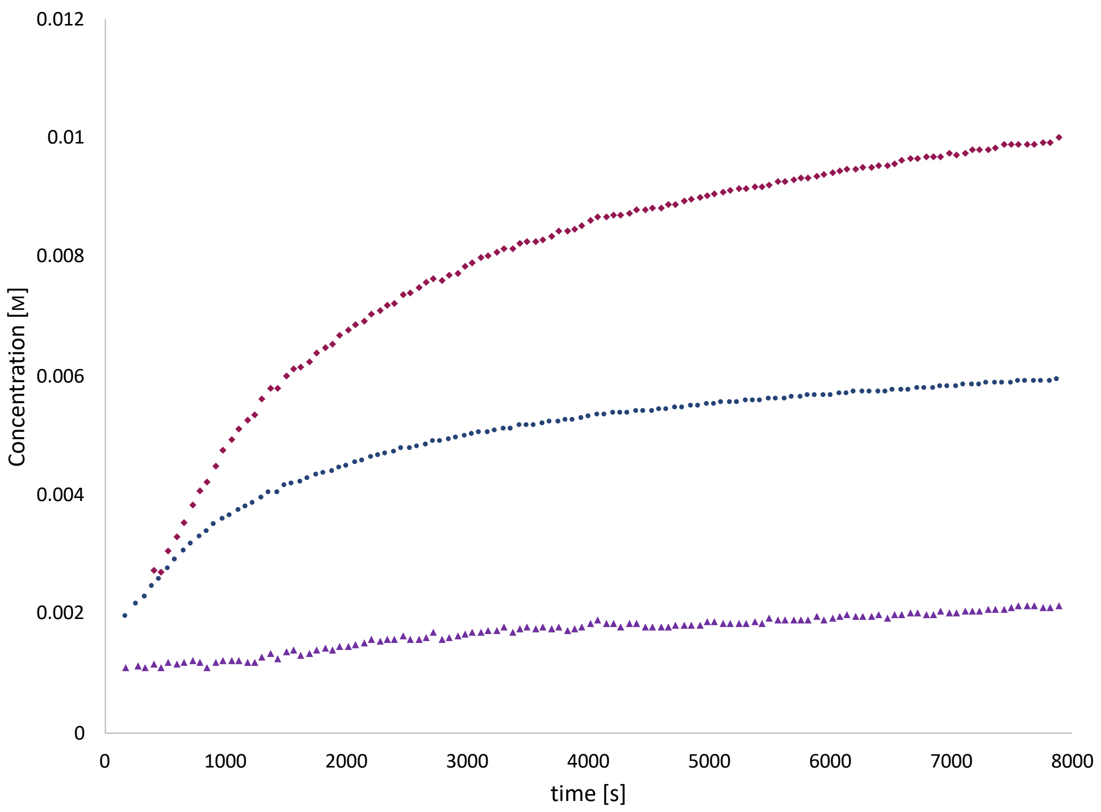

F. 2. EPR spectroscopy

F. 2. i. Detection of Radical Intermediates

To probe the potential detection of radical intermediates, *in situ* EPR (electron paramagnetic resonance) spectroscopic experiments were undertaken. Diphenyl-substituted salt **2e** was chosen for these studies in order to maximise the probability of radical detection, since this substrate gives the largest degree of intermolcularity. Firstly, background correction was carried out on a sample tube containing the diphenyl-substituted salt **2e**, tetramisole hydrochloride catalyst (20 mol%) and MeCN (0.1 M). Unfortunately, MeCN absorbs strongly in the microwave region that is applied, and therefore background noise correction rendered the detection of radicals difficult. In addition, dissolution of the solid starting materials was poor.

F. 2. ii. Observing the Consumption of TEMPO

An EPR sample tube was charged with ammonium salt **2e** (100 mM, 1 equiv.), tetramisole hydrochloride (20 mM, 0.2 equiv.) and TEMPO (10 mM, 0.1 equiv.) in anhydrous acetonitrile (heterogenous mixture), while the spectrometer sample holder was heated to 50 °C. An EPR spectrum was recorded (Figure S13, left, black line). After addition of Et_3N (160 mM, 1.6 equiv.), EPR spectra were recorded every 5 seconds to obtain a temporal profile of TEMPO concentration (Figure S13, right).[23] After 3 minutes reaction time, TEMPO (0.1 equiv.) was fully consumed.

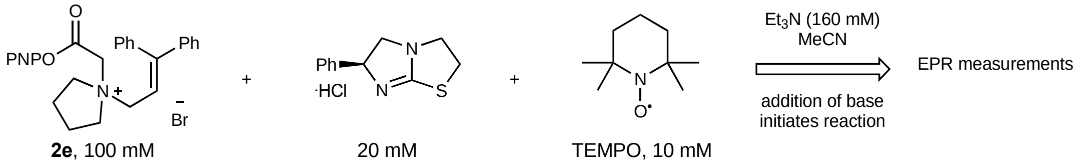

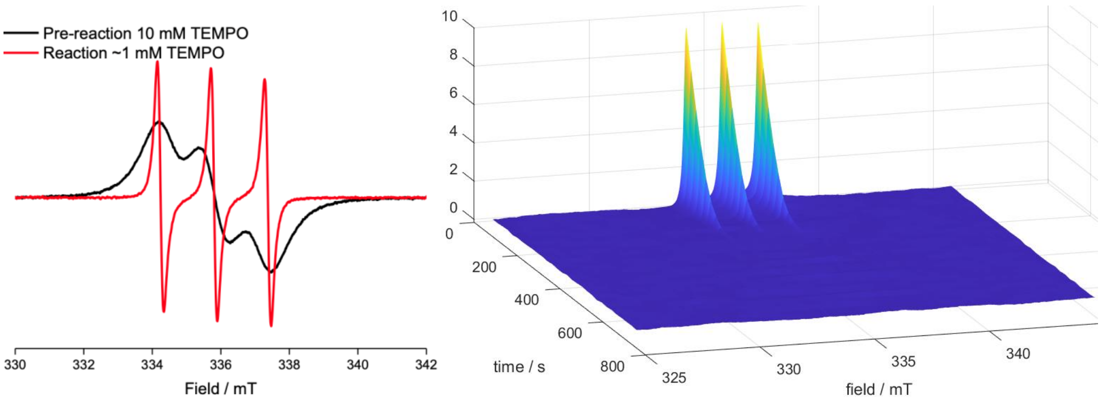

Figure S13: a) Example of the EPR signal of the aminoxyl radical of TEMPO. b) Decay of TEMPO EPR triplet over time under the reaction conditions of the [1,2]-rearrangement of **2e**.

F. 2. iii. EPR in Flow

To negate issues with solubility and background noise, an alternative approach using a flow reactor was proposed. A constant solvent flow allows for a much smaller diameter of sample tube to be used in the spectrometer during the experiment, which reduces the problems associated with microwave absorption. A 'standard' reaction (without TEMPO) was set-up using a stirrer-hotplate, with a 2 mm diameter plastic tube submerged in the reaction vessel (Figure S14). A peristaltic pump was used to operate the flow of solvent, and the tubing was passed through an EPR sample tube that was placed in the spectrometer. A five-fold lower concentration of reagents (0.02 M with respect to ammonium salt

2e) was used to mitigate blockages in the flow tubing. No radical intermediates were detected using this set-up.

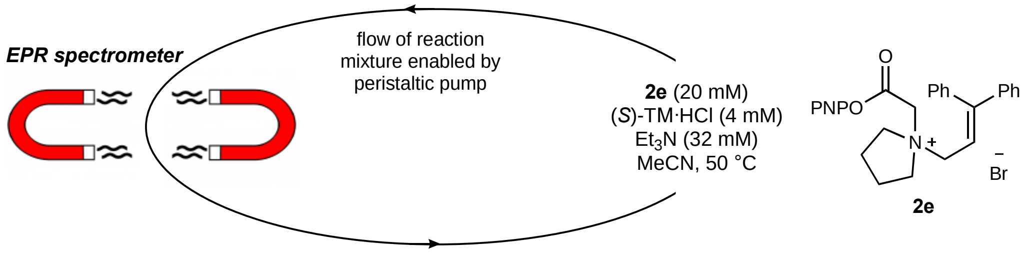

Figure S14: EPR using a flow set-up.

Next, the consumption of TEMPO was monitored over time using the flow reactor set-up (Figure S15). The relative amount of TEMPO present in the reaction was altered to 0.5 equivalents. During this reaction the concentration of TEMPO decreased by approximately 20%, corresponding to a loss of ~0.1 equivalents. This is consistent with the formation of allyl-TEMPO adduct **6** in 11% yield, which can be attributed to radical-radical coupling. Therefore, TEMPO efficiently traps the diffused allyl radical in the reaction which could otherwise have reacted intermolecularly.

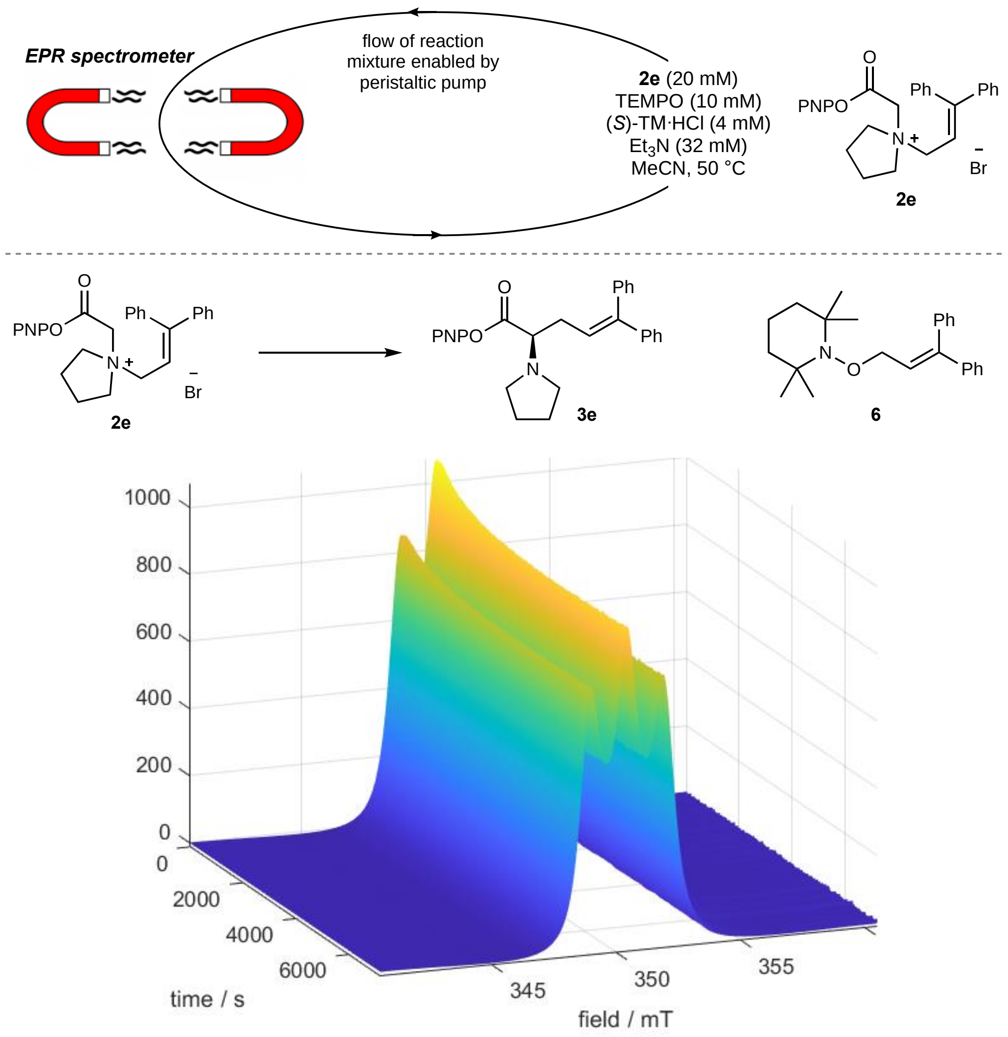

Figure S15: EPR monitoring of TEMPO concentration during the [1,2]-rearrangement of **2e**.

It was deemed important to deduce whether this process was distinct to the [1,2]-rearrangement. The proposed competitive [2,3]-rearrangement process has a much greater degree of intramolecularity than the [1,2]-rearrangement. The experiment was repeated using an allylic ammonium salt **2x** with only monosubstitution at the terminal position, which undergoes exclusive [2,3]-rearrangement (Figure S16). The concentration of TEMPO remained constant during the reaction, demonstrating that the consumption of TEMPO is only operative during the [1,2]-rearrangement. This leads to the conclusion that TEMPO is not interfering with the intramolecular process but reacting only with the diffused allyl radical to prevent intermolecular reaction.

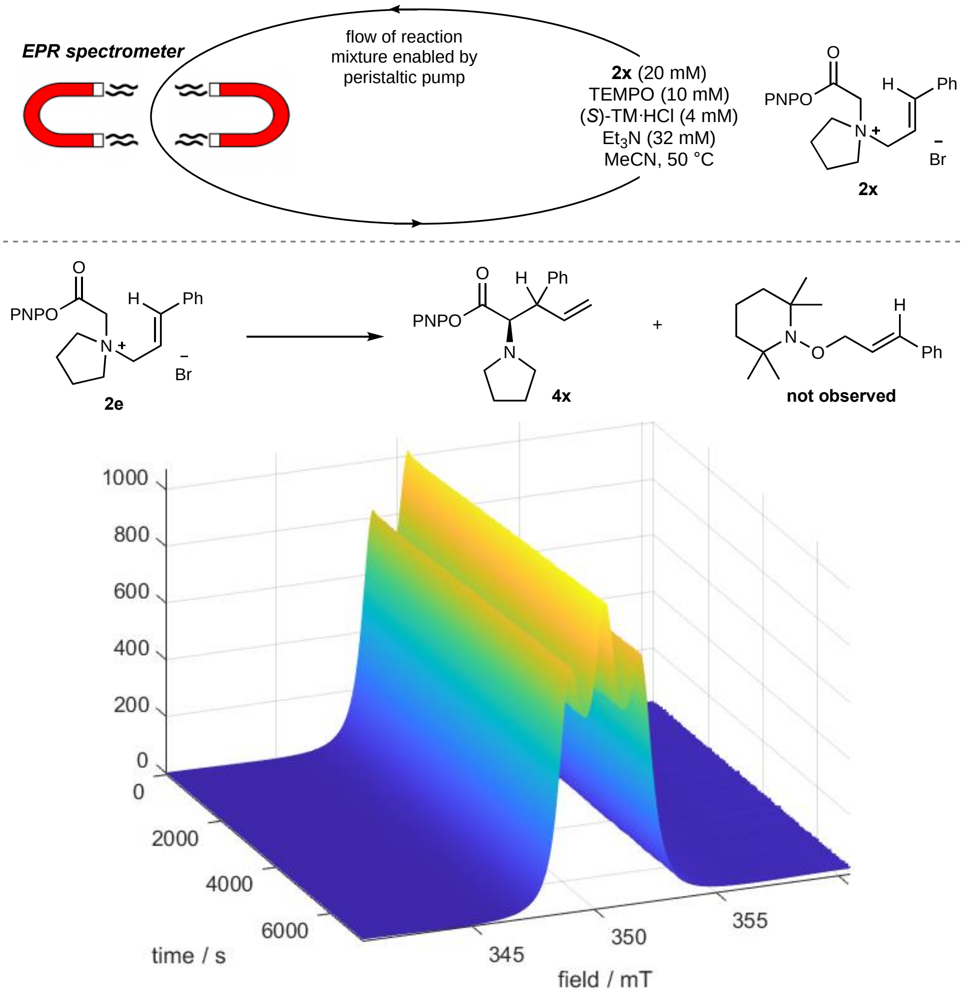

Figure S16: EPR monitoring of the concentration of TEMPO in the [2,3]-rearrangement of **2x**.

F. 3. Product Isomerisation

A sample of the major diastereoisomer of the [2,3]-rearrangement product **4a** was subjected to the catalytic reaction conditions in an NMR tube for 16 hours, after which it was analysed (Figure **S17**). No [1,2]-rearrangement product was formed, indicating that a [2,3]- to [1,2]- product isomerisation is unlikely to be a productive pathway for product formation under the reaction conditions. From the diastereomerically-pure [2,3]-rearrangement product **4a** (>99:1 dr), a mixture of diastereoisomers had formed (85:15 dr), confirming that the minor diastereoisomer can form from the major diastereoisomer of the [2,3]-product in the reaction. As the C(3)-position is quaternary, this is proposed to occur through epimerisation at the C(2)-position.

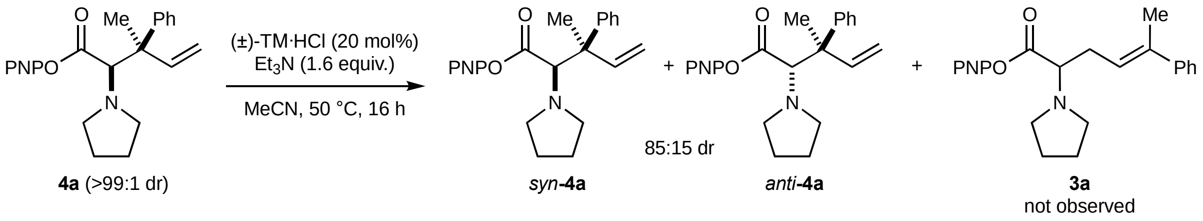

Figure S17: Subjection of the [2,3]-rearrangement product major diastereoisomer to the standard reaction conditions.

G. Solvent properties

entry	Solvent	Density at 20 °C [g/cm³]	Density at 50 °C [g/cm³]	Viscosity at 40 °C [Pa·s]	Dielectric constant [F/m]
1	MeCN	0.782[25]	0.749[25]	0.298[26]	37.50[27]
2[a]	EC		1.305[28]	1.888[28]	89.78[29]
3	DMF	0.949[30]	0.919[30]	0.667[31]	36.71[27]
4[a]	DMSO	1.100[32]	1.070[33]	1.532[33]	46.68[27]
5	DCM	1.327[34]		0.433[35]	9.10[27]
6	*t*BuOH	0.807[36]	0.781[36]	2.096[37]	10.60[38]
7	THF	0.887[39]	0.854[39]	0.418[40]	7.58[27]
8	EtOAc	0.900[41]	0.876[41]	0.370[41]	6.00[27]
9	Toluene	0.867[25]	0.839[25]	0.486[42]	2.33[27]
10	1,4-dioxane	1.034[43]	1.000[40]	0.921[40]	2.20[27]
11	DMC	1.070[44]	1.030[44]	0.491[45]	3.11[29]
12	DMA	0.937[46]		0.945[46]	37.8[47]

Table S18: Solvent densities, viscosities, and dielectric constants.

H. Computational Details

Complete authorship of software packages used in this work

Gaussian 16, Revision A.03, M. J. Frisch, G. W. Trucks, H. B. Schlegel, G. E. Scuseria, M. A. Robb, J. R. Cheeseman, G. Scalmani, V. Barone, G. A. Petersson, H. Nakatsuji, X. Li, M. Caricato, A. V. Marenich, J. Bloino, B. G. Janesko, R. Gomperts, B. Mennucci, H. P. Hratchian, J. V. Ortiz, A. F. Izmaylov, J. L. Sonnenberg, D. Williams-Young, F. Ding, F. Lipparini, F. Egidi, J. Goings, B. Peng, A. Petrone, T. Henderson, D. Ranasinghe, V. G. Zakrzewski, J. Gao, N. Rega, G. Zheng, W. Liang, M. Hada, M. Ehara, K. Toyota, R. Fukuda, J. Hasegawa, M. Ishida, T. Nakajima, Y. Honda, O. Kitao, H. Nakai, T. Vreven, K. Throssell, J. A. Montgomery, Jr., J. E. Peralta, F. Ogliaro, M. J. Bearpark, J. J. Heyd, E. N. Brothers, K. N. Kudin, V. N. Staroverov, T. A. Keith, R. Kobayashi, J. Normand, K. Raghavachari, A. P. Rendell, J. C. Burant, S. S. Iyengar, J. Tomasi, M. Cossi, J. M. Millam, M. Klene, C. Adamo, R. Cammi, J. W. Ochterski, R. L. Martin, K. Morokuma, O. Farkas, J. B. Foresman, and D. J. Fox, *Gaussian, Inc.*, Wallingford CT, **2016**.

Complete authorship of CYLview.

Legault, C. Y. *CYLview*, 1.0.565 BETA; University of Sherbrooke, Québec, Montreal, Canada, 2012. https://www.cylview.org/

GaussView, Version 6.0.16

Roy Dennington, Todd A. Keith, and John M. Millam, Semichem Inc., Shawnee Mission, KS, 2016.

Schrödinger Macromodel

Schrödinger Release 2019-4: MacroModel, Schrödinger, LLC, New York, NY, 2019.

General computational procedure

Conformational searches were performed using Schrödinger Macromodel software package. All computations were performed using Density Functional Theory (DFT) with the Gaussian 16 software package. All reactants, intermediates, and products were optimized using PBE-D3BJ[48,49]/ 6-31G(d)[50] for all other atoms. Solvation model based on density (SMD)[51] was used for CH_2Cl_2 at 1 atm and 243.15 K (45 °C). All reported energy values are free energies in kcal/mol and all distances are in Ångströms (Å).

H. 1. Computed Geometries, Dispersion & Solvation Corrections, and Energies

Structures within this section:

Catalytic Cycle Structures

Starting Material

Tetramisole

Acyl Ammonium

Deprotonation TS

Acyl Ammonium Ylide

C-N Homolysis TS (*Si*)

Geminate Radical Pair

[1,2] Radical Recombination TS (*Si*)

[2,3] Radical Recombination TS (*Re*)

(*R*)-[1,2]-Intermediate

Concerted [2,3] TS (*Re*)

(2*R*,3*R*)-[2,3]-Intermediate

(2*S*,3*R*)-[2,3]-Intermediate

(*R*)-[1,2]-Product (major)

(2*R*,3*R*)-[2,3]-Product (minor)

(2*S*,3*R*)-[2.3]-Product (minor)

$PNPO^-$

PNPOH

Br^-

Et_3N

HBr

Et_3NH—Br Complex

PNPOH—NEt_3 Complex

Desmethyl Variation Structures

Desmethyl C-N Homolysis (*Re*)

Desmethyl C-N Homolysis (*Si*)

Desmethyl Concerted [2,3] TS (*Re*)

Desmethyl Concerted [2,3] TS (*Si*)

Desmethyl [1,2] Radical Recombination TS (*Re*)

Desmethyl [1,2] Radical Recombination TS (*Si*)

Desmethyl [2,3] Radical Recombination TS (*Re*)

Desmethyl [2,3] Radical Recombination TS (*Si*)

Unfavored Transition Structures

C-N Homolysis TS (*Re*)

[1,2] Radical Recombination TS (*Re*)

[2,3] Radical Recombination TS (*Si*)

Dimethyl Amine Variation Structures

Dimethyl Amine C-N Homolysis TS

Dimethyl Amine Concerted [2,3] TS

Dimethyl Amine [1,2] Radical Recombination TS

Dimethyl Amine [2,3] Radical Recombination TS

Distortion Interaction Analysis Structures

[1,2] Radical Recombination Allyl TS Distortion

[1,2] Radical Recombination Allyl Distortion Interaction Ground State

[1,2] Radical Recombination Amide TS Distortion

[1,2] Radical Recombination Amide Distortion Interaction Ground State

[2,3] Radical Recombination Allyl TS Distortion

[2,3] Radical Recombination Allyl Distortion Interaction Ground State

[2,3] Radical Recombination Amide TS Distortion

[2,3] Radical Recombination Amide Distortion Interaction Ground State

Dimethyl Amine [1,2] Radical Recombination Allyl TS Distortion

Dimethyl Amine [1,2] Radical Recombination Allyl Distortion Interaction Ground State

Dimethyl Amine [1,2] Radical Recombination Amide TS Distortion

Dimethyl Amine [1,2] Radical Recombination Amide Distortion Interaction Ground State

Dimehtyl Amine [2,3] Radical Recombination Allyl TS Distortion

Dimethyl Amine [2,3] Radical Recombination Allyl Distortion Interaction Ground State

Dimethyl Amine [2,3] Radical Recombination Amide TS Distortion

Dimethyl Amine [2,3] Radical Recombination Amide Distortion Interaction Ground State

Supporting Information: **Starting Material**

```
----------------------------------------------------------------------
 Using  Gaussian 16:  ES64L-G16RevA.03 25-Dec-2016
======================================================================
 # m06/6-31g(d) gfprint gfinput scf=(direct,tight,maxcycle=300,xqc)
 opt=(maxcycle=250) freq=noraman scrf=(pcm,solvent=acetonitrile)
 iop(1/8=18) temperature=323.15
 #N Geom=AllCheck Guess=TCheck SCRF=Check Test GenChk RM06/6-31G(d) Freq
----------------------------------------------------------------------
 Pointgroup= C1   Stoichiometry= C22H25BrN2O4   C1[X(C22H25BrN2O4)]  #Atoms= 54
 Charge = 0       Multiplicity = 1
----------------------------------------------------------------------
 SCF Energy= -3834.42202352 Predicted Change= -7.742789D-08
======================================================================
 Optimization completed.           {Found        1        times}
 Item      Max Val.    Criteria    Pass?     RMS Val.    Criteria    Pass?
```

Force 0.00003 || 0.00045 [YES] 0.00000 || 0.00030 [YES]

Displ 0.00475 || 0.00180 [NO] 0.00475 || 0.00180 [YES]

Atomic Type	Coordinates (Angstroms) X	Y	Z
C	-0.489689	0.441514	0.233039
O	-0.850035	0.540498	1.377162
C	0.672563	1.128930	-0.423873
N	1.522124	1.924058	0.501495
C	2.263254	1.010351	1.487258
C	3.244900	0.100948	0.841657
C	3.074197	-1.211812	0.587824
C	4.188046	-1.997508	0.007231
C	0.767345	2.996222	1.278699
C	2.479219	2.758105	-0.324946
H	3.226282	2.106450	-0.786293
H	1.865856	3.211546	-1.113339
O	-1.093725	-0.326925	-0.694772
C	-2.323251	-0.881653	-0.360175
C	-2.385808	-2.245351	-0.112677
C	-3.619634	-2.815933	0.161708
C	-4.742135	-1.996290	0.181182
N	-6.043635	-2.596033	0.472285
O	-7.022804	-1.863916	0.485700
O	-6.086568	-3.799063	0.687619
C	-4.676095	-0.628654	-0.067154
C	-3.443549	-0.059911	-0.343814
C	5.522556	-1.699874	0.314754
C	6.563791	-2.430012	-0.243496
C	6.293931	-3.477613	-1.119910
C	4.973687	-3.794246	-1.424083
C	3.931707	-3.068061	-0.859375
H	1.311653	0.405845	-0.944538

H	1.472536	0.491330	2.035153
H	2.759389	1.684609	2.193753
H	4.204430	0.552941	0.585805
C	1.798065	-1.959045	0.844012
H	1.282902	-2.187784	-0.100900
H	1.091145	-1.411653	1.476070
H	2.002277	-2.921935	1.327935
C	1.803241	4.083865	1.570255
H	2.110017	4.073296	2.621390
H	1.361804	5.065981	1.374565
C	2.996780	3.804258	0.637717
H	3.324477	4.693131	0.091252
H	3.865911	3.432690	1.193849
H	0.325216	2.530893	2.161091
H	-0.041717	3.335905	0.616314
H	0.223466	1.813371	-1.161476
H	-1.479599	-2.845005	-0.139867
H	-3.718628	-3.878127	0.360913
H	-5.580937	-0.029616	-0.044178
H	-3.322461	1.003896	-0.559598
H	5.746544	-0.906131	1.025927
H	7.592031	-2.186407	0.017427
H	7.109593	-4.051071	-1.556077
H	4.750636	-4.613465	-2.105101
H	2.906203	-3.329335	-1.114554
Br	-1.876347	3.254524	-1.323219

Statistical Thermodynamic Analysis

Temperature= 323.150 Kelvin   Pressure= 1.00000 Atm

===

SCF Energy=   -3834.42202352   Predicted Change= -7.742789D-08

Zero-point correction (ZPE)=   -3833.9805   0.44151

Internal Energy (U)=   -3833.9488   0.47318

Enthalpy (H)=   -3833.9478   0.47420

Gibbs Free Energy (G)= -3834.0507 0.37127

Entropy (S)= 0.00031851

Frequencies -- 20.2605 23.7258 25.9207

Supporting Information: **Tetramisole**

Using Gaussian 16: ES64L-G16RevA.03 25-Dec-2016

==

m06/6-31g(d) gfprint gfinput scf=(direct,tight,maxcycle=300,xqc)

opt=(maxcycle=250) freq=noraman scrf=(pcm,solvent=acetonitrile)

iop(1/8=18) temperature=323.15

#N Geom=AllCheck Guess=TCheck SCRF=Check Test GenChk RM06/6-31G(d) Freq

----

Pointgroup= C1 Stoichiometry= C11H12N2S C1[X(C11H12N2S)] #Atoms= 26

Charge = 0 Multiplicity = 1

SCF Energy= -933.661092062 Predicted Change= -8.018380D-08

==

Optimization completed. {Found 1 times}

Item	Max Val.	Criteria	Pass?	RMS Val.	Criteria	Pass?
Force	0.00002 ||	0.00045	[YES]	0.00000 ||	0.00030	[YES]
Displ	0.01002 ||	0.00180	[NO]	0.01002 ||	0.00180	[NO]

Atomic Type	Coordinates (Angstroms) X	Y	Z
N	-1.723897	0.971044	0.088053
C	-0.340852	1.342607	0.363924
H	-0.045502	0.994042	1.372244
H	-0.175514	2.423346	0.299341
C	0.398779	0.540116	-0.731598
H	0.456267	1.156776	-1.645758

C	1.792834	0.148946	-0.325876
N	-0.483152	-0.614245	-1.012406
C	-1.611981	-0.271646	-0.521053
S	-3.113627	-1.190751	-0.426798
C	-3.911605	0.195669	0.492582
H	-4.584080	-0.216696	1.248580
H	-4.488319	0.800131	-0.214968
C	-2.763288	0.988228	1.099963
H	-2.407297	0.515598	2.034104
H	-3.073345	2.014547	1.328325
C	2.013017	-0.964380	0.486835
H	1.163228	-1.579209	0.782813
C	3.298979	-1.291583	0.902449
H	3.458852	-2.166379	1.530925
C	4.381080	-0.504013	0.515373
H	5.387962	-0.760574	0.840352
C	4.169501	0.609732	-0.291826
H	5.010315	1.228229	-0.601576
C	2.881624	0.930509	-0.711603
H	2.716265	1.799513	-1.350165

Statistical Thermodynamic Analysis

Temperature= 323.150 Kelvin Pressure= 1.00000 Atm

===

SCF Energy= -933.661092062 Predicted Change= -8.018380D-08

Zero-point correction (ZPE)= -933.4491 0.21196

Internal Energy (U)= -933.4358 0.22523

Enthalpy (H)= -933.4348 0.22626

Gibbs Free Energy (G)= -933.4929 0.16810

Entropy (S)= 0.00017997

Frequencies -- 15.8475 51.8604 82.6814

Supporting Information: **Acyl Ammonium**

Using Gaussian 16: ES64L-G16RevA.03 25-Dec-2016

==

m06/6-31g(d) gfprint gfinput scf=(direct,tight,maxcycle=300,xqc)

opt=(maxcycle=250) freq=noraman scrf=(pcm,solvent=acetonitrile)

iop(1/8=18) temperature=323.15

#N Geom=AllCheck Guess=TCheck SCRF=Check Test GenChk RM06/6-31G(d) Freq

----

Pointgroup= C1 Stoichiometry= C33H37BrN4O4S C1[X(C33H37BrN4O4S)] #Atoms= 80

Charge = 0 Multiplicity = 1

SCF Energy= -4768.10066692 Predicted Change= -3.731092D-07

==

Optimization completed. {Found 1 times}

Item	Max Val.	Criteria	Pass?	RMS Val.	Criteria	Pass?
Force	0.00002 ||	0.00045	[YES]	0.00000 ||	0.00030	[YES]
Displ	0.05272 ||	0.00180	[NO]	0.05272 ||	0.00180	[NO]

Atomic Type	Coordinates (Angstroms) X	Y	Z
C	-0.146435	0.723242	-0.455167
N	-0.449753	-0.319268	0.417938
C	0.806900	1.752952	0.086416
N	1.191377	2.801223	-0.896276
C	2.020405	3.843084	-0.167505
H	1.521778	4.004707	0.795343
H	3.019434	3.442116	0.022780
C	0.014323	3.628554	-1.396772
O	-0.670077	0.781075	-1.549236
C	0.321972	-0.864994	1.605761
C	0.832984	0.145288	2.585968
C	-0.675176	-1.892909	2.173555

C	-1.416217	-1.222653	0.107727
C	-2.441769	-3.230513	0.758548
H	-3.092709	-3.482057	1.602128
H	-1.751201	-4.061716	0.551695
C	-3.249907	-2.825196	-0.473711
H	-3.362783	-3.651309	-1.179534
H	-4.241460	-2.430973	-0.218994
S	-2.318581	-1.465655	-1.326986
C	2.206241	0.199810	2.830079
C	2.727290	1.146910	3.705866
C	1.875950	2.047324	4.338587
C	0.503908	1.994059	4.101080
C	-0.018290	1.041422	3.234558
N	-1.625833	-2.069796	1.083873
C	1.976383	2.211226	-2.080010
C	3.105622	1.353004	-1.640236
C	3.189809	0.013875	-1.774502
C	4.396255	-0.681094	-1.263448
C	5.664000	-0.093389	-1.366720
C	6.789677	-0.729486	-0.858177
C	6.669895	-1.967952	-0.233738
C	5.417868	-2.567343	-0.135773
C	4.289897	-1.939104	-0.653845
H	0.292130	2.246221	0.920285
H	1.728485	1.291740	0.459153
H	-0.694269	3.674549	-0.558031
H	-0.462902	3.087920	-2.215734
C	0.608235	4.981194	-1.777632
H	-0.078126	5.780075	-1.481165
H	0.744372	5.061271	-2.861448
C	1.959087	5.079583	-1.043372
H	2.797098	5.107181	-1.748891
H	2.036951	5.977504	-0.423656
H	1.157569	-1.424772	1.156898

H	-1.193162	-1.543188	3.076276
H	-0.168875	-2.844661	2.372955
H	2.871527	-0.492619	2.311516
H	3.799375	1.186011	3.886589
H	2.281573	2.796133	5.016036
H	-0.162402	2.702864	4.588418
H	-1.082892	1.044400	3.005225
H	2.317733	3.078771	-2.656411
H	1.234506	1.677636	-2.677055
H	3.936391	1.867861	-1.154185
C	2.148205	-0.847122	-2.422647
H	1.371059	-0.275290	-2.938219
H	1.647869	-1.513079	-1.698350
H	2.622093	-1.512784	-3.156515
H	5.772088	0.862199	-1.878533
H	7.766255	-0.259159	-0.959118
H	7.550688	-2.467999	0.165098
H	5.314401	-3.538290	0.346213
H	3.320541	-2.436266	-0.565152
C	-3.633462	2.248960	-0.613276
C	-2.954094	1.931106	0.619910
O	-1.919092	2.544940	1.000489
C	-3.525990	0.844550	1.380797
C	-4.585209	0.108837	0.920805
C	-5.169610	0.419579	-0.321000
N	-6.213675	-0.385437	-0.827483
O	-6.520564	-1.425273	-0.219259
O	-6.792171	-0.045268	-1.867555
C	-4.697100	1.518038	-1.067602
H	-3.248628	3.087147	-1.194440
H	-3.084597	0.614997	2.351552
H	-4.988889	-0.716657	1.504010
H	-5.178230	1.753592	-2.013849
Br	0.903287	-3.699986	-0.311717

Statistical Thermodynamic Analysis

Temperature= 323.150 Kelvin Pressure= 1.00000 Atm

==

SCF Energy= -4768.10066692 Predicted Change= -3.731092D-07

Zero-point correction (ZPE)= -4767.4452 0.65544

Internal Energy (U)= -4767.3984 0.70226

Enthalpy (H)= -4767.3973 0.70328

Gibbs Free Energy (G)= -4767.5335 0.56709

Entropy (S)= 0.00042146

Frequencies -- 3.1554 22.3439 27.5815

Supporting Information: **Deprotonation TS**

Using Gaussian 16: ES64L-G16RevA.03 25-Dec-2016

==

#m06/6-31g(d) scf=(direct,tight,maxcycle=300,xqc)

opt=(nofreeze,maxcycle=250,ts,calcfc,noeigentest) iop(1/8=18) freq=noraman

scrf=(pcm,solvent=acetonitrile) temperature=323.15

#N Geom=AllCheck Guess=TCheck SCRF=Check Test GenChk RM06/6-31G(d) Freq

----

Pointgroup= C1 Stoichiometry= C33H37BrN4O4S C1[X(C33H37BrN4O4S)] #Atoms= 80

Charge = 0 Multiplicity = 1

SCF Energy= -4768.07883038 Predicted Change= -6.069279D-09

==

Optimization completed. {Found 2 times}

Item Max Val. Criteria Pass? RMS Val. Criteria Pass?

Force 0.00000 || 0.00045 [YES] 0.00000 || 0.00030 [YES]

Displ 0.00122 || 0.00180 [YES] 0.00122 || 0.00180 [YES]

Atomic Coordinates (Angstroms)

Type	X	Y	Z
C	0.354500	0.573581	0.096304
N	0.138668	-0.689910	0.720161
C	-0.108014	1.710056	0.866186
H	-0.037581	1.602885	1.951531
N	0.406560	3.061517	0.496269
C	-0.384064	4.108380	1.266969
C	0.108437	3.452867	-0.936555
H	0.782383	2.920636	-1.607707
H	-0.917584	3.113057	-1.118896
O	0.741802	0.567283	-1.064782
C	-0.264966	-1.011703	2.132509
C	-1.665869	-0.572049	2.472836
C	-0.092867	-2.548252	2.161131
C	0.209325	-1.834920	0.017162
C	0.208374	-4.178281	0.132066
H	-0.540448	-4.920675	0.424937
H	1.205704	-4.479764	0.484451
C	0.182303	-3.917956	-1.370639
H	0.913553	-4.525755	-1.908259
H	-0.809854	-4.079700	-1.802450
S	0.610286	-2.128023	-1.626054
C	-2.760234	-1.067003	1.758985
C	-4.052070	-0.711201	2.123314
C	-4.263470	0.142338	3.203817
C	-3.178405	0.634968	3.921210
C	-1.883786	0.276051	3.557277
N	-0.101114	-2.890395	0.741647
C	1.885307	3.267020	0.860580
C	2.851031	2.611591	-0.050279
C	3.600657	1.528155	0.220633
C	4.553313	1.043421	-0.805711
C	5.257726	1.944947	-1.615321

C	6.130664	1.493378	-2.597259
C	6.319061	0.127362	-2.789913
C	5.634873	-0.777671	-1.984210
C	4.766459	-0.328659	-0.994463
C	-0.234596	5.401775	0.469128
H	0.495755	6.078288	0.925791
H	-1.192061	5.931116	0.452019
C	0.207159	4.967242	-0.942250
H	-0.427683	5.384357	-1.729516
H	1.232856	5.292924	-1.154947
H	0.003234	4.141356	2.290281
H	-1.419931	3.759680	1.287265
H	0.460344	-0.536032	2.804891
H	-0.910726	-3.045174	2.691388
H	0.883333	-2.846516	2.570734
H	-2.606661	-1.733503	0.909132
H	-4.897063	-1.097859	1.556320
H	-5.276656	0.420985	3.487538
H	-3.337402	1.298331	4.769056
H	-1.033577	0.653269	4.127299
H	1.960588	2.925061	1.897538
H	2.039145	4.352956	0.863456
H	2.963765	3.081578	-1.028957
C	3.524581	0.744042	1.493176
H	4.531234	0.546494	1.886127
H	3.074305	-0.249566	1.330480
H	2.947677	1.246343	2.277139
H	5.139813	3.015693	-1.452565
H	6.674219	2.212597	-3.207692
H	7.003824	-0.228814	-3.557632
H	5.778224	-1.848380	-2.122899
H	4.247004	-1.063044	-0.374216
H	-1.433711	1.792715	0.578224
C	-4.740110	1.415988	-0.410375

C	-3.321285	1.357764	-0.435176
O	-2.638233	2.126495	0.360488
C	-2.713744	0.451474	-1.345714
C	-3.477037	-0.385973	-2.128432
C	-4.872522	-0.328014	-2.051002
N	-5.664357	-1.201407	-2.863735
O	-5.091404	-2.008431	-3.597754
O	-6.891929	-1.117141	-2.799507
C	-5.503174	0.588500	-1.198879
H	-5.205187	2.119521	0.277835
H	-1.627801	0.426137	-1.437125
H	-3.009503	-1.086414	-2.815608
H	-6.588545	0.624989	-1.166072
Br	3.230998	-2.935171	1.292211

Statistical Thermodynamic Analysis

Temperature= 323.150 Kelvin Pressure= 1.00000 Atm

==

SCF Energy= -4768.07883038 Predicted Change= -6.069279D-09

Zero-point correction (ZPE)= -4767.4275 0.65132

Internal Energy (U)= -4767.3817 0.69706

Enthalpy (H)= -4767.3807 0.69809

Gibbs Free Energy (G)= -4767.5120 0.56682

Entropy (S)= 0.00040621

Frequencies -- -1563.4837 14.5385 20.7206

Supporting Information: **Acyl Ammonium Ylide**

Using Gaussian 16: ES64L-G16RevA.03 25-Dec-2016

==

m06/6-31g(d) gfprint gfinput scf=(direct,tight,maxcycle=300,xqc)

opt=(maxcycle=250) freq=noraman scrf=(pcm,solvent=acetonitrile)

iop(1/8=18) temperature=323.15

#N Geom=AllCheck Guess=TCheck SCRF=Check Test GenChk RM06/6-31G(d) Freq

----

Pointgroup= C1 Stoichiometry= C27H32BrN3OS C1[X(C27H32BrN3OS)] #Atoms= 65

Charge = 0 Multiplicity = 1

SCF Energy= -4256.38741696 Predicted Change= -2.203163D-08

==

Optimization completed. {Found 1 times}

Item	Max Val.	Criteria	Pass?	RMS Val.	Criteria	Pass?
Force	0.00001 ||	0.00045	[YES]	0.00000 ||	0.00030	[YES]
Displ	0.00228 ||	0.00180	[NO]	0.00228 ||	0.00180	[YES]

Atomic Type	Coordinates (Angstroms) X	Y	Z
C	-3.679873	-3.135429	0.943926
H	-4.607121	-2.581476	0.730626
H	-3.805832	-3.761078	1.833517
C	-3.222408	-3.954913	-0.256862
H	-2.603455	-4.807936	0.038321
H	-4.063766	-4.311182	-0.856282
N	-2.610308	-2.168576	1.158686
C	-1.977100	-1.797445	0.046966
S	-2.185686	-2.832360	-1.313714
C	-2.629772	-1.034426	2.078515
C	-1.402701	-0.228466	1.613700
C	-1.543915	1.258321	1.795074
N	-1.276399	-0.681709	0.195876
C	-0.388543	-0.199052	-0.852844
O	-0.576001	-0.702134	-1.983853
C	0.535612	0.729745	-0.450426
H	0.633993	1.122030	0.551223
N	1.403707	1.358494	-1.459555

C	0.583262	2.075193	-2.514318
C	2.135581	2.510027	-0.824649
H	2.591739	2.171215	0.110107
H	2.931432	2.791675	-1.525085
C	2.356209	0.362193	-2.100496
C	3.252927	-0.280510	-1.104496
H	2.751241	-0.964142	-0.418745
C	4.580068	-0.100329	-0.975419
C	5.326192	-0.875610	0.044817
C	6.445308	-0.329149	0.687200
C	7.132808	-1.045778	1.660317
C	6.728007	-2.332607	2.001208
C	5.630969	-2.897944	1.356517
C	4.941674	-2.178938	0.388523
C	-2.473059	1.993566	1.055290
C	-2.574886	3.367515	1.243460
C	-1.763600	4.015574	2.173928
C	-0.848457	3.282510	2.922760
C	-0.737440	1.908196	2.728738
H	-2.550478	-1.354952	3.120949
H	-3.564098	-0.472025	1.917906
H	-0.501842	-0.576131	2.144499
C	0.051299	3.305874	-1.797173
H	-0.064784	4.135308	-2.502089
C	1.085638	3.605953	-0.691372
H	1.553552	4.588753	-0.807093
H	0.614534	3.583080	0.299359
H	-0.932005	3.102872	-1.357553
H	-0.165416	1.375555	-2.892255
H	1.277414	2.339443	-3.322579
H	2.895763	0.908058	-2.882126
H	1.694268	-0.372066	-2.576107
C	5.384922	0.867726	-1.793103
H	4.847059	1.243470	-2.668462

H	6.316575	0.405384	-2.143138
H	5.672435	1.742832	-1.192667
H	6.776935	0.677775	0.438654
H	7.991686	-0.594177	2.153836
H	7.270806	-2.896452	2.757590
H	5.316812	-3.911572	1.599463
H	4.107053	-2.645665	-0.132668
H	-3.123193	1.480750	0.340743
H	-3.296362	3.937955	0.660665
H	-1.848857	5.091314	2.316839
H	-0.215040	3.779329	3.655203
H	-0.009955	1.333765	3.303775
Br	-4.985812	-0.243860	-0.609998

Statistical Thermodynamic Analysis

Temperature= 323.150 Kelvin Pressure= 1.00000 Atm

==

SCF Energy= -4256.38741696 Predicted Change= -2.203163D-08

Zero-point correction (ZPE)= -4255.8423 0.54504

Internal Energy (U)= -4255.8061 0.58129

Enthalpy (H)= -4255.8050 0.58231

Gibbs Free Energy (G)= -4255.9177 0.46970

Entropy (S)= 0.00034847

Frequencies -- 12.4469 16.7223 17.4922

Supporting Information: **C-N Homolysis TS (*si*)**

Using Gaussian 16: ES64L-G16RevA.03 25-Dec-2016

==

#um06/6-31G(d) scf=(direct,tight,maxcycle=300,xqc) guess=(mix,always)

opt=(maxcycle=250,ts,calcfc,noeigentest) iop(1/8=18) freq=noraman

SCRF=(PCM,SOLVENT=acetonitrile) Temperature=323.15

#N Geom=AllCheck Guess=TCheck SCRF=Check Test GenChk UM06/6-31G(d) Freq

----

Pointgroup= C1 Stoichiometry= C27H32BrN3OS C1[X(C27H32BrN3OS)] #Atoms= 65

Charge = 0 Multiplicity = 1

SCF Energy= -4256.36973736 Predicted Change= -8.870817D-10

==

Optimization completed. {Found 2 times}

Item	Max Val.		Criteria	Pass?	RMS Val.		Criteria	Pass?
Force	0.00000	||	0.00045	[YES]	0.00000	||	0.00030	[YES]
Displ	0.00036	||	0.00180	[YES]	0.00036	||	0.00180	[YES]

Atomic Type	Coordinates (Angstroms) X	Y	Z
C	-4.376333	1.294688	-0.323824
H	-4.528463	2.308902	-0.722852
H	-5.006177	1.167125	0.564000
C	-4.659426	0.214955	-1.362662
H	-4.973609	-0.722148	-0.891034
H	-5.410319	0.522398	-2.093566
N	-2.979351	1.108356	0.026303
C	-2.217455	0.536654	-0.895668
S	-3.064880	-0.117019	-2.253230
C	-2.168105	1.778632	1.035327
C	-0.714529	1.485787	0.574058
C	0.076503	2.719997	0.214877
N	-0.925629	0.621006	-0.629735
C	0.085057	-0.055717	-1.437482
O	-0.279720	-0.424321	-2.568962
C	1.300681	-0.166662	-0.771824
H	1.388821	0.132436	0.270159
N	2.411157	-0.829497	-1.229374
C	2.634254	-1.036974	-2.671821

C	3.694803	-0.332296	-0.681508
H	3.731824	0.754481	-0.863976
H	3.699004	-0.485673	0.405597
C	-0.388254	3.589278	-0.775537
C	0.330507	4.733471	-1.099337
C	1.520879	5.019645	-0.433770
C	1.988367	4.155213	0.550545
C	1.271037	3.006361	0.874543
H	-2.381506	1.363036	2.027649
H	-2.386687	2.854619	1.044459
H	-0.180492	0.906700	1.342589
C	4.066837	-1.555194	-2.761588
H	4.082655	-2.650340	-2.816064
C	4.770055	-1.063824	-1.477618
H	5.183737	-1.906987	-0.912235
H	5.605402	-0.390869	-1.697596
H	4.553050	-1.186006	-3.670556
H	2.523705	-0.064442	-3.176649
H	1.872460	-1.708024	-3.078990
H	-1.317861	3.370350	-1.304741
H	-0.038478	5.403870	-1.873341
H	2.084016	5.916174	-0.686905
H	2.920784	4.372158	1.068934
H	1.646487	2.310263	1.629290
C	2.338014	-2.891240	-0.395685
H	3.322525	-2.891020	0.068220
H	2.289856	-3.354790	-1.378359
C	1.215930	-2.753647	0.416160
Br	2.238365	-0.057333	2.831586
C	-0.090898	-2.764734	-0.039880
C	-0.430762	-3.284087	-1.397923
H	0.442392	-3.671175	-1.929246
H	-1.160135	-4.102522	-1.337603
H	-0.869435	-2.490550	-2.024153

H	1.414605	-2.392274	1.426262
C	-1.213254	-2.349006	0.820078
C	-1.008240	-1.672753	2.038595
C	-2.545127	-2.582179	0.430662
C	-2.077768	-1.263691	2.822523
H	-0.001652	-1.407553	2.364957
C	-3.615003	-2.177795	1.222417
H	-2.761383	-3.092203	-0.505320
C	-3.390340	-1.513231	2.423453
H	-1.880235	-0.734972	3.754772
H	-4.632797	-2.394439	0.899541
H	-4.226837	-1.191943	3.041592

Statistical Thermodynamic Analysis

Temperature= 323.150 Kelvin Pressure= 1.00000 Atm

==

SCF Energy= -4256.36973736 Predicted Change= -8.870817D-10

Zero-point correction (ZPE)= -4255.8274 0.54229

Internal Energy (U)= -4255.7914 0.57827

Enthalpy (H)= -4255.7904 0.57929

Gibbs Free Energy (G)= -4255.8982 0.47143

Entropy (S)= 0.00033377

Frequencies -- -195.9279 28.7598 33.5367

Supporting Information: **Geminate Radical Pair**

Using Gaussian 16: ES64L-G16RevA.03 25-Dec-2016

==

um06/6-31g(d) gfprint gfinput scf=(direct,tight,maxcycle=300,xqc)

guess=(mix,always) opt=(maxcycle=250) freq=noraman

scrf=(pcm,solvent=acetonitrile) iop(1/8=18) temperature=323.15

#N Geom=AllCheck Guess=TCheck SCRF=Check Test GenChk UM06/6-31G(d) Freq

----

Pointgroup= C1   Stoichiometry= C27H32BrN3OS   C1[X(C27H32BrN3OS)] #Atoms= 65

Charge = 0   Multiplicity = 1

SCF Energy= -4256.37223867 Predicted Change= -7.234836D-09

==

Optimization completed.   {Found   2   times}

Item	Max Val.		Criteria	Pass?	RMS Val.		Criteria	Pass?
Force	0.00004	||	0.00045	[YES]	0.00000	||	0.00030	[YES]
Displ	0.00133	||	0.00180	[YES]	0.00133	||	0.00180	[YES]

Atomic Type	Coordinates (Angstroms) X	Y	Z
C	3.722331	1.500657	-0.176682
H	4.506668	0.784832	0.101909
H	3.895507	2.459126	0.329483
C	3.654493	1.645554	-1.703055
H	3.880582	2.660537	-2.039017
H	4.327311	0.929751	-2.184409
N	2.445101	0.959490	0.247042
C	1.554199	0.690857	-0.683407
S	1.939909	1.218632	-2.273090
C	2.137968	0.249879	1.476573
C	0.661899	-0.152482	1.271211
C	0.339265	-1.534405	1.769564
N	0.489359	0.034429	-0.206180
C	-0.678336	-0.156209	-1.015920
O	-0.612279	0.210869	-2.196269
C	-1.789971	-0.757930	-0.374676
H	-1.756529	-1.002439	0.682499
N	-2.912014	-1.116255	-1.013969
C	-3.148304	-1.085155	-2.464461
C	-3.986758	-1.822346	-0.306560

H	-3.560333	-2.497290	0.446826
H	-4.628131	-1.093308	0.213509
C	0.864829	-2.668823	1.146626
C	0.540079	-3.932039	1.627420
C	-0.299379	-4.073108	2.730929
C	-0.810752	-2.944095	3.362419
C	-0.492867	-1.677793	2.879945
H	2.272157	0.892641	2.353431
H	2.799119	-0.628612	1.540734
H	0.006151	0.582232	1.762664
C	-4.585777	-1.566836	-2.594350
H	-5.275941	-0.718500	-2.480832
C	-4.741624	-2.529807	-1.421129
H	-5.784252	-2.730234	-1.155154
H	-4.257436	-3.488874	-1.650615
H	-4.777720	-2.031212	-3.566905
H	-2.439867	-1.768479	-2.957421
H	-2.972285	-0.080508	-2.857804
H	1.546925	-2.556015	0.299386
H	0.946556	-4.814988	1.137433
H	-0.551989	-5.065360	3.100106
H	-1.462035	-3.046600	4.228121
H	-0.898240	-0.789060	3.367260
C	-4.418880	1.761651	-1.084338
H	-5.129422	1.268541	-0.422925
H	-4.747494	1.919777	-2.109560
C	-3.200303	2.217708	-0.674339
H	-2.604979	2.729451	-1.431646
C	-2.638539	2.112174	0.621872
C	-1.361537	2.729784	0.943507
C	-0.458590	3.180993	-0.046062
C	0.757493	3.754742	0.292304
C	1.130099	3.899865	1.628737
C	0.258200	3.463518	2.623862

C	-0.961530	2.890069	2.289847
C	-3.427434	1.489500	1.733806
H	-3.850682	2.251433	2.406789
H	-2.810276	0.828921	2.358334
H	-4.264034	0.891275	1.361324
H	-0.694982	3.055737	-1.101383
H	1.429281	4.085627	-0.500170
H	2.087330	4.347845	1.889432
H	0.527742	3.573484	3.672991
H	-1.623271	2.569250	3.092004
Br	3.938443	-1.827521	-0.921626

Statistical Thermodynamic Analysis

Temperature= 323.150 Kelvin   Pressure= 1.00000 Atm

==

SCF Energy=   -4256.37223867   Predicted Change= -7.234836D-09

Zero-point correction (ZPE)=   -4255.8329   0.53924

Internal Energy (U)=   -4255.7948   0.57736

Enthalpy (H)=   -4255.7938   0.57838

Gibbs Free Energy (G)=   -4255.9097   0.46252

Entropy (S)=   0.00035852

Frequencies --   18.3483   20.1926   24.2521

Supporting Information: **[1,2]-Radical Recombination TS (*si*)**

Using Gaussian 16: ES64L-G16RevA.03 25-Dec-2016

==

#um06/6-31G(d) scf=(maxcycle=300,direct,tight,xqc) guess=(mix,always)

density=current opt=(maxcycle=250,modredundant) iop(1/8=18)

Temperature=323.15 SCRF=(PCM,SOLVENT=acetonitrile)

Modredundant Input: B   3   44 F

Modredundant Input:

#um06/6-31G(d) scf=(direct,tight,maxcycle=300,xqc) guess=(mix,always) opt=(nofreeze,maxcycle=250,ts,calcfc,noeigentest) iop(1/8=18) freq=noraman SCRF=(PCM,SOLVENT=acetonitrile) Temperature=323.15 geom=check

#N Geom=AllCheck Guess=TCheck SCRF=Check Test GenChk UM06/6-31G(d) Freq

----

Pointgroup= C1 Stoichiometry= C27H32BrN3OS C1[X(C27H32BrN3OS)] #Atoms= 65

Charge = 0 Multiplicity = 1

SCF Energy= -4256.37591105 Predicted Change= 3.249056D-10

===

Optimization completed. {Found 3 times}

Item	Max Val.		Criteria	Pass?	RMS Val.		Criteria	Pass?
Force	0.00000	||	0.00045	[YES]	0.00000	||	0.00030	[YES]
Displ	0.00032	||	0.00180	[YES]	0.00032	||	0.00180	[YES]

Atomic Type	Coordinates (Angstroms) X	Y	Z
C	0.719657	-0.319608	-1.345001
N	-0.107314	0.628345	-0.665547
C	1.885947	-0.693285	-0.616678
N	2.883891	-1.433034	-1.118924
C	2.798662	-2.296516	-2.302705
H	1.895067	-2.921887	-2.231916
H	2.708847	-1.700647	-3.217699
C	4.037559	-1.784249	-0.279935
O	0.335679	-0.733332	-2.443521
C	0.266224	1.522696	0.479270
C	1.365744	2.478301	0.085186
C	-1.078132	2.229108	0.799076
C	-1.358857	0.881638	-1.042997
C	-3.209317	2.308091	-0.731020
H	-2.988608	3.289295	-1.178070
H	-3.923382	2.442693	0.089814

C	-3.730027	1.305091	-1.753732
H	-4.379400	0.554992	-1.288183
H	-4.258548	1.786256	-2.579458
S	-2.252964	0.413891	-2.442456
C	1.221541	3.298201	-1.037676
C	2.218028	4.205004	-1.375734
C	3.366323	4.302898	-0.591829
C	3.514626	3.486948	0.524350
C	2.519097	2.574071	0.863170
H	2.079296	-0.261433	0.364537
H	3.772151	-1.674540	0.780989
H	4.871830	-1.097607	-0.495630
C	4.367075	-3.196253	-0.731963
H	3.688992	-3.911485	-0.243042
H	5.397250	-3.486383	-0.500858
C	4.075163	-3.128499	-2.229025
H	4.894789	-2.610303	-2.746030
H	3.952002	-4.110488	-2.697075
H	0.589965	0.901377	1.328707
H	-0.994362	3.320947	0.717239
H	-1.461169	1.984467	1.797102
H	0.325660	3.228555	-1.658014
H	2.098978	4.838015	-2.252994
H	4.146299	5.014582	-0.856273
H	4.413024	3.555720	1.135125
H	2.638357	1.909176	1.721916
N	-1.979115	1.721147	-0.227302
C	0.450325	-2.417253	0.532982
H	1.267456	-2.318883	1.245473
H	0.597501	-3.091559	-0.309633
C	-0.801562	-1.946779	0.907006
C	-2.011103	-2.106157	0.236911
C	-2.108928	-2.990100	-0.969993
H	-1.130753	-3.349515	-1.301000

H	-2.732850	-3.874672	-0.771288
H	-2.565839	-2.462540	-1.820024
C	-3.233182	-1.453475	0.701530
C	-4.477833	-1.750068	0.108555
C	-3.233976	-0.472266	1.716233
C	-5.643951	-1.101344	0.497741
H	-4.538046	-2.503572	-0.673744
C	-4.396126	0.177904	2.101247
H	-2.303453	-0.203974	2.213788
C	-5.613423	-0.126163	1.491168
H	-6.585625	-1.360865	0.016474
H	-4.350293	0.934678	2.883478
H	-6.524908	0.387007	1.791027
H	-0.790847	-1.356505	1.827134
Br	2.186087	-0.484266	3.011462

Statistical Thermodynamic Analysis

Temperature= 323.150 Kelvin   Pressure= 1.00000 Atm

==

SCF Energy= -4256.37591105 Predicted Change= 3.249056D-10

Zero-point correction (ZPE)= -4255.8351 0.54073

Internal Energy (U)= -4255.7987 0.57718

Enthalpy (H)= -4255.7977 0.57820

Gibbs Free Energy (G)= -4255.9083 0.46759

Entropy (S)= 0.00034228

Frequencies -- -199.7430 14.3165 28.1022

Supporting Information: **[2,3]-Radical Recombination TS (*re*)**

Using Gaussian 16: ES64L-G16RevA.03 25-Dec-2016

==

#um06/6-31g(d) scf=(direct,tight,maxcycle=300,xqc) guess=(mix,always)

opt=(nofreeze,maxcycle=250,ts,calcfc,noeigentest) iop(1/8=18) freq=noraman

scrf=(pcm,solvent=acetonitrile) temperature=323.15

#N Geom=AllCheck Guess=TCheck SCRF=Check Test GenChk UM06/6-31G(d) Freq

----

Pointgroup= C1 Stoichiometry= C27H32BrN3OS C1[X(C27H32BrN3OS)] #Atoms= 65

Charge = 0 Multiplicity = 1

SCF Energy= -4256.37086786 Predicted Change= -1.151327D-09

==

Optimization completed. {Found 2 times}

Item	Max Val.		Criteria	Pass?	RMS Val.		Criteria	Pass?
Force	0.00000	||	0.00045	[YES]	0.00000	||	0.00030	[YES]
Displ	0.00044	||	0.00180	[YES]	0.00044	||	0.00180	[YES]

Atomic Type	Coordinates (Angstroms) X	Y	Z
C	0.853218	-0.791514	-0.915818
N	-0.422880	-0.151636	-0.918270
C	1.824173	-0.175306	-0.066647
N	3.145480	-0.397167	-0.172552
C	4.106423	0.510800	0.464693
H	4.052657	1.503172	-0.015225
H	3.873730	0.637476	1.528494
C	3.797499	-1.238190	-1.192184
O	0.985747	-1.800330	-1.609259
C	-0.813671	1.134513	-0.275907
C	-0.148841	2.322051	-0.928644
C	-2.347926	1.110114	-0.429656
C	-1.443164	-0.592172	-1.658270
C	-3.751427	-0.435402	-2.032893
H	-4.434551	0.340233	-2.392144
H	-4.239364	-0.992028	-1.217827
C	-3.296854	-1.350237	-3.162651

H	-3.942294	-2.223569	-3.277574
H	-3.232588	-0.818164	-4.116595
S	-1.587177	-1.932735	-2.726388
C	0.218969	2.325881	-2.274330
C	0.791435	3.461825	-2.838535
C	0.999066	4.599169	-2.063696
C	0.631856	4.597980	-0.720607
C	0.060890	3.464279	-0.152639
H	1.549838	0.718349	0.489544
H	3.303851	-1.111444	-2.161627
H	3.710007	-2.295333	-0.911597
C	5.243059	-0.745923	-1.194841
H	5.366154	0.041659	-1.950916
H	5.950422	-1.547235	-1.432189
C	5.443394	-0.155228	0.198297
H	5.619710	-0.951474	0.934209
H	6.277800	0.551569	0.251319
H	-0.585576	1.112384	0.796802
H	-2.756679	2.094019	-0.680888
H	-2.830694	0.737447	0.486681
H	0.073055	1.437469	-2.889905
H	1.079368	3.455269	-3.888161
H	1.450097	5.485415	-2.506276
H	0.796033	5.483495	-0.109109
H	-0.242083	3.448408	0.897154
N	-2.524143	0.165519	-1.529907
C	3.314654	-2.343146	2.020996
H	4.078650	-3.086194	1.799862
H	3.628135	-1.508784	2.645698
C	2.047071	-2.515942	1.590217
H	1.860633	-3.413264	0.999919
C	0.948632	-1.608423	1.766571
C	-0.408679	-2.073030	1.455566
C	-1.536158	-1.398419	1.967910

C	-2.828968	-1.815981	1.676246
C	-3.051714	-2.929163	0.869542
C	-1.953028	-3.612045	0.353047
C	-0.660120	-3.191689	0.631815
C	1.096341	-0.522959	2.800272
H	0.848368	-0.915799	3.800263
H	0.441518	0.344795	2.640438
H	2.122928	-0.146636	2.850201
H	-1.419142	-0.513707	2.592531
H	-3.669091	-1.259689	2.093592
H	-4.064669	-3.261650	0.648186
H	-2.102744	-4.479883	-0.287812
H	0.162125	-3.738802	0.178388
Br	-1.754935	2.191166	2.906252

Statistical Thermodynamic Analysis

Temperature= 323.150 Kelvin Pressure= 1.00000 Atm

==

SCF Energy= -4256.37086786 Predicted Change= -1.151327D-09

Zero-point correction (ZPE)= -4255.8300 0.54082

Internal Energy (U)= -4255.7935 0.57730

Enthalpy (H)= -4255.7925 0.57833

Gibbs Free Energy (G)= -4255.9027 0.46812

Entropy (S)= 0.00034104

Frequencies -- -305.6237 17.6204 26.6773

Supporting Information: **(*R*)-[1,2]-Intermediate**

Using Gaussian 16: ES64L-G16RevA.03 25-Dec-2016

==

#m06/6-31g(d) scf=(maxcycle=300,direct,vshift=200,tight,yqc)

density=current scrf=(pcm,solvent=ch3cn) opt=(gdiis,maxcycle=250)

freq=noraman temperature=323.15

#N Geom=AllCheck Guess=TCheck SCRF=Check GenChk RM06/6-31G(d) Freq

----

Pointgroup= C1   Stoichiometry= C27H32BrN3OS   C1[X(C27H32BrN3OS)] #Atoms= 65

Charge = 0   Multiplicity = 1

SCF Energy= -4256.42191774 Predicted Change= -3.161206D-08

==

Optimization completed.   {Found   1   times}

Item	Max Val.		Criteria	Pass?	RMS Val.		Criteria	Pass?
Force	0.00002	||	0.00045	[YES]	0.00000	||	0.00030	[YES]
Displ	0.02006	||	0.00180	[NO]	0.02006	||	0.00180	[NO]

Atomic Type	Coordinates (Angstroms) X	Y	Z
C	-0.491981	-0.408479	1.336553
N	0.087005	0.747383	0.759185
C	-1.366705	-1.261230	0.436254
N	-2.707571	-1.337936	0.969398
C	-2.974458	-2.227181	2.094092
H	-2.674730	-3.274887	1.885338
H	-2.437642	-1.895319	2.991023
C	-3.734347	-1.599248	-0.046317
O	-0.196929	-0.697792	2.476043
C	-0.350595	1.485164	-0.473113
C	-1.721028	2.080536	-0.294064
C	0.784085	2.525973	-0.658395
C	1.262174	1.235972	1.185493
C	2.953901	2.831739	0.828407
H	2.697932	3.784925	1.310933
H	3.597585	3.029926	-0.035833
C	3.606626	1.841239	1.786501
H	4.266586	1.140193	1.260639

H	4.157191	2.334068	2.590151
S	2.239999	0.832387	2.540518
C	-2.031513	2.835283	0.839841
C	-3.293875	3.398146	0.979626
C	-4.252804	3.214906	-0.015143
C	-3.945331	2.467396	-1.147021
C	-2.682214	1.897944	-1.287951
H	-1.445013	-0.793112	-0.555698
H	-3.379804	-2.318620	-0.808963
H	-3.969590	-0.665193	-0.578872
C	-4.927131	-2.174367	0.735092
H	-5.113463	-3.210494	0.425143
H	-5.852005	-1.614077	0.559058
C	-4.486090	-2.134842	2.202984
H	-4.761279	-1.177158	2.664548
H	-4.922520	-2.940248	2.804255
H	-0.354716	0.787771	-1.325516
H	0.432273	3.555688	-0.510463
H	1.260955	2.451615	-1.642727
H	-1.284973	2.980341	1.622624
H	-3.531655	3.980927	1.867395
H	-5.241833	3.655576	0.095784
H	-4.693097	2.320073	-1.924207
H	-2.432848	1.289098	-2.160009
N	1.733329	2.170343	0.390125
C	-0.597372	-2.595695	0.221085
H	-1.189382	-3.209743	-0.474634
H	-0.524086	-3.138161	1.171168
C	0.730521	-2.237555	-0.364376
C	1.929369	-2.201228	0.248039
C	2.184437	-2.852576	1.577172
H	1.275852	-3.279953	2.009282
H	2.911785	-3.671097	1.476210
H	2.600167	-2.145308	2.307565

C	3.065680	-1.463297	-0.364627
C	4.358446	-1.568252	0.170446
C	2.884196	-0.565984	-1.434640
C	5.418324	-0.815115	-0.328406
H	4.549158	-2.248362	0.998049
C	3.940792	0.186213	-1.930843
H	1.903496	-0.444972	-1.899818
C	5.215718	0.073195	-1.378732
H	6.407779	-0.925729	0.112514
H	3.763094	0.875257	-2.755796
H	6.039692	0.670210	-1.764964
H	0.633369	-1.814720	-1.367099
Br	-0.846146	-0.705567	-3.289783

Statistical Thermodynamic Analysis

Temperature= 323.150 Kelvin Pressure= 1.00000 Atm

==

SCF Energy= -4256.42191774 Predicted Change= -3.161206D-08

Zero-point correction (ZPE)= -4255.8760 0.54582

Internal Energy (U)= -4255.8400 0.58190

Enthalpy (H)= -4255.8389 0.58293

Gibbs Free Energy (G)= -4255.9495 0.47233

Entropy (S)= 0.00034223

Frequencies -- 14.8304 25.2624 35.1434

Supporting Information: **Concerted [2,3] TS (*re*)**

Using Gaussian 16: ES64L-G16RevA.03 25-Dec-2016

==

#m06/6-31G(d) scf=(maxcycle=300,direct,tight,xqc) density=current
opt=(maxcycle=250,modredundant) iop(1/8=18) Temperature=323.15
SCRF=(PCM,SOLVENT=acetonitrile)

Modredundant Input: B 4 44 F

Modredundant Input: B 3 49 F

Modredundant Input:

#m06/6-31G(d) scf=(direct,tight,maxcycle=300,xqc)

opt=(nofreeze,maxcycle=250,ts,calcfc,noeigentest) iop(1/8=18) freq=noraman

SCRF=(PCM,SOLVENT=acetonitrile) Temperature=323.15 geom=check guess=read

#N Geom=AllCheck Guess=TCheck SCRF=Check Test GenChk RM06/6-31G(d) Freq

----

Pointgroup= C1 Stoichiometry= C27H32BrN3OS C1[X(C27H32BrN3OS)] #Atoms= 65

Charge = 0 Multiplicity = 1

SCF Energy= -4256.36822578 Predicted Change= -1.891596D-07

==

Optimization completed. {Found 2 times}

Item	Max Val.		Criteria	Pass?	RMS Val.		Criteria	Pass?
Force	0.00009	||	0.00045	[YES]	0.00001	||	0.00030	[YES]
Displ	0.00799	||	0.00180	[NO]	0.00799	||	0.00180	[YES]

Atomic Coordinates (Angstroms)

Type	X	Y	Z
C	1.030727	0.638043	0.960420
N	-0.292620	0.057962	0.997065
C	1.934863	-0.033535	0.126924
N	3.243291	0.355466	-0.023201
C	4.156903	-0.668433	-0.568123
H	4.099233	-1.561675	0.074462
H	3.838171	-0.954853	-1.577744
C	3.964428	1.010278	1.094513
O	1.203780	1.642254	1.667010
C	-0.803591	-1.140214	0.281552
C	-0.253392	-2.429290	0.843780
C	-2.327210	-0.989682	0.467386
C	-1.252701	0.531963	1.782810

C	-3.561615	0.571928	2.175411
H	-4.321795	-0.143502	2.504148
H	-3.988865	1.209542	1.384928
C	-3.019397	1.389795	3.339444
H	-3.584917	2.310069	3.502094
H	-2.996031	0.809718	4.267100
S	-1.270639	1.839920	2.902969
C	0.180886	-2.543589	2.163997
C	0.631334	-3.767938	2.648682
C	0.649720	-4.884963	1.818950
C	0.216166	-4.773676	0.500224
C	-0.233061	-3.551420	0.012220
H	1.626007	-0.892384	-0.462056
H	3.733543	0.459052	2.017793
H	3.595288	2.030474	1.219701
C	5.450250	0.904477	0.731845
H	6.010055	0.493249	1.578893
H	5.883222	1.887330	0.513670
C	5.528769	-0.023492	-0.493913
H	5.736646	0.548505	-1.406071
H	6.316257	-0.778104	-0.400918
H	-0.580800	-1.067171	-0.790556
H	-2.822619	-1.950244	0.640379
H	-2.785722	-0.496534	-0.403320
H	0.182857	-1.672512	2.819893
H	0.972180	-3.847010	3.679435
H	1.004610	-5.841280	2.198944
H	0.232121	-5.643161	-0.154774
H	-0.584730	-3.448360	-1.017407
N	-2.400190	-0.129817	1.647255
C	3.305767	1.969367	-1.589257
H	4.136740	2.566304	-1.217678
H	3.567701	1.237381	-2.349232
C	2.011256	2.431553	-1.422563

H	1.894767	3.288714	-0.761038
C	0.868280	1.756054	-1.832318
C	-0.467540	2.225704	-1.437830
C	-1.620307	1.653691	-2.008855
C	-2.893727	2.073913	-1.644141
C	-3.060672	3.085431	-0.702578
C	-1.933202	3.657378	-0.116796
C	-0.660634	3.232112	-0.470733
C	0.941754	0.682922	-2.879777
H	0.594058	1.086742	-3.844082
H	0.300182	-0.185652	-2.673654
H	1.959536	0.314822	-3.032283
H	-1.539769	0.836488	-2.723599
H	-3.759961	1.597743	-2.103220
H	-4.057656	3.422038	-0.422385
H	-2.045016	4.436754	0.635399
H	0.189620	3.677238	0.039465
Br	-1.961789	-1.895486	-2.930153

Statistical Thermodynamic Analysis

Temperature= 323.150 Kelvin Pressure= 1.00000 Atm

==

SCF Energy= -4256.36822578 Predicted Change= -1.891596D-07

Zero-point correction (ZPE)= -4255.8257 0.54248

Internal Energy (U)= -4255.7899 0.57825

Enthalpy (H)= -4255.7889 0.57927

Gibbs Free Energy (G)= -4255.8963 0.47184

Entropy (S)= 0.00033245

Frequencies -- -173.1763 24.6861 34.7104

Supporting Information: **(*R*,*R*)-[2,3]-Intermediate**

Using Gaussian 16: ES64L-G16RevA.03 25-Dec-2016

==

#m06/6-31g(d) scf=(maxcycle=300,direct,vshift=200,tight,yqc)

density=current scrf=(pcm,solvent=ch3cn) opt=(gdiis,maxcycle=250)

freq=noraman temperature=323.15

#N Geom=AllCheck Guess=TCheck SCRF=Check GenChk RM06/6-31G(d) Freq

----

Pointgroup= C1 Stoichiometry= C27H32BrN3OS C1[X(C27H32BrN3OS)] #Atoms= 65

Charge = 0 Multiplicity = 1

SCF Energy= -4256.40404791 Predicted Change= -1.682088D-08

==

Optimization completed. {Found 2 times}

Item	Max Val.	Criteria	Pass?	RMS Val.	Criteria	Pass?
Force	0.00000 ||	0.00045	[YES]	0.00000 ||	0.00030	[YES]
Displ	0.00175 ||	0.00180	[YES]	0.00175 ||	0.00180	[YES]

Atomic Type	Coordinates (Angstroms) X	Y	Z
C	-0.269644	-0.342578	-1.178922
N	-0.890328	0.655396	-0.376722
C	0.793236	-1.197289	-0.497636
N	2.045501	-1.273400	-1.197644
C	2.913494	-0.108105	-1.030920
H	2.414434	0.840222	-1.331672
H	3.195745	-0.009423	0.028387
C	2.166146	-1.788460	-2.571165
O	-0.693253	-0.508881	-2.304357
C	-0.319669	1.450080	0.756502
C	0.660055	2.475956	0.235390
C	-1.599241	2.055565	1.369014
C	-2.112963	1.133728	-0.658750
C	-3.971483	2.231159	0.244535

H	-4.195836	3.255452	0.555733
H	-4.434381	1.529695	0.955716
C	-4.413986	1.968128	-1.190238
H	-5.417059	1.540551	-1.245210
H	-4.369616	2.870600	-1.806436
S	-3.212890	0.751145	-1.917817
C	0.493750	3.095544	-1.005840
C	1.402191	4.056179	-1.437066
C	2.481771	4.407954	-0.631920
C	2.651573	3.791901	0.604335
C	1.745612	2.829670	1.038489
H	1.038452	-0.739680	0.471470
H	1.236774	-1.677802	-3.146534
H	2.409099	-2.862034	-2.548382
C	3.302648	-0.965930	-3.188075
H	2.884884	-0.131271	-3.769369
H	3.929749	-1.557078	-3.865174
C	4.054344	-0.409115	-1.981637
H	4.711479	-1.175347	-1.546377
H	4.661921	0.471955	-2.217111
H	0.179641	0.793296	1.484657
H	-1.448823	3.084192	1.710054
H	-1.995619	1.445247	2.194158
H	-0.332412	2.820223	-1.662105
H	1.267204	4.527285	-2.408725
H	3.195245	5.155812	-0.972983
H	3.502470	4.052102	1.231320
H	1.907267	2.296440	1.976489
N	-2.530539	2.004886	0.242671
C	-0.181170	-3.397749	-1.357962
C	0.147948	-2.589235	-0.122011
C	0.054288	-4.695754	-1.532543
H	0.547812	-5.315194	-0.785201
H	-0.242705	-5.197289	-2.451835

H	-0.679526	-2.854273	-2.162923
C	1.155466	-3.326258	0.763674
H	0.685582	-4.196232	1.240451
H	2.003448	-3.672283	0.161578
H	1.556070	-2.669677	1.546314
C	-1.138702	-2.260070	0.655710
C	-2.405392	-2.365762	0.071482
C	-1.060951	-1.740755	1.955637
C	-3.550161	-1.940462	0.743019
H	-2.512834	-2.778019	-0.930203
C	-2.203550	-1.322040	2.629598
H	-0.092548	-1.608457	2.440968
C	-3.455748	-1.409467	2.024358
H	-4.519752	-2.024984	0.253972
H	-2.111140	-0.918932	3.637820
H	-4.348488	-1.076489	2.551539
Br	2.226118	-0.277002	2.874545

Statistical Thermodynamic Analysis

Temperature= 323.150 Kelvin Pressure= 1.00000 Atm

==

SCF Energy= -4256.40404791 Predicted Change= -1.682088D-08

Zero-point correction (ZPE)= -4255.8602 0.54376

Internal Energy (U)= -4255.8239 0.58010

Enthalpy (H)= -4255.8229 0.58112

Gibbs Free Energy (G)= -4255.9323 0.47169

Entropy (S)= 0.00033863

Frequencies -- 30.8762 31.5206 40.0939

Supporting Information: **(*S*,*R*)-[2,3]-Intermediate**

Using Gaussian 16: ES64L-G16RevA.03 25-Dec-2016

==

#m06/6-31g(d) scf=(maxcycle=300,direct,vshift=200,tight,yqc)

density=current scrf=(pcm,solvent=ch3cn) opt=(gdiis,maxcycle=250)

freq=noraman temperature=323.15

#N Geom=AllCheck Guess=TCheck SCRF=Check GenChk RM06/6-31G(d) Freq

----

Pointgroup= C1 Stoichiometry= C27H32BrN3OS C1[X(C27H32BrN3OS)] #Atoms= 65

Charge = 0 Multiplicity = 1

SCF Energy= -4256.40552994 Predicted Change= -1.278676D-07

==

Optimization completed. {Found 1 times}

Item	Max Val.		Criteria	Pass?	RMS Val.		Criteria	Pass?
Force	0.00003	||	0.00045	[YES]	0.00000	||	0.00030	[YES]
Displ	0.00305	||	0.00180	[NO]	0.00305	||	0.00180	[YES]

Atomic Type	Coordinates (Angstroms) X	Y	Z
C	-0.421233	-0.025233	-1.256120
N	-1.003911	0.717489	-0.199219
C	0.777103	-0.877260	-0.891105
N	1.912715	-0.603137	-1.749601
C	3.221478	-0.919138	-1.136708
H	3.119769	-1.075732	-0.050093
H	3.624725	-1.849473	-1.566177
C	1.989146	0.722319	-2.376045
O	-0.965528	-0.006746	-2.339310
C	-0.367226	1.254919	1.059335
C	0.587375	2.371471	0.710214
C	-1.613668	1.677437	1.861615
C	-2.261788	1.185215	-0.277885
C	-4.068742	2.003110	0.968339
H	-4.279030	2.930343	1.509161

H	-4.474614	1.155292	1.539845
C	-4.606057	2.047031	-0.456936
H	-5.608243	1.621686	-0.538527
H	-4.607722	3.062596	-0.863261
S	-3.448793	1.041575	-1.507192
C	0.159199	3.489664	-0.013744
C	1.060697	4.487205	-0.361664
C	2.398822	4.381957	0.014201
C	2.825933	3.279657	0.745624
C	1.925437	2.274411	1.092358
H	1.043416	-0.685318	0.163064
H	1.664279	1.532008	-1.690127
H	1.352581	0.772026	-3.269110
C	3.465438	0.886624	-2.685169
H	3.741077	1.933893	-2.857352
H	3.729195	0.310292	-3.583643
C	4.115649	0.278677	-1.449391
H	5.165738	-0.000504	-1.593265
H	4.070874	1.002081	-0.621162
H	0.169942	0.454623	1.588469
H	-1.464620	2.622216	2.393202
H	-1.931194	0.902693	2.574178
H	-0.883243	3.586967	-0.318838
H	0.718254	5.350063	-0.929500
H	3.104839	5.162987	-0.261736
H	3.868958	3.193863	1.046564
H	2.257477	1.397060	1.653338
N	-2.629559	1.819177	0.820250
C	1.299125	-3.237598	-0.189828
C	0.290653	-2.392841	-0.945238
C	1.856844	-4.367633	-0.618549
H	1.659335	-4.799528	-1.598533
H	2.551866	-4.916609	0.014805
H	1.568685	-2.866138	0.803504

C	0.179075	-2.850112	-2.393208
H	1.170455	-2.836680	-2.857924
H	-0.219315	-3.871249	-2.448644
H	-0.469649	-2.193537	-2.980513
C	-1.049954	-2.466187	-0.194897
C	-2.255936	-2.690221	-0.866477
C	-1.106877	-2.233520	1.188221
C	-3.474968	-2.669468	-0.192146
H	-2.258396	-2.873015	-1.938741
C	-2.325022	-2.201735	1.859440
H	-0.194601	-2.046299	1.759280
C	-3.518134	-2.416019	1.173659
H	-4.395624	-2.845118	-0.746586
H	-2.338128	-2.012058	2.932359
H	-4.470241	-2.392848	1.701069
Br	2.099118	-1.023683	2.730201

Statistical Thermodynamic Analysis

Temperature= 323.150 Kelvin Pressure= 1.00000 Atm

==

SCF Energy= -4256.40552994 Predicted Change= -1.278676D-07

Zero-point correction (ZPE)= -4255.8602 0.54530

Internal Energy (U)= -4255.8245 0.58096

Enthalpy (H)= -4255.8235 0.58198

Gibbs Free Energy (G)= -4255.9298 0.47571

Entropy (S)= 0.00032886

Frequencies -- 29.8171 37.1284 53.1679

Supporting Information: **(*R*)-[1,2]-Product (major)**

Using Gaussian 16: ES64L-G16RevA.03 25-Dec-2016

==

m06/6-31g(d) gfprint gfinput scf=(direct,tight,maxcycle=300,xqc)

opt=(maxcycle=250) freq=noraman scrf=(pcm,solvent=acetonitrile)

iop(1/8=18) temperature=323.15

#N Geom=AllCheck Guess=TCheck SCRF=Check Test GenChk RM06/6-31G(d) Freq

----

Pointgroup= C1 Stoichiometry= C22H24N2O4 C1[X(C22H24N2O4)] #Atoms= 52

Charge = 0 Multiplicity = 1

SCF Energy= -1262.25033100 Predicted Change= -4.621875D-09

==

Optimization completed. {Found 1 times}

Item	Max Val.	Criteria	Pass?	RMS Val.	Criteria	Pass?
Force	0.00000 ||	0.00045	[YES]	0.00000 ||	0.00030	[YES]
Displ	0.00303 ||	0.00180	[NO]	0.00303 ||	0.00180	[YES]

Atomic Type	Coordinates (Angstroms) X	Y	Z
C	1.153113	-1.095581	1.067439
C	2.561856	-0.567403	0.892829
H	3.073514	-0.801261	1.853058
N	3.211907	-1.226399	-0.220763
C	3.283208	-2.677679	-0.050399
H	2.289078	-3.134605	-0.154776
H	3.664380	-2.932020	0.962291
C	4.268919	-3.122051	-1.125281
H	4.821332	-4.016970	-0.819044
H	3.737430	-3.368404	-2.052066
C	5.180264	-1.894413	-1.331508
H	5.143973	-1.555167	-2.373265
H	6.229625	-2.103103	-1.095928
C	4.608200	-0.827772	-0.397489
H	5.139187	-0.838096	0.579086
H	4.682955	0.187183	-0.806155

O	0.406285	-0.856245	-0.047972
C	-0.951178	-1.080770	-0.019975
O	0.726059	-1.638968	2.051775
C	-1.752691	-0.555215	0.992202
H	-1.300815	-0.002346	1.812056
C	-3.123019	-0.741115	0.928839
H	-3.779181	-0.344148	1.697053
C	-3.657959	-1.444724	-0.146243
N	-5.102768	-1.638102	-0.212240
O	-5.785719	-1.186564	0.696975
O	-5.559102	-2.241839	-1.173505
C	-2.863517	-1.964964	-1.161524
H	-3.319161	-2.505624	-1.984906
C	-1.492184	-1.780095	-1.093251
H	-0.830704	-2.167415	-1.863655
C	1.597792	1.584997	1.730141
C	2.495061	0.955515	0.710271
H	2.149019	1.160213	-0.311040
H	3.516406	1.356565	0.796450
H	1.808987	1.308509	2.768613
C	0.539328	2.381690	1.508058
C	-0.315736	2.856884	2.647889
H	0.044990	2.470570	3.608348
H	-1.362277	2.539786	2.530684
H	-0.332451	3.954702	2.698515
C	0.154878	2.866004	0.158561
C	1.089659	3.488065	-0.677703
H	2.112109	3.620436	-0.324250
C	0.724471	3.957039	-1.935129
H	1.467065	4.441946	-2.566567
C	-0.586047	3.813700	-2.381021
H	-0.873170	4.179077	-3.365342
C	-1.529289	3.206153	-1.556642
H	-2.557497	3.090942	-1.895542

C -1.164303 2.746516 -0.296300

H -1.913904 2.270994 0.336632

Statistical Thermodynamic Analysis

Temperature= 323.150 Kelvin Pressure= 1.00000 Atm

===

SCF Energy= -1262.25033100 Predicted Change= -4.621875D-09

Zero-point correction (ZPE)= -1261.8244 0.42586

Internal Energy (U)= -1261.7948 0.45551

Enthalpy (H)= -1261.7937 0.45654

Gibbs Free Energy (G)= -1261.8917 0.35857

Entropy (S)= 0.00030316

Frequencies -- 12.4484 23.7159 29.3690

Supporting Information: ***(R,R)*-[2,3]-Product (minor)**

Using Gaussian 16: ES64L-G16RevA.03 25-Dec-2016

===

#m06/6-31g(d) scf=(maxcycle=300,direct,vshift=200,tight,yqc)

density=current scrf=(pcm,solvent=ch3cn) opt=(gdiis,maxcycle=250)

freq=noraman temperature=323.15

#N Geom=AllCheck Guess=TCheck SCRF=Check GenChk RM06/6-31G(d) Freq

----

Pointgroup= C1 Stoichiometry= C22H24N2O4 C1[X(C22H24N2O4)] #Atoms= 52

Charge = 0 Multiplicity = 1

SCF Energy= -1262.23476283 Predicted Change= -1.083737D-09

==

Optimization completed. {Found 2 times}

Item Max Val. Criteria Pass? RMS Val. Criteria Pass?

Force 0.00000 || 0.00045 [YES] 0.00000 || 0.00030 [YES]

Displ 0.00046 || 0.00180 [YES] 0.00046 || 0.00180 [YES]

Atomic Type	Coordinates (Angstroms) X	Y	Z
C	3.803380	1.389089	2.348897
C	2.777513	0.892853	1.661652
C	2.547096	1.038601	0.171718
C	1.475834	2.113471	-0.090197
C	0.664458	2.616798	0.930522
C	-0.329536	3.556669	0.665474
C	-0.536757	4.012568	-0.631122
C	0.260196	3.517245	-1.660755
C	1.250615	2.580358	-1.391438
C	3.850310	1.392384	-0.548206
C	1.989345	-0.278823	-0.461166
N	2.882388	-1.417080	-0.442171
C	3.259644	-2.065812	0.814325
C	4.076125	-3.259623	0.329399
C	3.439848	-3.635270	-1.024594
C	2.433405	-2.514837	-1.296939
C	0.606435	-0.598441	0.093513
O	-0.342759	-0.390273	-0.866390
C	-1.670573	-0.521507	-0.511783
C	-2.435586	0.638167	-0.467467
C	-3.787394	0.541824	-0.177395
C	-4.328544	-0.716037	0.065152
N	-5.753008	-0.821916	0.371146
O	-6.213728	-1.934308	0.586713
O	-6.413566	0.207197	0.396477
C	-3.564648	-1.878184	0.025619
C	-2.214559	-1.778049	-0.267610
O	0.339146	-0.997539	1.199364
H	1.832593	-0.048220	-1.525378
H	4.619230	1.940738	1.884165

H	3.867953	1.255256	3.427078
H	2.002899	0.349100	2.206618
H	0.812045	2.284957	1.956188
H	-0.942141	3.933054	1.483032
H	-1.310089	4.749583	-0.839676
H	0.112825	3.864542	-2.681974
H	1.855218	2.209614	-2.218415
H	4.179939	2.403393	-0.279048
H	4.640765	0.681578	-0.282653
H	3.730735	1.359300	-1.637208
H	3.828058	-1.382364	1.453663
H	2.371609	-2.405774	1.380697
H	5.123060	-2.963473	0.189601
H	4.061523	-4.082926	1.052191
H	2.941969	-4.610952	-0.994794
H	4.198175	-3.683784	-1.814466
H	1.410655	-2.851423	-1.019366
H	2.396844	-2.191405	-2.346185
H	-1.960027	1.598556	-0.663484
H	-4.420473	1.422440	-0.135231
H	-4.029665	-2.839285	0.220537
H	-1.581753	-2.660797	-0.313614

Statistical Thermodynamic Analysis

Temperature= 323.150 Kelvin Pressure= 1.00000 Atm

==

SCF Energy= -1262.23476283 Predicted Change= -1.083737D-09

Zero-point correction (ZPE)= -1261.8094 0.42534

Internal Energy (U)= -1261.7798 0.45496

Enthalpy (H)= -1261.7787 0.45598

Gibbs Free Energy (G)= -1261.8745 0.36023

Entropy (S)= 0.00029629

Frequencies -- 25.9121 28.7758 34.5020

Supporting Information: **(*S*,*R*)-[2,3]-Product (minor)**

Using Gaussian 16: ES64L-G16RevA.03 25-Dec-2016

==

#m06/6-31g(d) scf=(maxcycle=300,direct,vshift=200,tight,yqc)

density=current scrf=(pcm,solvent=ch3cn) opt=(gdiis,maxcycle=250)

freq=noraman temperature=323.15

#N Geom=AllCheck Guess=TCheck SCRF=Check GenChk RM06/6-31G(d) Freq

----

Pointgroup= C1 Stoichiometry= C22H24N2O4 C1[X(C22H24N2O4)] #Atoms= 52

Charge = 0 Multiplicity = 1

SCF Energy= -1262.23442631 Predicted Change= -4.079384D-10

==

Optimization completed. {Found 2 times}

Item	Max Val.		Criteria	Pass?	RMS Val.		Criteria	Pass?
Force	0.00000	||	0.00045	[YES]	0.00000	||	0.00030	[YES]
Displ	0.00055	||	0.00180	[YES]	0.00055	||	0.00180	[YES]

Atomic Type	Coordinates (Angstroms) X	Y	Z
C	-0.203865	-2.222191	1.557676
C	-1.169884	-1.359754	1.248724
C	-1.700349	-1.054034	-0.135761
C	-1.184186	-2.067438	-1.167670
C	-3.225955	-1.154034	-0.167020
C	-3.961771	-0.590946	-1.216259
C	-5.338902	-0.753706	-1.292947
C	-6.015576	-1.490388	-0.322455
C	-5.296724	-2.067661	0.716201
C	-3.914472	-1.901378	0.789206

C	-1.245459	0.404569	-0.545951
N	-2.116227	1.452186	-0.111118
C	-2.032221	2.707962	-0.841986
C	-2.889965	3.641779	-0.006899
C	-2.515943	3.218190	1.410256
C	-2.381929	1.698569	1.309270
C	0.201756	0.672061	-0.131730
O	1.072547	-0.079129	-0.872384
C	2.415174	-0.072554	-0.561602
C	3.300015	0.146355	-1.612112
C	4.663534	0.087786	-1.374773
C	5.104248	-0.186734	-0.085018
N	6.539603	-0.247312	0.171217
O	7.300312	-0.059792	-0.768492
O	6.910775	-0.482629	1.313021
C	4.223756	-0.410628	0.968865
C	2.861626	-0.355507	0.727652
O	0.566721	1.465046	0.695422
H	-1.242142	0.428247	-1.649384
H	0.301954	-2.836390	0.813374
H	0.121020	-2.350773	2.588642
H	-1.629287	-0.789405	2.059751
H	-1.507056	-3.077831	-0.885455
H	-1.603340	-1.844846	-2.156937
H	-0.093104	-2.063108	-1.255054
H	-3.455620	-0.012041	-1.988218
H	-5.888520	-0.300745	-2.116656
H	-7.095446	-1.615098	-0.381393
H	-5.807791	-2.654672	1.477718
H	-3.365107	-2.373448	1.603180
H	-0.994902	3.095558	-0.906054
H	-2.399713	2.575479	-1.872162
H	-2.695627	4.700395	-0.211955
H	-3.953824	3.443226	-0.200187

H	-1.539953	3.652283	1.672270
H	-3.238541	3.532751	2.171674
H	-3.309809	1.182891	1.614337
H	-1.572441	1.336121	1.957150
H	2.908165	0.357622	-2.603536
H	5.382232	0.253429	-2.170961
H	4.609682	-0.629776	1.959391
H	2.145263	-0.533869	1.525231

Statistical Thermodynamic Analysis

Temperature= 323.150 Kelvin   Pressure= 1.00000 Atm

==

SCF Energy=   -1262.23442631   Predicted Change= -4.079384D-10

Zero-point correction (ZPE)=   -1261.8091   0.42527

Internal Energy (U)=   -1261.7798   0.45460

Enthalpy (H)=   -1261.7787   0.45562

Gibbs Free Energy (G)=   -1261.8741   0.36023

Entropy (S)=   0.00029521

Frequencies --   17.2121   22.0394   28.6469

Supporting Information: **PNPO^{-}**

Using Gaussian 16: ES64L-G16RevA.03 25-Dec-201

==

m06/6-31g(d) gfprint gfinput scf=(direct,tight,maxcycle=300,xqc)

opt=(maxcycle=250) freq=noraman scrf=(pcm,solvent=acetonitrile)

iop(1/8=18) temperature=323.15

#N Geom=AllCheck Guess=TCheck SCRF=Check Test GenChk RM06/6-31G(d) Freq

----

Pointgroup= C1   Stoichiometry= C6H4NO3(1-)   C1[X(C6H4NO3)] #Atoms= 14

Charge = -1   Multiplicity = 1

SCF Energy= -511.209546086 Predicted Change= -7.944908D-07

==

Optimization completed. {Found 1 times}

Item	Max Val.		Criteria	Pass?	RMS Val.		Criteria	Pass?
Force	0.00022	||	0.00045	[YES]	0.00008	||	0.00030	[YES]
Displ	0.00190	||	0.00180	[NO]	0.00190	||	0.00180	[YES]

Atomic Type	Coordinates (Angstroms) X	Y	Z
C	-1.415430	1.222570	0.000029
H	-1.969140	2.161940	-0.000001
C	-0.049070	1.221080	0.000029
H	0.515900	2.150710	0.000009
C	0.660490	0.000000	0.000039
N	2.066360	0.000000	0.000019
O	2.675370	-1.084300	-0.000081
C	-0.049070	-1.221080	0.000029
H	0.515900	-2.150710	-0.000001
C	-1.415430	-1.222570	0.000029
H	-1.969140	-2.161940	-0.000011
C	-2.195830	0.000000	0.000109
O	-3.447240	0.000000	-0.000051
O	2.675370	1.084300	-0.000081

Statistical Thermodynamic Analysis

Temperature= 323.150 Kelvin Pressure= 1.00000 Atm

==

SCF Energy= -511.209546086 Predicted Change= -7.944908D-07

Zero-point correction (ZPE)= -511.1150 0.09454

Internal Energy (U)= -511.1061 0.10339

Enthalpy (H)= -511.1051 0.10441

Gibbs Free Energy (G)= -511.1515 0.05800

Entropy (S)= 0.00014363

Frequencies -- 80.2515 100.3539 232.2018

Supporting Information: **PNPOH**

Using Gaussian 16: ES64L-G16RevA.03 25-Dec-2016

==

m06/6-31g(d) gfprint gfinput scf=(direct,tight,maxcycle=300,xqc)

opt=(maxcycle=250) freq=noraman scrf=(pcm,solvent=acetonitrile)

iop(1/8=18) temperature=323.15

#N Geom=AllCheck Guess=TCheck SCRF=Check Test GenChk RM06/6-31G(d) Freq

----

Pointgroup= C1 Stoichiometry= C6H5NO3 C1[X(C6H5NO3)] #Atoms= 15

Charge = 0 Multiplicity = 1

SCF Energy= -511.679063312 Predicted Change= -5.586288D-07

==

Optimization completed. {Found 1 times}

Item	Max Val.		Criteria	Pass?	RMS Val.		Criteria	Pass?
Force	0.00018	||	0.00045	[YES]	0.00006	||	0.00030	[YES]
Displ	0.00217	||	0.00180	[NO]	0.00217	||	0.00180	[YES]

Atomic Type	Coordinates (Angstroms) X	Y	Z
C	1.366037	1.227611	0.000054
H	1.925436	2.159717	0.000033
C	-0.013945	1.221101	0.000109
H	-0.577412	2.148869	0.000137
N	-2.137692	-0.007573	0.000166
C	-0.688782	0.000436	0.000133
C	-0.002071	-1.211302	0.000078
H	-0.554611	-2.145629	0.000089

C	1.380154	-1.203275	0.000032
H	1.933788	-2.140726	-0.000008
C	2.068754	0.015635	0.000016
O	3.409531	0.087916	-0.000036
H	3.793985	-0.803102	-0.000063
O	-2.724132	1.069316	-0.000210
O	-2.712676	-1.090652	-0.000240

Statistical Thermodynamic Analysis

Temperature= 323.150 Kelvin Pressure= 1.00000 Atm

==

SCF Energy= -511.679063312 Predicted Change= -5.586288D-07

Zero-point correction (ZPE)= -511.5714 0.10763

Internal Energy (U)= -511.5622 0.11680

Enthalpy (H)= -511.5612 0.11783

Gibbs Free Energy (G)= -511.6082 0.07086

Entropy (S)= 0.00014536

Frequencies -- 64.9477 113.1560 231.0322

Supporting Information: **Br¯**

Using Gaussian 16: ES64L-G16RevA.03 25-Dec-2016

==

m06/6-31g(d) gfprint gfinput scf=(direct,tight,maxcycle=300,xqc)

opt=(maxcycle=250) freq=noraman scrf=(pcm,solvent=acetonitrile)

iop(1/8=18) temperature=323.15

#N Geom=AllCheck Guess=TCheck SCRF=Check Test GenChk RM06/6-31G(d) Freq

----

Pointgroup= OH Stoichiometry= Br(1-) OH[O(Br)] #Atoms= 1

Charge = -1 Multiplicity = 1

SCF Energy= -2571.70514867 Predicted Change= -0.000000D+00

==

Optimization completed. {Found 2 times}

Item Max Val. Criteria Pass? RMS Val. Criteria Pass?

Force 0.00000 || 0.00045 [YES] 0.00000 || 0.00030 [YES]

Displ 0.00000 || 0.00180 [YES] 0.00000 || 0.00180 [YES]

Atomic Coordinates (Angstroms)

Type X Y Z

Br 0.000000 0.000000 0.000000

Statistical Thermodynamic Analysis

Temperature= 323.150 Kelvin Pressure= 1.00000 Atm

==

SCF Energy= -2571.70514867 Predicted Change= -0.000000D+00

Zero-point correction (ZPE)= -2571.7051 0.00000

Internal Energy (U)= -2571.7036 0.00153

Enthalpy (H)= -2571.7025 0.00255

Gibbs Free Energy (G)= -2571.7228 -0.01773

Entropy (S)= 6.281e-05

Supporting Information: **Et_3N**

Using Gaussian 16: ES64L-G16RevA.03 25-Dec-2016

==

m06/6-31G(d) gfprint gfinput scf=(direct,tight,maxcycle=300,xqc)

opt=(maxcycle=250) freq=noraman SCRF=(PCM,SOLVENT=acetonitrile)

iop(1/8=18) Temperature=323.15

#N Geom=AllCheck Guess=TCheck SCRF=Check Test GenChk RM06/6-31G(d) Freq

----

Pointgroup= C1 Stoichiometry= C6H15N C1[X(C6H15N)] #Atoms= 22

Charge = 0 Multiplicity = 1

SCF Energy= -292.179158900 Predicted Change= -1.967410D-08

==

Optimization completed. {Found 2 times}

Item	Max Val.	Criteria	Pass?	RMS Val.	Criteria	Pass?
Force	0.00004 ||	0.00045	[YES]	0.00000 ||	0.00030	[YES]
Displ	0.00058 ||	0.00180	[YES]	0.00058 ||	0.00180	[YES]

Atomic Type	Coordinates (Angstroms) X	Y	Z
N	-0.000006	-0.417916	0.284824
C	-0.000050	0.995641	0.655679
H	-0.873052	1.174104	1.298396
H	0.872858	1.174126	1.298513
C	1.191695	-0.833927	-0.445015
H	1.100244	-1.919136	-0.607249
H	1.232029	-0.383700	-1.460665
C	-1.191681	-0.833951	-0.445025
H	-1.100223	-1.919162	-0.607241
H	-1.232008	-0.383733	-1.460679
C	0.000021	1.998474	-0.492883
H	-0.000020	3.023440	-0.101814
H	0.886821	1.888812	-1.131650
H	-0.886682	1.888789	-1.131780
C	2.490517	-0.553624	0.286149
H	3.322040	-1.062938	-0.215251
H	2.734372	0.515996	0.315168
H	2.438763	-0.919096	1.320200
C	-2.490500	-0.553624	0.286132
H	-2.734295	0.516008	0.315214
H	-3.322040	-1.062866	-0.215313
H	-2.438782	-0.919161	1.320162

Statistical Thermodynamic Analysis

Temperature= 323.150 Kelvin   Pressure= 1.00000 Atm

==

SCF Energy=   -292.179158900   Predicted Change= -1.967410D-08

Zero-point correction (ZPE)=   -291.9734   0.20572

Internal Energy (U)=   -291.9629   0.21621

Enthalpy (H)=   -291.9619   0.21724

Gibbs Free Energy (G)=   -292.0100   0.16912

Entropy (S)=   0.0001489

Frequencies --   109.9994   116.8031   145.5594

Supporting Information: **HBr**

Using Gaussian 16: ES64L-G16RevA.03 25-Dec-2016

==

#m06/6-31g(d) scf=(maxcycle=300,direct,vshift=200,tight,yqc)

density=current scrf=(pcm,solvent=ch3cn) opt=(gdiis,maxcycle=250)

freq=noraman temperature=323.15

#N Geom=AllCheck Guess=TCheck SCRF=Check GenChk RM06/6-31G(d) Freq

----

Pointgroup= C*V   Stoichiometry= BrH   C*V[C*(HBr)] #Atoms= 2

Charge = 0   Multiplicity = 1

SCF Energy= -2572.13806447 Predicted Change= -1.666293D-07

==

Optimization completed.   {Found   2   times}

Item   Max Val.   Criteria   Pass?   RMS Val.   Criteria   Pass?

Force   0.00029 || 0.00045   [YES]   0.00029 || 0.00030   [YES]

Displ   0.00056 || 0.00180   [YES]   0.00056 || 0.00180   [YES]

Atomic   Coordinates (Angstroms)

Type   X   Y   Z

Br 0.000000 0.000000 0.039907

H 0.000000 0.000000 -1.396731

Statistical Thermodynamic Analysis

Temperature= 323.150 Kelvin Pressure= 1.00000 Atm

===

SCF Energy= -2572.13806447 Predicted Change= -1.666293D-07

Zero-point correction (ZPE)= -2572.1320 0.00601

Internal Energy (U)= -2572.1294 0.00857

Enthalpy (H)= -2572.1284 0.00959

Gibbs Free Energy (G)= -2572.1531 -0.01513

Entropy (S)= 7.652e-05

Frequencies -- 2639.8294

Supporting Information: **Et3NH—Br Complex**

Using Gaussian 16: ES64L-G16RevA.03 25-Dec-2016

===

#m06/6-31G(d) scf=(maxcycle=300,direct,tight,xqc) freq=noraman

density=current opt=(maxcycle=250) iop(1/8=18) Temperature=323.15

SCRF=(PCM,SOLVENT=acetonitrile)

#N Geom=AllCheck Guess=TCheck SCRF=Check Test GenChk RM06/6-31G(d) Freq

----

Pointgroup= C1 Stoichiometry= C6H16BrN C1[X(C6H16BrN)] #Atoms= 24

Charge = 0 Multiplicity = 1

SCF Energy= -2864.37401929 Predicted Change= -1.202059D-07

===

Optimization completed. {Found 1 times}

Item	Max Val.		Criteria	Pass?	RMS Val.		Criteria	Pass?
Force	0.00014	||	0.00045	[YES]	0.00003	||	0.00030	[YES]
Displ	0.02001	||	0.00180	[NO]	0.02001	||	0.00180	[NO]

Atomic Type	Coordinates (Angstroms) X	Y	Z
N	-0.896758	0.000200	0.270046
C	-1.181783	1.233236	1.072711
C	-0.757199	2.498950	0.364562
C	-1.182390	-1.231987	1.073734
C	-0.762278	-2.498637	0.364304
H	-0.612896	1.106828	2.001739
H	-1.406126	2.750179	-0.481853
H	0.277983	2.407472	0.009156
H	-0.801424	3.333515	1.072063
H	0.272215	-2.409719	0.006573
H	-1.413847	-2.748156	-0.480730
H	-0.807065	-3.333154	1.071765
H	-2.247230	-1.223597	1.335299
H	0.150682	-0.000144	0.111622
Br	2.237337	-0.000837	-0.203979
C	-1.490677	0.000105	-1.104747
H	-1.078043	-0.876701	-1.614770
H	-1.076863	0.876120	-1.615197
C	-2.999949	0.001428	-1.116901
H	-3.344333	0.000836	-2.156210
H	-3.420085	-0.887773	-0.631780
H	-3.418435	0.892765	-0.633652
H	-0.611040	-1.106216	2.001473
H	-2.247325	1.227059	1.331462

Statistical Thermodynamic Analysis

Temperature= 323.150 Kelvin Pressure= 1.00000 Atm

==

SCF Energy= -2864.37401929 Predicted Change= -1.202059D-07

Zero-point correction (ZPE)= -2864.1529 0.22108

Internal Energy (U)= -2864.1398 0.23413

Enthalpy (H)= -2864.1388 0.23515

Gibbs Free Energy (G)= -2864.1973 0.17671

Entropy (S)= 0.00018085

Frequencies -- 9.0560 66.8344 101.1487

Supporting Information: **PNPOH—NEt$_3$ Complex**

Using Gaussian 16: ES64L-G16RevA.03 25-Dec-2016

==

#m06/6-31G(d) scf=(maxcycle=300,direct,tight,xqc) freq=noraman

density=current opt=(maxcycle=250) iop(1/8=18) Temperature=323.15

SCRF=(PCM,SOLVENT=acetonitrile)

#N Geom=AllCheck Guess=TCheck SCRF=Check Test GenChk RM06/6-31G(d) Freq

----

Pointgroup= C1 Stoichiometry= C12H20N2O3 C1[X(C12H20N2O3)] #Atoms= 37

Charge = 0 Multiplicity = 1

SCF Energy= -803.878263139 Predicted Change= -2.958011D-08

==

Optimization completed. {Found 1 times}

Item	Max Val.		Criteria	Pass?	RMS Val.		Criteria	Pass?
Force	0.00001	||	0.00045	[YES]	0.00000	||	0.00030	[YES]
Displ	0.00296	||	0.00180	[NO]	0.00296	||	0.00180	[YES]

Atomic Type	Coordinates (Angstroms) X	Y	Z
N	-2.636250	0.093256	-0.072238
C	-2.556499	0.780096	-1.368518
C	-3.784215	1.544142	-1.837832
C	-2.698417	0.996795	1.078814

C	-2.348647	0.295540	2.377818
H	-1.700976	1.470111	-1.305606
H	-4.134856	2.258011	-1.081161
H	-4.613532	0.871862	-2.088313
H	-3.540457	2.114142	-2.742671
H	-3.099383	-0.452176	2.662092
H	-2.284287	1.022790	3.195026
H	-1.379487	-0.216450	2.300389
H	-3.684719	1.485660	1.164632
C	3.093725	-1.338673	-0.345177
C	1.810443	-1.836756	-0.412172
C	0.707870	-0.997632	-0.169207
O	-0.506746	-1.526496	-0.246982
C	0.925762	0.355760	0.148363
C	2.210241	0.856953	0.215702
C	3.288698	0.008483	-0.032074
N	4.631754	0.532857	0.037818
O	4.782096	1.720120	0.313379
O	5.568435	-0.230298	-0.181565
H	3.953074	-1.975583	-0.532473
H	1.624142	-2.880381	-0.654566
H	-1.231698	-0.825940	-0.101097
H	0.074609	1.006317	0.346511
H	2.391642	1.899349	0.459877
H	-2.282336	0.019193	-2.115623
H	-1.976268	1.806050	0.890762
C	-3.589708	-1.030575	-0.024672
H	-3.199909	-1.767213	0.695597
H	-3.541772	-1.519702	-1.008371
C	-5.034027	-0.712326	0.333345
H	-5.627742	-1.633296	0.288521
H	-5.127037	-0.316194	1.352328
H	-5.489164	0.013219	-0.349921

Statistical Thermodynamic Analysis

Temperature= 323.150 Kelvin   Pressure= 1.00000 Atm

==

SCF Energy=   -803.878263139   Predicted Change= -2.958011D-08

Zero-point correction (ZPE)=   -803.5634   0.31483

Internal Energy (U)=   -803.5420   0.33623

Enthalpy (H)=   -803.5410   0.33726

Gibbs Free Energy (G)=   -803.6177   0.26055

Entropy (S)=   0.00023737

Frequencies --   21.4863   33.8816   43.4879

Supporting Information: **Desmethyl C-N Homolysis TS (*re*)**

Using Gaussian 16: ES64L-G16RevA.03 25-Dec-2016

==

#um06/6-31G(d) scf=(direct,tight,maxcycle=300,xqc) guess=(mix,always)

opt=(maxcycle=250,ts,calcfc,noeigentest) iop(1/8=18) freq=noraman

SCRF=(PCM,SOLVENT=acetonitrile) Temperature=323.15

#N Geom=AllCheck Guess=TCheck SCRF=Check Test GenChk UM06/6-31G(d) Freq

----

Pointgroup= C1   Stoichiometry= C26H30BrN3OS   C1[X(C26H30BrN3OS)] #Atoms= 62

Charge = 0   Multiplicity = 1

SCF Energy= -4217.09250325 Predicted Change= -1.548897D-09

==

Optimization completed.   {Found   2   times}

Item	Max Val.	Criteria	Pass?	RMS Val.	Criteria	Pass?
Force	0.00000 ||	0.00045	[YES]	0.00000 ||	0.00030	[YES]
Displ	0.00124 ||	0.00180	[YES]	0.00124 ||	0.00180	[YES]

Atomic   Coordinates (Angstroms)

Type   X   Y   Z

C	-4.279207	1.421127	-0.350304
H	-4.324675	2.450210	-0.738227
H	-4.957574	1.336244	0.505603
C	-4.601802	0.384949	-1.420245
H	-5.013773	-0.529762	-0.980650
H	-5.289662	0.765565	-2.178250
N	-2.918640	1.115533	0.057788
C	-2.164108	0.507053	-0.845170
S	-3.000124	-0.066213	-2.242876
C	-2.104315	1.702257	1.115841
C	-0.653602	1.394326	0.658896
C	0.141195	2.624796	0.294010
N	-0.875037	0.522892	-0.536966
C	0.111188	-0.212847	-1.300423
O	-0.272771	-0.694036	-2.376314
C	1.367478	-0.261187	-0.687294
H	1.523222	0.105537	0.323521
N	2.442233	-0.921407	-1.229734
C	2.628254	-1.000690	-2.697331
C	3.748486	-0.511886	-0.667213
H	3.846242	0.577496	-0.804709
H	3.753649	-0.713130	0.412303
C	-0.292815	3.471504	-0.729431
C	0.426430	4.618219	-1.043551
C	1.584897	4.930416	-0.334817
C	2.020705	4.089652	0.683991
C	1.303301	2.938363	0.998200
H	-2.347672	1.235968	2.078404
H	-2.291767	2.781366	1.184907
H	-0.119124	0.818048	1.429263
C	4.098726	-1.393694	-2.889295
H	4.195092	-2.412370	-3.282121
C	4.764622	-1.265180	-1.504569

H	4.968175	-2.252958	-1.073906
H	5.718779	-0.730229	-1.546506
H	4.568420	-0.727578	-3.621218
H	2.416007	-0.008327	-3.120912
H	1.905841	-1.703354	-3.119304
H	-1.198097	3.235071	-1.292390
H	0.082411	5.270598	-1.843998
H	2.147993	5.828857	-0.581065
H	2.928227	4.326900	1.236463
H	1.652226	2.257942	1.779018
C	2.301631	-3.012526	-0.671112
H	2.917039	-2.811983	0.206169
H	2.807638	-3.501201	-1.502353
C	0.923634	-3.136803	-0.504363
Br	2.033874	-0.215465	2.926238
C	0.329109	-2.482036	0.549121
H	0.993400	-2.060668	1.310100
H	0.326335	-3.567108	-1.310446
C	-1.085046	-2.359538	0.844874
C	-1.461135	-1.723638	2.043166
C	-2.097442	-2.826552	-0.009137
C	-2.800064	-1.563273	2.374190
H	-0.671300	-1.345755	2.698277
C	-3.435614	-2.664685	0.325760
H	-1.835385	-3.315886	-0.946417
C	-3.794237	-2.028770	1.514194
H	-3.072238	-1.073405	3.308295
H	-4.207352	-3.035938	-0.347961
H	-4.845008	-1.902428	1.770764

Statistical Thermodynamic Analysis

Temperature= 323.150 Kelvin Pressure= 1.00000 Atm

==

SCF Energy= -4217.09250325 Predicted Change= -1.548897D-09

Zero-point correction (ZPE)= -4216.5780 0.51443

Internal Energy (U)= -4216.5436 0.54884

Enthalpy (H)= -4216.5426 0.54986

Gibbs Free Energy (G)= -4216.6487 0.44376

Entropy (S)= 0.00032834

Frequencies -- -235.0562 11.7443 34.8068

Supporting Information: **Desmethyl C-N Homolysis TS (*si*)**

Using Gaussian 16: ES64L-G16RevA.03 25-Dec-2016

==

#um06/6-31G(d) scf=(maxcycle=300,direct,tight,xqc) guess=(mix,always)

density=current opt=(maxcycle=250,modredundant) iop(1/8=18)

Temperature=323.15 SCRF=(PCM,SOLVENT=acetonitrile)

Modredundant Input: B 18 44 F

Modredundant Input:

#um06/6-31G(d) scf=(direct,tight,maxcycle=300,xqc)

opt=(nofreeze,maxcycle=250,ts,calcfc,noeigentest) iop(1/8=18) freq=noraman

SCRF=(PCM,SOLVENT=acetonitrile) Temperature=323.15 geom=check

guess=(mix,always)

#N Geom=AllCheck Guess=TCheck SCRF=Check Test GenChk UM06/6-31G(d) Freq

----

Pointgroup= C1 Stoichiometry= C26H30BrN3OS C1[X(C26H30BrN3OS)] #Atoms= 62

Charge = 0 Multiplicity = 1

SCF Energy= -4217.08602664 Predicted Change= -5.654207D-10

==

Optimization completed. {Found 3 times}

Item	Max Val.		Criteria	Pass?	RMS Val.		Criteria	Pass?
Force	0.00000	||	0.00045	[YES]	0.00000	||	0.00030	[YES]
Displ	0.00032	||	0.00180	[YES]	0.00032	||	0.00180	[YES]

Atomic Type	Coordinates (Angstroms) X	Y	Z
C	-4.439253	1.099912	-0.186299
H	-4.599585	2.171842	-0.376851
H	-5.075492	0.791466	0.650809
C	-4.699916	0.245887	-1.423102
H	-5.032241	-0.761621	-1.153555
H	-5.431627	0.697767	-2.096100
N	-3.043264	0.865939	0.137603
C	-2.265968	0.486530	-0.865183
S	-3.086798	0.080244	-2.328674
C	-2.252536	1.333143	1.270287
C	-0.792061	1.201313	0.760828
C	-0.076360	2.522163	0.613222
N	-0.975983	0.549764	-0.575259
C	0.048581	0.035772	-1.470765
O	-0.307498	-0.206274	-2.634272
C	1.286456	-0.091580	-0.836458
H	1.376380	0.049967	0.237560
N	2.424179	-0.591613	-1.411694
C	2.649872	-0.495475	-2.866825
C	3.686745	-0.154390	-0.768675
H	3.660511	0.945815	-0.707446
H	3.713046	-0.540832	0.258211
C	-0.609886	3.515554	-0.212182
C	0.032114	4.740415	-0.342990
C	1.214388	4.983975	0.353255
C	1.749546	3.997414	1.174864
C	1.108846	2.767591	1.305567
H	-2.447302	0.705667	2.148398
H	-2.510702	2.372686	1.510156
H	-0.218782	0.526908	1.414838
C	4.094979	-0.944019	-3.046628

H	4.141425	-2.017536	-3.266186
C	4.793404	-0.631998	-1.705620
H	5.300289	-1.520153	-1.310939
H	5.557693	0.144698	-1.814578
H	4.560136	-0.425853	-3.891527
H	2.512479	0.556262	-3.162796
H	1.905567	-1.091211	-3.402420
H	-1.534253	3.332427	-0.763762
H	-0.390268	5.507248	-0.989635
H	1.718242	5.943531	0.251650
H	2.675359	4.182376	1.716922
H	1.536969	1.980084	1.931755
C	2.442592	-2.754882	-1.032731
H	3.488847	-2.902761	-0.771554
H	2.178525	-2.964155	-2.069457
C	1.450158	-2.816909	-0.056041
Br	2.265664	-0.531187	2.727033
C	0.142057	-2.615931	-0.424420
H	1.737781	-2.776511	0.996747
C	-1.028852	-2.505193	0.426749
C	-0.957509	-2.168458	1.790362
C	-2.294858	-2.714939	-0.145404
C	-2.118075	-2.051290	2.544167
H	0.010455	-1.934950	2.240248
C	-3.452044	-2.611598	0.617049
H	-2.360384	-2.975859	-1.202585
C	-3.368912	-2.273228	1.965543
H	-2.048462	-1.777876	3.596274
H	-4.422028	-2.802627	0.159553
H	-4.273264	-2.183603	2.564819
H	-0.074391	-2.642657	-1.497737

Statistical Thermodynamic Analysis

Temperature= 323.150 Kelvin Pressure= 1.00000 Atm

==

SCF Energy= -4217.08602664 Predicted Change= -5.654207D-10

Zero-point correction (ZPE)= -4216.5714 0.51456

Internal Energy (U)= -4216.5372 0.54879

Enthalpy (H)= -4216.5362 0.54982

Gibbs Free Energy (G)= -4216.6403 0.44570

Entropy (S)= 0.00032219

Frequencies -- -229.3412 29.6199 38.8157

Supporting Information: **Desmethyl Concerted [2,3] TS (*re*)**

Using Gaussian 16: ES64L-G16RevA.03 25-Dec-2016

==

#m06/6-31G(d) scf=(maxcycle=300,direct,tight,xqc) density=current

opt=(maxcycle=250,modredundant) iop(1/8=18) Temperature=323.15

SCRF=(PCM,SOLVENT=acetonitrile)

Modredundant Input: B 4 44 F

Modredundant Input: B 3 49 F

Modredundant Input:

#m06/6-31G(d) scf=(direct,tight,maxcycle=300,xqc)

opt=(nofreeze,maxcycle=250,ts,calcfc,noeigentest) iop(1/8=18) freq=noraman

SCRF=(PCM,SOLVENT=acetonitrile) Temperature=323.15 geom=check

#N Geom=AllCheck Guess=TCheck SCRF=Check Test GenChk RM06/6-31G(d) Freq

----

Pointgroup= C1 Stoichiometry= C26H30BrN3OS C1[X(C26H30BrN3OS)] #Atoms= 62

Charge = 0 Multiplicity = 1

SCF Energy= -4217.09255822 Predicted Change= -4.391511D-08

==

Optimization completed. {Found 2 times}

Item	Max Val.	Criteria	Pass?	RMS Val.	Criteria	Pass?
Force	0.00001 ||	0.00045	[YES]	0.00000 ||	0.00030	[YES]

Displ 0.00296 || 0.00180 [NO] 0.00296 || 0.00180 [YES]

Atomic Type	Coordinates (Angstroms) X	Y	Z
C	-0.089656	-0.229207	1.291816
N	0.900081	0.498806	0.523767
C	-1.360413	-0.229719	0.706151
N	-2.447693	-0.843605	1.273945
C	-3.749430	-0.388078	0.739548
H	-3.807437	0.704135	0.878182
H	-3.784711	-0.589437	-0.339482
C	-2.602684	-0.918909	2.744507
O	0.304467	-0.742975	2.348349
C	0.692583	1.350730	-0.690779
C	-0.044275	2.625035	-0.349961
C	2.148077	1.566703	-1.161895
C	2.171332	0.565574	0.889978
C	4.348418	1.069972	0.173122
H	4.895313	1.975638	-0.107088
H	4.649097	0.249941	-0.497322
C	4.562817	0.716710	1.639324
H	5.404002	0.034881	1.783525
H	4.710908	1.608976	2.255134
S	3.013062	-0.124450	2.224628
C	0.438203	3.495751	0.631306
C	-0.237183	4.676888	0.914655
C	-1.399990	5.001088	0.219024
C	-1.883899	4.137523	-0.757845
C	-1.210372	2.952470	-1.042161
H	-1.527142	0.157445	-0.295229
H	-2.348040	0.065114	3.164953
H	-1.895524	-1.646428	3.149907
C	-4.081246	-1.261435	2.968482

H	-4.511419	-0.581482	3.711872
H	-4.203882	-2.277245	3.361438
C	-4.772618	-1.106673	1.598843
H	-5.019441	-2.086018	1.171489
H	-5.706443	-0.538997	1.662657
H	0.123408	0.778495	-1.439002
H	2.324195	2.588341	-1.512885
H	2.435093	0.852544	-1.949325
H	1.348925	3.254499	1.180736
H	0.146204	5.347521	1.681507
H	-1.928186	5.926359	0.442251
H	-2.794843	4.383169	-1.300980
H	-1.597442	2.258302	-1.792361
N	2.913932	1.286804	0.050374
C	-2.395643	-2.958306	0.711556
H	-2.908025	-3.427727	1.549821
H	-3.015128	-2.725303	-0.154571
C	-1.025553	-3.119715	0.530701
H	-0.432269	-3.573484	1.326698
C	-0.422087	-2.478237	-0.527292
C	0.992273	-2.414340	-0.839977
C	1.376130	-1.850460	-2.070793
C	2.715404	-1.775935	-2.431706
C	3.700940	-2.252046	-1.567259
C	3.334710	-2.797190	-0.337046
C	1.996617	-2.878096	0.025197
H	0.593949	-1.462223	-2.728653
H	2.993922	-1.347327	-3.393651
H	4.750802	-2.199209	-1.851378
H	4.100854	-3.158693	0.347372
H	1.726956	-3.297837	0.993423
H	-1.080011	-2.040683	-1.284465
Br	-2.087640	-0.196940	-2.896485

Statistical Thermodynamic Analysis

Temperature= 323.150 Kelvin   Pressure= 1.00000 Atm

==

SCF Energy=   -4217.09255822   Predicted Change= -4.391511D-08

Zero-point correction (ZPE)=   -4216.5778   0.51475

Internal Energy (U)=   -4216.5435   0.54903

Enthalpy (H)=   -4216.5425   0.55005

Gibbs Free Energy (G)=   -4216.6479   0.44461

Entropy (S)=   0.00032629

Frequencies --   -223.5264   20.6618   34.0381

Supporting Information: **Desmethyl Concerted [2,3] TS (*si*)**

Using Gaussian 16: ES64L-G16RevA.03 25-Dec-2016

==

#m06/6-31G(d) scf=(maxcycle=300,direct,tight,xqc) density=current

opt=(maxcycle=250,modredundant) iop(1/8=18) Temperature=323.15

SCRF=(PCM,SOLVENT=acetonitrile)

Modredundant Input: B   4   44 F

Modredundant Input: B   3   49 F

Modredundant Input:

#m06/6-31G(d) scf=(direct,tight,maxcycle=300,xqc)

opt=(nofreeze,maxcycle=250,ts,calcfc,noeigentest) iop(1/8=18) freq=noraman

SCRF=(PCM,SOLVENT=acetonitrile) Temperature=323.15 geom=check

#N Geom=AllCheck Guess=TCheck SCRF=Check Test GenChk RM06/6-31G(d) Freq

----

Pointgroup= C1   Stoichiometry= C26H30BrN3OS   C1[X(C26H30BrN3OS)] #Atoms= 62

Charge = 0   Multiplicity = 1

SCF Energy= -4217.08692700 Predicted Change= -1.943891D-11

==

Optimization completed.   {Found   3   times}

Item	Max Val.	Criteria	Pass?	RMS Val.	Criteria	Pass?
Force	0.00000 ||	0.00045	[YES]	0.00000 ||	0.00030	[YES]
Displ	0.00006 ||	0.00180	[YES]	0.00006 ||	0.00180	[YES]

Atomic Type	Coordinates (Angstroms) X	Y	Z
C	-0.042886	0.079645	1.484607
N	1.005949	0.511285	0.574598
C	-1.292087	0.014028	0.862271
N	-2.452074	-0.393339	1.464572
C	-3.691168	0.087519	0.807986
H	-3.601092	1.180028	0.693116
H	-3.748488	-0.346078	-0.198521
C	-2.661468	-0.223669	2.915424
O	0.305192	-0.163401	2.650216
C	0.860987	1.081238	-0.805677
C	0.264681	2.468950	-0.757478
C	2.319666	1.025835	-1.307990
C	2.283891	0.527266	0.917795
C	4.483904	0.614057	0.102691
H	5.119712	1.371402	-0.366354
H	4.674478	-0.357864	-0.379298
C	4.698907	0.559786	1.610176
H	5.459395	-0.170373	1.895912
H	4.967415	1.538952	2.018012
S	3.080334	0.057647	2.372263
C	0.893130	3.490570	-0.039147
C	0.346654	4.767683	-0.009398
C	-0.833429	5.038255	-0.698717
C	-1.462027	4.025603	-1.415225
C	-0.917533	2.744065	-1.445322
H	-1.386429	0.138807	-0.213104
H	-2.461165	0.829822	3.166447

H	-1.950126	-0.839087	3.472936
C	-4.128753	-0.579213	3.119995
H	-4.556689	-0.002569	3.946454
H	-4.235659	-1.638995	3.381432
C	-4.817443	-0.279277	1.771357
H	-5.382916	-1.148970	1.417204
H	-5.529938	0.548357	1.852516
H	0.224189	0.416671	-1.408227
H	2.593294	1.910549	-1.891365
H	2.512854	0.119708	-1.903429
H	1.818947	3.292252	0.501989
H	0.844173	5.555283	0.553533
H	-1.261488	6.038833	-0.674729
H	-2.385845	4.230448	-1.953563
H	-1.418001	1.942890	-1.995877
N	3.079781	0.960812	-0.061078
C	-2.601445	-2.576497	1.180117
H	-2.321116	-2.742361	2.220231
H	-3.660740	-2.679494	0.952052
C	-1.640457	-2.736236	0.185257
C	-0.314880	-2.576431	0.512971
H	-1.952746	-2.742888	-0.860788
C	0.840494	-2.590461	-0.364494
C	2.111559	-2.739768	0.214652
C	0.754199	-2.450882	-1.761603
C	3.258411	-2.780085	-0.568996
H	2.188855	-2.839390	1.298239
C	1.903089	-2.487595	-2.540921
H	-0.212896	-2.245827	-2.224882
C	3.157993	-2.660942	-1.953414
H	4.231942	-2.910278	-0.096927
H	1.822186	-2.373853	-3.620869
H	4.052695	-2.693849	-2.572651
H	-0.076013	-2.546917	1.580820

Br -2.290720 -0.544638 -2.677040

Statistical Thermodynamic Analysis

Temperature= 323.150 Kelvin Pressure= 1.00000 Atm

===

SCF Energy= -4217.08692700 Predicted Change= -1.943891D-11

Zero-point correction (ZPE)= -4216.5727 0.51417

Internal Energy (U)= -4216.5384 0.54848

Enthalpy (H)= -4216.5374 0.54950

Gibbs Free Energy (G)= -4216.6420 0.44490

Entropy (S)= 0.00032371

Frequencies -- -223.5906 29.2518 37.6358

Supporting Information: **Desmethyl [1,2] Radical Recombination TS (*re*)**

Using Gaussian 16: ES64L-G16RevA.03 25-Dec-2016

===

#um06/6-31G(d) scf=(maxcycle=300,direct,tight,xqc) guess=(mix,always)

density=current opt=(maxcycle=250,modredundant) iop(1/8=18)

Temperature=323.15 SCRF=(PCM,SOLVENT=acetonitrile)

Modredundant Input: B 3 44 F

Modredundant Input:

#um06/6-31G(d) scf=(direct,tight,maxcycle=300,xqc) guess=(mix,always)

opt=(nofreeze,maxcycle=250,ts,calcfc,noeigentest) iop(1/8=18) freq=noraman

SCRF=(PCM,SOLVENT=acetonitrile) Temperature=323.15 geom=check

#N Geom=AllCheck Guess=TCheck SCRF=Check Test GenChk UM06/6-31G(d) Freq

----

Pointgroup= C1 Stoichiometry= C26H30BrN3OS C1[X(C26H30BrN3OS)] #Atoms= 62

Charge = 0 Multiplicity = 1

SCF Energy= -4217.09311629 Predicted Change= -1.562429D-09

===

Optimization completed. {Found 3 times}

Item	Max Val.	Criteria	Pass?	RMS Val.	Criteria	Pass?
Force	0.00000 ||	0.00045	[YES]	0.00000 ||	0.00030	[YES]
Displ	0.00036 ||	0.00180	[YES]	0.00036 ||	0.00180	[YES]

Atomic Coordinates (Angstroms)

Type	X	Y	Z
C	0.853589	-0.087413	-1.455604
N	-0.118591	0.624655	-0.680858
C	1.936139	-0.599774	-0.682047
N	3.057466	-1.116142	-1.196192
C	3.312578	-1.455667	-2.600525
H	2.415551	-1.886331	-3.058142
H	3.562015	-0.542554	-3.161475
C	4.099627	-1.641136	-0.308937
O	0.650170	-0.207015	-2.667708
C	0.043479	1.213537	0.688889
C	1.085869	2.303638	0.695813
C	-1.394225	1.692791	0.994666
C	-1.301485	0.979041	-1.174458
C	-3.434750	1.825984	-0.641839
H	-3.801814	2.814102	-0.345977
H	-4.000647	1.057827	-0.091849
C	-3.527725	1.628750	-2.153319
H	-4.433114	1.087884	-2.440696
H	-3.486227	2.577726	-2.695449
S	-2.053988	0.627397	-2.681565
C	1.015925	3.373596	-0.200195
C	1.969706	4.383107	-0.160776
C	3.000381	4.333381	0.775810
C	3.073753	3.270065	1.669488
C	2.120862	2.255463	1.630161
H	1.939429	-0.485641	0.402744

H	3.732041	-2.547876	0.200493
H	4.344985	-0.900376	0.463259
C	5.235905	-1.960521	-1.265196
H	5.918822	-2.716407	-0.864809
H	5.816983	-1.052083	-1.475921
C	4.492600	-2.416804	-2.517935
H	5.108085	-2.397866	-3.423106
H	4.125398	-3.443723	-2.379849
H	0.329604	0.417697	1.393047
H	-1.413717	2.689901	1.446842
H	-1.934029	0.989478	1.647492
H	0.213005	3.421729	-0.936962
H	1.909353	5.212099	-0.863443
H	3.747563	5.124378	0.805200
H	3.880035	3.224960	2.399429
H	2.177224	1.405416	2.314559
N	-2.019159	1.704192	-0.324157
C	0.433846	-2.601557	-0.343063
H	0.932633	-2.600781	0.627801
H	0.965473	-3.033452	-1.190003
C	-0.932946	-2.356190	-0.416107
H	-1.416215	-2.467356	-1.391156
C	-1.680808	-1.919777	0.663472
C	-3.085720	-1.593899	0.662566
C	-3.910775	-1.707592	-0.476672
C	-5.249984	-1.348881	-0.425965
C	-5.811797	-0.864142	0.756659
C	-5.015937	-0.754657	1.896709
C	-3.677281	-1.116833	1.850428
H	-3.497563	-2.085601	-1.410959
H	-5.865205	-1.447116	-1.319305
H	-6.862017	-0.581401	0.789791
H	-5.443346	-0.385627	2.827443
H	-3.059358	-1.035050	2.746176

H -1.146357 -1.798053 1.613628

Br 1.558882 -1.271261 2.921549

Statistical Thermodynamic Analysis

Temperature= 323.150 Kelvin Pressure= 1.00000 Atm

===

SCF Energy= -4217.09311629 Predicted Change= -1.562429D-09

Zero-point correction (ZPE)= -4216.5814 0.51165

Internal Energy (U)= -4216.5461 0.54695

Enthalpy (H)= -4216.5451 0.54797

Gibbs Free Energy (G)= -4216.6542 0.43891

Entropy (S)= 0.00033748

Frequencies -- -184.7341 18.9811 29.6223

Supporting Information: **Desmethyl [1,2] Radical Recombination TS (*si*)**

Using Gaussian 16: ES64L-G16RevA.03 25-Dec-2016

==

#um06/6-31G(d) scf=(direct,tight,maxcycle=300,xqc) guess=(mix,always)

opt=(nofreeze,maxcycle=250,ts,calcfc,noeigentest) iop(1/8=18) freq=noraman

SCRF=(PCM,SOLVENT=acetonitrile) Temperature=323.15

#N Geom=AllCheck Guess=TCheck SCRF=Check Test GenChk UM06/6-31G(d) Freq

----

Pointgroup= C1 Stoichiometry= C26H30BrN3OS C1[X(C26H30BrN3OS)] #Atoms= 62

Charge = 0 Multiplicity = 1

SCF Energy= -4217.09200130 Predicted Change= 9.814210D-09

===

Optimization completed. {Found 1 times}

Item Max Val. Criteria Pass? RMS Val. Criteria Pass?

Force 0.00001 || 0.00045 [YES] 0.00000 || 0.00030 [YES]

Displ 0.00250 || 0.00180 [NO] 0.00250 || 0.00180 [YES]

Atomic Type	Coordinates (Angstroms) X	Y	Z
C	-1.007379	-1.294059	-0.916239
N	0.206460	-1.134924	-0.168001
C	-1.936463	-0.234084	-0.707812
N	-3.200711	-0.229791	-1.142581
C	-3.750869	-1.047118	-2.230014
H	-2.964554	-1.286188	-2.953454
H	-4.135716	-1.996887	-1.829071
C	-4.070171	0.908457	-0.818908
O	-1.105971	-2.277722	-1.657698
C	0.396136	-0.410532	1.132933
C	-0.522953	-0.967661	2.191761
C	1.905116	-0.642846	1.424026
C	1.330477	-1.761355	-0.497865
C	3.510112	-2.328485	0.180601
H	3.452919	-3.142324	0.918839
H	4.420711	-1.746673	0.362992
C	3.456508	-2.845093	-1.252937
H	3.961544	-2.168767	-1.950287
H	3.881330	-3.846040	-1.352718
S	1.662625	-2.919959	-1.732694
C	-0.512261	-2.334052	2.486010
C	-1.339332	-2.844215	3.478384
C	-2.183409	-1.991504	4.187565
C	-2.199772	-0.631999	3.895246
C	-1.374367	-0.118477	2.897769
H	-1.679010	0.596229	-0.046775
H	-3.601219	1.841411	-1.174504
H	-4.181487	0.998757	0.269652
C	-5.359986	0.586055	-1.555924

H	-5.935489	1.484642	-1.799695
H	-5.994004	-0.065888	-0.939122
C	-4.866173	-0.171219	-2.785473
H	-5.647537	-0.754346	-3.283714
H	-4.449718	0.535553	-3.518056
H	0.191385	0.660712	0.982758
H	2.060663	-1.178767	2.369813
H	2.475613	0.293983	1.444530
H	0.143869	-3.009038	1.933131
H	-1.326470	-3.909728	3.699613
H	-2.831119	-2.390945	4.965861
H	-2.862575	0.035771	4.442720
H	-1.399090	0.943467	2.641825
N	2.340932	-1.473145	0.305389
C	-0.592538	0.860980	-2.597515
H	-1.563270	1.336794	-2.731258
H	-0.392992	-0.022527	-3.207838
C	0.436995	1.516567	-1.935315
Br	-1.272463	3.021118	0.859912
C	1.737611	1.051703	-1.883574
C	2.787708	1.553275	-1.031483
C	4.085033	1.017903	-1.151989
C	2.564925	2.519449	-0.025648
C	5.114892	1.421972	-0.313956
H	4.275641	0.274369	-1.928086
C	3.600775	2.924864	0.803763
H	1.559595	2.912167	0.144187
C	4.879695	2.381675	0.669115
H	6.107070	0.988193	-0.428925
H	3.406325	3.665319	1.578305
H	5.683843	2.700356	1.329561
H	0.154458	2.389867	-1.342301
H	2.002636	0.206321	-2.528980

Statistical Thermodynamic Analysis

Temperature= 323.150 Kelvin Pressure= 1.00000 Atm

==

SCF Energy= -4217.09200130 Predicted Change= 9.814210D-09

Zero-point correction (ZPE)= -4216.5802 0.51176

Internal Energy (U)= -4216.5451 0.54690

Enthalpy (H)= -4216.5440 0.54792

Gibbs Free Energy (G)= -4216.6518 0.44015

Entropy (S)= 0.00033351

Frequencies -- -194.2627 27.9854 31.6249

Supporting Information: **Desmethyl [2,3] Radical Recombination TS (*re*)**

Using Gaussian 16: ES64L-G16RevA.03 25-Dec-2016

==

#um06/6-31G(d) scf=(maxcycle=300,direct,tight,xqc) guess=(mix,always)

density=current opt=(maxcycle=250,modredundant) iop(1/8=18)

Temperature=323.15 SCRF=(PCM,SOLVENT=acetonitrile)

Modredundant Input: B 3 49 F

Modredundant Input:

#um06/6-31G(d) scf=(direct,tight,maxcycle=300,xqc) guess=(mix,always)

opt=(nofreeze,maxcycle=250,ts,calcfc,noeigentest) iop(1/8=18) freq=noraman

SCRF=(PCM,SOLVENT=acetonitrile) Temperature=323.15 geom=check

#N Geom=AllCheck Guess=TCheck SCRF=Check Test GenChk UM06/6-31G(d) Freq

----

Pointgroup= C1 Stoichiometry= C26H30BrN3OS C1[X(C26H30BrN3OS)] #Atoms= 62

Charge = 0 Multiplicity = 1

SCF Energy= -4217.09636241 Predicted Change= -4.971253D-09

==

Optimization completed. {Found 3 times}

Item Max Val. Criteria Pass? RMS Val. Criteria Pass?

Force 0.00000 || 0.00045 [YES] 0.00000 || 0.00030 [YES]

Displ 0.00140 || 0.00180 [YES] 0.00140 || 0.00180 [YES]

Atomic Type	Coordinates (Angstroms) X	Y	Z
C	0.037546	-0.160890	-1.268019
N	-0.942247	0.471315	-0.436671
C	1.323468	-0.258754	-0.663381
N	2.441505	-0.540534	-1.340055
C	3.739995	-0.398154	-0.670726
H	3.916207	0.668084	-0.441819
H	3.729115	-0.937877	0.285378
C	2.589062	-0.560736	-2.806292
O	-0.318905	-0.542525	-2.385854
C	-0.716830	1.255268	0.824871
C	0.045099	2.528326	0.537931
C	-2.167468	1.473931	1.309144
C	-2.215468	0.592556	-0.803115
C	-4.383314	1.111263	-0.064665
H	-4.892455	2.013840	0.287080
H	-4.729020	0.251236	0.529362
C	-4.589622	0.898592	-1.559764
H	-5.448209	0.258228	-1.773563
H	-4.703290	1.846820	-2.093826
S	-3.058518	0.069908	-2.210130
C	-0.451581	3.470807	-0.367553
C	0.249574	4.644584	-0.614031
C	1.453710	4.889866	0.042694
C	1.952003	3.955500	0.944053
C	1.252400	2.777109	1.191848
H	1.470095	0.007626	0.382581
H	2.014197	0.259041	-3.253284
H	2.193170	-1.500236	-3.207631

C	4.095885	-0.408678	-3.016488
H	4.345290	0.651306	-3.161931
H	4.445556	-0.954670	-3.898848
C	4.718225	-0.905964	-1.712172
H	4.753817	-2.003769	-1.694762
H	5.733522	-0.529064	-1.551057
H	-0.159657	0.634527	1.542236
H	-2.327228	2.486420	1.693556
H	-2.462302	0.741647	2.073631
H	-1.394294	3.292883	-0.886467
H	-0.145605	5.371480	-1.321108
H	2.002462	5.809660	-0.151336
H	2.893892	4.140229	1.457612
H	1.648484	2.031154	1.885749
N	-2.942597	1.250485	0.092478
C	2.295046	-3.418177	-1.028991
H	2.688741	-3.950417	-1.892734
H	3.009850	-3.130345	-0.257564
C	0.969403	-3.189895	-0.865980
H	0.279441	-3.539119	-1.637850
C	0.456378	-2.454131	0.231908
C	-0.936782	-2.395108	0.615954
C	-1.266445	-1.976548	1.922937
C	-2.586389	-1.938037	2.350090
C	-3.620046	-2.298166	1.483542
C	-3.312560	-2.693783	0.182485
C	-1.992790	-2.743004	-0.248139
H	-0.453840	-1.678514	2.590521
H	-2.813585	-1.628188	3.369740
H	-4.655115	-2.273866	1.820674
H	-4.111677	-2.965520	-0.506545
H	-1.778080	-3.046134	-1.272305
H	1.166167	-2.192077	1.021762
Br	2.106979	-0.456236	2.874645

Statistical Thermodynamic Analysis

Temperature= 323.150 Kelvin Pressure= 1.00000 Atm

===

SCF Energy= -4217.09636241 Predicted Change= -4.971253D-09

Zero-point correction (ZPE)= -4216.5835 0.51284

Internal Energy (U)= -4216.5486 0.54768

Enthalpy (H)= -4216.5476 0.54871

Gibbs Free Energy (G)= -4216.6538 0.44249

Entropy (S)= 0.0003287

Frequencies -- -179.4398 23.1925 32.8775

Supporting Information: **Desmethyl Radical Recombination [2,3] TS (*si*)**

Using Gaussian 16: ES64L-G16RevA.03 25-Dec-2016

===

#um06/6-31G(d) scf=(maxcycle=300,direct,tight,xqc) guess=(mix,always)

density=current opt=(maxcycle=250,modredundant) iop(1/8=18)

Temperature=323.15 SCRF=(PCM,SOLVENT=acetonitrile)

Modredundant Input: B 3 45 F

Modredundant Input:

#um06/6-31G(d) scf=(direct,tight,maxcycle=300,xqc) guess=(mix,always)

opt=(nofreeze,maxcycle=250,ts,calcfc,noeigentest) iop(1/8=18) freq=noraman

SCRF=(PCM,SOLVENT=acetonitrile) Temperature=323.15 geom=check

#N Geom=AllCheck Guess=TCheck SCRF=Check Test GenChk UM06/6-31G(d) Freq

----

Pointgroup= C1 Stoichiometry= C26H30BrN3OS C1[X(C26H30BrN3OS)] #Atoms= 62

Charge = 0 Multiplicity = 1

SCF Energy= -4217.09332310 Predicted Change= -8.026705D-10

==

Optimization completed. {Found 3 times}

Item	Max Val.	Criteria	Pass?	RMS Val.	Criteria	Pass?
Force	0.00000 ||	0.00045	[YES]	0.00000 ||	0.00030	[YES]
Displ	0.00061 ||	0.00180	[YES]	0.00061 ||	0.00180	[YES]

Atomic Type	Coordinates (Angstroms) X	Y	Z
C	0.034621	0.074425	1.405156
N	1.044811	0.422280	0.450130
C	-1.260559	-0.071194	0.825751
N	-2.405504	-0.099782	1.511406
C	-3.683697	0.159026	0.832815
H	-3.746702	1.235119	0.590749
H	-3.725024	-0.399286	-0.110329
C	-2.555341	-0.101158	2.971286
O	0.381283	-0.076823	2.579838
C	0.853470	1.001343	-0.925425
C	0.264639	2.390929	-0.833867
C	2.294325	0.942515	-1.473486
C	2.336759	0.482799	0.765574
C	4.515040	0.628381	-0.100592
H	5.096938	1.398196	-0.616809
H	4.750502	-0.352278	-0.542041
C	4.760675	0.656586	1.404118
H	5.550645	-0.033468	1.708727
H	5.002768	1.662722	1.759027
S	3.176761	0.141264	2.228951
C	0.929016	3.405773	-0.137071
C	0.381300	4.680110	-0.059872
C	-0.836492	4.955790	-0.678107
C	-1.500773	3.951019	-1.372430
C	-0.954849	2.671937	-1.450968
H	-1.387594	-0.009085	-0.255438
H	-1.883909	0.633204	3.429677

H	-2.275882	-1.088432	3.367003
C	-4.034406	0.220190	3.181040
H	-4.161901	1.303647	3.311100
H	-4.440392	-0.270364	4.071740
C	-4.708503	-0.223829	1.882685
H	-4.860276	-1.311945	1.878706
H	-5.677518	0.258630	1.717723
H	0.194991	0.345286	-1.513725
H	2.549582	1.825065	-2.068410
H	2.469268	0.032627	-2.066843
H	1.884038	3.206129	0.350128
H	0.907417	5.461652	0.485090
H	-1.265355	5.954316	-0.615926
H	-2.453456	4.159508	-1.856242
H	-1.480749	1.878129	-1.987930
N	3.090816	0.888519	-0.250001
C	-1.812085	-2.824885	0.144372
C	-0.519035	-2.480733	0.623103
Br	-2.262622	-0.580453	-2.692318
C	-2.858985	-3.055876	0.970499
H	-2.735520	-3.070194	2.054307
H	-3.850779	-3.287633	0.586540
H	-1.980712	-2.823733	-0.934836
C	0.701962	-2.558772	-0.148939
C	1.926217	-2.715257	0.528347
C	0.734766	-2.483391	-1.557319
C	3.127890	-2.810091	-0.162302
H	1.920240	-2.774904	1.617722
C	1.937818	-2.583696	-2.242259
H	-0.186359	-2.282689	-2.107321
C	3.142080	-2.752836	-1.554541
H	4.058670	-2.934636	0.391467
H	1.939862	-2.522128	-3.329995
H	4.080915	-2.834025	-2.099936

H -0.386334 -2.520964 1.709143

Statistical Thermodynamic Analysis

Temperature= 323.150 Kelvin Pressure= 1.00000 Atm

==

SCF Energy= -4217.09332310 Predicted Change= -8.026705D-10

Zero-point correction (ZPE)= -4216.5808 0.51245

Internal Energy (U)= -4216.5460 0.54725

Enthalpy (H)= -4216.5450 0.54827

Gibbs Free Energy (G)= -4216.6505 0.44275

Entropy (S)= 0.00032654

Frequencies -- -213.9975 29.4325 36.0244

Supporting Information: **C-N Homolysis TS (*re*)**

Using Gaussian 16: ES64L-G16RevA.03 25-Dec-2016

==

#um06/6-31G(d) scf=(maxcycle=300,direct,tight,xqc) guess=(mix,always)

density=current opt=(maxcycle=250,modredundant) iop(1/8=18)

Temperature=323.15 SCRF=(PCM,SOLVENT=acetonitrile)

Modredundant Input: B 18 44 F

Modredundant Input:

#um06/6-31G(d) scf=(direct,tight,maxcycle=300,xqc)

opt=(nofreeze,maxcycle=250,ts,calcfc,noeigentest) iop(1/8=18) freq=noraman

SCRF=(PCM,SOLVENT=acetonitrile) Temperature=323.15 geom=check

guess=(mix,always)

#N Geom=AllCheck Guess=TCheck SCRF=Check Test GenChk UM06/6-31G(d) Freq

----

Pointgroup= C1 Stoichiometry= C27H32BrN3OS C1[X(C27H32BrN3OS)] #Atoms= 65

Charge = 0 Multiplicity = 1

SCF Energy= -4256.36632222 Predicted Change= -2.085583D-09

==

Optimization completed. {Found 3 times}

Item	Max Val.		Criteria	Pass?	RMS Val.		Criteria	Pass?
Force	0.00001	||	0.00045	[YES]	0.00000	||	0.00030	[YES]
Displ	0.00103	||	0.00180	[YES]	0.00103	||	0.00180	[YES]

Atomic Type	Coordinates (Angstroms) X	Y	Z
C	-3.513531	0.208101	-2.483178
H	-3.364639	0.834742	-3.375235
H	-4.450032	0.504338	-1.997968
C	-3.507443	-1.280232	-2.814880
H	-4.116861	-1.852976	-2.107691
H	-3.848406	-1.482567	-3.832453
N	-2.399162	0.392577	-1.569215
C	-1.420998	-0.496085	-1.660771
S	-1.758527	-1.875349	-2.642388
C	-1.945172	1.607528	-0.900990
C	-0.483159	1.269990	-0.514544
C	0.527081	2.227090	-1.093548
N	-0.313979	-0.115065	-1.035490
C	0.808729	-1.008304	-0.846109
O	0.745887	-2.096478	-1.438802
C	1.817237	-0.501934	-0.020662
H	1.697774	0.442241	0.499177
N	2.963581	-1.216738	0.277274
C	3.531751	-2.121910	-0.767741
C	4.088207	-0.390883	0.828045
H	3.852019	0.671294	0.702443
H	4.190067	-0.588903	1.901556
C	0.864456	2.182584	-2.447419
C	1.771382	3.095678	-2.972918
C	2.344367	4.062792	-2.149813

C	2.003696	4.114554	-0.801671
C	1.097096	3.199976	-0.272566
H	-2.540911	1.828726	-0.005344
H	-2.004853	2.457807	-1.594275
H	-0.412046	1.261340	0.581789
C	4.992224	-2.243777	-0.384832
H	5.123935	-2.950511	0.446743
C	5.328215	-0.824377	0.055877
H	6.235666	-0.755494	0.664433
H	5.464567	-0.184765	-0.826549
H	5.604597	-2.594693	-1.221909
H	3.425494	-1.628334	-1.744130
H	2.967296	-3.056161	-0.798388
H	0.424278	1.421534	-3.094003
H	2.034273	3.051000	-4.028279
H	3.056331	4.775981	-2.561352
H	2.447109	4.870252	-0.155530
H	0.802304	3.241002	0.778568
C	2.511923	-2.633506	1.825886
H	2.955687	-2.023320	2.608759
H	3.134460	-3.465103	1.499541
C	1.129601	-2.729107	1.674557
H	0.780246	-3.528951	1.023846
C	0.236427	-1.738268	2.037529
C	-1.180440	-1.805542	1.641312
C	-1.707446	-2.867274	0.882801
C	-3.042360	-2.888964	0.502124
C	-3.897199	-1.845518	0.853237
C	-3.395685	-0.784522	1.600734
C	-2.063919	-0.768546	1.995030
C	0.626338	-0.651313	2.997999
H	0.109068	-0.804599	3.957987
H	0.341462	0.357509	2.666000
H	1.700175	-0.636709	3.202706

H	-1.073389	-3.694934	0.574049
H	-3.416799	-3.728552	-0.082585
H	-4.942988	-1.863990	0.549027
H	-4.040036	0.048462	1.881651
H	-1.713180	0.097652	2.554136
Br	-1.289562	2.742837	2.595228

Statistical Thermodynamic Analysis

Temperature= 323.150 Kelvin   Pressure= 1.00000 Atm

==

SCF Energy=   -4256.36632222   Predicted Change= -2.085583D-09

Zero-point correction (ZPE)=   -4255.8241   0.54218

Internal Energy (U)=   -4255.7882   0.57811

Enthalpy (H)=   -4255.7871   0.57914

Gibbs Free Energy (G)=   -4255.8963   0.46995

Entropy (S)=   0.00033789

Frequencies --   -248.3992   17.7488   27.3042

Supporting Information: **[1,2] Radical Recombination TS (*re*)**

Using Gaussian 16: ES64L-G16RevA.03 25-Dec-2016

==

#um06/6-31G(d) scf=(maxcycle=300,direct,tight,xqc) guess=(mix,always)

density=current opt=(maxcycle=250,modredundant) iop(1/8=18)

Temperature=323.15 SCRF=(PCM,SOLVENT=acetonitrile)

Modredundant Input: B   3   44 F

Modredundant Input:

#um06/6-31G(d) scf=(direct,tight,maxcycle=300,xqc)

opt=(nofreeze,maxcycle=250,ts,calcfc,noeigentest) iop(1/8=18) freq=noraman

SCRF=(PCM,SOLVENT=acetonitrile) Temperature=323.15 geom=check

guess=(mix,always)

#N Geom=AllCheck Guess=TCheck SCRF=Check Test GenChk UM06/6-31G(d) Freq

----

Pointgroup= C1 Stoichiometry= C27H32BrN3OS C1[X(C27H32BrN3OS)] #Atoms= 65

Charge = 0 Multiplicity = 1

SCF Energy= -4256.37469887 Predicted Change= -9.531822D-10

==

Optimization completed. {Found 3 times}

Item	Max Val.	Criteria	Pass?	RMS Val.	Criteria	Pass?
Force	0.00000 ||	0.00045	[YES]	0.00000 ||	0.00030	[YES]
Displ	0.00038 ||	0.00180	[YES]	0.00038 ||	0.00180	[YES]

Atomic Type	Coordinates (Angstroms) X	Y	Z
C	0.684675	0.170955	1.384752
N	-0.149647	-0.604904	0.521435
C	1.833936	0.709788	0.735210
N	2.855748	1.291007	1.372350
C	2.928631	1.638173	2.796320
H	1.990166	2.095084	3.129822
H	3.076269	0.723651	3.389149
C	3.971422	1.866251	0.616063
O	0.320224	0.321641	2.555479
C	0.234977	-1.299327	-0.749642
C	1.263619	-2.370077	-0.479192
C	-1.129402	-1.846707	-1.241157
C	-1.388638	-0.955492	0.858332
C	-3.205943	-2.350613	0.307995
H	-2.943695	-3.394816	0.538153
H	-3.943699	-2.339565	-0.502484
C	-3.721504	-1.597835	1.531149
H	-4.427731	-0.807083	1.253845
H	-4.187900	-2.263157	2.261028
S	-2.256532	-0.788842	2.337011

C	1.014692	-3.372236	0.463181
C	1.951200	-4.373681	0.685375
C	3.144726	-4.383689	-0.034204
C	3.397369	-3.387128	-0.970679
C	2.461502	-2.380091	-1.193294
H	1.992302	0.550396	-0.332598
H	3.645126	2.799962	0.125599
H	4.287236	1.174177	-0.174285
C	5.002255	2.141849	1.695367
H	5.732849	2.899720	1.394960
H	5.546214	1.218886	1.939819
C	4.130896	2.574607	2.872185
H	4.640808	2.515489	3.839200
H	3.802987	3.613605	2.727993
H	0.639216	-0.564825	-1.462415
H	-1.078135	-2.914259	-1.487736
H	-1.510218	-1.302322	-2.114978
H	0.084746	-3.374543	1.035454
H	1.750158	-5.148993	1.422156
H	3.878323	-5.168691	0.140011
H	4.331028	-3.388130	-1.530295
H	2.659107	-1.579024	-1.909628
N	-2.008646	-1.632018	-0.096505
C	0.286652	2.574979	0.103641
H	0.823780	2.583612	-0.846781
H	0.784459	3.041073	0.952889
C	-1.076349	2.297766	0.177667
H	-1.518982	2.329395	1.176236
C	-1.878805	1.885100	-0.878267
C	-3.276841	1.514916	-0.658840
C	-4.009307	1.996511	0.445666
C	-5.330867	1.629463	0.654229
C	-5.972519	0.759507	-0.227858
C	-5.269283	0.269175	-1.325482

C	-3.948137	0.643265	-1.539107
C	-1.306161	1.853693	-2.265949
H	-0.863604	2.830555	-2.510279
H	-2.060822	1.644258	-3.030063
H	-0.477955	1.137029	-2.387605
H	-3.545673	2.702978	1.131922
H	-5.870359	2.035122	1.508567
H	-7.010245	0.475362	-0.064983
H	-5.752489	-0.411912	-2.024526
H	-3.423390	0.233973	-2.401565
Br	2.367254	1.013372	-2.805266

Statistical Thermodynamic Analysis

Temperature= 323.150 Kelvin Pressure= 1.00000 Atm

==

SCF Energy= -4256.37469887 Predicted Change= -9.531822D-10

Zero-point correction (ZPE)= -4255.8347 0.53991

Internal Energy (U)= -4255.7979 0.57670

Enthalpy (H)= -4255.7969 0.57773

Gibbs Free Energy (G)= -4255.9080 0.46666

Entropy (S)= 0.0003437

Frequencies -- -209.2990 23.3611 29.2384

Supporting Information: **[2,3] Radical Recombination TS (*si*)**

Using Gaussian 16: ES64L-G16RevA.03 25-Dec-2016

==

#um06/6-31G(d) scf=(maxcycle=300,direct,tight,xqc) guess=(mix,always)

density=current opt=(maxcycle=250,modredundant) iop(1/8=18)

Temperature=323.15 SCRF=(PCM,SOLVENT=acetonitrile)

Modredundant Input: B 3 45 F

Modredundant Input:

#um06/6-31G(d) scf=(direct,tight,maxcycle=300,xqc) guess=(mix,always)

opt=(nofreeze,maxcycle=250,ts,calcfc,noeigentest) iop(1/8=18) freq=noraman

SCRF=(PCM,SOLVENT=acetonitrile) Temperature=323.15 geom=check

#N Geom=AllCheck Guess=TCheck SCRF=Check Test GenChk UM06/6-31G(d) Freq

----

Pointgroup= C1 Stoichiometry= C27H32BrN3OS C1[X(C27H32BrN3OS)] #Atoms= 65

Charge = 0 Multiplicity = 1

SCF Energy= -4256.37225393 Predicted Change= -8.361857D-10

==

Optimization completed. {Found 3 times}

Item	Max Val.		Criteria	Pass?	RMS Val.		Criteria	Pass?
Force	0.00000	||	0.00045	[YES]	0.00000	||	0.00030	[YES]
Displ	0.00091	||	0.00180	[YES]	0.00091	||	0.00180	[YES]

Atomic Type	Coordinates (Angstroms) X	Y	Z
C	-0.036257	-0.011656	-1.358395
N	-1.059987	0.417615	-0.447056
C	1.230868	-0.231086	-0.733197
N	2.398288	-0.282319	-1.391305
C	3.659521	0.037192	-0.692935
H	3.531764	0.946023	-0.088386
H	3.921429	-0.774562	-0.004643
C	2.591095	-0.360893	-2.840620
O	-0.348179	-0.181056	-2.538222
C	-0.873386	1.223187	0.809968
C	-0.284335	2.576287	0.484457
C	-2.316583	1.265508	1.355220
C	-2.350955	0.427172	-0.773202
C	-4.532870	0.702043	0.054260
H	-5.117458	1.547623	0.429578
H	-4.767412	-0.188246	0.656922

C	-4.777370	0.470554	-1.433150
H	-5.557084	-0.272131	-1.615276
H	-5.032388	1.398034	-1.953988
S	-3.185358	-0.151371	-2.163994
C	-0.922262	3.439835	-0.412276
C	-0.380234	4.687846	-0.691146
C	0.805520	5.087398	-0.077758
C	1.444406	4.232082	0.812784
C	0.903749	2.979540	1.094172
H	1.338774	-0.056344	0.339073
H	2.329351	0.600601	-3.313612
H	1.948203	-1.130023	-3.277663
C	4.079544	-0.641641	-2.957742
H	4.478954	-0.383928	-3.944402
H	4.269011	-1.709250	-2.775777
C	4.665075	0.201441	-1.827993
H	5.674563	-0.104858	-1.534512
H	4.712654	1.254090	-2.139755
H	-0.217067	0.680745	1.505749
H	-2.572917	2.243719	1.774371
H	-2.495175	0.483362	2.106673
H	-1.852167	3.143741	-0.899018
H	-0.885318	5.351943	-1.389982
H	1.230021	6.065313	-0.297615
H	2.373067	4.537008	1.292121
H	1.410478	2.296231	1.780434
N	-3.109665	0.986429	0.160774
C	1.738205	-2.773255	0.237567
C	0.440795	-2.523703	-0.349413
C	2.803695	-3.304528	-0.392351
H	2.774802	-3.675952	-1.415129
H	3.753108	-3.414757	0.128799
H	1.888548	-2.435467	1.265439
C	0.214387	-3.015928	-1.746329

H	1.128647	-2.955147	-2.344673
H	-0.108030	-4.069460	-1.743805
H	-0.550638	-2.437377	-2.278968
C	-0.735727	-2.434682	0.527817
C	-2.020988	-2.729880	0.033607
C	-0.645694	-2.067135	1.887532
C	-3.148823	-2.672310	0.846065
H	-2.149952	-3.028580	-1.004360
C	-1.772890	-2.010479	2.694970
H	0.311116	-1.765605	2.314760
C	-3.036080	-2.316779	2.186149
H	-4.123228	-2.916166	0.422459
H	-1.662869	-1.718627	3.739059
H	-3.916626	-2.275144	2.825069
Br	2.275146	-0.018151	2.791014

Statistical Thermodynamic Analysis

Temperature= 323.150 Kelvin   Pressure= 1.00000 Atm

==

SCF Energy=   -4256.37225393   Predicted Change= -8.361857D-10

Zero-point correction (ZPE)=   -4255.8316   0.54059

Internal Energy (U)=   -4255.7949   0.57732

Enthalpy (H)=   -4255.7939   0.57834

Gibbs Free Energy (G)=   -4255.9053   0.46690

Entropy (S)=   0.00034486

Frequencies --   -351.3330   17.6590   19.5831

Supporting Information: **Dimethyl Amine C-N Homolysis TS**

Using Gaussian 16: ES64L-G16RevA.03 25-Dec-2016

==

#um06/6-31G(d) scf=(maxcycle=300,direct,tight,xqc) density=current

opt=(maxcycle=250,modredundant) iop(1/8=18) guess=(mix,always)

Temperature=323.15 SCRF=(PCM,SOLVENT=acetonitrile)

Modredundant Input: B 18 32 F

Modredundant Input:

#um06/6-31G(d) scf=(direct,tight,maxcycle=300,xqc)

opt=(nofreeze,maxcycle=250,ts,calcfc,noeigentest) iop(1/8=18) freq=noraman

SCRF=(PCM,SOLVENT=acetonitrile) Temperature=323.15 geom=check

guess=(mix,always)

#N Geom=AllCheck Guess=TCheck SCRF=Check Test GenChk UM06/6-31G(d) Freq

----

Pointgroup= C1 Stoichiometry= C25H30BrN3OS C1[X(C25H30BrN3OS)] #Atoms= 61

Charge = 0 Multiplicity = 1

SCF Energy= -4179.00527606 Predicted Change= -7.562478D-10

===

Optimization completed. {Found 3 times}

Item	Max Val.		Criteria	Pass?	RMS Val.		Criteria	Pass?
Force	0.00000	||	0.00045	[YES]	0.00000	||	0.00030	[YES]
Displ	0.00068	||	0.00180	[YES]	0.00068	||	0.00180	[YES]

Atomic Type	Coordinates (Angstroms) X	Y	Z
C	3.585254	2.294938	-0.624866
H	3.472599	3.367657	-0.404379
H	4.077648	2.183724	-1.597488
C	4.346567	1.556528	0.469572
H	4.853425	0.669674	0.076091
H	5.073528	2.193383	0.978027
N	2.278864	1.661072	-0.652662
C	1.879996	1.104527	0.483368
S	3.091774	0.982099	1.710344
C	1.147347	1.907304	-1.538883
C	-0.062332	1.360891	-0.733974

C	-1.076004	2.416426	-0.364296
N	0.592845	0.803091	0.492362
C	-0.025766	0.070676	1.593782
O	0.631569	0.032681	2.650843
C	-1.262829	-0.459747	1.236957
H	-1.594898	-0.388967	0.204294
N	-2.060236	-1.286321	1.995853
C	-0.685574	3.535285	0.376529
C	-1.610590	4.518812	0.703587
C	-2.935611	4.393072	0.290662
C	-3.328769	3.279908	-0.444929
C	-2.403773	2.291406	-0.771273
H	1.289859	1.378515	-2.488444
H	1.053224	2.982417	-1.741688
H	-0.551586	0.544805	-1.287006
H	0.350162	3.641108	0.706107
H	-1.297612	5.386186	1.281823
H	-3.660655	5.163918	0.545742
H	-4.363953	3.175931	-0.765545
H	-2.708877	1.404020	-1.332038
C	-1.544192	-3.389162	1.460638
H	-2.557488	-3.707337	1.223497
H	-1.213851	-3.617950	2.471290
C	-0.654381	-3.135976	0.422976
Br	-2.710993	-1.194088	-2.140881
C	0.661640	-2.737469	0.595544
C	1.346942	-2.888238	1.913228
H	0.715644	-3.372165	2.662646
H	2.256960	-3.495132	1.819856
H	1.641428	-1.904869	2.315646
H	-1.106134	-3.033721	-0.565168
C	1.470116	-2.229138	-0.526092
C	0.889851	-1.860200	-1.754920
C	2.860256	-2.055412	-0.393725

C	1.660721	-1.352618	-2.790847
H	-0.190010	-1.912832	-1.897752
C	3.631980	-1.554330	-1.436786
H	3.359777	-2.317759	0.536034
C	3.038364	-1.195573	-2.642559
H	1.173318	-1.068068	-3.723323
H	4.708590	-1.452247	-1.305126
H	3.640565	-0.797636	-3.457494
C	-1.903702	-1.274058	3.451149
H	-2.268832	-0.319954	3.859498
H	-2.494553	-2.093370	3.873828
H	-0.850388	-1.388039	3.712277
C	-3.470015	-1.251840	1.585138
H	-3.917421	-0.286730	1.867033
H	-3.527832	-1.376206	0.495860
H	-4.012091	-2.061278	2.085721

Statistical Thermodynamic Analysis

Temperature= 323.150 Kelvin Pressure= 1.00000 Atm

==

SCF Energy= -4179.00527606 Predicted Change= -7.562478D-10

Zero-point correction (ZPE)= -4178.4987 0.50657

Internal Energy (U)= -4178.4643 0.54096

Enthalpy (H)= -4178.4632 0.54198

Gibbs Free Energy (G)= -4178.5664 0.43886

Entropy (S)= 0.00031913

Frequencies -- -182.8371 31.9186 38.1536

Supporting Information: **Dimethyl Amine Concerted [2,3] TS**

Using Gaussian 16: ES64L-G16RevA.03 25-Dec-2016

==

#um06/6-31G(d) scf=(maxcycle=300,direct,tight,xqc) guess=(mix,always)

density=current opt=(maxcycle=250,modredundant) iop(1/8=18)

Temperature=323.15 SCRF=(PCM,SOLVENT=acetonitrile)

Modredundant Input: B 4 32 F

Modredundant Input: B 3 37 F

Modredundant Input:

#um06/6-31G(d) scf=(direct,tight,maxcycle=300,xqc)

opt=(nofreeze,maxcycle=250,ts,calcfc,noeigentest) iop(1/8=18) freq=noraman

SCRF=(PCM,SOLVENT=acetonitrile) Temperature=323.15 geom=check

guess=(mix,always)

#N Geom=AllCheck Guess=TCheck SCRF=Check Test GenChk UM06/6-31G(d) Freq

----

Pointgroup= C1 Stoichiometry= C25H30BrN3OS C1[X(C25H30BrN3OS)] #Atoms= 61

Charge = 0 Multiplicity = 1

SCF Energy= -4179.00238120 Predicted Change= -1.266568D-08

==

Optimization completed. {Found 2 times}

Item	Max Val.		Criteria	Pass?	RMS Val.		Criteria	Pass?
Force	0.00001	||	0.00045	[YES]	0.00000	||	0.00030	[YES]
Displ	0.00516	||	0.00180	[NO]	0.00516	||	0.00180	[YES]

Atomic Type	Coordinates (Angstroms) X	Y	Z
C	0.262771	1.365737	-1.120086
N	0.087690	-0.064880	-1.009800
C	-0.660310	2.113899	-0.371476
N	-0.702978	3.487649	-0.336108
O	1.189703	1.738598	-1.854649

C	-0.923251	-0.824793	-0.228876
C	-2.305644	-0.736653	-0.829260
C	-0.330608	-2.248843	-0.246326
C	0.846097	-0.923420	-1.683199
C	1.575269	-3.147082	-1.807461
H	1.123250	-4.114772	-2.045946
H	2.284810	-3.279019	-0.974706
C	2.234775	-2.519405	-3.027960
H	3.286485	-2.798724	-3.123553
H	1.706521	-2.774988	-3.951642
S	2.132287	-0.678510	-2.801436
C	-2.518862	-0.508090	-2.188113
C	-3.813741	-0.483356	-2.697628
C	-4.901859	-0.687804	-1.854312
C	-4.691341	-0.917063	-0.497021
C	-3.399045	-0.940215	0.015750
H	-1.394508	1.608643	0.248225
H	-0.940642	-0.475977	0.811147
H	-1.100767	-3.018136	-0.361859
H	0.246876	-2.448800	0.669907
H	-1.674387	-0.334257	-2.855604
H	-3.971752	-0.299743	-3.758846
H	-5.913881	-0.665085	-2.254509
H	-5.538975	-1.073193	0.168068
H	-3.214296	-1.125803	1.076718
N	0.548668	-2.192622	-1.412292
C	0.824645	4.187900	1.222240
H	1.130914	5.120706	0.752672
H	0.027369	4.279550	1.955447
C	1.642931	3.079012	1.146646
H	2.497089	3.164020	0.476472
C	1.330731	1.822569	1.661019
C	2.180461	0.658465	1.378533
C	1.945531	-0.568916	2.028664

C	2.734300	-1.684579	1.775501
C	3.789827	-1.613069	0.870571
C	4.032210	-0.411673	0.207810
C	3.239981	0.701200	0.450225
C	0.276492	1.669392	2.718663
H	0.758333	1.530701	3.699722
H	-0.371760	0.792387	2.581596
H	-0.367638	2.548624	2.797925
H	1.113840	-0.681415	2.722856
H	2.514438	-2.617273	2.295038
H	4.414984	-2.483899	0.679031
H	4.843371	-0.341570	-0.515167
H	3.446048	1.608714	-0.110926
Br	-1.372494	-1.817151	3.101798
C	-1.945808	4.036596	0.207297
H	-2.227912	3.494293	1.115469
H	-1.795821	5.092562	0.455145
H	-2.759124	3.952063	-0.528382
C	-0.236911	4.242928	-1.503931
H	0.779530	3.942746	-1.758309
H	-0.896011	4.048214	-2.363548
H	-0.268810	5.309663	-1.261421

Statistical Thermodynamic Analysis

Temperature= 323.150 Kelvin   Pressure= 1.00000 Atm

==

SCF Energy= -4179.00238120 Predicted Change= -1.266568D-08

Zero-point correction (ZPE)= -4178.4965 0.50584

Internal Energy (U)= -4178.4618 0.54050

Enthalpy (H)= -4178.4608 0.54153

Gibbs Free Energy (G)= -4178.5656 0.43673

Entropy (S)= 0.0003243

Frequencies -- -128.9837 18.3888 33.3843

Supporting Information: **Dimethyl Amine [1,2] Radical Recombination TS**

Using Gaussian 16: ES64L-G16RevA.03 25-Dec-2016

==

#um06/6-31G(d) scf=(maxcycle=300,direct,tight,xqc) density=current

opt=(maxcycle=250,modredundant) iop(1/8=18) guess=(mix,always)

Temperature=323.15 SCRF=(PCM,SOLVENT=acetonitrile)

Modredundant Input: B 3 32 F

Modredundant Input:

#um06/6-31G(d) scf=(direct,tight,maxcycle=300,xqc)

opt=(nofreeze,maxcycle=250,ts,calcfc,noeigentest) iop(1/8=18) freq=noraman

SCRF=(PCM,SOLVENT=acetonitrile) Temperature=323.15 geom=check

guess=(mix,always)

#N Geom=AllCheck Guess=TCheck SCRF=Check Test GenChk UM06/6-31G(d) Freq

----

Pointgroup= C1 Stoichiometry= C25H30BrN3OS C1[X(C25H30BrN3OS)] #Atoms= 61

Charge = 0 Multiplicity = 1

SCF Energy= -4179.00786728 Predicted Change= -2.707136D-09

==

Optimization completed. {Found 3 times}

Item	Max Val.		Criteria	Pass?	RMS Val.		Criteria	Pass?
Force	0.00000	||	0.00045	[YES]	0.00000	||	0.00030	[YES]
Displ	0.00123	||	0.00180	[YES]	0.00123	||	0.00180	[YES]

Atomic Type	Coordinates (Angstroms) X	Y	Z
C	-0.763392	-0.128227	1.614454
N	-0.167745	0.640105	0.563306
C	-1.874524	-0.910347	1.184879
N	-2.744756	-1.555620	1.981508
O	-0.238548	-0.058615	2.732386

C	-0.767818	1.015931	-0.759406
C	-2.015092	1.844002	-0.573036
C	0.390179	1.793046	-1.440452
C	1.025710	1.216192	0.692897
C	2.539136	2.742979	-0.273412
H	2.150713	3.764453	-0.142341
H	3.168024	2.712789	-1.170771
C	3.300702	2.247751	0.949677
H	4.057722	1.502822	0.678703
H	3.769865	3.060984	1.507580
S	2.064653	1.413692	2.056392
C	-1.987292	2.991384	0.224653
C	-3.125038	3.775983	0.363291
C	-4.299688	3.422677	-0.298400
C	-4.331702	2.280639	-1.091390
C	-3.193840	1.489324	-1.228138
H	-2.133096	-0.922979	0.127376
H	-1.000000	0.097918	-1.321228
H	0.095595	2.818261	-1.701702
H	0.760845	1.296953	-2.345689
H	-1.071499	3.275256	0.747146
H	-3.096157	4.666089	0.988895
H	-5.190887	4.038260	-0.190693
H	-5.249451	1.997770	-1.603892
H	-3.215039	0.573396	-1.823180
N	1.425919	1.820009	-0.416535
C	-0.167368	-2.716276	0.579918
H	-1.029515	-3.021617	-0.010855
H	-0.142030	-3.014831	1.627748
C	0.933264	-2.206473	-0.089335
Br	-2.310431	-2.008354	-2.264307
C	2.185770	-1.891260	0.436916
C	2.536148	-2.248638	1.849733
H	1.667519	-2.598233	2.414199

H	3.295453	-3.044706	1.886207
H	2.951584	-1.387290	2.392521
C	3.211128	-1.243596	-0.376170
C	4.529395	-1.100597	0.104381
C	2.935229	-0.686762	-1.643607
C	5.504668	-0.437381	-0.631049
H	4.799823	-1.517921	1.072047
C	3.906742	-0.020950	-2.374632
H	1.934418	-0.765145	-2.065426
C	5.201294	0.115331	-1.872903
H	6.511951	-0.350736	-0.226664
H	3.648457	0.402495	-3.344583
H	5.962878	0.641343	-2.445059
H	0.750355	-2.011127	-1.149459
C	-2.569869	-1.744614	3.408678
H	-3.556328	-1.744437	3.887486
H	-2.089651	-2.715644	3.611525
H	-1.951378	-0.950535	3.825751
C	-3.725387	-2.432353	1.365150
H	-3.483884	-3.482612	1.591452
H	-4.725684	-2.216016	1.761510
H	-3.715647	-2.300768	0.276670

Statistical Thermodynamic Analysis

Temperature= 323.150 Kelvin Pressure= 1.00000 Atm

==

SCF Energy= -4179.00786728 Predicted Change= -2.707136D-09

Zero-point correction (ZPE)= -4178.5040 0.50382

Internal Energy (U)= -4178.4683 0.53955

Enthalpy (H)= -4178.4672 0.54057

Gibbs Free Energy (G)= -4178.5757 0.43214

Entropy (S)= 0.00033555

Frequencies -- -139.4644 19.1444 33.2143

Supporting Information: **Dimethyl Amine [2,3] Radical Recombination TS**

Using Gaussian 16: ES64L-G16RevA.03 25-Dec-2016

==

#um06/6-31G(d) scf=(maxcycle=300,direct,tight,xqc) density=current

opt=(maxcycle=250,modredundant) iop(1/8=18) guess=(mix,always)

Temperature=323.15 SCRF=(PCM,SOLVENT=acetonitrile)

Modredundant Input: B 3 33 F

Modredundant Input:

#um06/6-31G(d) scf=(direct,tight,maxcycle=300,xqc)

opt=(nofreeze,maxcycle=250,ts,calcfc,noeigentest) iop(1/8=18) freq=noraman

SCRF=(PCM,SOLVENT=acetonitrile) Temperature=323.15 geom=check

guess=(mix,always)

#N Geom=AllCheck Guess=TCheck SCRF=Check Test GenChk UM06/6-31G(d) Freq

----

Pointgroup= C1 Stoichiometry= C25H30BrN3OS C1[X(C25H30BrN3OS)] #Atoms= 61

Charge = 0 Multiplicity = 1

SCF Energy= -4179.00789892 Predicted Change= -2.253484D-09

==

Optimization completed. {Found 3 times}

Item	Max Val.	Criteria	Pass?	RMS Val.	Criteria	Pass?
Force	0.00000 ||	0.00045	[YES]	0.00000 ||	0.00030	[YES]
Displ	0.00078 ||	0.00180	[YES]	0.00078 ||	0.00180	[YES]

Atomic Type	Coordinates (Angstroms) X	Y	Z
C	0.124760	0.039726	1.494018
N	0.859756	0.511720	0.352130
C	-1.227658	-0.305741	1.189232
N	-2.235087	-0.451683	2.073317
O	0.737103	-0.055112	2.561263

C	0.309537	1.230800	-0.850176
C	-0.337804	2.529691	-0.428422
C	1.578941	1.386950	-1.714084
C	2.182984	0.660853	0.370630
C	4.078213	1.088645	-0.949551
H	4.475201	1.959595	-1.479892
H	4.253641	0.188988	-1.559212
C	4.678832	0.961849	0.446402
H	5.549757	0.303031	0.464683
H	4.953201	1.935407	0.863063
S	3.366993	0.239447	1.547232
C	0.388585	3.499636	0.270289
C	-0.216657	4.689900	0.652744
C	-1.554648	4.924489	0.341962
C	-2.281007	3.963613	-0.352381
C	-1.677292	2.768805	-0.737024
H	-1.564808	-0.200734	0.158370
H	-0.423281	0.592554	-1.364945
H	1.630035	2.364546	-2.204079
H	1.662006	0.591138	-2.469013
H	1.436617	3.330999	0.520728
H	0.357893	5.437598	1.196244
H	-2.028596	5.856716	0.643927
H	-3.327288	4.140232	-0.595717
H	-2.246135	2.004887	-1.273151
N	2.647422	1.241253	-0.729284
C	-1.727179	-2.904327	0.338915
C	-0.378288	-2.574749	0.723245
Br	-2.910274	-0.428593	-2.158482
C	-2.703838	-3.337892	1.161050
H	-2.551462	-3.562372	2.215321
H	-3.708397	-3.506021	0.776737
H	-2.012438	-2.702112	-0.694566
C	0.066215	-2.955523	2.100538

H	-0.752177	-2.872652	2.822855
H	0.419081	-3.998714	2.123412
H	0.881739	-2.320554	2.468016
C	0.653771	-2.452365	-0.315649
C	2.012190	-2.654120	-0.006940
C	0.343858	-2.127549	-1.653807
C	3.005372	-2.544333	-0.975054
H	2.307282	-2.913857	1.007442
C	1.336951	-2.021374	-2.616786
H	-0.682576	-1.896245	-1.941682
C	2.677736	-2.230403	-2.289677
H	4.045366	-2.707940	-0.692816
H	1.059749	-1.765813	-3.639334
H	3.452825	-2.148370	-3.050061
C	-2.075924	-0.419598	3.515245
H	-2.385121	0.560375	3.912223
H	-2.719390	-1.187498	3.965081
H	-1.036100	-0.601575	3.788443
C	-3.594871	-0.208662	1.613196
H	-4.282786	-0.903075	2.110265
H	-3.897933	0.822654	1.860901
H	-3.656336	-0.349644	0.527187

Statistical Thermodynamic Analysis

Temperature= 323.150 Kelvin   Pressure= 1.00000 Atm

==

SCF Energy=   -4179.00789892   Predicted Change= -2.253484D-09

Zero-point correction (ZPE)=   -4178.5033   0.50450

Internal Energy (U)=   -4178.4679   0.53992

Enthalpy (H)=   -4178.4669   0.54094

Gibbs Free Energy (G)=   -4178.5732   0.43463

Entropy (S)=   0.00032899

Frequencies --   -316.9581   27.3475   33.6446

Supporting Information: **[1,2] Radical Recombination Allyl TS Distortion**

Using Gaussian 16: ES64L-G16RevA.03 25-Dec-2016

==

#m06/6-31g(d) scf=(maxcycle=300,direct,vshift=200,tight,yqc)

density=current scrf=(pcm,solvent=ch3cn) freq=noraman temperature=323.15

Pointgroup= C1 Stoichiometry= C10H11(2) C1[X(C10H11)] #Atoms= 21

Charge = 0 Multiplicity = 2

SCF Energy= -387.325491972 Predicted Change= -2.178589D-03

==

Optimization incomplete.

Item	Max Val.		Criteria	Pass?	RMS Val.		Criteria	Pass?
Force	0.02637	||	0.00045	[NO]	0.00478	||	0.00030	[NO]
Displ	0.12269	||	0.00180	[NO]	0.12269	||	0.00180	[NO]

Atomic Type	Coordinates (Angstroms) X	Y	Z
C	3.531683	-0.809974	0.019235
H	4.073817	-1.722531	-0.222357
H	4.107052	0.112241	0.088644
C	2.147816	-0.823480	-0.095533
C	1.270215	0.252352	0.003583
C	1.785989	1.656064	0.108920
H	2.872172	1.690650	0.229707
H	1.537179	2.244409	-0.787275
H	1.346636	2.183493	0.968236
C	-0.178208	0.061696	-0.029053
C	-1.048981	1.169414	-0.087872
C	-0.780415	-1.212801	0.044392
C	-2.430008	1.013255	-0.064788

H	-0.640264	2.175426	-0.153110
C	-2.157590	-1.368309	0.071945
H	-0.159222	-2.105920	0.087932
C	-2.997362	-0.255260	0.023484
H	-3.068061	1.894259	-0.114308
H	-2.580449	-2.370131	0.137099
H	-4.078337	-0.377418	0.048861
H	1.730644	-1.822217	-0.249310

Statistical Thermodynamic Analysis

Temperature= 323.150 Kelvin Pressure= 1.00000 Atm

===

SCF Energy= -387.325491972 Predicted Change= -2.178589D-03

Zero-point correction (ZPE)= -387.1494 0.17608

Internal Energy (U)= -387.1385 0.18695

Enthalpy (H)= -387.1375 0.18797

Gibbs Free Energy (G)= -387.1895 0.13590

Entropy (S)= 0.00016115

Frequencies -- 12.1428 131.5511 167.4029

Supporting Information: **[1,2] Radical Recombination Allyl Distortion Interaction Ground State**

Using Gaussian 16: ES64L-G16RevA.03 25-Dec-2016

===

#m06/6-31g(d) scf=(maxcycle=300,direct,vshift=200,tight,yqc)

density=current scrf=(pcm,solvent=ch3cn) opt=(gdiis,maxcycle=250)

freq=noraman temperature=323.15

#N Geom=AllCheck Guess=TCheck SCRF=Check GenChk UM06/6-31G(d) Freq

----

Pointgroup= C1 Stoichiometry= C10H11(2) C1[X(C10H11)] #Atoms= 21

Charge = 0 Multiplicity = 2

SCF Energy= -387.327669482 Predicted Change= -3.032836D-09

==

Optimization completed. {Found 2 times}

Item	Max Val.	Criteria	Pass?	RMS Val.	Criteria	Pass?
Force	0.00000 ||	0.00045	[YES]	0.00000 ||	0.00030	[YES]
Displ	0.00087 ||	0.00180	[YES]	0.00087 ||	0.00180	[YES]

Atomic Type	Coordinates (Angstroms) X	Y	Z
C	3.513520	-0.805061	-0.120530
H	4.084561	-1.716999	-0.277811
H	4.090656	0.105152	0.030708
C	2.148737	-0.836441	-0.120791
C	1.262320	0.254633	0.044274
C	1.794410	1.646117	0.196359
H	2.861550	1.656341	0.435248
H	1.664490	2.235421	-0.724723
H	1.274660	2.191681	0.995111
C	-0.177189	0.063524	0.020811
C	-1.044797	1.170062	-0.112032
C	-0.781688	-1.208823	0.137051
C	-2.422991	1.013659	-0.143357
H	-0.629772	2.171014	-0.210387
C	-2.158606	-1.360789	0.109959
H	-0.165665	-2.094306	0.279537
C	-2.993162	-0.252380	-0.033770
H	-3.058647	1.890545	-0.256229
H	-2.588039	-2.356402	0.210382
H	-4.074323	-0.375211	-0.054492
H	1.697204	-1.814249	-0.295187

Statistical Thermodynamic Analysis

Temperature= 323.150 Kelvin Pressure= 1.00000 Atm

==

SCF Energy= -387.327669482 Predicted Change= -3.032836D-09

Zero-point correction (ZPE)= -387.1517 0.17597

Internal Energy (U)= -387.1406 0.18702

Enthalpy (H)= -387.1396 0.18804

Gibbs Free Energy (G)= -387.1915 0.13609

Entropy (S)= 0.00016076

Frequencies -- 29.8962 102.1861 123.9076

Supporting Information: **[1,2] Radical Recombination Amide TS Distortion**

Using Gaussian 16: ES64L-G16RevA.03 25-Dec-2016

==
===

#m06/6-31g(d) scf=(maxcycle=300,direct,vshift=200,tight,yqc)

density=current scrf=(pcm,solvent=ch3cn) freq=noraman temperature=323.15

Pointgroup= C1 Stoichiometry= C17H21BrN3OS(2) C1[X(C17H21BrN3OS)] #Atoms= 44

Charge = 0 Multiplicity = 2

SCF Energy= -3869.01917290 Predicted Change= -1.942189D-03

==

Optimization incomplete.

Item	Max Val.	Criteria	Pass?	RMS Val.	Criteria	Pass?
Force	0.01333 ||	0.00045	[NO]	0.00224 ||	0.00030	[NO]
Displ	0.12353 ||	0.00180	[NO]	0.12353 ||	0.00180	[NO]

Atomic Type	Coordinates (Angstroms) X	Y	Z
C	-0.132684	1.398732	0.238749
N	-1.131514	0.485700	-0.222632
C	1.198876	0.906776	0.115279

N	2.277294	1.512072	0.631008
C	2.355328	2.918429	1.043695
H	1.939219	3.554475	0.246906
H	1.758464	3.096851	1.944945
C	3.613894	0.936036	0.431272
O	-0.508459	2.500338	0.651796
C	-0.995659	-0.990959	-0.448511
C	-0.662168	-1.708243	0.836675
C	-2.388547	-1.367392	-1.020116
C	-2.387929	0.861258	-0.453672
C	-4.565383	0.096611	-0.933566
H	-4.985898	-0.382812	-0.036538
H	-5.026022	-0.346822	-1.824038
C	-4.738784	1.610831	-0.921128
H	-4.785600	2.019541	-1.936920
H	-5.619688	1.926055	-0.357808
S	-3.232121	2.324191	-0.101733
C	-1.467525	-1.544869	1.967416
C	-1.181208	-2.236698	3.137438
C	-0.089009	-3.101198	3.186357
C	0.716140	-3.264179	2.064394
C	0.433873	-2.568685	0.891431
H	1.379093	-0.084451	-0.299053
H	3.595800	0.243018	-0.421623
H	3.906519	0.366156	1.327718
C	4.489740	2.165017	0.260536
H	4.407205	2.540751	-0.770192
H	5.545819	1.966065	0.470222
C	3.850734	3.150461	1.236032
H	4.143165	2.899575	2.265184
H	4.130638	4.192942	1.053216
H	-0.207995	-1.168098	-1.197244
H	-2.889818	-2.126599	-0.405167
H	-2.340591	-1.733720	-2.052645

H	-2.325164	-0.869669	1.938781
H	-1.811899	-2.101983	4.014109
H	0.134179	-3.644192	4.102943
H	1.573980	-3.933251	2.100829
H	1.076329	-2.668502	0.013342
N	-3.127710	-0.112998	-0.962003
Br	2.221693	-1.648573	-2.276626

Statistical Thermodynamic Analysis

Temperature= 323.150 Kelvin   Pressure= 1.00000 Atm

==

SCF Energy=   -3869.01917290   Predicted Change= -1.942189D-03

Zero-point correction (ZPE)=   -3868.6580   0.36111

Internal Energy (U)=   -3868.6333   0.38580

Enthalpy (H)=   -3868.6323   0.38682

Gibbs Free Energy (G)=   -3868.7189   0.30021

Entropy (S)=   0.00026802

Frequencies --   -10.6603   20.2761   36.1552

Supporting Information: **[1,2] Radical Recombination Amide Distortion Interaction Ground State**

Using Gaussian 16: ES64L-G16RevA.03 25-Dec-2016

==

#m06/6-31g(d) scf=(maxcycle=300,direct,vshift=200,tight,yqc)

density=current scrf=(pcm,solvent=ch3cn) opt=(gdiis,maxcycle=250)

freq=noraman temperature=323.15

#N Geom=AllCheck Guess=TCheck SCRF=Check GenChk UM06/6-31G(d) Freq

----

Pointgroup= C1   Stoichiometry= C17H21BrN3OS(2)   C1[X(C17H21BrN3OS)] #Atoms= 44

Charge = 0   Multiplicity = 2

SCF Energy= -3869.02142902 Predicted Change= -6.109863D-08

==

Optimization completed. {Found 1 times}

Item	Max Val.		Criteria	Pass?	RMS Val.		Criteria	Pass?
Force	0.00001	||	0.00045	[YES]	0.00000	||	0.00030	[YES]
Displ	0.00342	||	0.00180	[NO]	0.00342	||	0.00180	[YES]

Atomic Coordinates (Angstroms)

Type	X	Y	Z
C	-0.116250	1.384417	0.184084
N	-1.125150	0.474773	-0.294880
C	1.196501	0.873873	0.105399
N	2.288295	1.546054	0.486288
C	2.349323	2.910762	1.020890
H	2.008861	3.615020	0.248248
H	1.677748	3.017224	1.879296
C	3.625896	0.962650	0.330457
O	-0.495019	2.500437	0.570251
C	-1.009503	-1.008714	-0.482580
C	-0.706513	-1.694325	0.826901
C	-2.401079	-1.374463	-1.065170
C	-2.381765	0.851362	-0.493440
C	-4.577220	0.109622	-0.880162
H	-4.950587	-0.309898	0.067171
H	-5.098323	-0.375319	-1.711411
C	-4.722501	1.624346	-0.935927
H	-4.772729	1.989418	-1.966148
H	-5.592376	1.982185	-0.381308
S	-3.193596	2.335700	-0.156535
C	-1.532568	-1.489784	1.935828
C	-1.277486	-2.149429	3.131282
C	-0.195581	-3.022715	3.227460
C	0.629980	-3.227025	2.127123
C	0.378841	-2.563903	0.928409

H	1.393395	-0.093297	-0.358001
H	3.658085	0.360384	-0.587646
H	3.830087	0.293618	1.181995
C	4.534320	2.179321	0.359581
H	4.566745	2.648662	-0.633345
H	5.558910	1.929443	0.652693
C	3.825357	3.092964	1.356917
H	4.021949	2.751307	2.382478
H	4.135819	4.140329	1.287034
H	-0.214169	-1.213101	-1.215435
H	-2.899764	-2.150584	-0.470888
H	-2.346548	-1.709916	-2.106242
H	-2.382817	-0.807361	1.871039
H	-1.924191	-1.982660	3.990587
H	0.003879	-3.540460	4.163905
H	1.479945	-3.903037	2.201047
H	1.038784	-2.694962	0.067399
N	-3.143318	-0.120154	-0.977746
Br	2.285934	-1.696576	-2.205082

Statistical Thermodynamic Analysis

Temperature= 323.150 Kelvin Pressure= 1.00000 Atm

==

SCF Energy= -3869.02142902 Predicted Change= -6.109863D-08

Zero-point correction (ZPE)= -3868.6599 0.36151

Internal Energy (U)= -3868.6344 0.38701

Enthalpy (H)= -3868.6333 0.38803

Gibbs Free Energy (G)= -3868.7221 0.29924

Entropy (S)= 0.00027477

Frequencies -- 21.4999 29.8563 40.4810

Using Gaussian 16: ES64L-G16RevA.03 25-Dec-2016

==

#m06/6-31g(d) scf=(maxcycle=300,direct,vshift=200,tight,yqc)

density=current scrf=(pcm,solvent=ch3cn) freq=noraman temperature=323.15

Pointgroup= C1   Stoichiometry= C10H11(2)   C1[X(C10H11)] #Atoms= 21

Charge = 0   Multiplicity = 2

SCF Energy= -387.320099663 Predicted Change= -5.172368D-03

==

Optimization incomplete.

Item	Max Val.	Criteria	Pass?	RMS Val.	Criteria	Pass?
Force	0.02981 ||	0.00045	[NO]	0.00597 ||	0.00030	[NO]
Displ	0.21903 ||	0.00180	[NO]	0.21903 ||	0.00180	[NO]

Atomic Type	Coordinates (Angstroms) X	Y	Z
C	-2.149012	-0.873963	0.014020
C	-1.272785	0.225030	-0.324448
C	-3.449216	-0.775352	0.351734
H	-3.968382	0.173159	0.476868
H	-4.036120	-1.670494	0.550256
H	-1.737282	-1.881270	-0.080982
C	-1.817943	1.611203	-0.162398
H	-2.888037	1.648842	-0.388361
H	-1.687290	1.965829	0.872520
H	-1.329640	2.336940	-0.824639
C	0.179549	0.054835	-0.171430
C	1.012478	1.170377	0.040882
C	0.807135	-1.208534	-0.216652

C	2.387083	1.036466	0.207969
H	0.582942	2.168283	0.094886
C	2.178657	-1.338352	-0.049850
H	0.227172	-2.104344	-0.440463
C	2.983890	-0.219428	0.169187
H	2.992209	1.927306	0.377102
H	2.626553	-2.330692	-0.095191
H	4.058851	-0.327258	0.303922

Statistical Thermodynamic Analysis

Temperature= 323.150 Kelvin Pressure= 1.00000 Atm

==
==

SCF Energy= -387.320099663 Predicted Change= -5.172368D-03

Zero-point correction (ZPE)= -387.1439 0.17612

Internal Energy (U)= -387.1330 0.18701

Enthalpy (H)= -387.1320 0.18803

Gibbs Free Energy (G)= -387.1833 0.13675

Entropy (S)= 0.00015872

Frequencies -- 45.3649 75.2838 170.2753

Supporting Information: **[2,3] Radical Recombination Allyl Distortion Interaction Ground State**

Using Gaussian 16: ES64L-G16RevA.03 25-Dec-2016

==

#m06/6-31g(d) scf=(maxcycle=300,direct,vshift=200,tight,yqc)

density=current scrf=(pcm,solvent=ch3cn) opt=(gdiis,maxcycle=250)

freq=noraman temperature=323.15

#N Geom=AllCheck Guess=TCheck SCRF=Check GenChk UM06/6-31G(d) Freq

----

Pointgroup= C1 Stoichiometry= C10H11(2) C1[X(C10H11)] #Atoms= 21

Charge = 0 Multiplicity = 2

SCF Energy= -387.327669483 Predicted Change= -1.941320D-10

==

Optimization completed. {Found 2 times}

Item	Max Val.		Criteria	Pass?	RMS Val.		Criteria	Pass?
Force	0.00000	||	0.00045	[YES]	0.00000	||	0.00030	[YES]
Displ	0.00108	||	0.00180	[YES]	0.00108	||	0.00180	[YES]

Atomic Type	Coordinates (Angstroms) X	Y	Z
C	-2.148732	-0.836381	0.120977
C	-1.262316	0.254670	-0.044235
C	-3.513524	-0.805115	0.120457
H	-4.090737	0.104975	-0.031215
H	-4.084482	-1.717071	0.277932
H	-1.697180	-1.814114	0.295768
C	-1.794391	1.646144	-0.196454
H	-2.861590	1.656371	-0.435062
H	-1.664217	2.235621	0.724481
H	-1.274828	2.191552	-0.995436
C	0.177182	0.063533	-0.020764
C	1.044822	1.170057	0.112121
C	0.781658	-1.208806	-0.137108
C	2.423003	1.013632	0.143407
H	0.629802	2.171002	0.210558
C	2.158582	-1.360796	-0.110056
H	0.165626	-2.094270	-0.279680
C	2.993155	-0.252419	0.033735
H	3.058681	1.890495	0.256331
H	2.587977	-2.356416	-0.210562
H	4.074315	-0.375259	0.054405

Statistical Thermodynamic Analysis

Temperature= 323.150 Kelvin Pressure= 1.00000 Atm

==

SCF Energy= -387.327669483 Predicted Change= -1.941320D-10

Zero-point correction (ZPE)= -387.1516 0.17597

Internal Energy (U)= -387.1406 0.18702

Enthalpy (H)= -387.1396 0.18804

Gibbs Free Energy (G)= -387.1915 0.13610

Entropy (S)= 0.00016073

Frequencies -- 30.1282 102.2213 124.0112

Supporting Information: **[2,3] Radical Recombination Amide TS Distortion**

Using Gaussian 16: ES64L-G16RevA.03 25-Dec-2016

==

#m06/6-31g(d) scf=(maxcycle=300,direct,vshift=200,tight,yqc)

density=current scrf=(pcm,solvent=ch3cn) freq=noraman temperature=323.15

Pointgroup= C1 Stoichiometry= C17H21BrN3OS(2) C1[X(C17H21BrN3OS)] #Atoms= 44

Charge = 0 Multiplicity = 2

SCF Energy= -3869.01504426 Predicted Change= -3.264409D-03

==

Optimization incomplete.

Item	Max Val.	Criteria	Pass?	RMS Val.	Criteria	Pass?
Force	0.01965 ||	0.00045	[NO]	0.00245 ||	0.00030	[NO]
Displ	0.22774 ||	0.00180	[NO]	0.22774 ||	0.00180	[NO]

Atomic Type	Coordinates (Angstroms) X	Y	Z
C	-0.306344	1.463795	-0.232477
N	-1.223850	0.389776	-0.492304
C	1.054035	1.138507	-0.529462

N	2.112044	1.811128	-0.053226
C	3.423287	1.143187	0.069338
H	3.294729	0.147083	0.515447
H	3.862912	0.998025	-0.924109
C	2.098066	3.100009	0.641351
O	-0.768622	2.533357	0.167901
C	-0.937397	-1.085136	-0.411895
C	-0.540801	-1.467530	0.995337
C	-2.282404	-1.677288	-0.883276
C	-2.542981	0.567707	-0.527944
C	-4.597142	-0.439731	-1.063210
H	-5.202438	-1.241370	-0.628919
H	-4.650751	-0.507646	-2.160178
C	-5.031755	0.928905	-0.549692
H	-5.751298	1.412457	-1.213866
H	-5.451748	0.871902	0.458715
S	-3.509289	1.991025	-0.452699
C	-1.386119	-1.199401	2.077154
C	-1.018457	-1.570379	3.364135
C	0.198067	-2.213073	3.585279
C	1.042611	-2.480122	2.513717
C	0.677118	-2.108841	1.221852
H	1.299278	0.142754	-0.903994
H	1.675027	2.984109	1.653428
H	1.478601	3.824369	0.105206
C	3.574015	3.454817	0.701984
H	3.797219	4.190317	1.482151
H	3.894059	3.868305	-0.265176
C	4.230749	2.098075	0.942645
H	5.297832	2.081411	0.697540
H	4.126553	1.820278	2.000713
H	-0.135494	-1.347594	-1.117001
H	-2.571532	-2.561029	-0.305609
H	-2.270070	-1.923939	-1.954418

H	-2.342724	-0.699487	1.921236
H	-1.684158	-1.357405	4.198388
H	0.485744	-2.502630	4.594360
H	1.996019	-2.978419	2.680664
H	1.344524	-2.301011	0.377885
N	-3.209390	-0.573631	-0.646155
Br	2.575040	-1.895875	-1.955991

Statistical Thermodynamic Analysis

Temperature= 323.150 Kelvin   Pressure= 1.00000 Atm

==

SCF Energy=   -3869.01504426   Predicted Change= -3.264409D-03

Zero-point correction (ZPE)=   -3868.6539   0.36111

Internal Energy (U)=   -3868.6292   0.38579

Enthalpy (H)=   -3868.6282   0.38682

Gibbs Free Energy (G)=   -3868.7149   0.30008

Entropy (S)=   0.0002684

Frequencies --   -30.5835   21.9935   30.4130

Supporting Information: **[2,3] Radical Recombination Amide Distortion Interaction Ground State**

Using Gaussian 16: ES64L-G16RevA.03 25-Dec-2016

==

#m06/6-31g(d) scf=(maxcycle=300,direct,vshift=200,tight,yqc)

density=current scrf=(pcm,solvent=ch3cn) opt=(gdiis,maxcycle=250)

freq=noraman temperature=323.15

#N Geom=AllCheck Guess=TCheck SCRF=Check GenChk UM06/6-31G(d) Freq

----

Pointgroup= C1   Stoichiometry= C17H21BrN3OS(2)   C1[X(C17H21BrN3OS)] #Atoms= 44

Charge = 0   Multiplicity = 2

SCF Energy= -3869.02103566 Predicted Change= -2.524039D-08

==

Optimization completed on the basis of negligible forces. {Found 2 times}

Item	Max Val.		Criteria	Pass?	RMS Val.		Criteria	Pass?
Force	0.00000	||	0.00045	[YES]	0.00000	||	0.00030	[YES]
Displ	0.00975	||	0.00180	[NO]	0.00975	||	0.00180	[NO]

Atomic Type	Coordinates (Angstroms) X	Y	Z
C	-0.139064	1.436088	0.079540
N	-1.141052	0.478907	-0.317573
C	1.184863	0.973620	-0.081510
N	2.269403	1.667870	0.277744
C	3.620133	1.161617	0.007738
H	3.632510	0.068093	0.088306
H	3.901453	1.417470	-1.026437
C	2.303481	2.928566	1.028120
O	-0.533823	2.546834	0.463037
C	-0.987012	-1.006385	-0.480820
C	-0.701864	-1.669460	0.845839
C	-2.348741	-1.378730	-1.106113
C	-2.427110	0.792410	-0.406692
C	-4.506017	0.100129	-1.240998
H	-5.235780	-0.678152	-0.997992
H	-4.444166	0.200034	-2.335476
C	-4.850432	1.420688	-0.565864
H	-5.482515	2.056612	-1.189209
H	-5.333416	1.268531	0.403997
S	-3.244145	2.300369	-0.255899
C	-1.583656	-1.530325	1.922195
C	-1.321098	-2.163685	3.130385
C	-0.176132	-2.944587	3.275099
C	0.703480	-3.085905	2.207572
C	0.444530	-2.450135	0.995772
H	1.394207	0.030477	-0.589843

H	1.879681	2.758386	2.029182
H	1.684175	3.684094	0.535088
C	3.787988	3.271590	1.081607
H	4.044301	3.847095	1.976734
H	4.065639	3.872320	0.204692
C	4.473767	1.909019	1.016862
H	5.524328	1.965784	0.714926
H	4.424651	1.408900	1.993941
H	-0.169947	-1.211530	-1.188348
H	-2.741004	-2.322078	-0.713878
H	-2.298137	-1.436404	-2.202815
H	-2.485754	-0.926185	1.820237
H	-2.013381	-2.048589	3.962301
H	0.028600	-3.440671	4.222147
H	1.601308	-3.691797	2.316619
H	1.140372	-2.542546	0.158025
N	-3.194970	-0.253629	-0.713935
Br	2.361812	-1.751416	-2.136233

Statistical Thermodynamic Analysis

Temperature= 323.150 Kelvin Pressure= 1.00000 Atm

==

SCF Energy= -3869.02103566 Predicted Change= -2.524039D-08

Zero-point correction (ZPE)= -3868.6596 0.36139

Internal Energy (U)= -3868.6341 0.38691

Enthalpy (H)= -3868.6330 0.38793

Gibbs Free Energy (G)= -3868.7229 0.29805

Entropy (S)= 0.00027813

Frequencies -- 16.6731 19.3907 28.9298

Supporting Information: **Dimethyl Amine [1,2] Radical Recombination Allyl TS Distortion**

Using Gaussian 16: ES64L-G16RevA.03 25-Dec-2016

==

#m06/6-31g(d) scf=(maxcycle=300,direct,vshift=200,tight,yqc)

density=current scrf=(pcm,solvent=ch3cn) freq=noraman temperature=323.15

Pointgroup= C1 Stoichiometry= C10H11(2) C1[X(C10H11)] #Atoms= 21

Charge = 0 Multiplicity = 2

SCF Energy= -387.325950878 Predicted Change= -1.681817D-03

==

Optimization incomplete.

Item	Max Val.	Criteria	Pass?	RMS Val.	Criteria	Pass?
Force	0.02346 ||	0.00045	[NO]	0.00425 ||	0.00030	[NO]
Displ	0.13553 ||	0.00180	[NO]	0.13553 ||	0.00180	[NO]

Atomic Type	Coordinates (Angstroms) X	Y	Z
C	3.527028	-0.812410	0.008851
H	4.068960	-1.724247	-0.236917
H	4.107677	0.100779	0.137903
C	2.147137	-0.821757	-0.113625
C	1.269129	0.255413	0.004204
C	1.786507	1.656744	0.127137
H	2.868160	1.684695	0.283986
H	1.568914	2.246024	-0.776743
H	1.321634	2.185731	0.971644
C	-0.178000	0.064172	-0.030227
C	-1.050538	1.170072	-0.100051
C	-0.777402	-1.210918	0.056124
C	-2.431130	1.011015	-0.076871
H	-0.642776	2.175851	-0.176265
C	-2.154267	-1.368948	0.084339
H	-0.153861	-2.101730	0.113976

C	-2.995959	-0.258086	0.023476
H	-3.071147	1.889956	-0.135953
H	-2.575197	-2.370779	0.161579
H	-4.076728	-0.381917	0.049513
H	1.729339	-1.816147	-0.292856

Statistical Thermodynamic Analysis

Temperature= 323.150 Kelvin Pressure= 1.00000 Atm

==

SCF Energy= -387.325950878 Predicted Change= -1.681817D-03

Zero-point correction (ZPE)= -387.1498 0.17612

Internal Energy (U)= -387.1389 0.18696

Enthalpy (H)= -387.1379 0.18798

Gibbs Free Energy (G)= -387.1891 0.13675

Entropy (S)= 0.00015853

Frequencies -- 28.4536 130.4222 165.5062

Supporting Information: **Dimethyl Amine [1,2] Radical Recombination Allyl Distortion Interaction Ground State**

Using Gaussian 16: ES64L-G16RevA.03 25-Dec-2016

==

#m06/6-31g(d) scf=(maxcycle=300,direct,vshift=200,tight,yqc)

density=current scrf=(pcm,solvent=ch3cn) opt=(gdiis,maxcycle=250)

freq=noraman temperature=323.15

#N Geom=AllCheck Guess=TCheck SCRF=Check GenChk UM06/6-31G(d) Freq

----

Pointgroup= C1 Stoichiometry= C10H11(2) C1[X(C10H11)] #Atoms= 21

Charge = 0 Multiplicity = 2

SCF Energy= -387.327669482 Predicted Change= -1.727898D-09

==

Optimization completed. {Found 2 times}

Item	Max Val.	Criteria	Pass?	RMS Val.	Criteria	Pass?
Force	0.00001 ||	0.00045	[YES]	0.00000 ||	0.00030	[YES]
Displ	0.00064 ||	0.00180	[YES]	0.00064 ||	0.00180	[YES]

Atomic Type	Coordinates (Angstroms) X	Y	Z
C	3.513518	-0.805087	-0.120644
H	4.084505	-1.717024	-0.278115
H	4.090691	0.105071	0.030775
C	2.148726	-0.836410	-0.120934
C	1.262333	0.254647	0.044309
C	1.794387	1.646122	0.196595
H	2.861515	1.656351	0.435513
H	1.664471	2.235586	-0.724384
H	1.274578	2.191535	0.995415
C	-0.177181	0.063526	0.020798
C	-1.044798	1.170049	-0.112183
C	-0.781678	-1.208805	0.137185
C	-2.422985	1.013637	-0.143515
H	-0.629760	2.170982	-0.210661
C	-2.158595	-1.360780	0.110103
H	-0.165640	-2.094258	0.279800
C	-2.993150	-0.252394	-0.033785
H	-3.058649	1.890500	-0.256520
H	-2.588021	-2.356380	0.210668
H	-4.074310	-0.375231	-0.054509
H	1.697163	-1.814166	-0.295552

Statistical Thermodynamic Analysis

Temperature= 323.150 Kelvin Pressure= 1.00000 Atm

===

SCF Energy= -387.327669482 Predicted Change= -1.727898D-09

Zero-point correction (ZPE)= -387.1517 0.17596

Internal Energy (U)= -387.1406 0.18702

Enthalpy (H)= -387.1396 0.18804

Gibbs Free Energy (G)= -387.1915 0.13609

Entropy (S)= 0.00016075

Frequencies -- 29.9486 102.1899 123.8715

Supporting Information: **Dimethyl Amine [1,2] Radical Recombination Amide TS Distortion**

Using Gaussian 16: ES64L-G16RevA.03 25-Dec-2016

==

#m06/6-31g(d) scf=(maxcycle=300,direct,vshift=200,tight,yqc)

density=current scrf=(pcm,solvent=ch3cn) freq=noraman temperature=323.15

Pointgroup= C1 Stoichiometry= C15H19BrN3OS(2) C1[X(C15H19BrN3OS)] #Atoms= 40

Charge = 0 Multiplicity = 2

SCF Energy= -3791.65153933 Predicted Change= -1.549123D-03

==

Optimization incomplete.

Item	Max Val.	Criteria	Pass?	RMS Val.	Criteria	Pass?
Force	0.01279 ||	0.00045	[NO]	0.00201 ||	0.00030	[NO]
Displ	0.10025 ||	0.00180	[NO]	0.10025 ||	0.00180	[NO]

Atomic Type	Coordinates (Angstroms) X	Y	Z
C	0.356836	-1.437446	0.730349
N	0.974966	-0.443308	-0.094050
C	-1.066648	-1.422984	0.664492
N	-1.904468	-2.090108	1.477703
O	1.105444	-2.197715	1.356184

C	0.353180	0.794003	-0.671419
C	-0.150359	1.706975	0.419016
C	1.522635	1.401725	-1.491019
C	2.275189	-0.465828	-0.380273
C	4.059544	0.756685	-1.316675
H	4.333711	1.572914	-0.631065
H	4.313452	1.051157	-2.341601
C	4.720309	-0.564433	-0.943562
H	4.854116	-1.209641	-1.819481
H	5.679879	-0.424715	-0.441157
S	3.568346	-1.435696	0.223555
C	0.698434	2.100359	1.457458
C	0.246729	2.973247	2.438948
C	-1.056628	3.464305	2.388158
C	-1.904689	3.073566	1.357512
C	-1.456029	2.194501	0.374882
H	-1.555577	-0.739718	-0.027913
H	-0.474173	0.502158	-1.336704
H	1.771925	2.417217	-1.155280
H	1.316339	1.432878	-2.567790
H	1.720844	1.720035	1.505385
H	0.912819	3.272184	3.246082
H	-1.410139	4.149304	3.156652
H	-2.925522	3.449505	1.318703
H	-2.121947	1.856074	-0.422270
N	2.632897	0.500625	-1.213108
Br	-2.905244	-0.118924	-2.201084
C	-1.495188	-3.111568	2.422282
H	-2.160498	-3.072975	3.293049
H	-1.581789	-4.111855	1.967771
H	-0.463005	-2.952635	2.732703
C	-3.323079	-2.080623	1.165223
H	-3.637479	-3.077842	0.819835
H	-3.904281	-1.827533	2.061118

H -3.527394 -1.356685 0.367498

Statistical Thermodynamic Analysis

Temperature= 323.150 Kelvin Pressure= 1.00000 Atm

==

SCF Energy= -3791.65153933 Predicted Change= -1.549123D-03

Zero-point correction (ZPE)= -3791.3268 0.32464

Internal Energy (U)= -3791.3021 0.34938

Enthalpy (H)= -3791.3011 0.35040

Gibbs Free Energy (G)= -3791.3880 0.26344

Entropy (S)= 0.00026911

Frequencies -- 21.8964 30.5068 41.2899

Supporting Information: **Dimethyl Amine [1,2] Radical Recombination Amide Distortion Interaction Ground State**

Using Gaussian 16: ES64L-G16RevA.03 25-Dec-2016

==

#m06/6-31g(d) scf=(maxcycle=300,direct,vshift=200,tight,yqc)

density=current scrf=(pcm,solvent=ch3cn) opt=(gdiis,maxcycle=250)

freq=noraman temperature=323.15

#N Geom=AllCheck Guess=TCheck SCRF=Check GenChk UM06/6-31G(d) Freq

----

Pointgroup= C1 Stoichiometry= C15H19BrN3OS(2) C1[X(C15H19BrN3OS)] #Atoms= 40

Charge = 0 Multiplicity = 2

SCF Energy= -3791.65375659 Predicted Change= -1.936089D-08

==

Optimization completed. {Found 2 times}

Item Max Val. Criteria Pass? RMS Val. Criteria Pass?

Force 0.00001 || 0.00045 [YES] 0.00000 || 0.00030 [YES]

Displ 0.00163 || 0.00180 [YES] 0.00163 || 0.00180 [YES]

Atomic Type	Coordinates (Angstroms) X	Y	Z
C	0.347503	-1.472626	0.641515
N	0.962107	-0.487979	-0.214253
C	-1.063758	-1.477331	0.555620
N	-1.902902	-2.218070	1.296389
O	1.108129	-2.199934	1.298649
C	0.357806	0.797133	-0.703245
C	-0.071723	1.656167	0.460649
C	1.516252	1.404344	-1.537780
C	2.265998	-0.483533	-0.463976
C	4.069558	0.774097	-1.296124
H	4.328846	1.527442	-0.536106
H	4.357588	1.153500	-2.281474
C	4.707857	-0.578367	-1.008651
H	4.824284	-1.173919	-1.919150
H	5.674009	-0.488042	-0.507857
S	3.543756	-1.485248	0.119455
C	0.848482	2.014703	1.450223
C	0.460826	2.829417	2.506303
C	-0.850455	3.295457	2.581426
C	-1.769771	2.938047	1.601171
C	-1.384944	2.118143	0.543103
H	-1.550457	-0.880643	-0.217412
H	-0.503847	0.570265	-1.348766
H	1.755437	2.427232	-1.220803
H	1.302288	1.409796	-2.611854
H	1.878571	1.654598	1.404115
H	1.183177	3.102140	3.273273
H	-1.153964	3.934778	3.408428
H	-2.796611	3.294577	1.660255
H	-2.105801	1.808882	-0.217789

N	2.638244	0.514957	-1.252297
Br	-3.049417	0.080827	-2.109458
C	-1.518587	-3.068325	2.407404
H	-1.965086	-2.682106	3.334184
H	-1.906391	-4.081741	2.239174
H	-0.433404	-3.099540	2.499815
C	-3.332633	-2.126830	1.056275
H	-3.740541	-3.130854	0.880044
H	-3.832732	-1.703070	1.938172
H	-3.527522	-1.494136	0.181932

Statistical Thermodynamic Analysis

Temperature= 323.150 Kelvin Pressure= 1.00000 Atm

==

SCF Energy= -3791.65375659 Predicted Change= -1.936089D-08

Zero-point correction (ZPE)= -3791.3287 0.32505

Internal Energy (U)= -3791.3041 0.34962

Enthalpy (H)= -3791.3031 0.35064

Gibbs Free Energy (G)= -3791.3891 0.26463

Entropy (S)= 0.00026617

Frequencies -- 25.6564 32.9326 44.8476

Supporting Information: **Dimethyl Amine [2,3] Radical Recombination Allyl TS Distortion**

Using Gaussian 16: ES64L-G16RevA.03 25-Dec-2016

==

#m06/6-31g(d) scf=(maxcycle=300,direct,vshift=200,tight,yqc)

density=current scrf=(pcm,solvent=ch3cn) freq=noraman temperature=323.15

Pointgroup= C1 Stoichiometry= C10H11(2) C1[X(C10H11)] #Atoms= 21

Charge = 0 Multiplicity = 2

SCF Energy= -387.321748910 Predicted Change= -4.678524D-03

==

Optimization incomplete.

Item	Max Val.		Criteria	Pass?	RMS Val.		Criteria	Pass?
Force	0.02818	||	0.00045	[NO]	0.00580	||	0.00030	[NO]
Displ	0.21705	||	0.00180	[NO]	0.21705	||	0.00180	[NO]

Atomic Type	Coordinates (Angstroms) X	Y	Z
C	-2.143819	-0.864261	0.065044
C	-1.271326	0.231000	-0.274066
C	-3.472507	-0.784269	0.279346
H	-4.027467	0.152173	0.293210
H	-4.050522	-1.685555	0.476027
H	-1.708640	-1.864752	0.062771
C	-1.818966	1.618949	-0.159256
H	-2.880864	1.653715	-0.422959
H	-1.725204	1.992472	0.872721
H	-1.303188	2.328574	-0.817832
C	0.181699	0.059062	-0.137694
C	1.021995	1.171100	0.058746
C	0.801647	-1.207450	-0.199571
C	2.399851	1.031061	0.191413
H	0.596801	2.170485	0.121595
C	2.175749	-1.343124	-0.064411
H	0.212409	-2.100036	-0.414145
C	2.989997	-0.226973	0.134635
H	3.013953	1.918966	0.341497
H	2.619160	-2.337083	-0.120717
H	4.067635	-0.339533	0.242721

Statistical Thermodynamic Analysis

Temperature= 323.150 Kelvin Pressure= 1.00000 Atm

==

SCF Energy= -387.321748910 Predicted Change= -4.678524D-03

Zero-point correction (ZPE)= -387.1456 0.17605

Internal Energy (U)= -387.1347 0.18699

Enthalpy (H)= -387.1337 0.18802

Gibbs Free Energy (G)= -387.1851 0.13656

Entropy (S)= 0.00015923

Frequencies -- 43.2898 79.8747 156.3720

Supporting Information: **Dimethyl Amine [2,3] Radical Recombination Allyl Distortion Interaction Ground State**

Using Gaussian 16: ES64L-G16RevA.03 25-Dec-2016

==

#m06/6-31g(d) scf=(maxcycle=300,direct,vshift=200,tight,yqc)

density=current scrf=(pcm,solvent=ch3cn) opt=(gdiis,maxcycle=250)

freq=noraman temperature=323.15

#N Geom=AllCheck Guess=TCheck SCRF=Check GenChk UM06/6-31G(d) Freq

----

Pointgroup= C1 Stoichiometry= C10H11(2) C1[X(C10H11)] #Atoms= 21

Charge = 0 Multiplicity = 2

SCF Energy= -387.327669482 Predicted Change= -3.970400D-09

==

Optimization completed. {Found 2 times}

Item	Max Val.	Criteria	Pass?	RMS Val.	Criteria	Pass?
Force	0.00001 ||	0.00045	[YES]	0.00000 ||	0.00030	[YES]
Displ	0.00146 ||	0.00180	[YES]	0.00146 ||	0.00180	[YES]

Atomic Coordinates (Angstroms)

Type X Y Z

C	-2.148734	-0.836425	0.120782
C	-1.262328	0.254655	-0.044273
C	-3.513531	-0.805090	0.120563
H	-4.090721	0.105097	-0.030588
H	-4.084484	-1.717091	0.277799
H	-1.697198	-1.814241	0.295126
C	-1.794435	1.646137	-0.196391
H	-2.861647	1.656336	-0.434926
H	-1.664156	2.235607	0.724521
H	-1.274955	2.191538	-0.995440
C	0.177191	0.063543	-0.020784
C	1.044827	1.170066	0.112056
C	0.781676	-1.208803	-0.137061
C	2.423016	1.013640	0.143370
H	0.629831	2.171028	0.210427
C	2.158593	-1.360792	-0.109976
H	0.165649	-2.094281	-0.279564
C	2.993170	-0.252406	0.033771
H	3.058680	1.890519	0.256255
H	2.587994	-2.356417	-0.210412
H	4.074329	-0.375253	0.054460

Statistical Thermodynamic Analysis

Temperature= 323.150 Kelvin   Pressure= 1.00000 Atm

==

SCF Energy= -387.327669482 Predicted Change= -3.970400D-09

Zero-point correction (ZPE)= -387.1516 0.17597

Internal Energy (U)= -387.1406 0.18702

Enthalpy (H)= -387.1396 0.18804

Gibbs Free Energy (G)= -387.1915 0.13610

Entropy (S)= 0.00016073

Frequencies -- 30.0947 102.2077 124.0405

Supporting Information: **Dimethyl Amine [2,3] Radical Recombination Amide TS Distortion**

Using Gaussian 16: ES64L-G16RevA.03 25-Dec-2016

==

#m06/6-31g(d) scf=(maxcycle=300,direct,vshift=200,tight,yqc)

density=current scrf=(pcm,solvent=ch3cn) freq=noraman temperature=323.15

Pointgroup= C1 Stoichiometry= C15H19BrN3OS(2) C1[X(C15H19BrN3OS)] #Atoms= 40

Charge = 0 Multiplicity = 2

SCF Energy= -3791.64924848 Predicted Change= -2.816925D-03

==

Optimization incomplete.

Item	Max Val.	Criteria	Pass?	RMS Val.	Criteria	Pass?
Force	0.01818 ||	0.00045	[NO]	0.00219 ||	0.00030	[NO]
Displ	0.21361 ||	0.00180	[NO]	0.21361 ||	0.00180	[NO]

Atomic Type	Coordinates (Angstroms) X	Y	Z
C	-0.423824	1.596167	0.424179
N	-1.006858	0.504743	-0.307790
C	0.991001	1.696731	0.252589
N	1.846615	2.376617	1.042214
O	-1.190892	2.325974	1.058171
C	-0.349417	-0.798385	-0.675207
C	0.065628	-1.541957	0.573346
C	-1.466120	-1.477594	-1.496020
C	-2.322833	0.379859	-0.469368
C	-4.007262	-0.805384	-1.598829
H	-4.407014	-1.822878	-1.550832
H	-3.974413	-0.484069	-2.651221
C	-4.814096	0.154345	-0.730823

H	-5.594755	0.668032	-1.296150
H	-5.263652	-0.351707	0.128546
S	-3.630680	1.424671	-0.067189
C	-0.873287	-1.876252	1.554838
C	-0.480479	-2.559971	2.698317
C	0.854914	-2.917058	2.874520
C	1.792033	-2.587136	1.901917
C	1.401610	-1.901199	0.754286
H	1.481334	0.977521	-0.402910
H	0.527162	-0.608866	-1.311656
H	-1.546057	-2.548219	-1.282052
H	-1.330430	-1.326615	-2.577189
H	-1.922181	-1.604004	1.431434
H	-1.219371	-2.814840	3.455705
H	1.162072	-3.451046	3.771949
H	2.837116	-2.861229	2.035282
H	2.135542	-1.629146	-0.008548
N	-2.660307	-0.766169	-1.048247
Br	3.211932	-0.267299	-2.041639
C	1.446257	3.247310	2.131771
H	1.570751	2.732695	3.097796
H	2.089057	4.137741	2.135099
H	0.403127	3.544277	2.019247
C	3.227325	1.920241	1.113841
H	3.897366	2.783280	1.207257
H	3.364868	1.272399	1.995988
H	3.485010	1.354965	0.209886

Statistical Thermodynamic Analysis

Temperature= 323.150 Kelvin Pressure= 1.00000 Atm

===

SCF Energy= -3791.64924848 Predicted Change= -2.816925D-03

Zero-point correction (ZPE)= -3791.3241 0.32508

Internal Energy (U)= -3791.2997 0.34945

Enthalpy (H)= -3791.2987 0.35047

Gibbs Free Energy (G)= -3791.3838 0.26537

Entropy (S)= 0.00026335

Frequencies -- 29.0334 35.4322 46.0016

Supporting Information: **Dimethyl Amine [2,3] Radical Recombination Amide Distortion Interaction Ground State**

Using Gaussian 16: ES64L-G16RevA.03 25-Dec-2016

==

#m06/6-31g(d) scf=(maxcycle=300,direct,vshift=200,tight,yqc)

density=current scrf=(pcm,solvent=ch3cn) opt=(gdiis,maxcycle=250)

freq=noraman temperature=323.15

#N Geom=AllCheck Guess=TCheck SCRF=Check GenChk UM06/6-31G(d) Freq

----

Pointgroup= C1 Stoichiometry= C15H19BrN3OS(2) C1[X(C15H19BrN3OS)] #Atoms= 40

Charge = 0 Multiplicity = 2

SCF Energy= -3791.65404731 Predicted Change= -2.295008D-08

==

Optimization completed. {Found 1 times}

Item	Max Val.	Criteria	Pass?	RMS Val.	Criteria	Pass?
Force	0.00000 ||	0.00045	[YES]	0.00000 ||	0.00030	[YES]
Displ	0.00273 ||	0.00180	[NO]	0.00273 ||	0.00180	[YES]

Atomic Type	Coordinates (Angstroms) X	Y	Z
C	-0.332084	1.508374	0.645751
N	-0.959577	0.487236	-0.155311
C	1.081061	1.469027	0.603968
N	1.913580	2.258872	1.300315
O	-1.084796	2.306629	1.224309

C	-0.352679	-0.780464	-0.686976
C	0.033574	-1.710522	0.438230
C	-1.492649	-1.302112	-1.588214
C	-2.271675	0.455058	-0.356280
C	-3.981033	-0.489941	-1.654021
H	-4.453056	-1.473070	-1.743610
H	-3.880011	-0.051388	-2.658538
C	-4.755463	0.419055	-0.709660
H	-5.508974	1.015977	-1.227665
H	-5.231672	-0.144259	0.098428
S	-3.517621	1.566119	0.065926
C	-0.911691	-2.130215	1.379184
C	-0.549273	-3.006960	2.393974
C	0.760368	-3.475526	2.477502
C	1.703815	-3.060586	1.544233
C	1.344473	-2.179483	0.527282
H	1.578574	0.805631	-0.103979
H	0.530550	-0.533673	-1.294920
H	-1.608220	-2.388433	-1.523269
H	-1.351296	-1.015977	-2.640198
H	-1.941048	-1.774549	1.322258
H	-1.292014	-3.327259	3.122213
H	1.043187	-4.162714	3.272906
H	2.729395	-3.420053	1.607686
H	2.084359	-1.833010	-0.198516
N	-2.663017	-0.615515	-1.047334
Br	3.083024	-0.084442	-2.036952
C	1.520644	3.188965	2.342690
H	1.943678	2.860057	3.302137
H	1.928146	4.182275	2.113385
H	0.434778	3.242495	2.411114
C	3.346960	2.132658	1.099628
H	3.775857	3.124054	0.904297
H	3.818566	1.727013	2.005494

H 3.550689 1.473294 0.247388

Statistical Thermodynamic Analysis

Temperature= 323.150 Kelvin Pressure= 1.00000 Atm

==

SCF Energy= -3791.65404731 Predicted Change= -2.295008D-08

Zero-point correction (ZPE)= -3791.3289 0.32505

Internal Energy (U)= -3791.3044 0.34959

Enthalpy (H)= -3791.3034 0.35062

Gibbs Free Energy (G)= -3791.3892 0.26474

Entropy (S)= 0.00026573

Frequencies -- 27.3727 38.3747 41.2007

H. 2. Transition States

C-N bond homolysis of the acyl ammonium ylide.

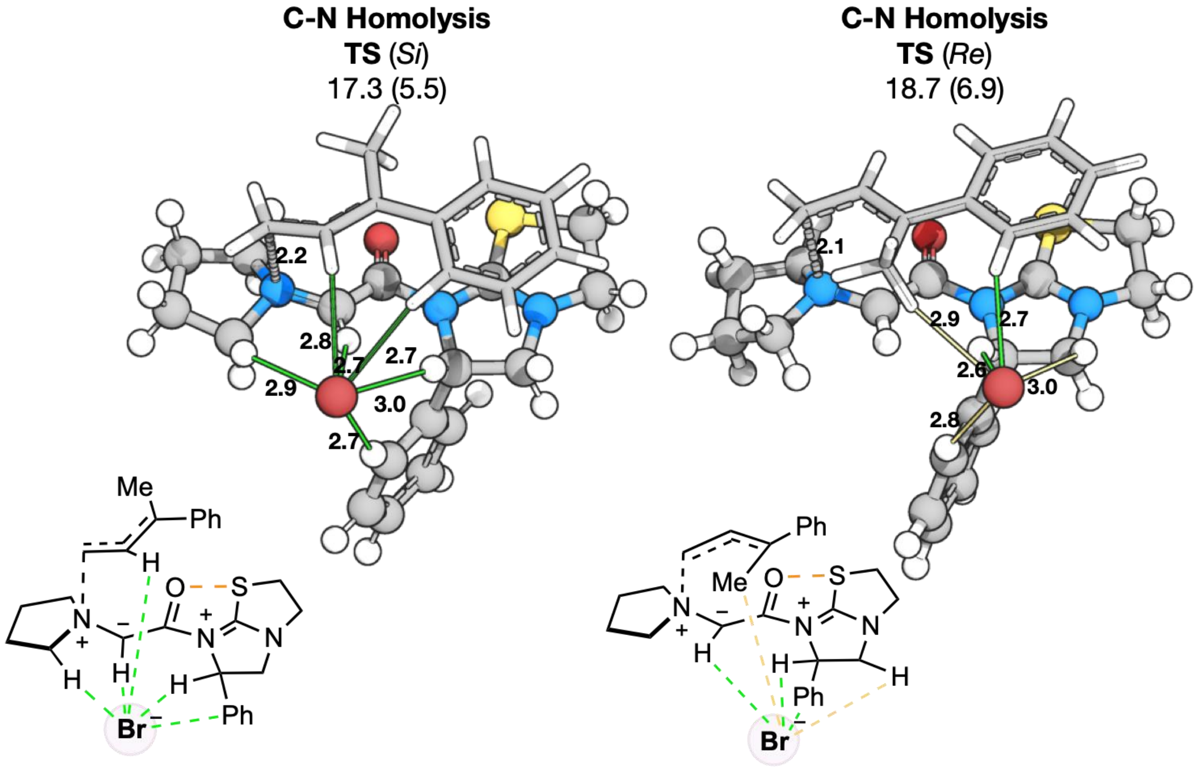

Figure S19: Comparison of the diastereoisomeric transition states for C-N bond homolytic cleavage that occur from either the *Si* or *Re* face of the planar allyl group.

Concerted [2,3] Transition State

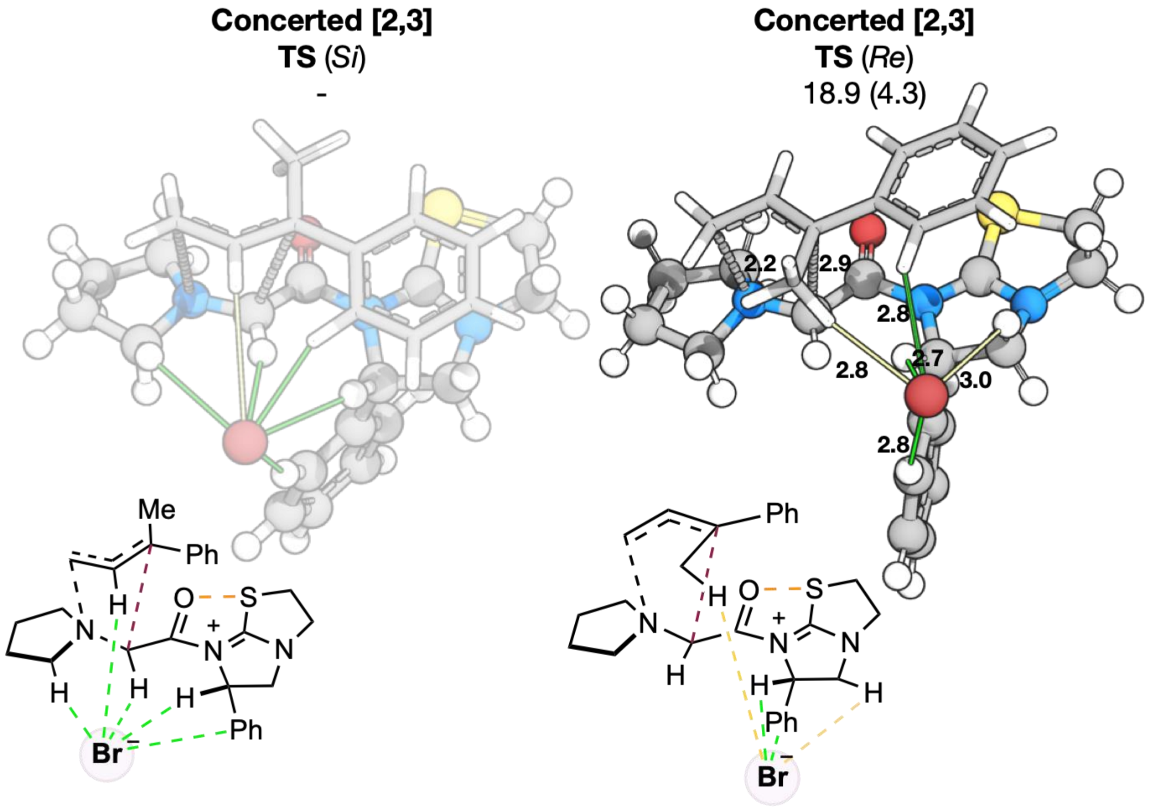

Figure S20: Comparison of the diastereoisomeric transition states for concerted [2,3]-sigmatropic rearrangement of the ammonium ylide that occur from either the *Si* or *Re* face of the planar allyl group.

[1,2] Radical Recombination Transition State

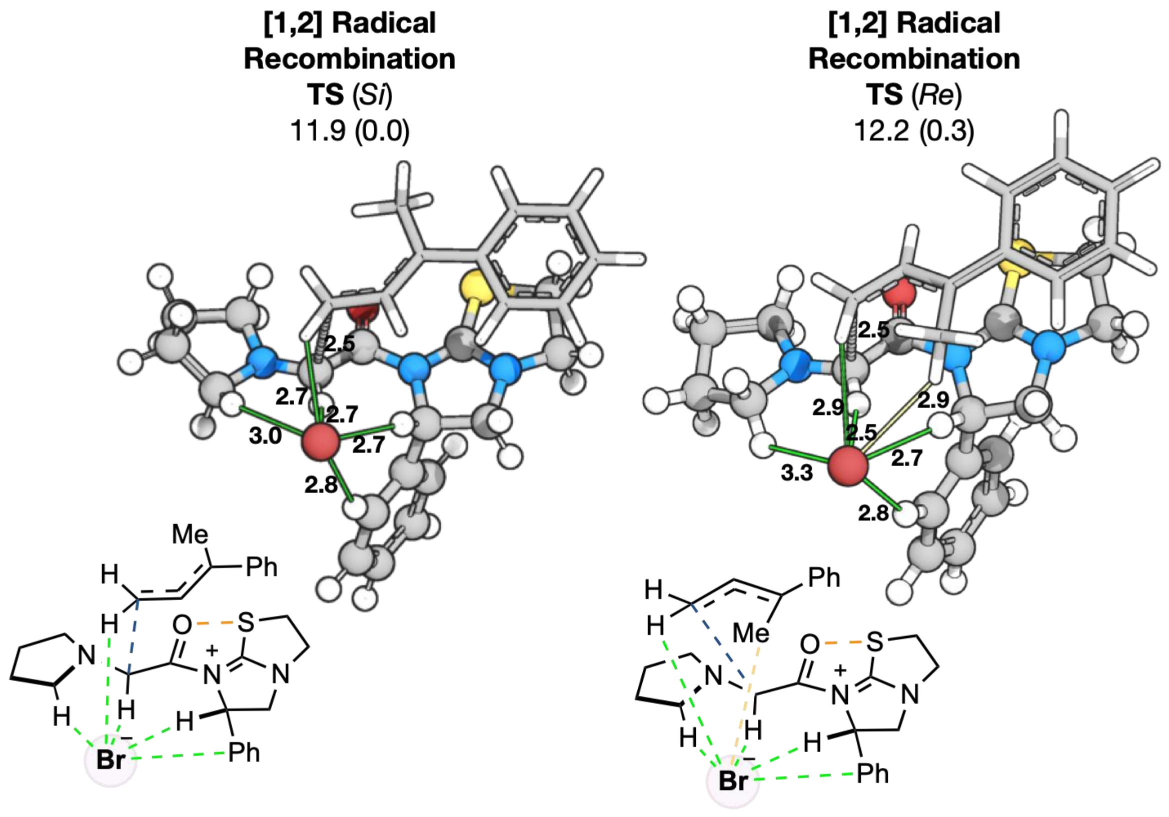

Figure S21: Comparison of the diastereoisomeric transition states for [1,2]-rearrangement C-C bond formation via radical-radical coupling that occur from either the Si or Re face of the planar allyl group.

[2,3] Radical Recombination Transition State

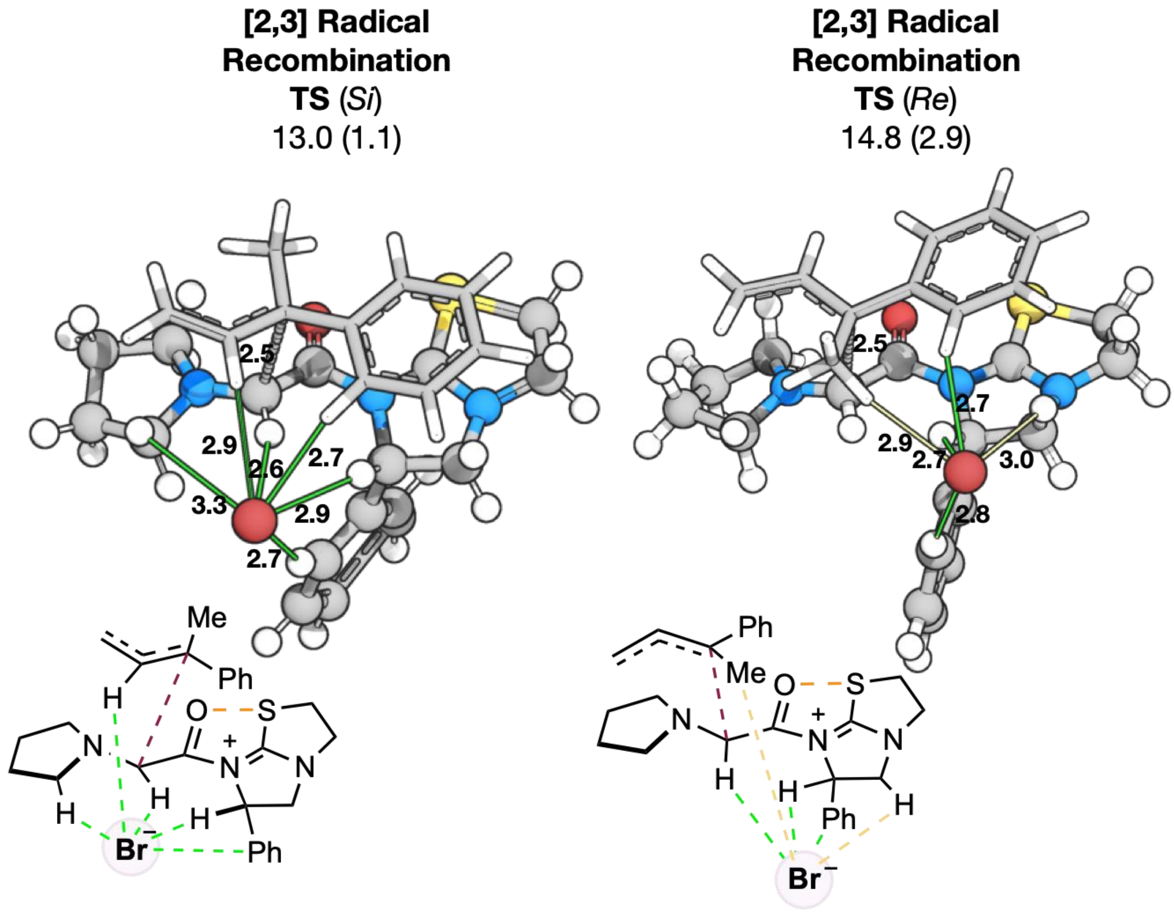

Figure S22: Comparison of the diastereoisomeric transition states for [2,3]-rearrangement C-C bond formation via radical-radical coupling that occur from either the Si or Re face of the planar allyl group.

H. 3. Juxtaposition to Acyclic N,N-Dimethylamino Substrate

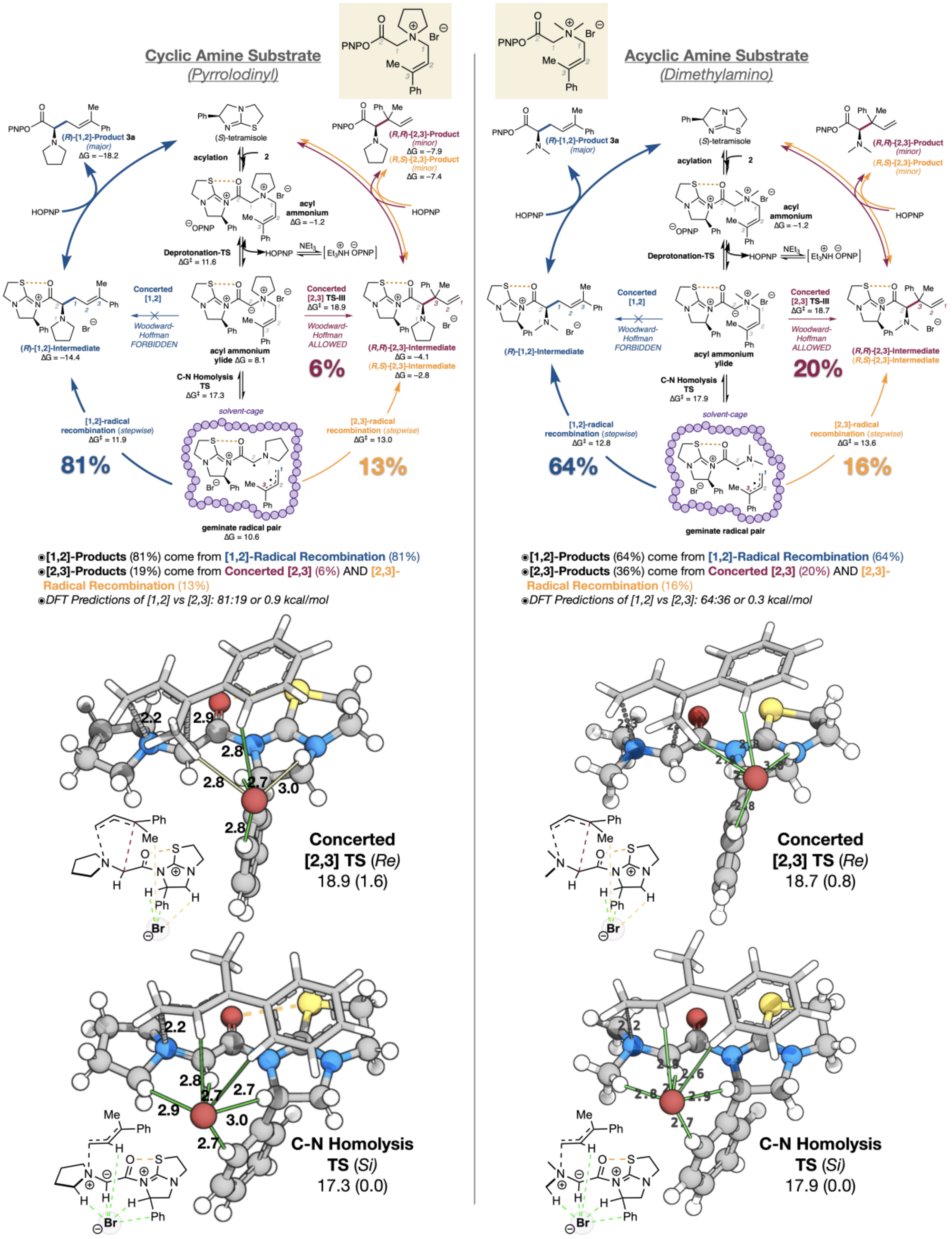

Figure S23: The catalytic cycles of the parent substrate (left) and the acyclic N,N-dimethyl amino substrate (right). [1,2] products arise exclusively from the **[1,2]-Radical Recombination** process, whereas the [2,3] product arises from both the **Concerted [2,3]** as well as the **[2,3]-Radical Recombination** processes. Energies given are kcal/mol.

We have expended significant effort into computationally understanding the effect resulting from change of pyrrolidinyl to N,N-dimethyl (Figure S23). Experimentally this changes the major product from [1,2] to the [2,3]. We have computed the four key transition structures for the N,N-dimethyl substrate. Computations are in agreement with experiments in that the N,N-

dimethyl substitution pattern exhibits significantly diminished 1,2 selectivity (64:36 or 0.3 kcal/mol) compared to pyrrolodinyl (81:19 or 0.9 kcal/mol). The computations reveal that the **Concerted-[2,3]** barrier slightly decreased while the **C-N Homolysis** barrier increased for the acyclic N,N-dimethyl substrate in comparison to the pyrrolodinyl. Together, these changes led to the increase of fraction of [2,3] products in the case of the N,N-dimethyl substrate.

Upon closer examination of the results, we believe these barrier changes arose from the diminished steric encumbrance of the N,N-dimethyl amine compared to the pyrrolodinyl. The less sterically encumbered dimethyl leads to a comparatively facile [2,3] process, hence the slightly lower barrier. The same diminished steric occlusion of the amine leads to a slightly more difficult C-N Homolysis step, as the reduction in steric crowding reduces the strain experienced by the acyl ammonium ylide that would be alleviated by the cleavage.

I. References

1. Daniels, D. S. B., Smith, S. R., Lebl, T., Shapland, P. & Smith, A. D. *Synthesis* **47**, 34–41 (2015).

2. Morrill, L. C., Douglas, J., Lebl, T., Slawin, A. M. Z., Fox, D. J. & Smith, A. D. Isothiourea-mediated asymmetric michael-lactonisation of trifluoromethylenones: a synthetic and mechanistic study, *Chem. Sci.* **4**, 4146-4155 (2013).

3. Fulmer, G. R., Miller, A. J. M., Sherden, N. H., Gottlieb, H. E., Nudelman, A., Stoltz, B. M., Bercaw, J. E. & Goldberg, K. I. NMR chemical shifts of trace impurities: common laboratory solvents, organics, and gases in deuterated solvents relevant to the organometallic chemist. *Organometallics* **29**, 2176–2179 (2010).

4. Wu, R., Beauchamps, M. G., Laquidara, J. M. & Sowa Jr., J. R. Ruthenium-catalyzed redox isomerization of trifluoromethylated allylic alcohols: mechanistic evidence for an enantiospecific pathway. *Angew. Chem. Int. Ed.*, **51**, 2106–2110 (2012).

5. Metternich, J. B. & Gilmour, R. A bio-inspired, catalytic E → Z isomerization of activated olefins *J. Am. Chem. Soc*. **137**, 11254–11257 (2015).

6. Bernasconi, M., Ramella, V., Tosatti, P. & Pfaltz, A. Iridium-catalyzed asymmetric hydrogenation of 3,3-disubstituted allylic alcohols in ethereal solvents. *Chem. Eur. J.* **20**, 2440–2444 (2014).

7. Cui, H., Lia, Y. & Zhang, S. A novel ketone olefination via organozinc reagents in the presence of diphenyl phosphite. *Org. Biomol. Chem.* **10**, 2862–2869 (2012).

8. Guchhait, S. K. & Priyadarshani, G. Synthesis of 2-arylpyridopyrimidinones, 6-aryluracils, and tri- and tetrasubstituted conjugated alkenes via Pd-catalyzed enolic C–O bond activation–arylation. *J. Org. Chem*. **80**, 6342–6349 (2015).

9. Chen, Y., Huang, L. & Zhang, X. P. *Org. Lett.* **5**, 2493–2496 (2003).

10. Wang, P., Liu, C.-R., Sun, X.-L., Chen, S.-S., Li, J.-F., Xieb, Z & Tang, Y. A newly-designed PE-supported arsine for efficient and practical catalytic Wittig olefination. *Chem. Commun.* **48**, 290–292 (2012).

11. Ren, K., Hu, B., Zhao, M., Tu, Y., Xie, X. & Zhang, Z. Ruthenium-catalyzed oxidation of allyl alcohols with intermolecular hydrogen transfer: synthesis of α,β-unsaturated carbonyl compounds. *J. Org. Chem.* **79**, 2170–2177 (2014).

12. Arai, N., Sato, K., Azuma, K. & Ohkuma, T. Enantioselective isomerization of primary allylic alcohols into chiral aldehydes with the tol-binap/dbapen/Ruthenium(II) catalyst. *Angew. Chem. Int. Ed*. **52**, 7500–7504 (2013).

13. Hayashi T. & Ishigedani M. Rhodium-catalyzed asymmetric arylation of α,β-unsaturated imines with arylstannanes. Catalytic asymmetric synthesis of allylic amines. *Tetrahedron* **57**, 2589–2595 (2001).

14. Zheng H., Lejkowski M. & Hall D. G. Mild and selective boronic acid catalyzed 1,3-transposition of allylic alcohols and Meyer–Schuster rearrangement of propargylic alcohols. *Chem. Sci.* **2**, 1305–1310 (2011).

15. Zi, W., Wang, Y.-M. & Toste, F. D. An in situ directing group strategy for chiral anion phase-transfer fluorination of allylic alcohols. *J. Am. Chem. Soc*. **136**, 12864–12867 (2014).

16. Matikonda, S. S., Fairhall, J. M., Tyndall, J. D. A., Hook, S. & Gamble, A. B. Stability, kinetic, and mechanistic investigation of 1,8-self-immolative cinnamyl ether spacers for controlled release of phenols and generation of resonance and inductively stabilized methides. *Org. Lett*. **19**, 528–531 (2017).

17. Nguyeb, T. N. T., Thiel, N. O. & Teichert, J. J. Copper(I)-catalysed asymmetric allylic reductions with hydrosilanes. *Chem. Commun*. **53**, 11686–11689 (2017).

18. Guduguntla, S., Gualtierotti, J.-B., Goh, S. S. & Feringa, B. L. Enantioselective synthesis of di- and tri-arylated all-carbon quaternary stereocenters via copper-catalyzed allylic arylations with organolithium compounds. *ACS Catal*. **6**, 6591–6595 (2016).

19. Reichl, K., Dunn, N. L., Fastuca, N. J. & Radosevich, A. T. Biphilic organophosphorus catalysis: regioselective reductive transposition of allylic bromides via P^{III}/P^{V} redox cycling. *J. Am. Chem. Soc*, **137**, 5292–5295 (2015).

20. Fañanás-Mastral, M., Pérez, M., Bos, P. H., Rudolph, A., Harutyunyan, S. R. & Feringa, B. L. Enantioselective synthesis of tertiary and quaternary stereogenic centers: copper/phosphoramidite-catalyzed allylic alkylation with organolithium reagents. *Angew. Chem. Int. Ed.* **51**, 1922–1925 (2012).

21. Beck, J. F., Samblanet, D. C. & Schmidt, J. A. R. Palladium catalyzed intermolecular hydroamination of 1-substituted allenes: an atom-economical method for the synthesis of *N*-allylamines. *RSC Adv*. **3**, 20708–20718 (2013).

22. Tani, K., Yamagata, T., Akutagwa, S., Kumobayashi, H., Taketomi, T., Takaya, H., Miyashita, A., Noyori, R. & Otsuka, S. Metal-assisted terpenoid synthesis. 7. Highly enantioselective isomerization of prochiral allylamines catalyzed by chiral diphosphine rhodium(I) complexes. Preparation of optically active enamines. *J. Am. Chem. Soc.* **106**, 5208–5217 (1984).

23. Kulchat, S. & Lehn, J.-M. Dynamic Covalent Chemistry of Nucleophilic Substitution Component Exchange of Quaternary Ammonium Salts. *Chem. Asian J.* **10**, 2484-2496 (2015).

24. Conte, M., Ma, Y., Loyns, C., Price, P., Rippon, D. & Chechik, V. Mechanistic insight into TEMPO-inhibited polymerisation: simultaneous determination of oxygen and inhibitor concentrations by EPR. *Org. Biomol. Chem.* **7**, 2685–2687 (2009).

25. Ayad, A., Belabbaci, A., Negadi, A., Hernández, A., Kabane, B., Bahadur, I., Mohammad, F., Soleiman, A. A. & Negadi, L. Measurements and modeling of thermodynamic properties of binary systems comprising (2-amino-2-methyl-1-propanol with acetonitrile, toluene, 1-pentanol or 1-hexanol) at different temperatures. *J. Chem. Eng. Data* **68**, 2789-2806 (2023).

26. Sadeghi, R. & Azizpour, S. Volumetric, compressibility, and viscometric measurements of binary mixtures of poly(vinylpyrrolidone) + water, + methanol, + ethanol, + acetonitrile, + 1-propanol, + 2-propanol, and + 1-butanol . *J. Chem. Eng. Data* **56**, 240–250 (2011).

27. Rohani, S., Horne, S. & Murthy, K. Control of product quality in batch crystallization of pharmaceuticals and fine chemicals. Part 1: Design of the crystallization process and the effect of solvent. *Org. Proc. Res. Dev.* **9**, 858–872 (2005).

28. Tachouaft, C., Damas, C. & Naejus, R. Effect of organic carbonate solvent composition on the volumetric and viscometric behavior of linear ethers. *J. Sol. Chem.* **52**, 1232–1254 (2023).

29. Schroeder, D. J., Hubaud, A. A. & Vaughey, J. T. Stability of the solid electrolyte Li_3OBr to common battery solvents. *Mater. Res. Bull.* **49**, 614-617 (2013).

30. Vraneš, M., Tot, A., Zec, N., Papović, S. & Gadžurić, S. Volumetric properties of binary mixtures of 1-butyl-3-methylimidazolium tris(pentafluoroethyl)trifluorophosphate with *N*-methylformamide, *N*-ethylformamide, *N*,*N*-dimethylformamide, *N*,*N*-dibutylformamide, and *N*,*N*-dimethylacetamide from (293.15 to 323.15) K. *J. Chem. Eng. Data* **59**, 3372–3379 (2014).

31. Fan, X.-H., Chen, Y.-P. & Su, C.-S. Density and viscosity measurements for binary mixtures of 1-ethyl-3-methylimidazolium tetrafluoroborate ([Emim][BF_4]) with dimethylacetamide, dimethylformamide, and dimethyl sulfoxide. *J. Chem. Eng. Data* **61**, 920-927 (2016).

32. Yue, X., Zhao, L., Ma, L., Shi, H., Yang, T., & Zhang, J.Density, dynamic viscosity, excess property and intermolecular interplay studies for 1,4-butanediol + dimethyl sulfoxide binary mixture. *J. Mol. Liq.* **263**, 40-48 (2018).

33. Agieienko, V. & Buchner, R. Variation of density, viscosity, and electrical conductivity of the deep eutectic solvent reline, composed of choline chloride and urea at a molar ratio of 1:2, mixed with dimethylsulfoxide as a cosolvent. *J. Chem. Eng. Data* **65**, 1900-1910 (2020).

34. Carrera, G. V. S. M., Afonso, C. A. M. & Branco, L. C. Interfacial properties, densities, and contact angles of task specific ionic liquids. *J. Chem. Eng. Data* **55**, 609-615 (2010).

35. Canongia Lopes, J. N., Costa Gomes, M. F., Husson, P., Pádua, A. A. H., Rebelo, L. P. N., Sarraute, S. & Tariq, M. polarity, viscosity, and ionic conductivity of liquid mixtures containing [C_4C_1im][NTf_2] and a molecular component. *J. Phys. Chem. B* **115**, 6088-6099 (2011).

36. Bravo-Sánchez, M. G., Guerrero-Zárate, D., Iglesias-Silva, G. A., Estrada-Baltazar, A., & Bouchot, C. P–ρ–T Data for 2-butanol and *tert*-butanol from 283.15 to 363.15 K and 303.15 to 363.15 K at pressures up to 66 MPa. *J. Chem. Eng. Data* **61**, 1555-1565 (2016).

37. Giner, B., Aldea, M. E., Martín, S., Gascón, I. & Lafuente, C. Viscosities of binary mixtures of isomeric butanols or isomeric chlorobutanes with 2-methyltetrahydrofuran. *J. Chem. Eng. Data*, **48**, 1296-1300 (2003).

38. Swain, B. B. Dielectric properties of binary mixtures of polar liquids, I. *Acta Chim, Hung.* **117**, 383-392 (1984).

39. Rodnikova, M. N., Gunina, M. A., Makarov, D. M., Egorov, G. I., & Val'kovskaya, T. M. Molar volumes of aqueous and ethylene glycol solutions of tetrahydrofuran. *Russ. J. Phys. Chem. A* **85**, 1676-1678 (2011).

40. Oke, E. A., Sharma, R., Malek, N. I. & Ijardar, S. P., Investigation on thermophysical properties of binary systems of [C4mim][NTf2] with cyclic ethers: Application of PFP and ERAS theories. *J. Mol. Liq.* **320**, 114411 (2020).

41. Li, X., Zhou, Q., Lu, X. & Zhang, S. Densities and viscosities of binary mixtures of magnetic ionic liquids 1-alkyl-3-methylimidazolium tetrachloroferrate with ethyl acetate at temperatures (293.15 to 323.15) K. *J. Mol. Liq.* **243**, 285-292 (2017).

42. Silva, A. A., Reis, R. A., & Paredes, M. L. L. Density and viscosity of decalin, cyclohexane, and toluene binary mixtures at (283.15, 293.15, 303.15, 313.15, and 323.15) K. *J. Chem. Eng. Data*, **54**, 2067-2072 (2009).

43. Pal, A., Kumar, H., Kumar, B. & Gaba, R. Density and speed of sound for binary mixtures of 1,4-dioxane with propanol and butanol isomers at different temperatures. *J. Mol. Liq.*, **187**, 278-286 (2013).

44. Gao, X., Hong, B., Zhang, M., & Li, D. Investigations on the thermophysical properties of binary systems of fatty acid esters + dimethyl carbonate. *J. Sol. Chem.* (2023) DOI:10.1007/s10953-023-01327-7.

45. Chen, F., Yang, Z., Chen, Z., Hu, J., Chen, C. & Cai, J. Density, viscosity, speed of sound, excess property and bulk modulus of binary mixtures of γ-butyrolactone with acetonitrile, dimethyl carbonate, and tetrahydrofuran at temperatures (293.15 to 333.15) K. *J. Mol. Liq.*, **209**, 683-692 (2015).

46. Iloukhani, H. & Khalarzadeh, K. Densities, viscosities, and refractive indicies for binary and ternary mixtures of N,N-dimethylacetamide (1) + 2-methylbutan-2-ol (2) + ethyl acetate (3) at 298.15 K for the liquid region and at ambient pressure. *J. Chem. Eng. Data* **51**, 1226–1231 (2006).

47. Wohlfarth, Ch. & Lechner, M. D. Dielectric constant of N,N-dimethylacetamide: Datasheet from Landolt-Börnstein - Group IV Physical Chemistry · Volume 17: 'Supplement to IV/6' in SpringerMaterials (https://doi.org/10.1007/978-3-540-75506-7_114). doi:10.1007/978-3-540-75506-7_114.

48. Perdew, J. P., Burke, K. & Ernzerhof, M. Generalized gradient approximation made simple. *Phys. Rev. Lett.* **77**, 3865-3868 (1996).

49. Grimme, S., Ehrlich, S. & Goerigk, L. Effect of the damping function in dispersion corrected density functional theory. *J. Comp. Chem.* **32**, 1456-1465 (2011).

50. Hehre, W. J., Ditchfield, R. & Pople, J. A. Self–consistent molecular orbital methods. xii. Further extensions of Gaussian–type basis sets for use in molecular orbital studies of organic molecules. *J. Chem. Phys.* **56**, 2257-2261 (1972).

51. Marenich, A. V., Cramer, C. J. & Truhlar, D. G. Universal solvation model based on solute electron density and on a continuum model of the solvent defined by the bulk dielectric constant and atomic surface tensions. *J Phys Chem B* **113**, 6378- 6396 (2009).

J. NMR spectra

S7 – ^{1}H NMR (400 MHz, $CDCl_3$)

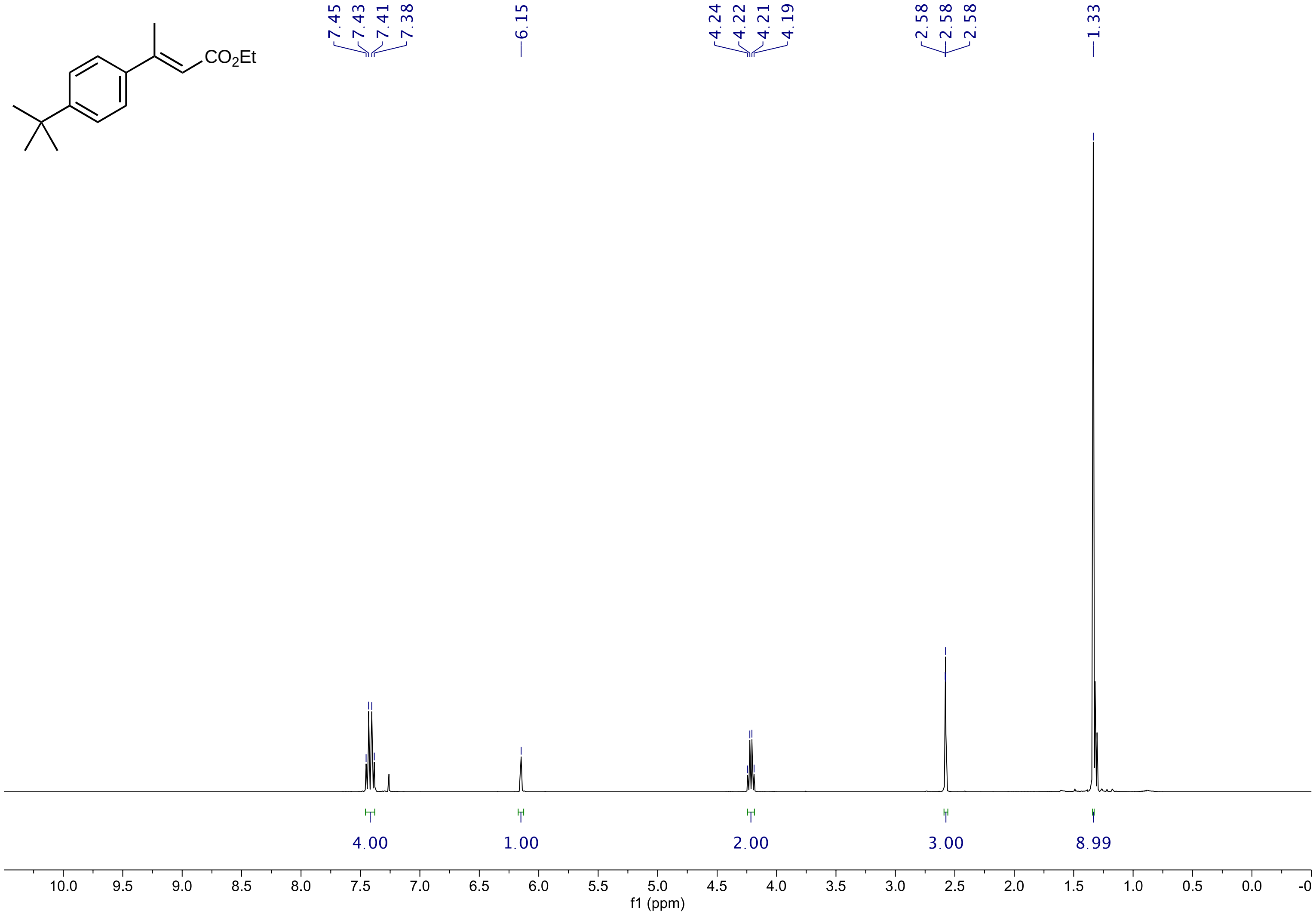

S7 – ^{13}C NMR (126 MHz, $CDCl_3$)

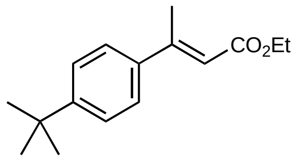

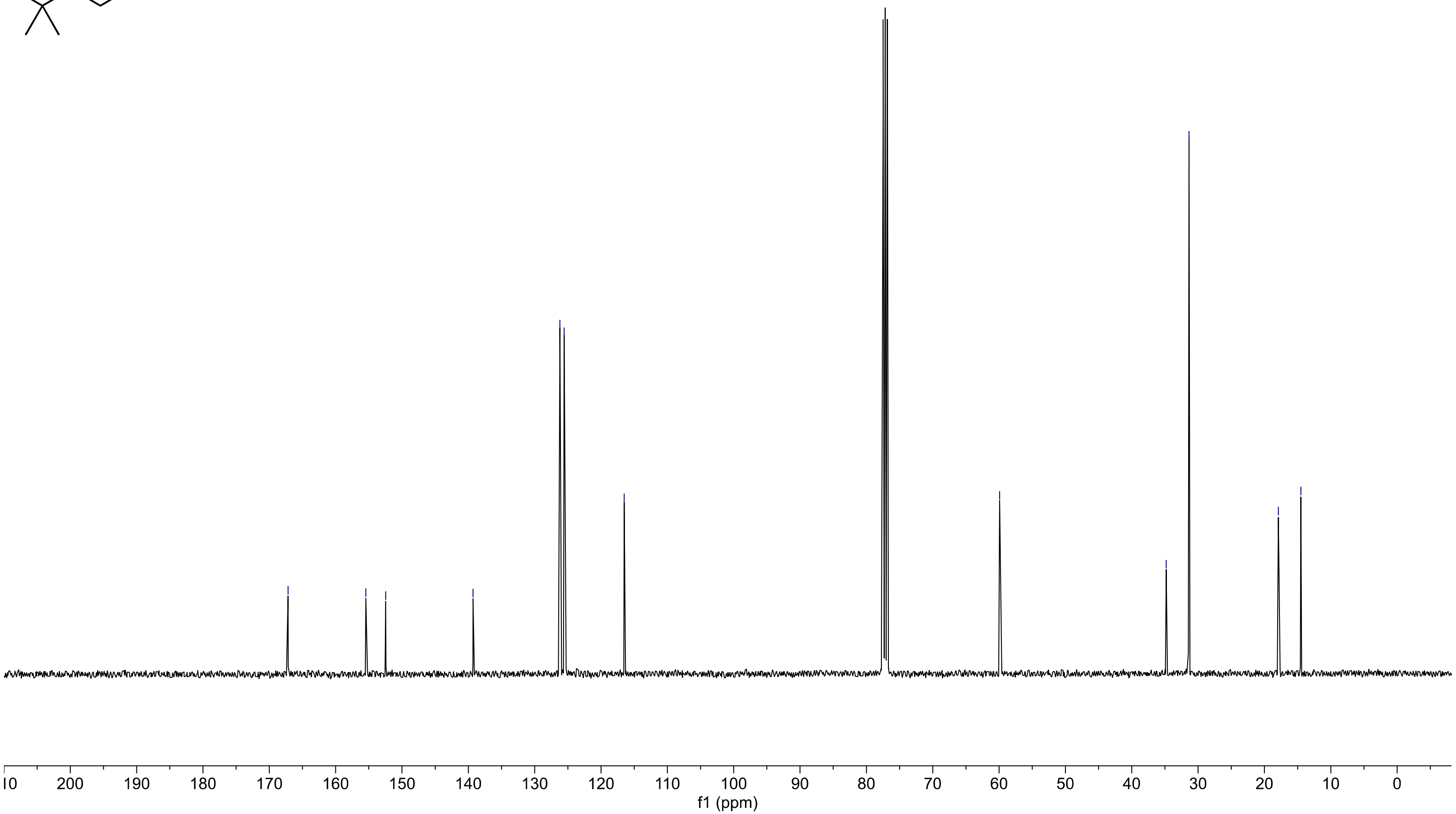

S11 – ^{1}H NMR (400 MHz, $CDCl_3$)

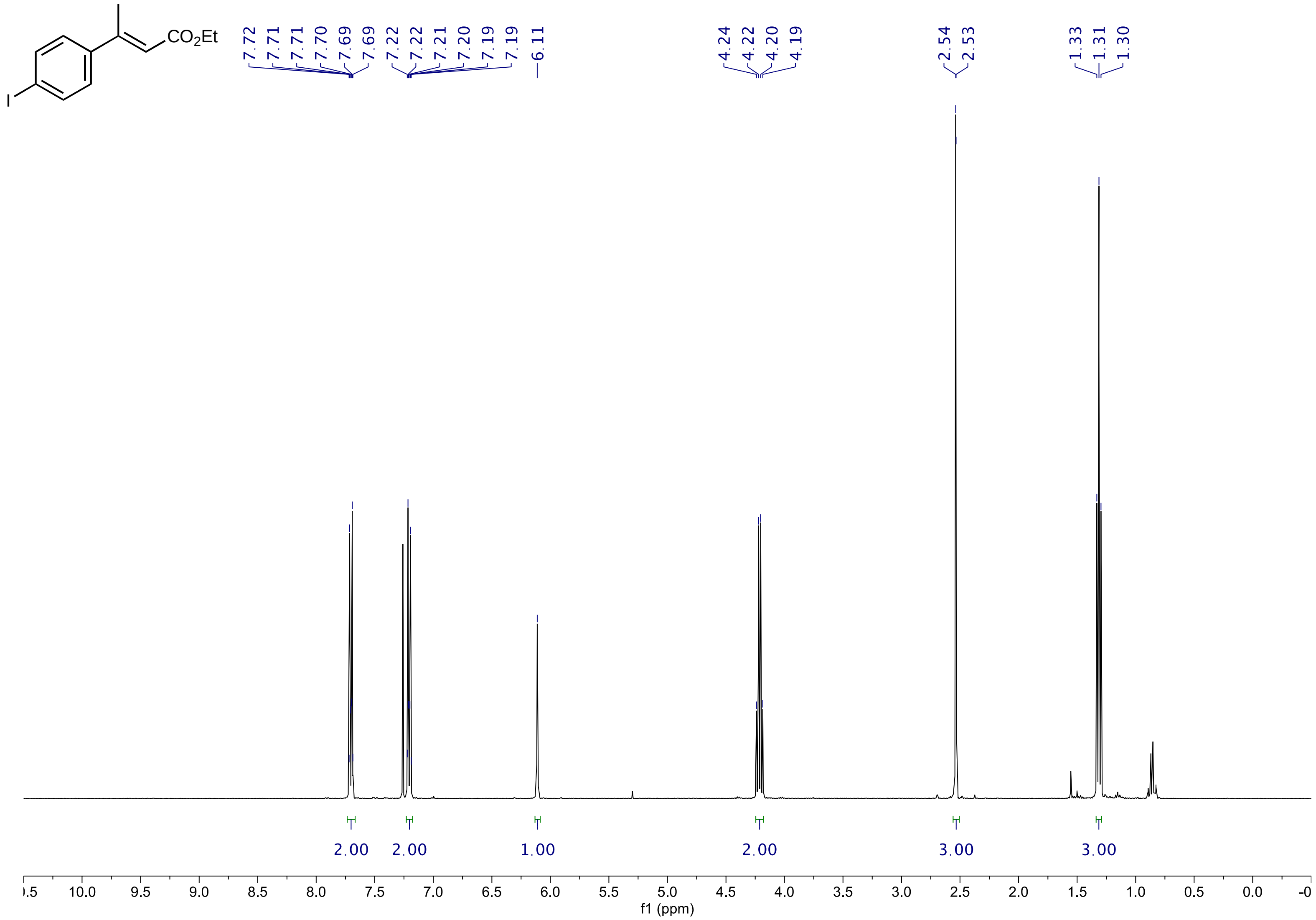

S11 – ^{13}C NMR (126 MHz, $CDCl_3$)

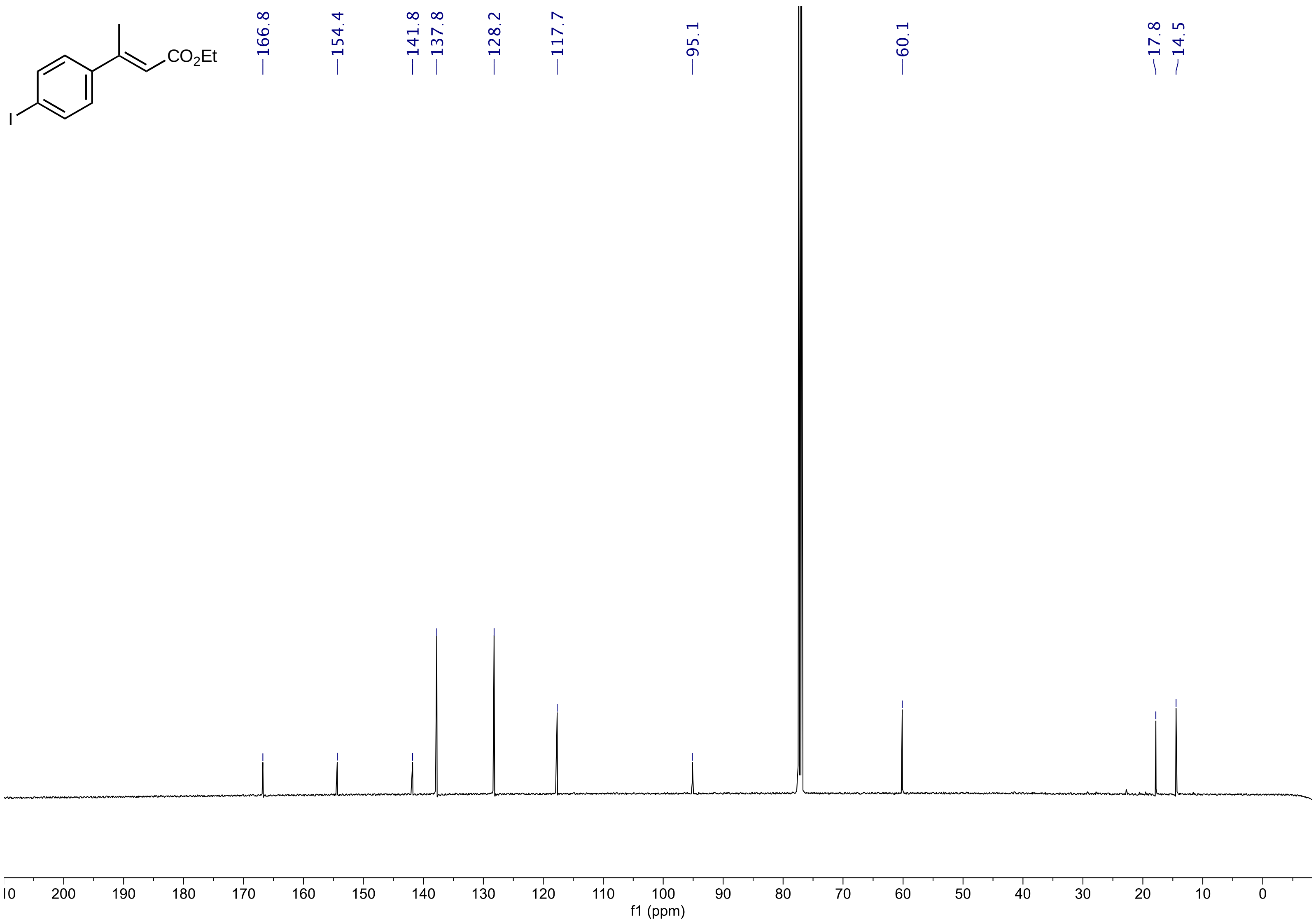

S12 – ^{1}H NMR (400 MHz, $CDCl_3$)

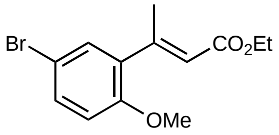

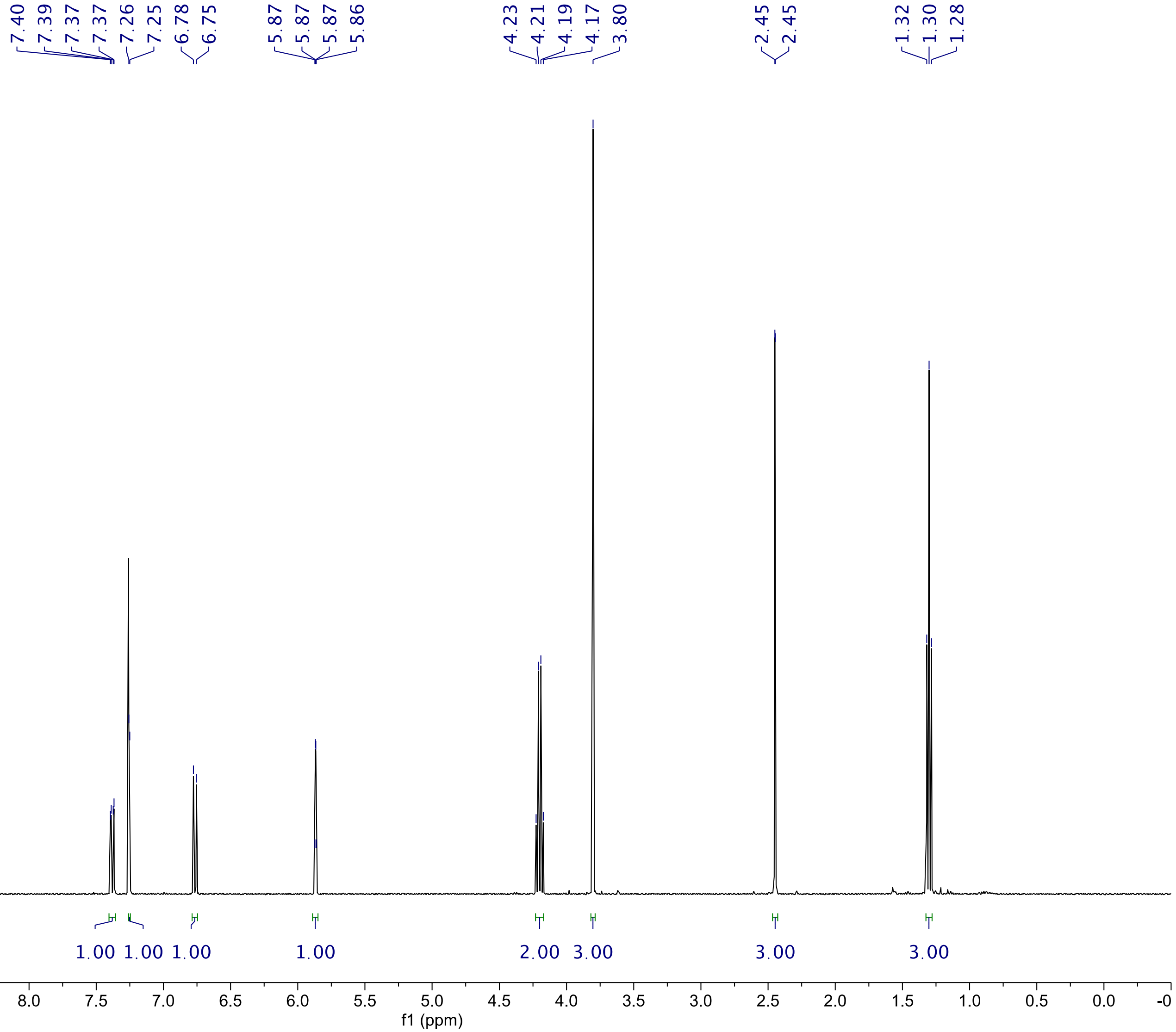

S12 – ^{13}C NMR (126 MHz, $CDCl_3$)

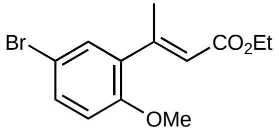

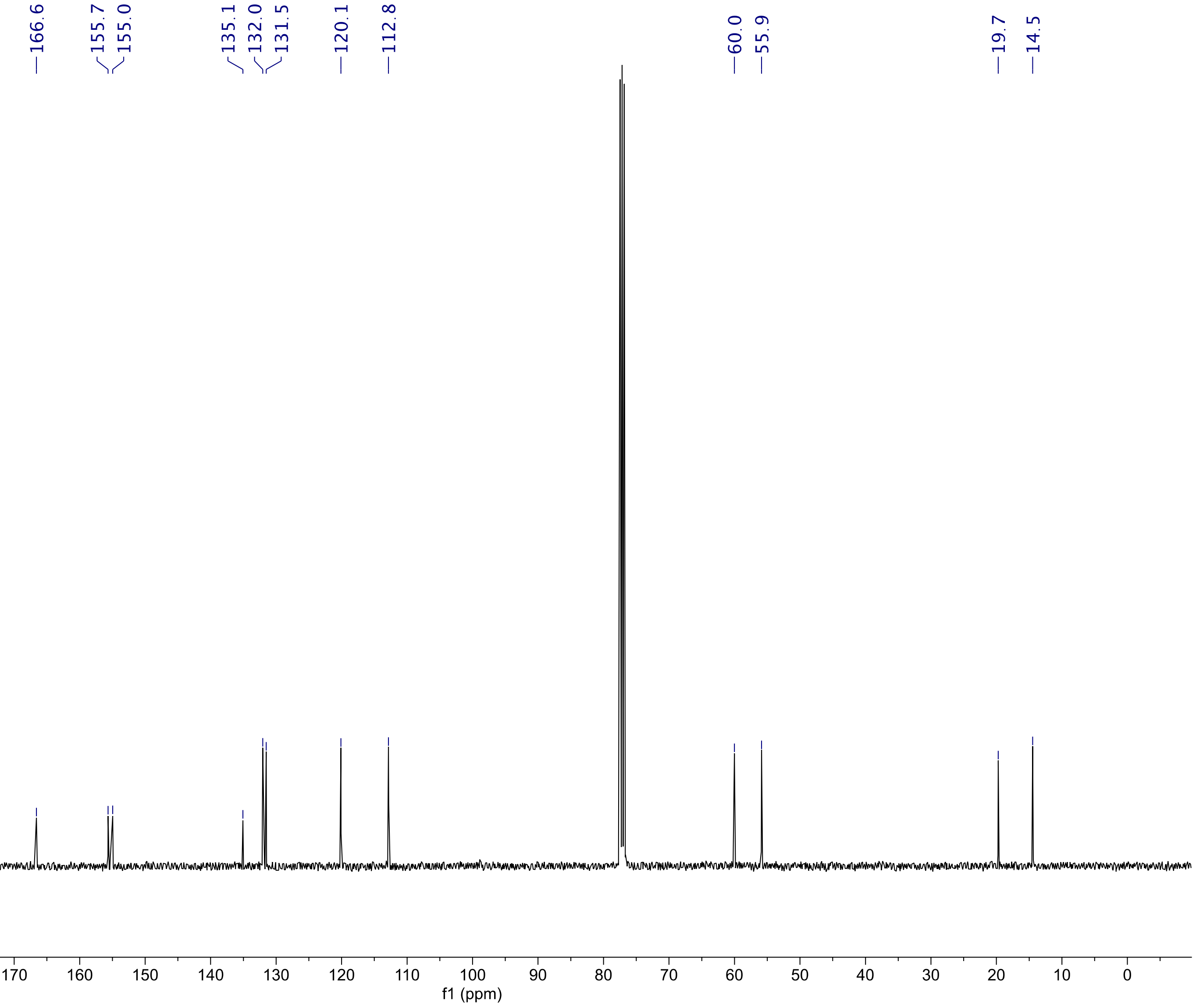

S13 – ^{1}H NMR (400 MHz, $CDCl_3$)

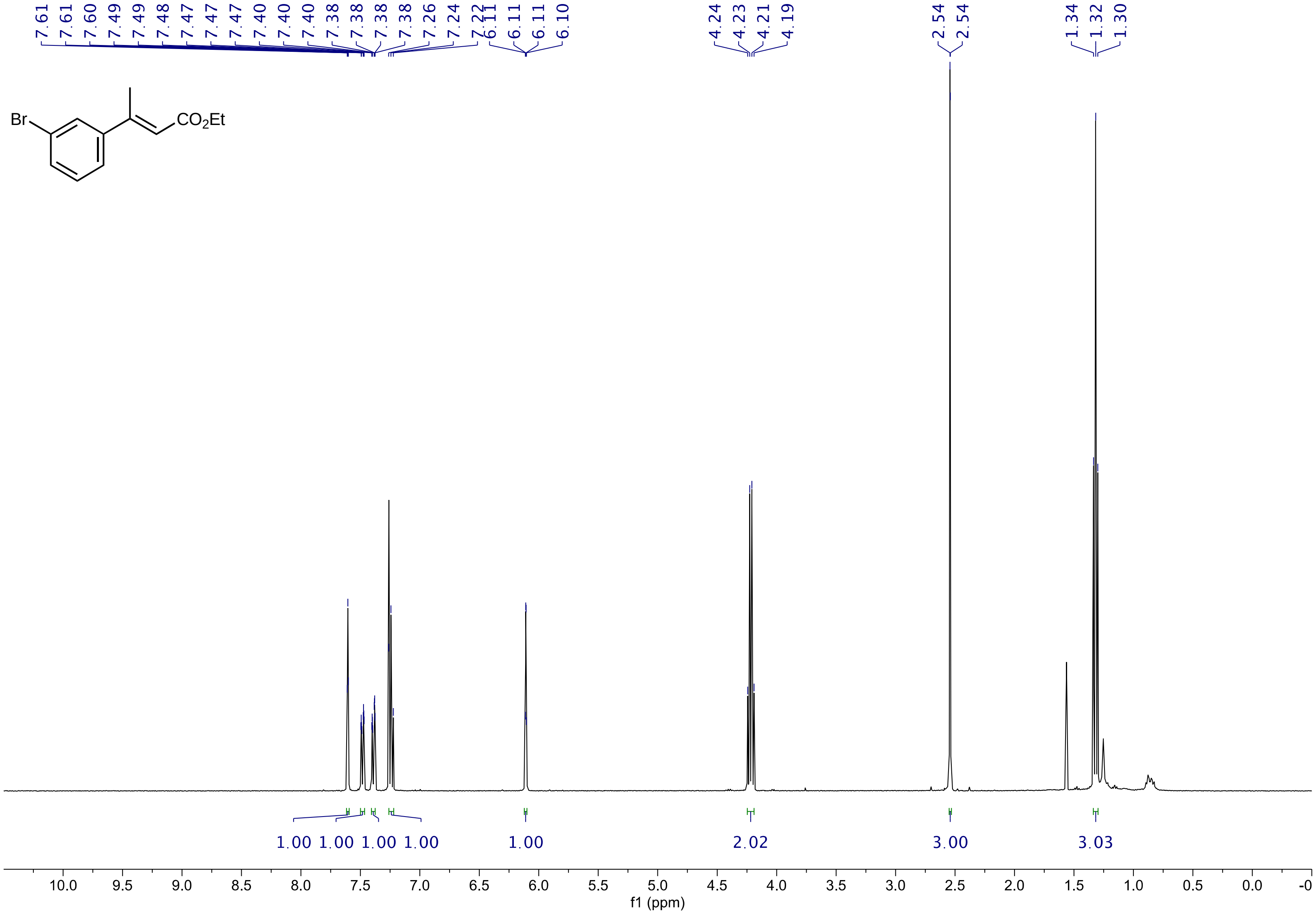

S13 – ^{13}C NMR (126 MHz, $CDCl_3$)

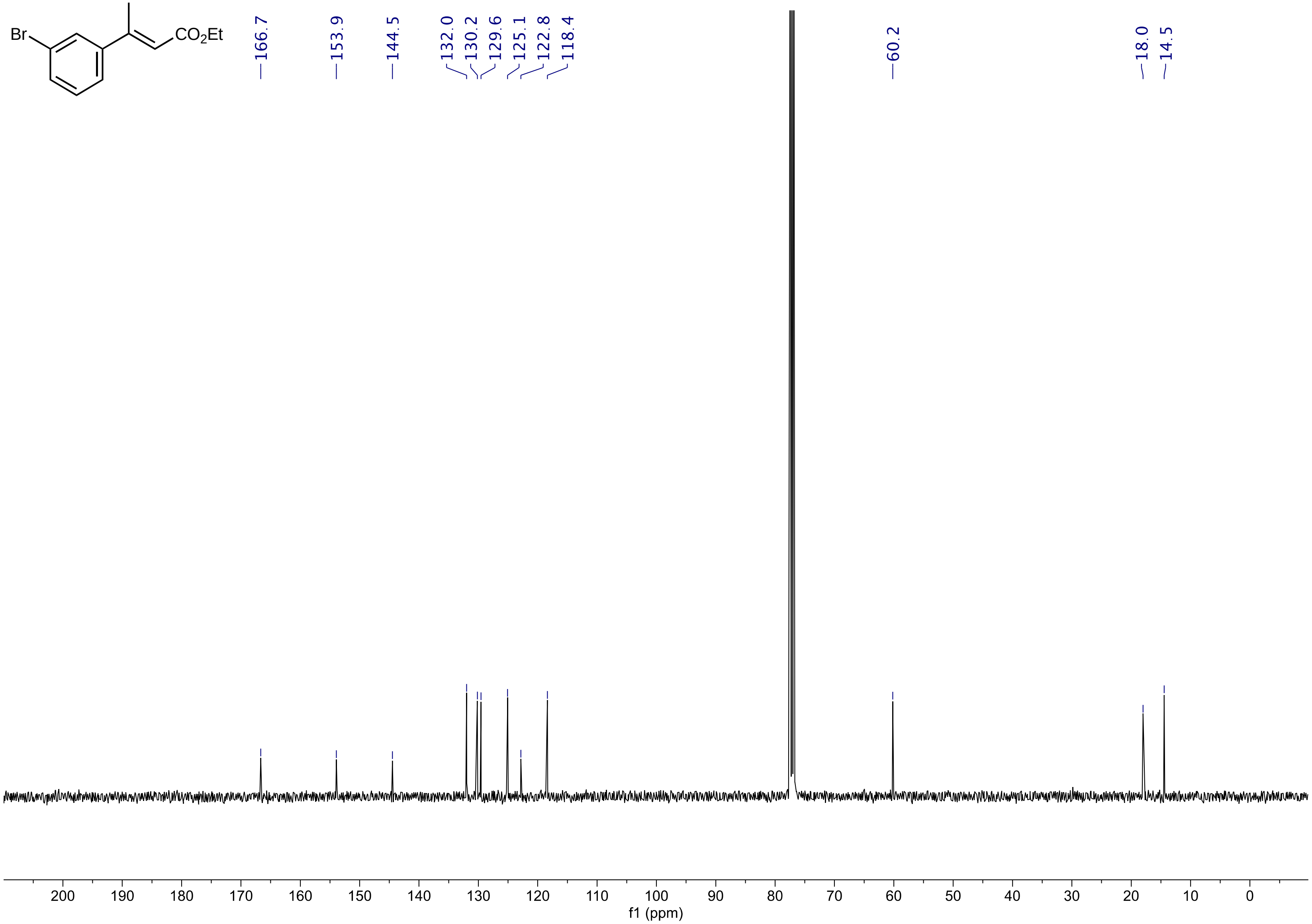

S24 – ^{1}H NMR (400 MHz, $CDCl_3$)

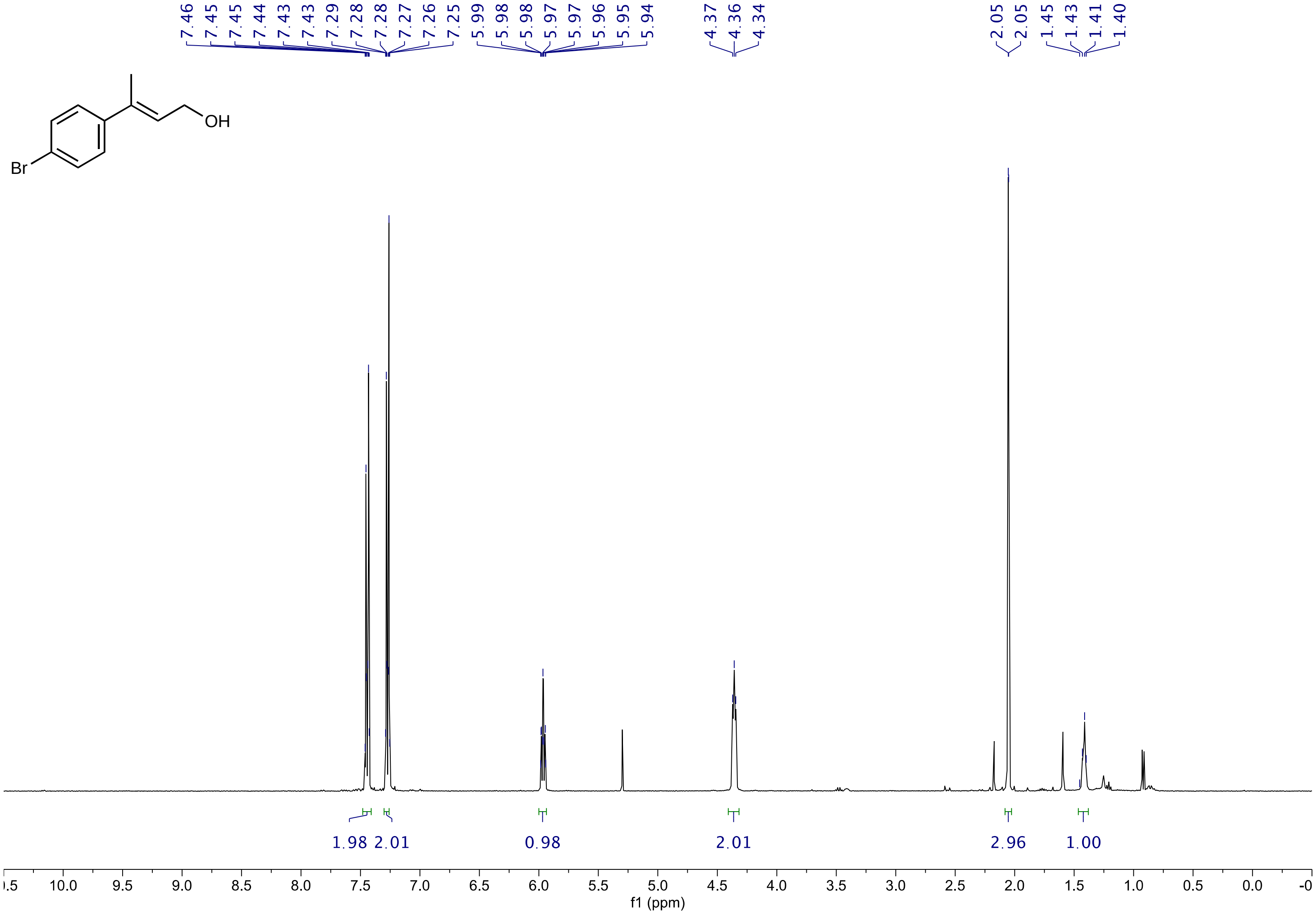

S24 – ^{13}C NMR (126 MHz, $CDCl_3$)

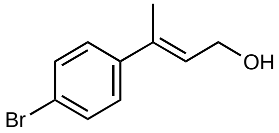

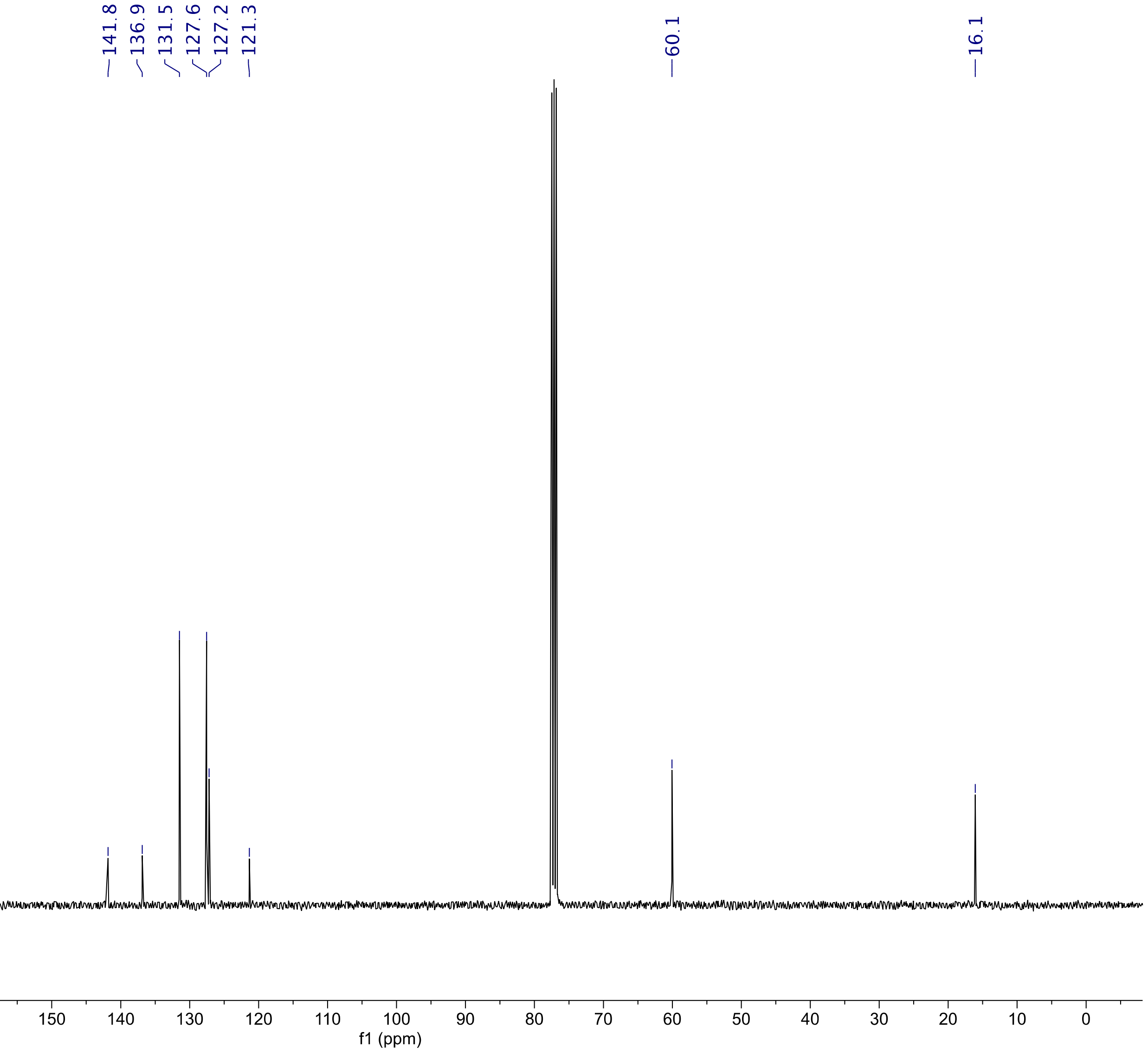

S25 – ^{1}H NMR (400 MHz, $CDCl_3$)

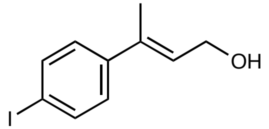

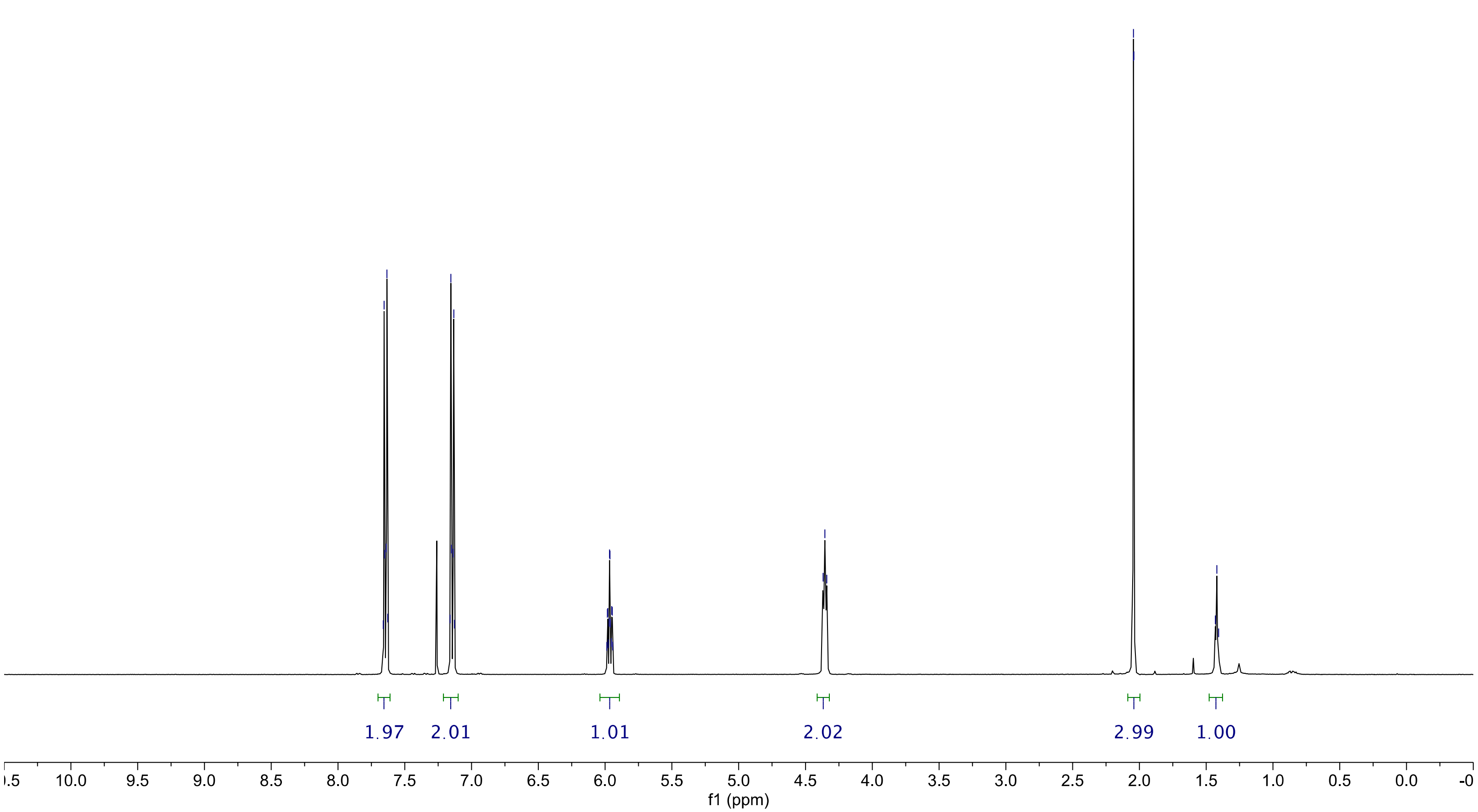

S25 – ^{13}C NMR (126 MHz, $CDCl_3$)

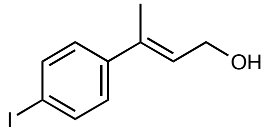

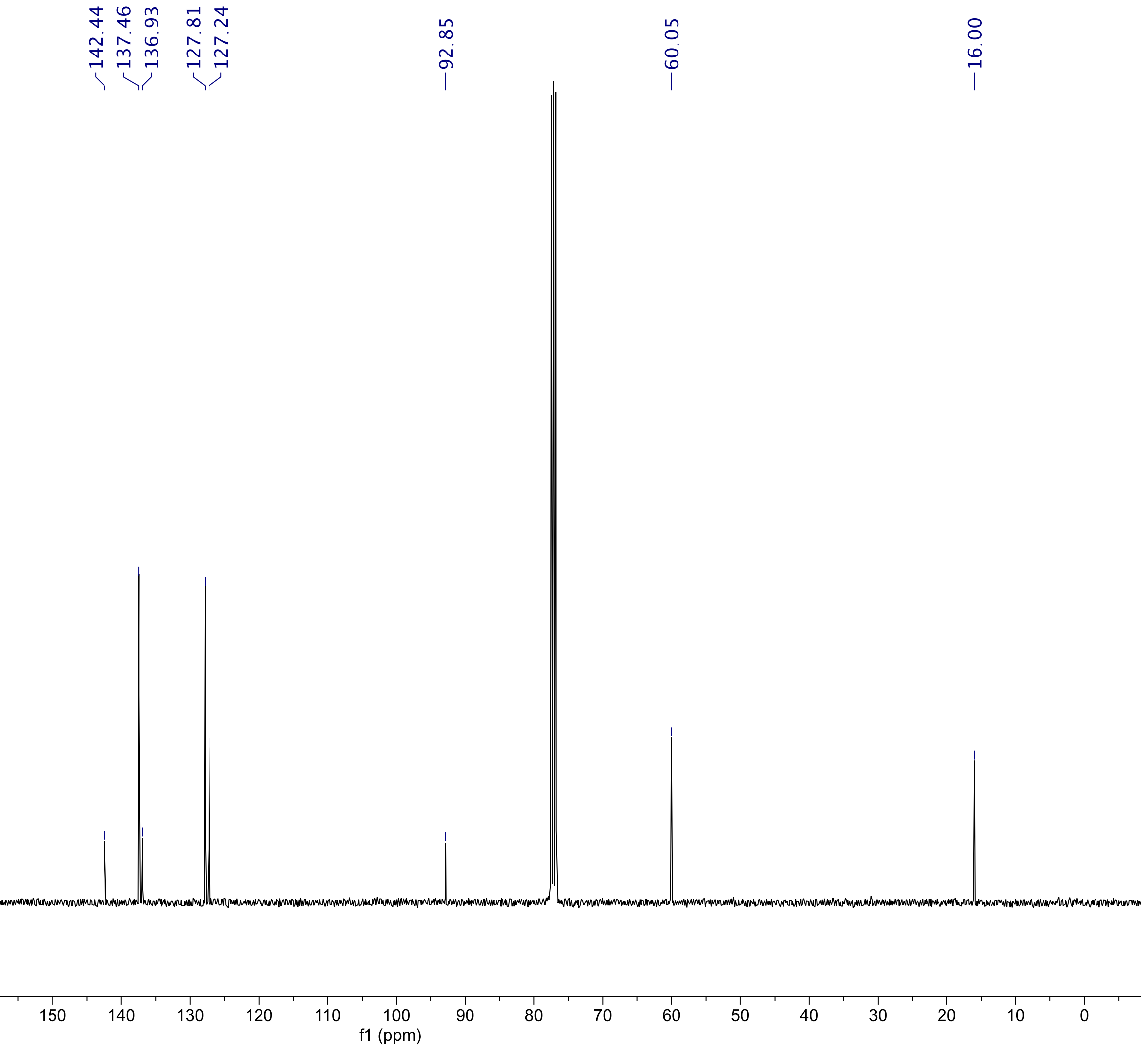

S26 – ^{1}H NMR (400 MHz, $CDCl_3$)

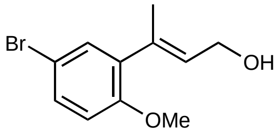

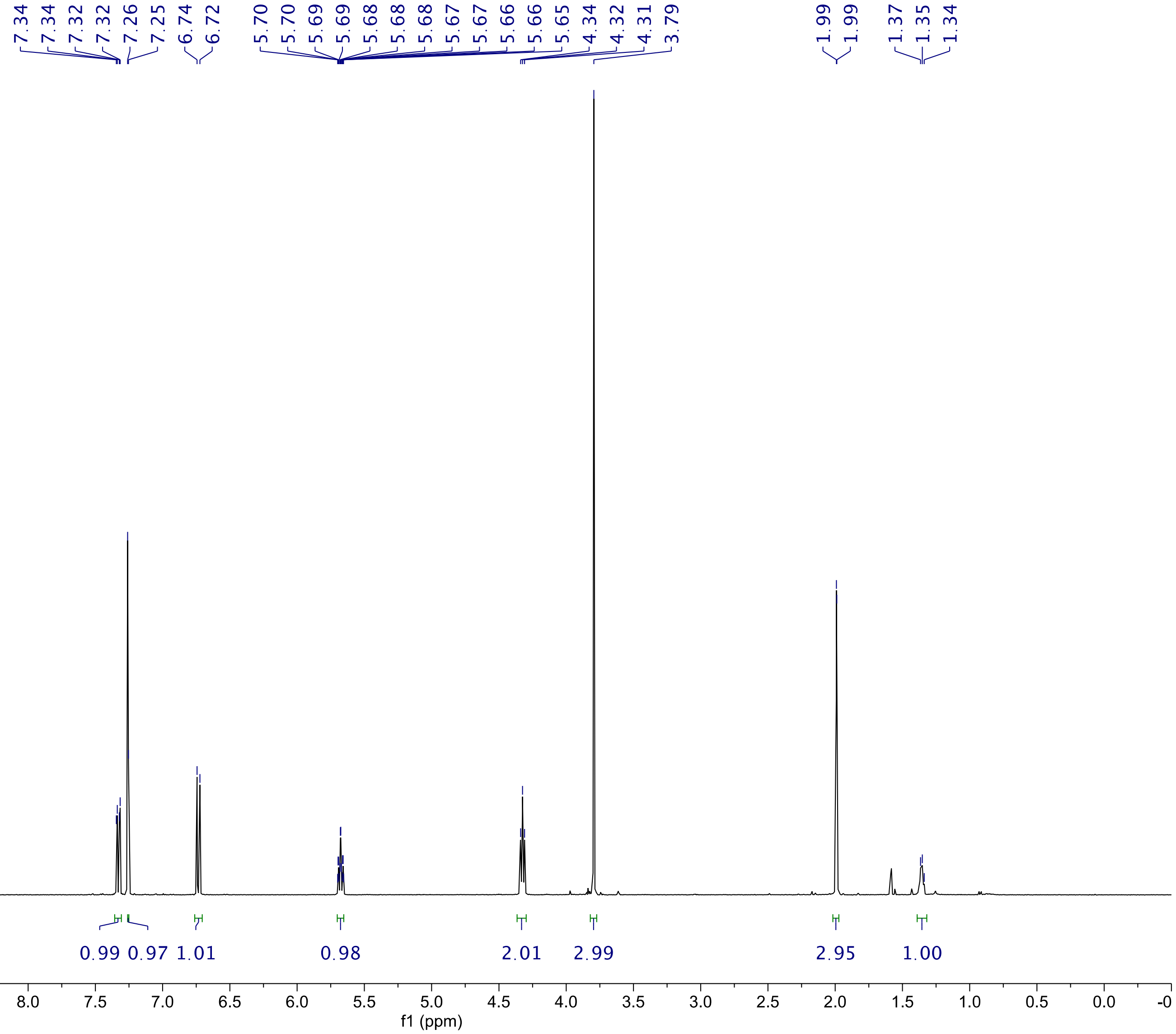

S26 – ^{13}C NMR (126 MHz, $CDCl_3$)

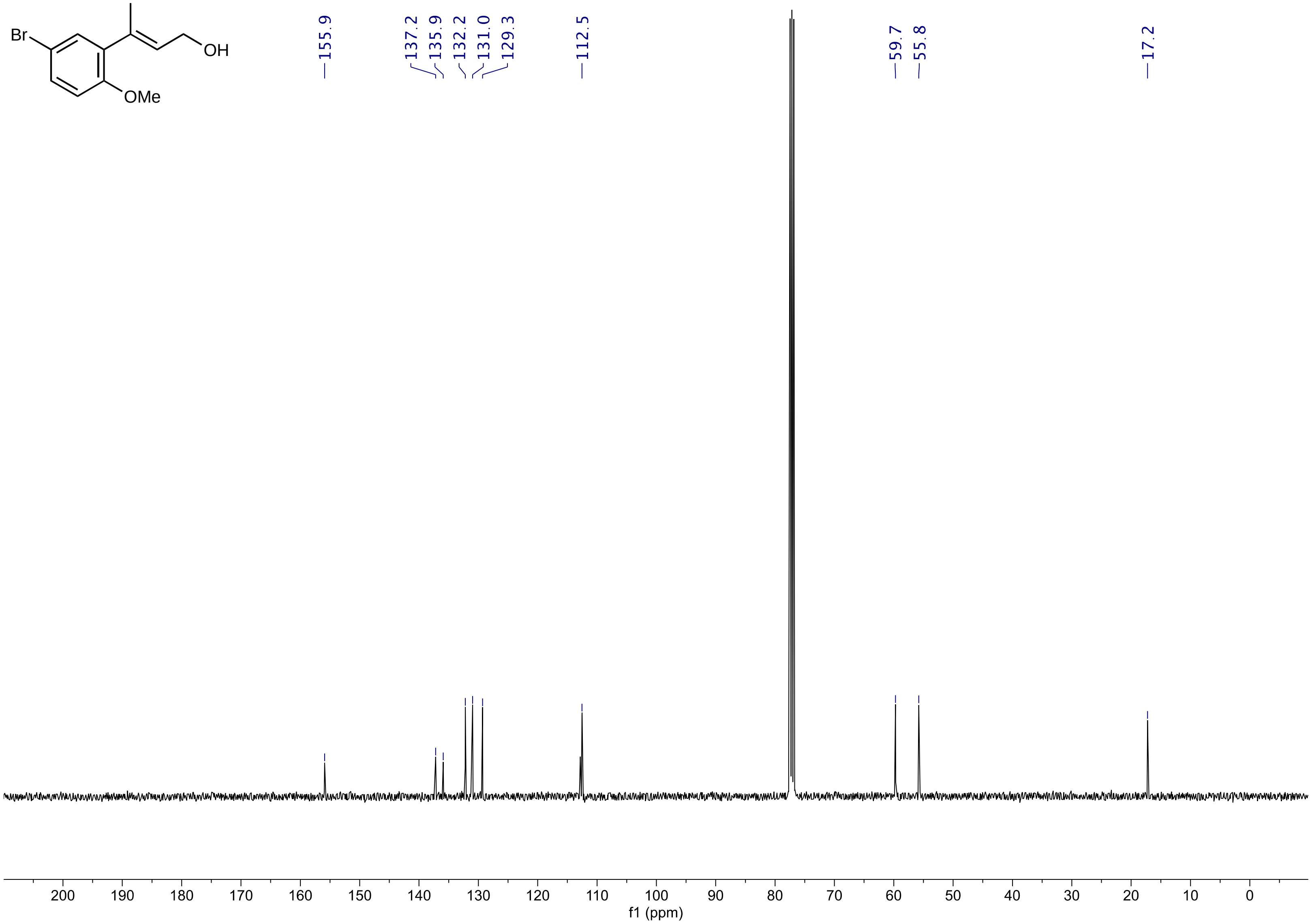

S27 – ^{1}H NMR (400 MHz, $CDCl_3$)

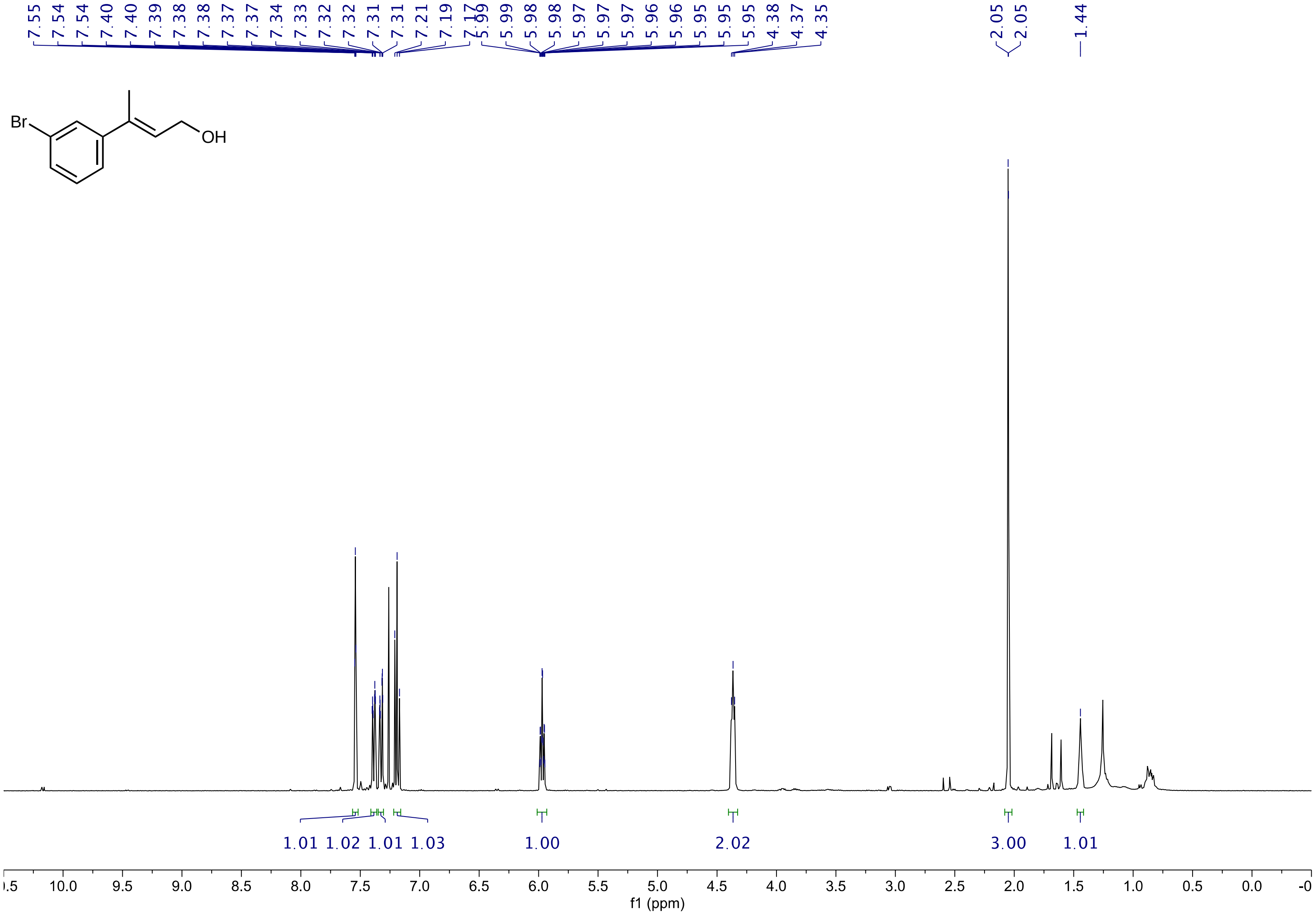

S27 – ^{13}C NMR (126 MHz, $CDCl_3$)

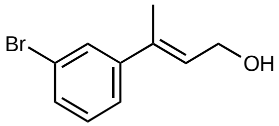

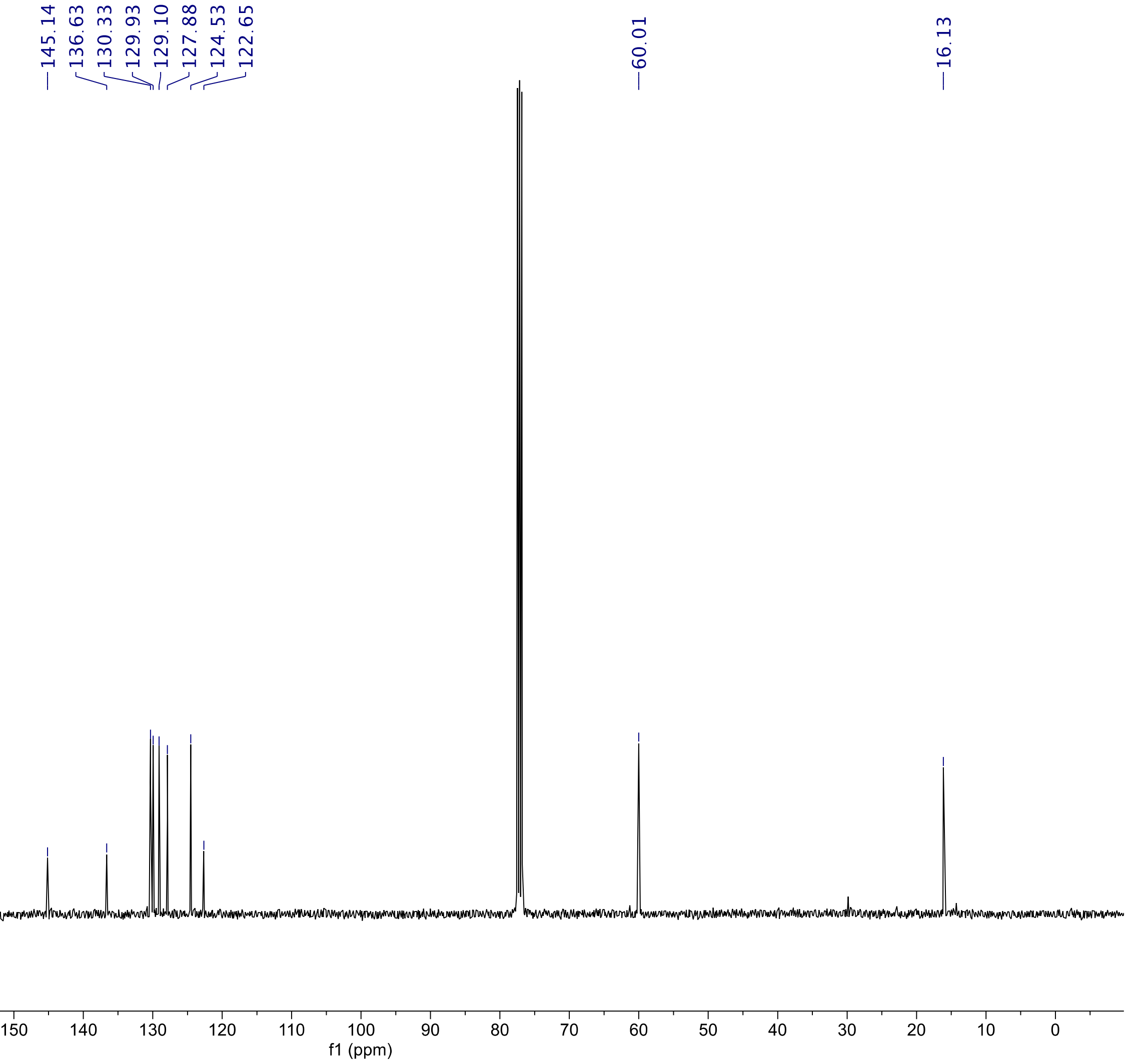

S43 – ^{1}H NMR (400 MHz, $CDCl_3$)

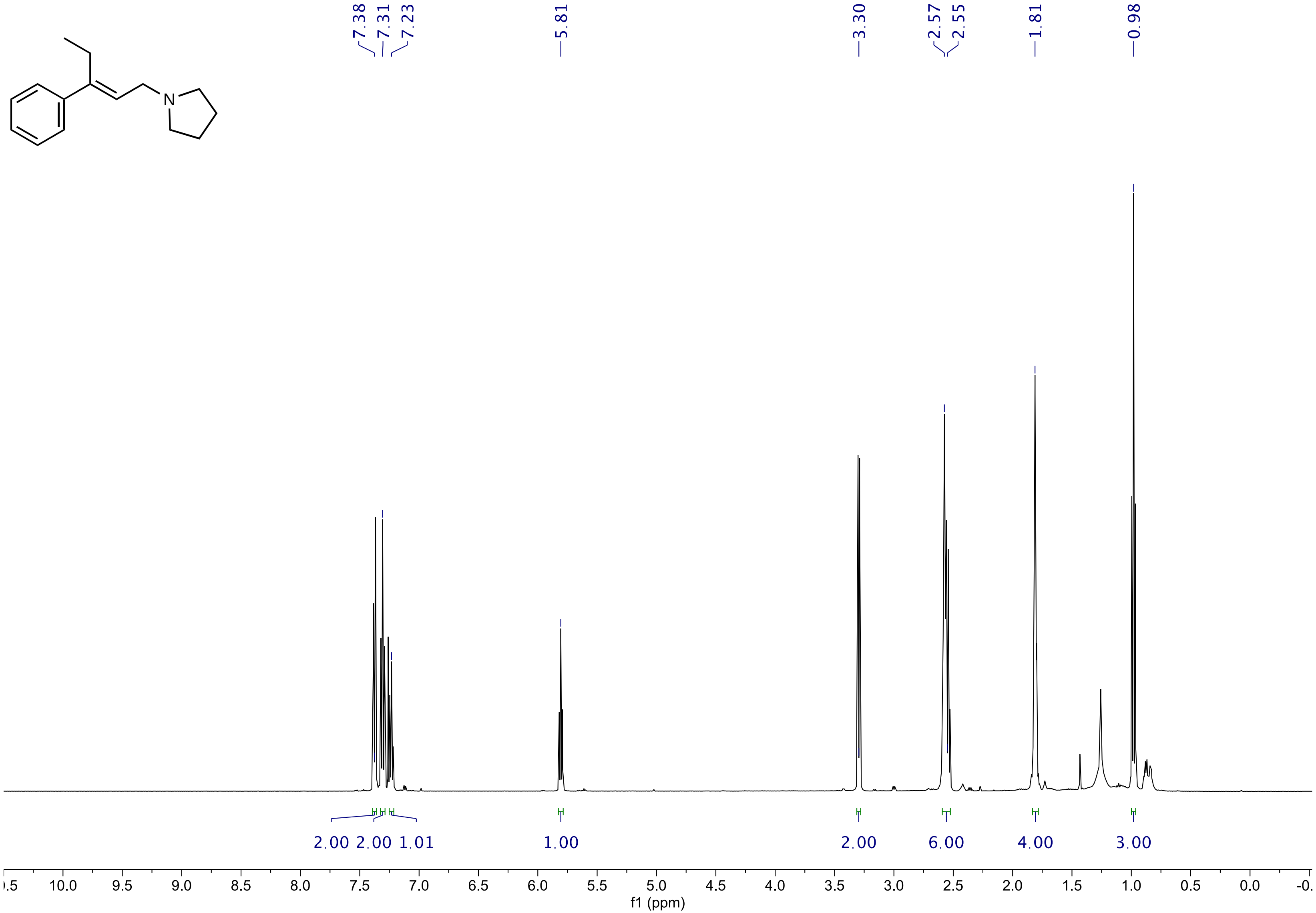

S43 – ^{13}C NMR (126 MHz, $CDCl_3$)

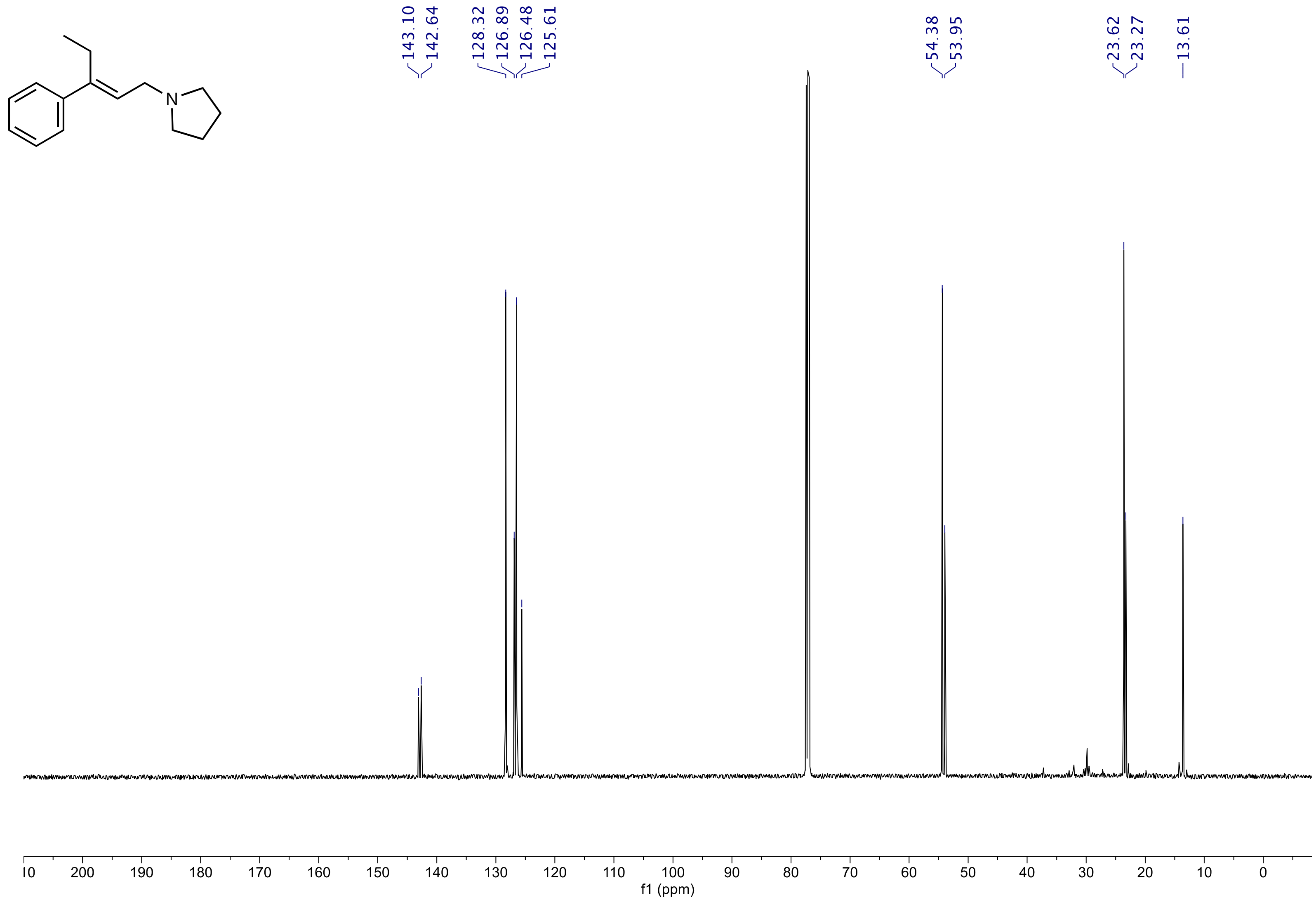

S44 – ^{1}H NMR (400 MHz, $CDCl_3$)

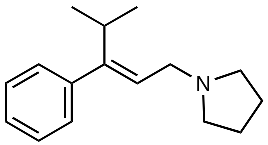

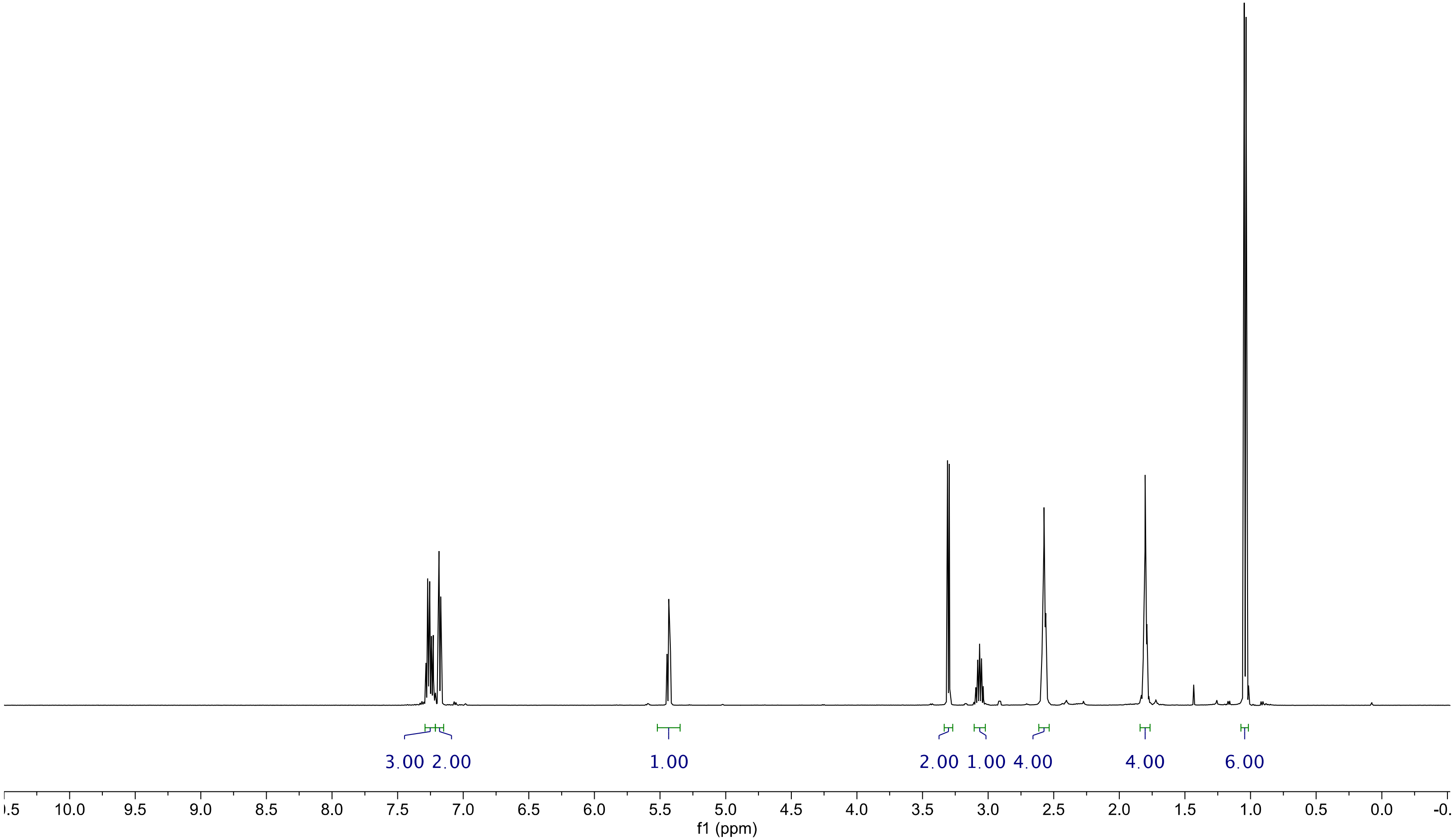

S44 – ^{13}C NMR (126 MHz, $CDCl_3$)

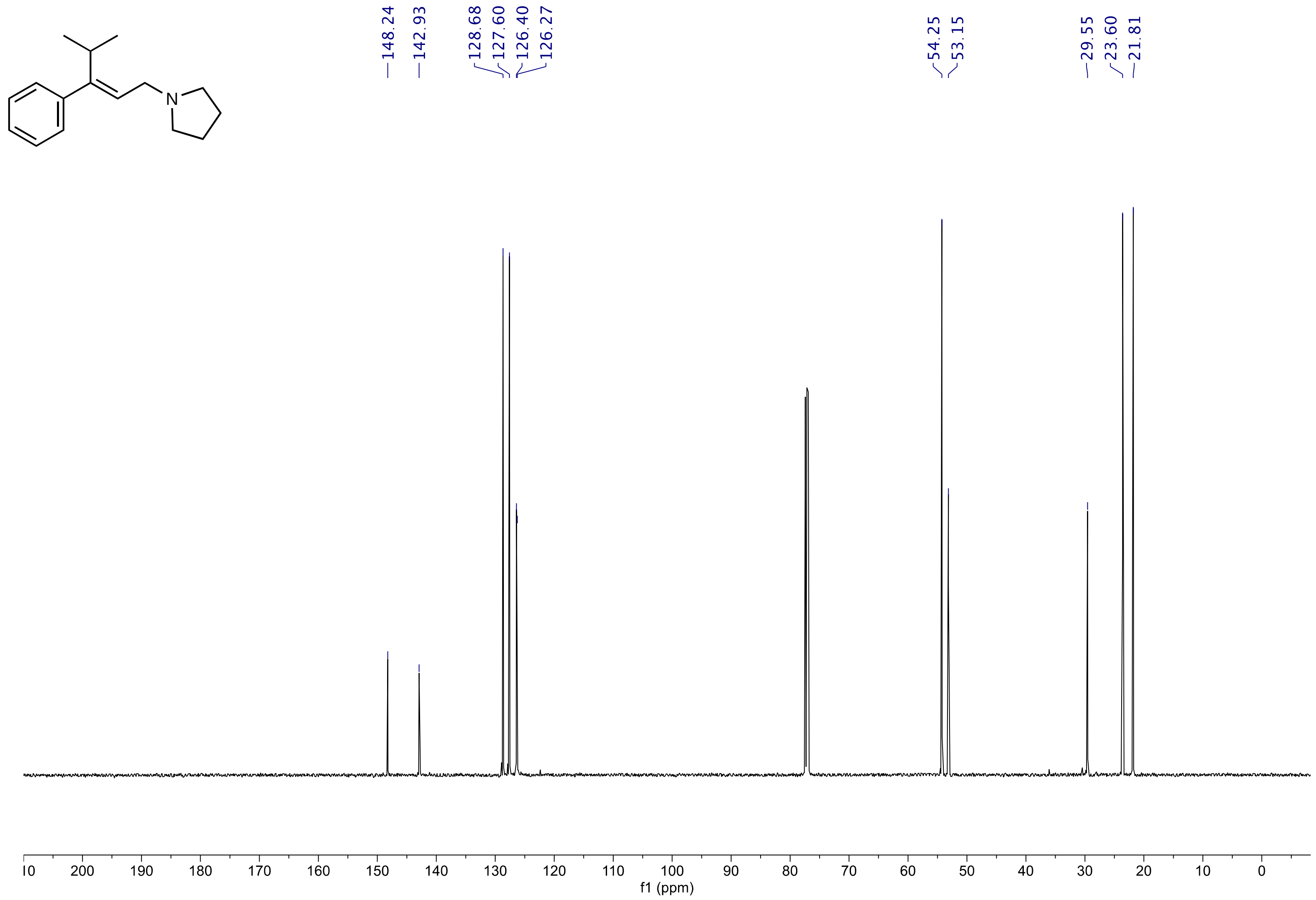

S47 – ^{1}H NMR (400 MHz, $CDCl_3$)

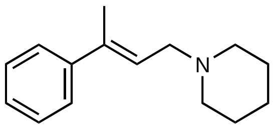

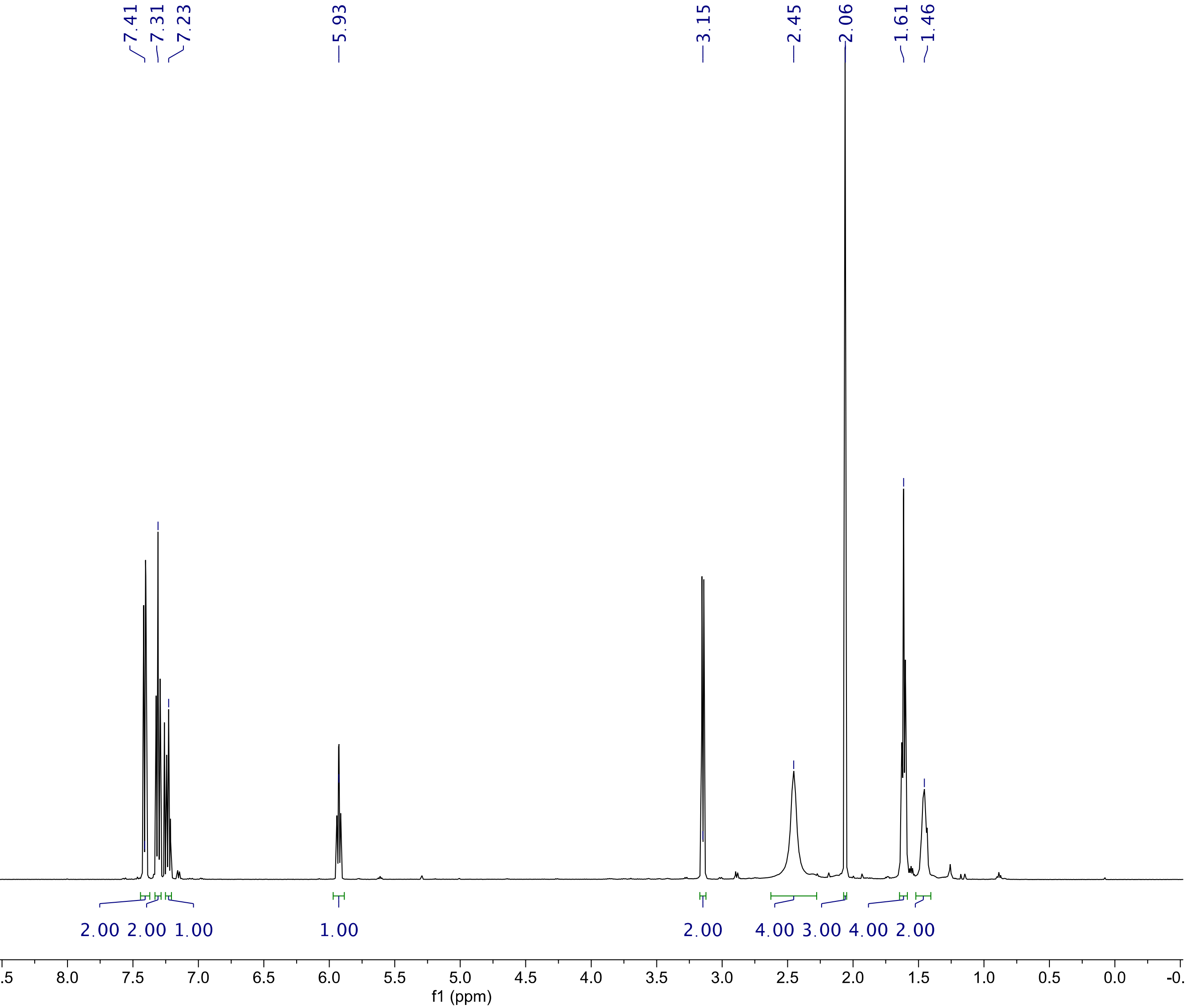

S47 – ^{13}C NMR (126 MHz, $CDCl_3$)

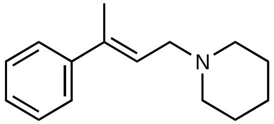

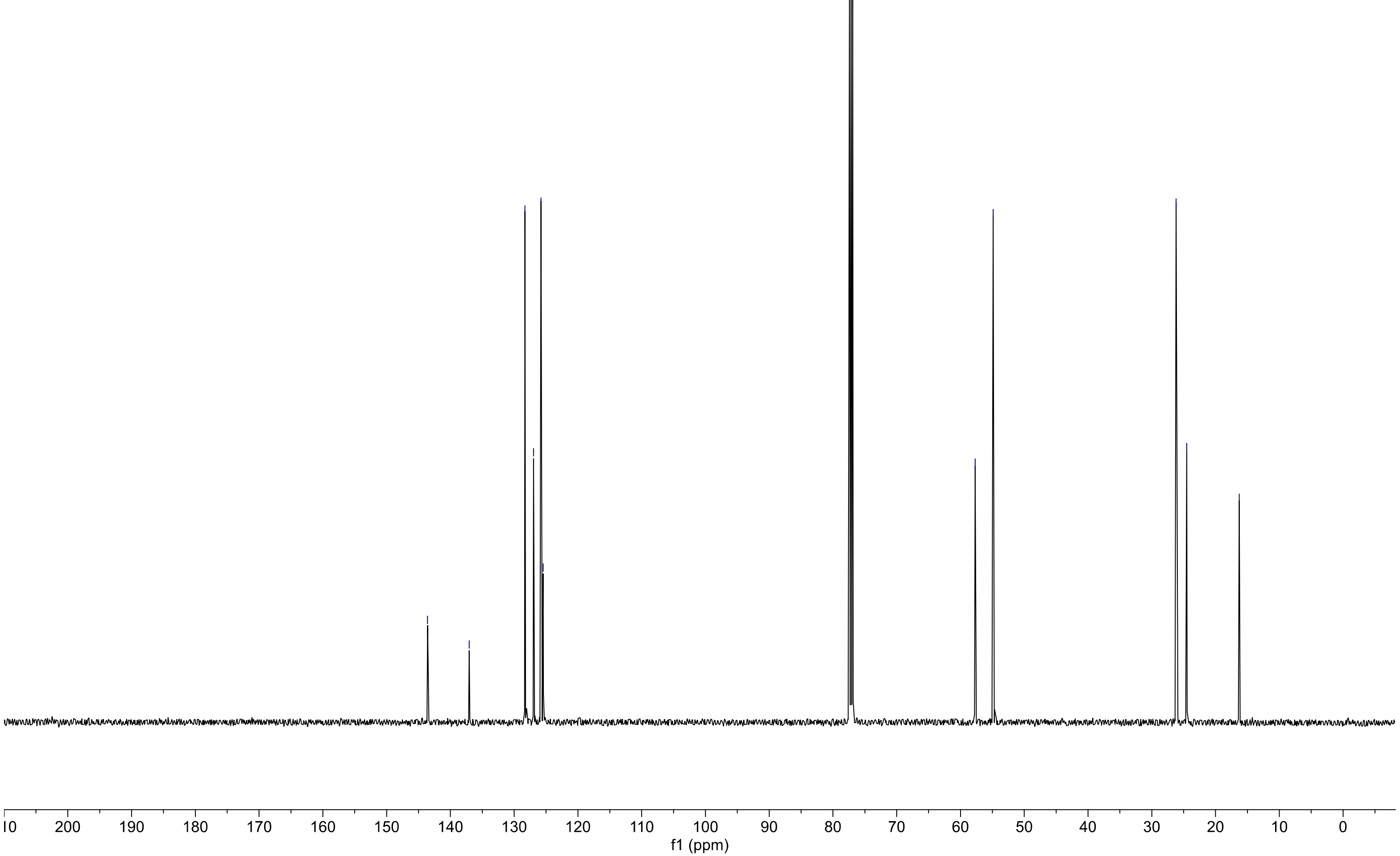

S51 – ^{1}H NMR (400 MHz, $CDCl_3$)

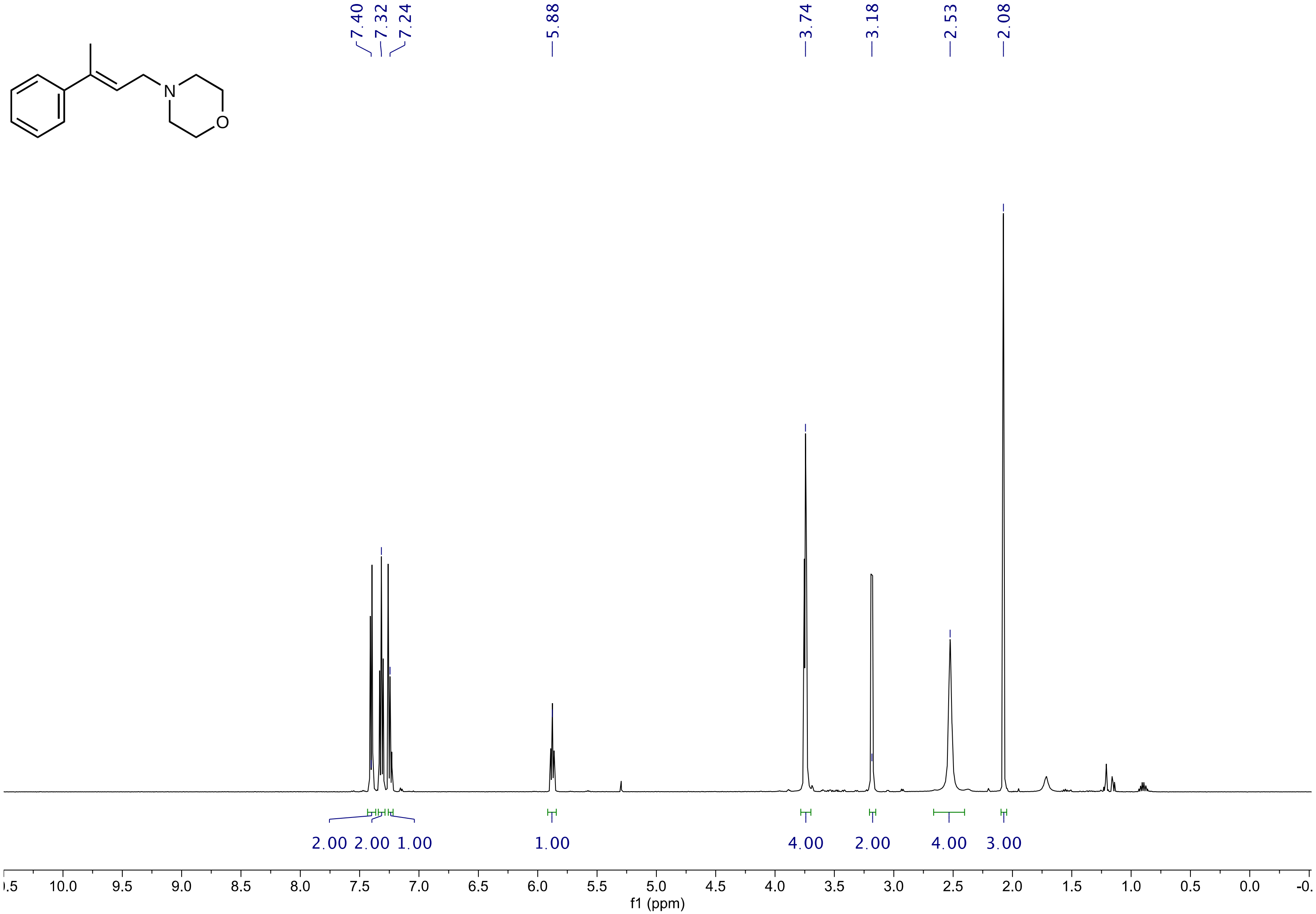

S51 – ^{13}C NMR (126 MHz, $CDCl_3$)

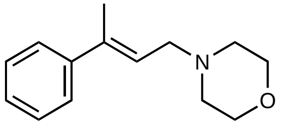

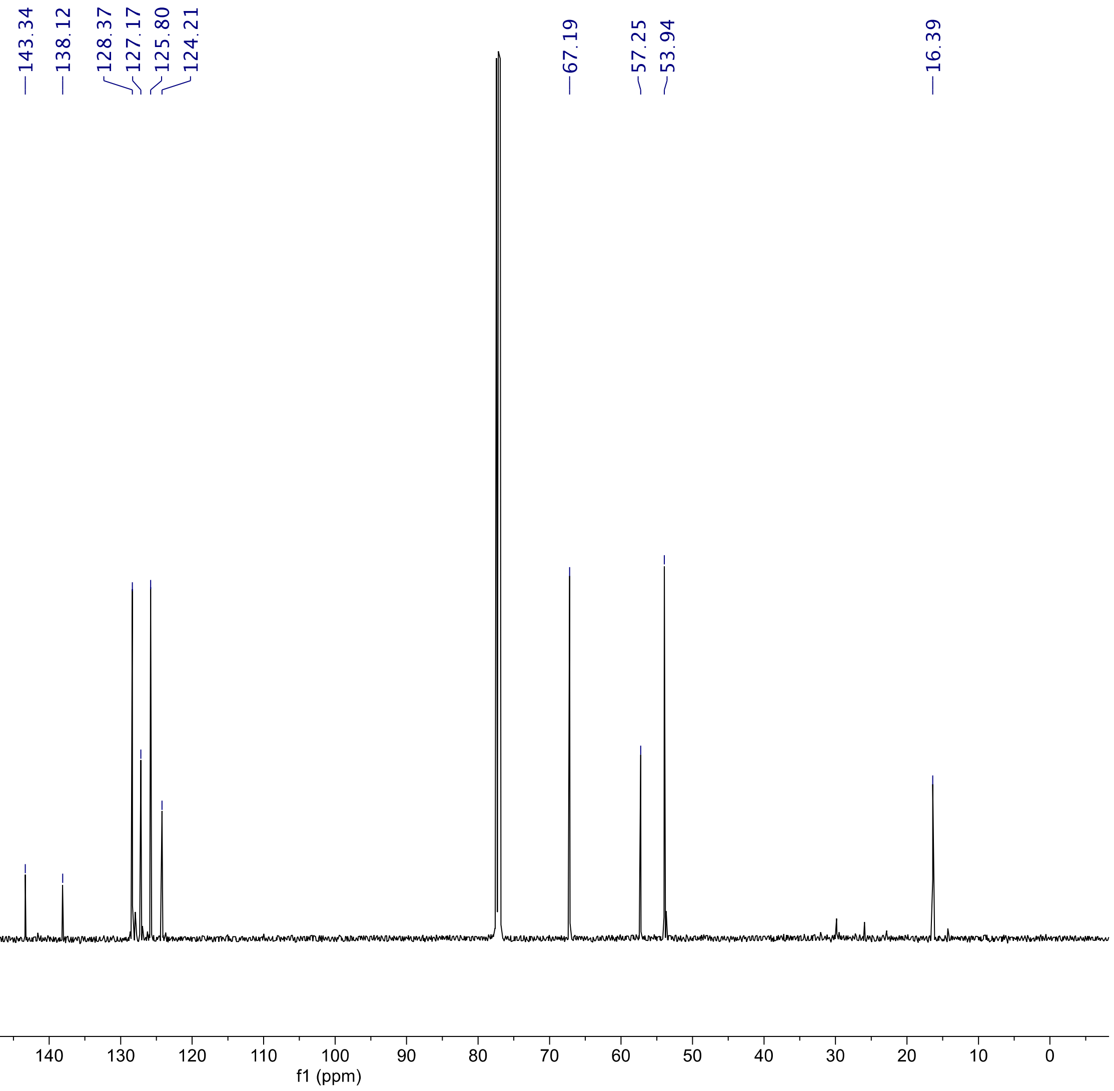

S53 – ^{1}H NMR (400 MHz, $CDCl_3$)

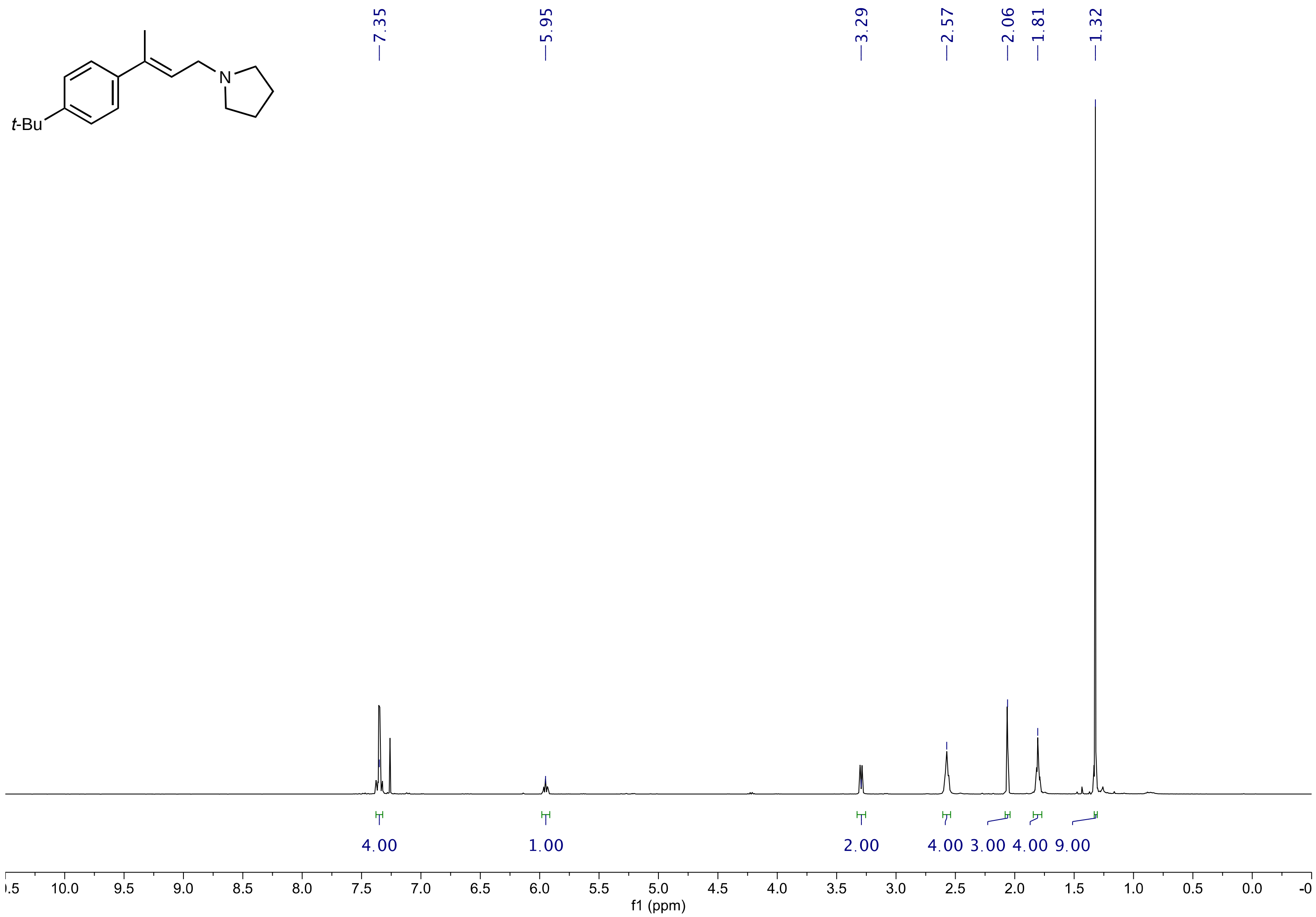

S53 – ^{13}C NMR (126 MHz, $CDCl_3$)

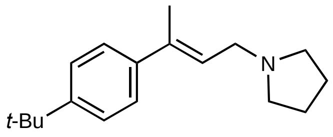

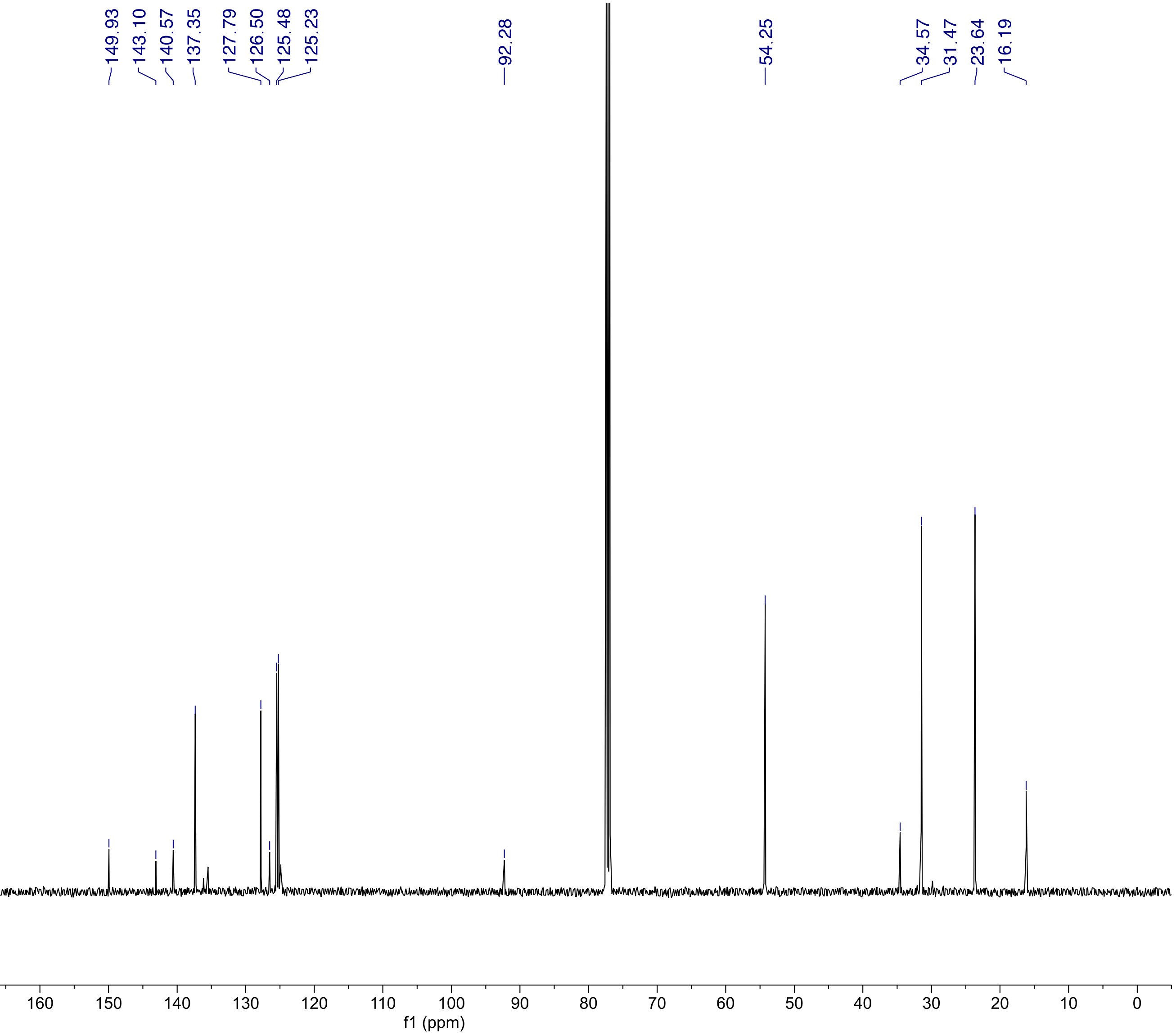

S54 – ^{1}H NMR (400 MHz, $CDCl_3$)

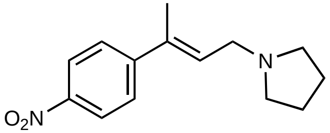

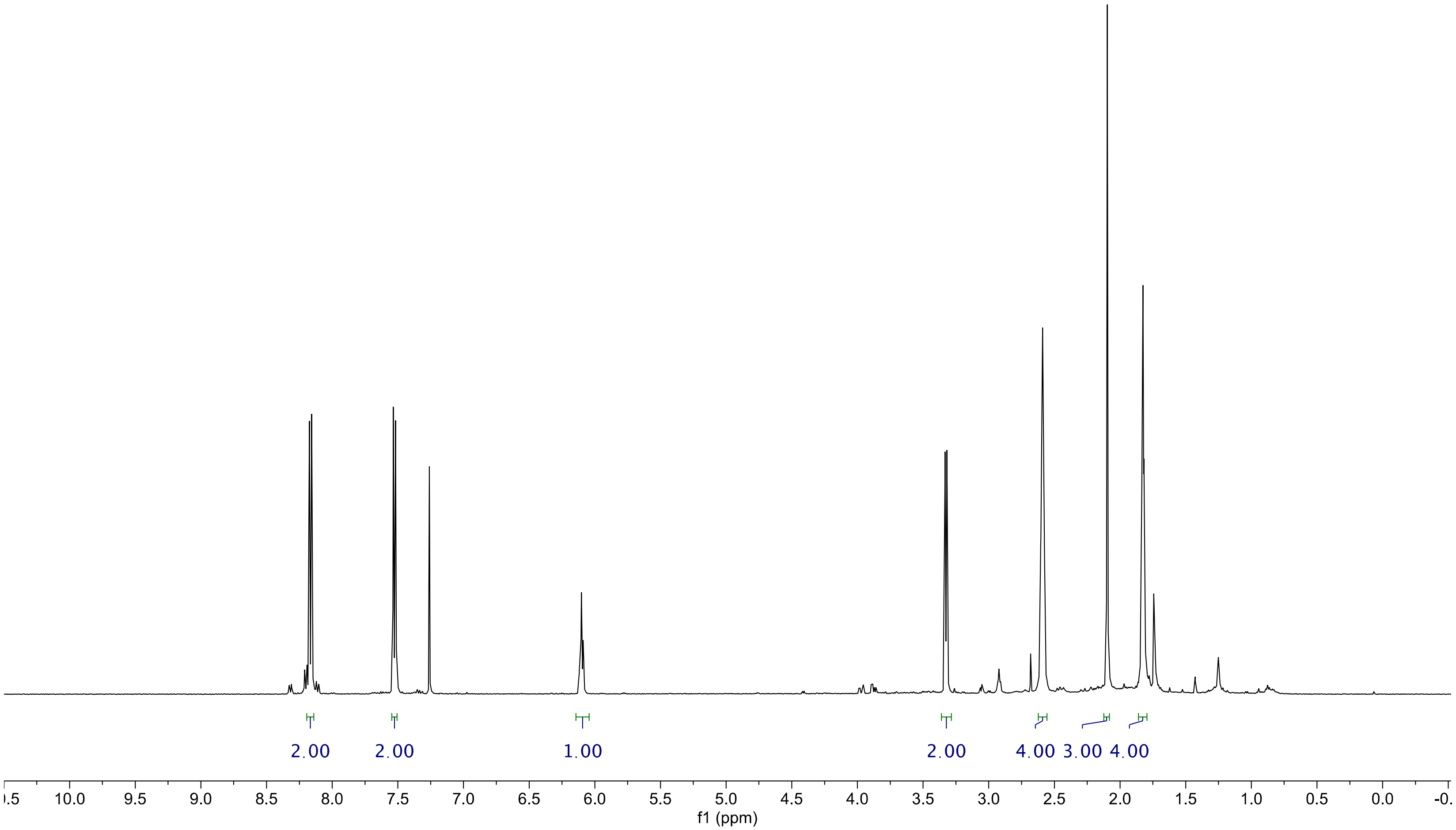

S54 – ^{13}C NMR (126 MHz, $CDCl_3$)

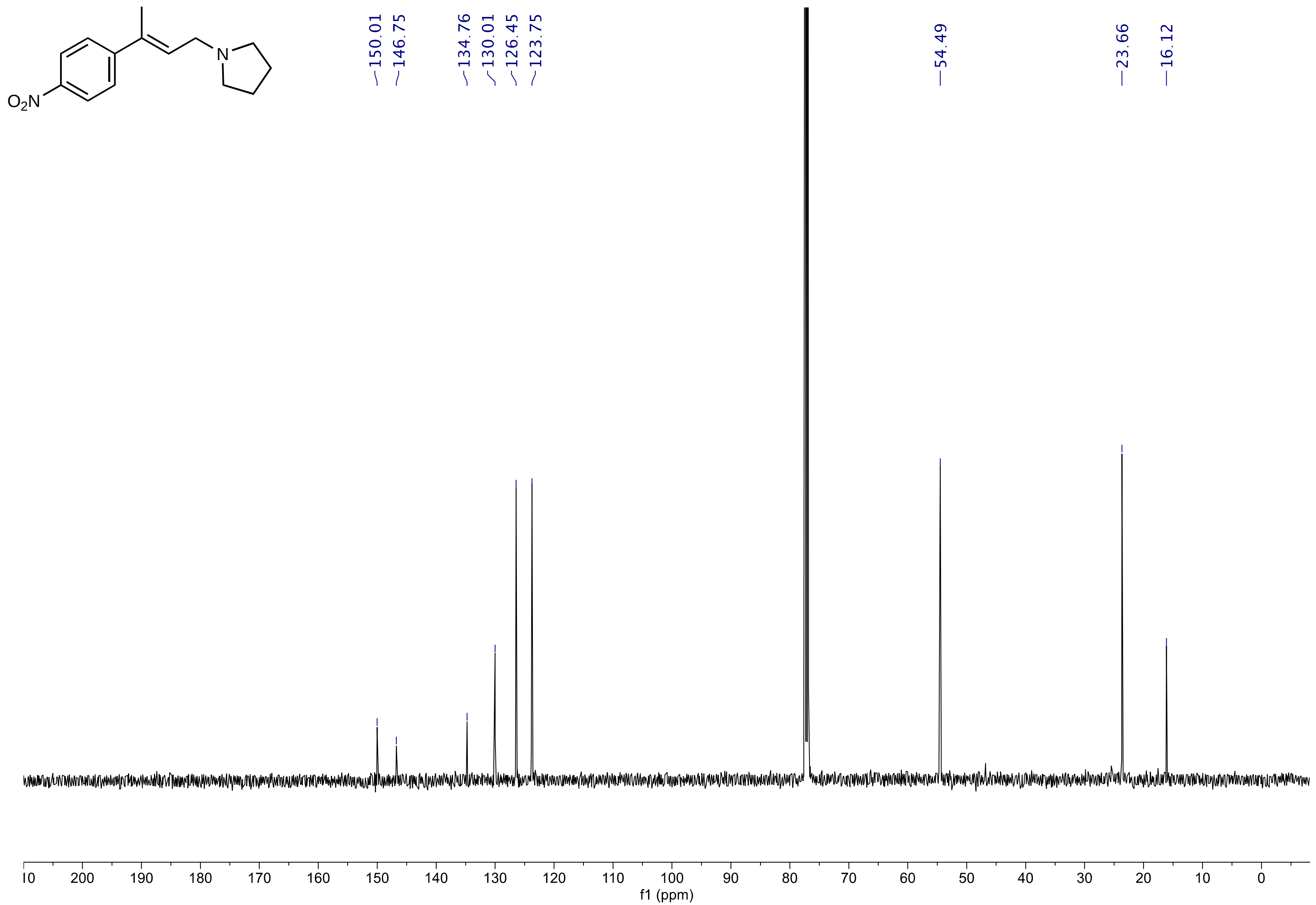

S56 – ^{1}H NMR (400 MHz, $CDCl_3$)

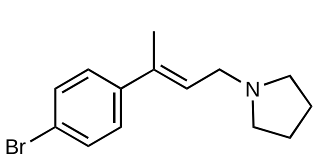

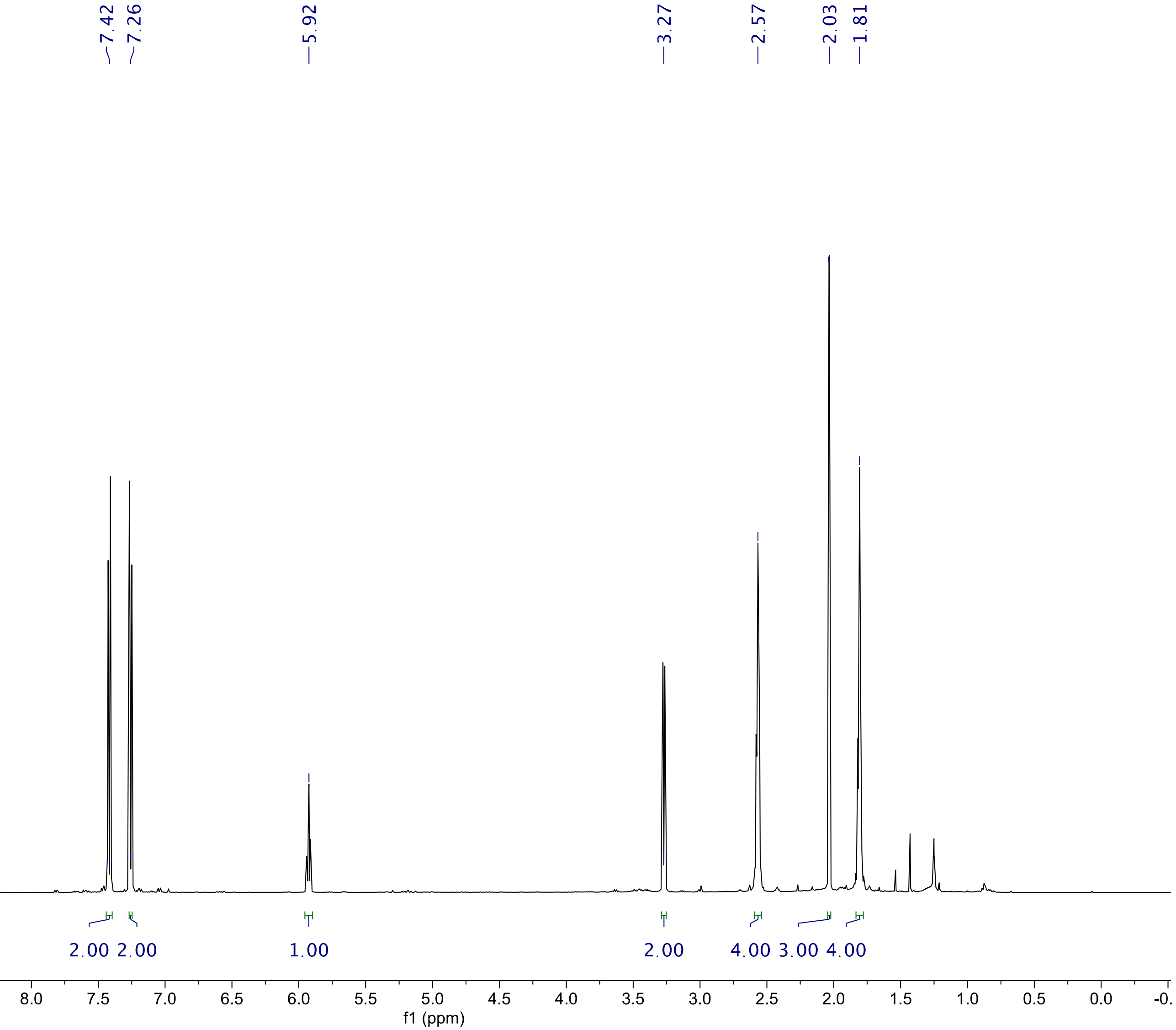

S56 – ^{13}C NMR (126 MHz, $CDCl_3$)

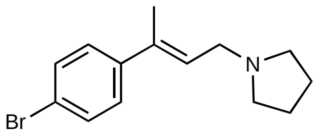

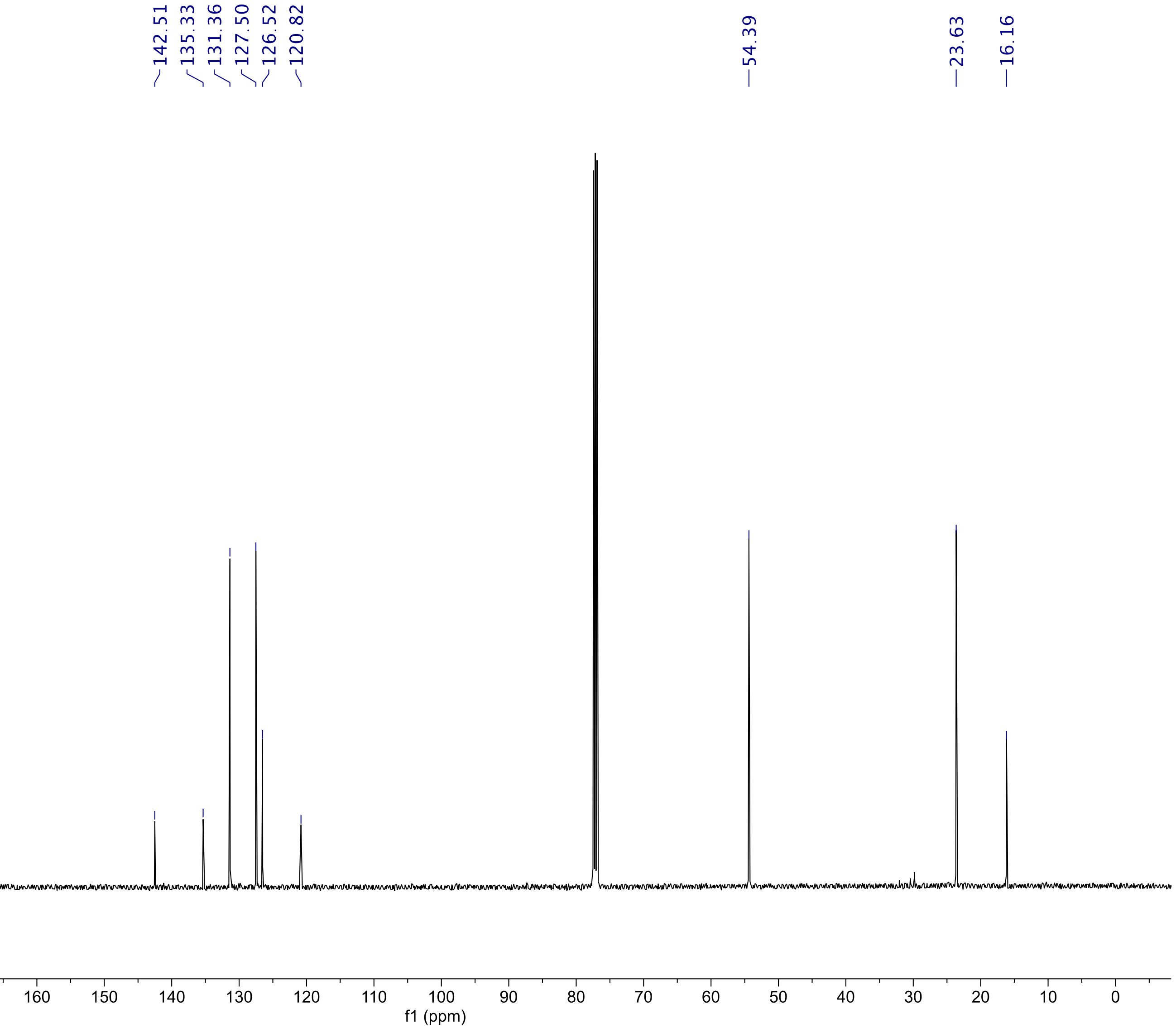

S57 – ^{1}H NMR (400 MHz, $CDCl_3$)

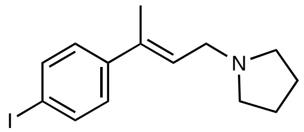

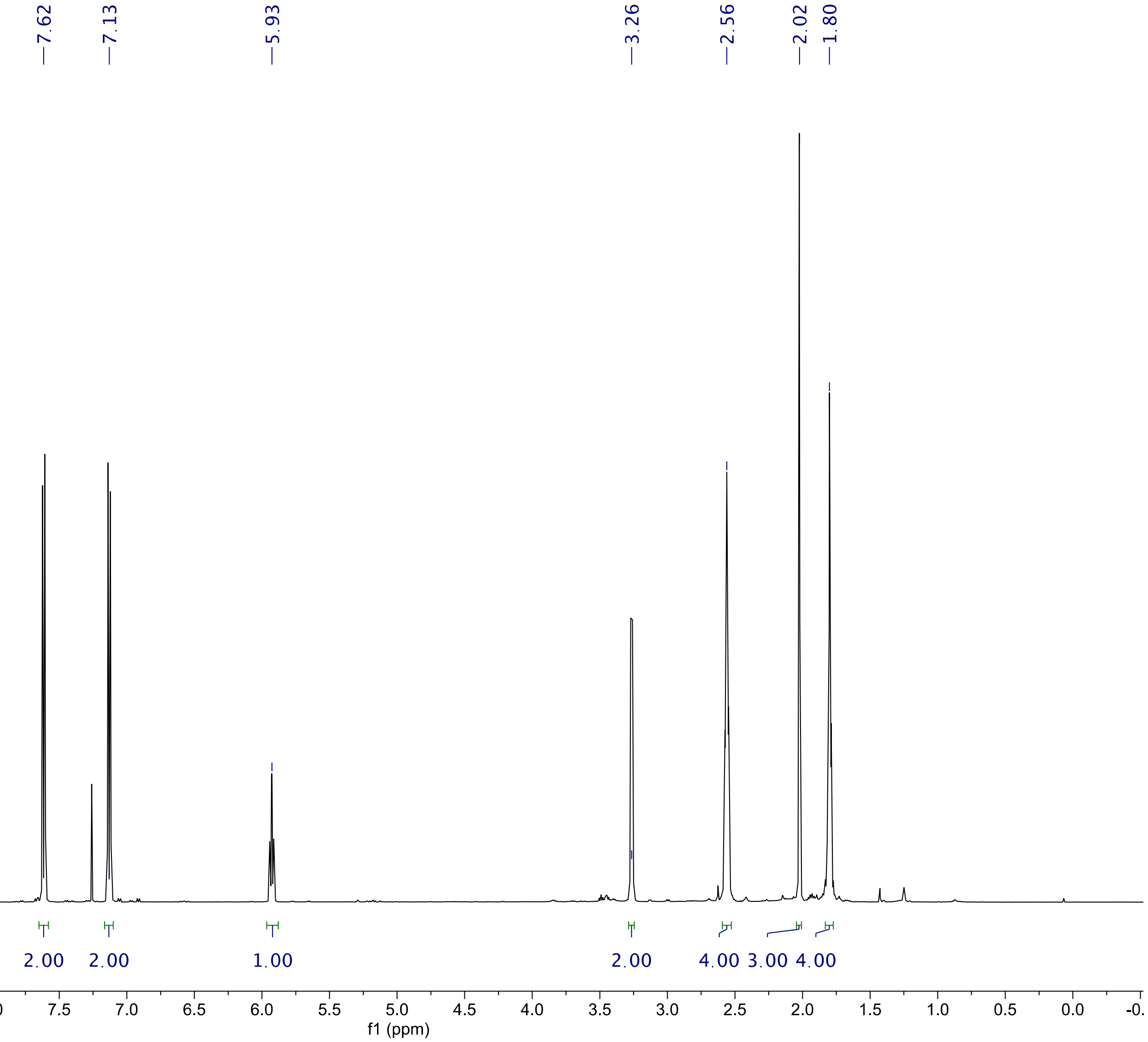

S57 – ^{13}C NMR (126 MHz, $CDCl_3$)

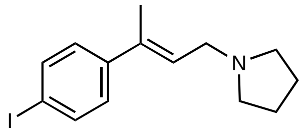

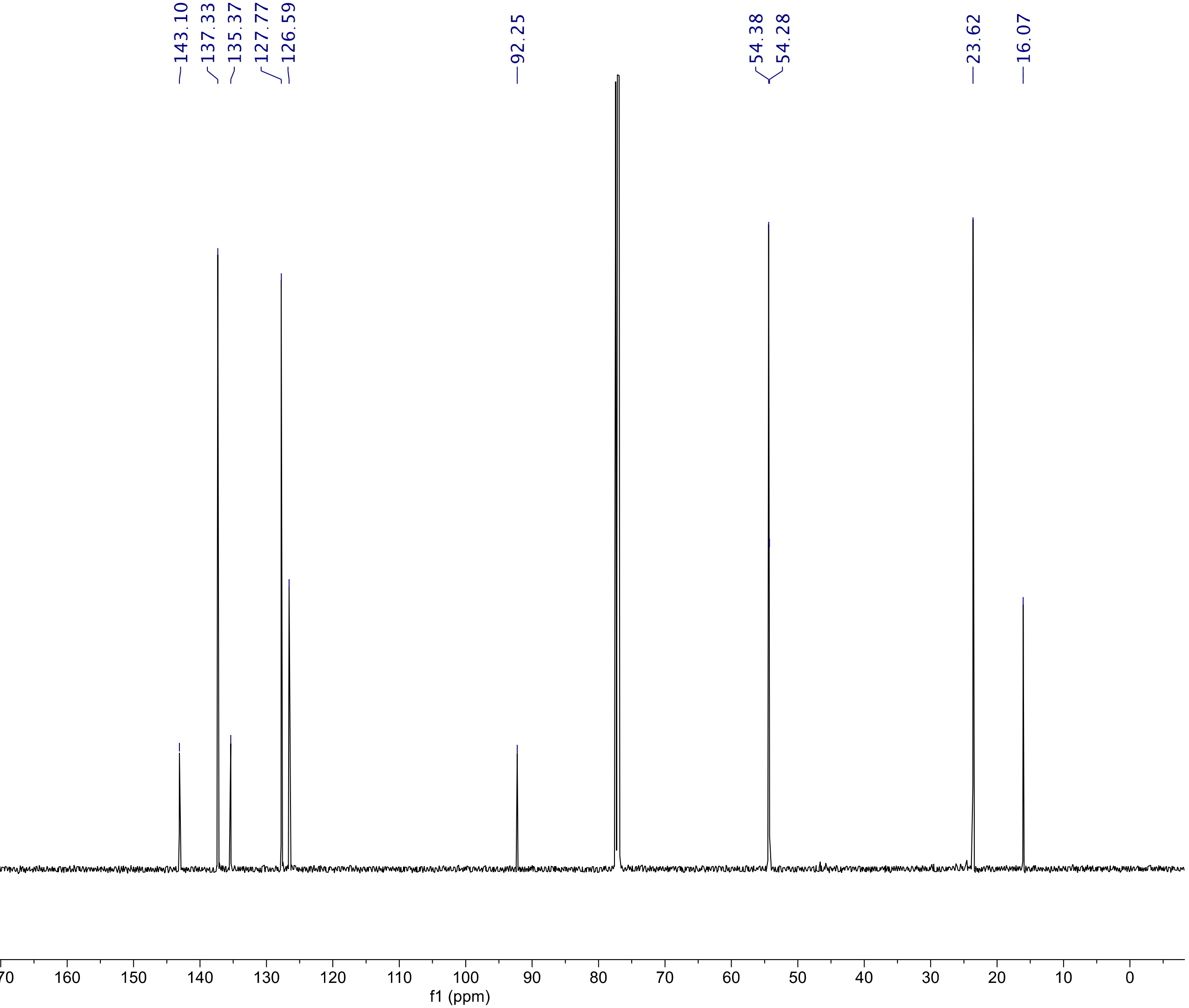

S58 – ^{1}H NMR (400 MHz, $CDCl_3$)

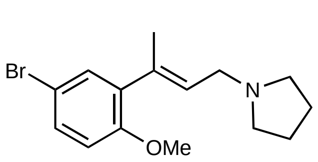

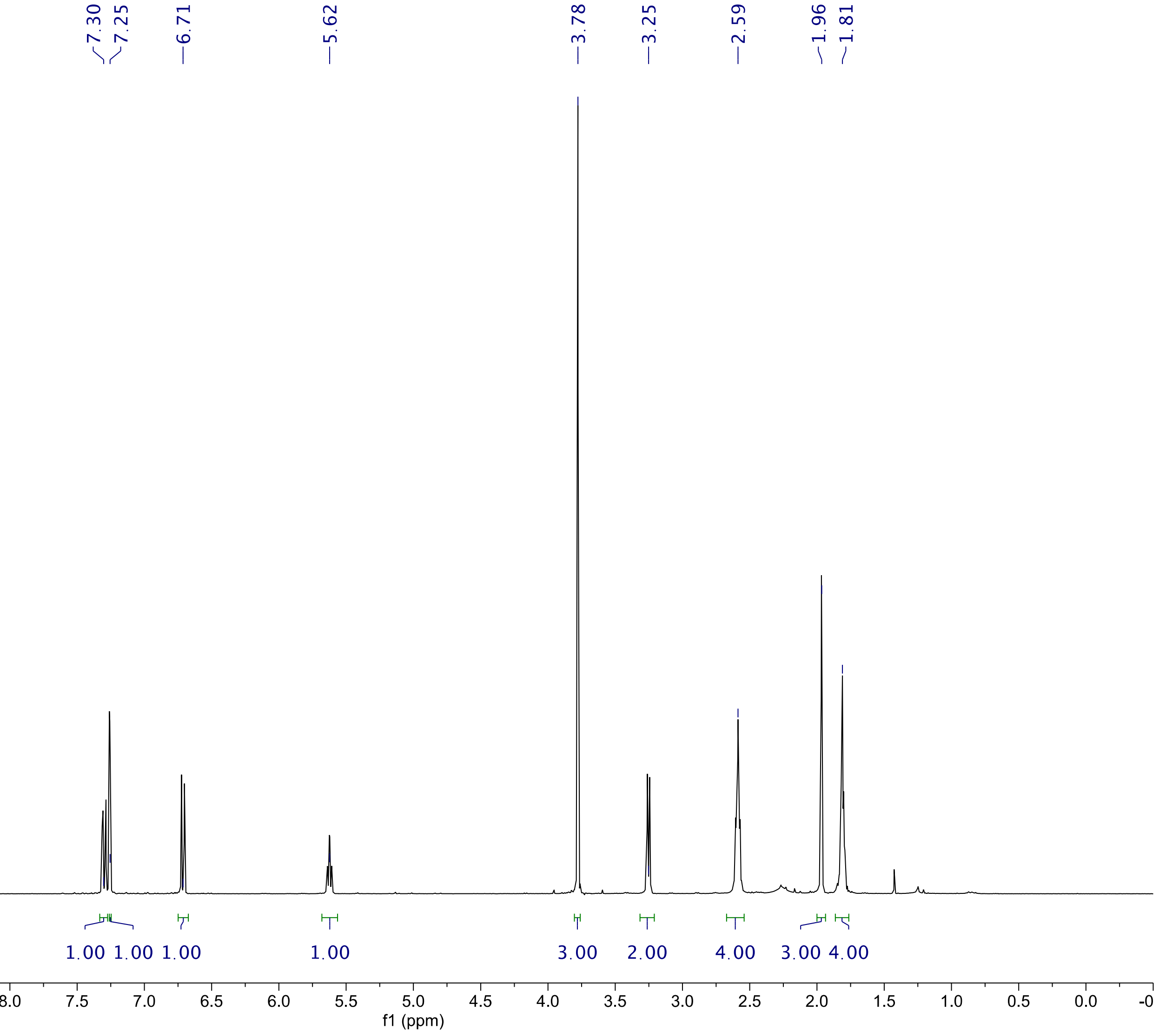

S58 – ^{13}C NMR (126 MHz, $CDCl_3$)

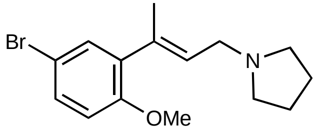

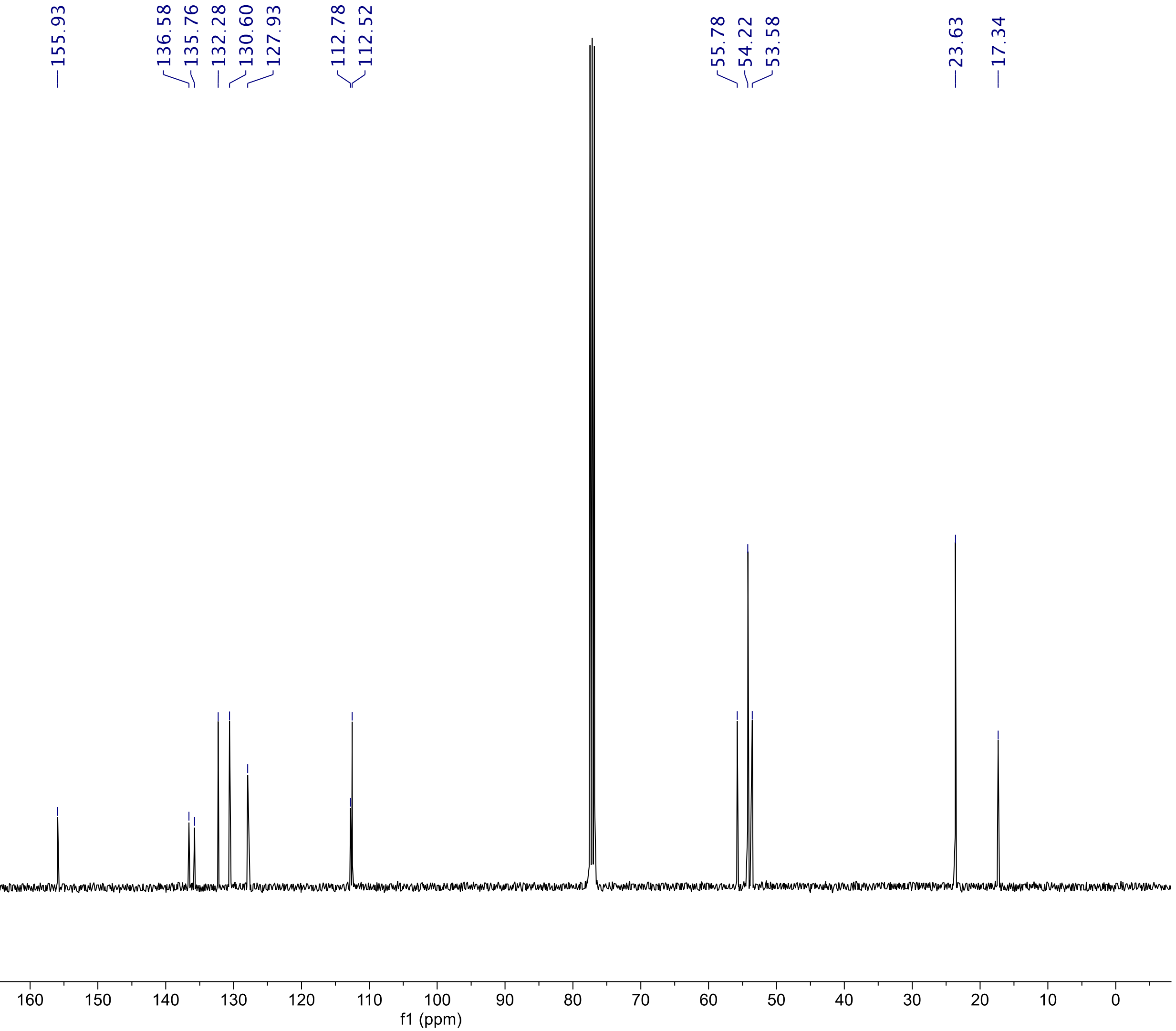

S59 – ^{1}H NMR (400 MHz, $CDCl_3$)

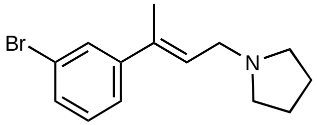

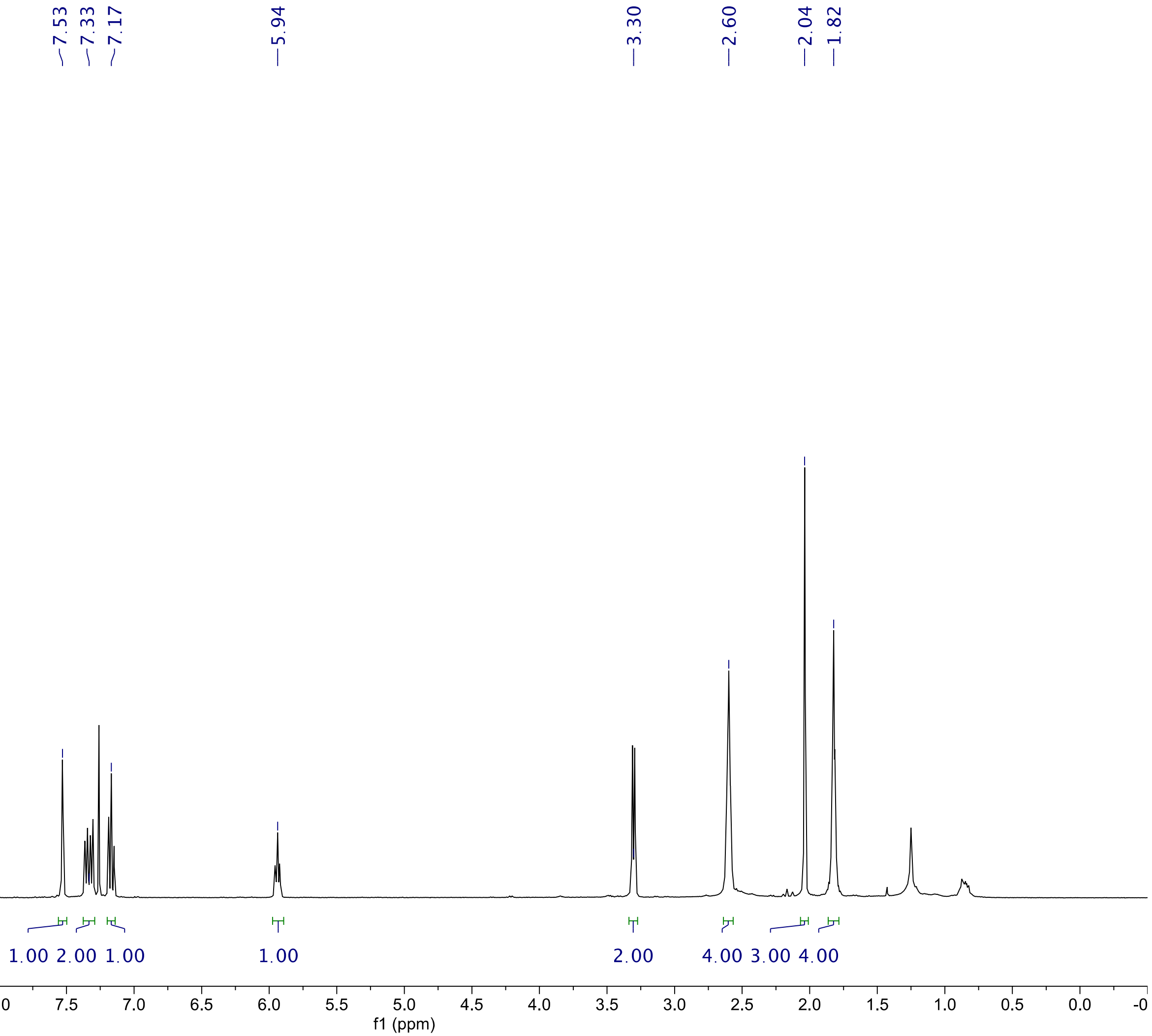

S59 – ^{13}C NMR (126 MHz, $CDCl_3$)

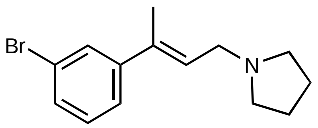

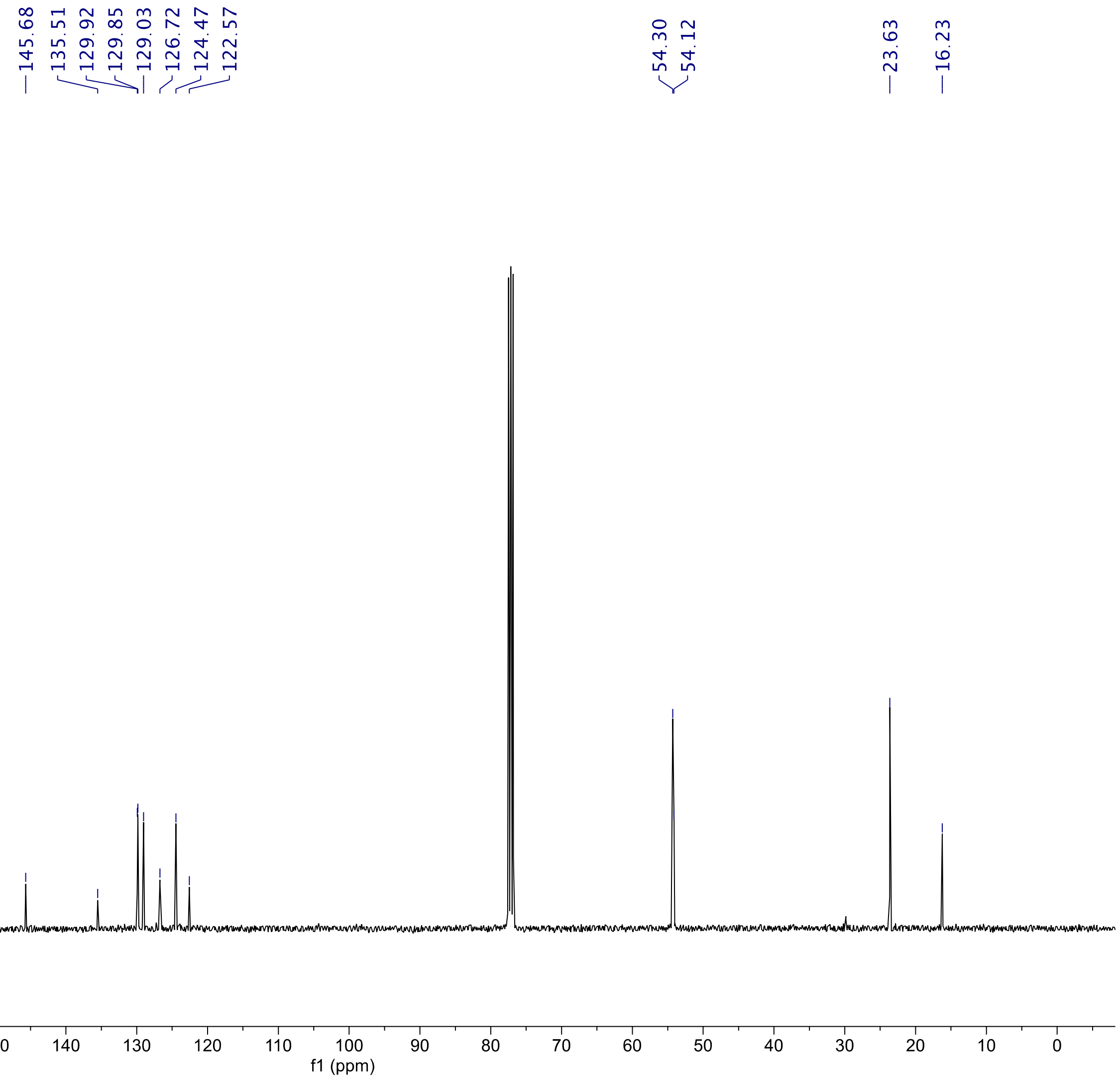

2a – ^{1}H NMR (400 MHz, d6-DMSO)

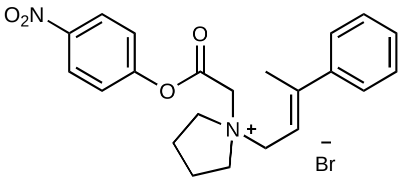

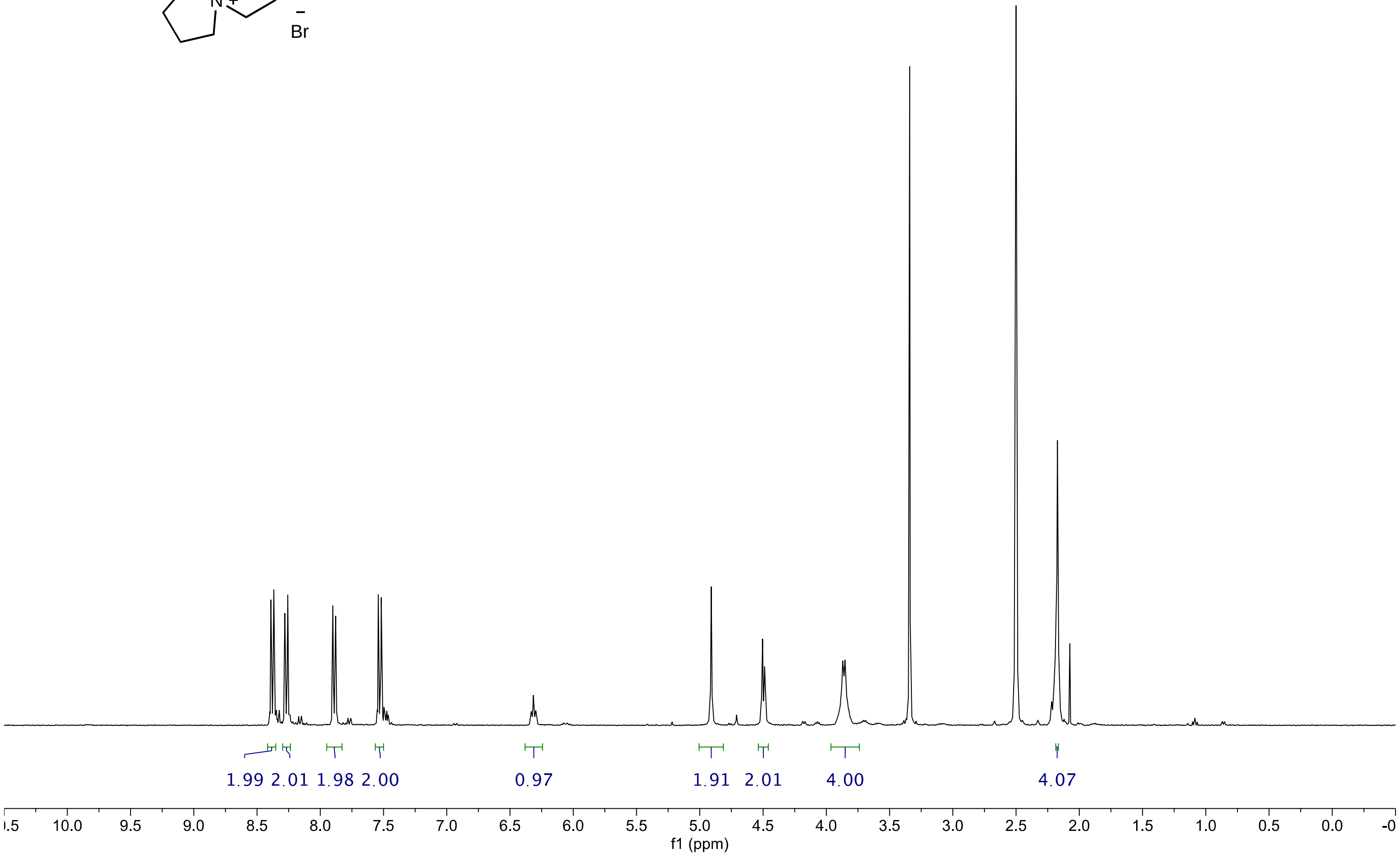

2a – ^{13}C NMR (126 MHz, d6-DMSO)

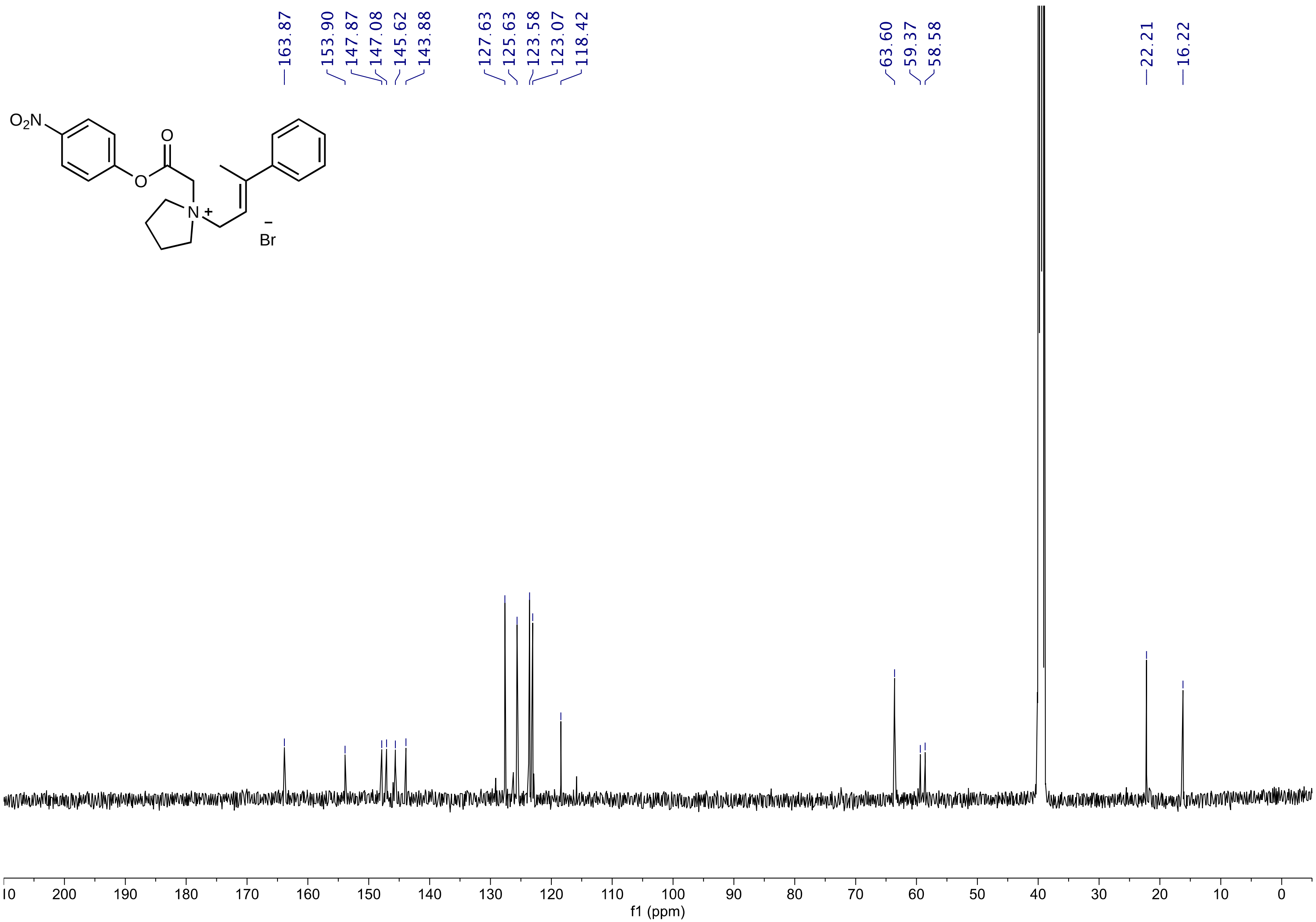

2b – ^{1}H NMR (400 MHz, d6-DMSO)

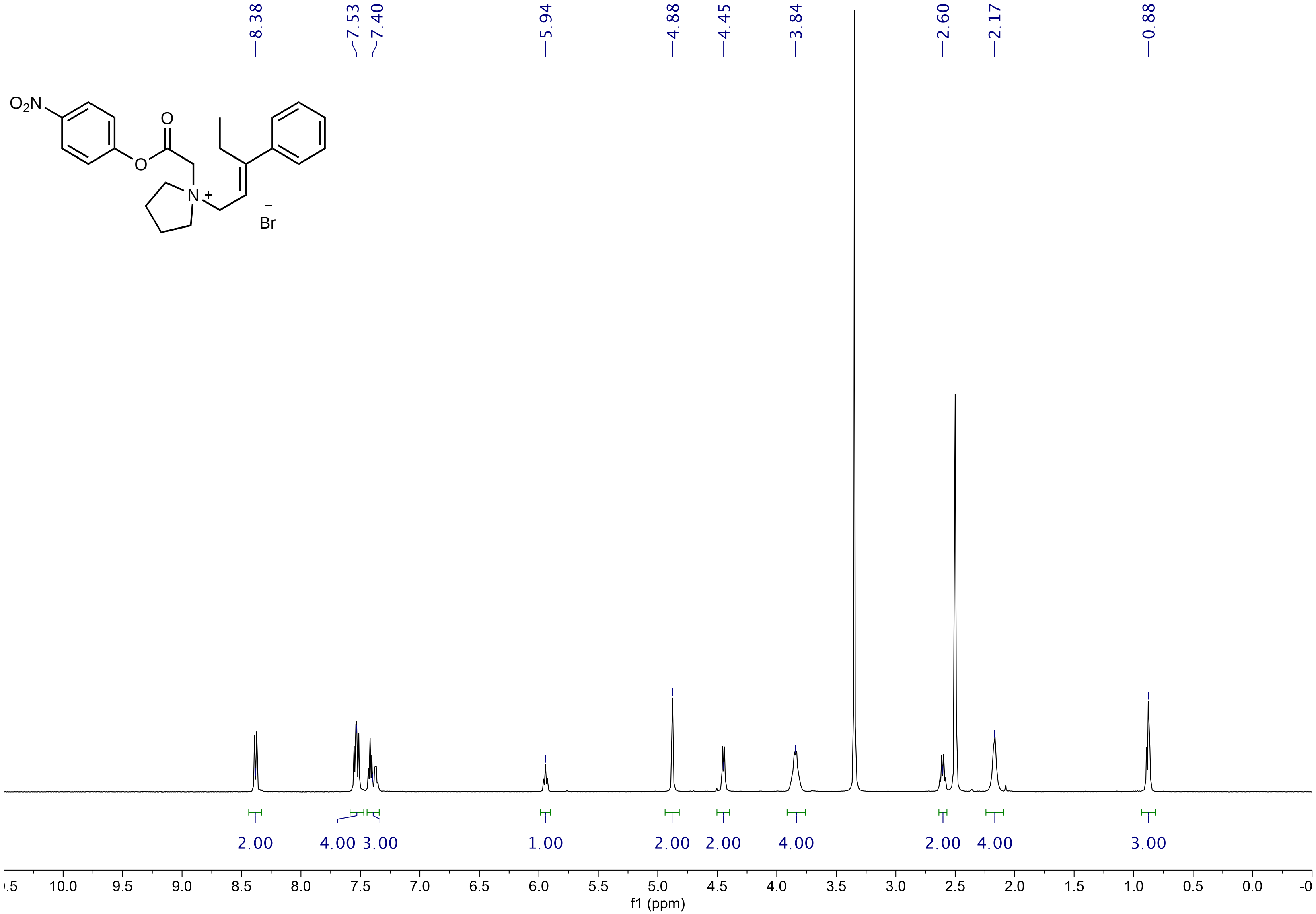

2b – ^{13}C NMR (126 MHz, d6-DMSO)

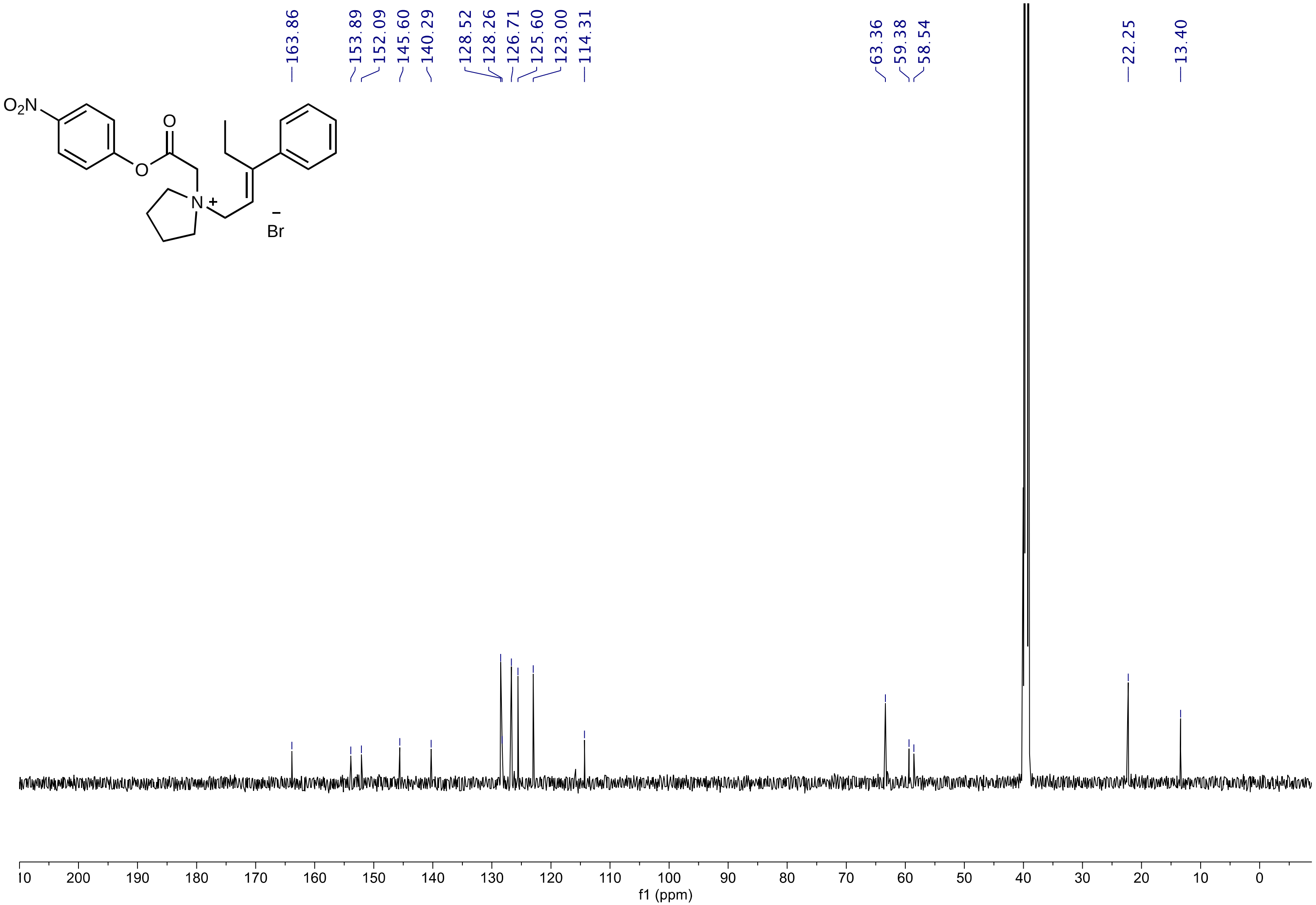

2c – ^{1}H NMR (400 MHz, d6-DMSO)

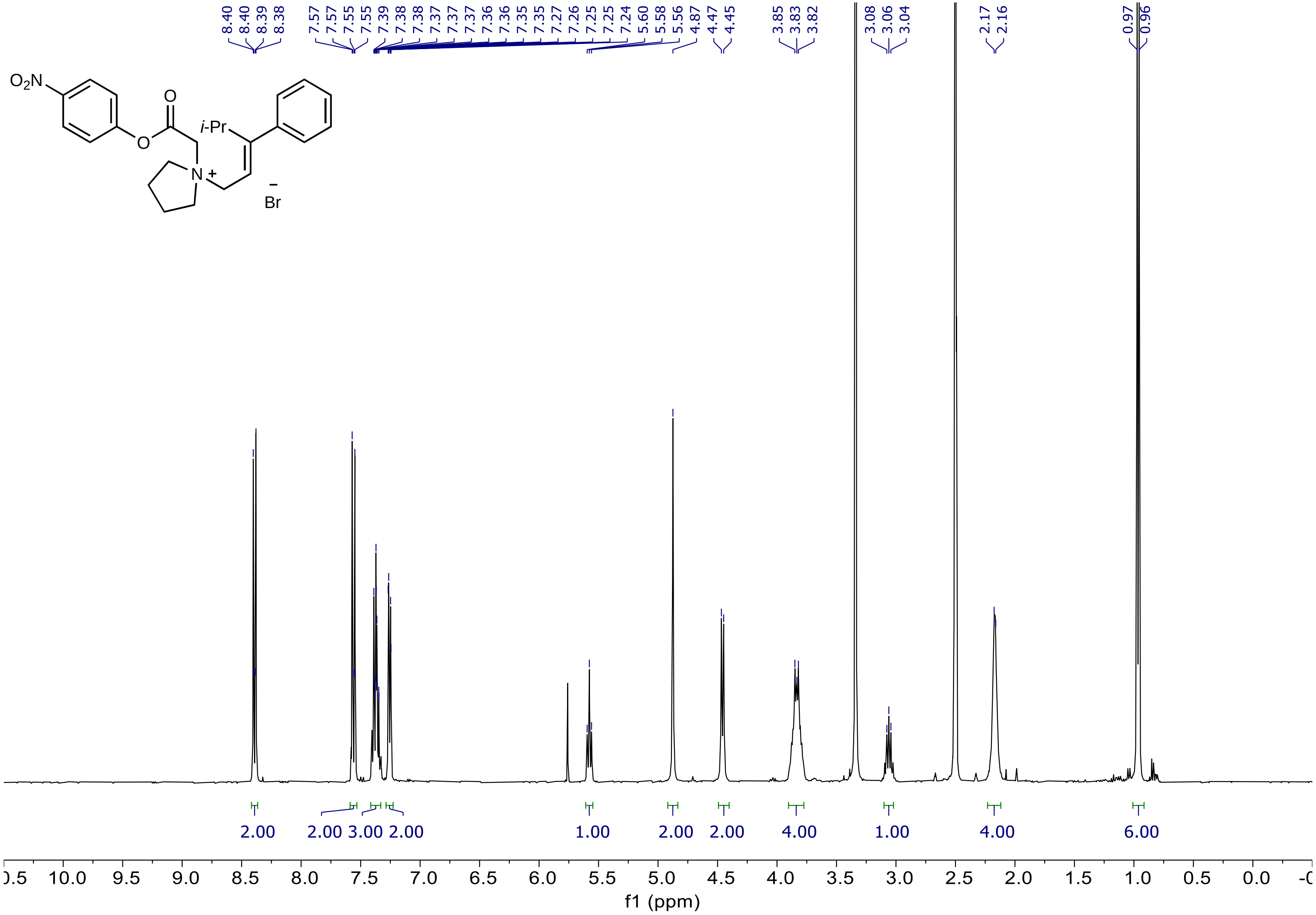

2c – ^{13}C NMR (126 MHz, d6-DMSO)

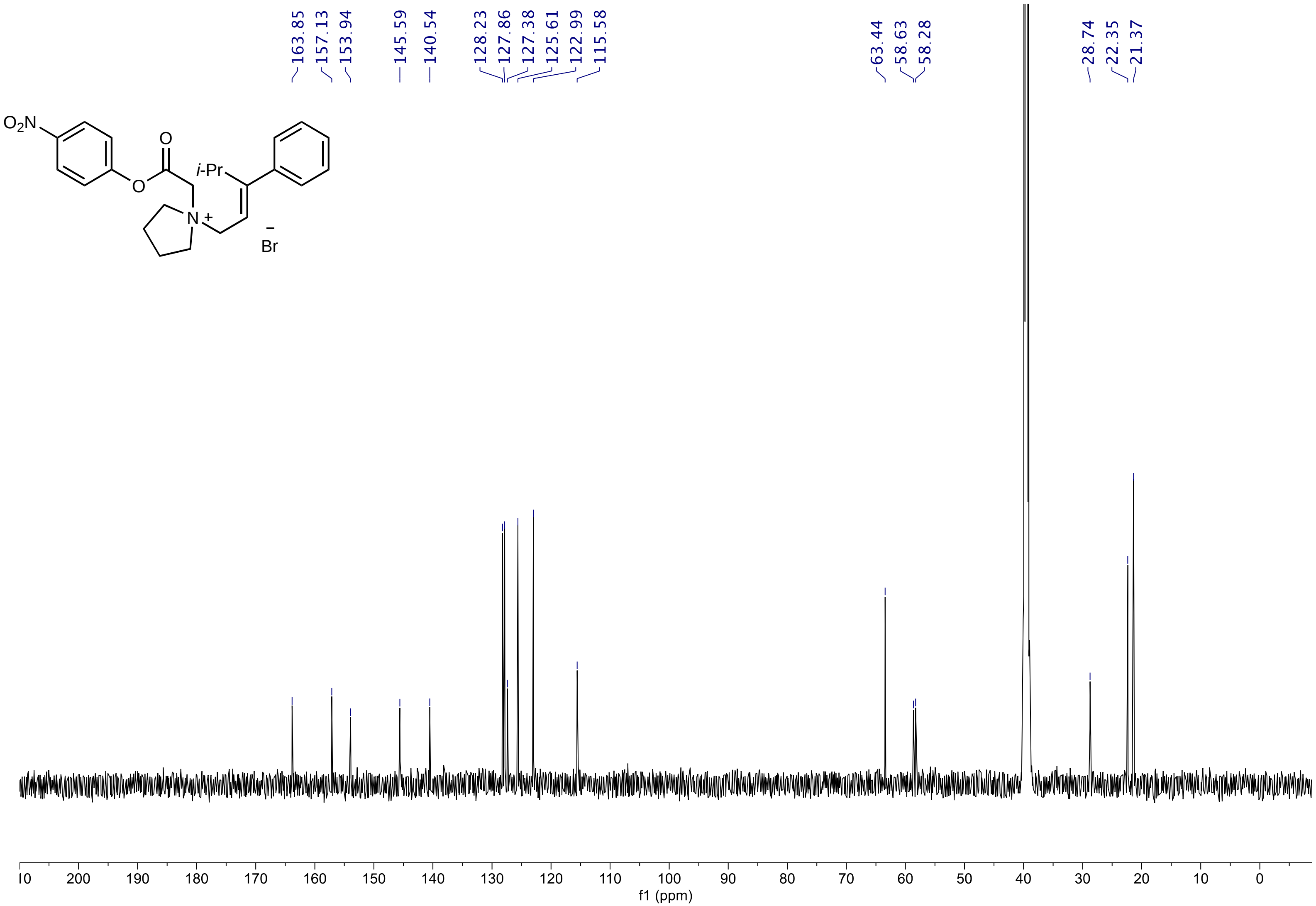

2d – ¹H NMR (400 MHz, d6-DMSO)

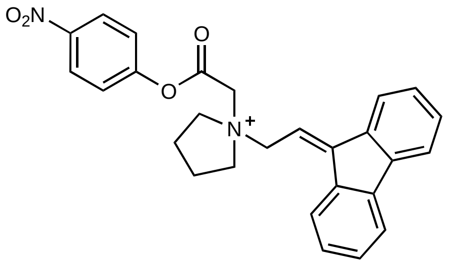

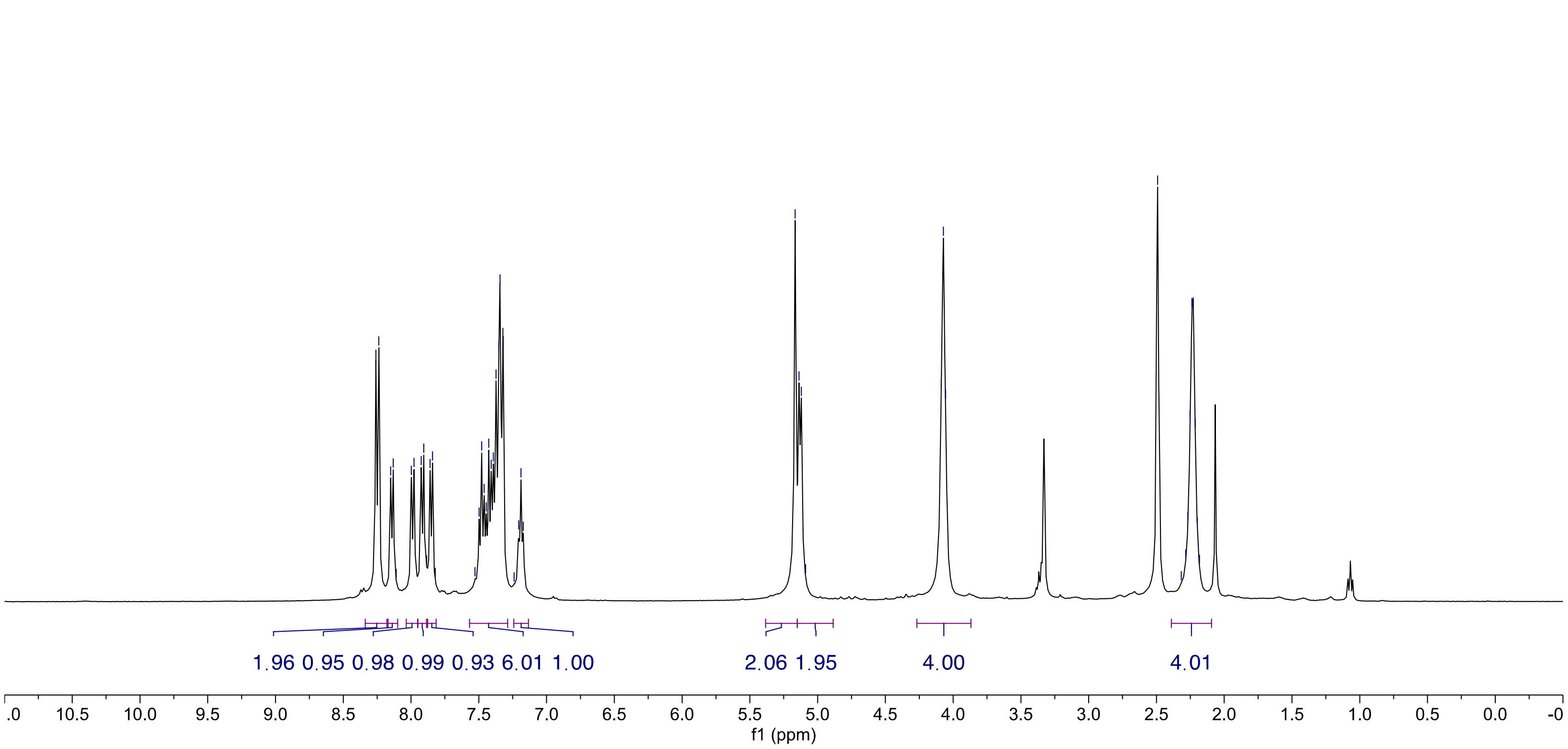

2d – ^{13}C NMR (126 MHz, d6-DMSO)

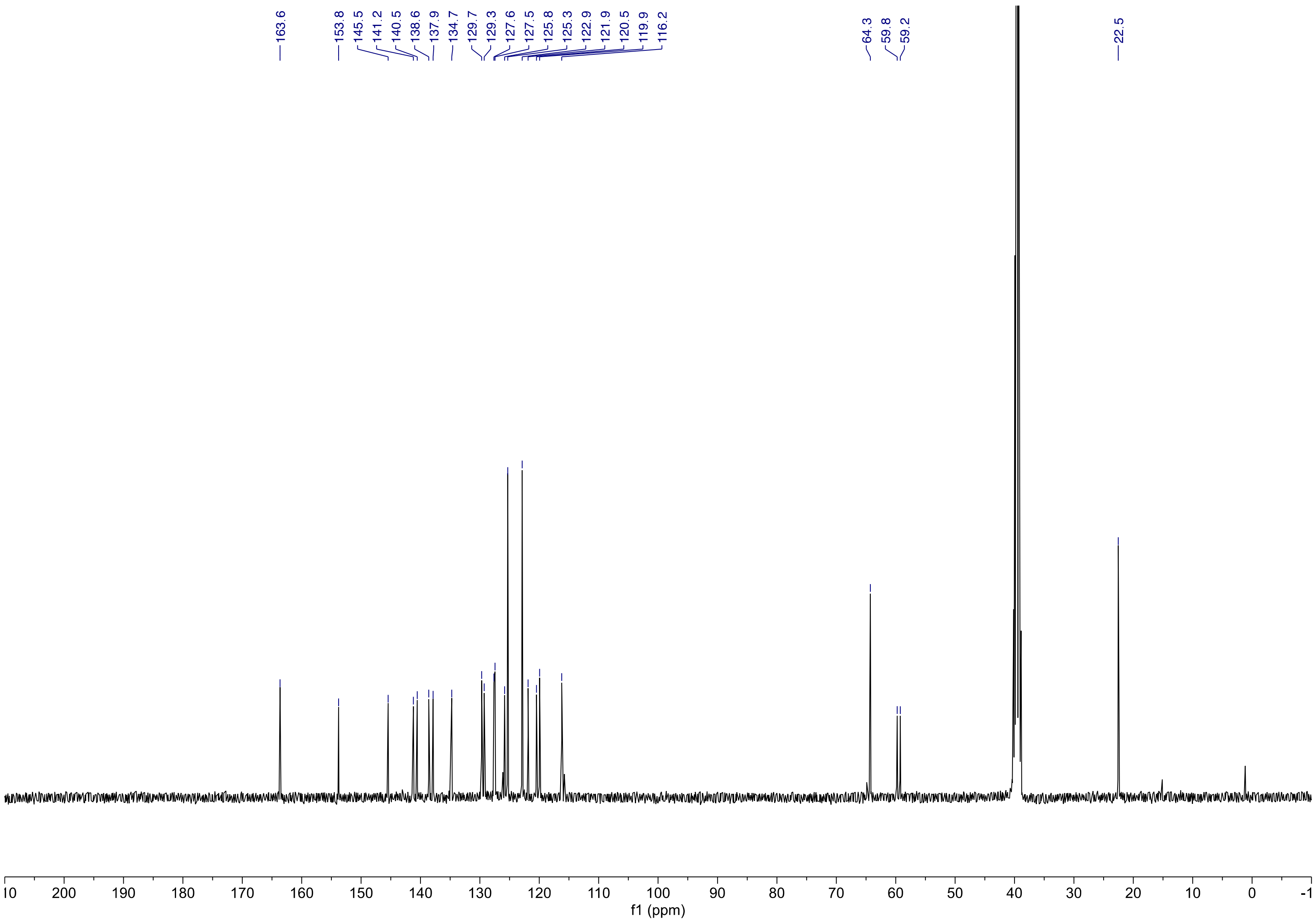

2e – ^{1}H NMR (400 MHz, d6-DMSO)

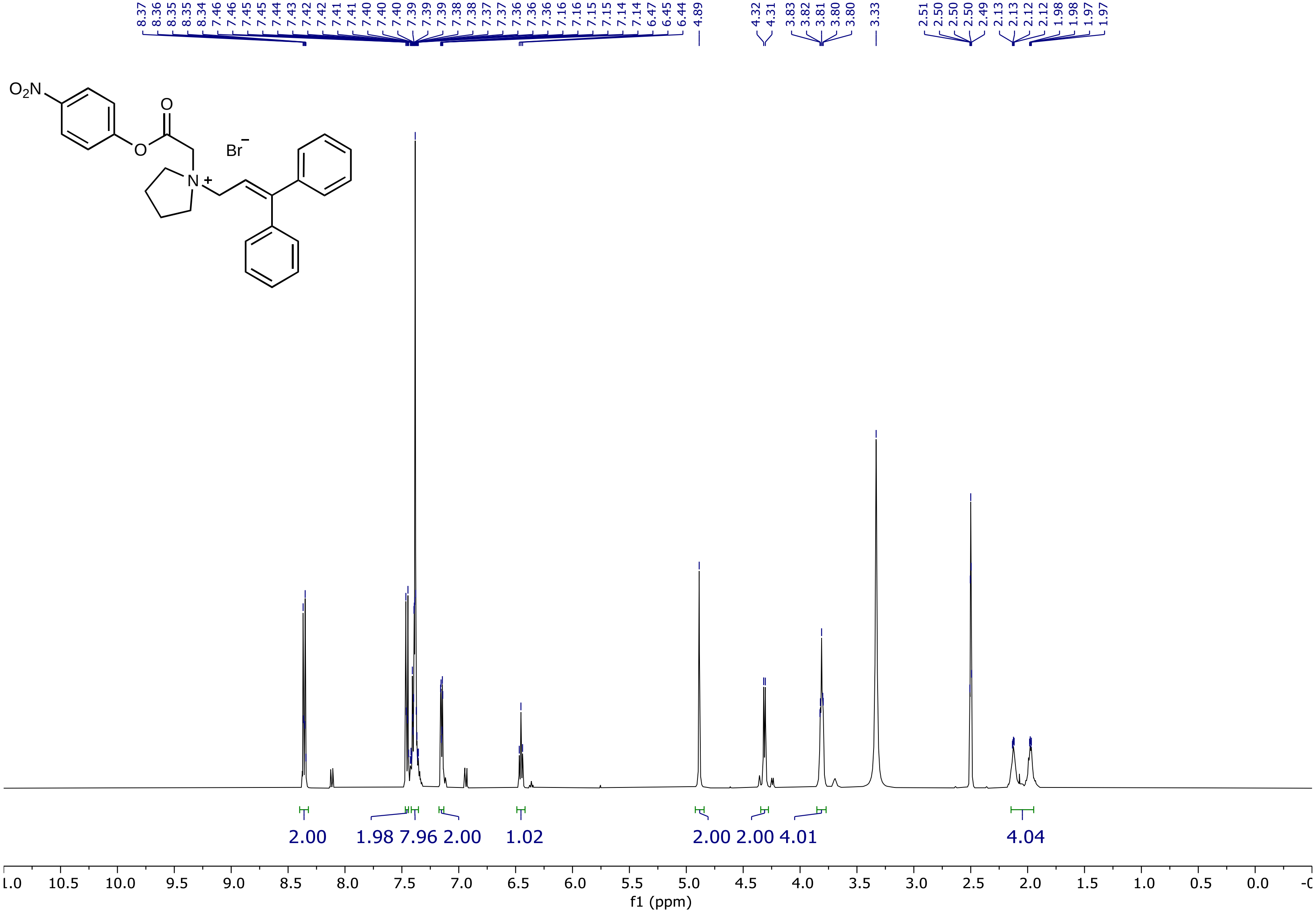

2e – ^{13}C NMR (126 MHz, d6-DMSO)

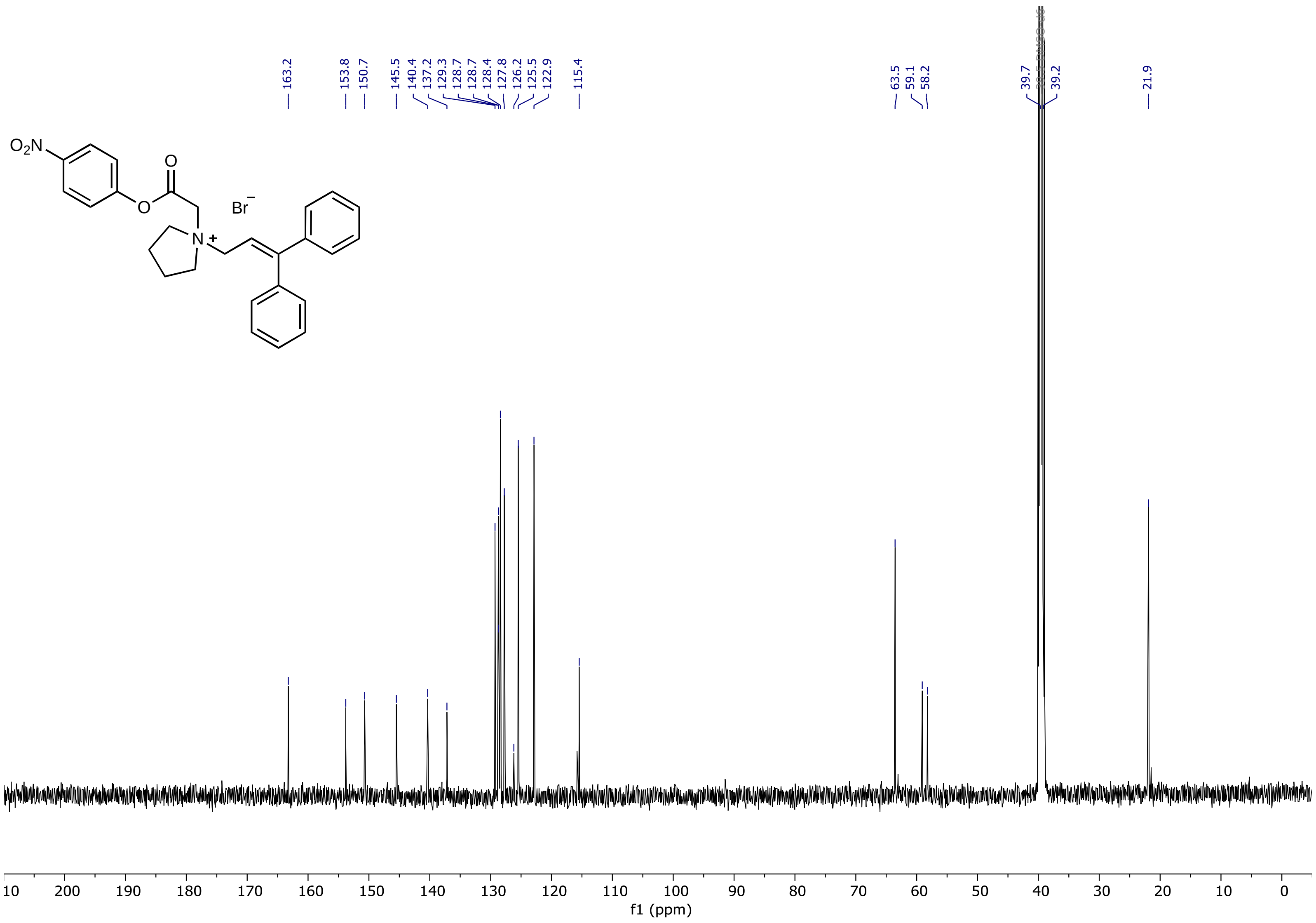

2f – ^{1}H NMR (400 MHz, d6-DMSO)

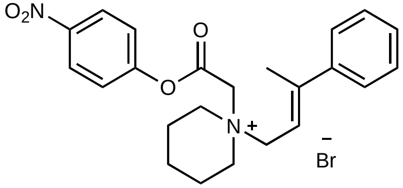

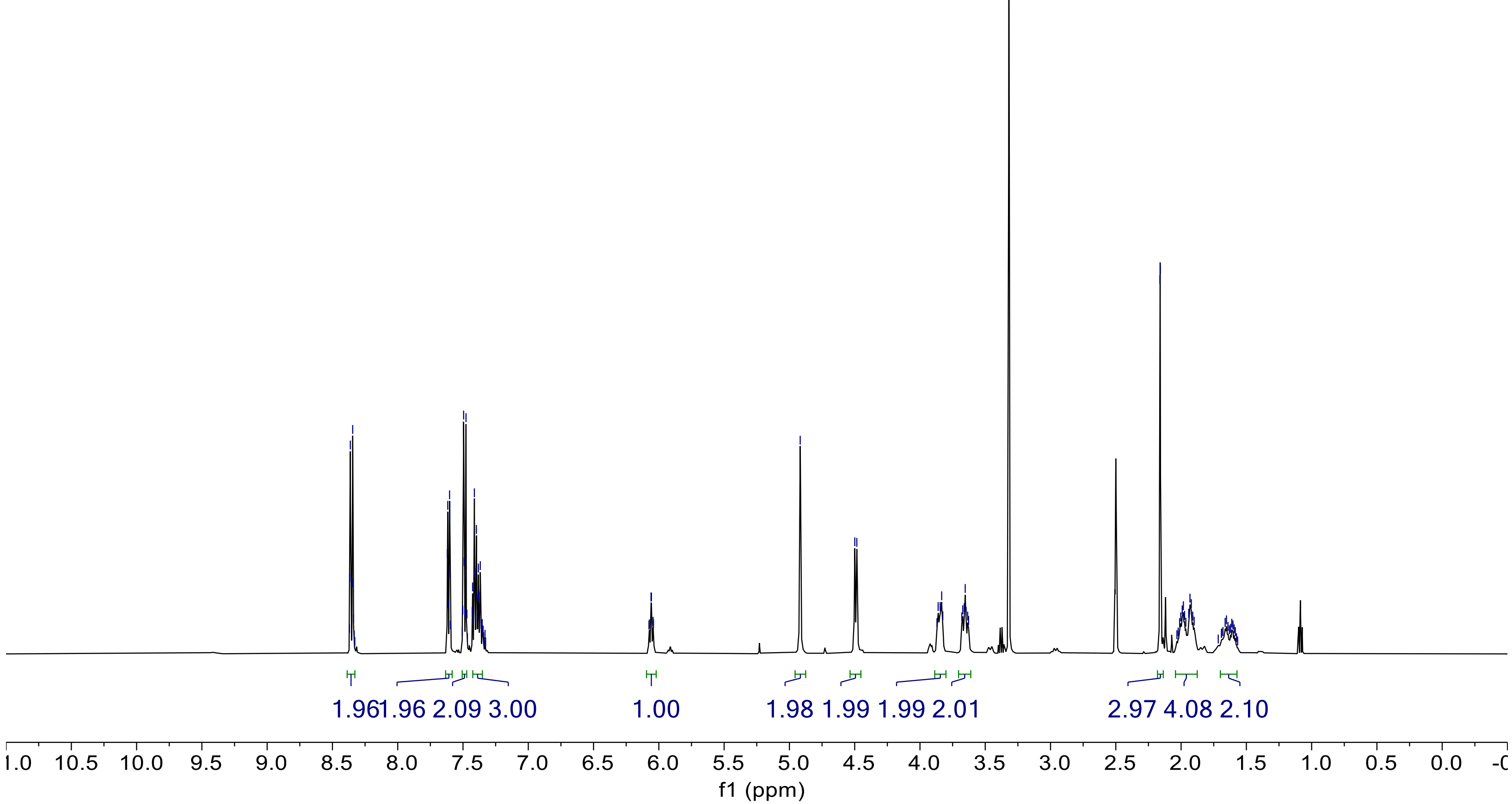

2f – ^{13}C NMR (126 MHz, d6-DMSO)

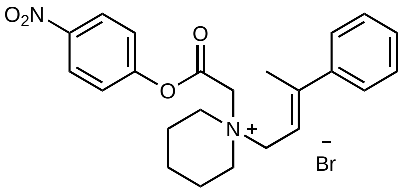

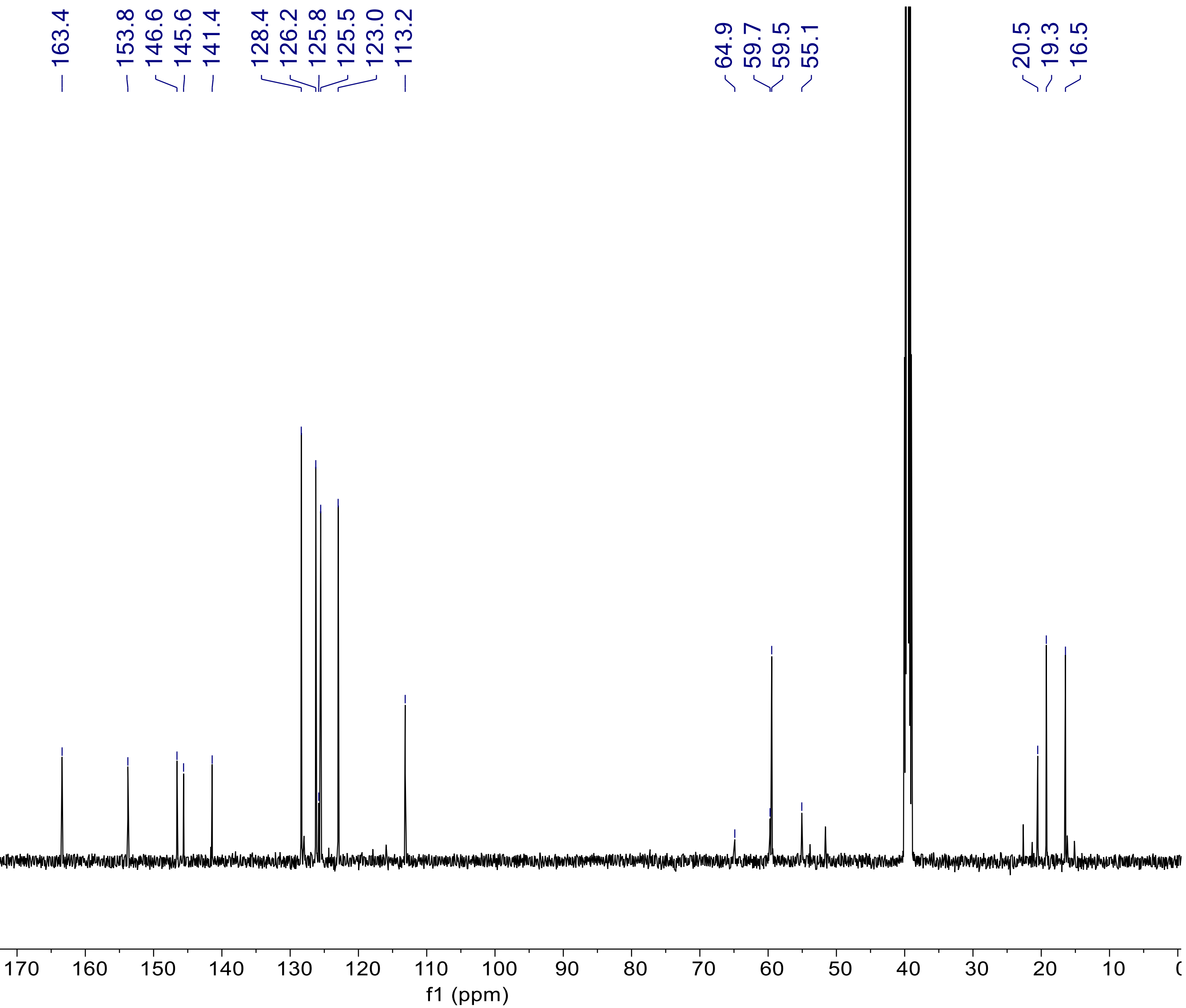

2g – ^{1}H NMR (400 MHz, d6-DMSO)

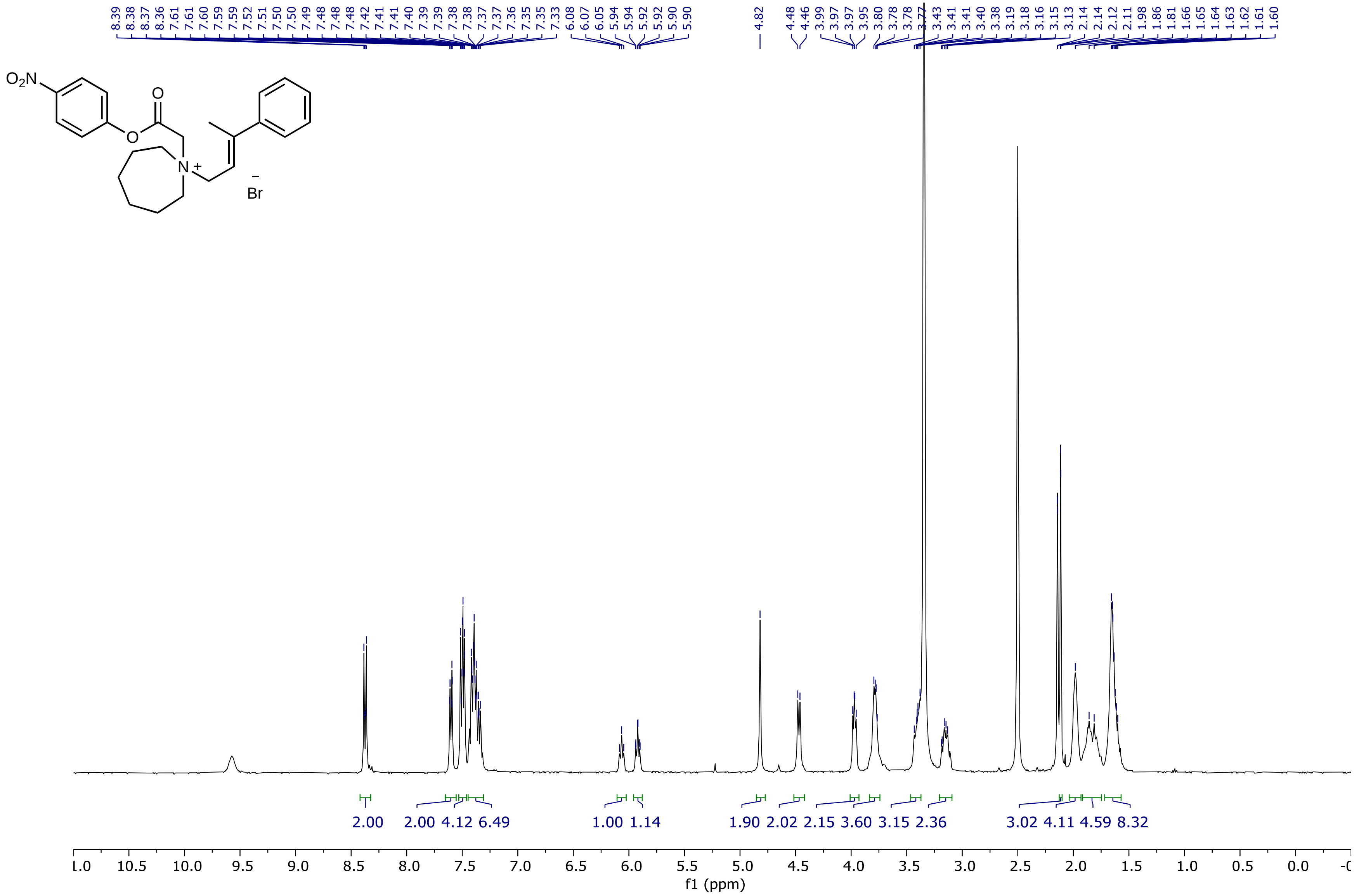

2g – ^{13}C NMR (126 MHz, d6-DMSO)

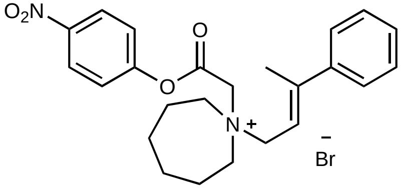

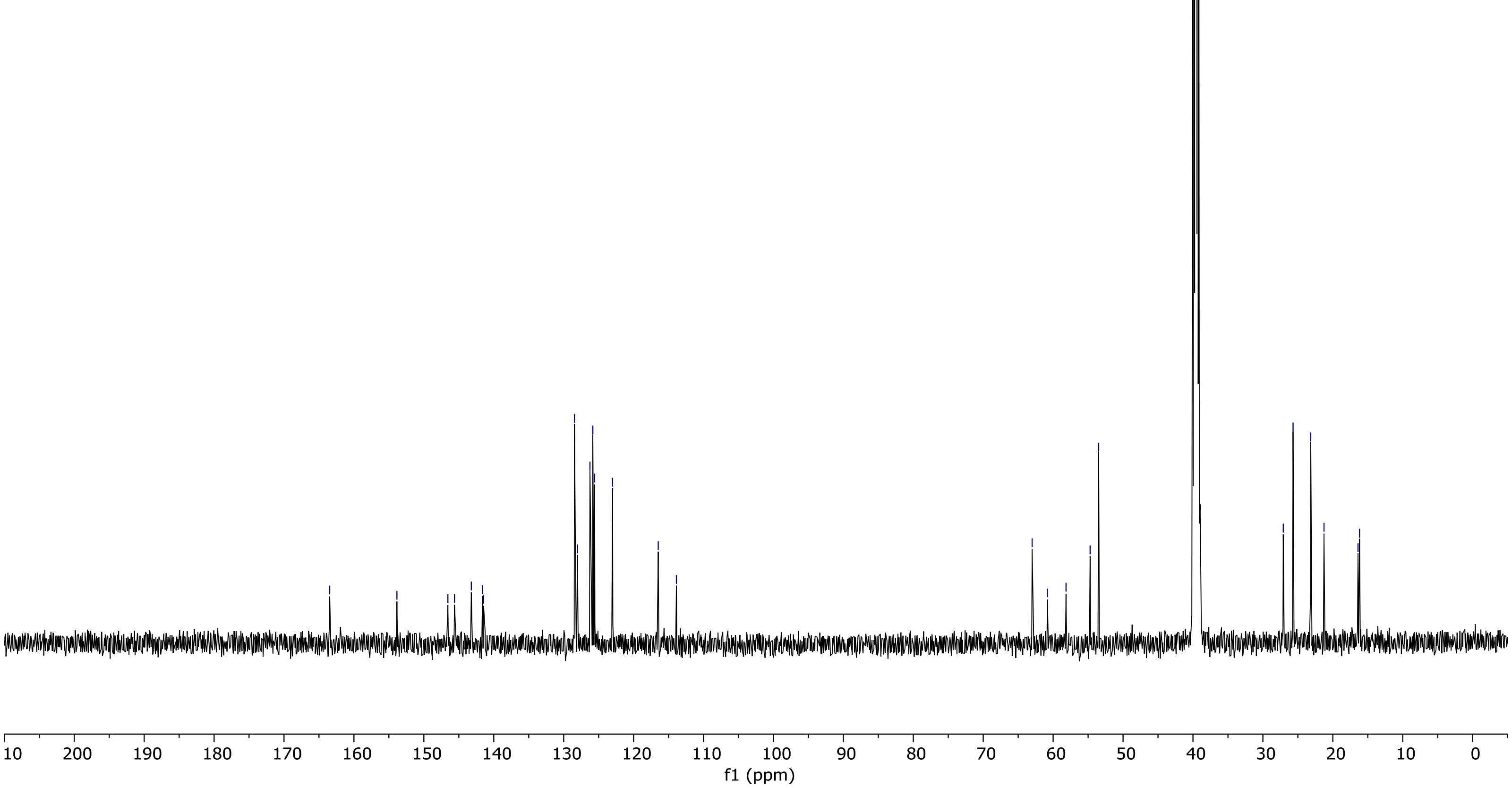

2h – ^{1}H NMR (400 MHz, d6-DMSO)

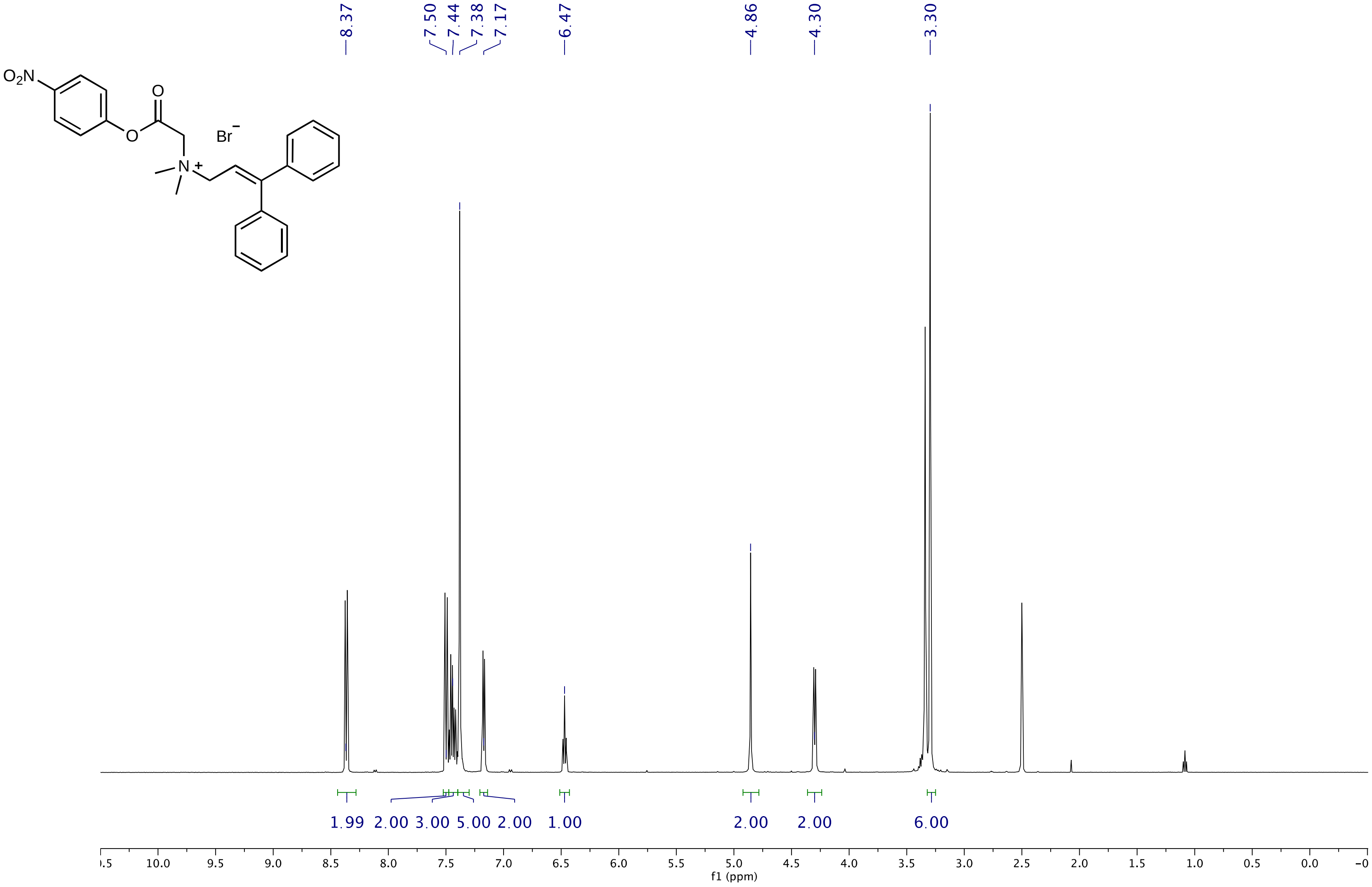

2h – ^{13}C NMR (126 MHz, d6-DMSO)

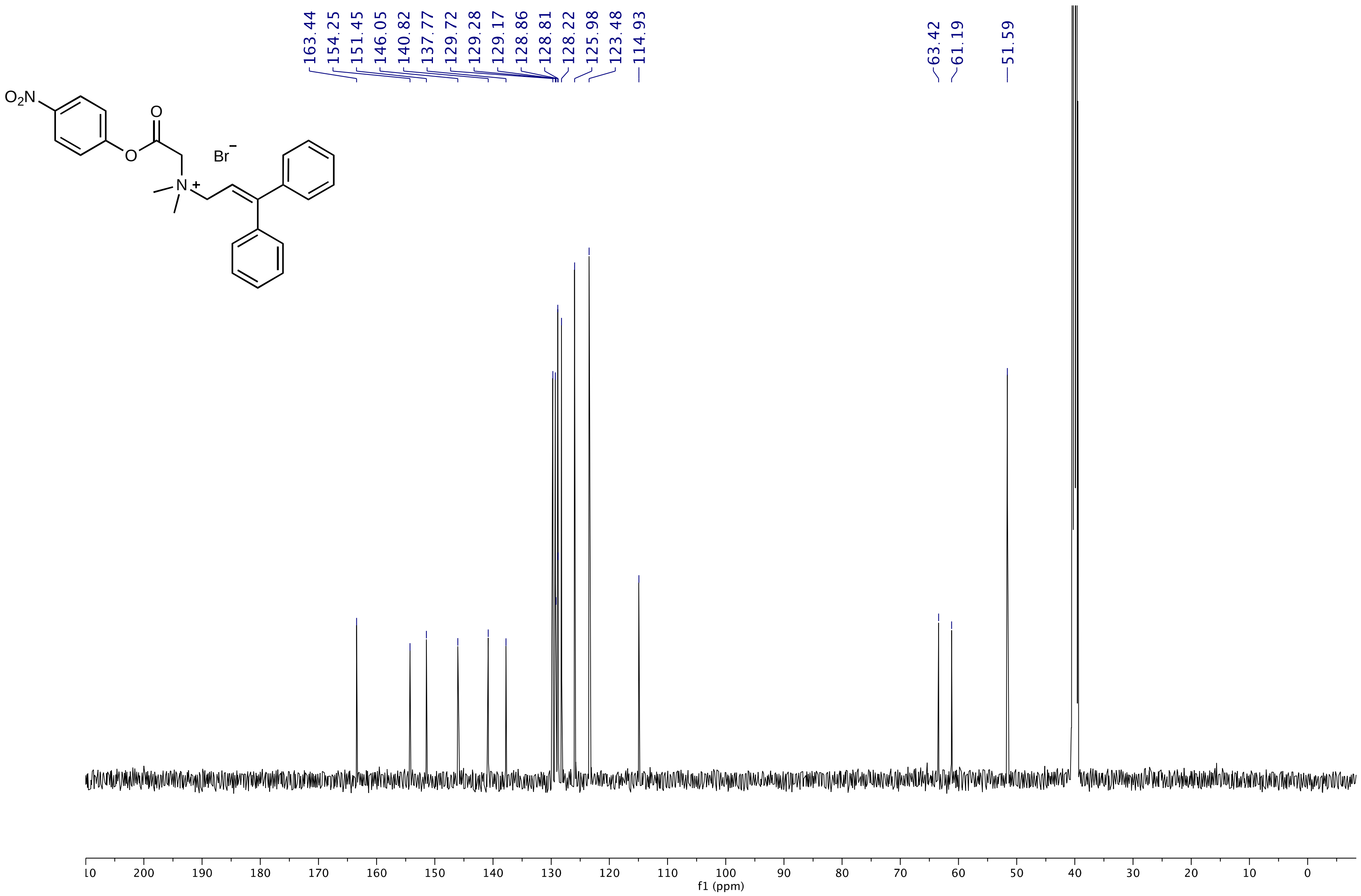

2i – ^{1}H NMR (400 MHz, d6-DMSO)

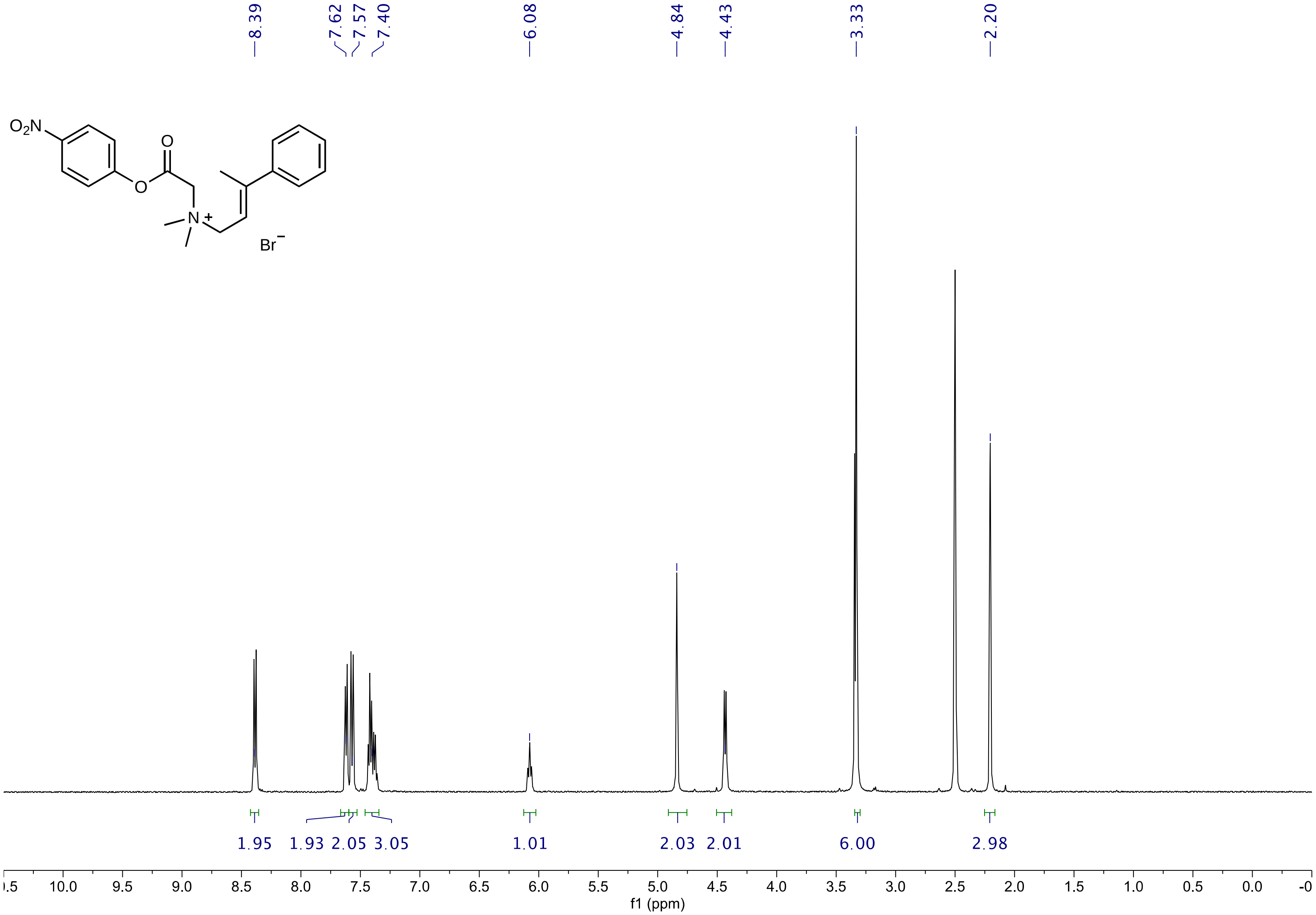

2i – ^{13}C NMR (126 MHz, d6-DMSO)

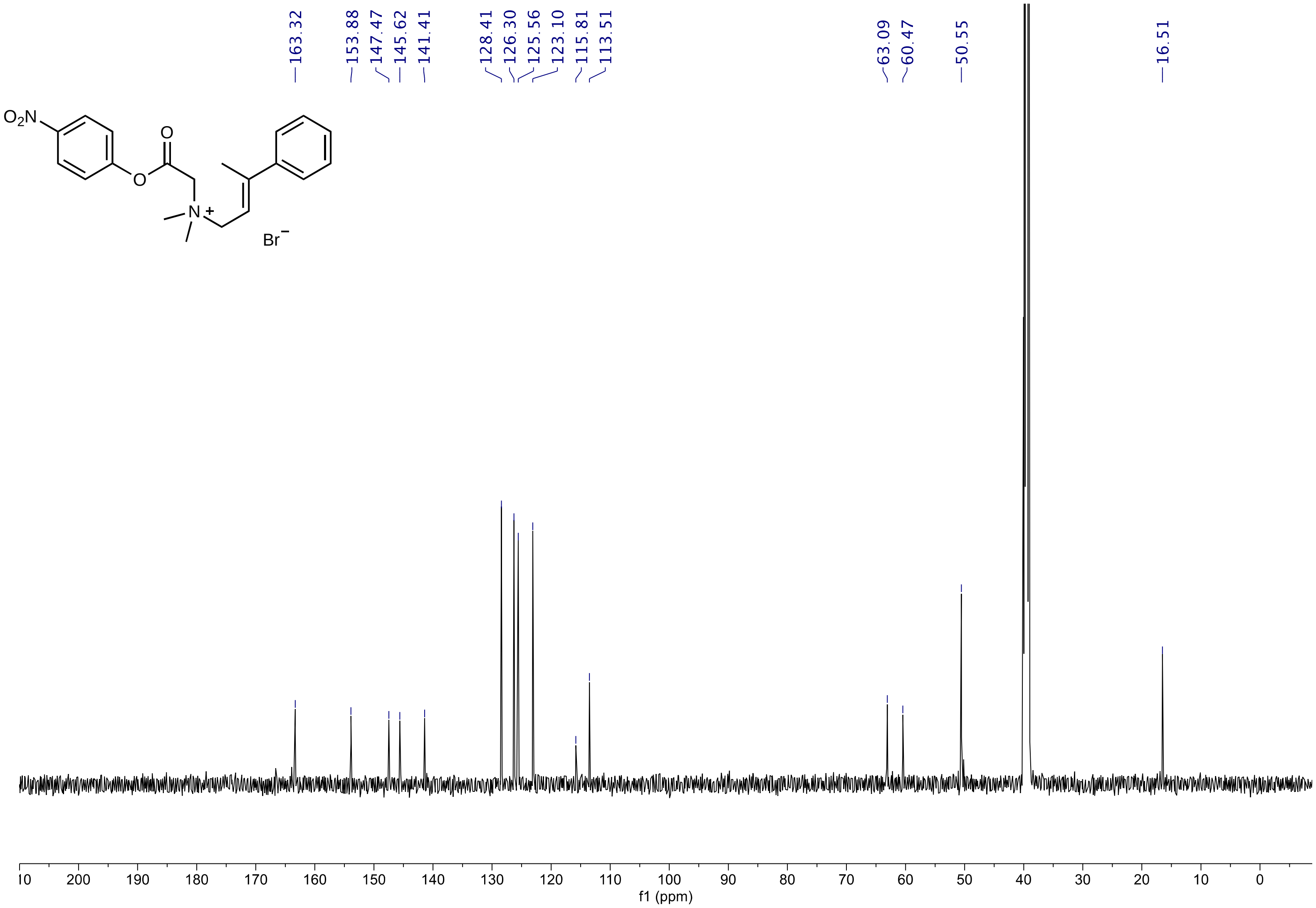

2j – ^{1}H NMR (400 MHz, d6-DMSO)

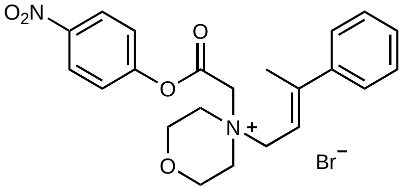

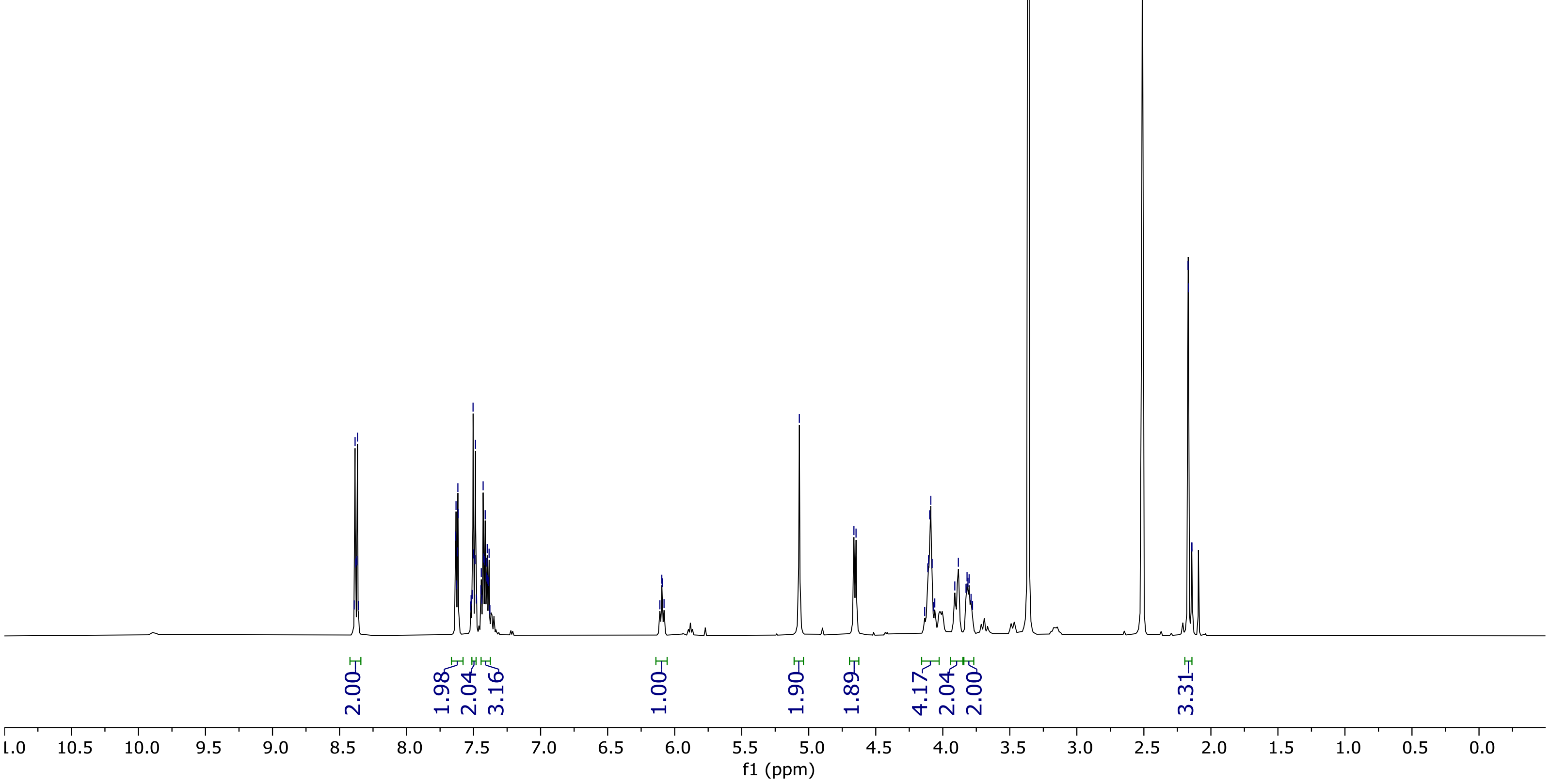

2j – ^{13}C NMR (126 MHz, d6-DMSO)

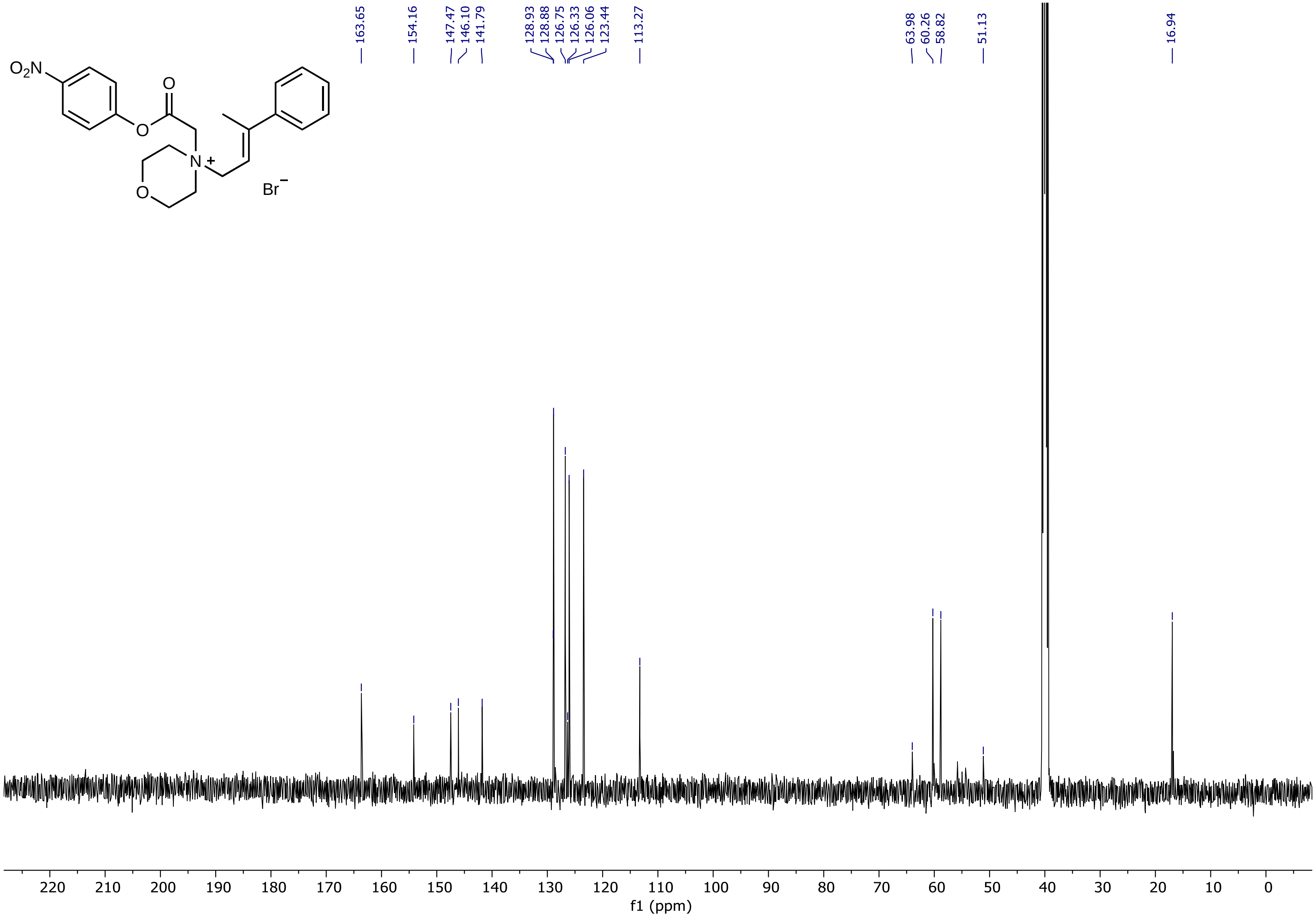

2k – ^{1}H NMR (400 MHz, d6-DMSO)

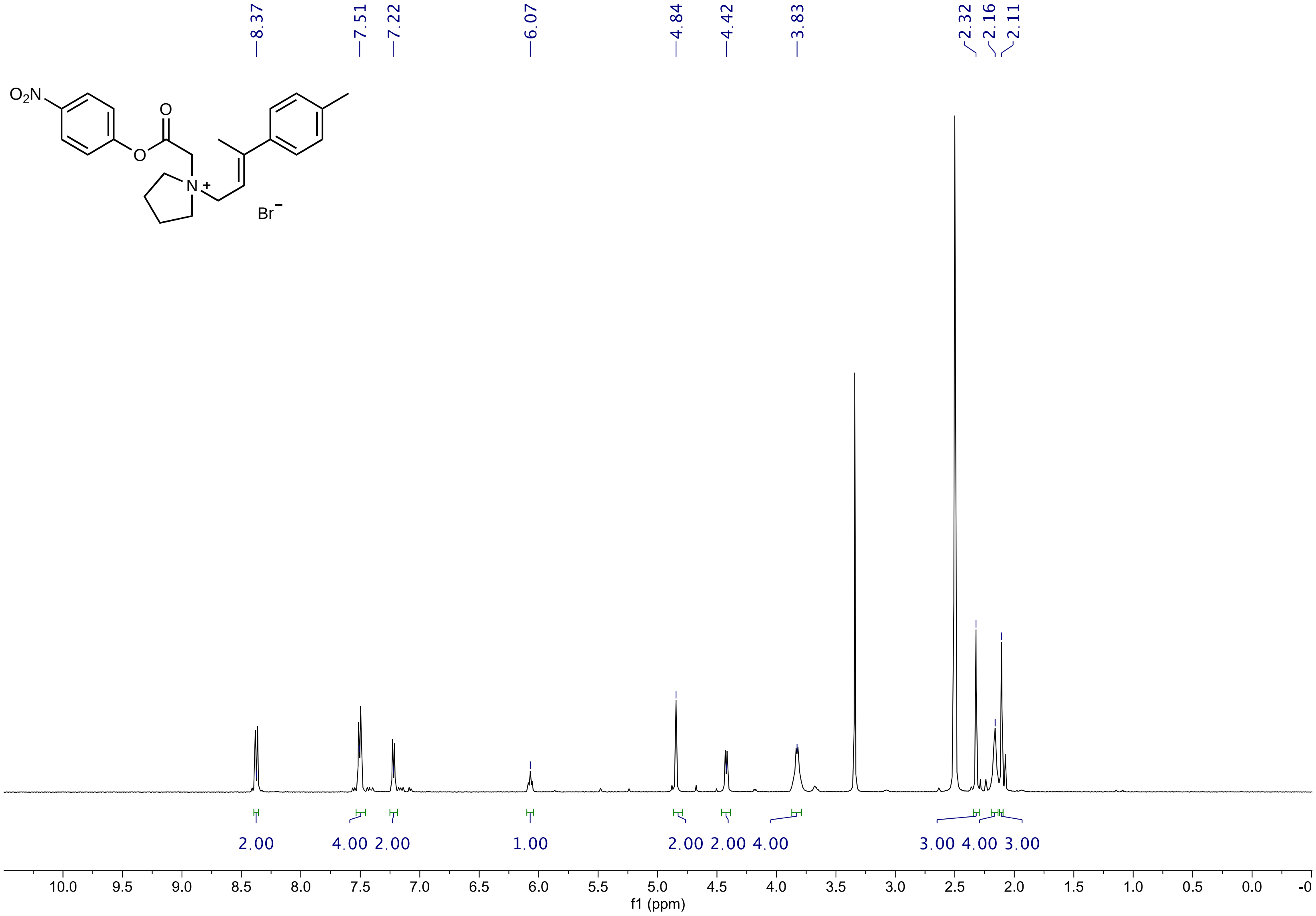

2k – ^{13}C NMR (126 MHz, d6-DMSO)

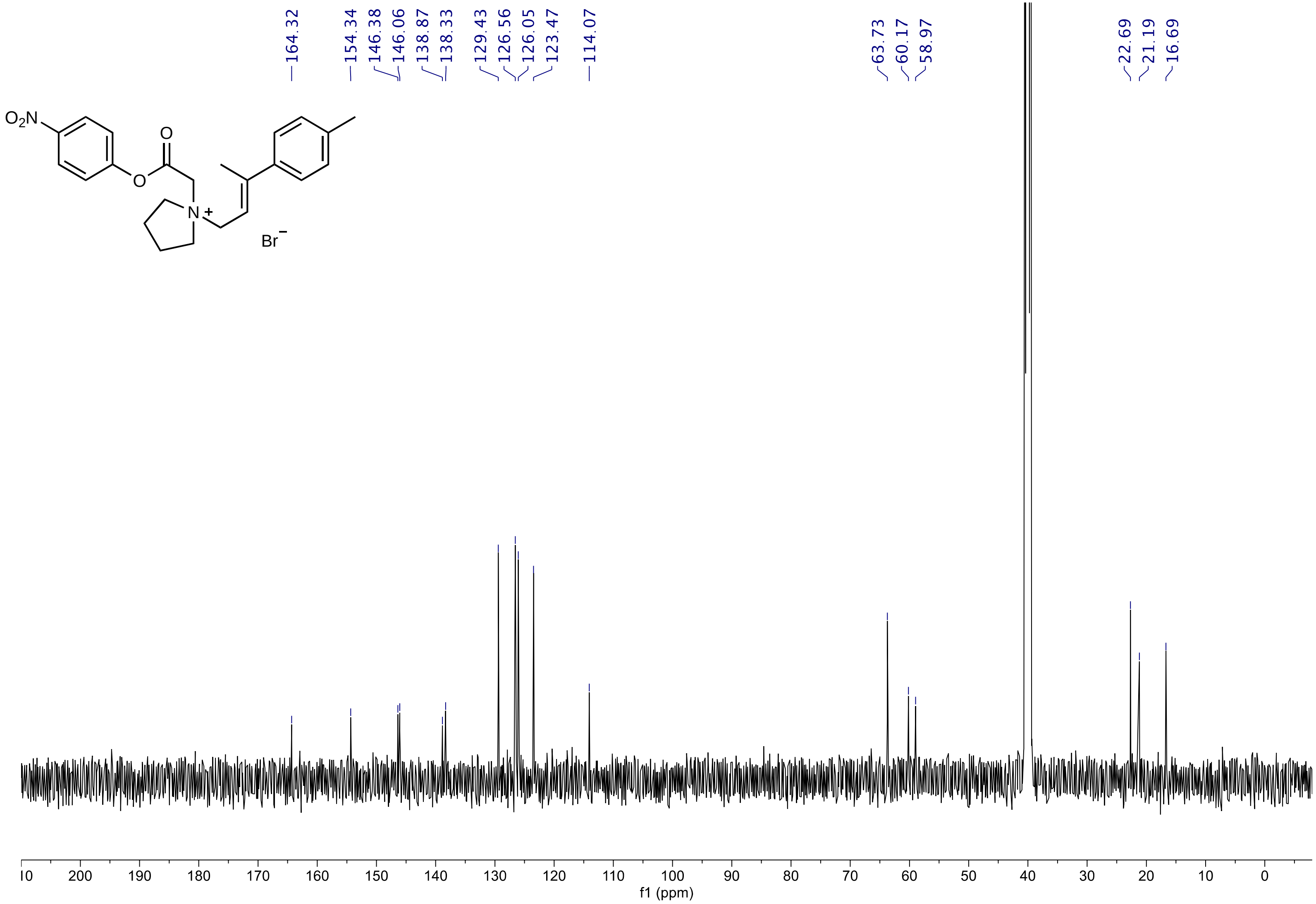

2l – ^{1}H NMR (400 MHz, d6-DMSO)

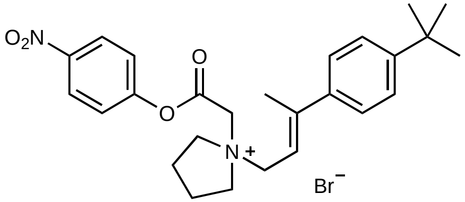

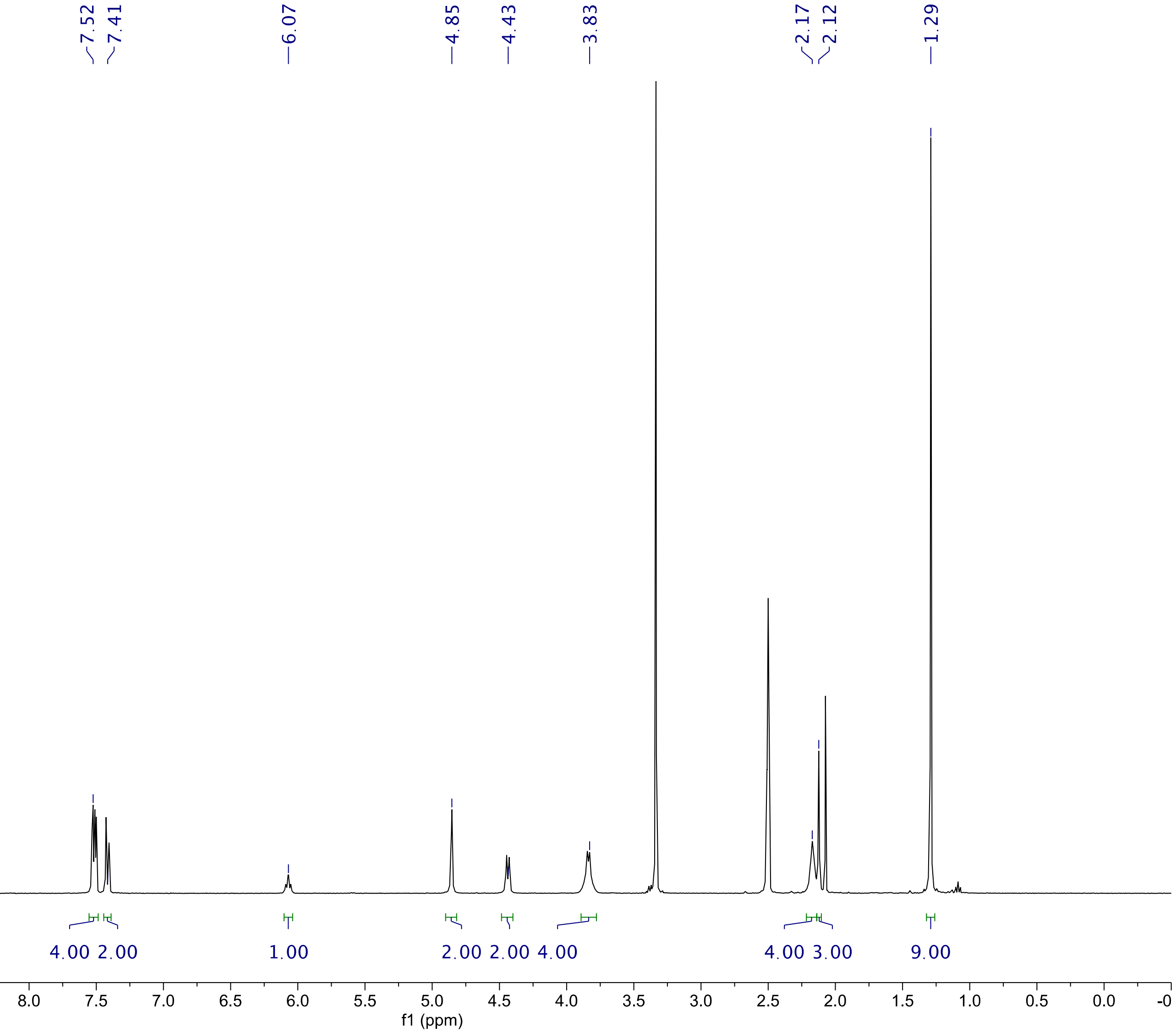

2l – ^{13}C NMR (126 MHz, d6-DMSO)

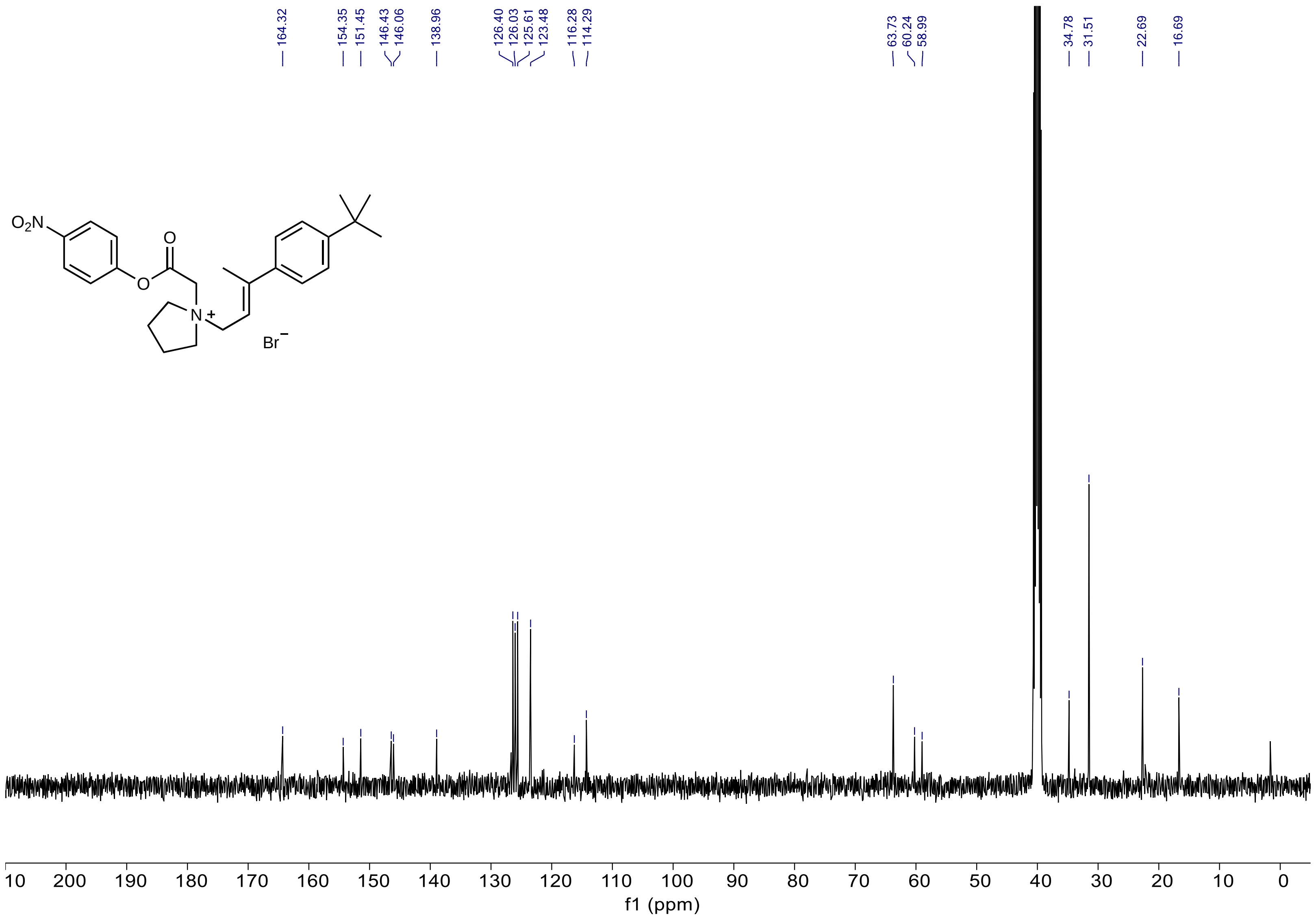

2m – ^{1}H NMR (400 MHz, d6-DMSO)

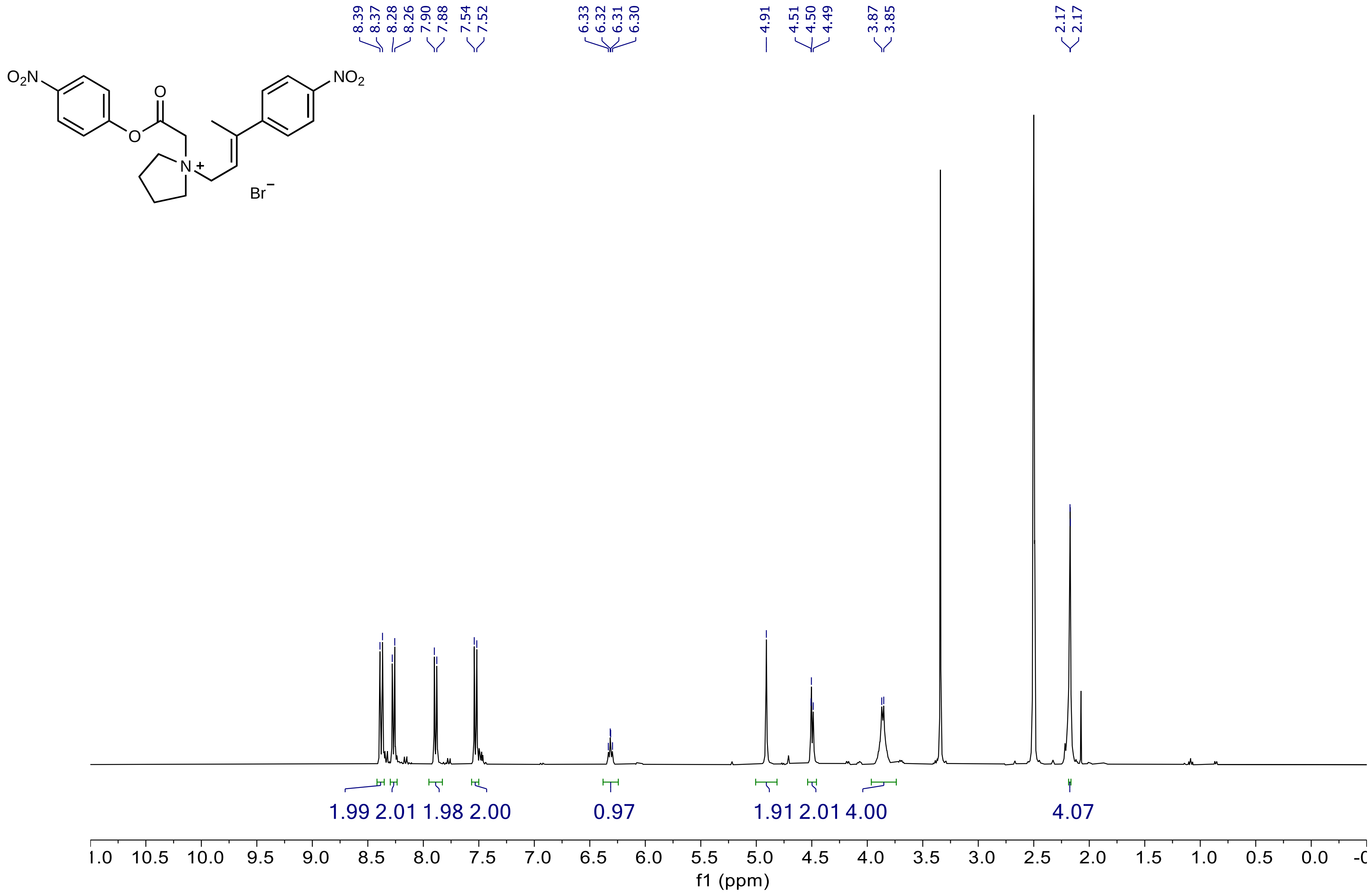

2m – ^{13}C NMR (126 MHz, d6-DMSO)

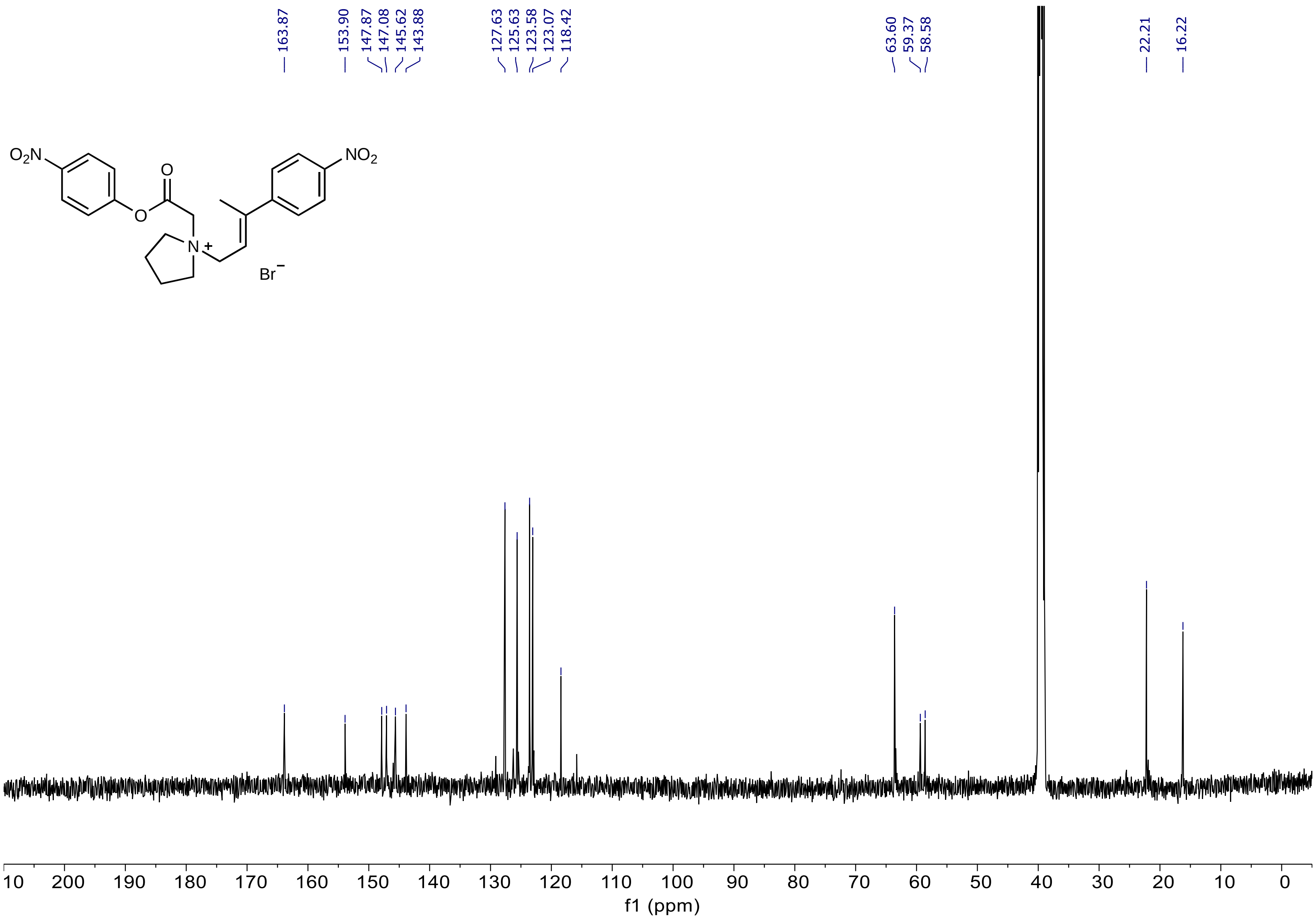

2n – ^{1}H NMR (500 MHz, d6-DMSO)

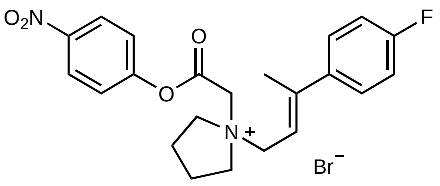

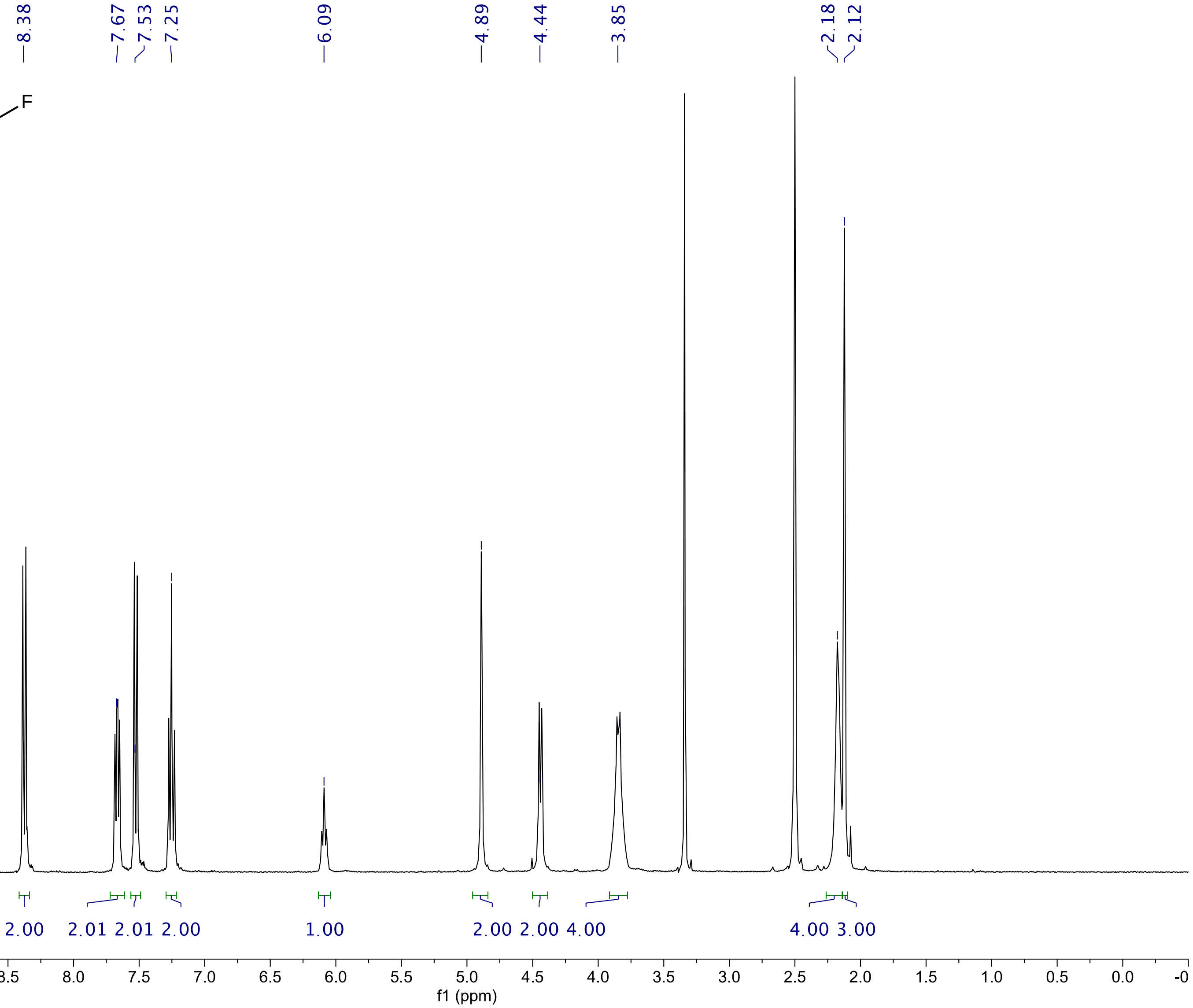

2n – ^{13}C NMR (126 MHz, d6-DMSO)

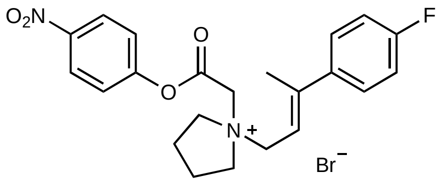

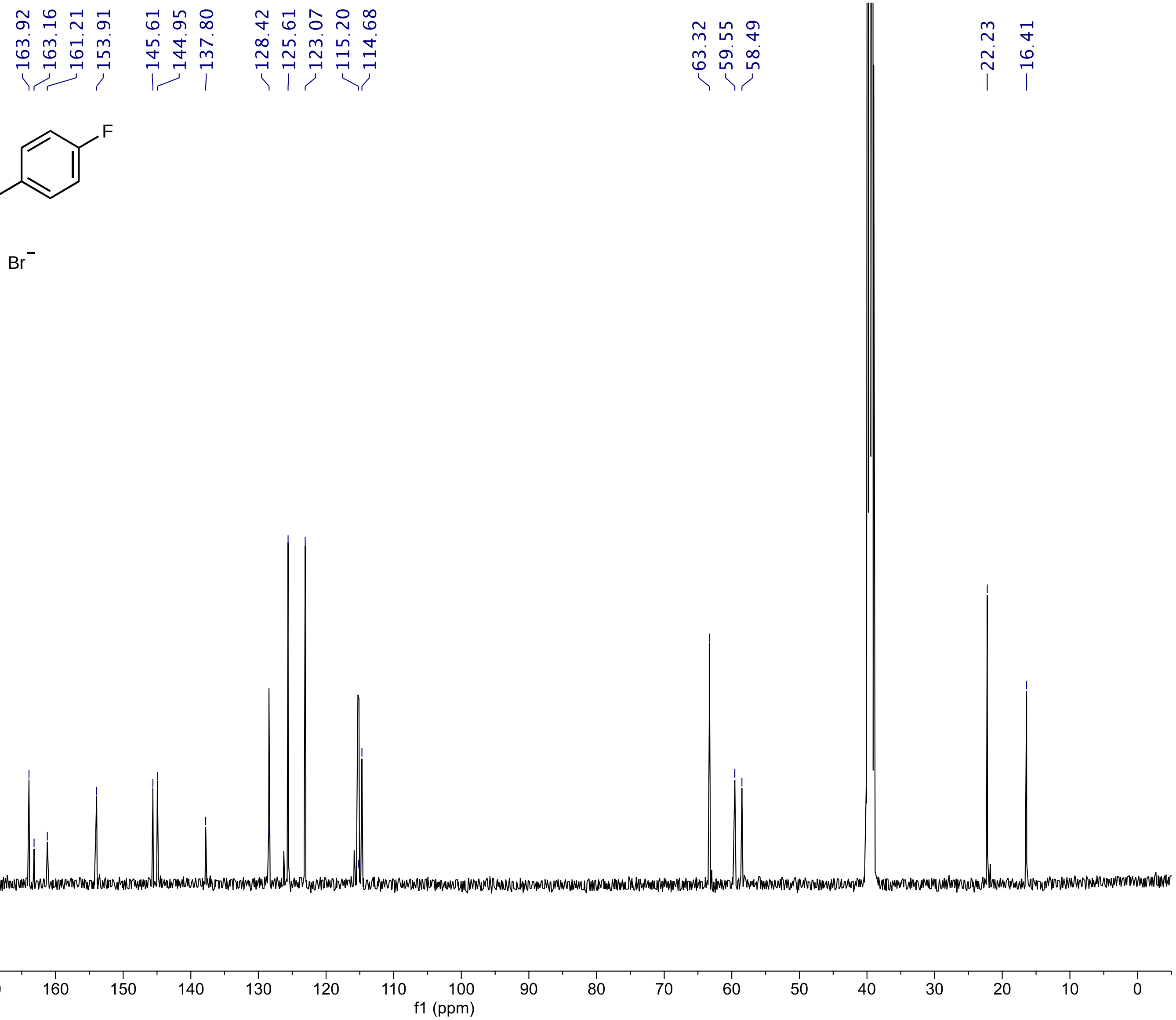

2n – ^{19}F NMR (377 MHz, d6-DMSO)

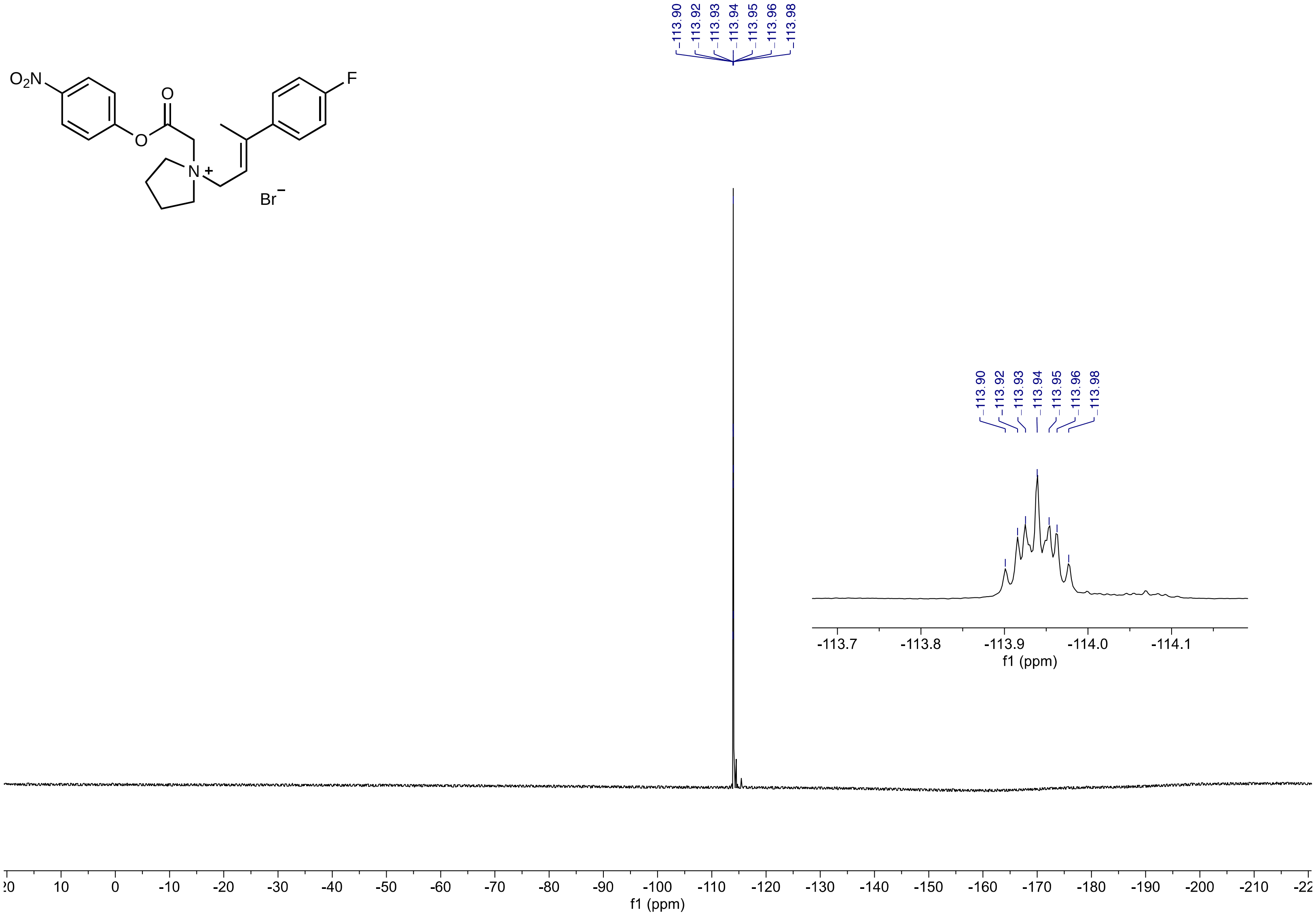

2o – ^{1}H NMR (500 MHz, d6-DMSO)

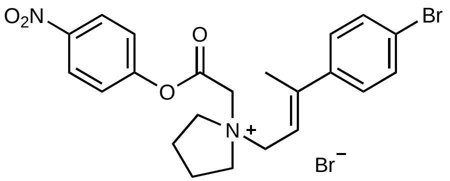

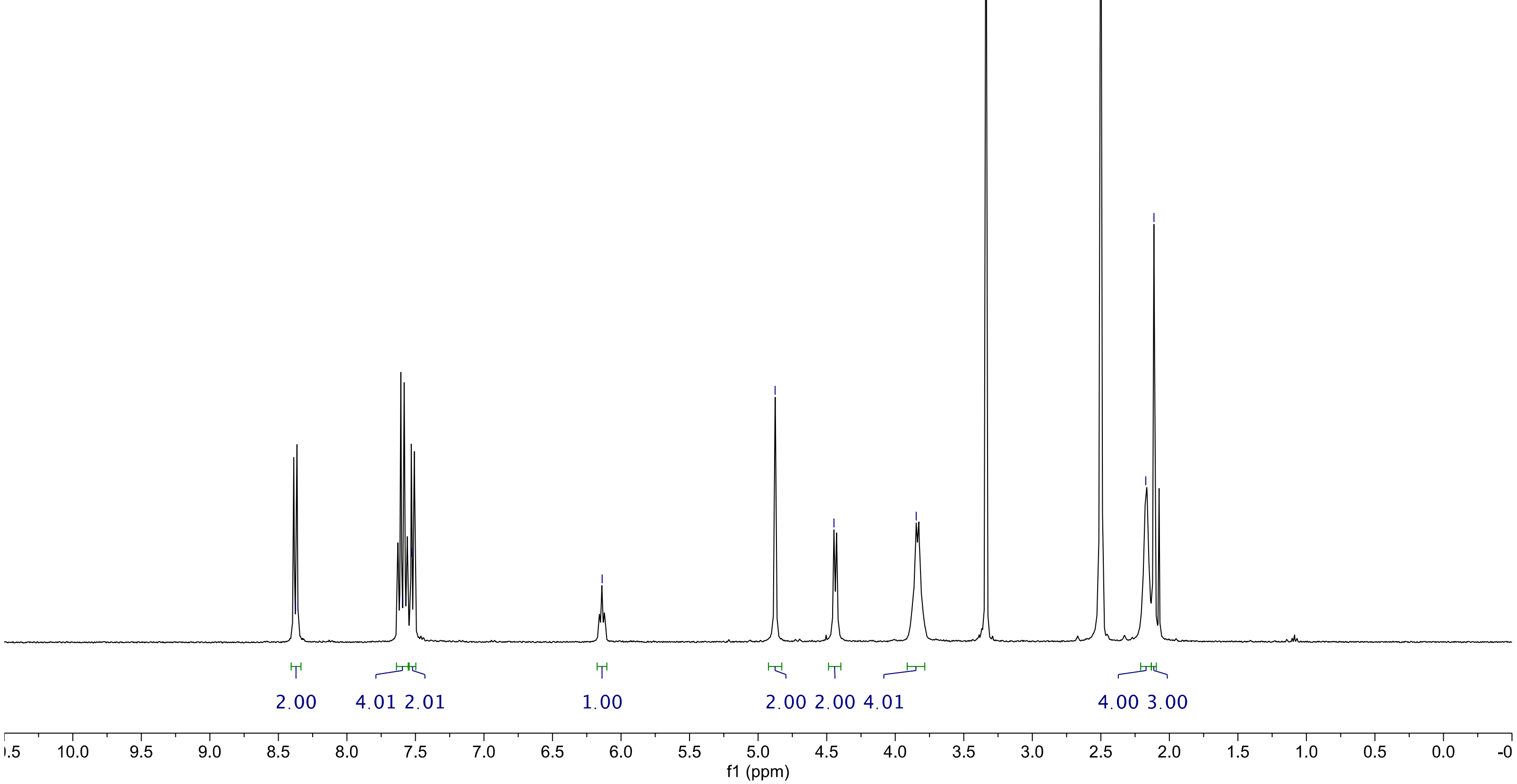

2o – ^{13}C NMR (126 MHz, d6-DMSO)

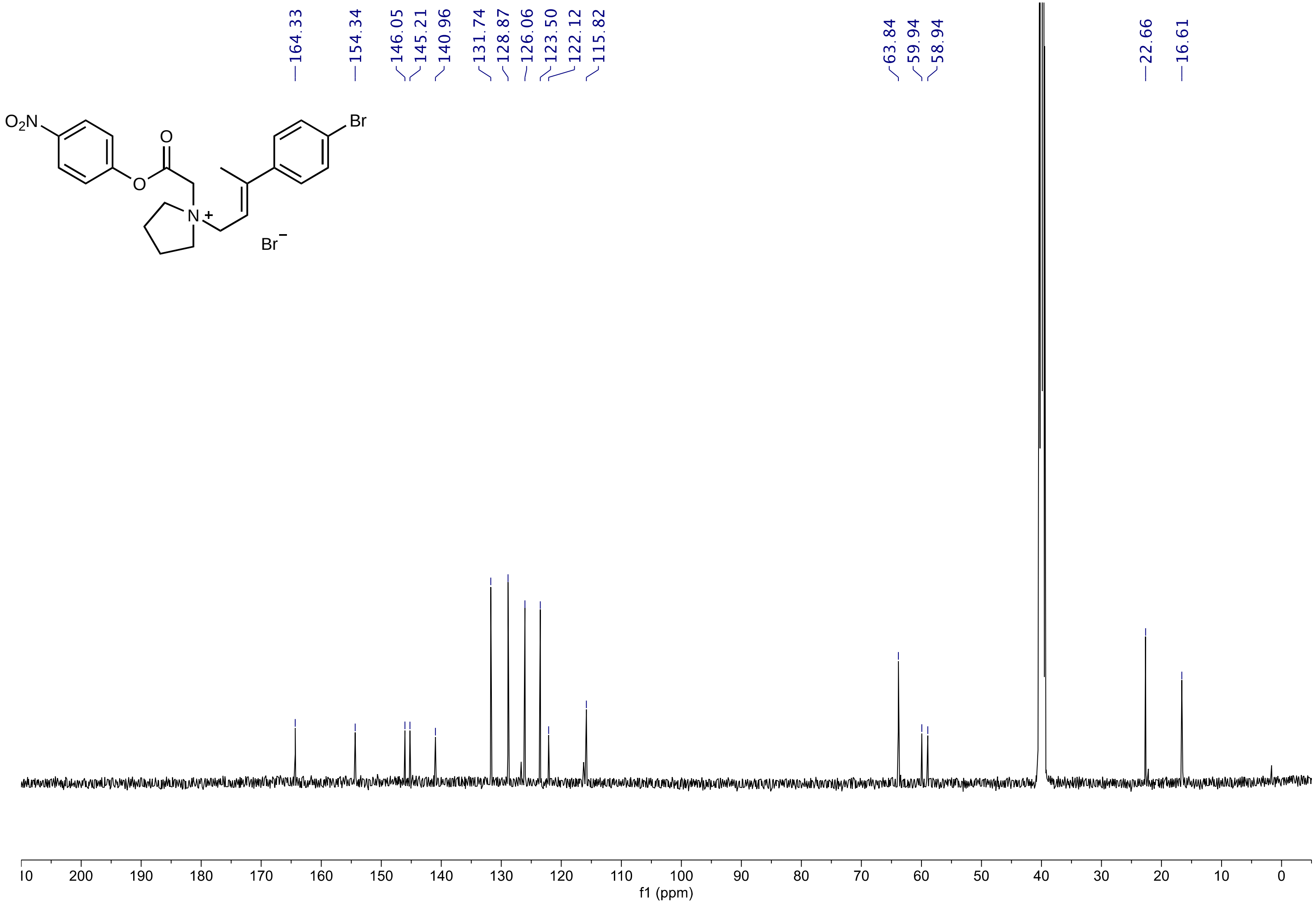

2p – ^{1}H NMR (500 MHz, d6-DMSO)

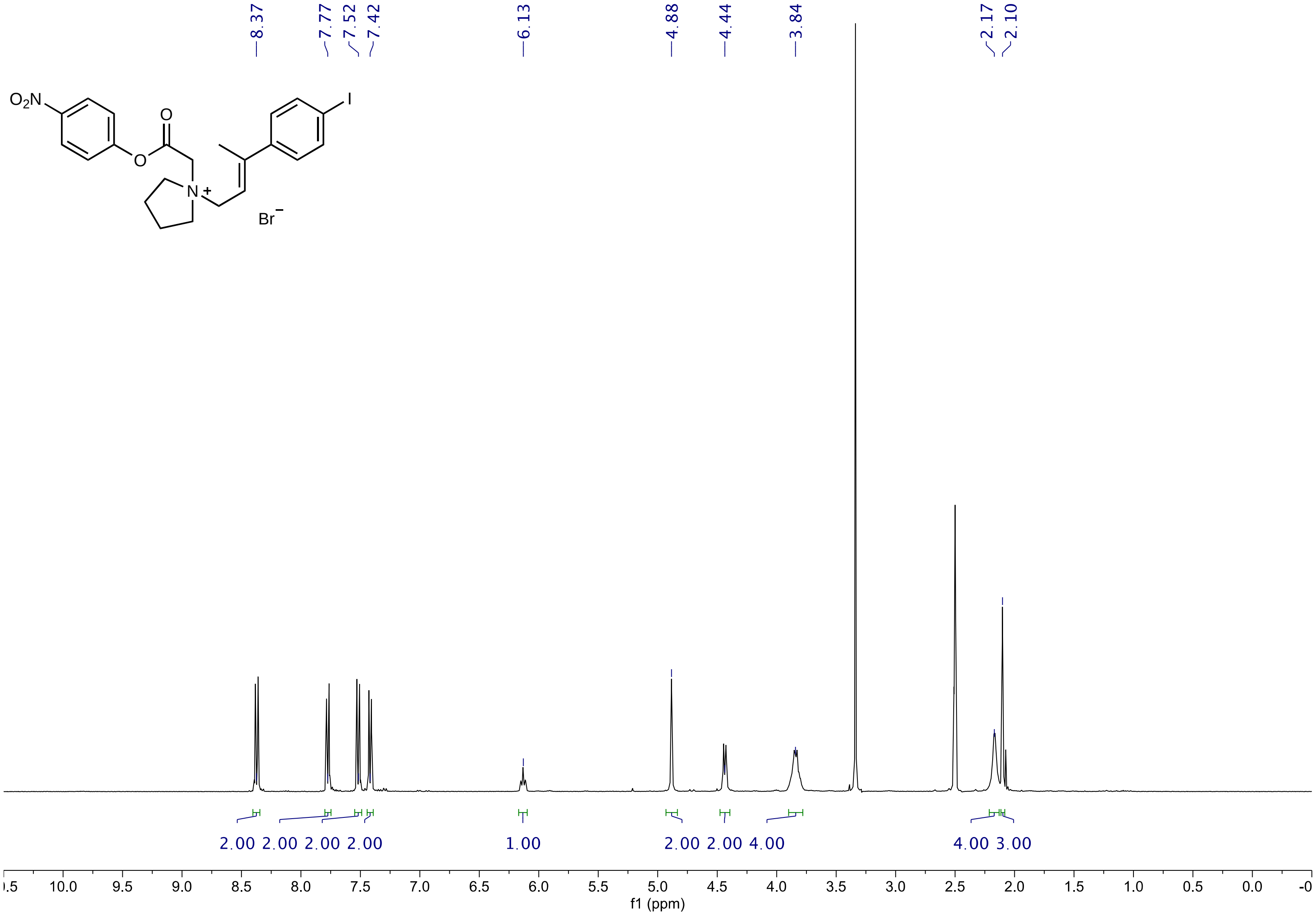

2p – ^{13}C NMR (126 MHz, d6-DMSO)

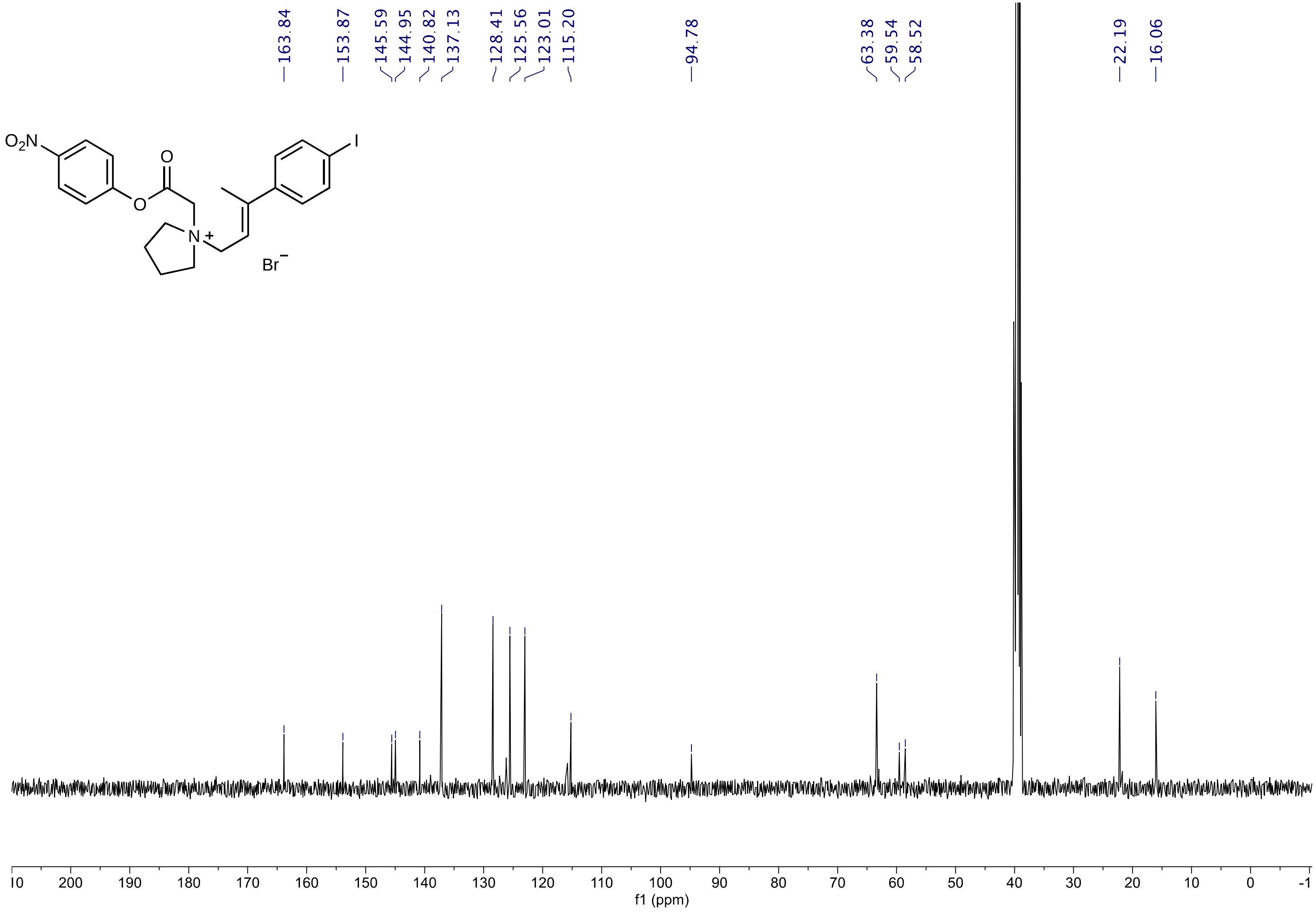

2r – ^{1}H NMR (500 MHz, d6-DMSO)

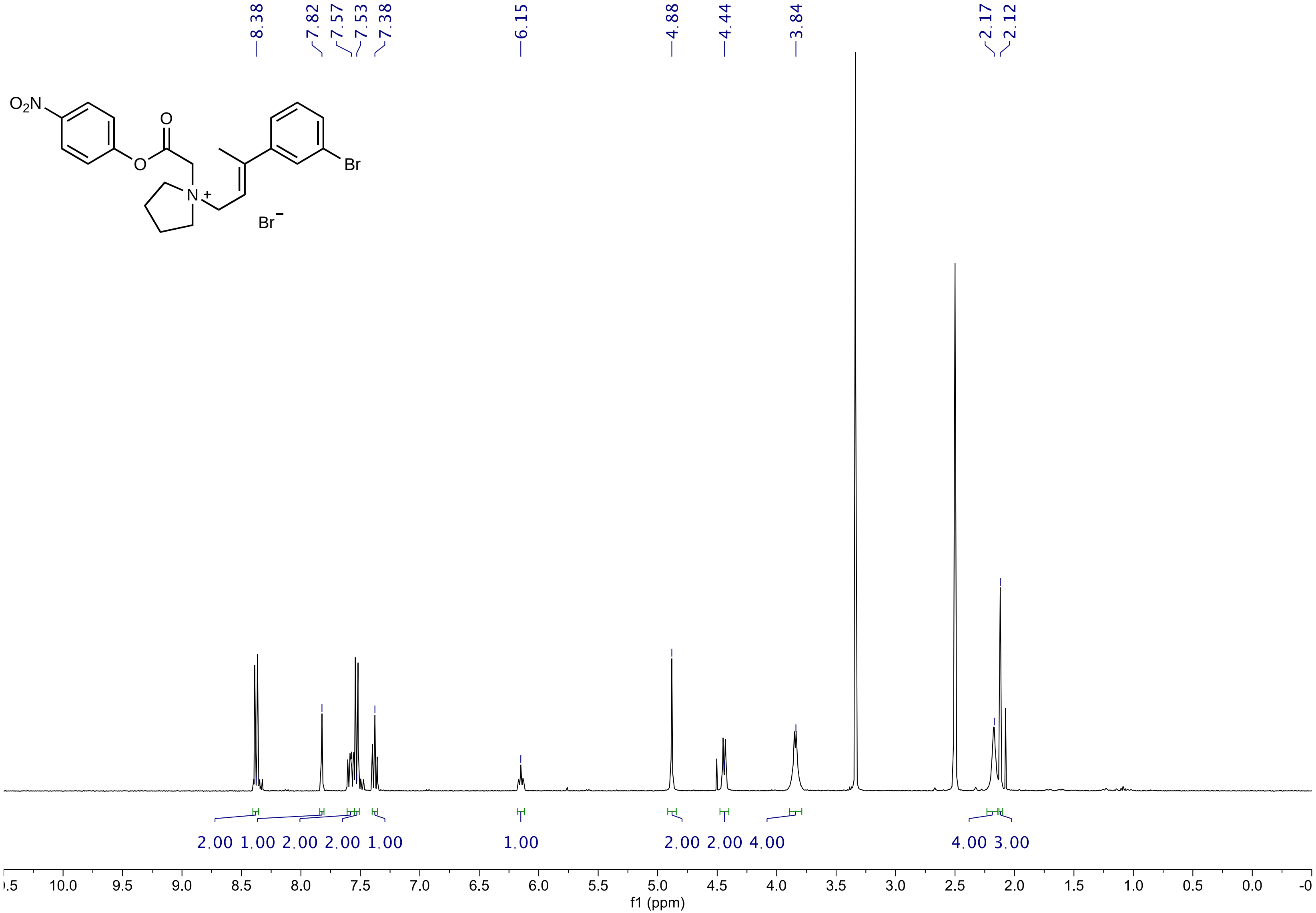

2r – ^{13}C NMR (126 MHz, d6-DMSO)

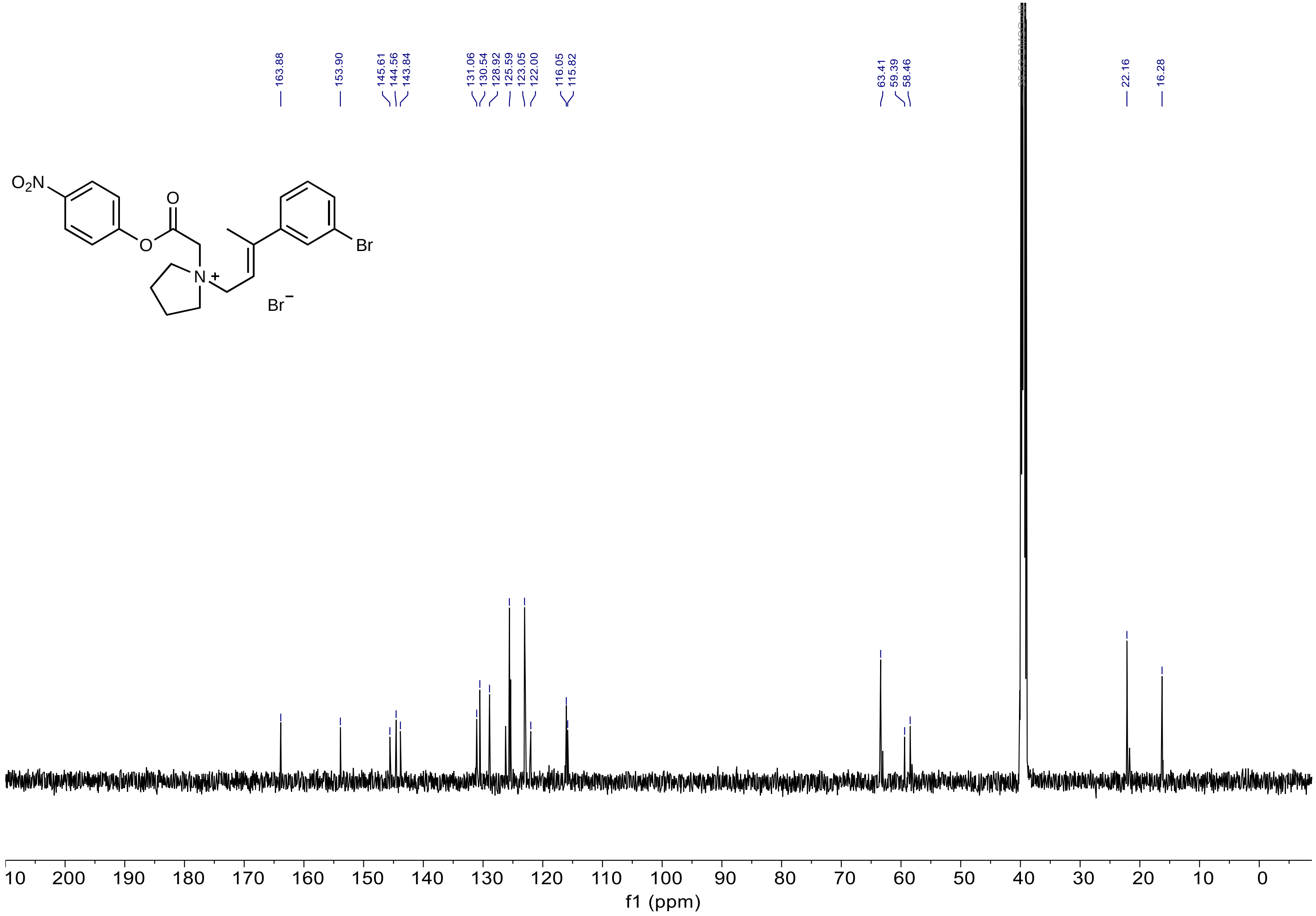

2q – ^{1}H NMR (500 MHz, d6-DMSO)

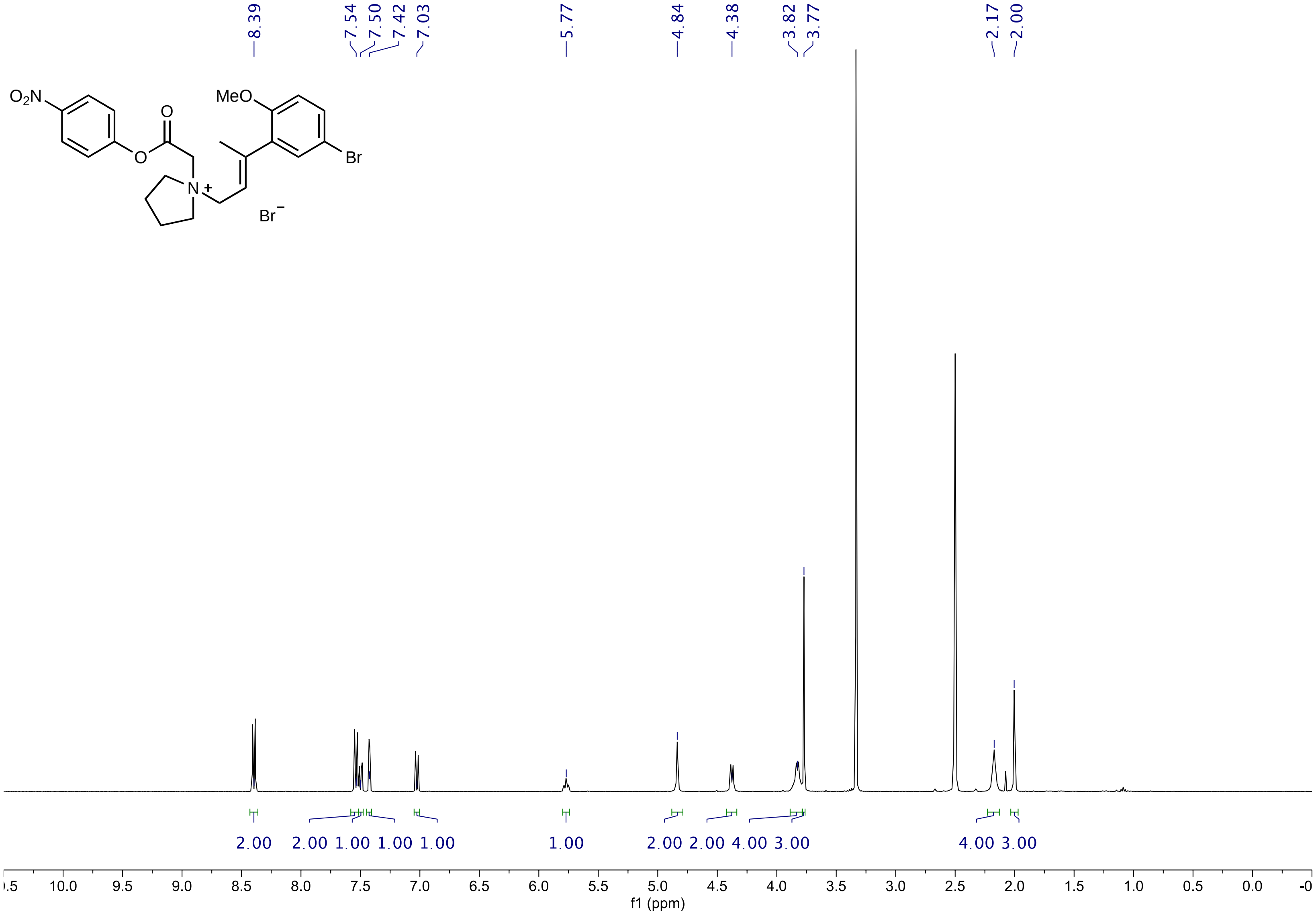

2q – ^{13}C NMR (126 MHz, d6-DMSO)

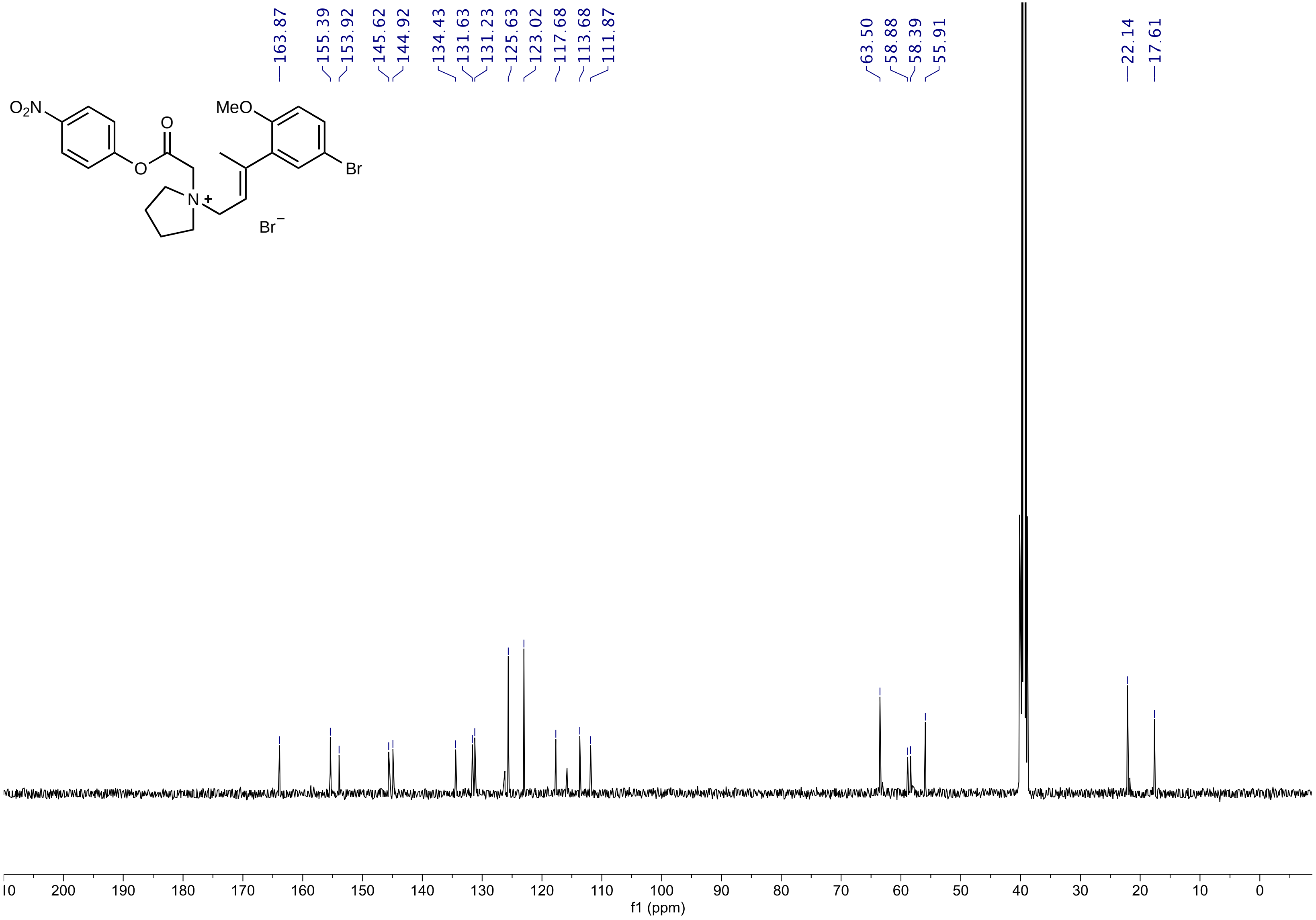

2w – ^{1}H NMR (400 MHz, d6-DMSO)

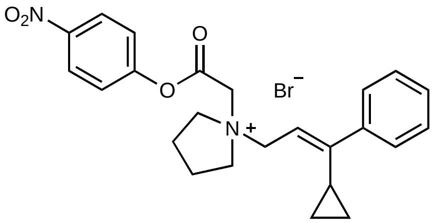

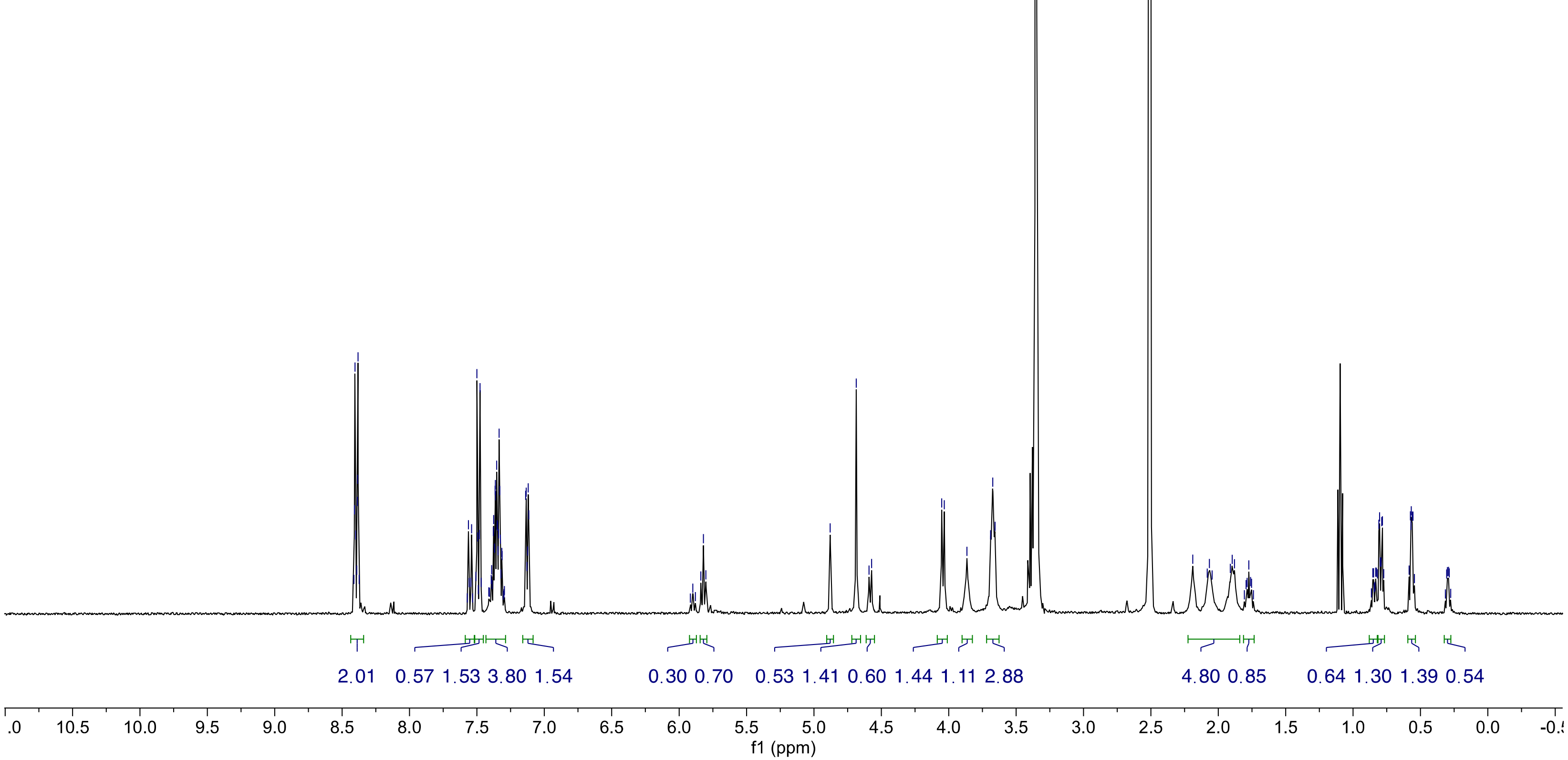

3a – ^{1}H NMR (500 MHz, $CDCl_3$)

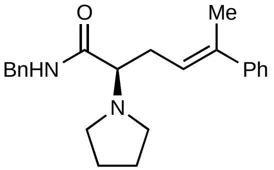

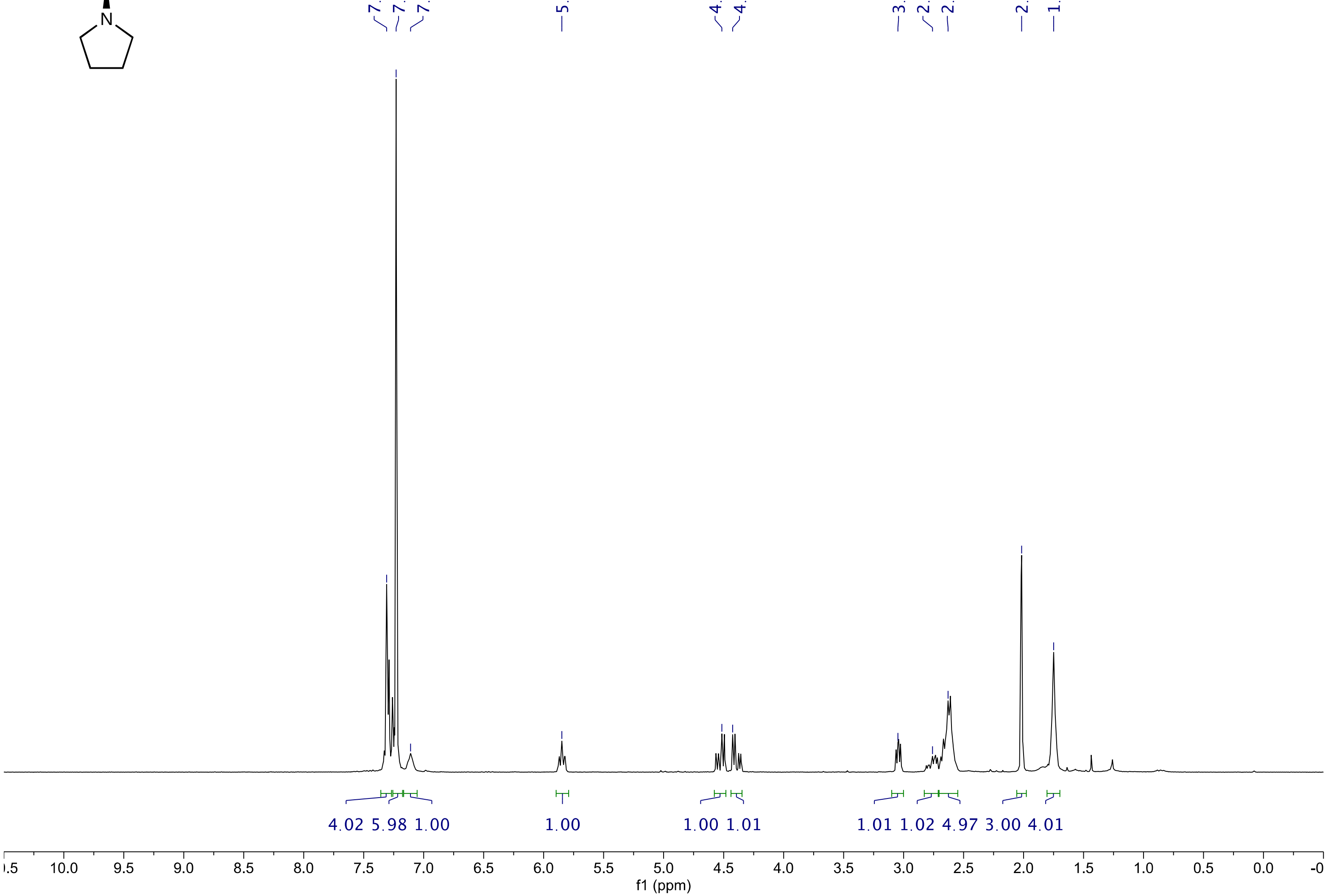

3a – ^{13}C NMR (126 MHz, $CDCl_3$)

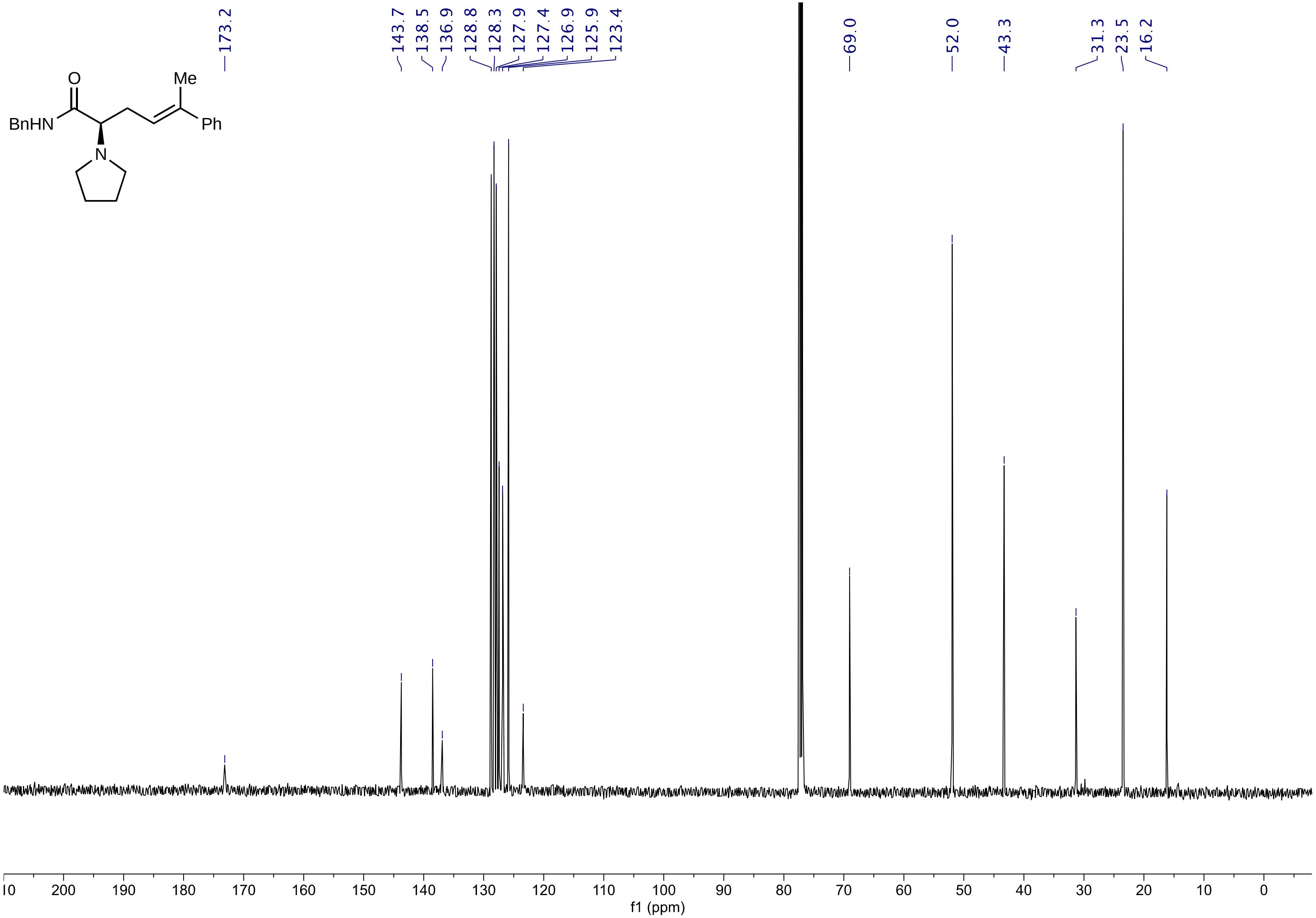

syn-**4a** – ^{1}H NMR (500 MHz, $CDCl_3$)

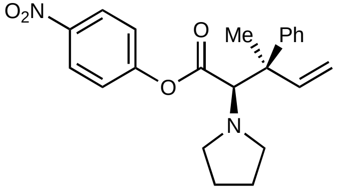

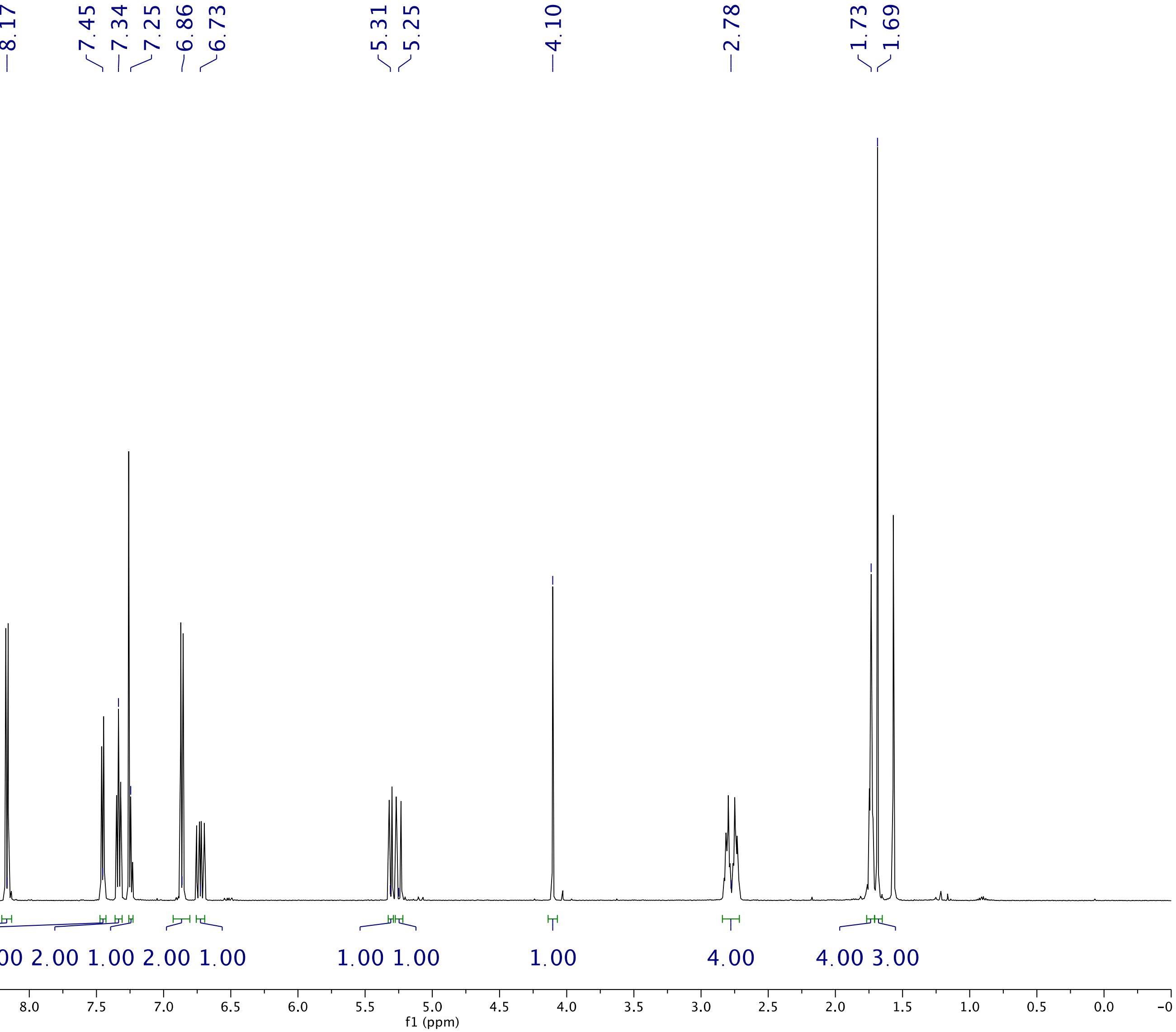

syn-**4a** – ^{13}C NMR (126 MHz, CDCl$_3$)

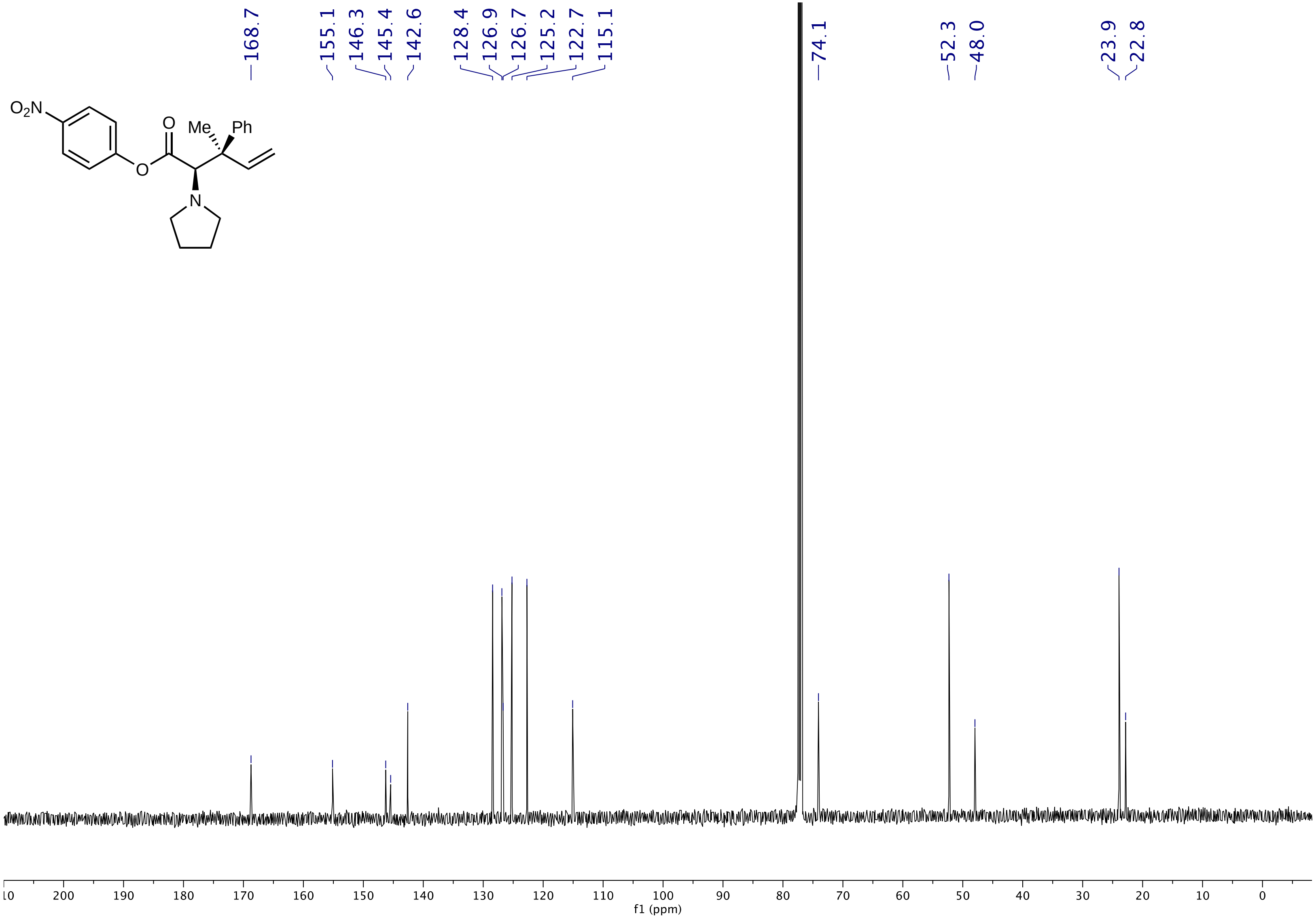

3b – ^{1}H NMR (500 MHz, $CDCl_3$)

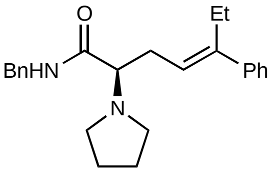

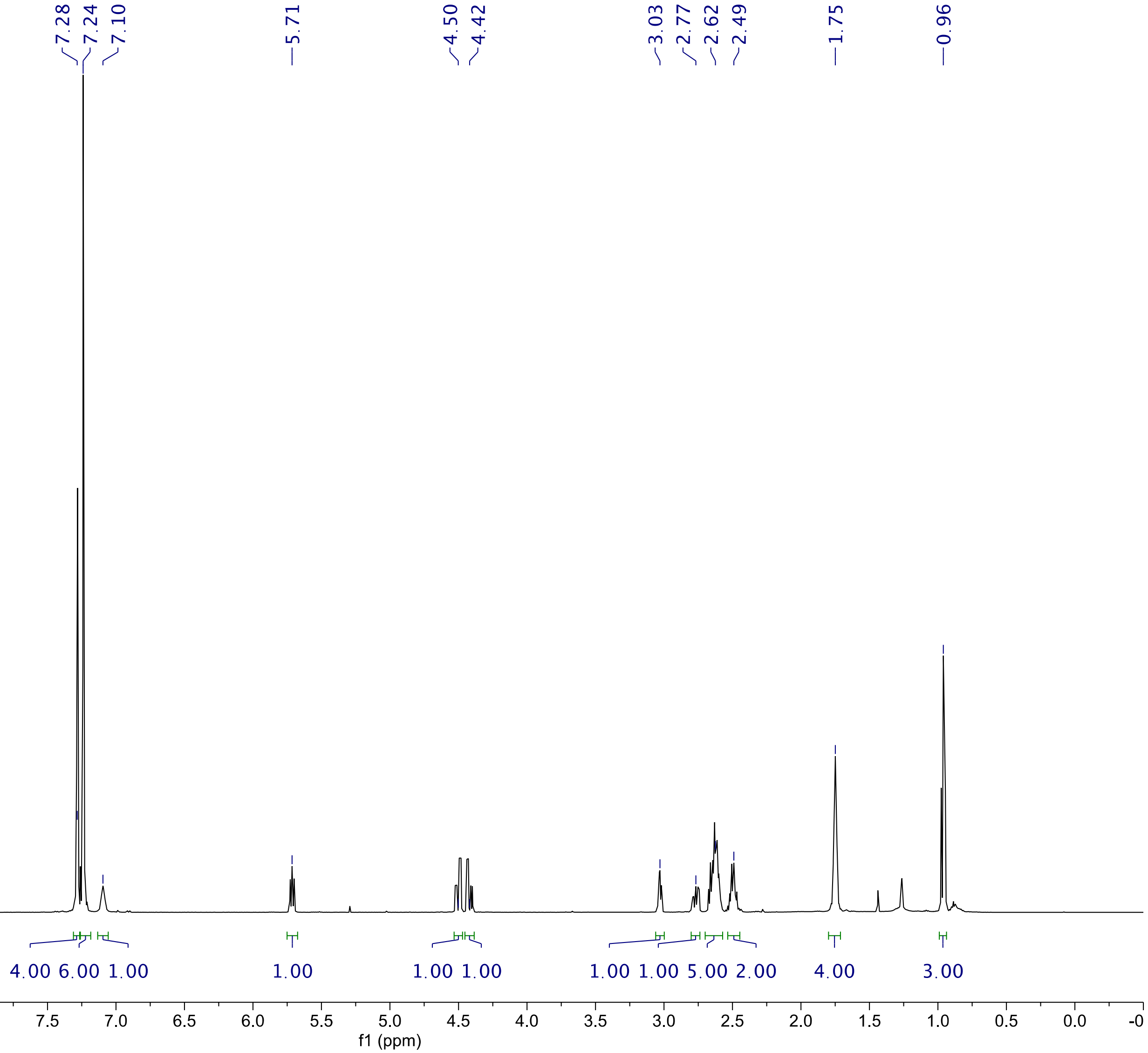

3b – ^{13}C NMR (126 MHz, $CDCl_3$)

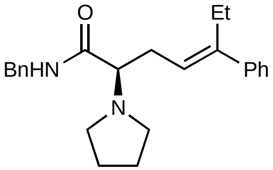

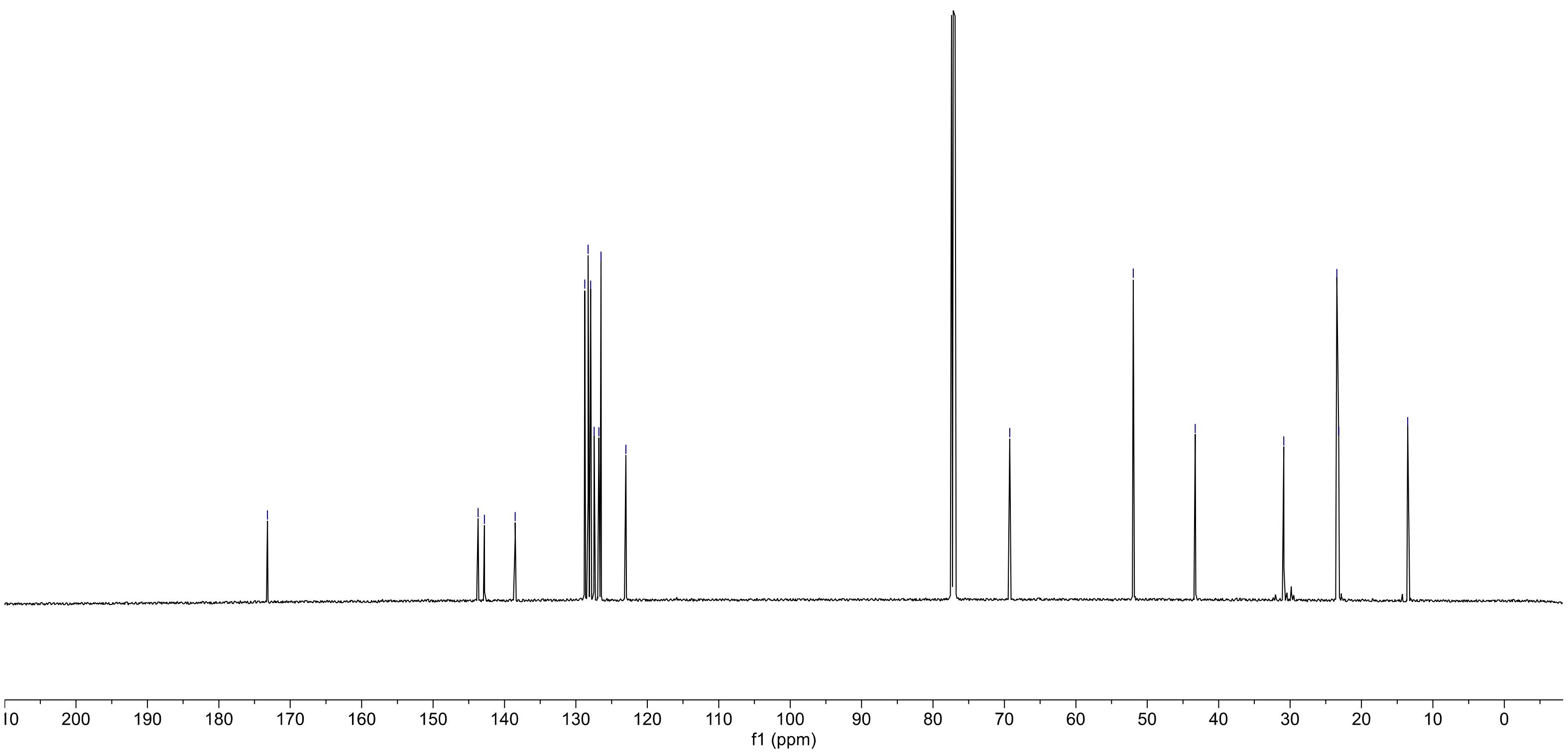

3c – ^{1}H NMR (400 MHz, CD_2Cl_2)

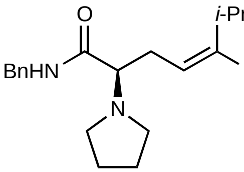

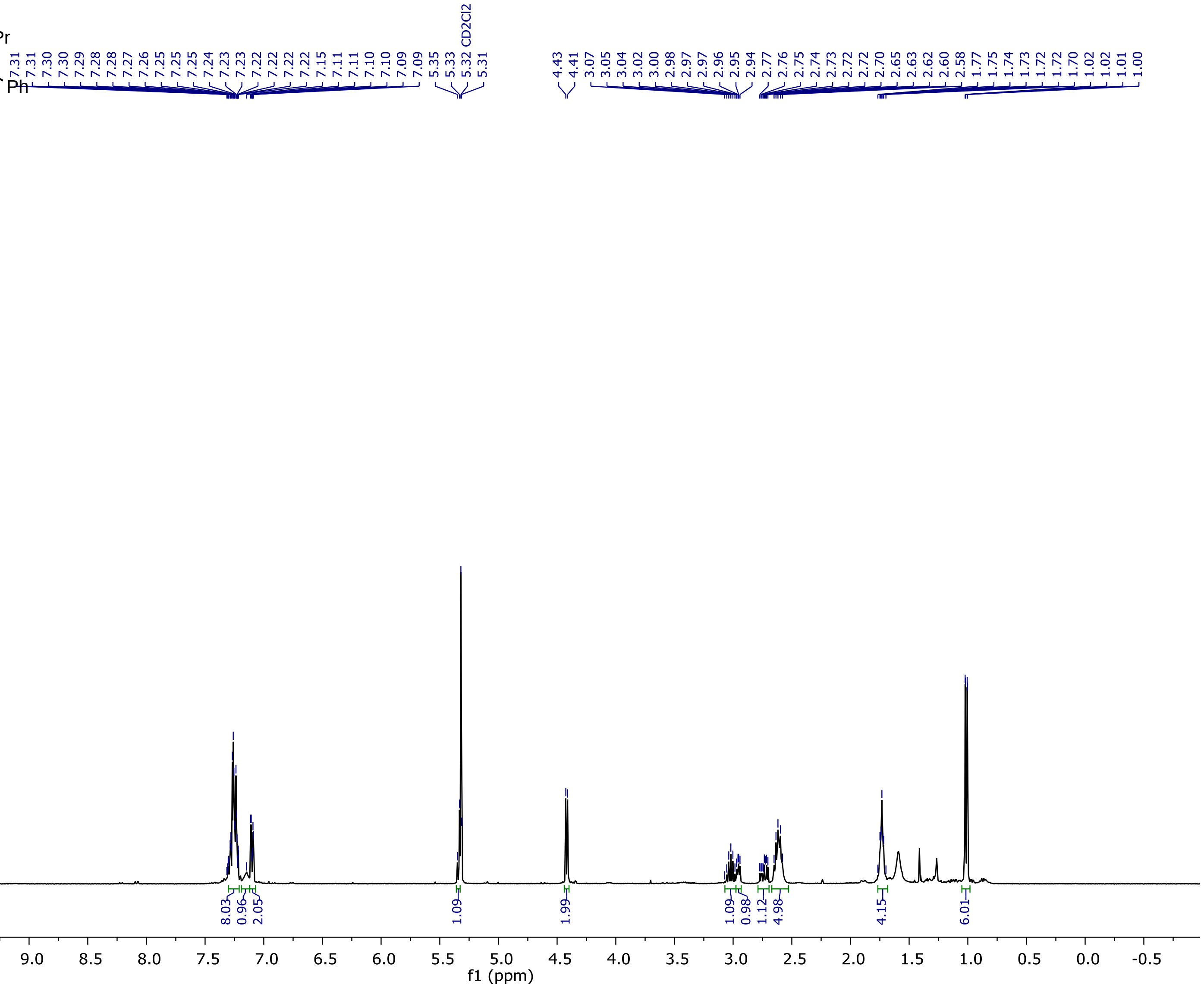

3c – ^{13}C NMR (101 MHz, CD_2Cl_2)

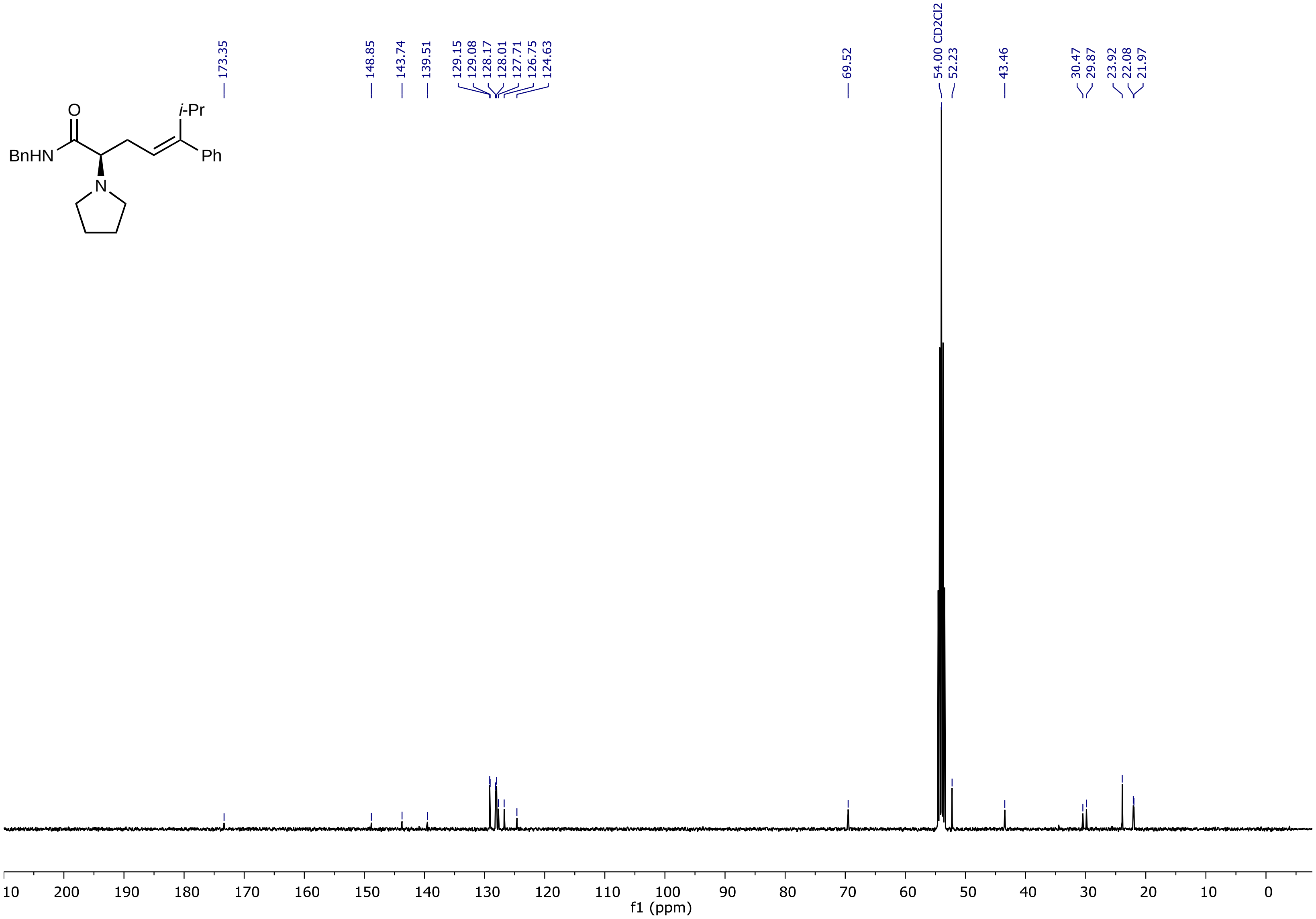

3d – ^{1}H NMR (500 MHz, $CDCl_3$)

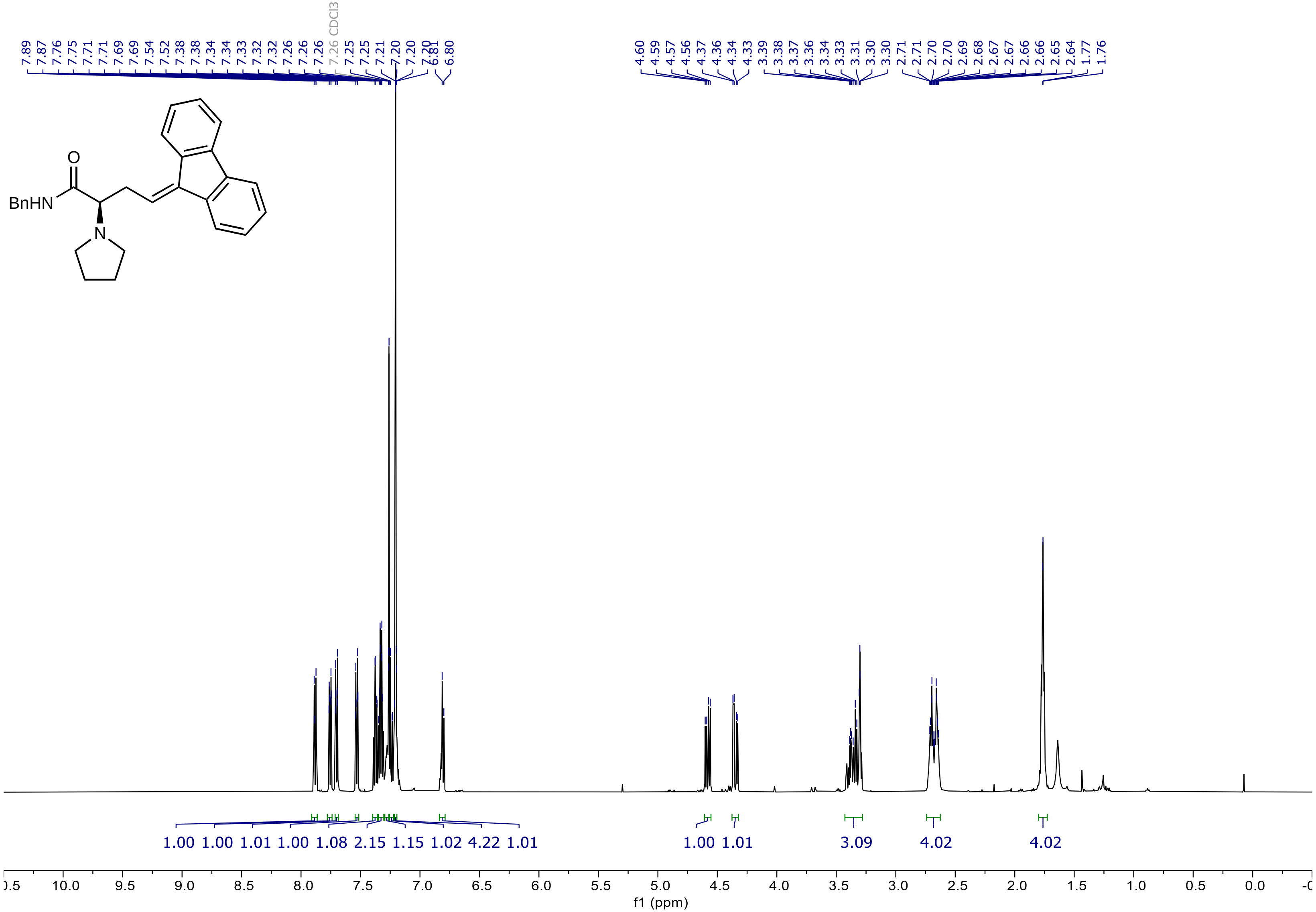

3d – ^{13}C NMR (126 MHz, $CDCl_3$)

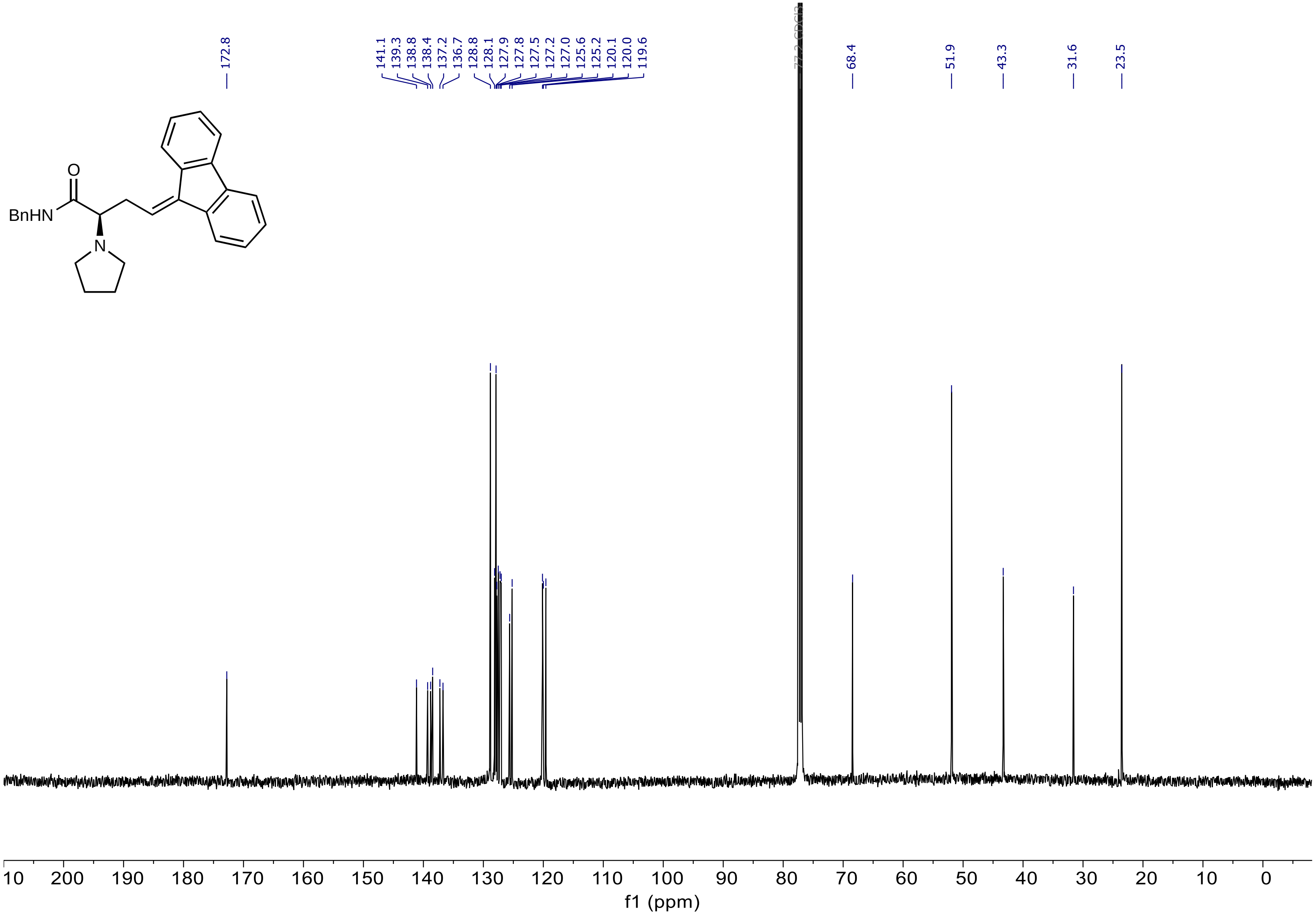

3e – ^{1}H NMR (500 MHz, $CDCl_3$)

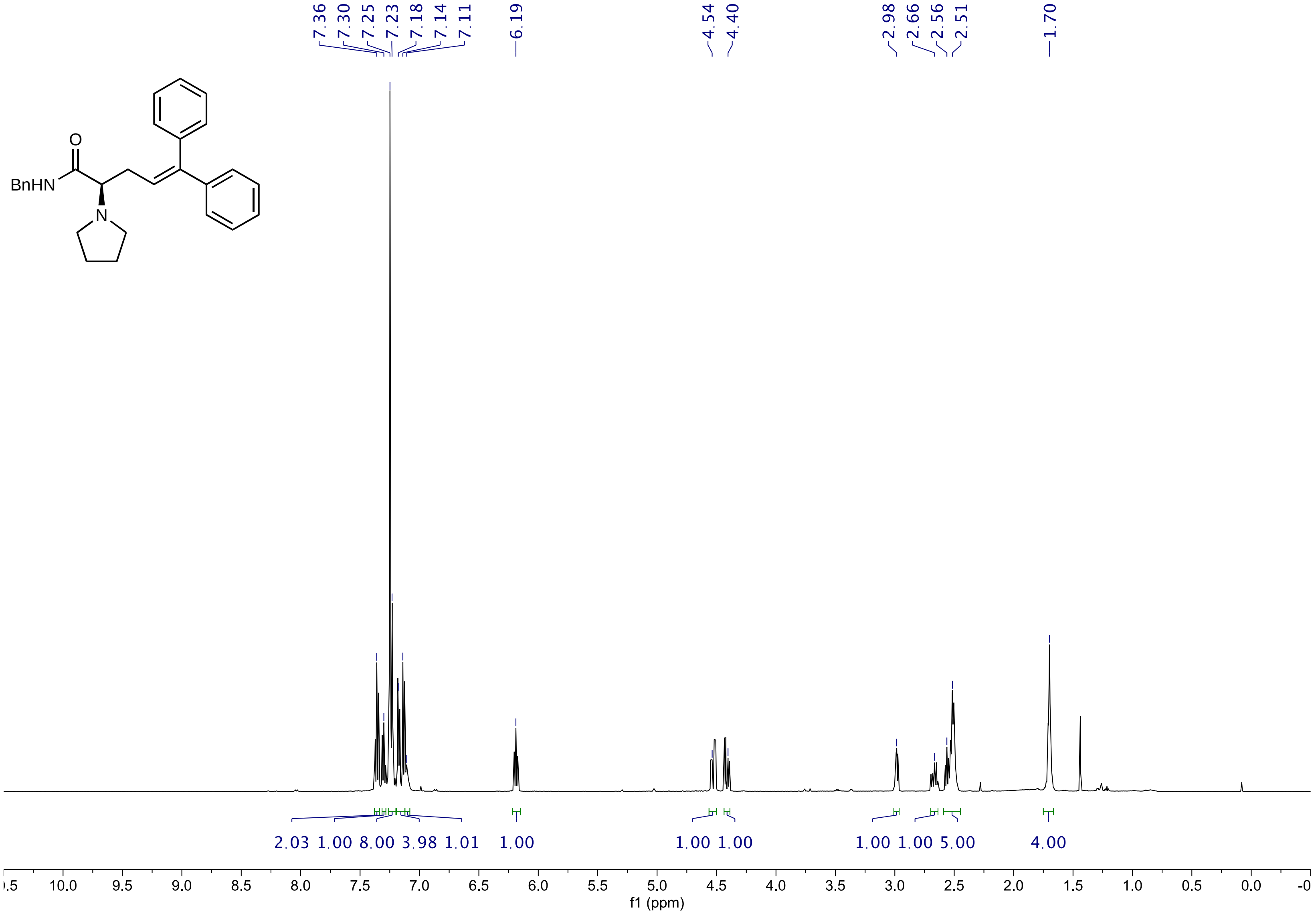

3e – ^{13}C NMR (126 MHz, CDCl$_3$)

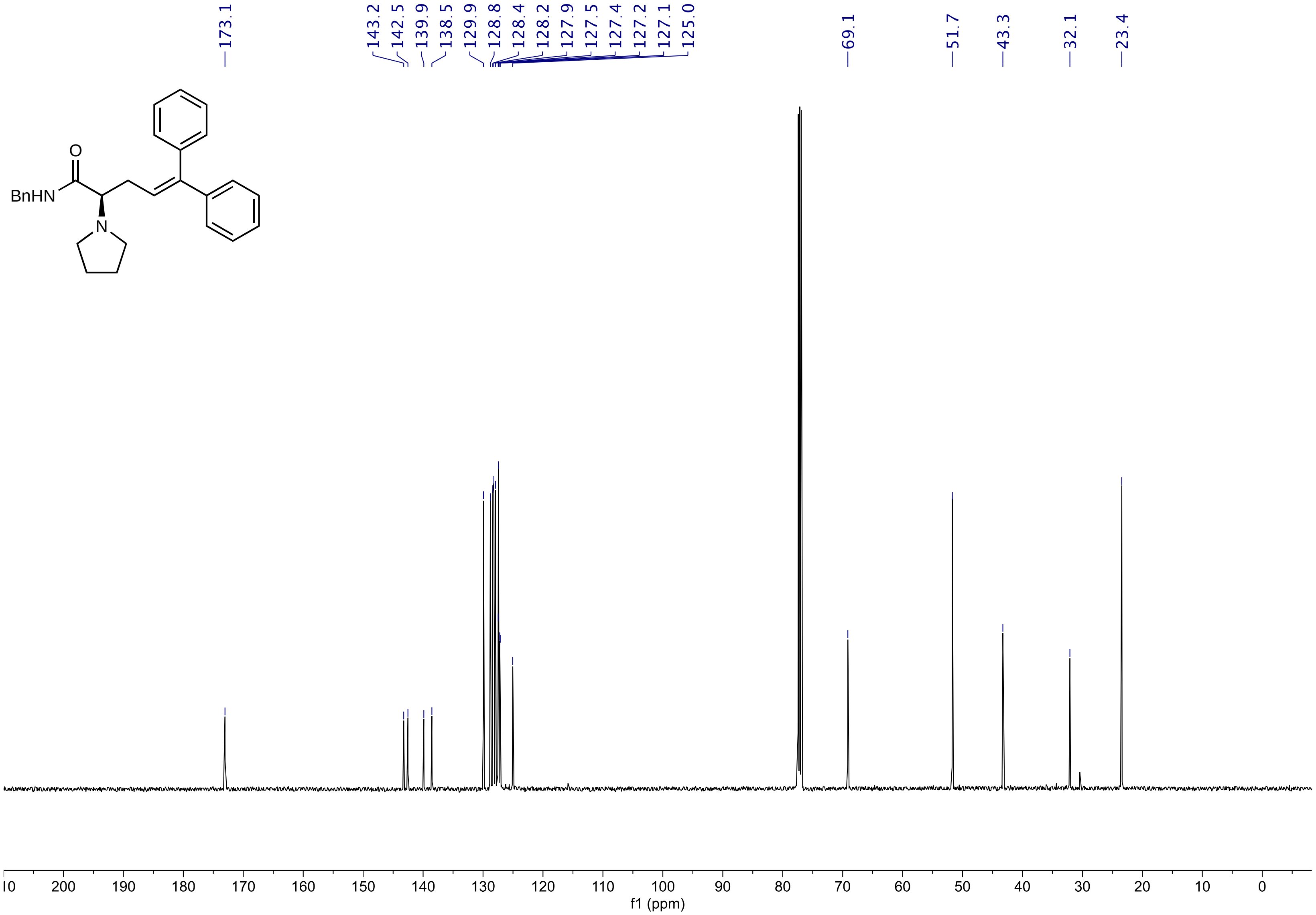

3f – 1H NMR (500 MHz, $CDCl_3$)

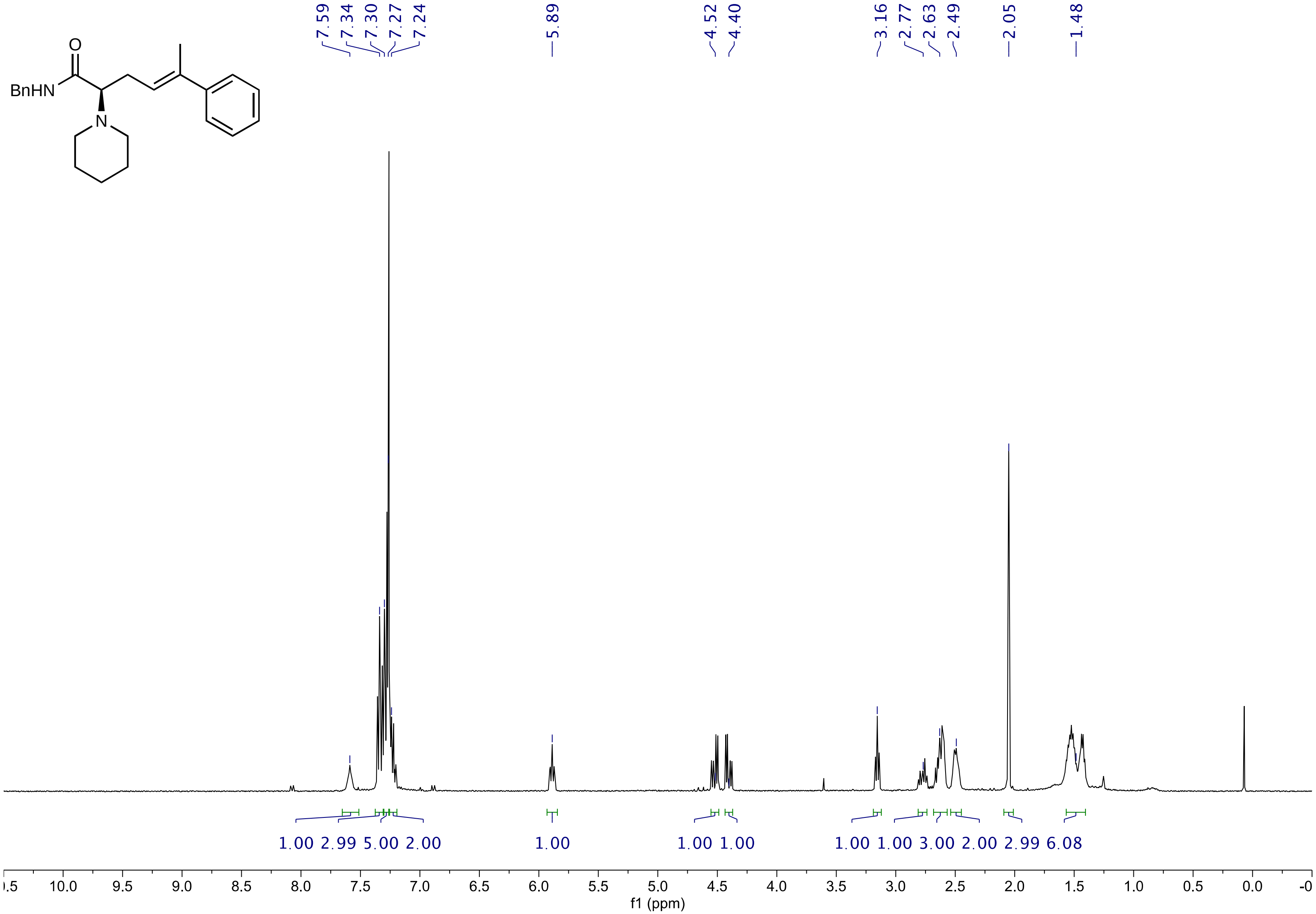

3f – ^{13}C NMR (126 MHz, $CDCl_3$)

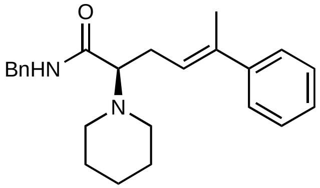

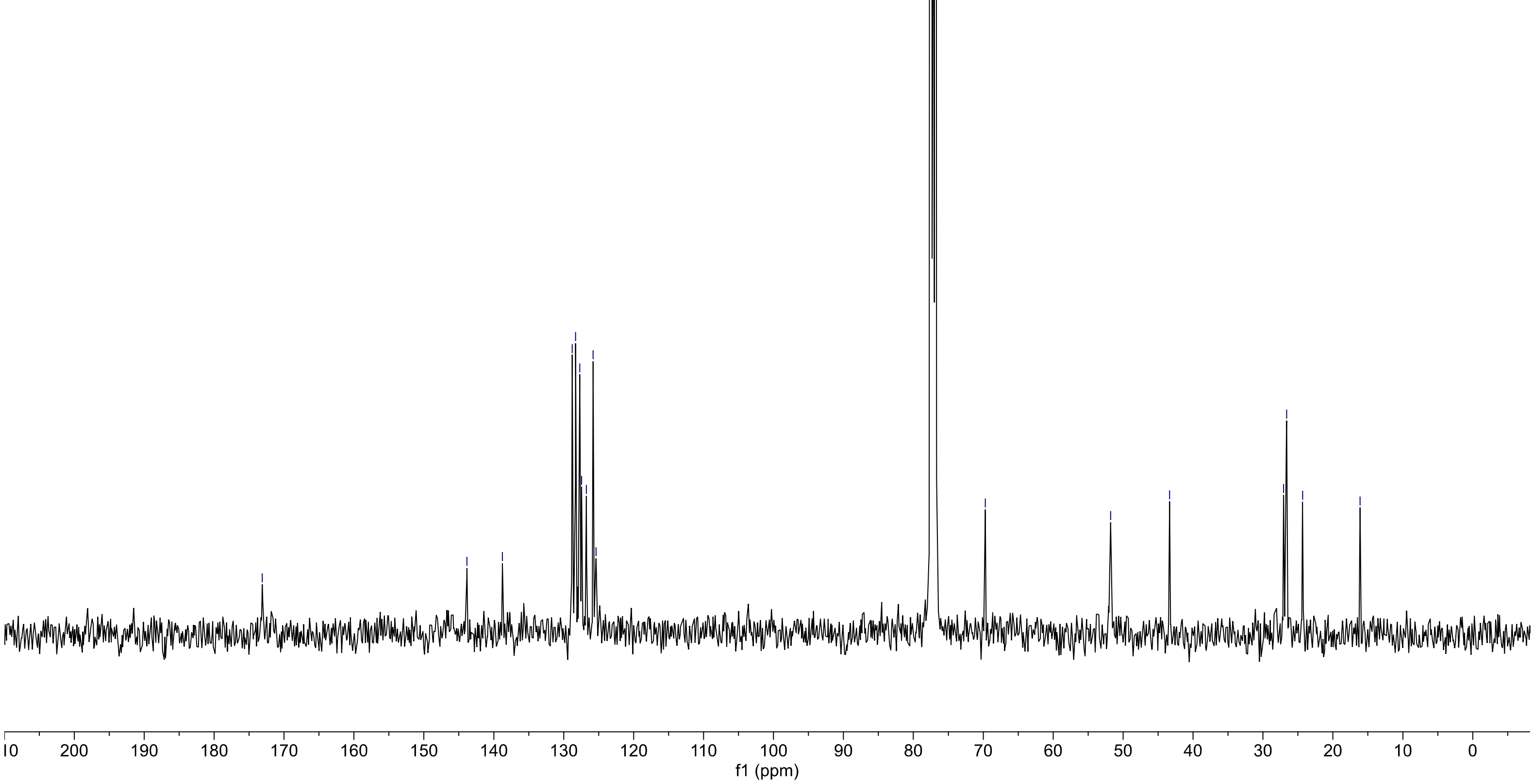

3g – ^{1}H NMR (500 MHz, $CDCl_3$)

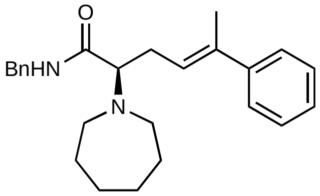

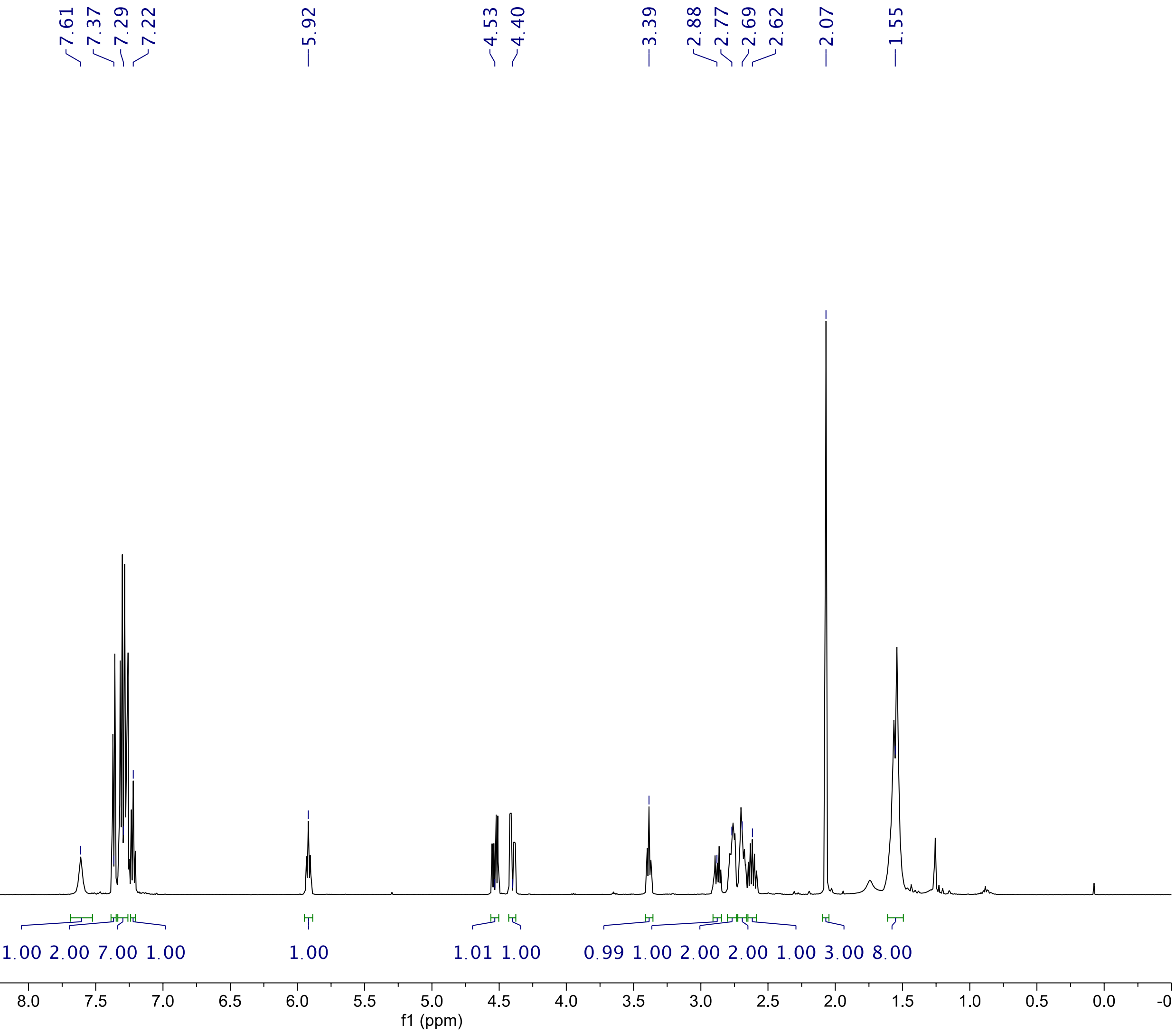

3g – ^{13}C NMR (126 MHz, $CDCl_3$)

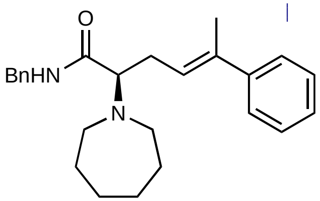

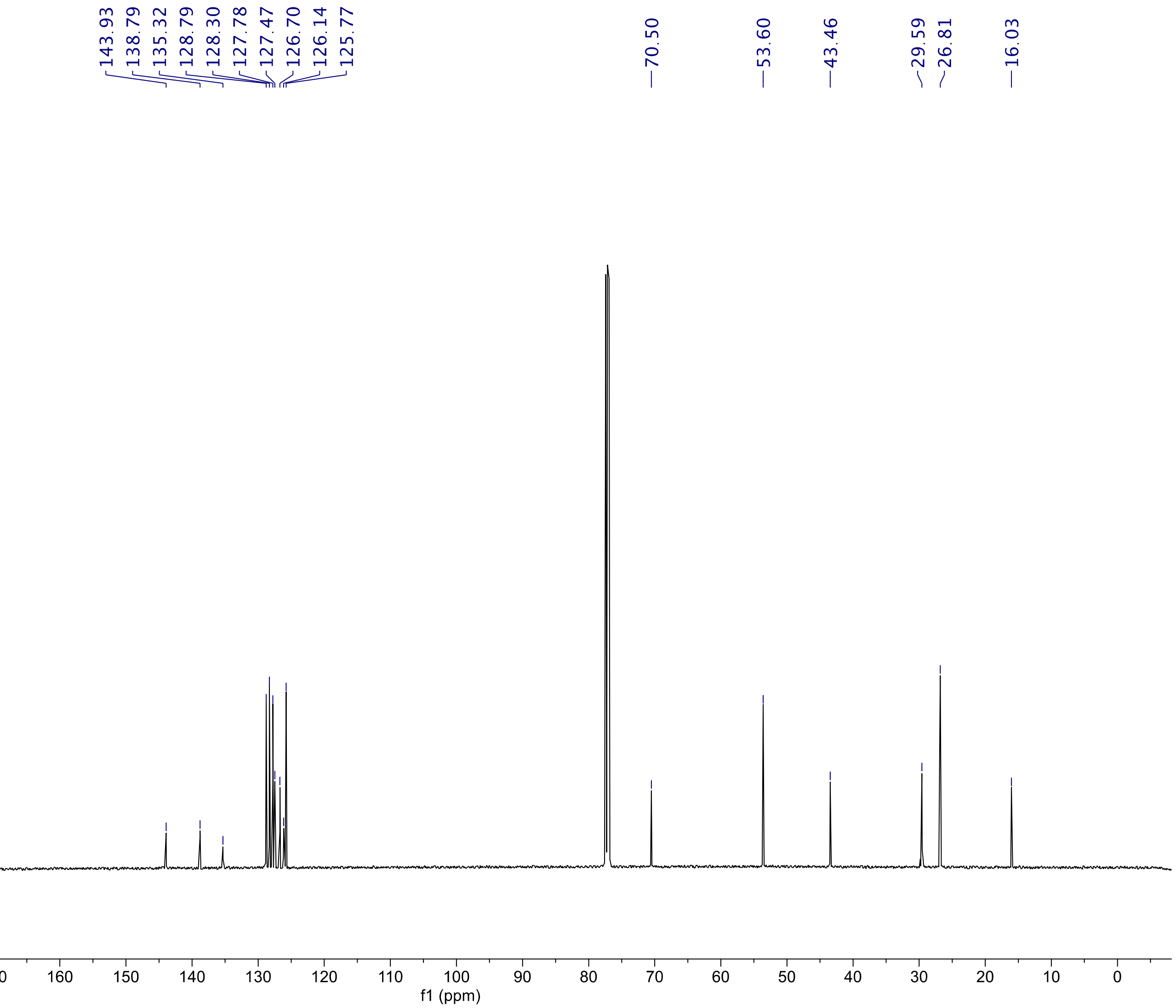

3h – ^{1}H NMR (500 MHz, $CDCl_3$)

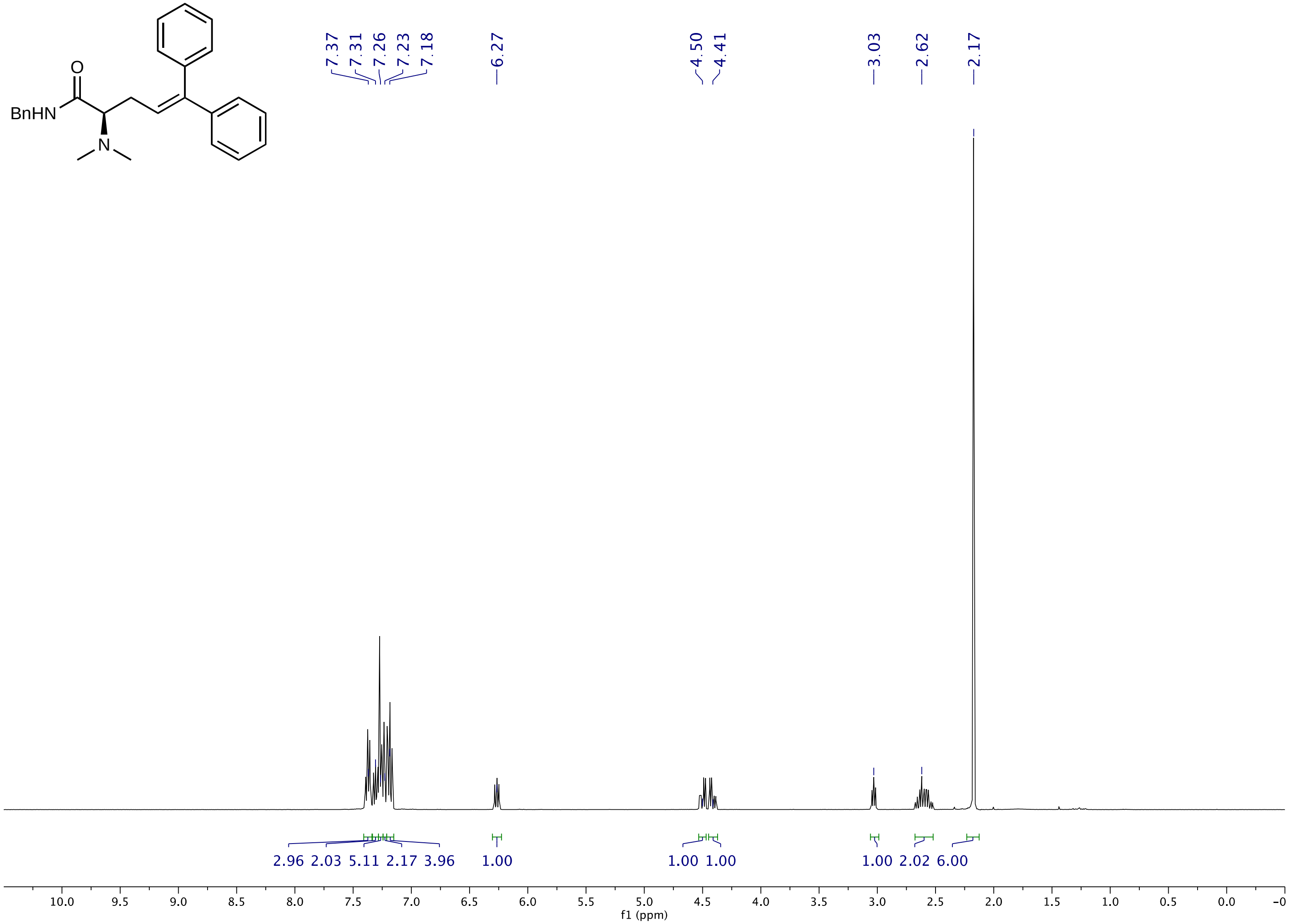

3h – ^{13}C NMR (126 MHz, $CDCl_3$)

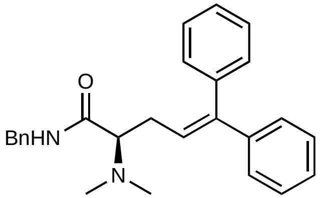

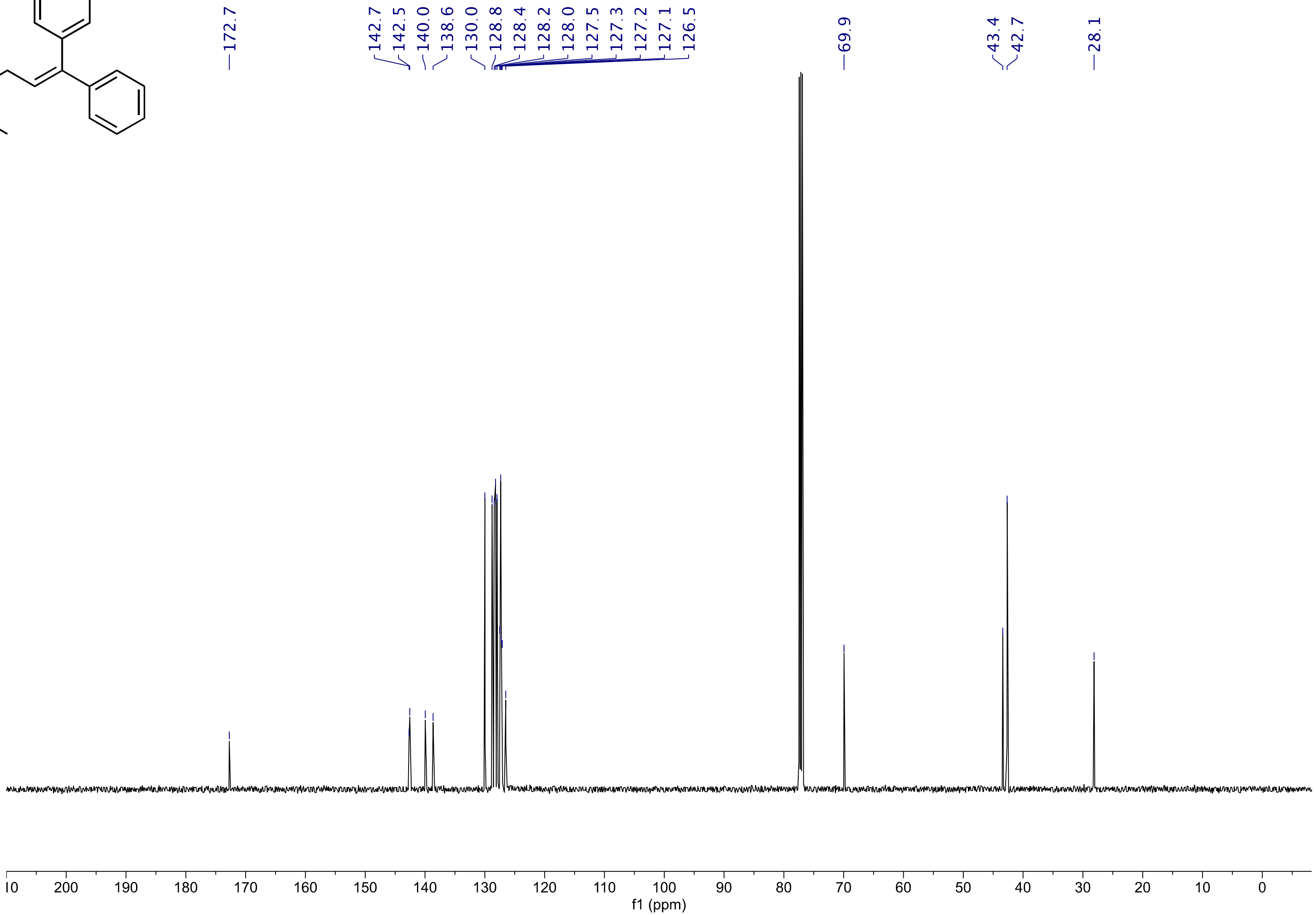

3i – ^{1}H NMR (500 MHz, $CDCl_3$)

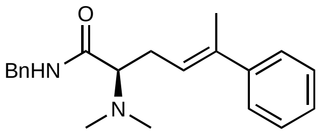

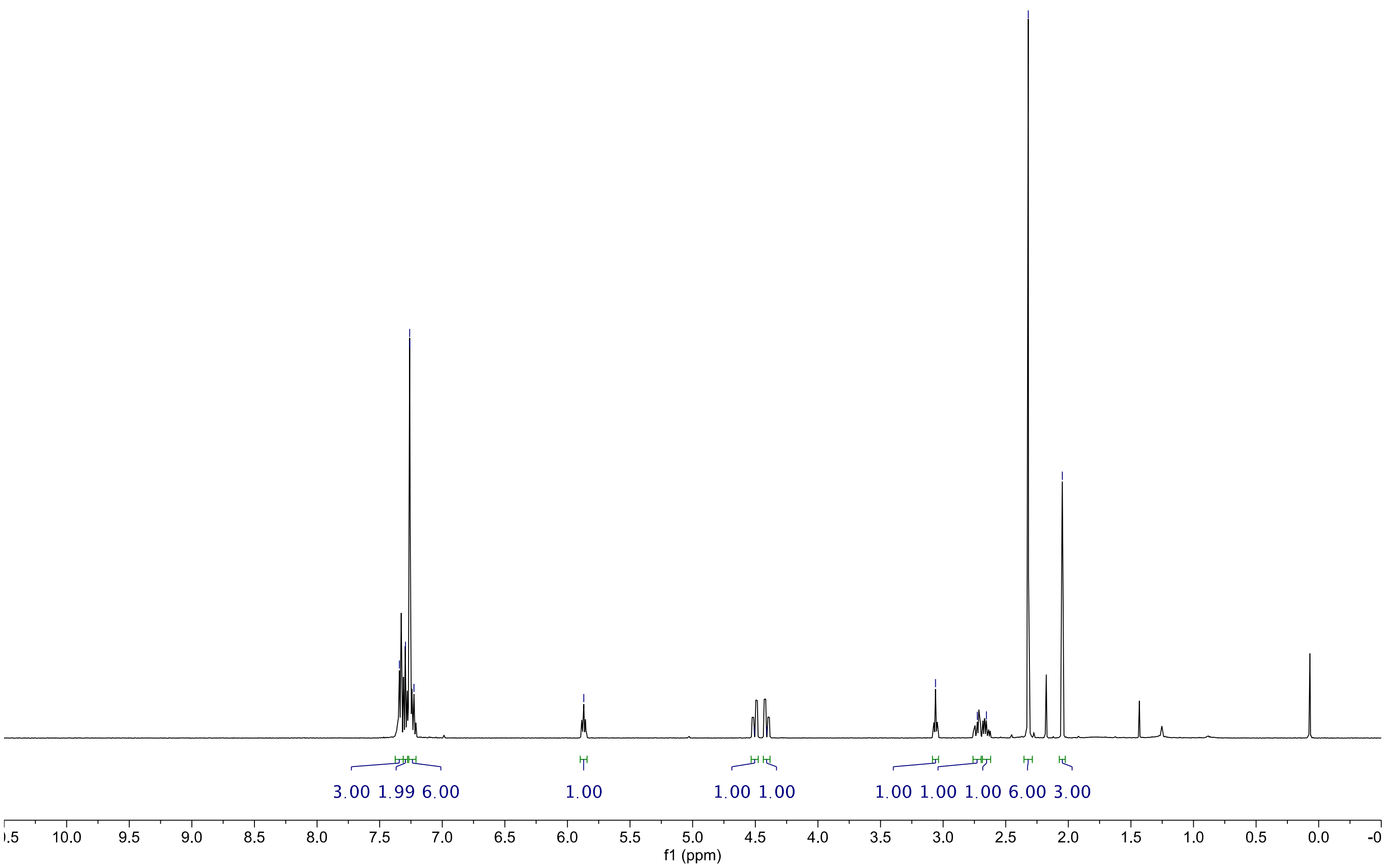

3i – ^{13}C NMR (126 MHz, $CDCl_3$)

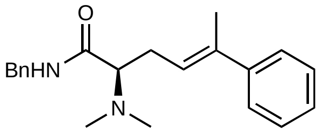

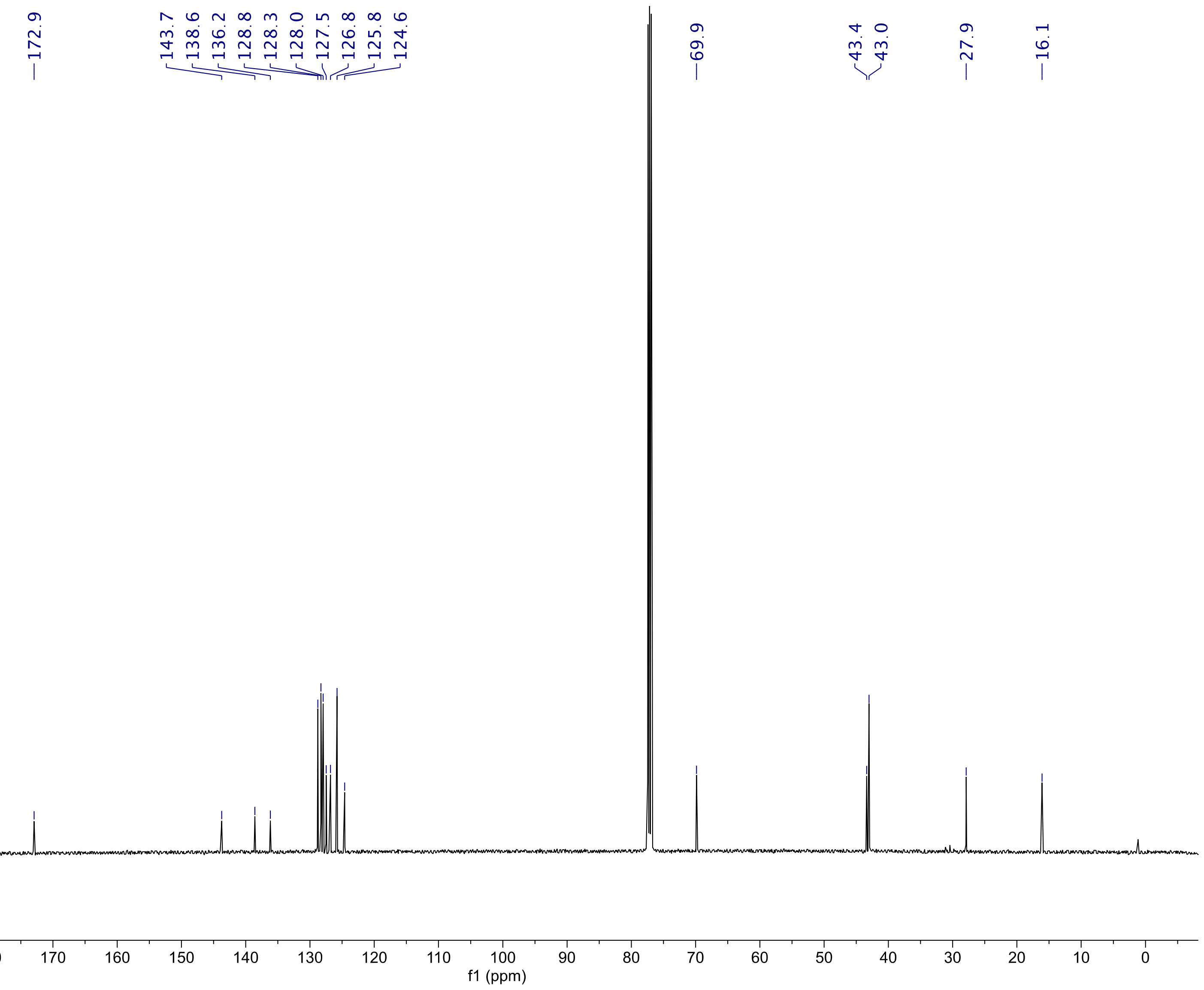

4i – ^{1}H NMR (500 MHz, $CDCl_3$)

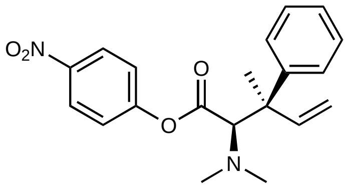

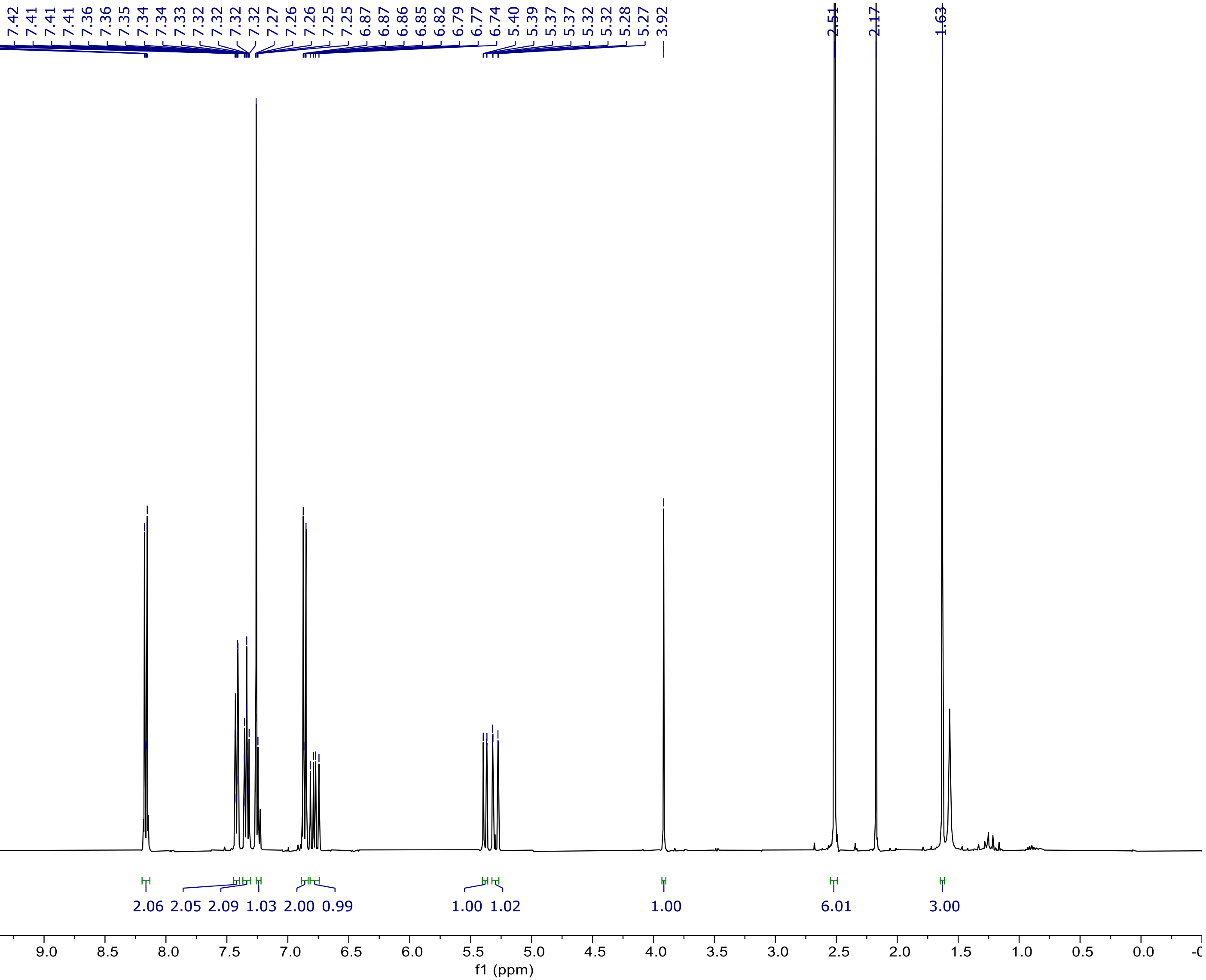

4i – ^{13}C NMR (126 MHz, $CDCl_3$)

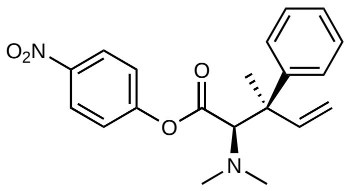

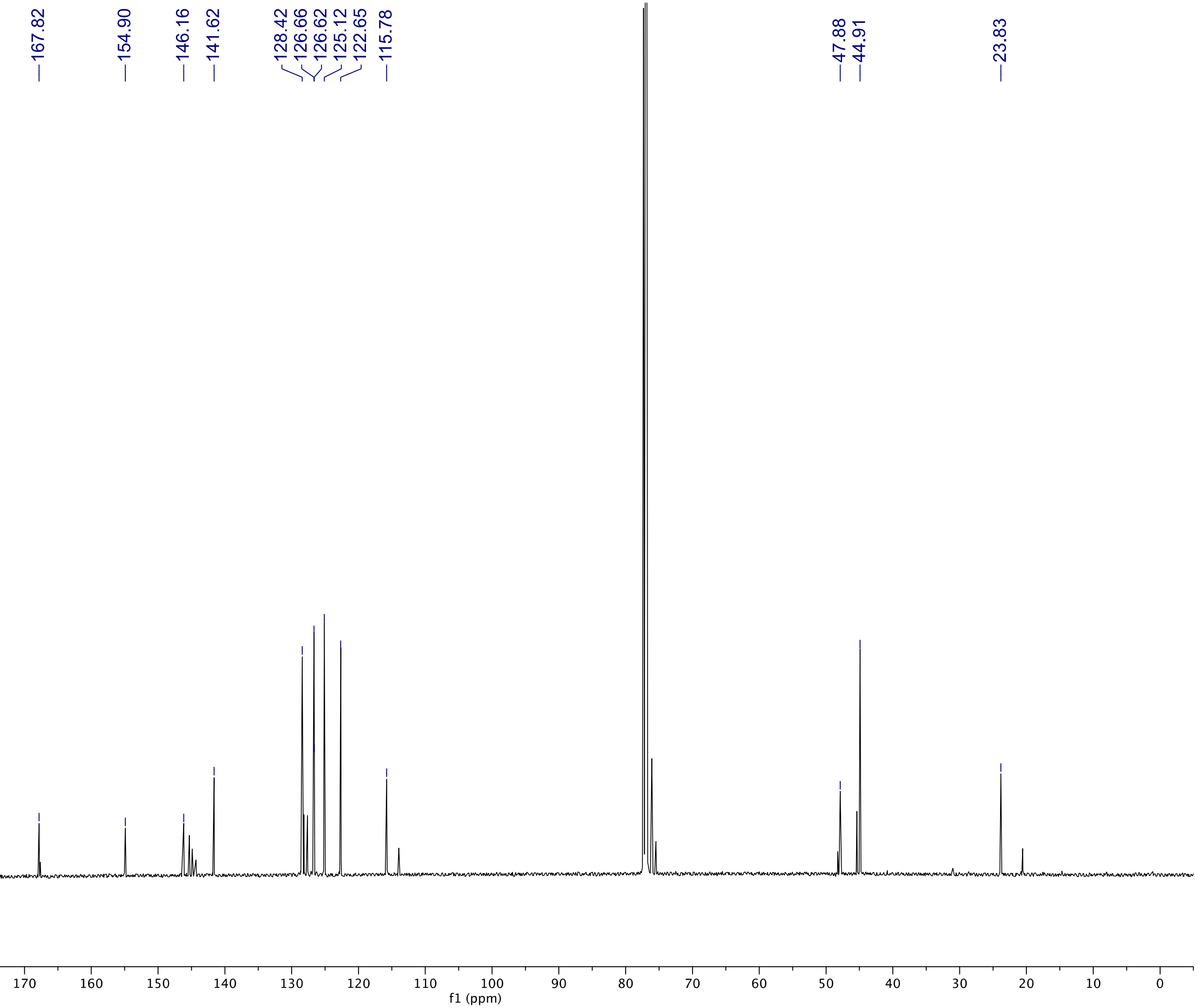

4j – ^{1}H NMR (500 MHz, $CDCl_3$)

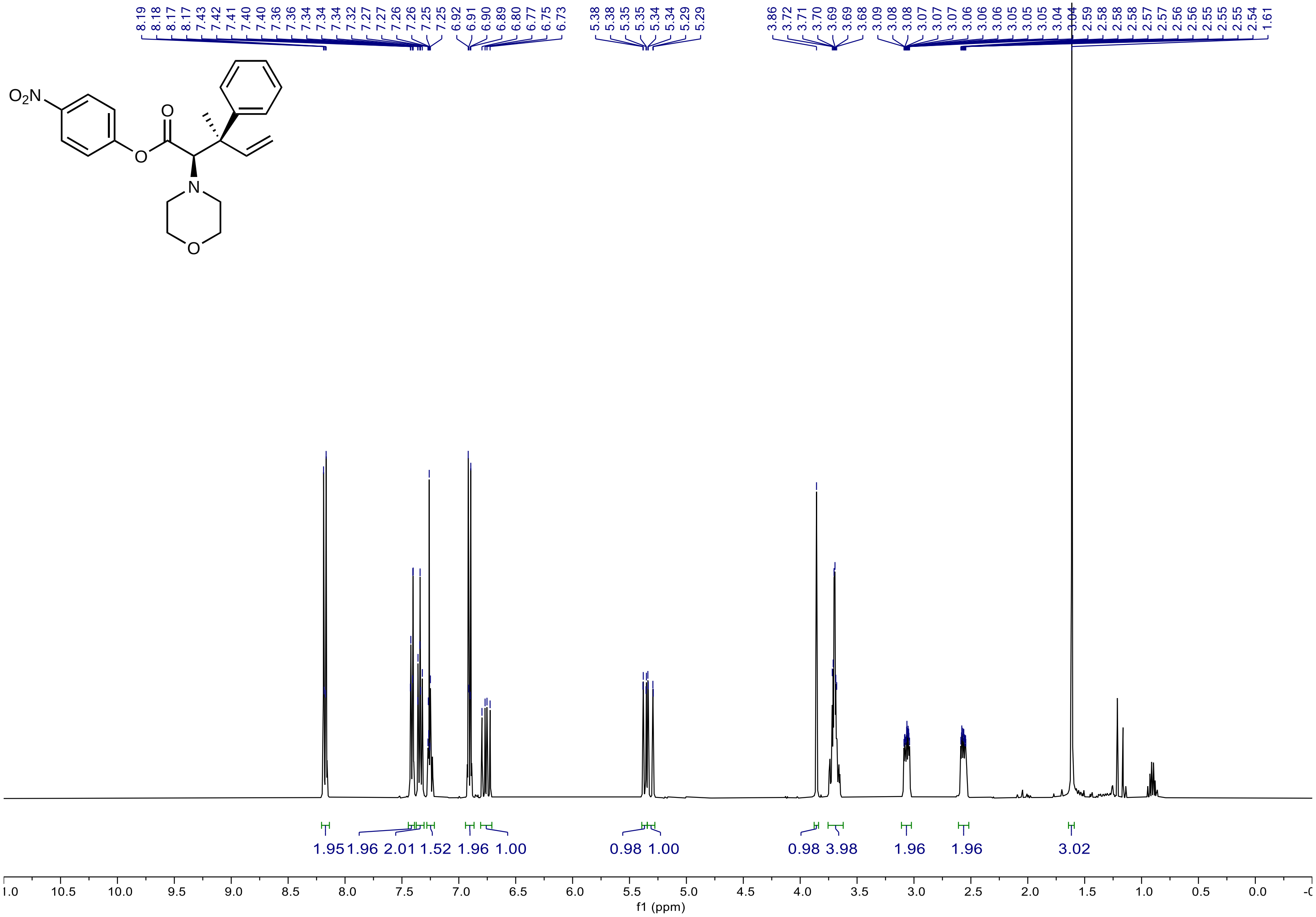

4j – ^{13}C NMR (126 MHz, $CDCl_3$)

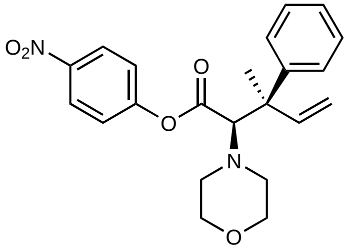

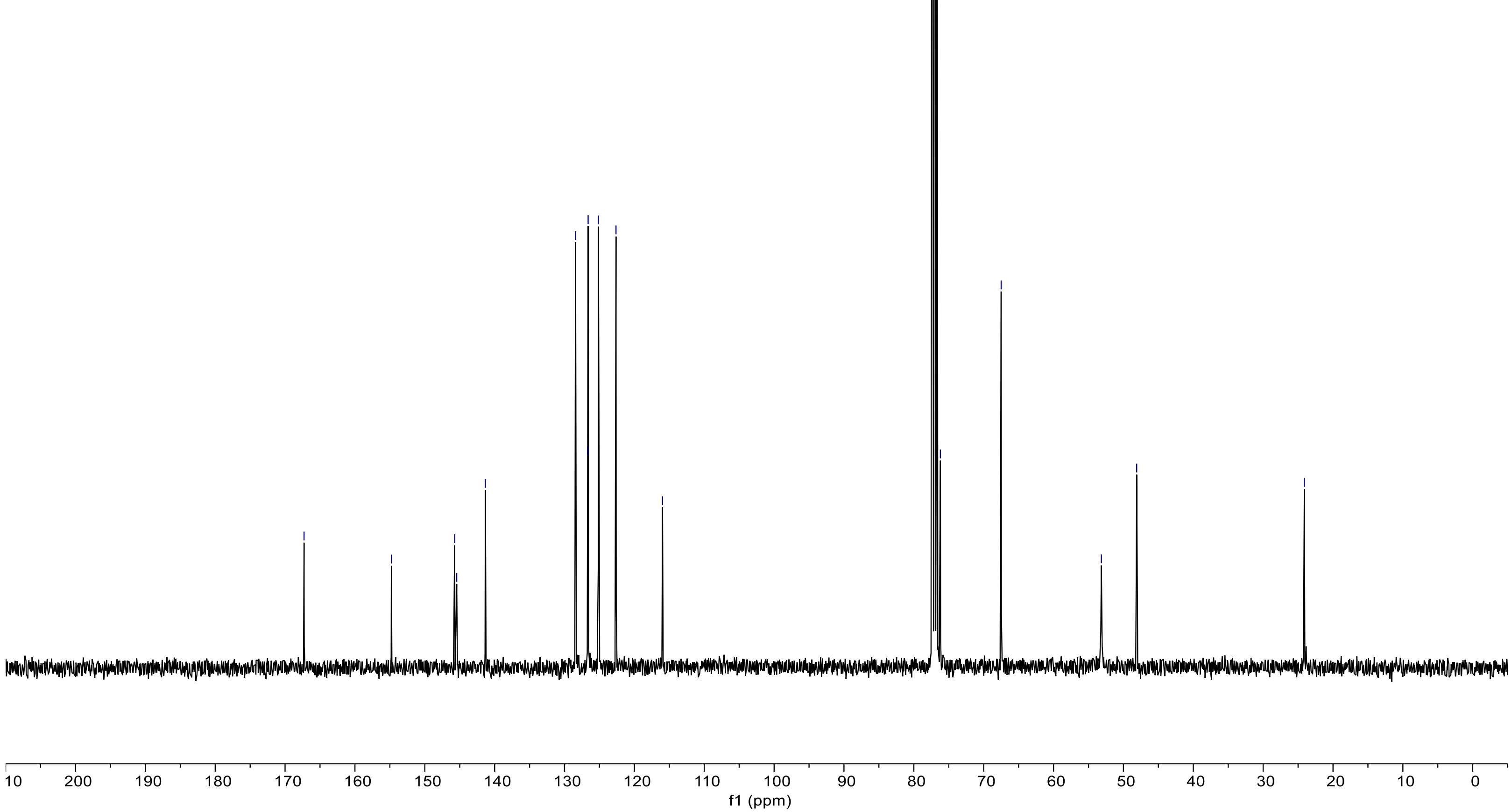

3k – ^{1}H NMR (500 MHz, $CDCl_3$)

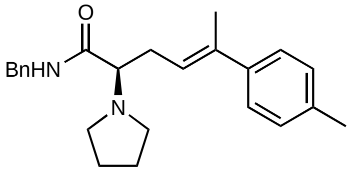

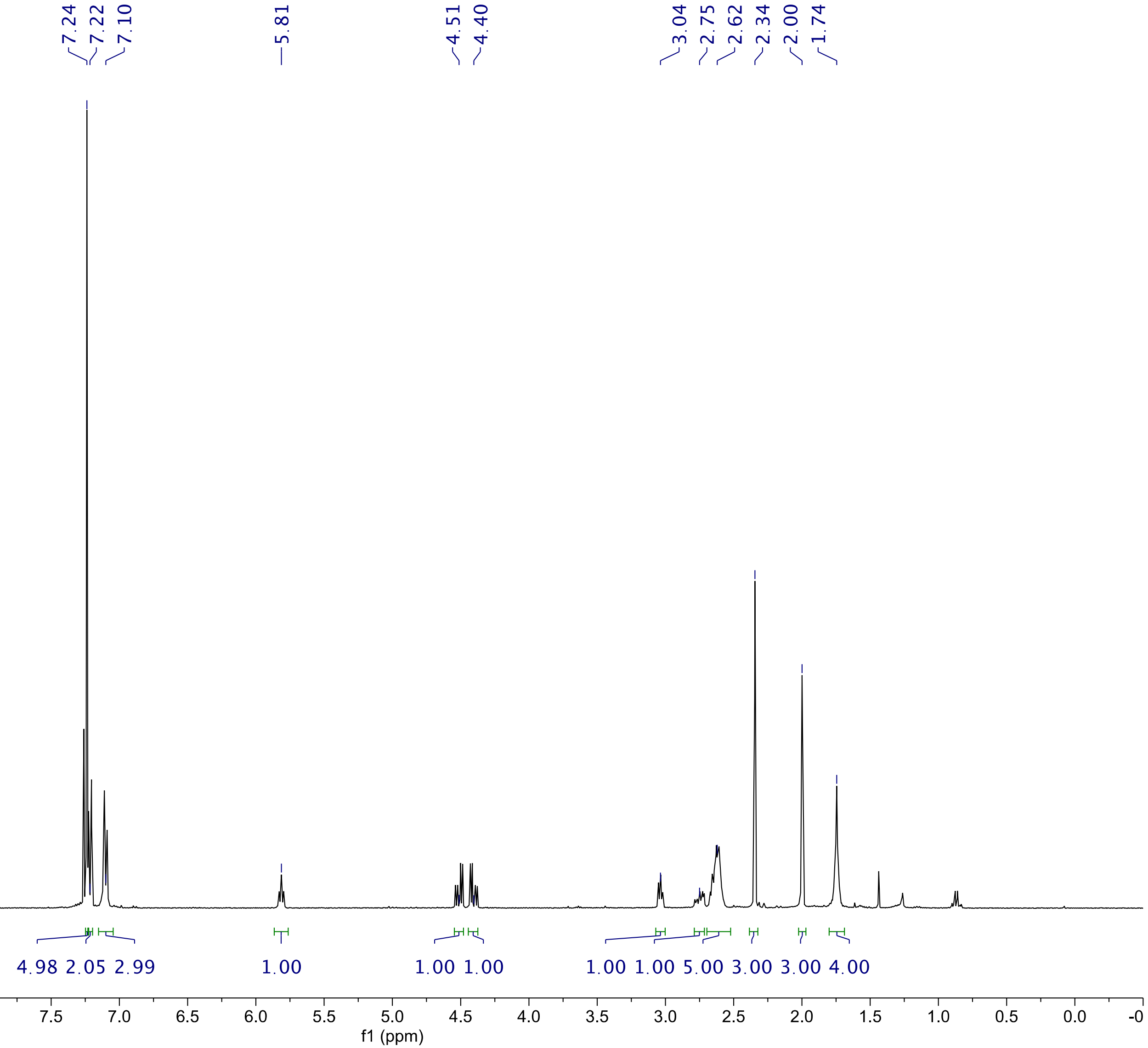

3k – ^{13}C NMR (126 MHz, $CDCl_3$)

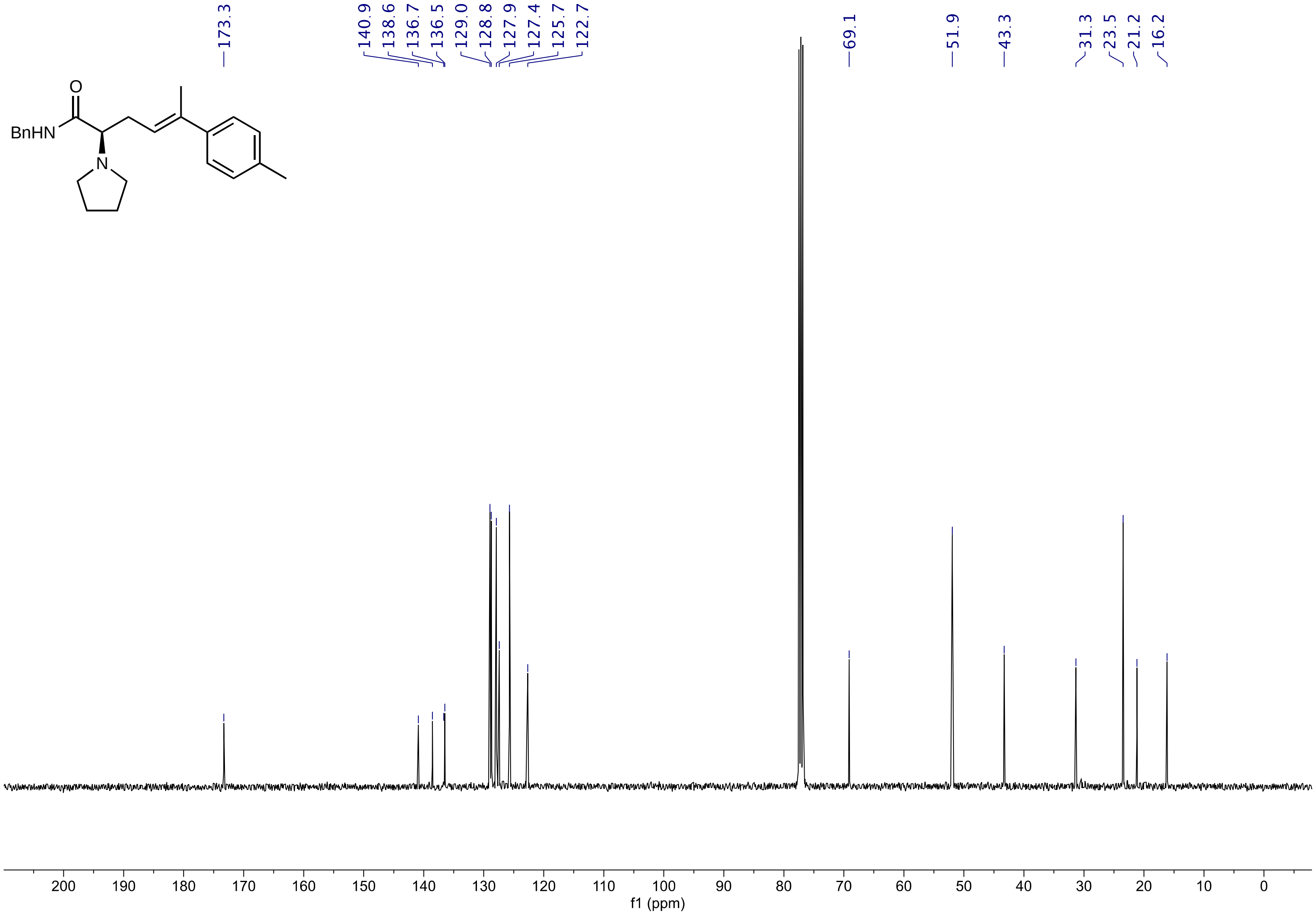

3l – ^{1}H NMR (500 MHz, $CDCl_3$)

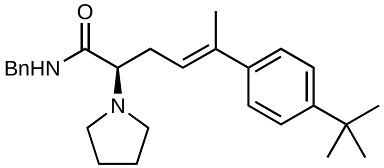

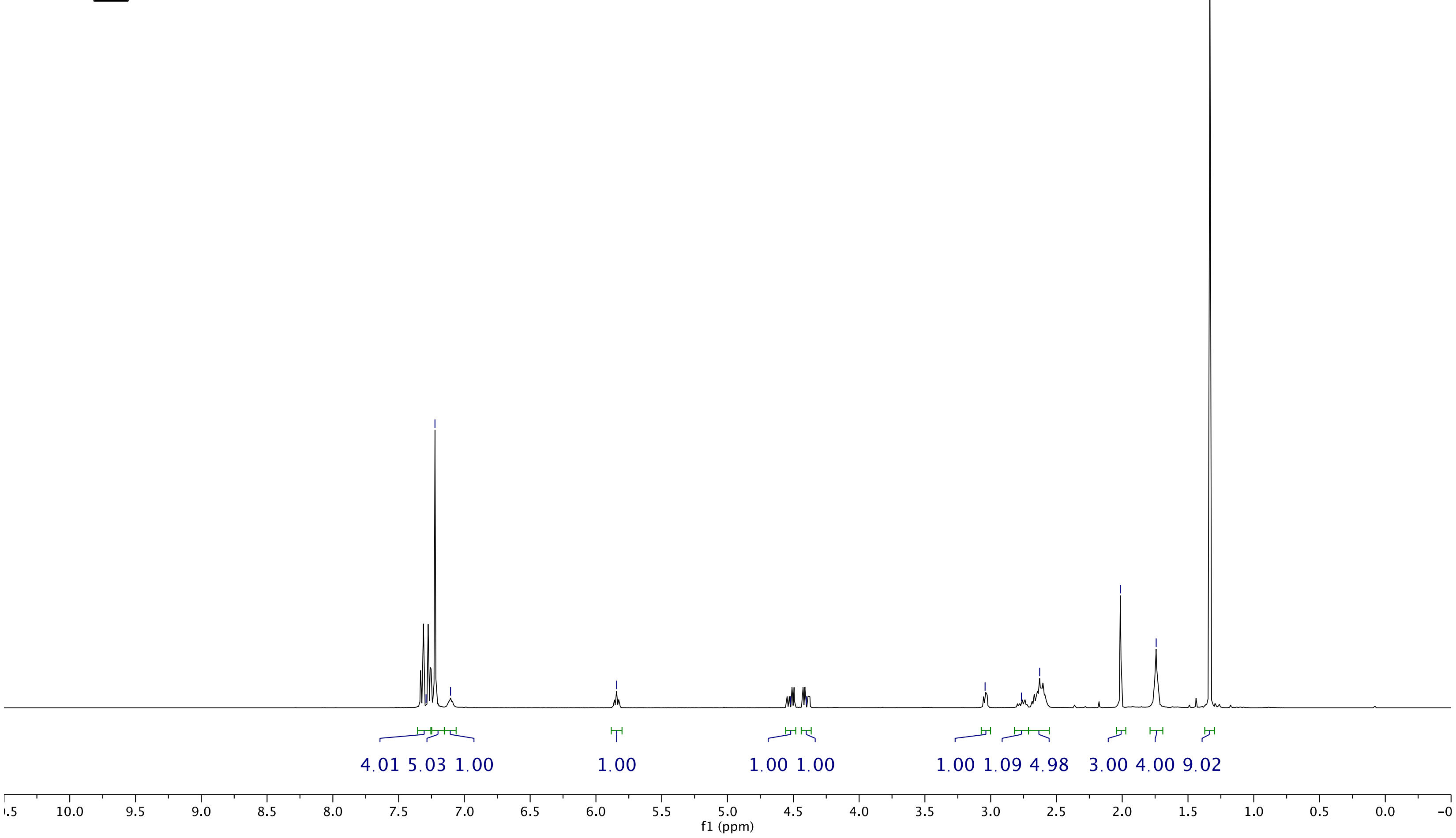

3l – ^{13}C NMR (126 MHz, $CDCl_3$)

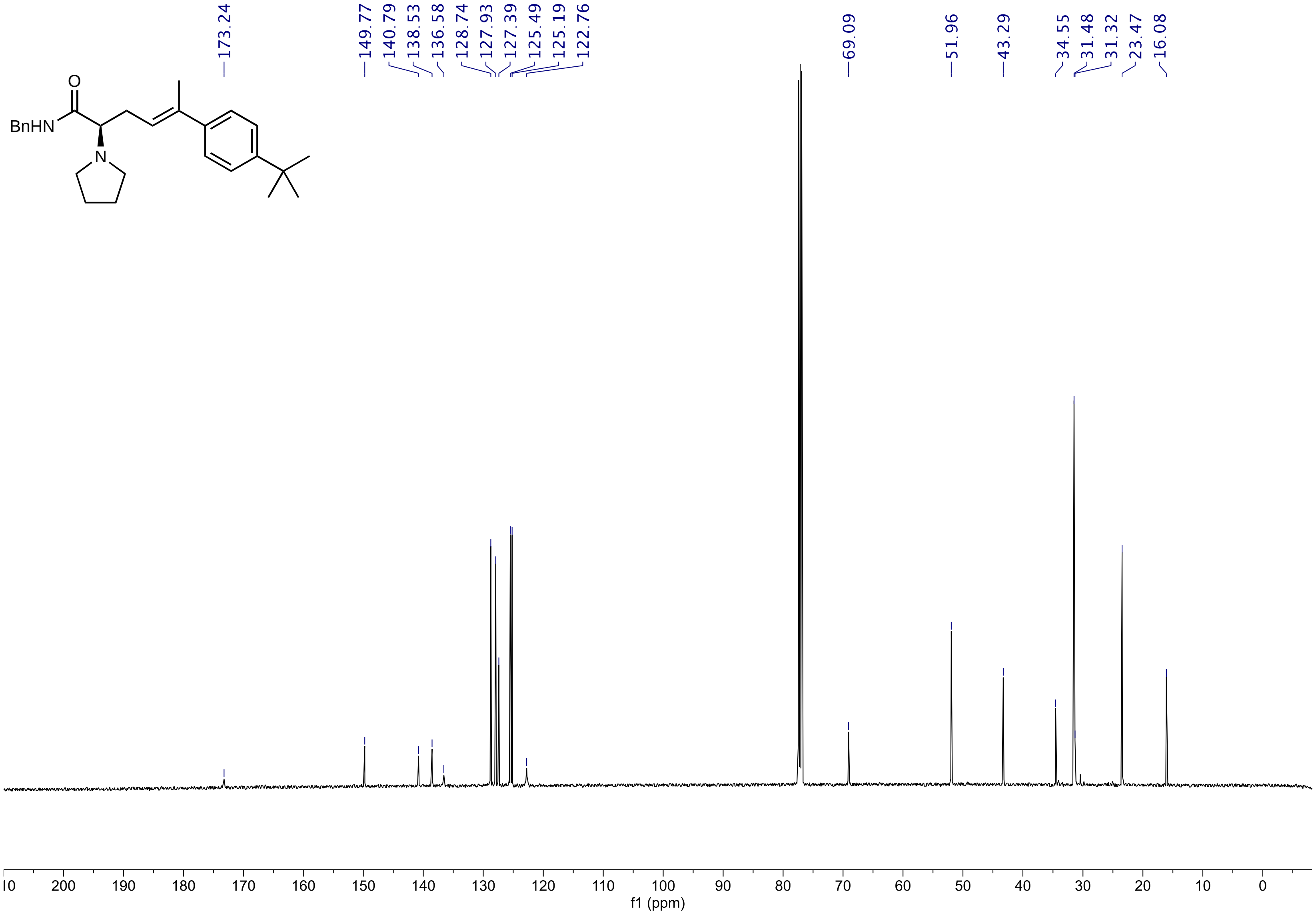

3m – ^{1}H NMR (500 MHz, $CDCl_3$)

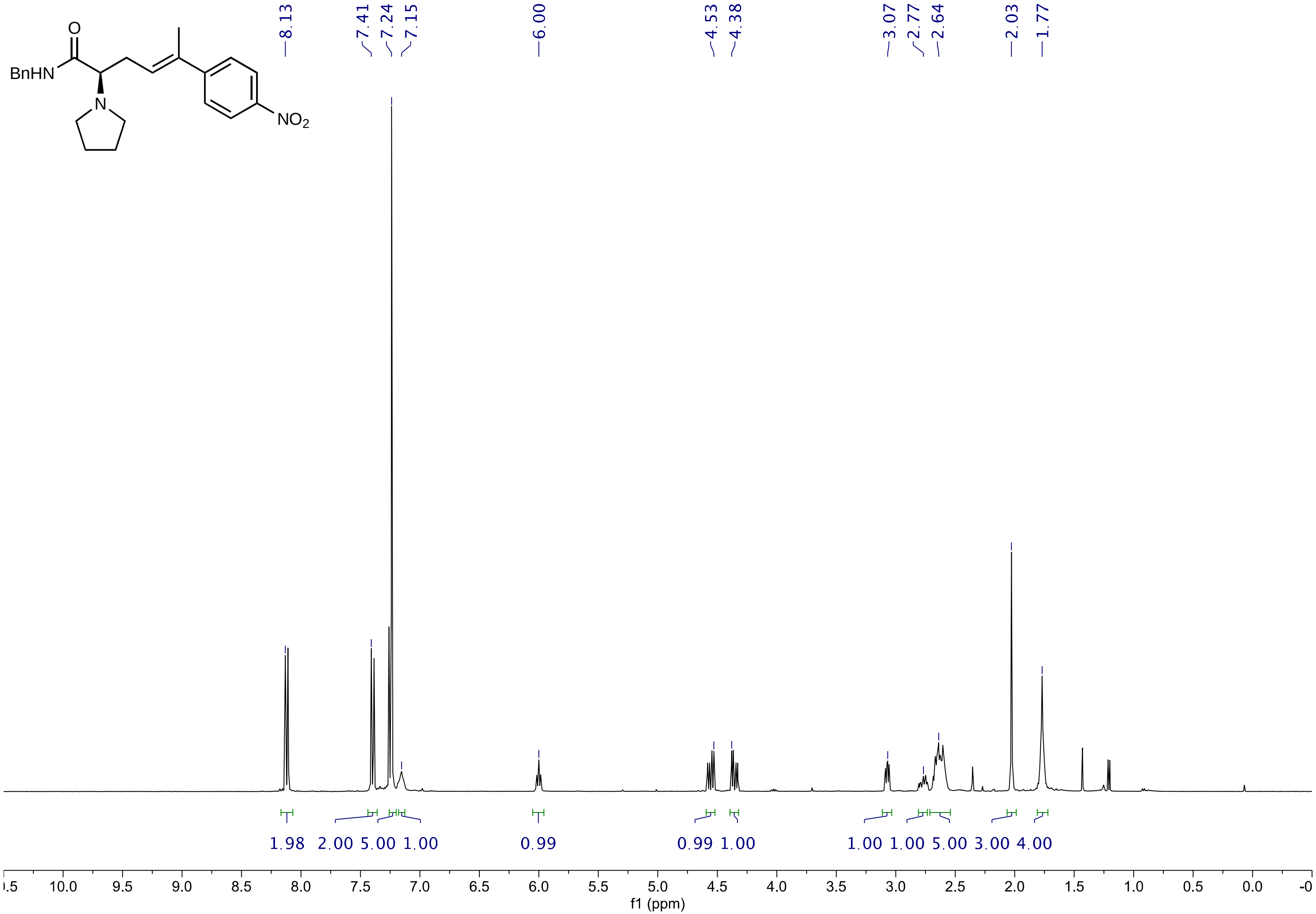

3m – ^{13}C NMR (126 MHz, $CDCl_3$)

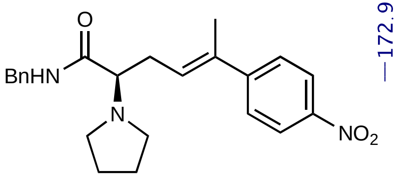

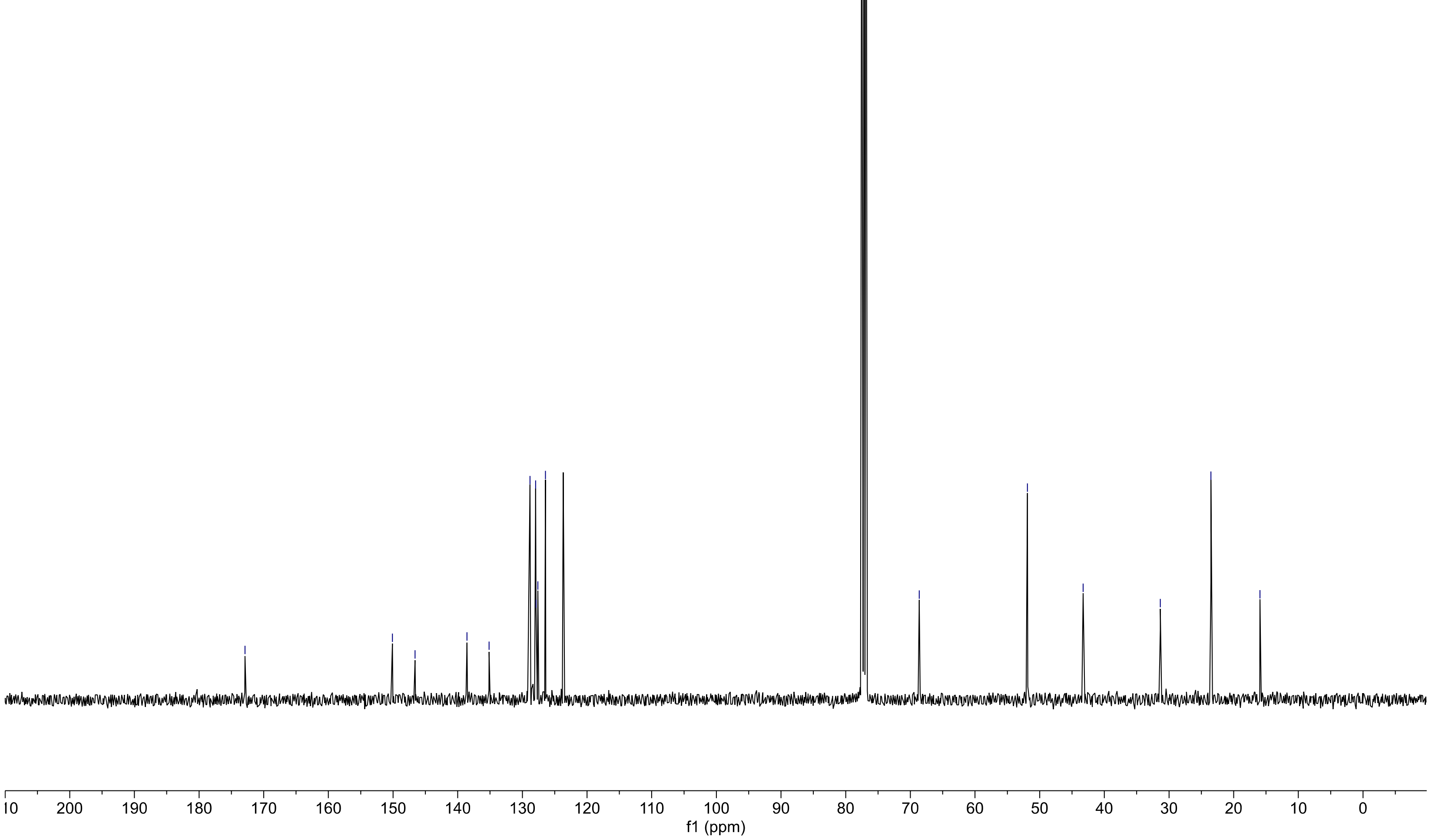

3n – ^{1}H NMR (500 MHz, $CDCl_3$)

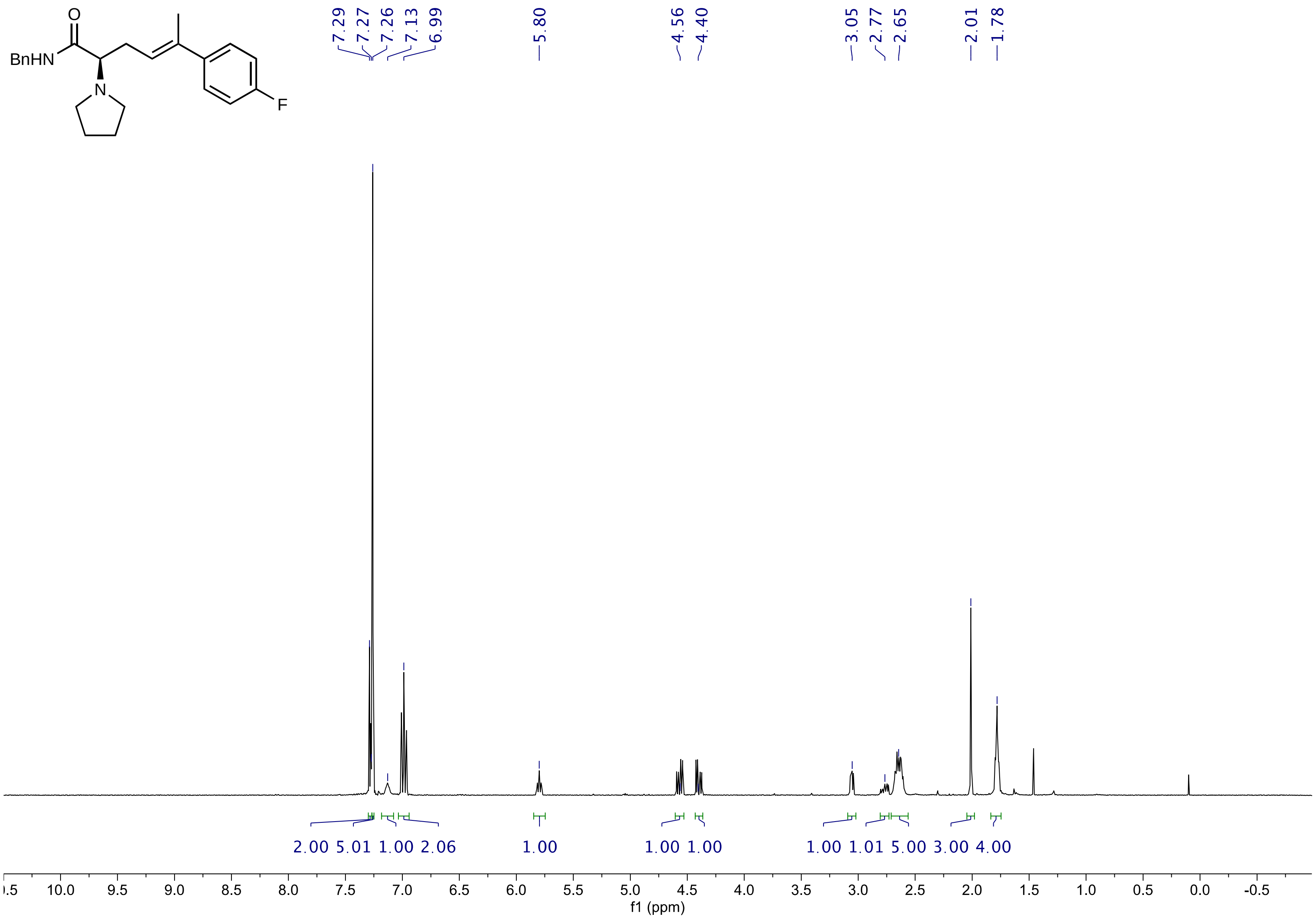

3n – ^{13}C NMR (126 MHz, $CDCl_3$)

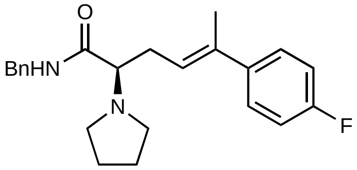

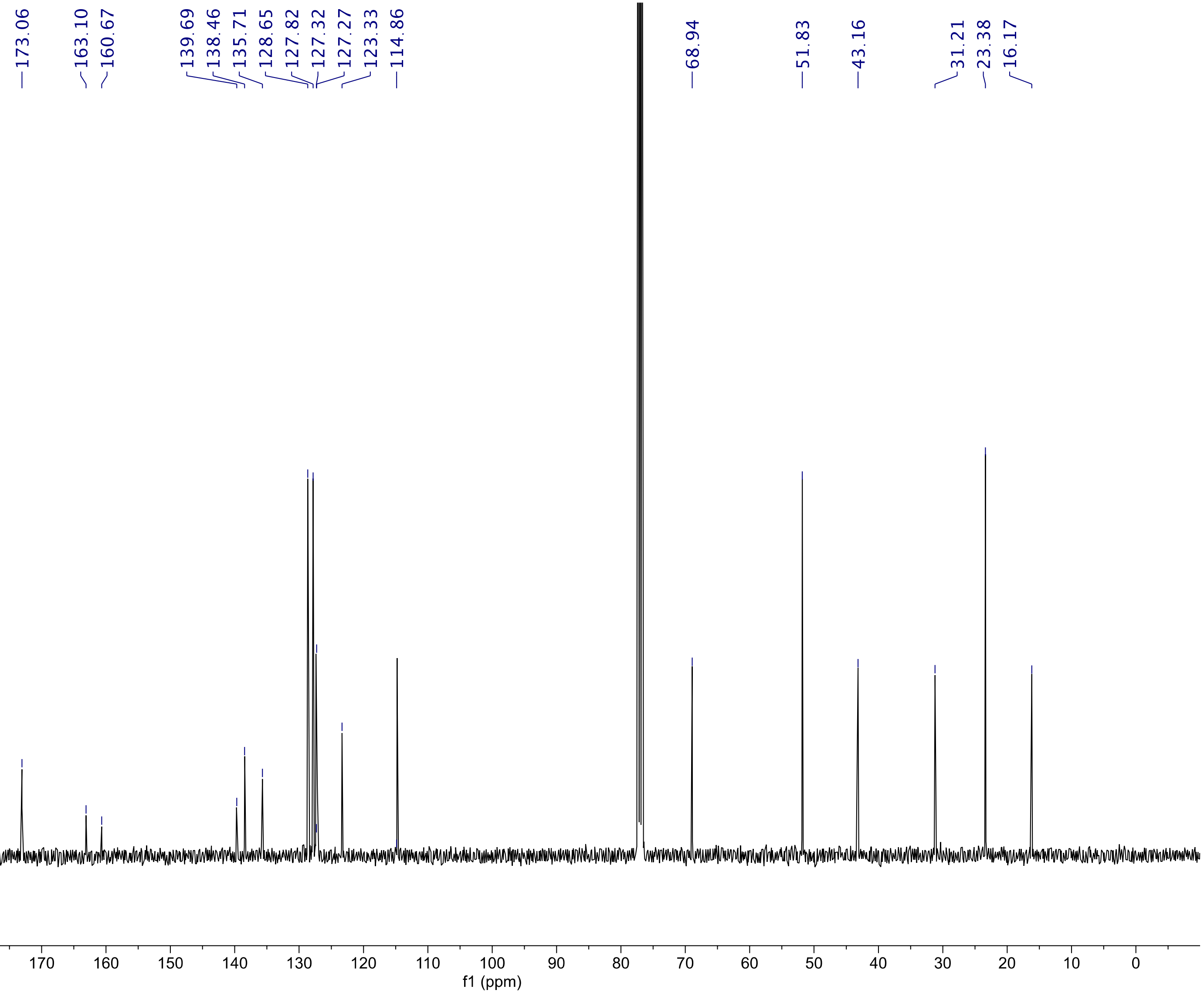

3n – ^{19}F NMR (377 MHz, $CDCl_3$)

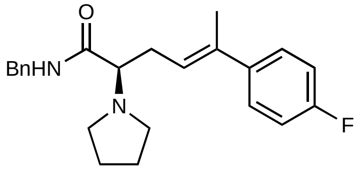

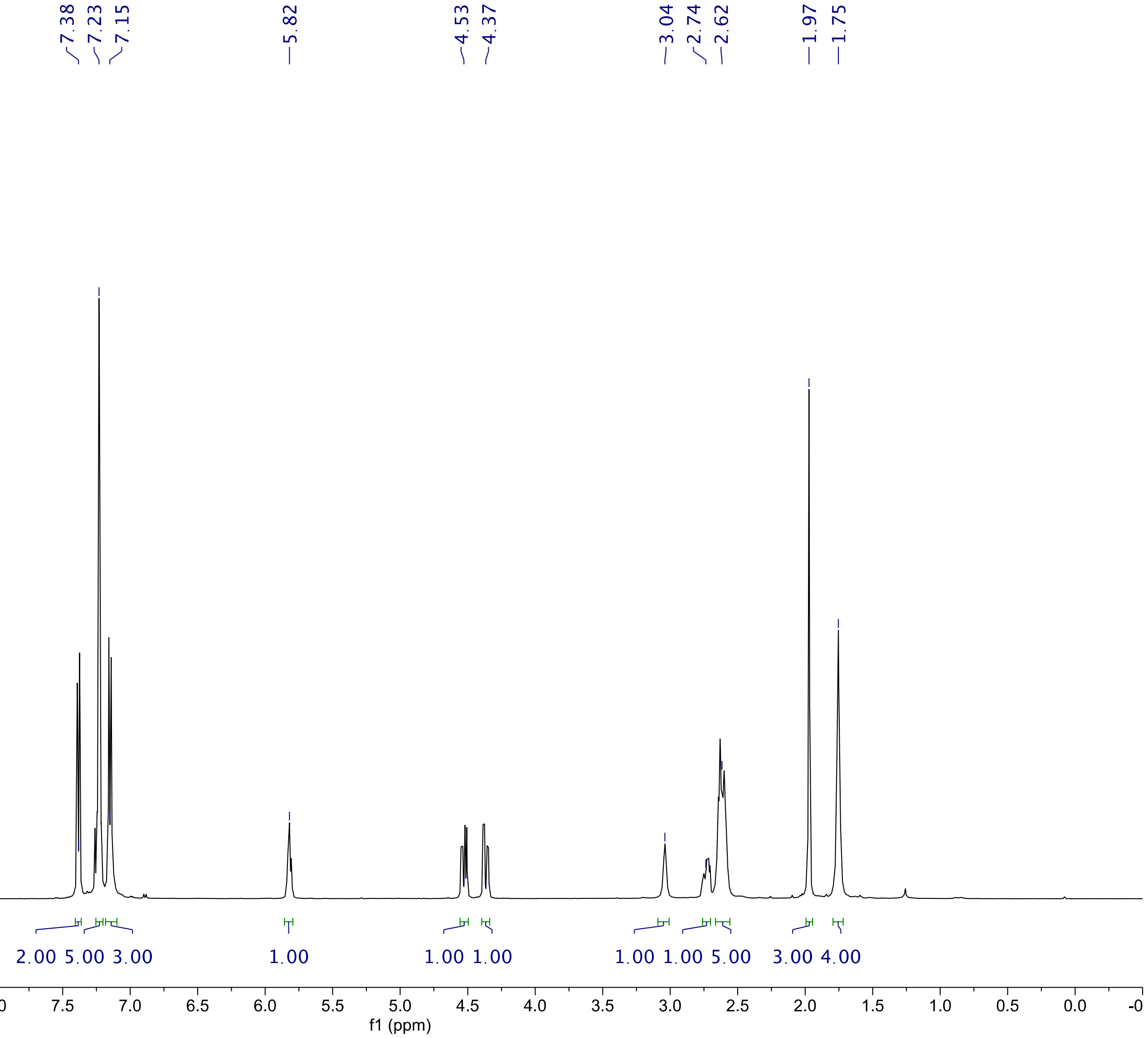

4n – ^{19}F NMR (377 MHz, $CDCl_3$)

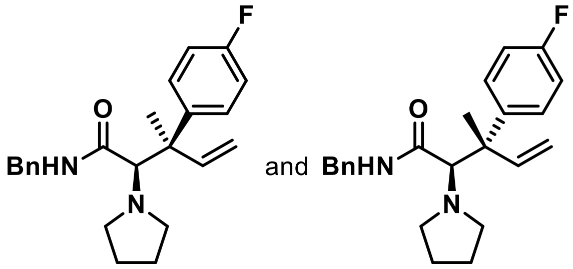

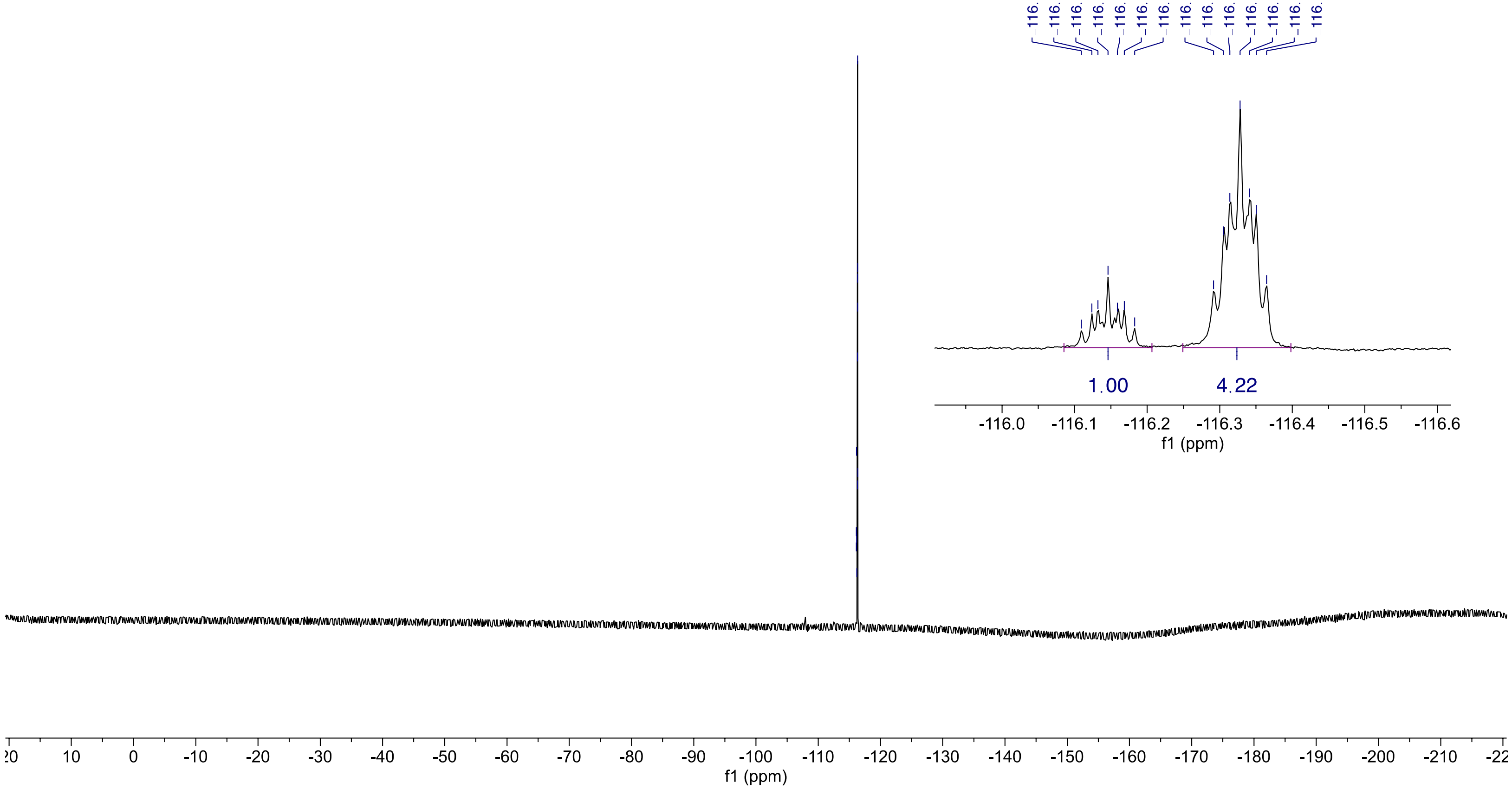

3o – ^{1}H NMR (500 MHz, $CDCl_3$)

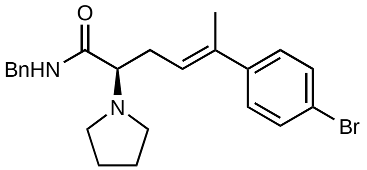

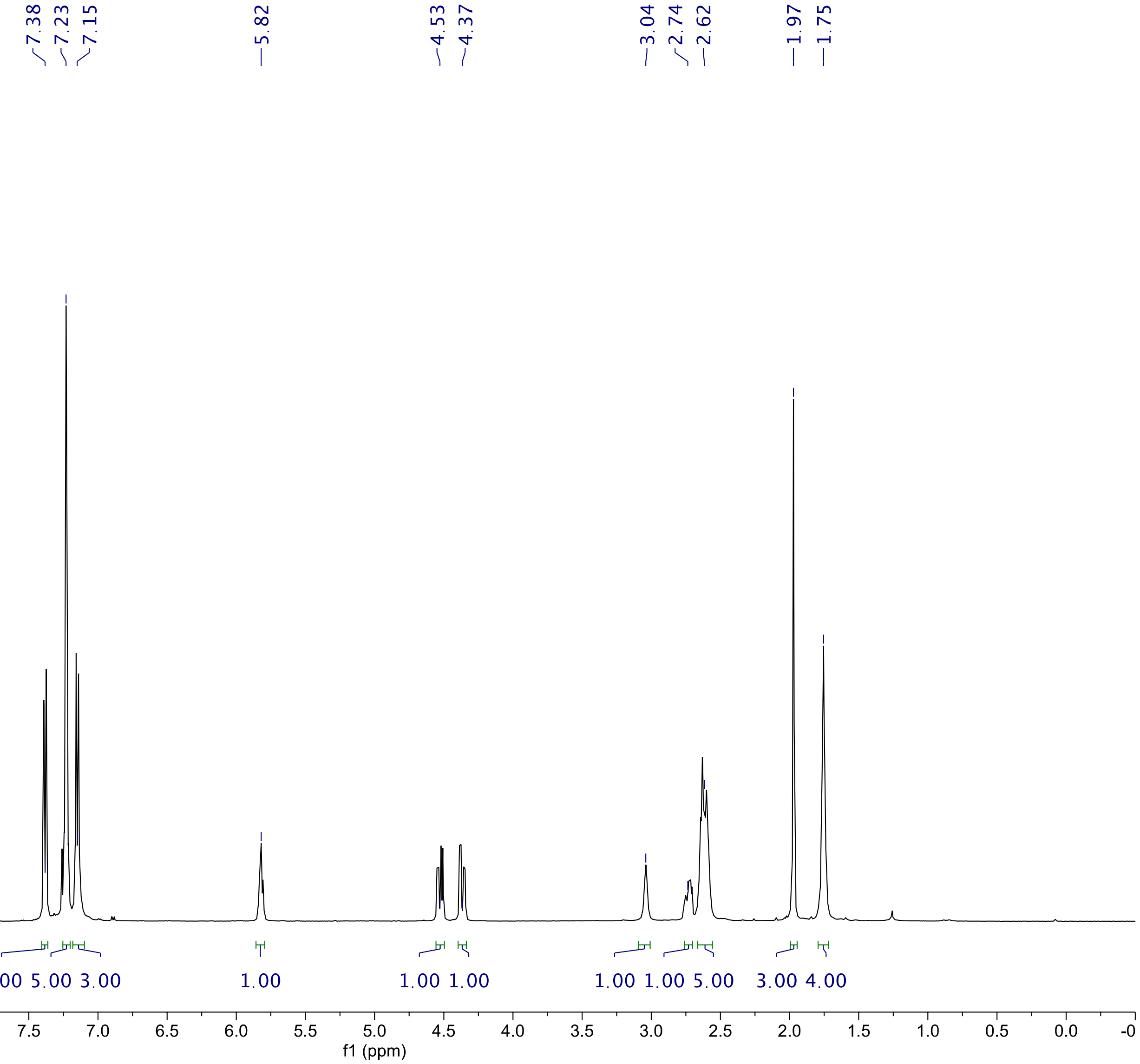

3o – ^{13}C NMR (126 MHz, $CDCl_3$)

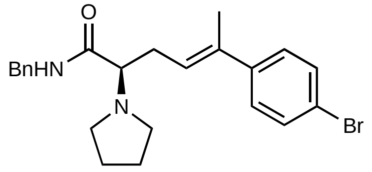

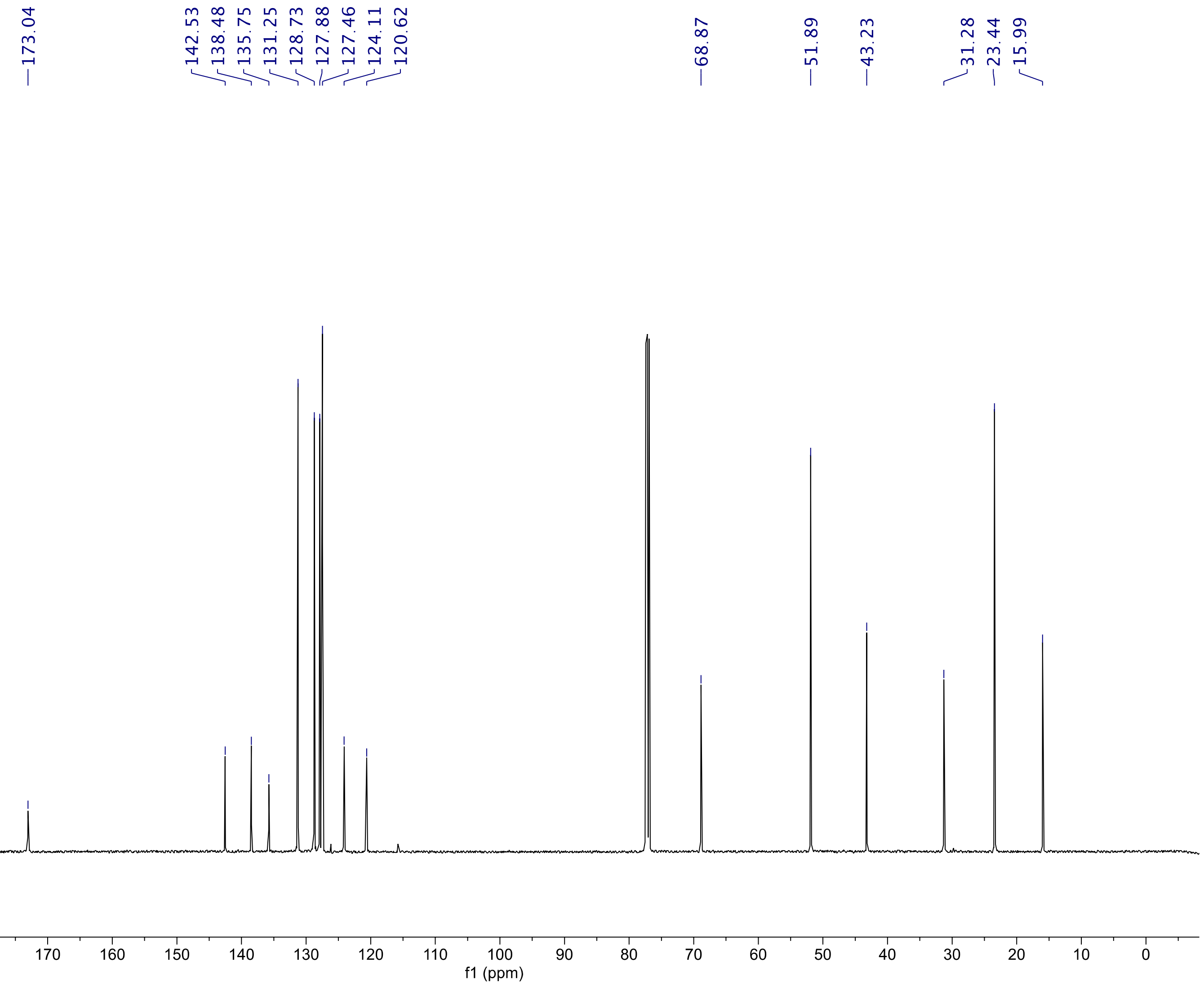

3p – ^{1}H NMR (500 MHz, $CDCl_3$)

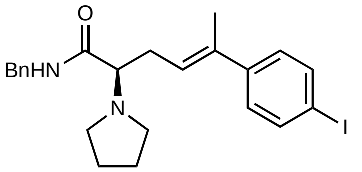

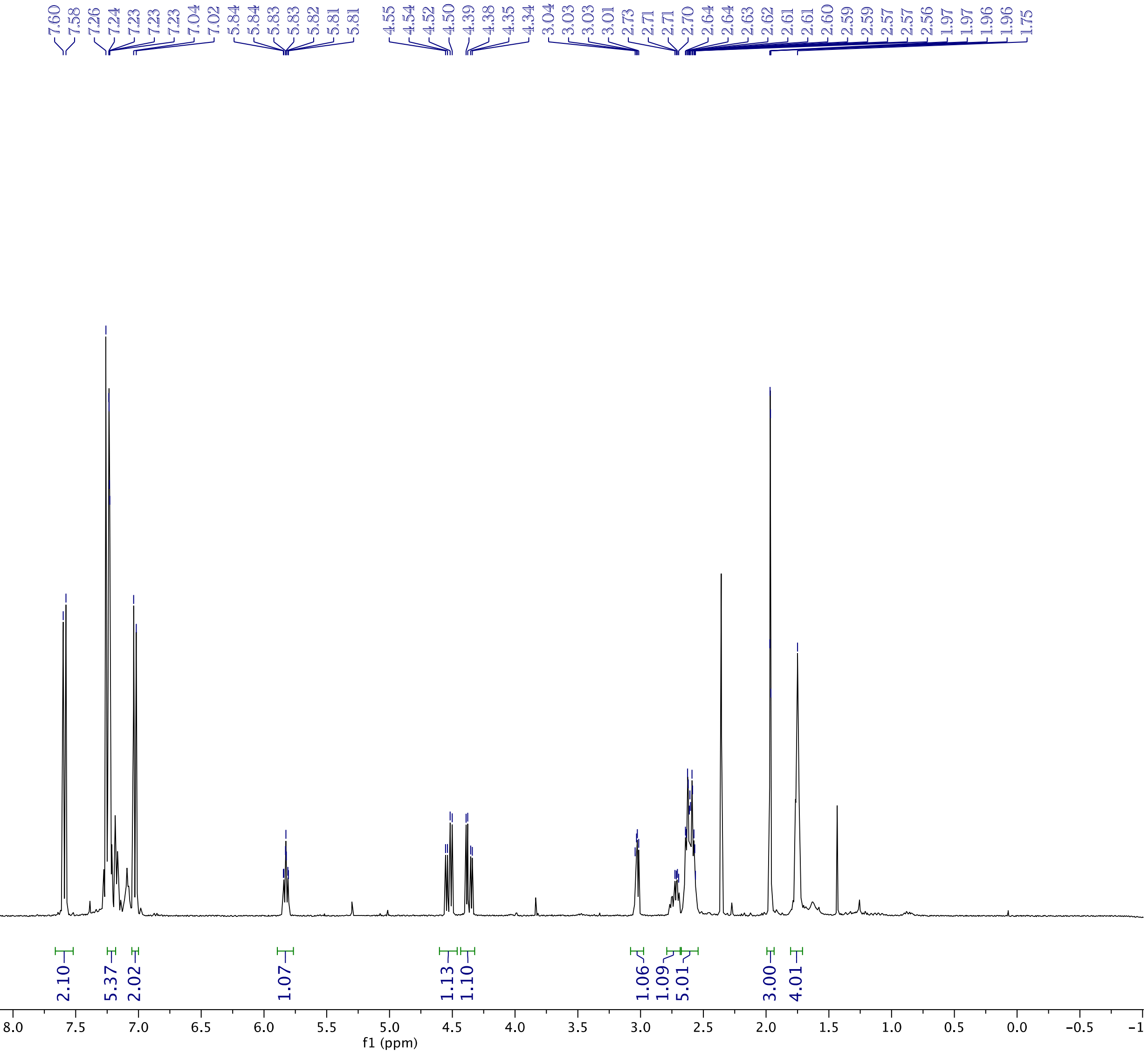

3p – ^{13}C NMR (126 MHz, CDCl$_3$)

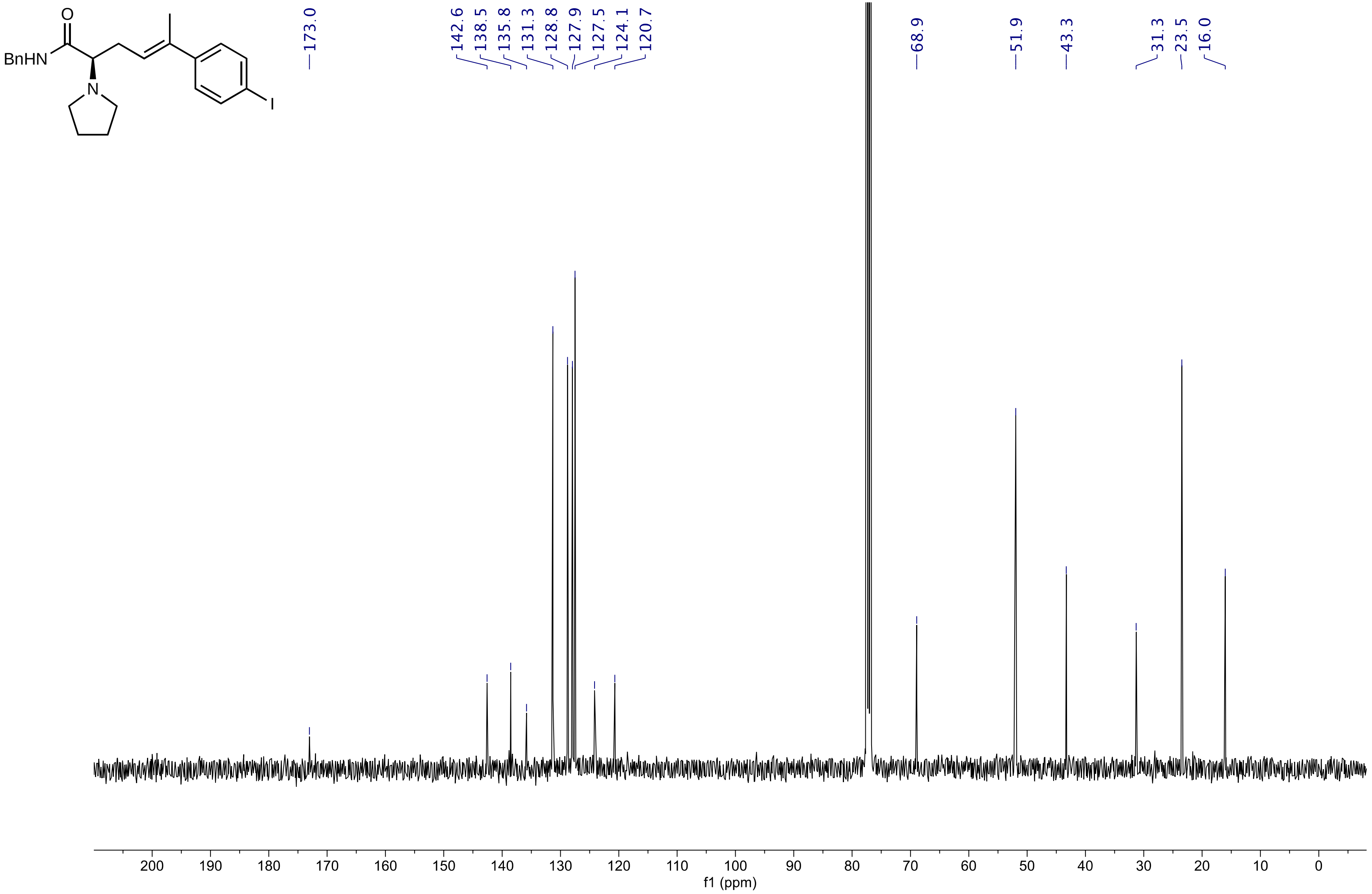

3r – ^{1}H NMR (500 MHz, $CDCl_3$)

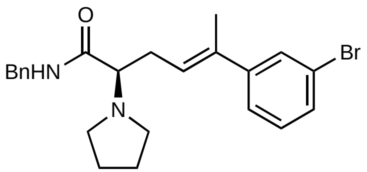

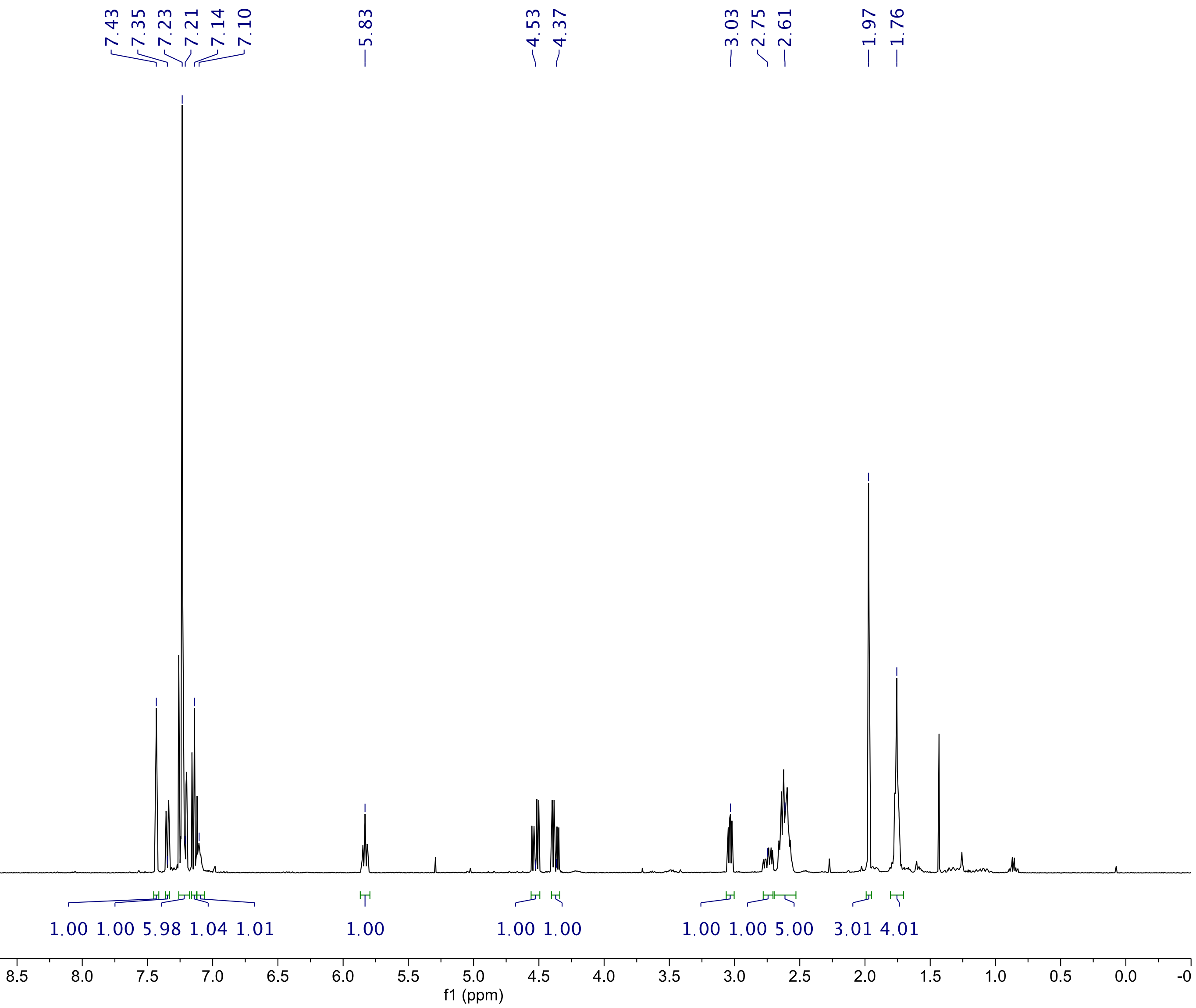

3r – ^{13}C NMR (126 MHz, $CDCl_3$)

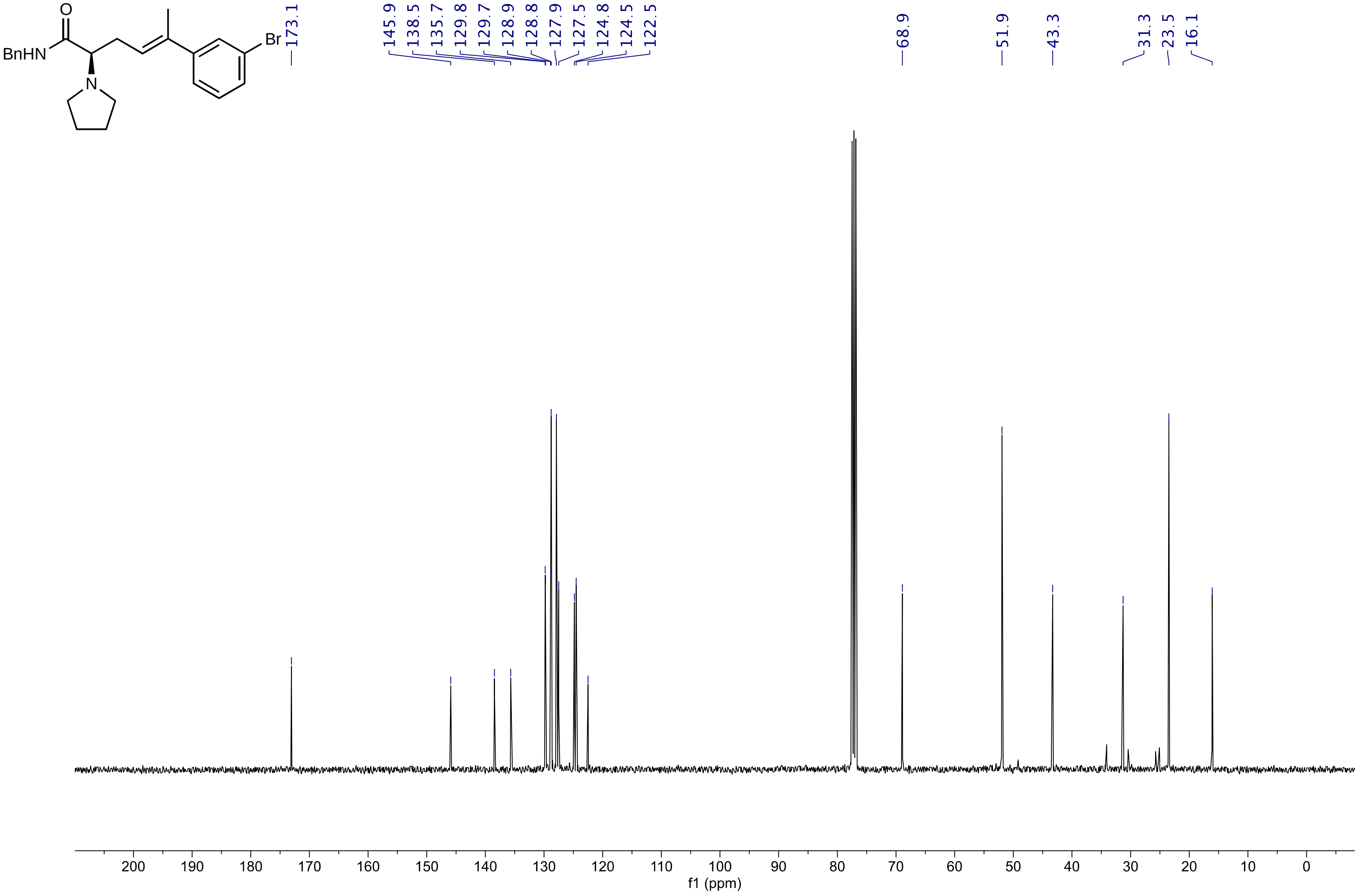

3s – ^{1}H NMR (500 MHz, $CDCl_3$)

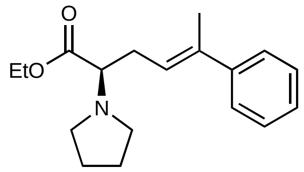

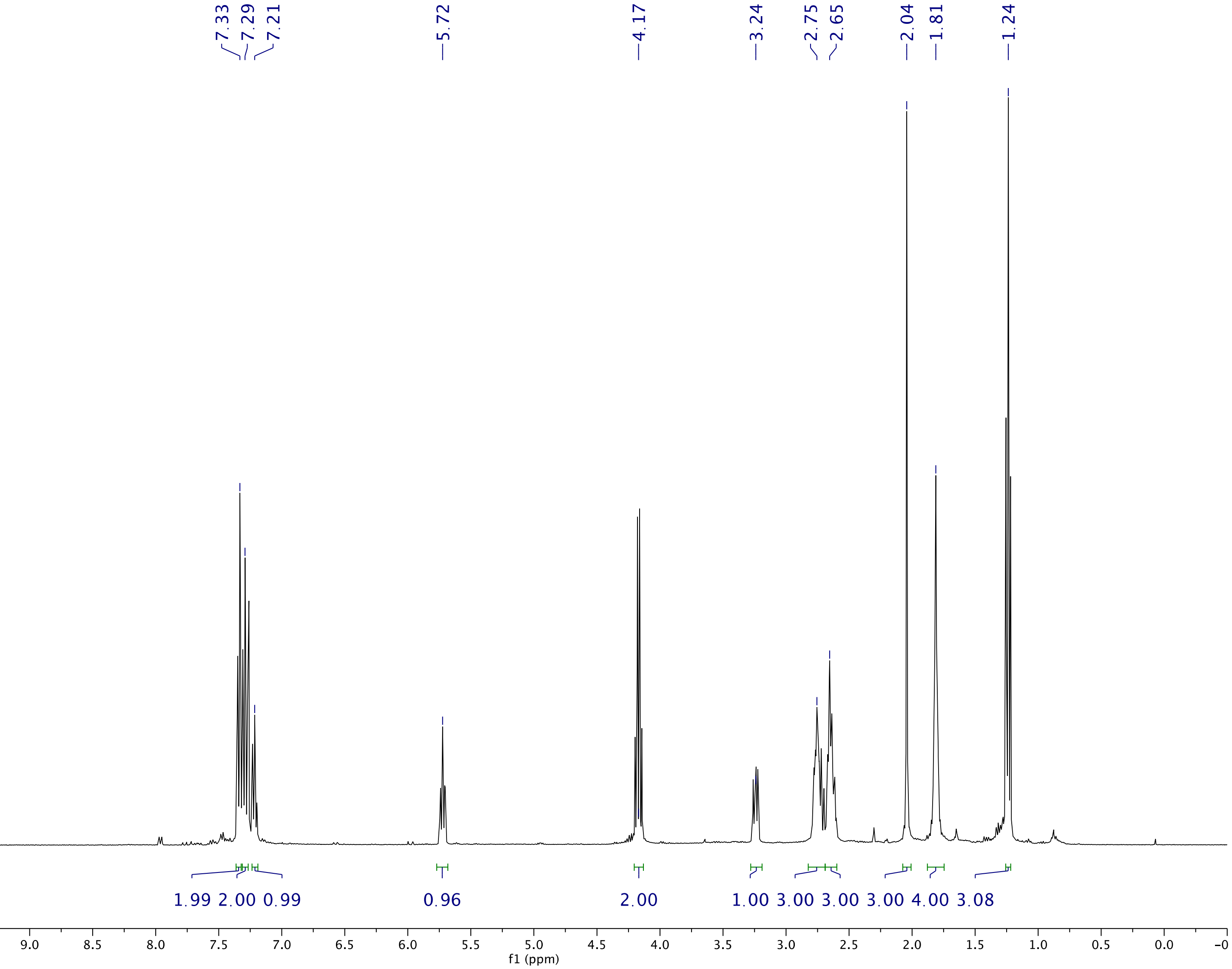

3s – ^{13}C NMR (126 MHz, $CDCl_3$)

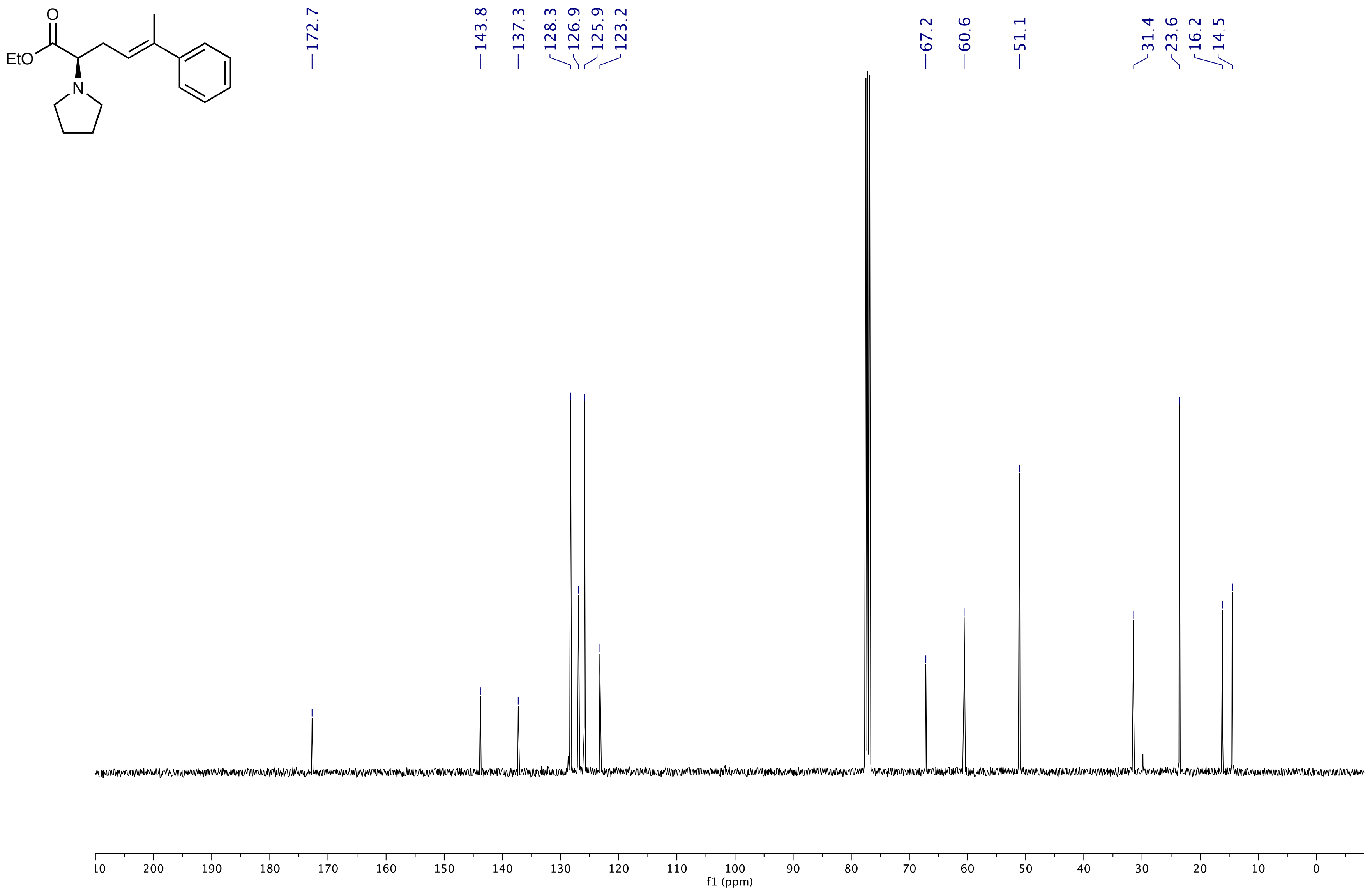

3t – ^{1}H NMR (500 MHz, $CDCl_3$)

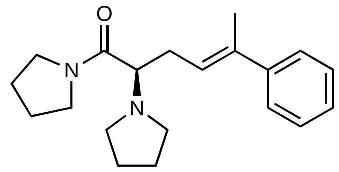

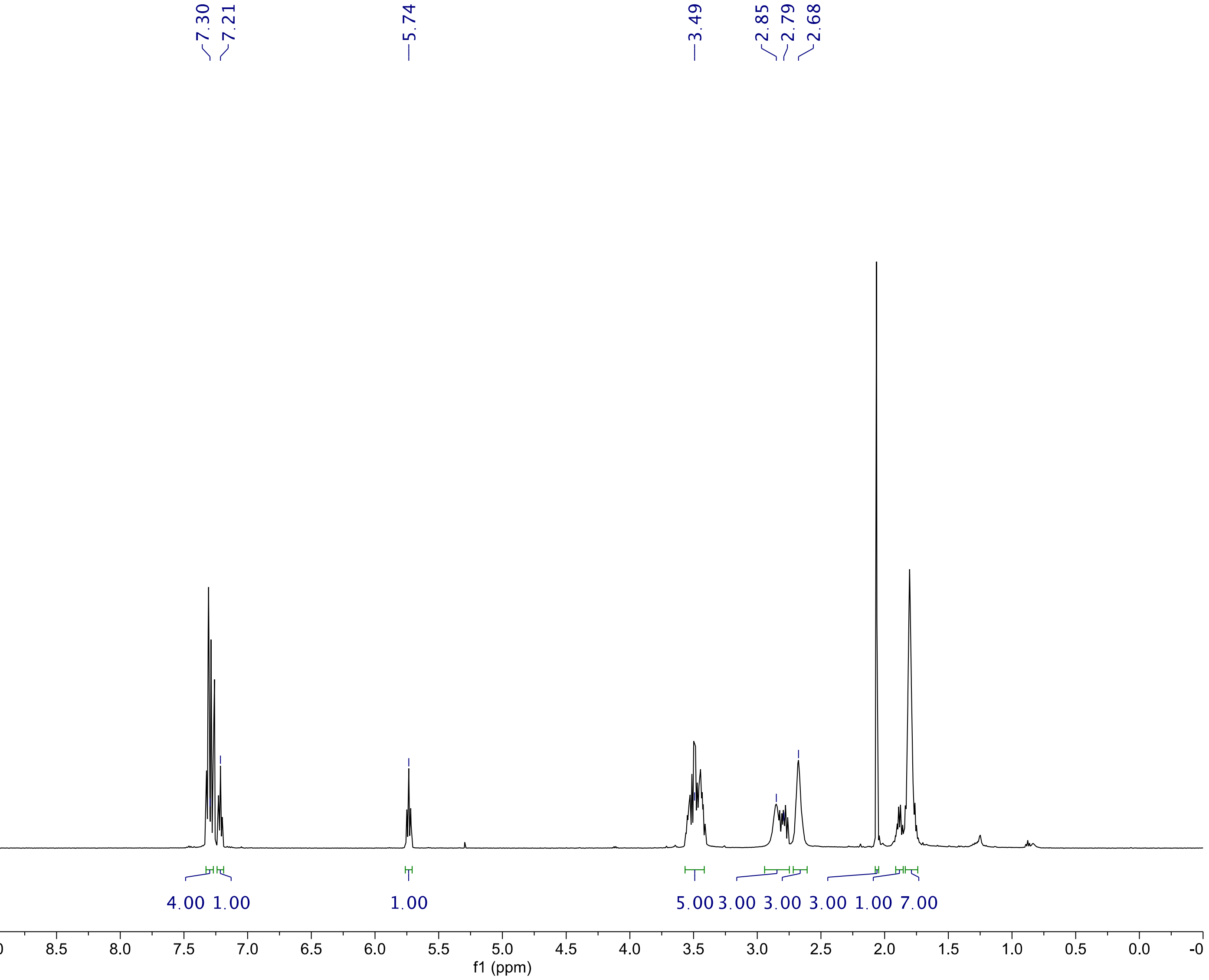

3t – ^{13}C NMR (126 MHz, $CDCl_3$)

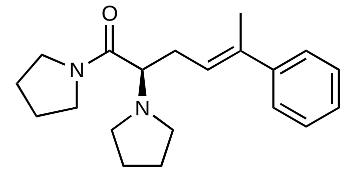

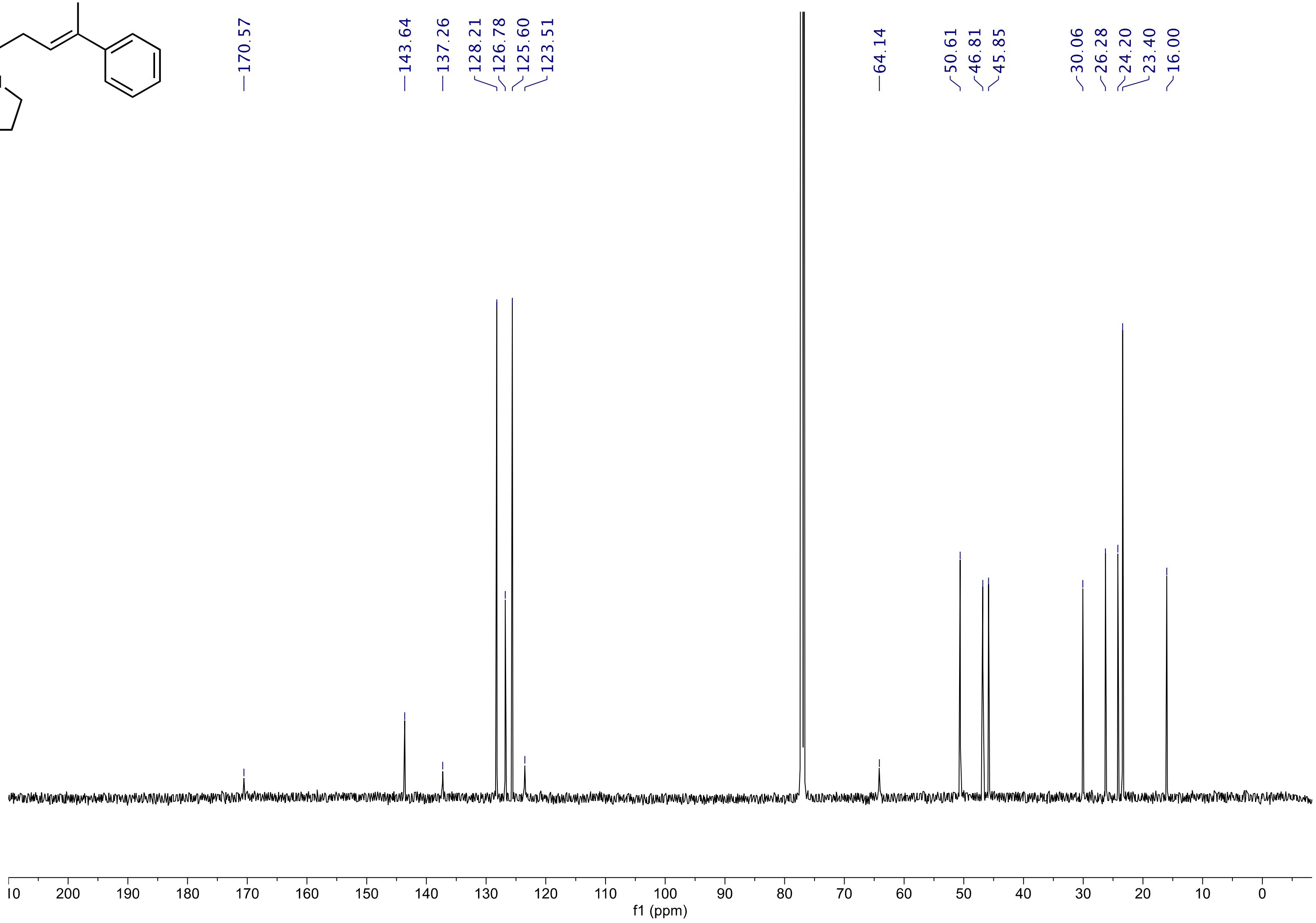

3u – ^{1}H NMR (500 MHz, $CDCl_3$)

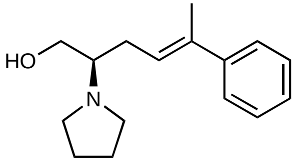

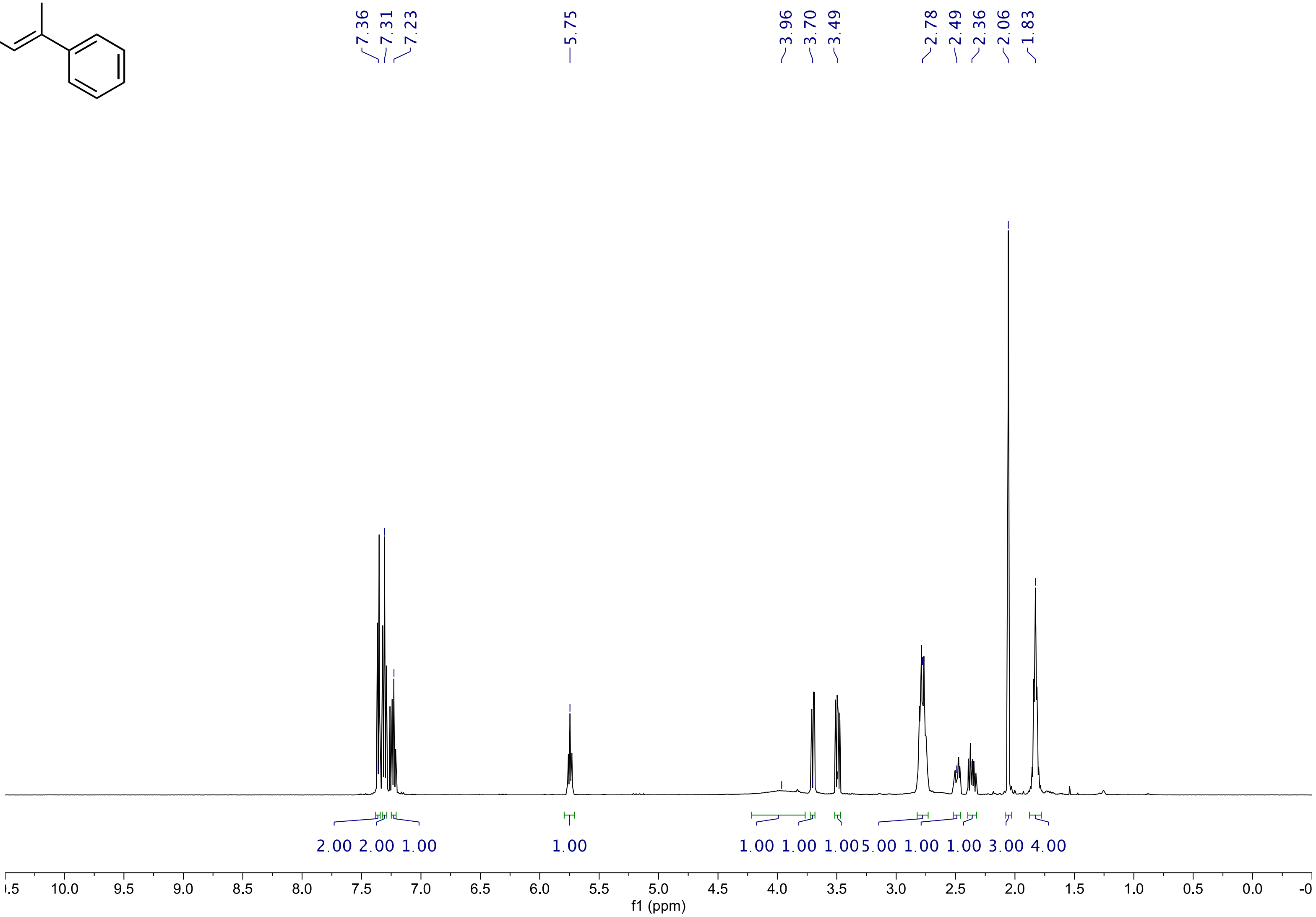

3u – ^{13}C NMR (126 MHz, $CDCl_3$)

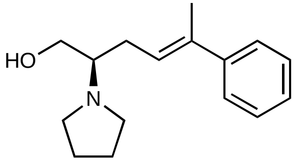

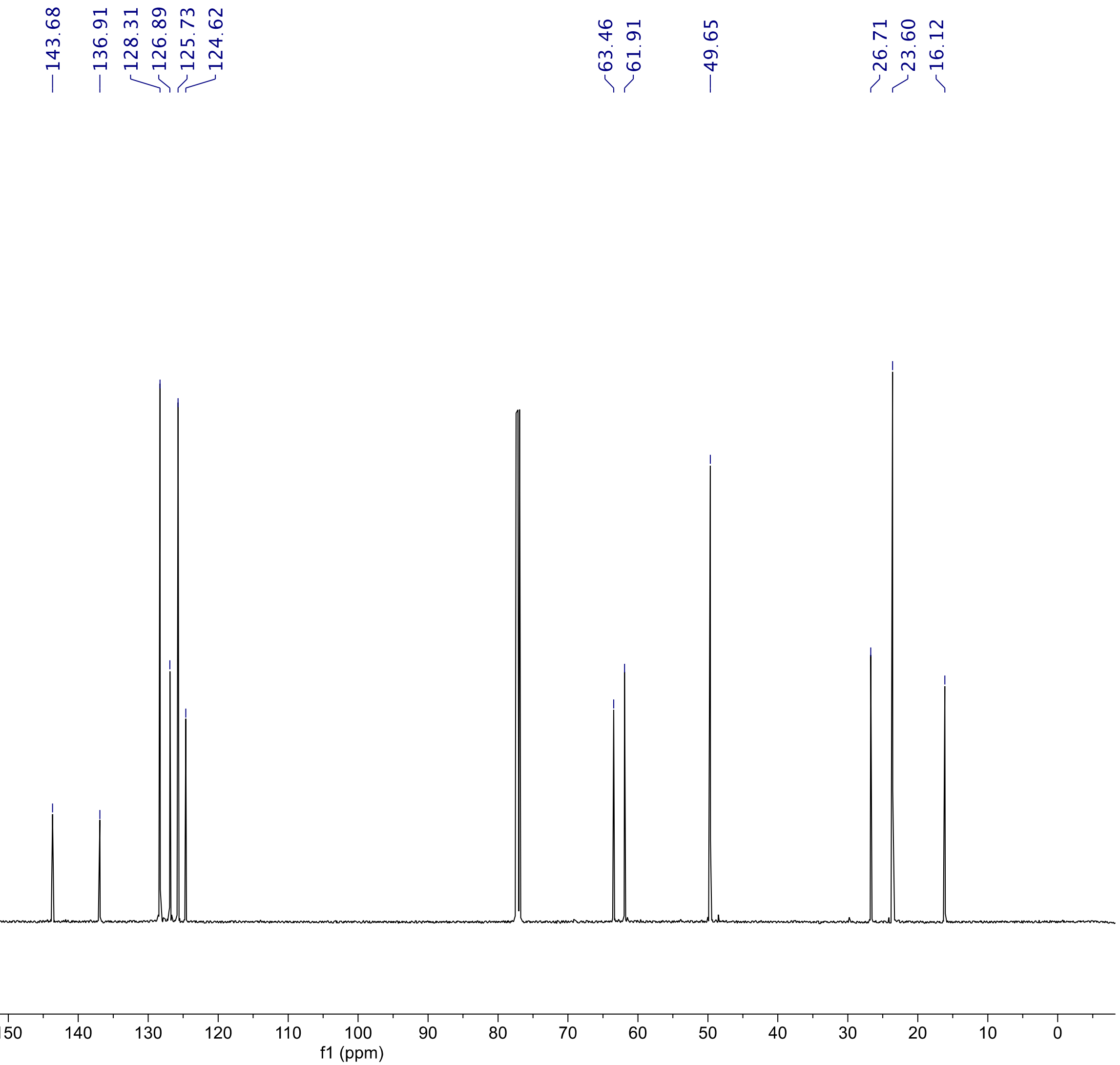

3v – ^{1}H NMR (500 MHz, $CDCl_3$)

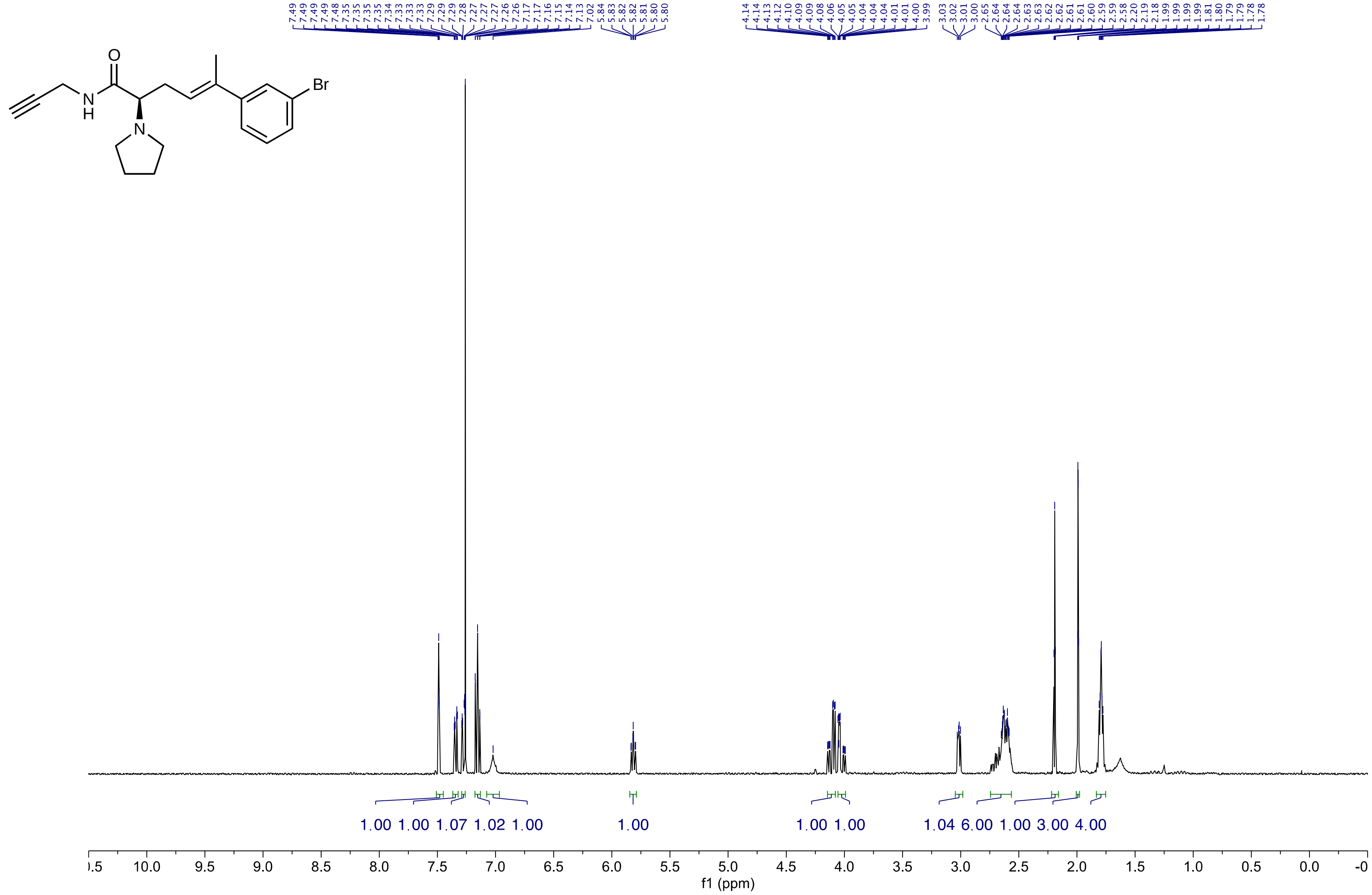

3v – ^{13}C NMR (126 MHz, $CDCl_3$)

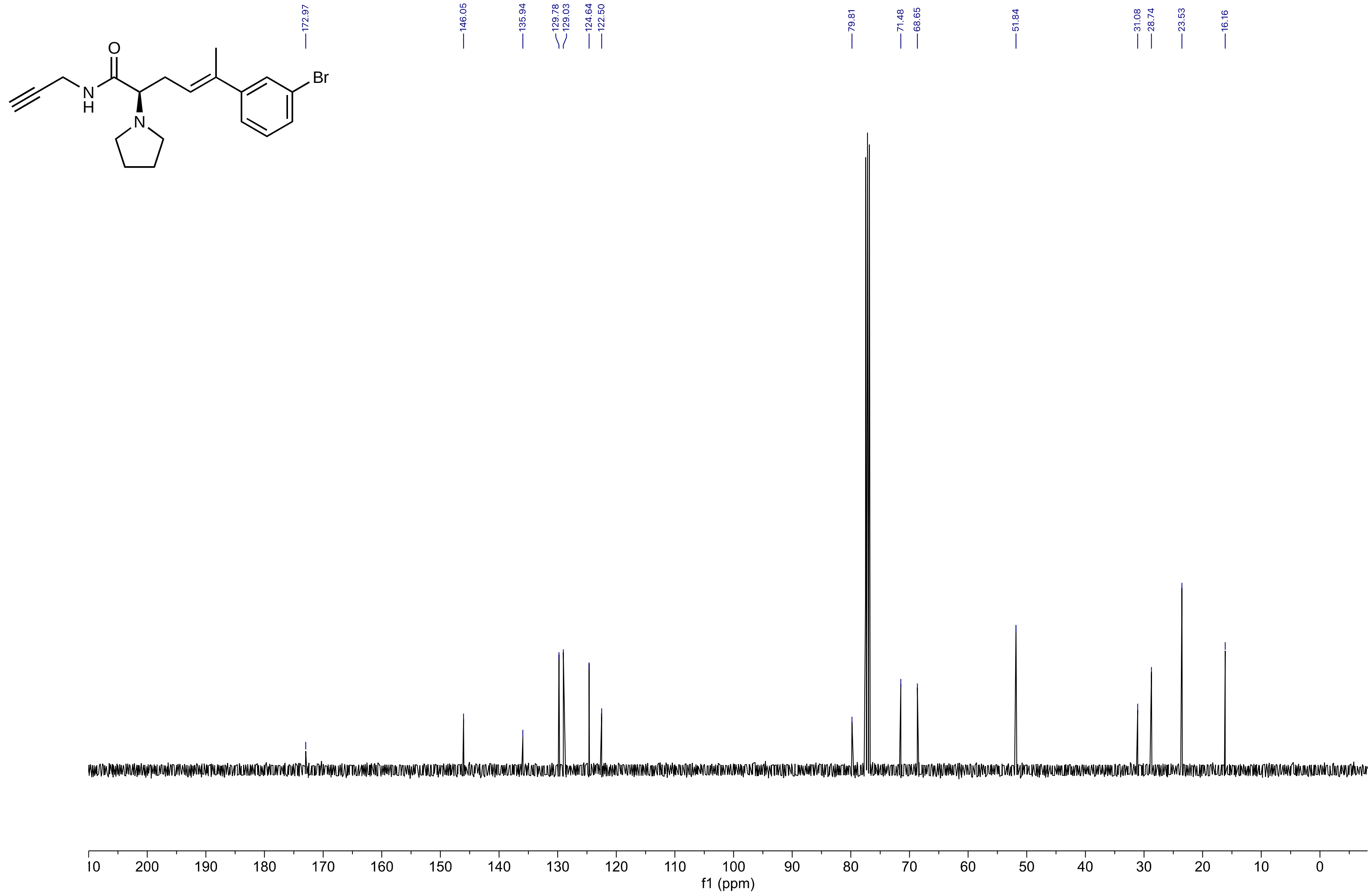

3w – ^{1}H NMR (500 MHz, $CDCl_3$)

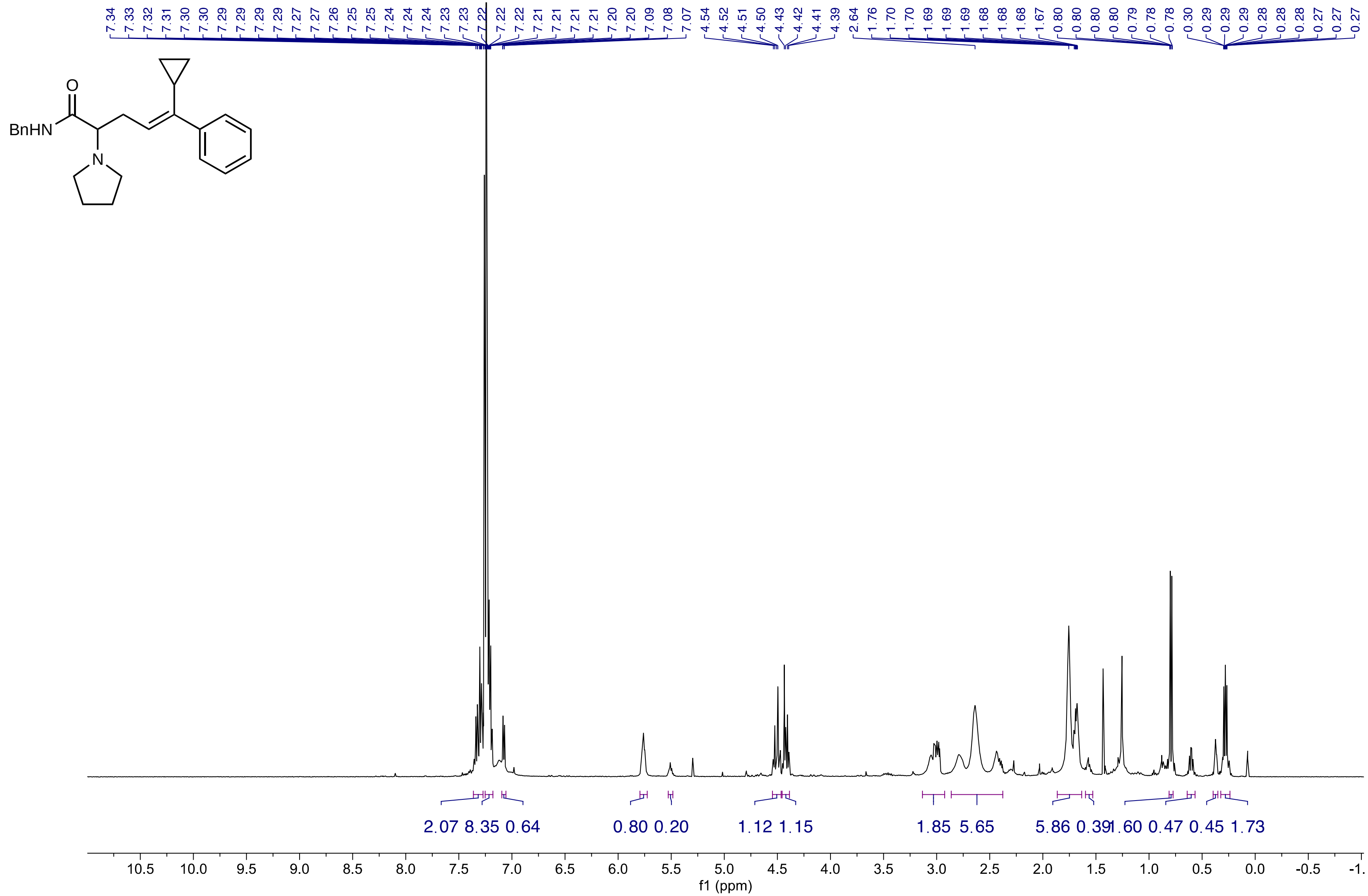

3w – ^{13}C NMR (126 MHz, $CDCl_3$)

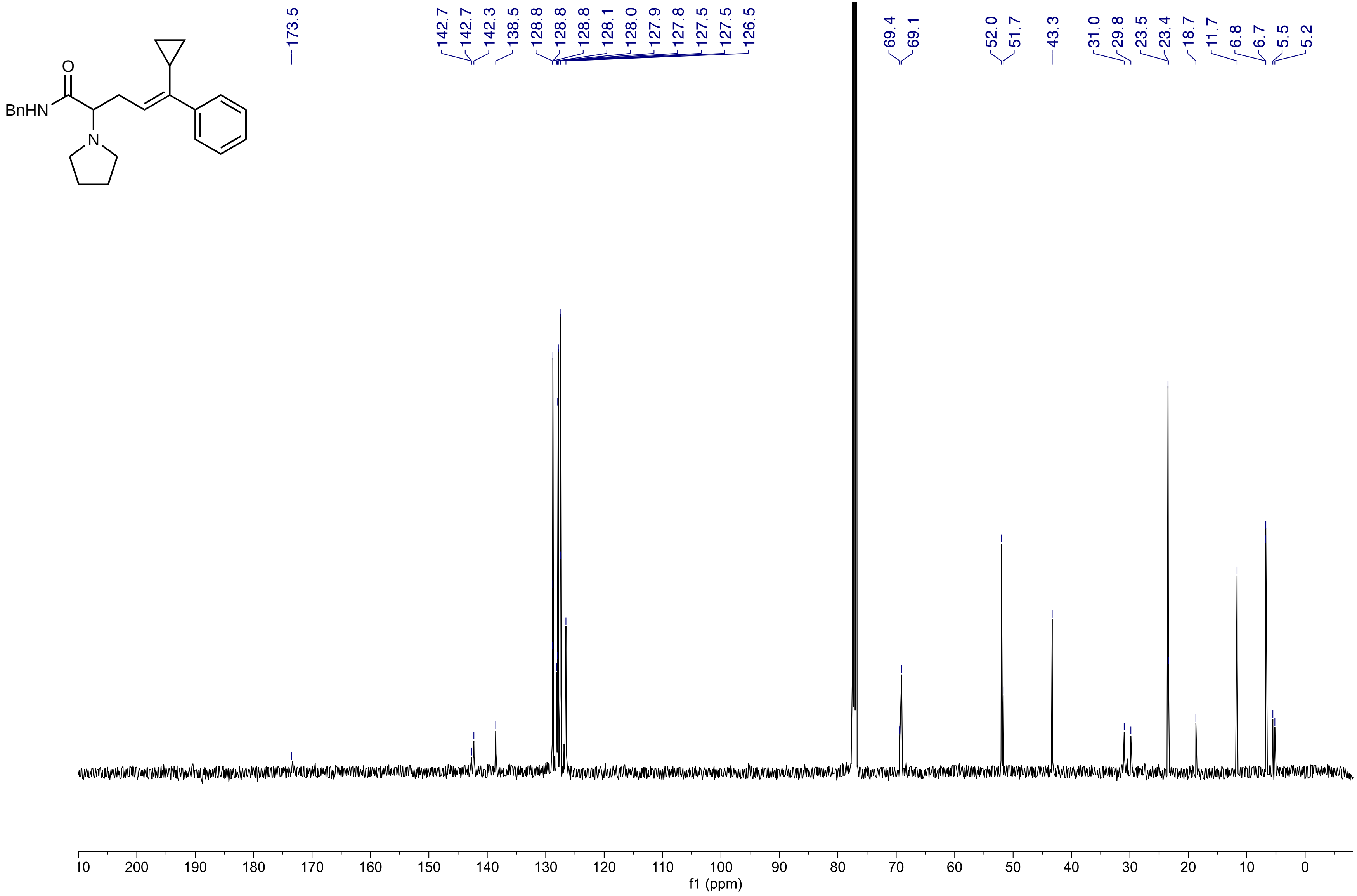

S61 – ^{1}H NMR (400 MHz, $CDCl_3$)

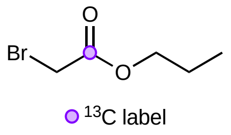

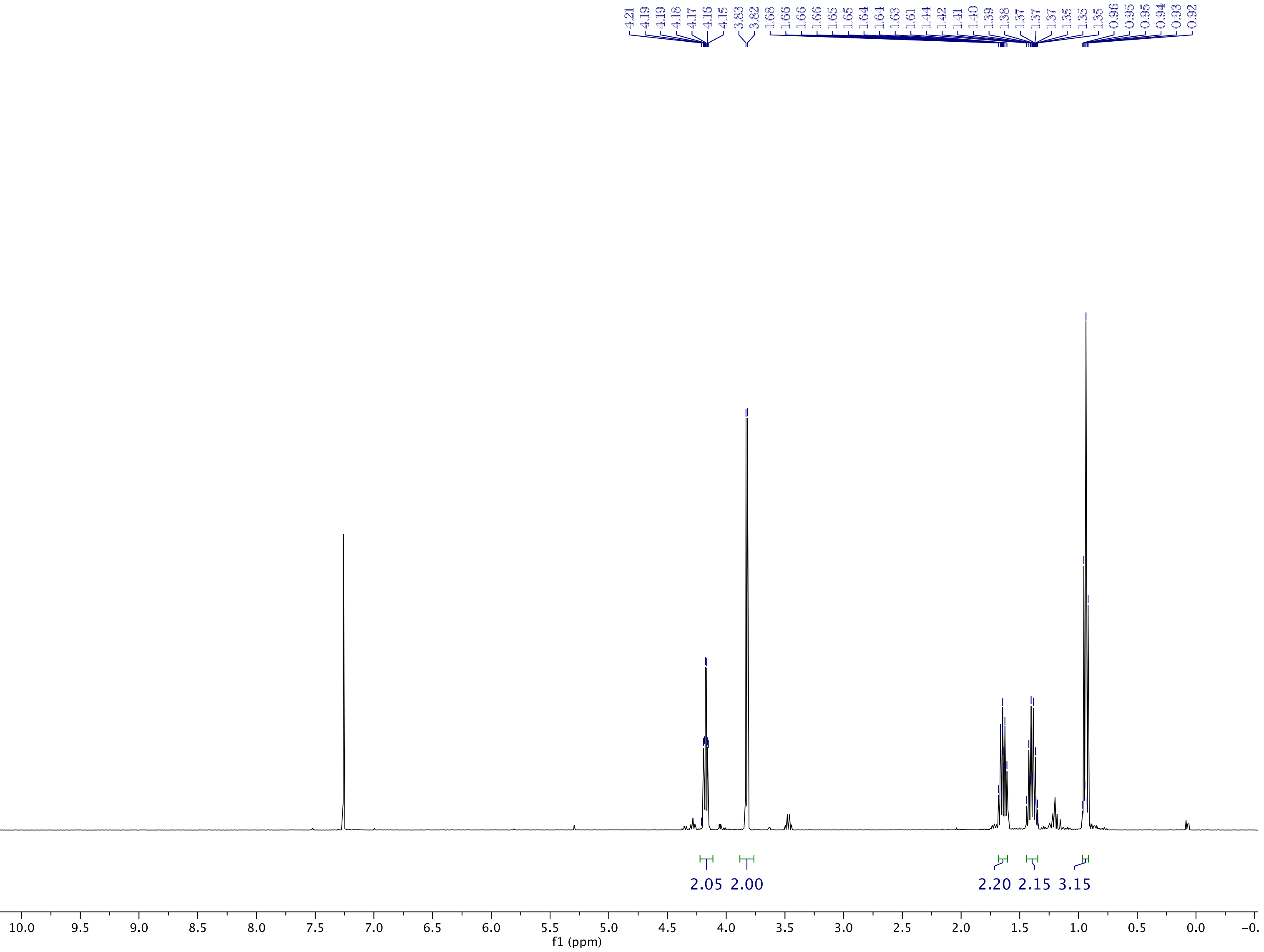

S61 – ^{13}C NMR (126 MHz, $CDCl_3$)

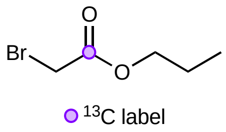

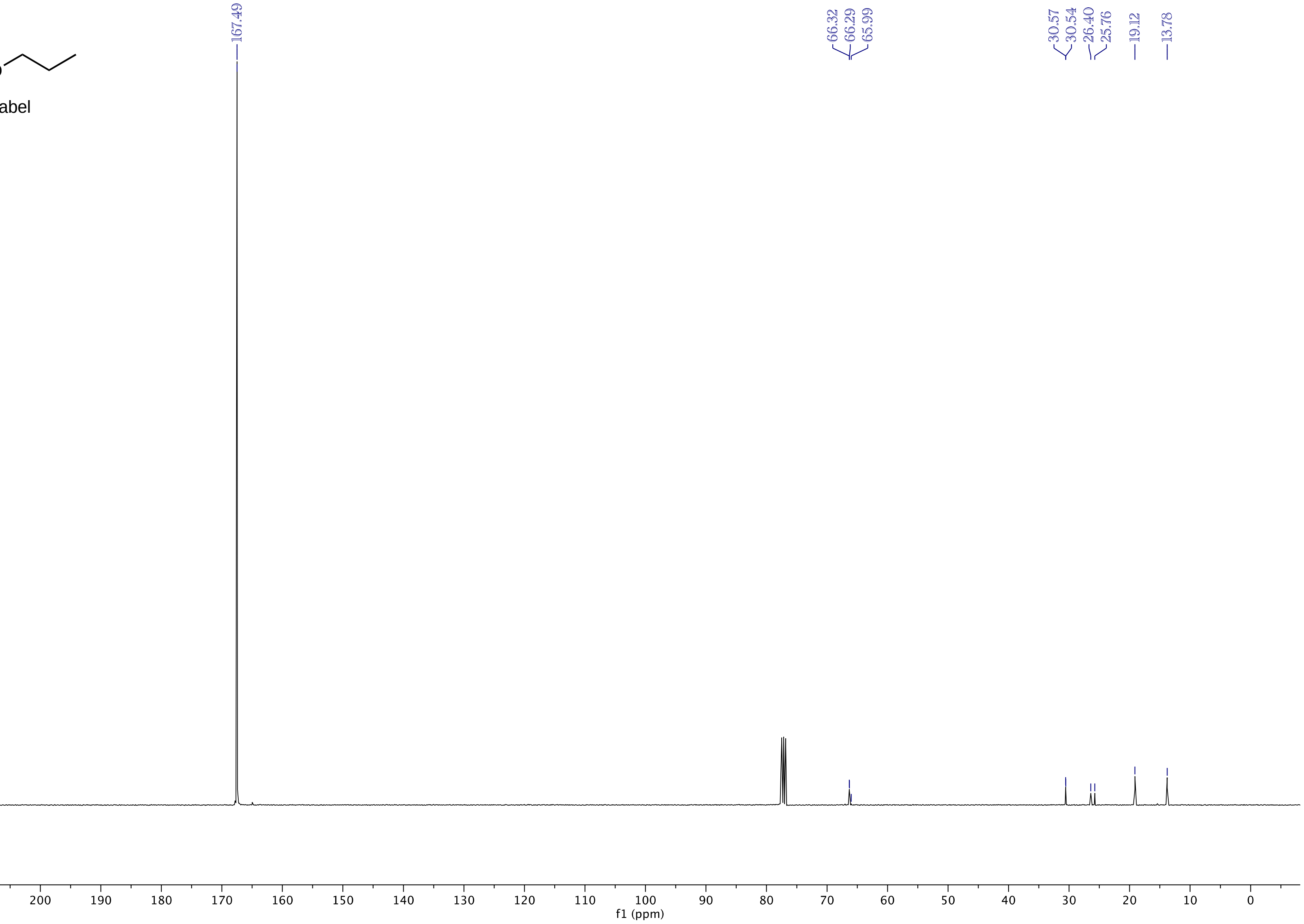

S62 – ^{1}H NMR (400 MHz, $CDCl_3$)

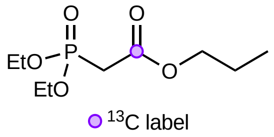

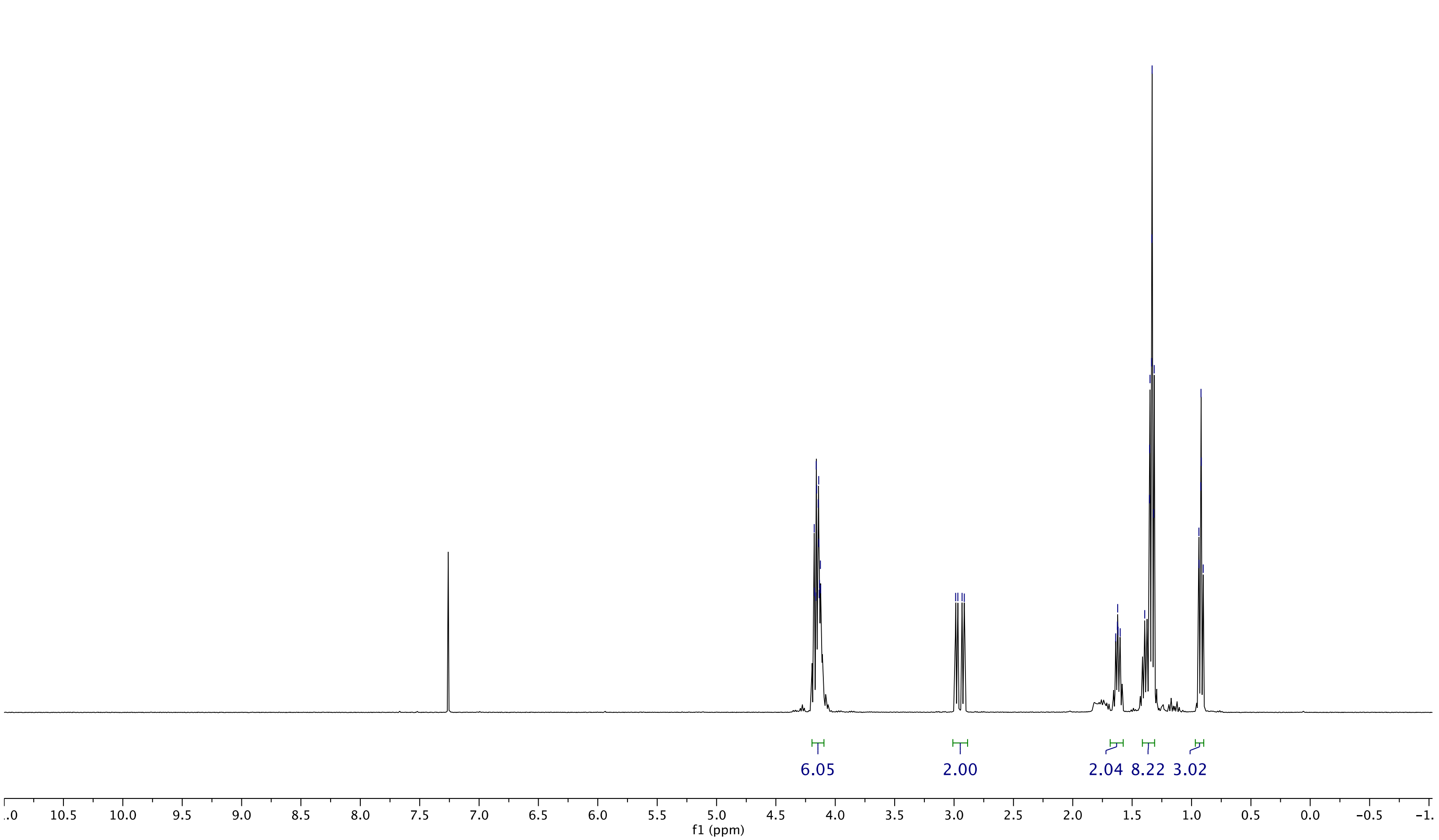

S62 – ^{13}C NMR (126 MHz, CDCl$_3$)

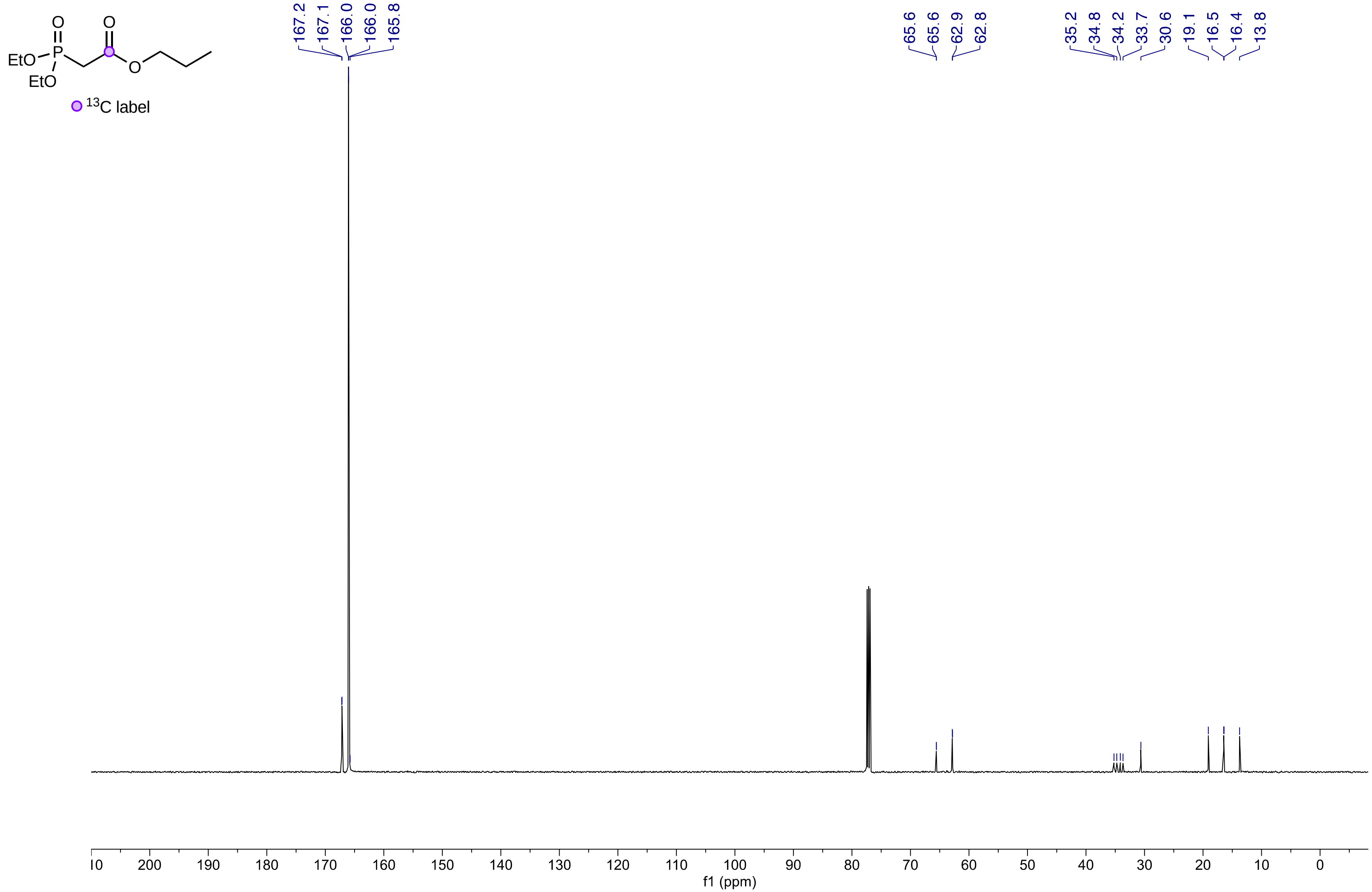

S63 – ^{1}H NMR (400 MHz, $CDCl_3$)

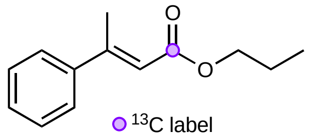

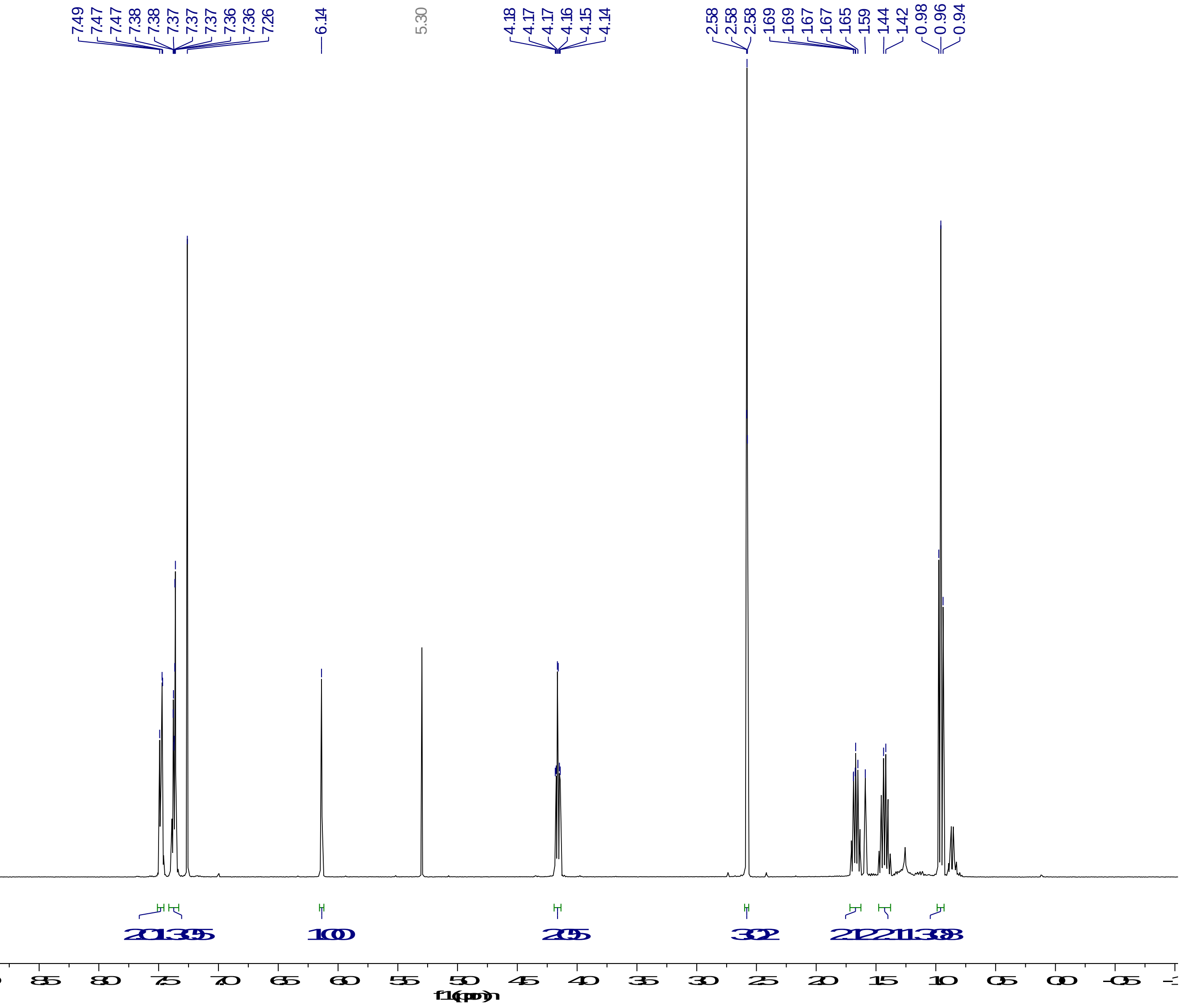

S63 – ^{13}C NMR (126 MHz, $CDCl_3$)

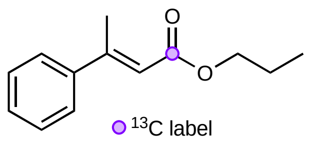

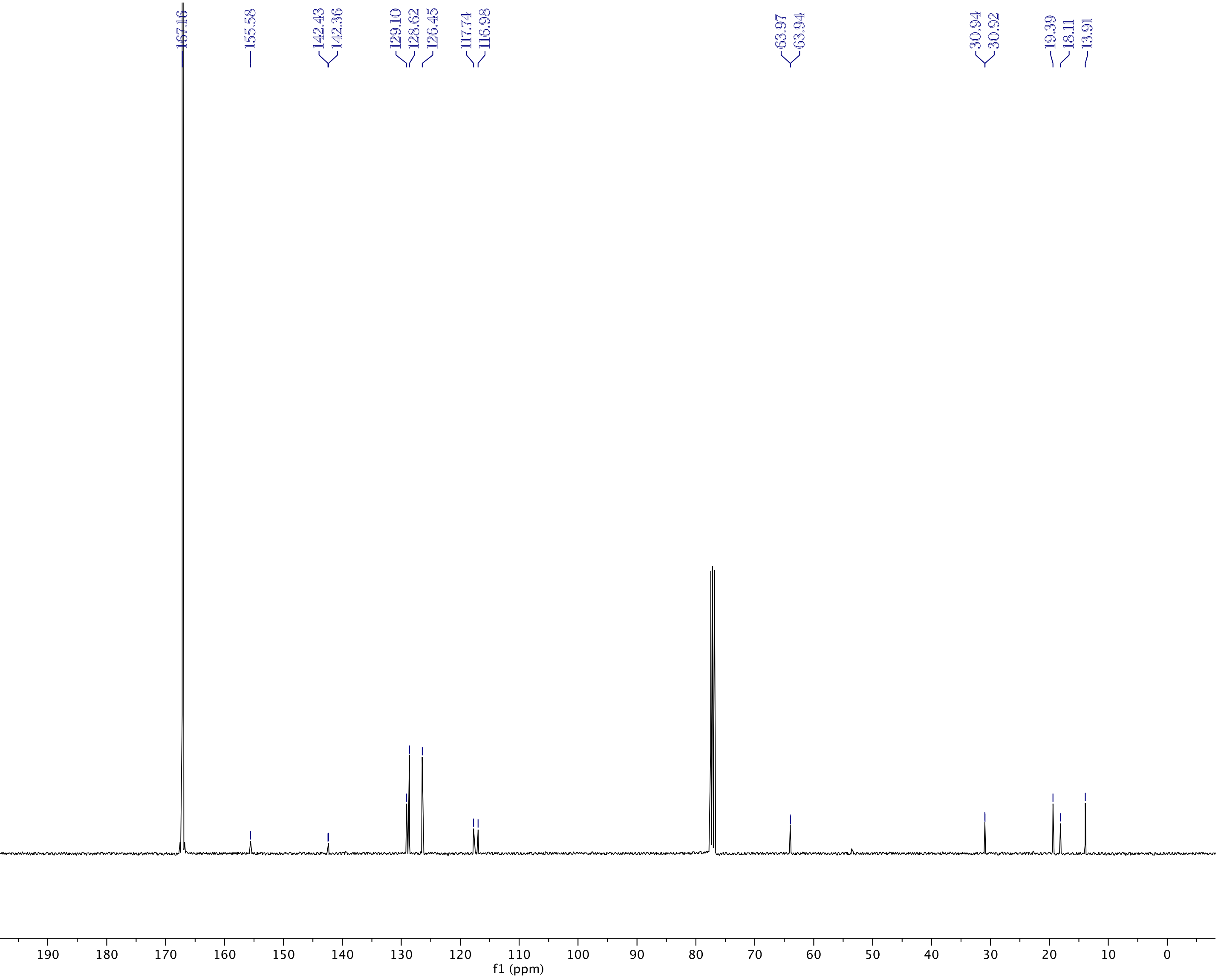

S64 – ^{1}H NMR (500 MHz, $CDCl_3$)

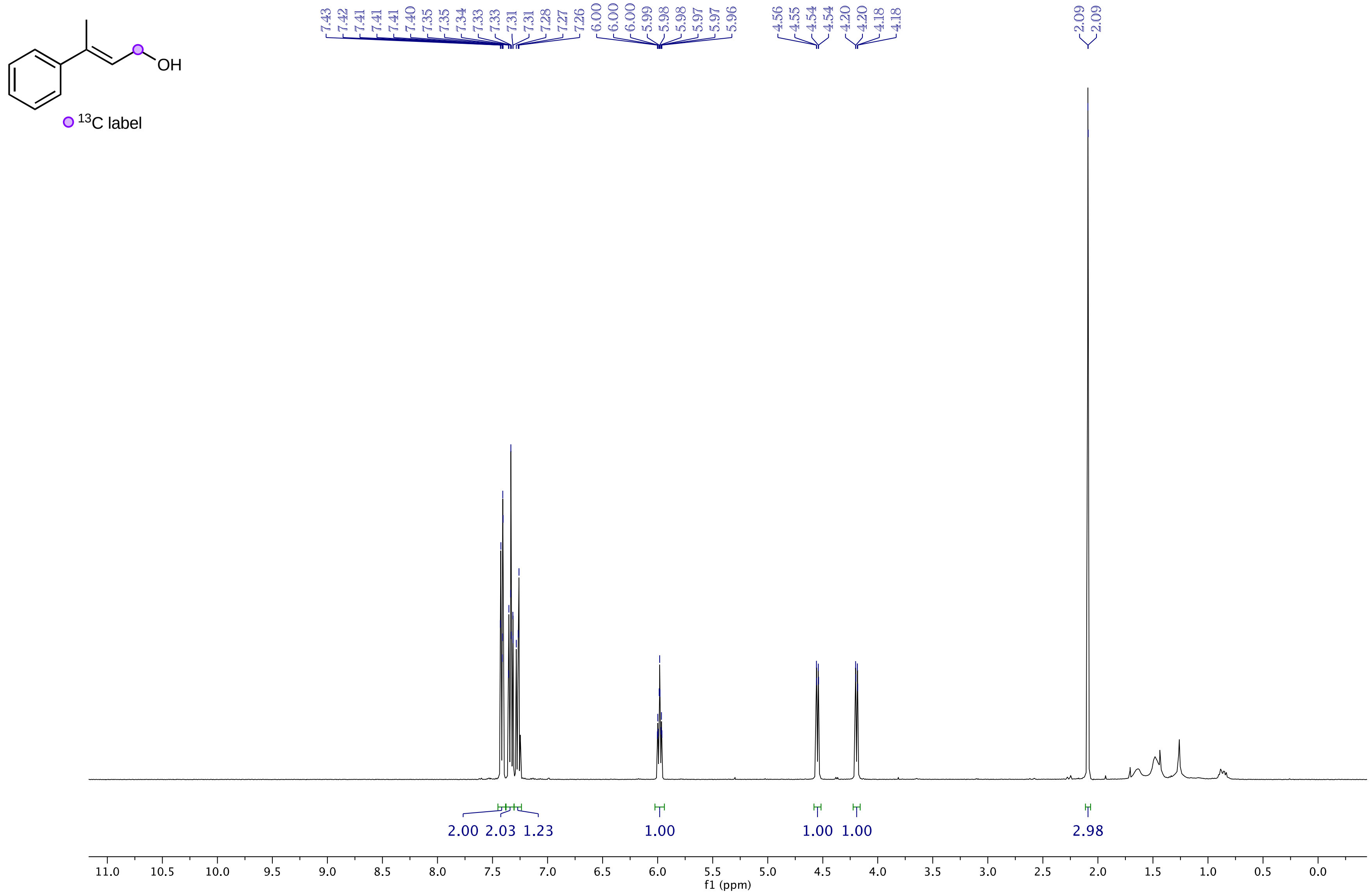

S64 – ^{13}C NMR (126 MHz, $CDCl_3$)

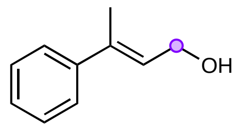

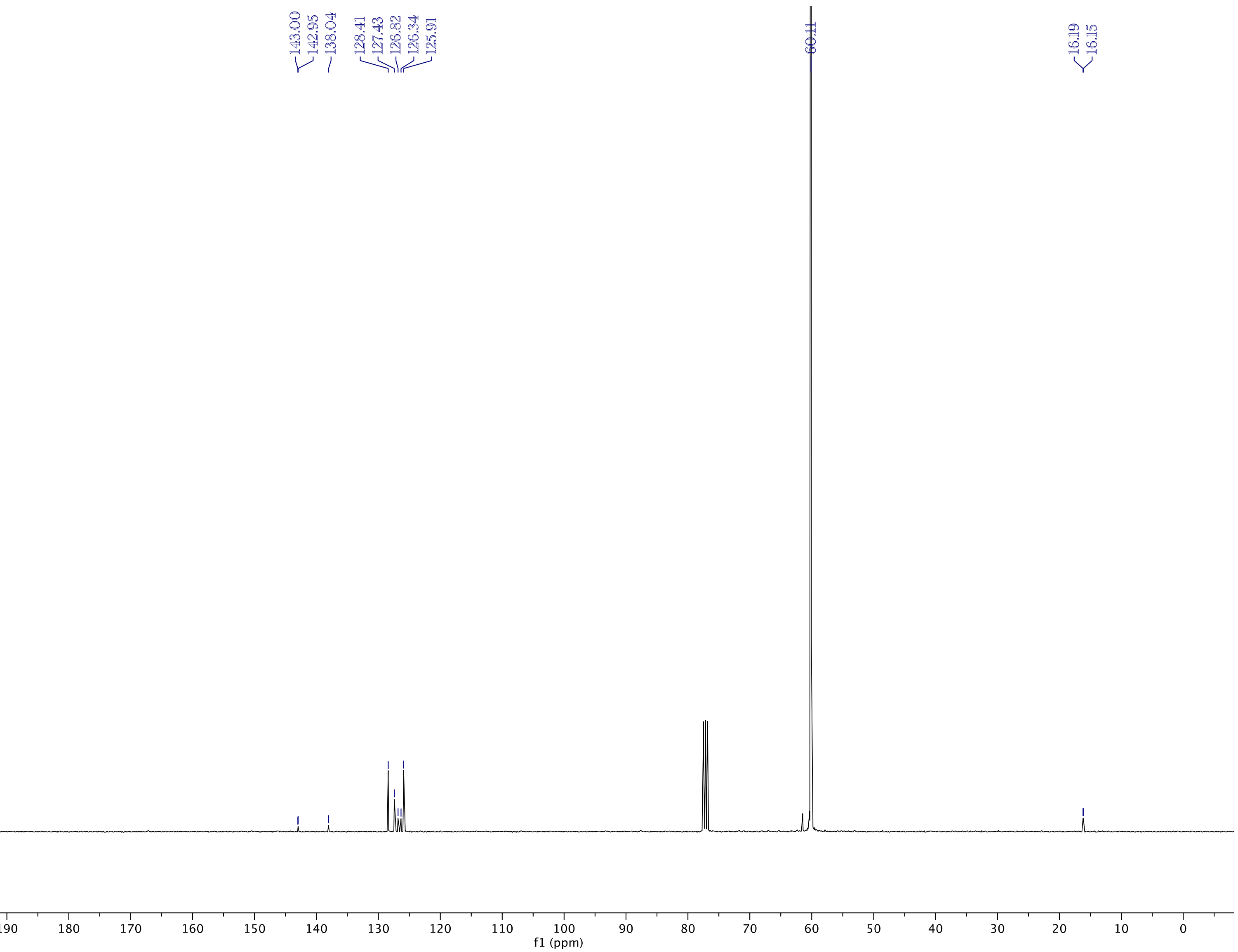

S65 – ^{1}H NMR (400 MHz, $CDCl_3$)

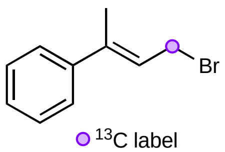

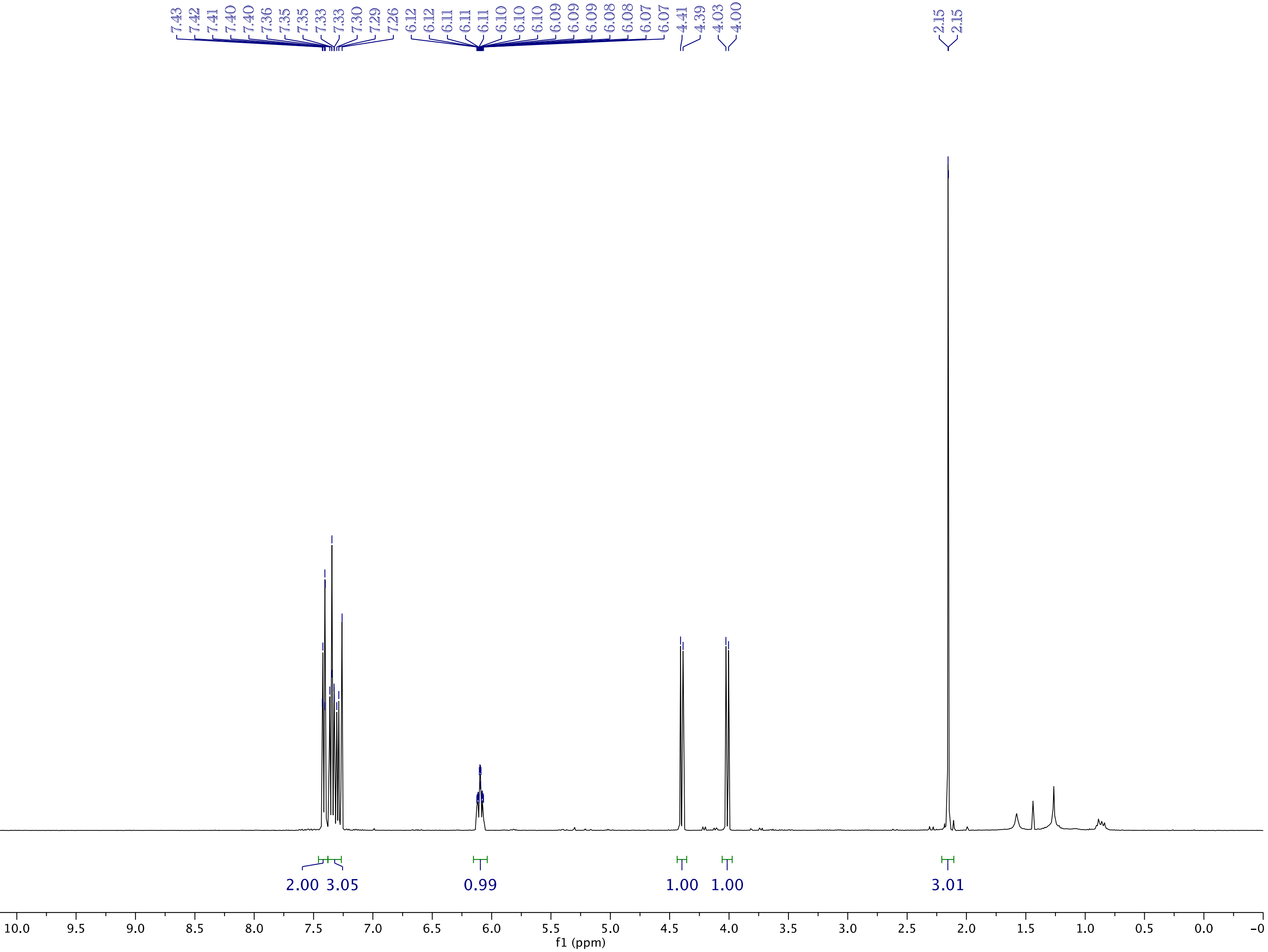

S65 – ^{13}C NMR (126 MHz, $CDCl_3$)

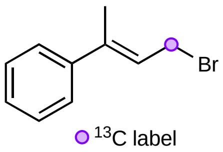

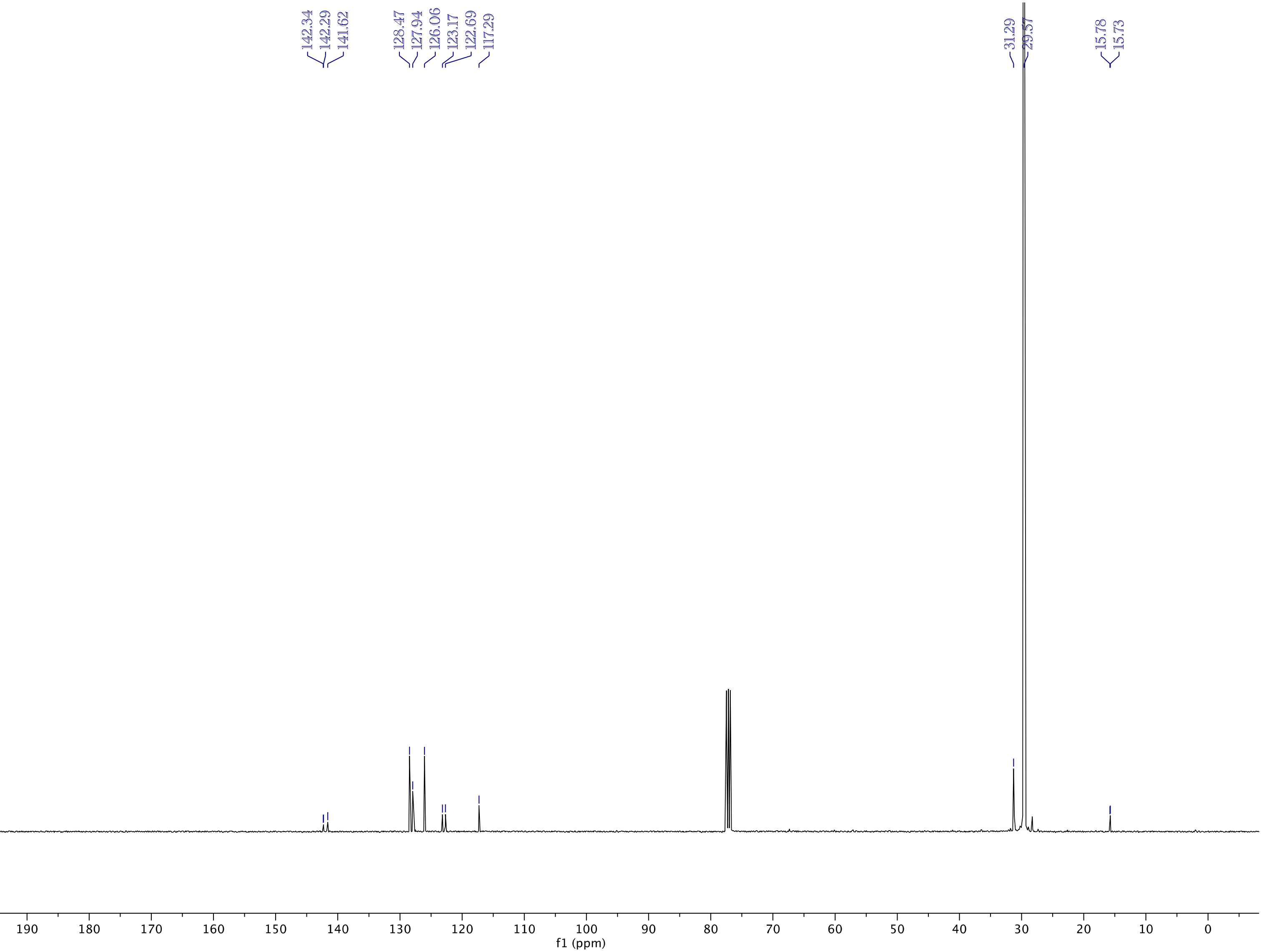

S66 – ^{1}H NMR (400 MHz, $CDCl_3$)

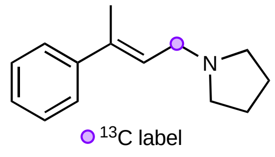

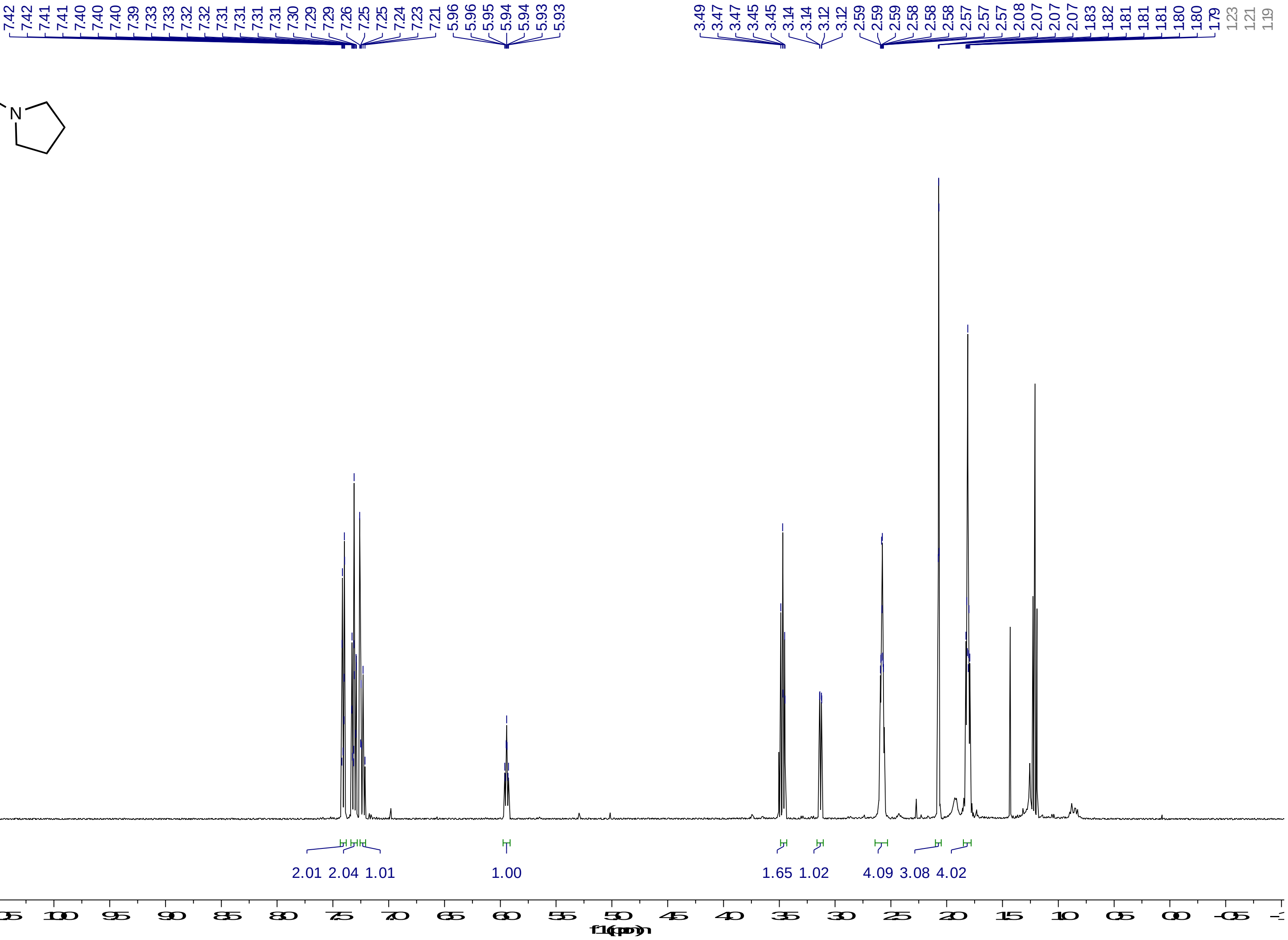

S66 - ^{13}C NMR (126 MHz, $CDCl_3$)

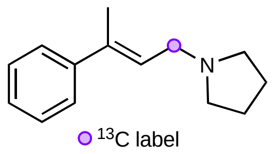

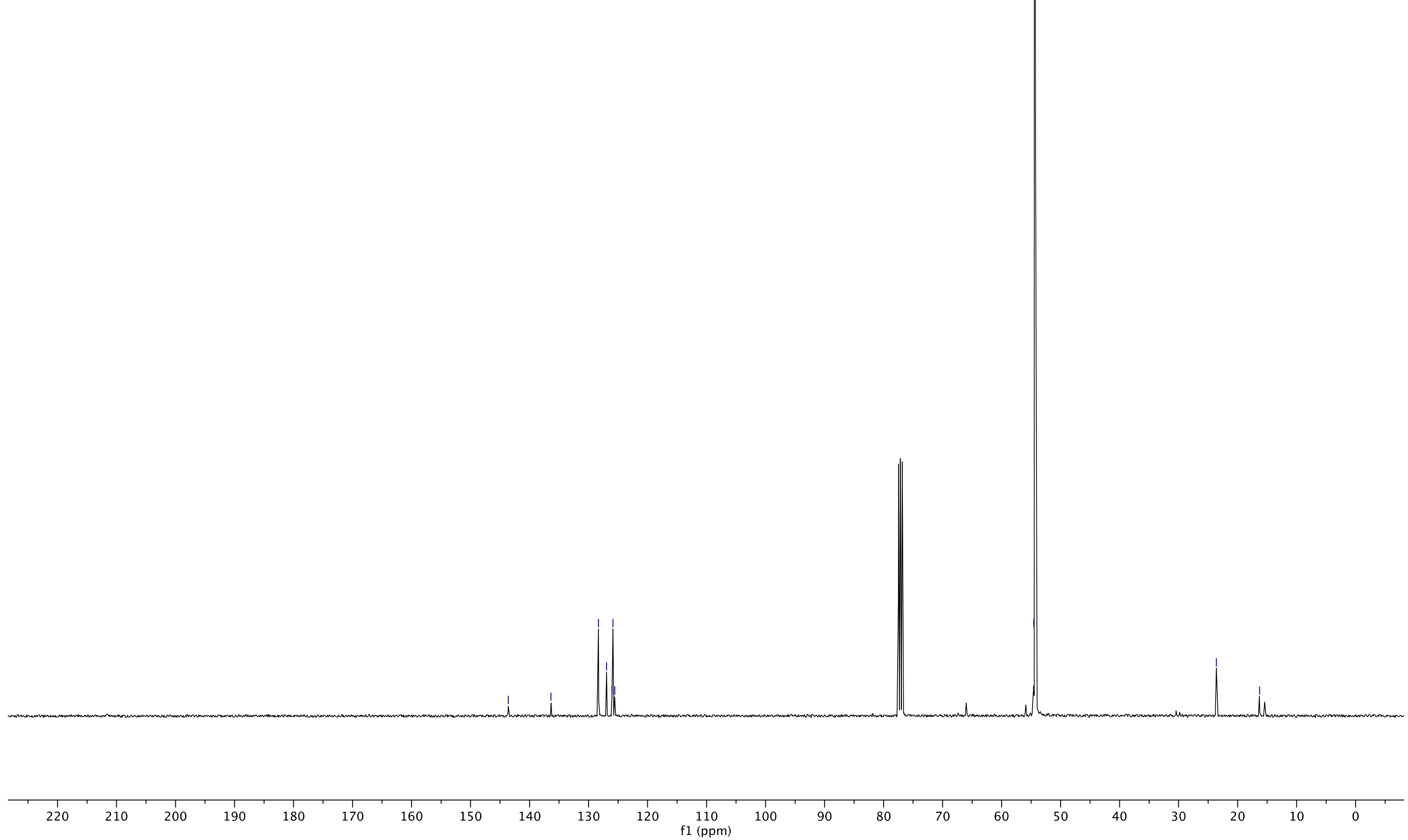

2'-[$^{13}C_1$]-**2a** – ^{1}H NMR (500 MHz, d6-DMSO)

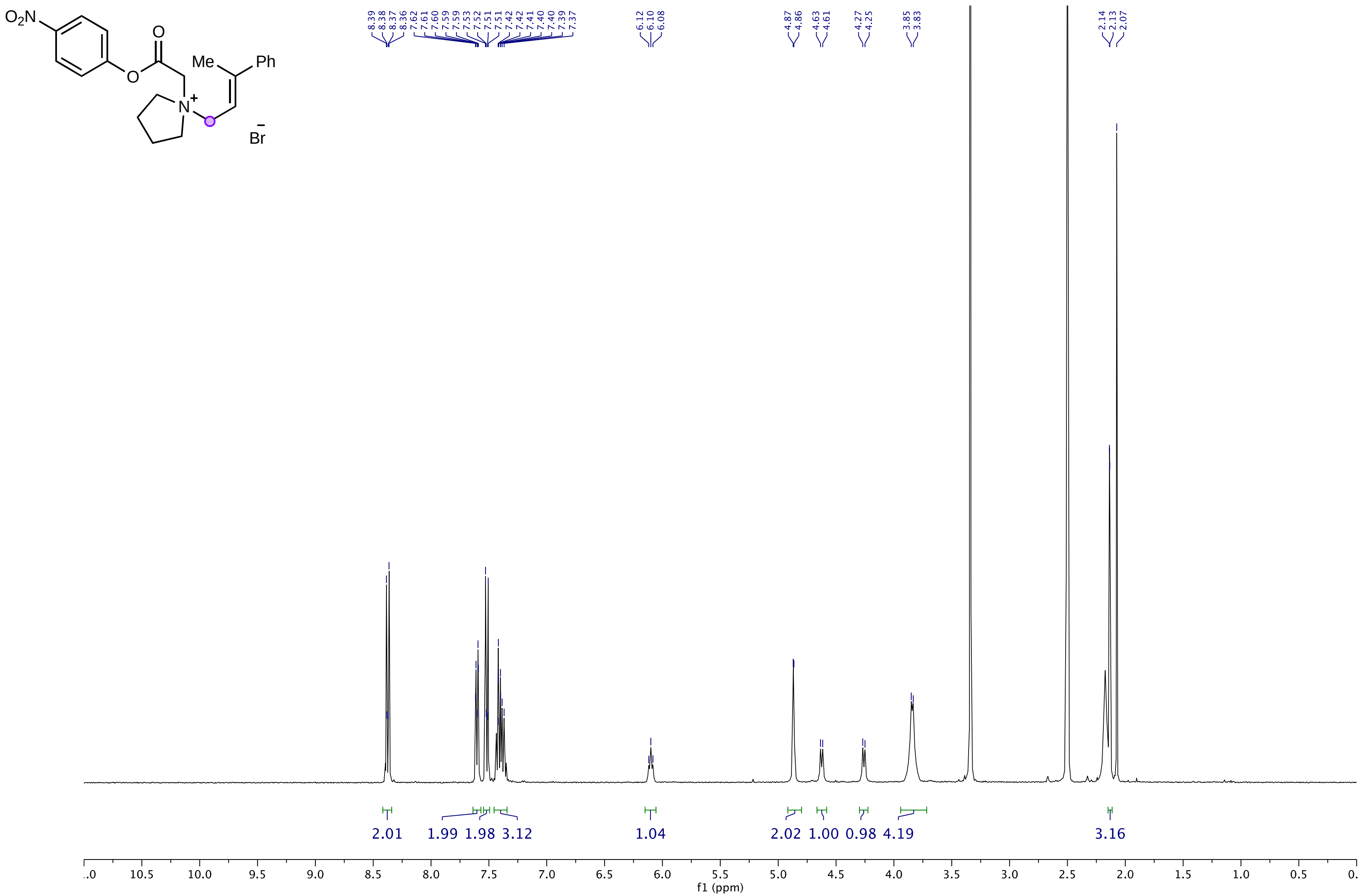

2’-[$^{13}C_1$]-**2a** – ^{1}H NMR (500 MHz, d6-DMSO)

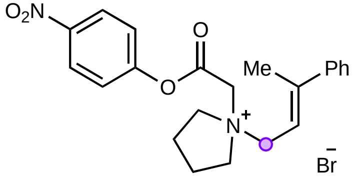

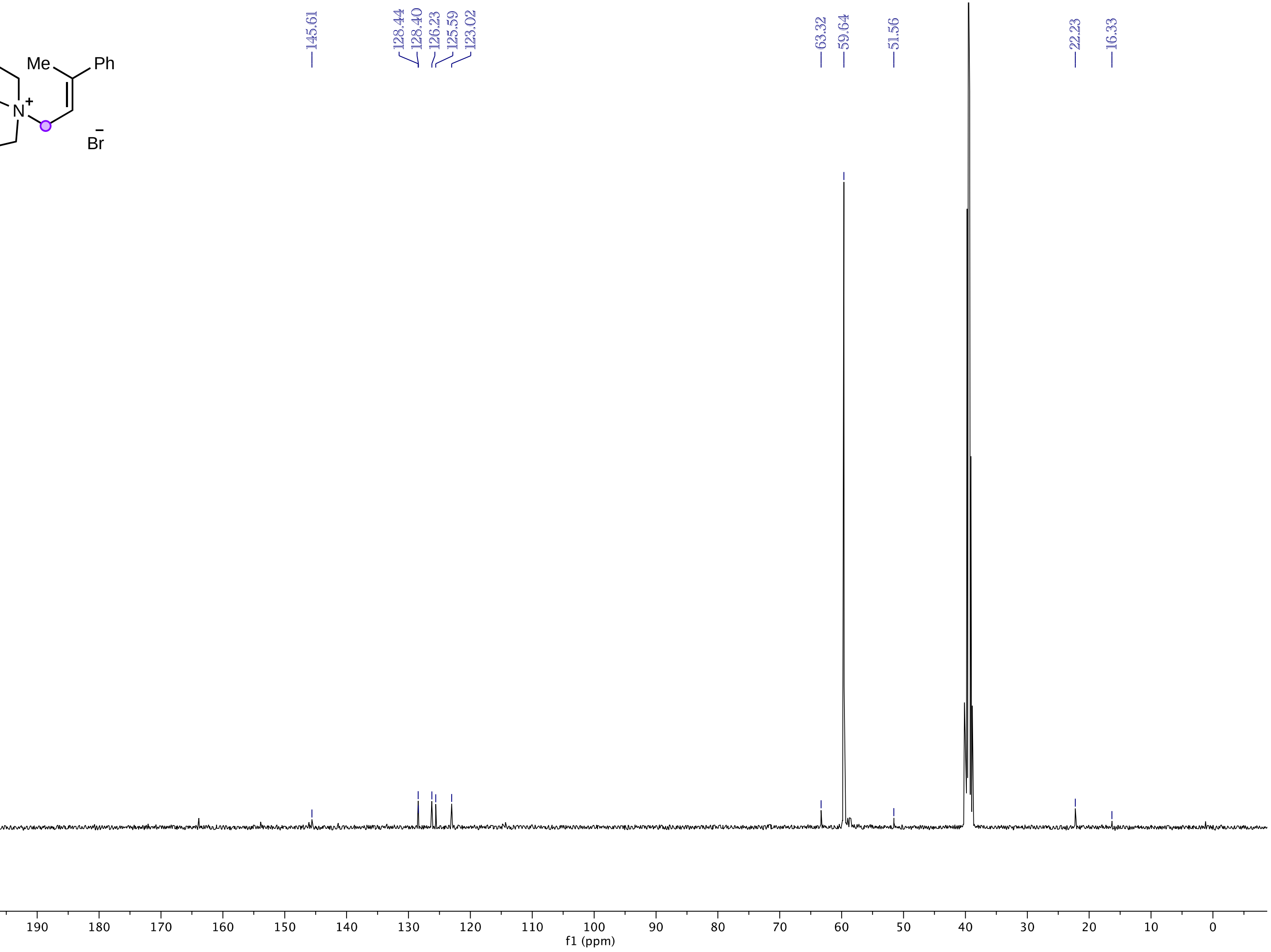

2-[$^{13}C_1$]-**2a** – 1H NMR (400 MHz, d6-DMSO)

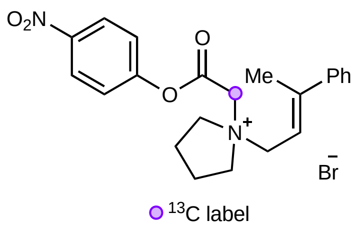

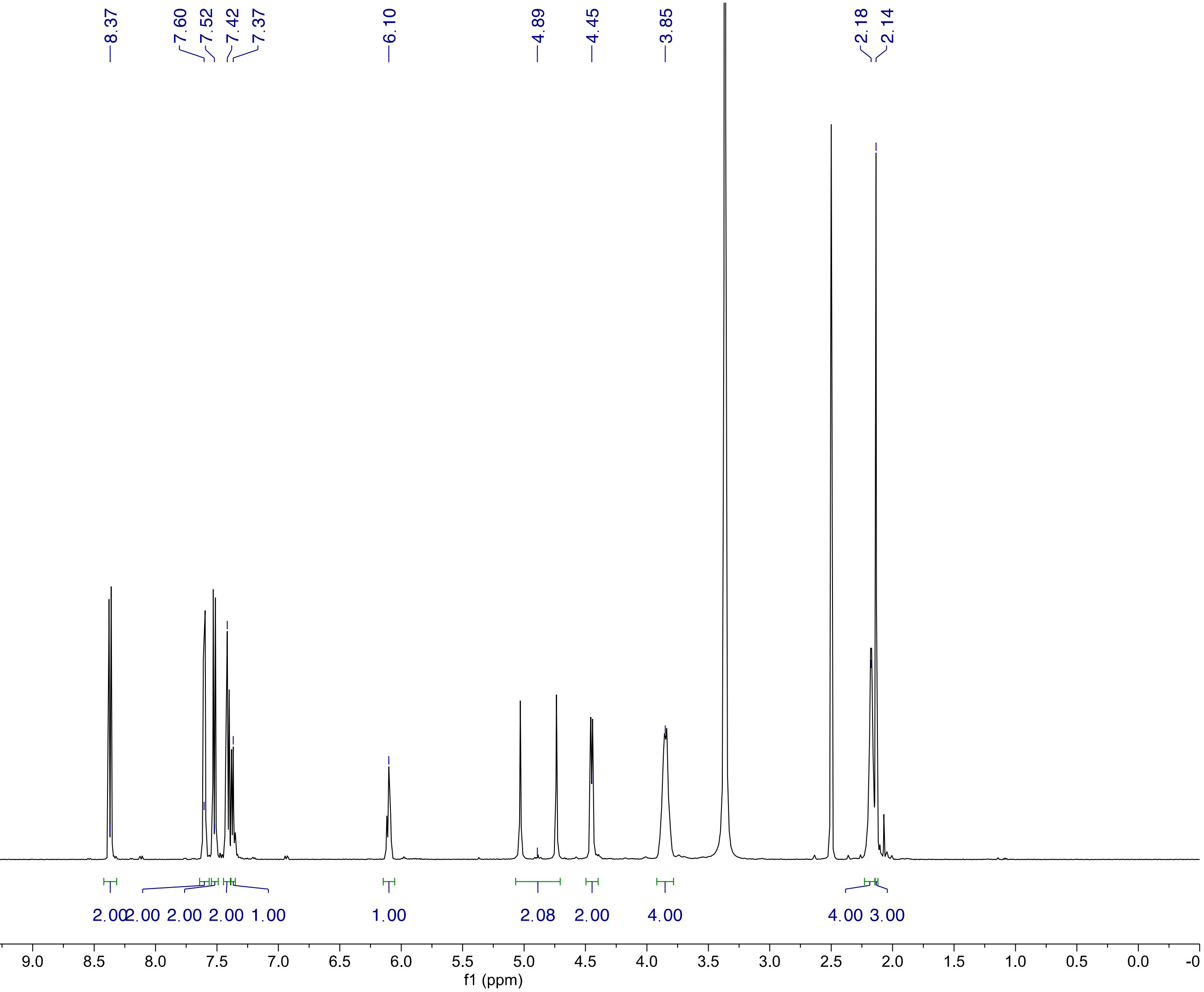

2-[$^{13}C_1$]-**2a** – ^{13}C NMR (126 MHz, d6-DMSO)

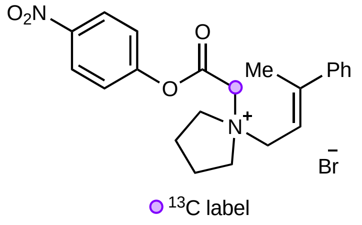

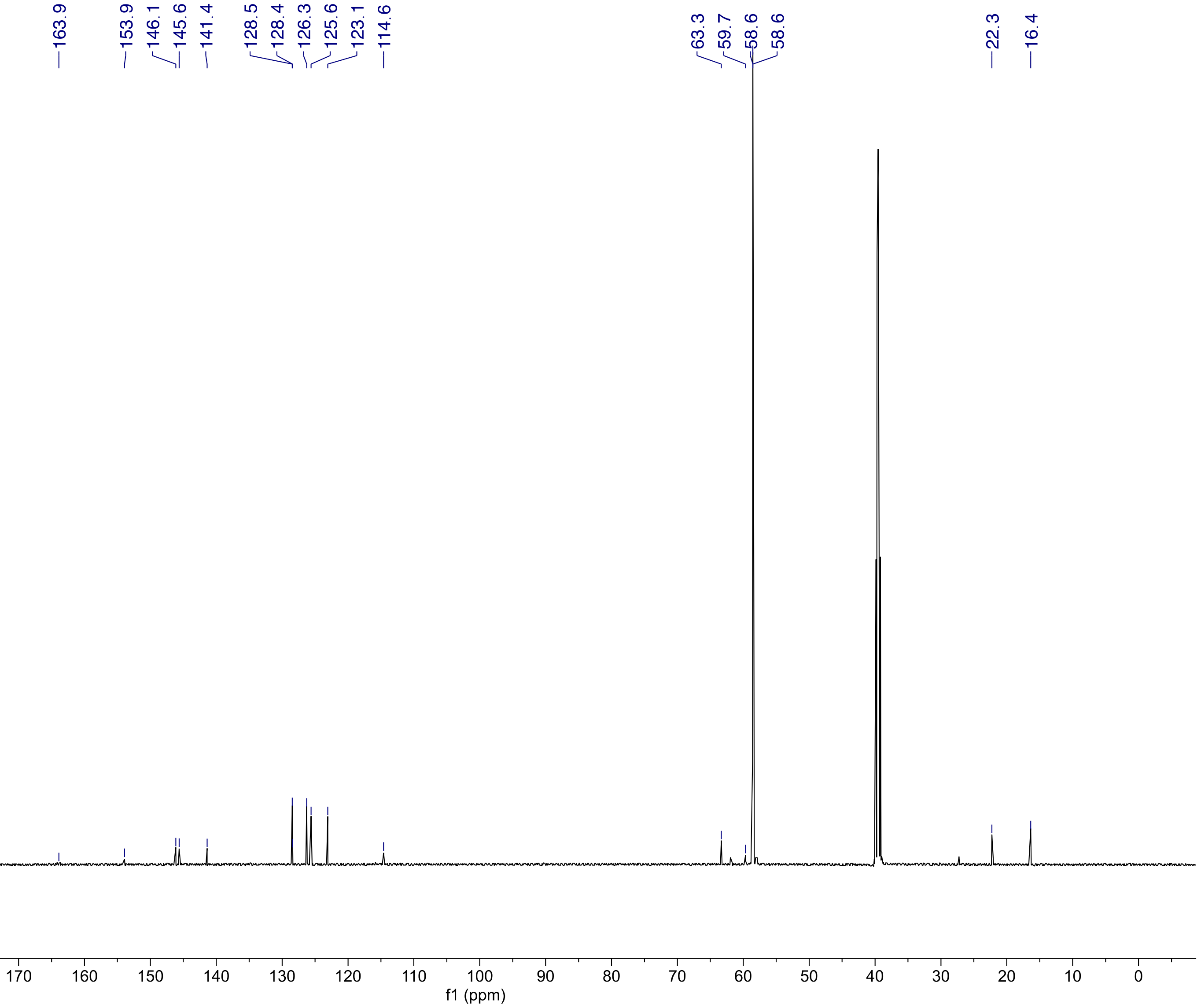

2,2’-[$^{13}C_2$]-**2a** – ^{1}H NMR (400 MHz, d6-DMSO)

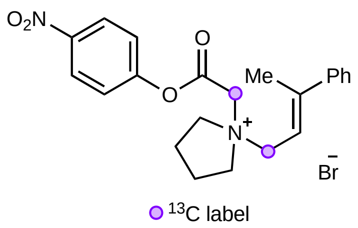

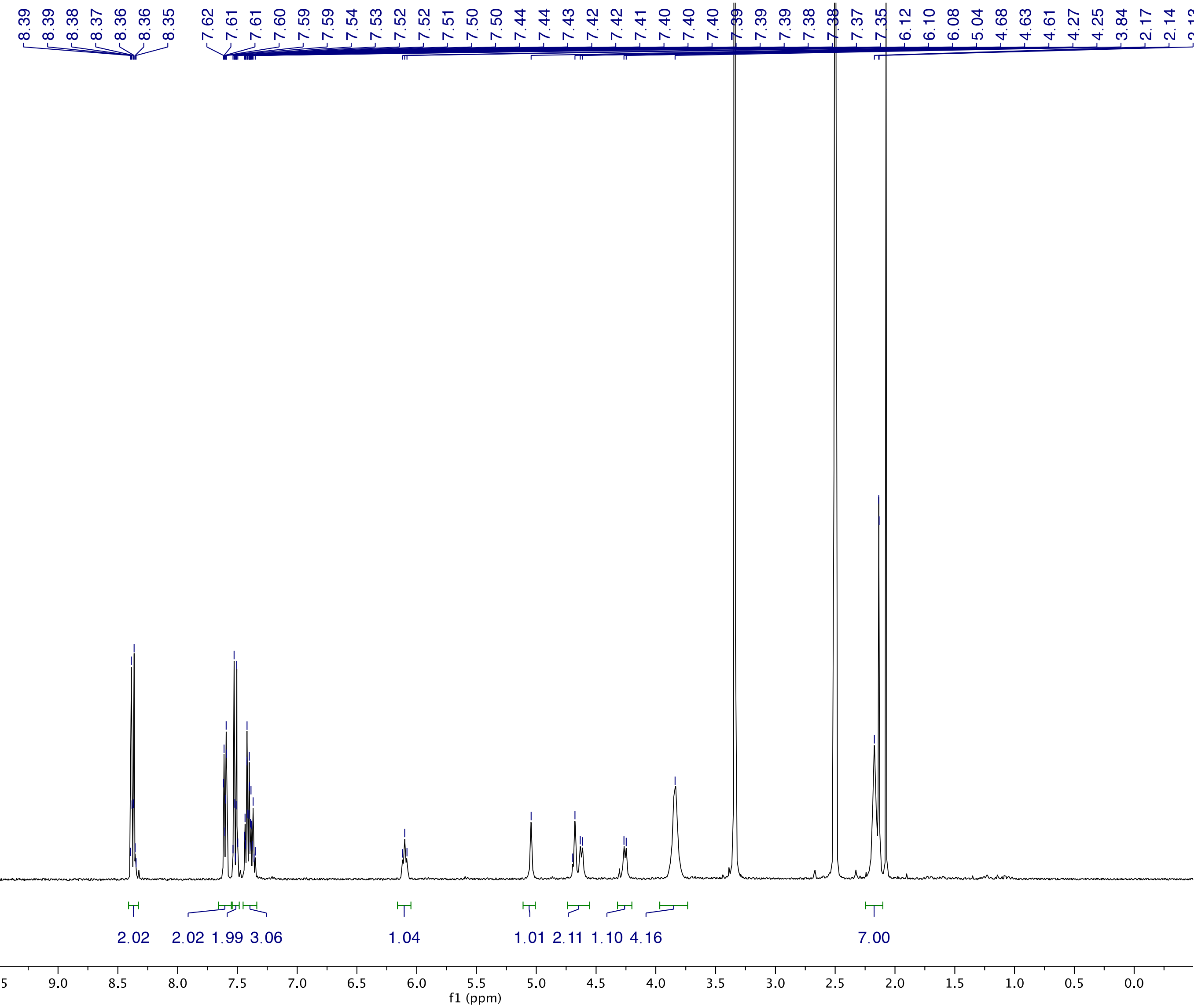

2,2’-[$^{13}C_2$]-**2a** – ^{13}C NMR (126 MHz, d6-DMSO)

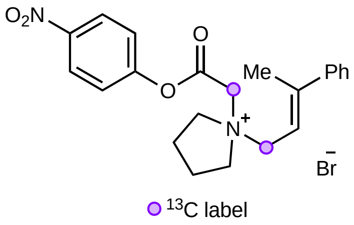

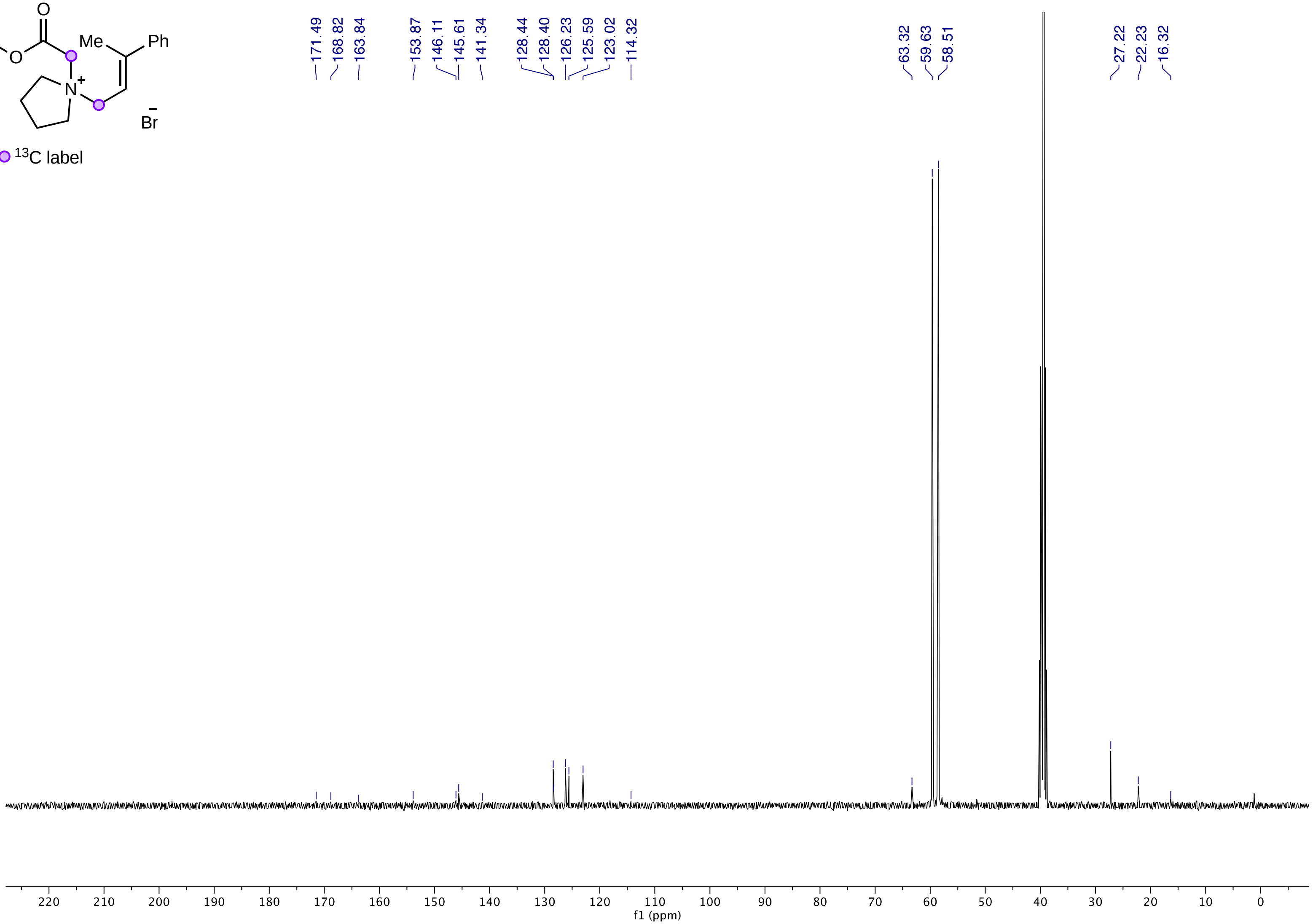

2-[$^{13}C_1$]-**3a** – ^{1}H NMR (500 MHz, $CDCl_3$)

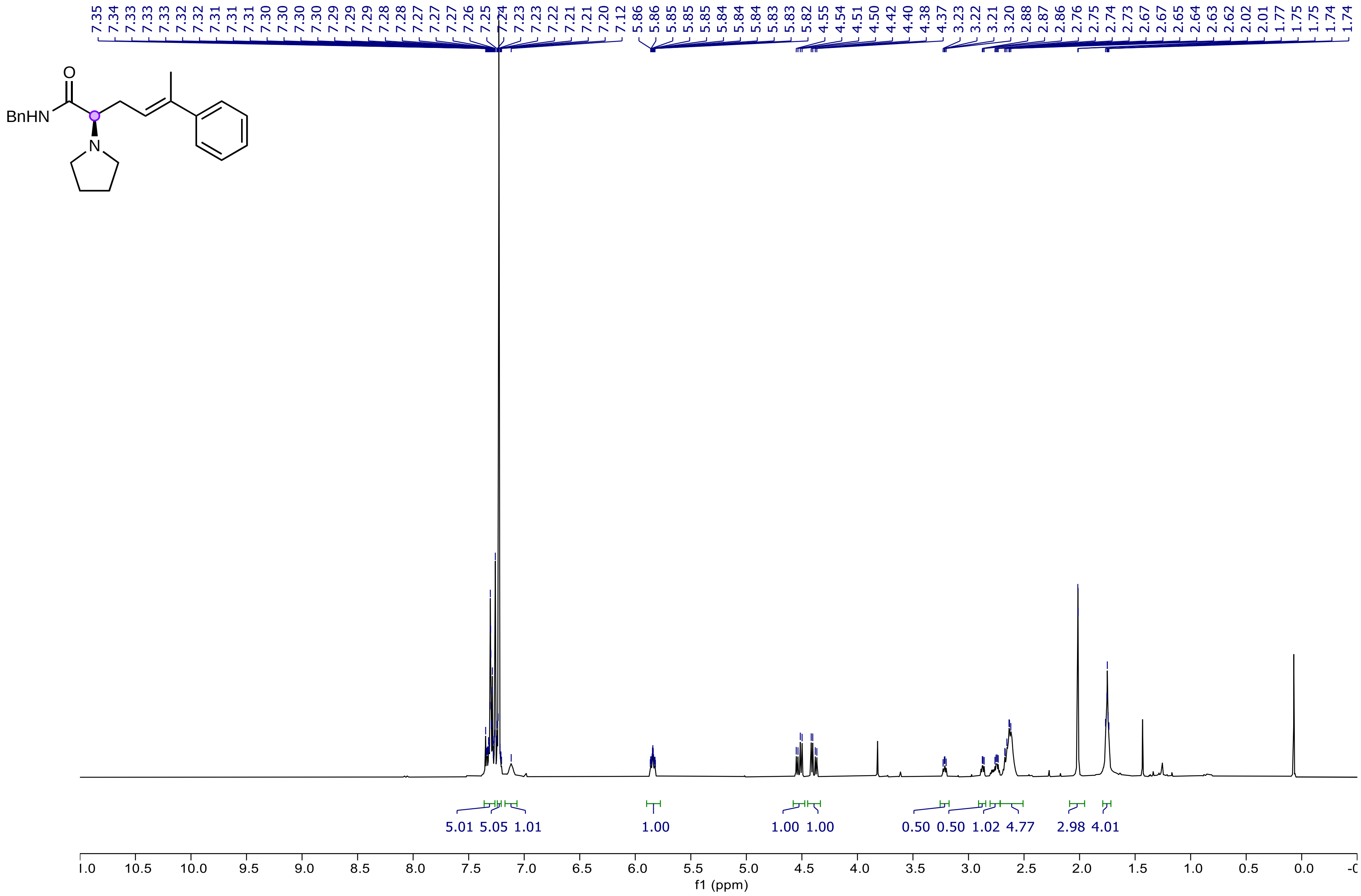

2-[$^{13}C_1$]-**3a** – ^{13}C NMR (126 MHz, $CDCl_3$)

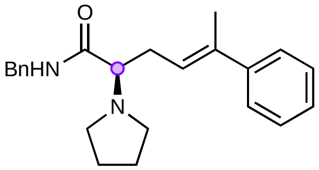

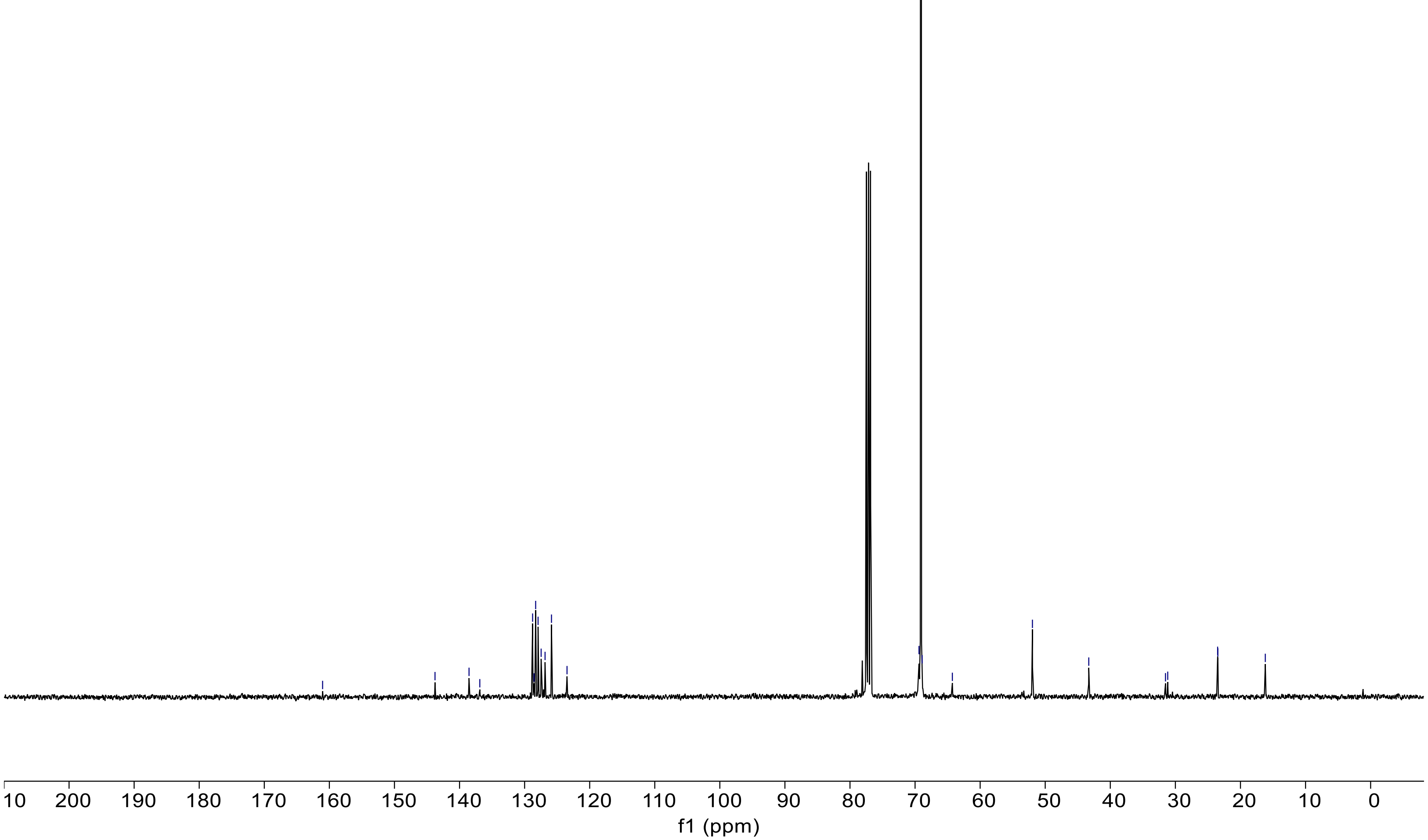

3-[$^{13}C_1$]-**3a** – ^{1}H NMR (500 MHz, $CDCl_3$)

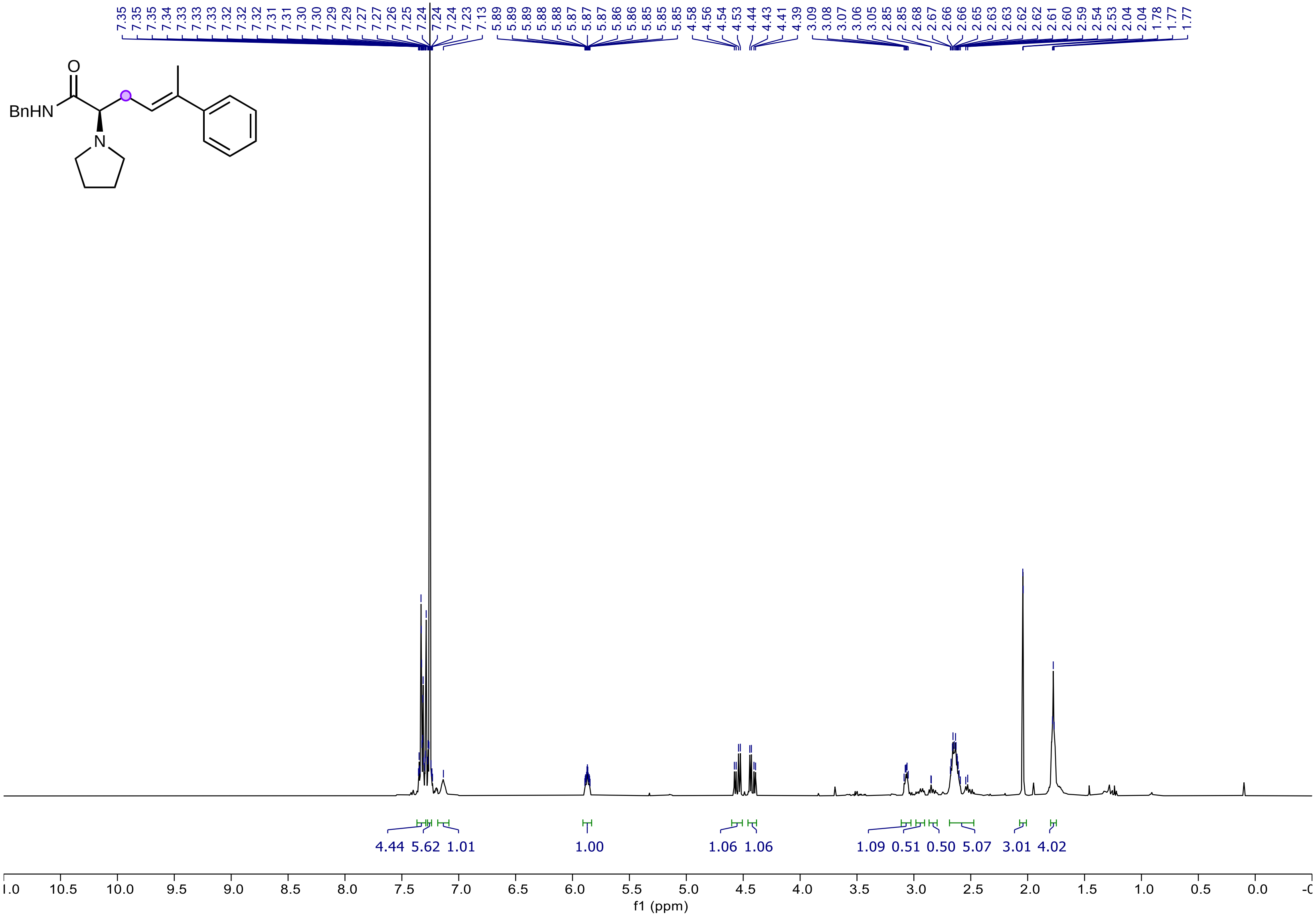

3-[$^{13}C_1$]-**3a** – ^{13}C NMR (126 MHz, $CDCl_3$)

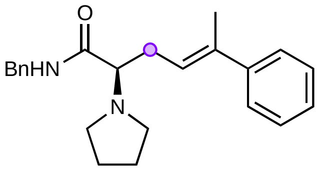

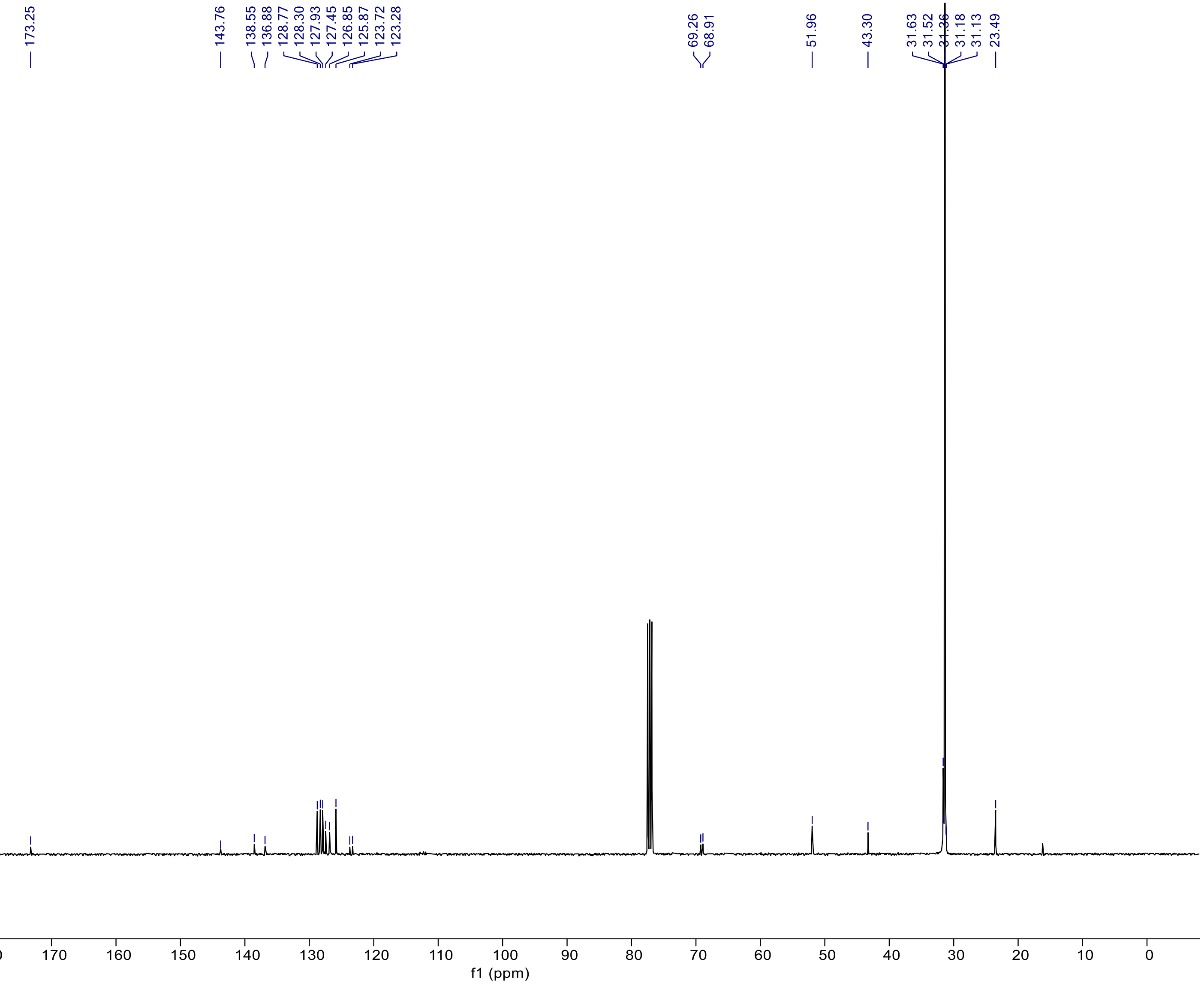

2,3-[$^{13}C_2$]-**3a** – ^{1}H NMR (500 MHz, $CDCl_3$)

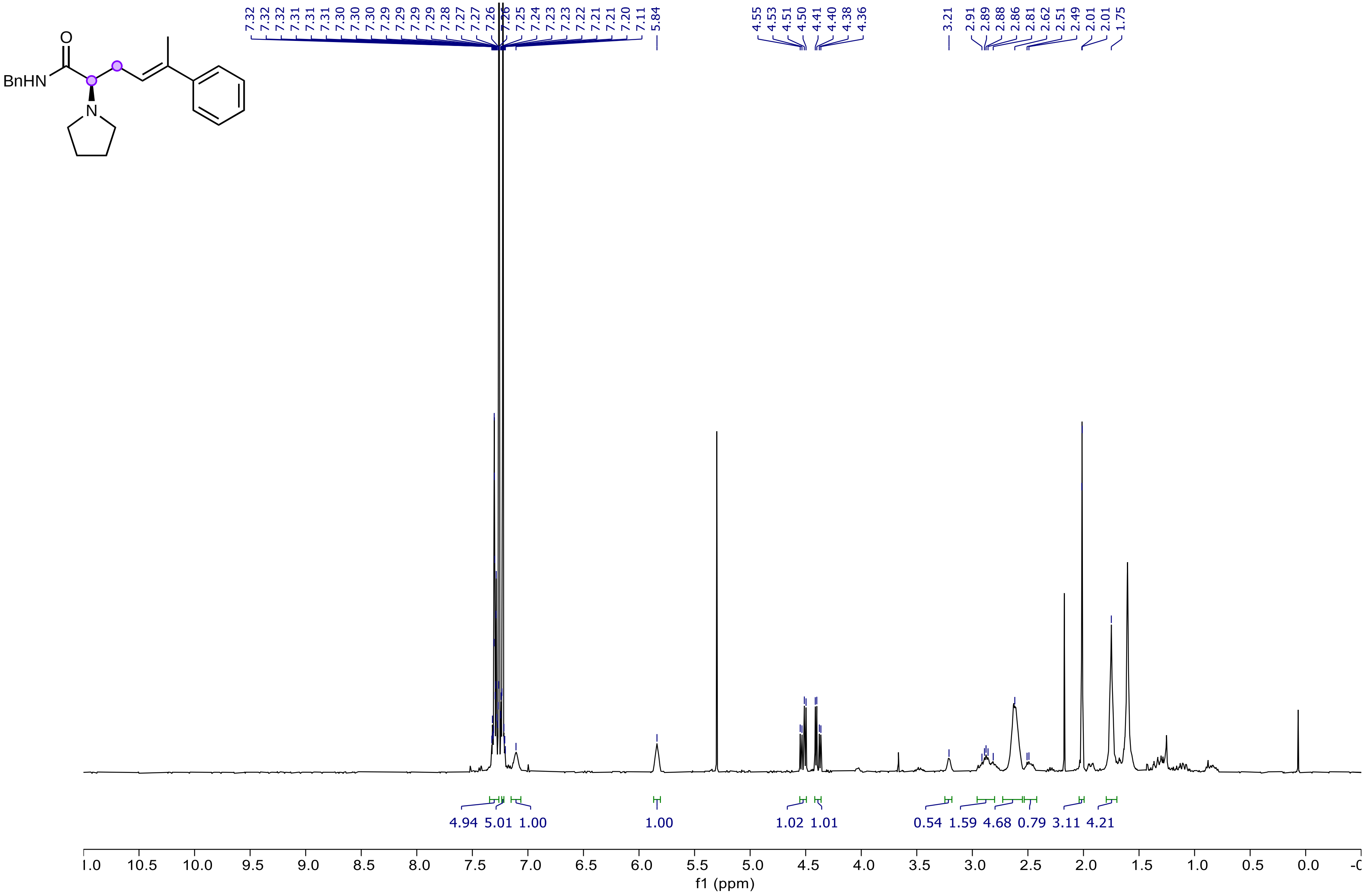

2,3-[$^{13}C_2$]-**3a** – ^{13}C NMR (126 MHz, $CDCl_3$)

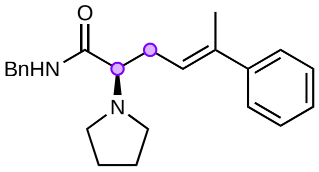

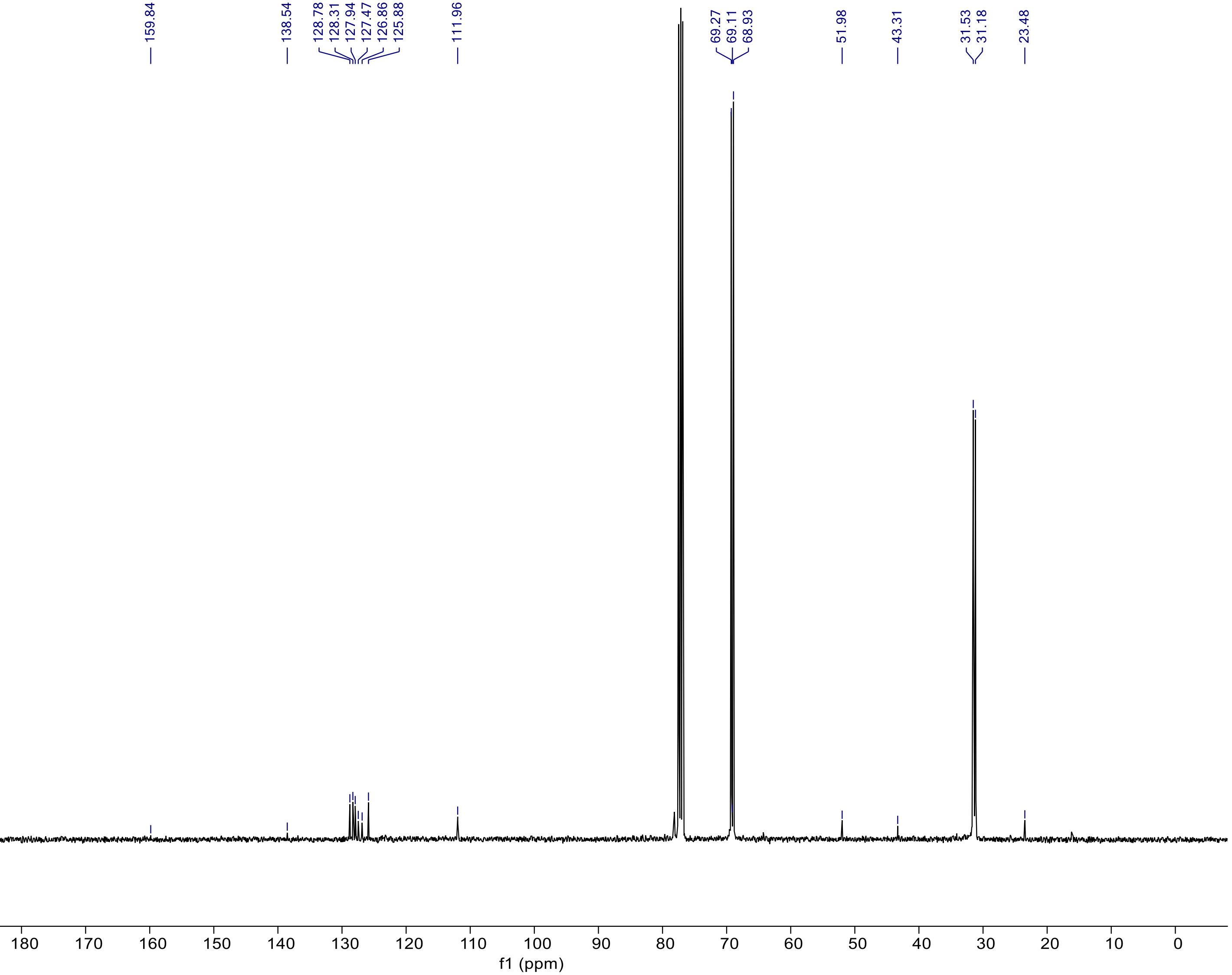

S68 – ^{1}H NMR (400 MHz, $CDCl_3$)

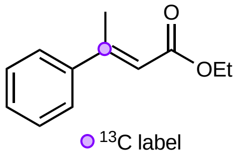

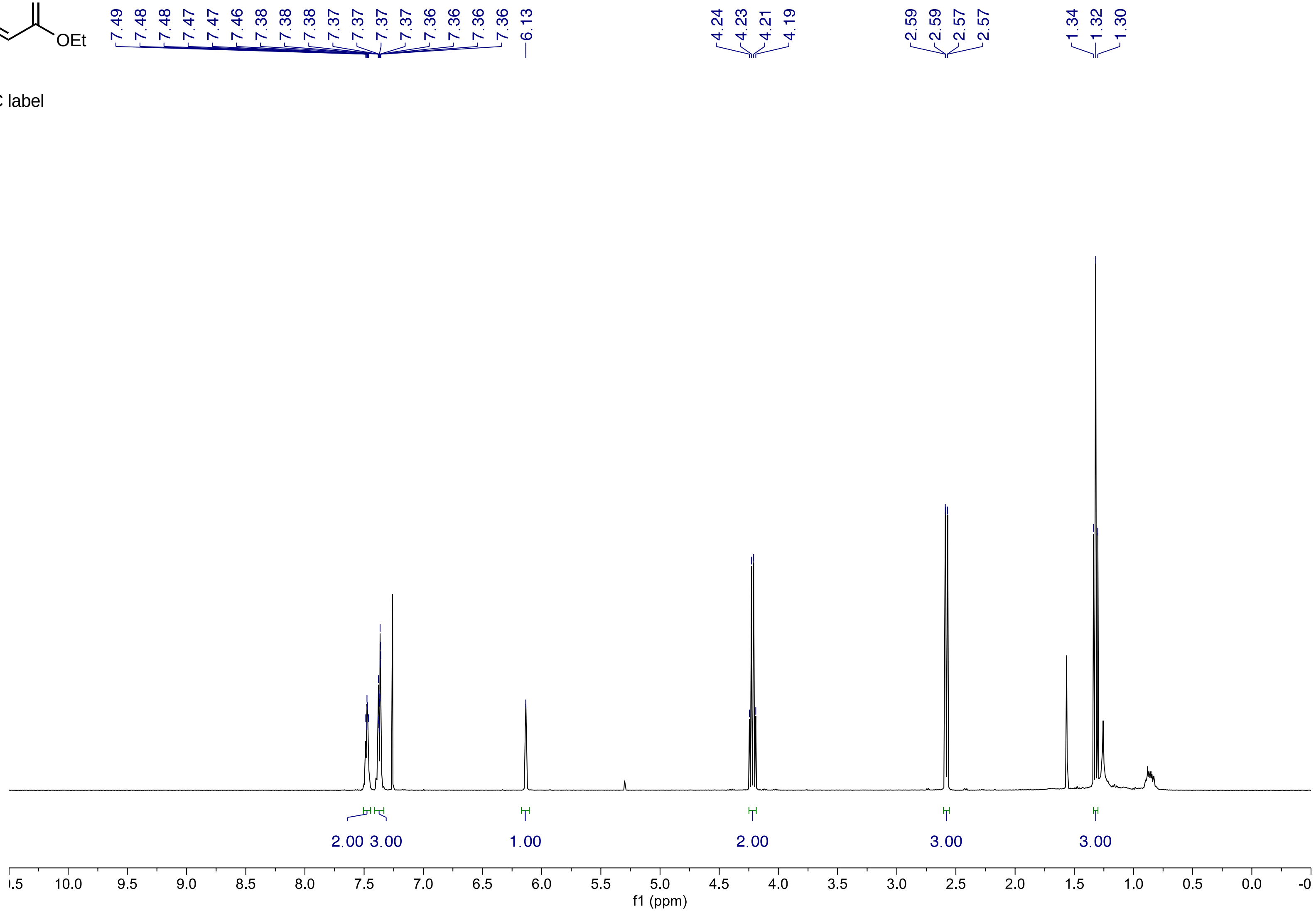

S68 – ^{13}C NMR (126 MHz, $CDCl_3$)

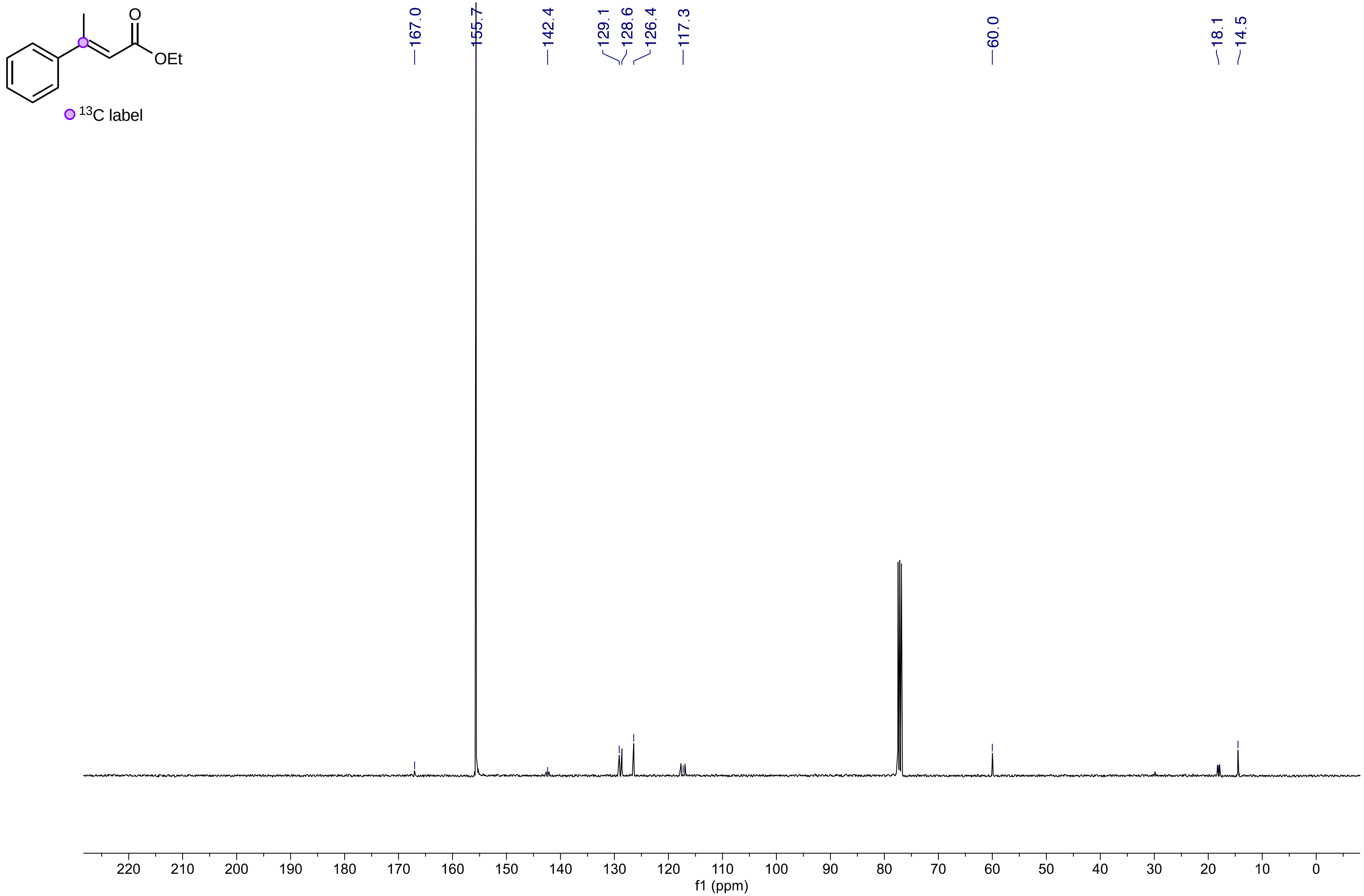

S69 – ^{1}H NMR (400 MHz, $CDCl_3$)

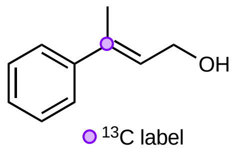

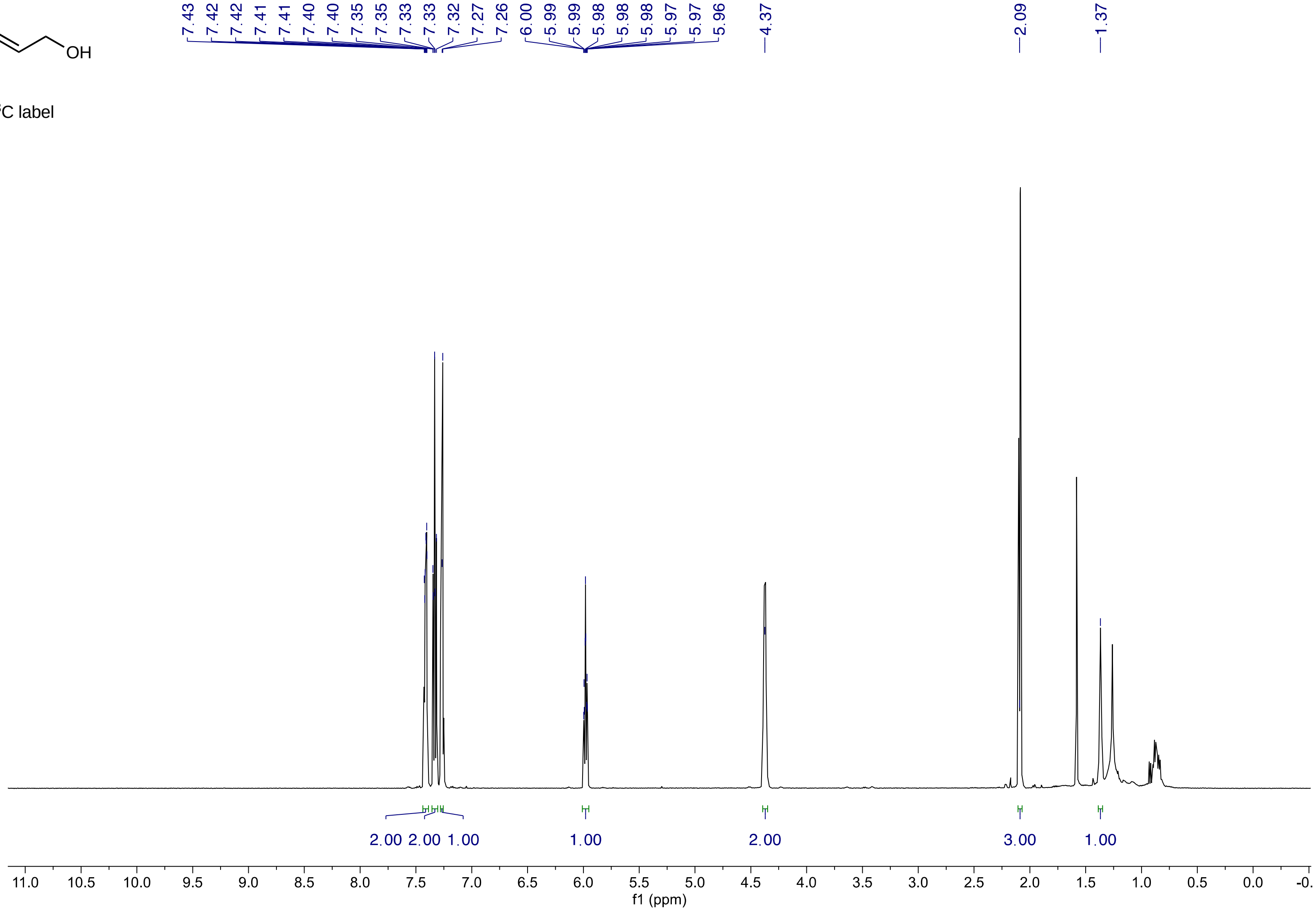

S69 – ^{13}C NMR (126 MHz, $CDCl_3$)

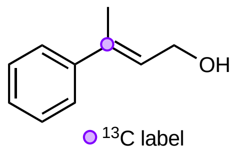

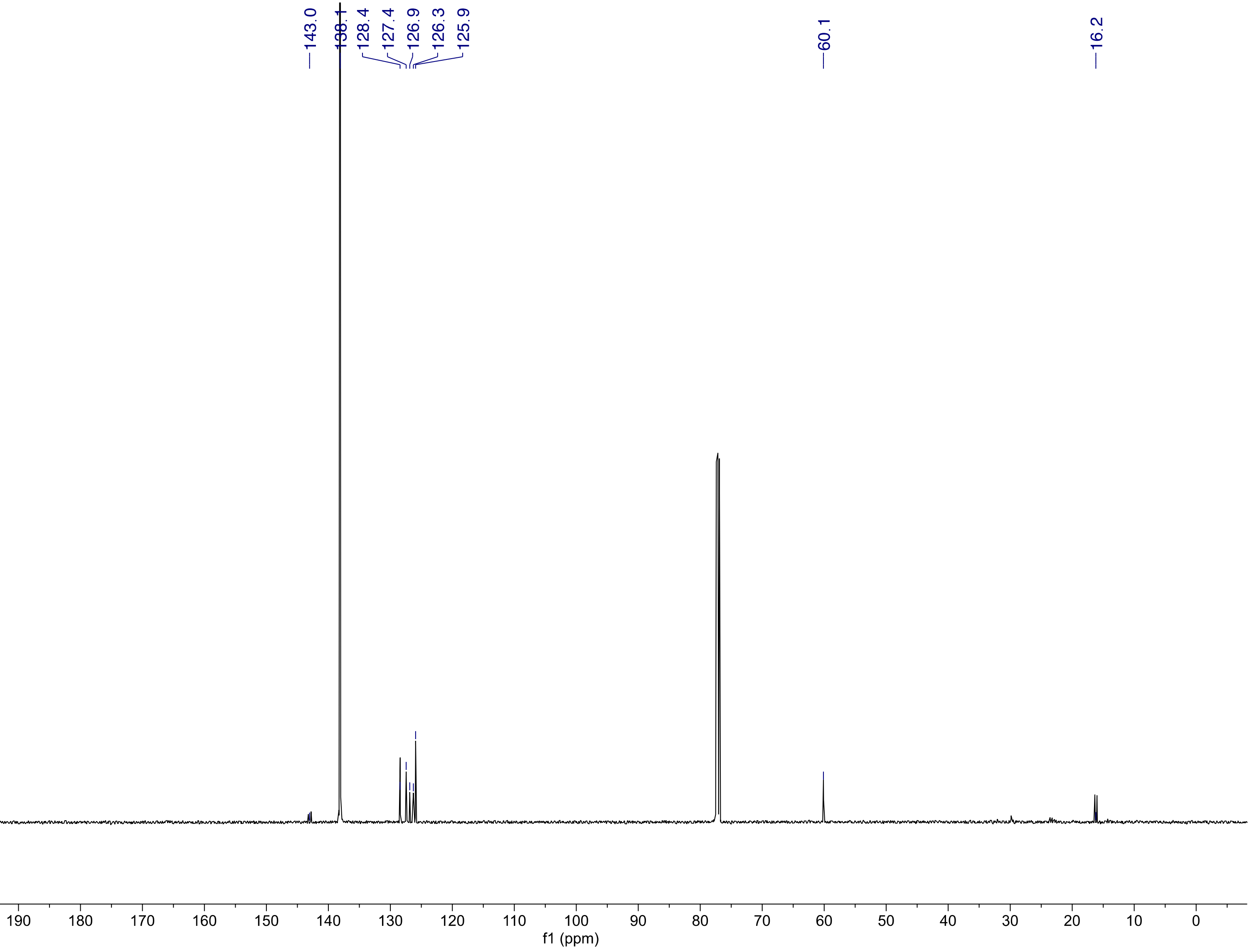

S70 – ^{1}H NMR (400 MHz, $CDCl_3$)

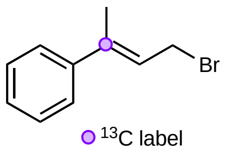

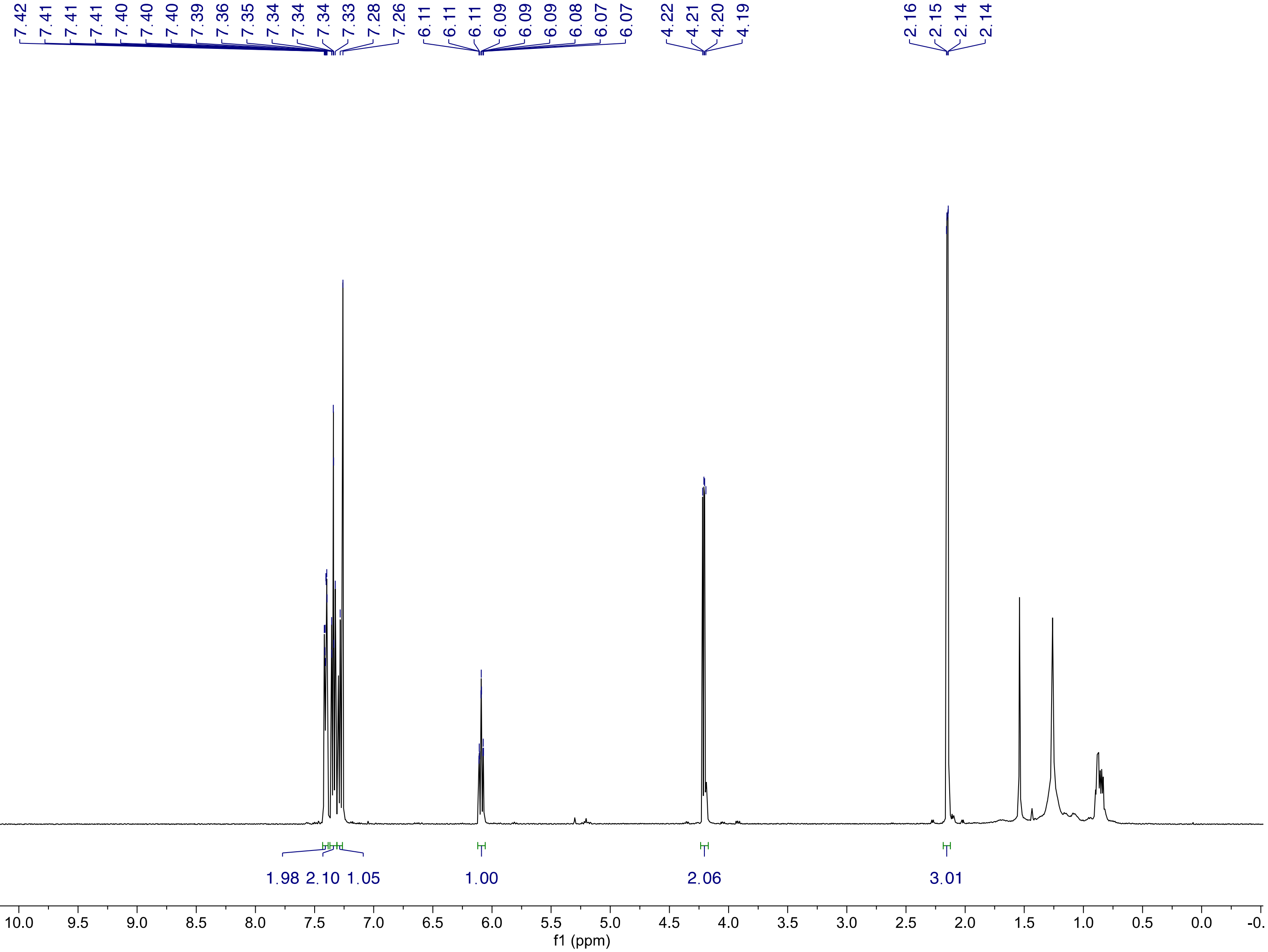

S70 – ^{13}C NMR (126 MHz, $CDCl_3$)

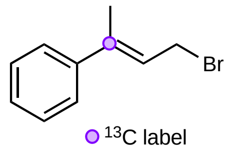

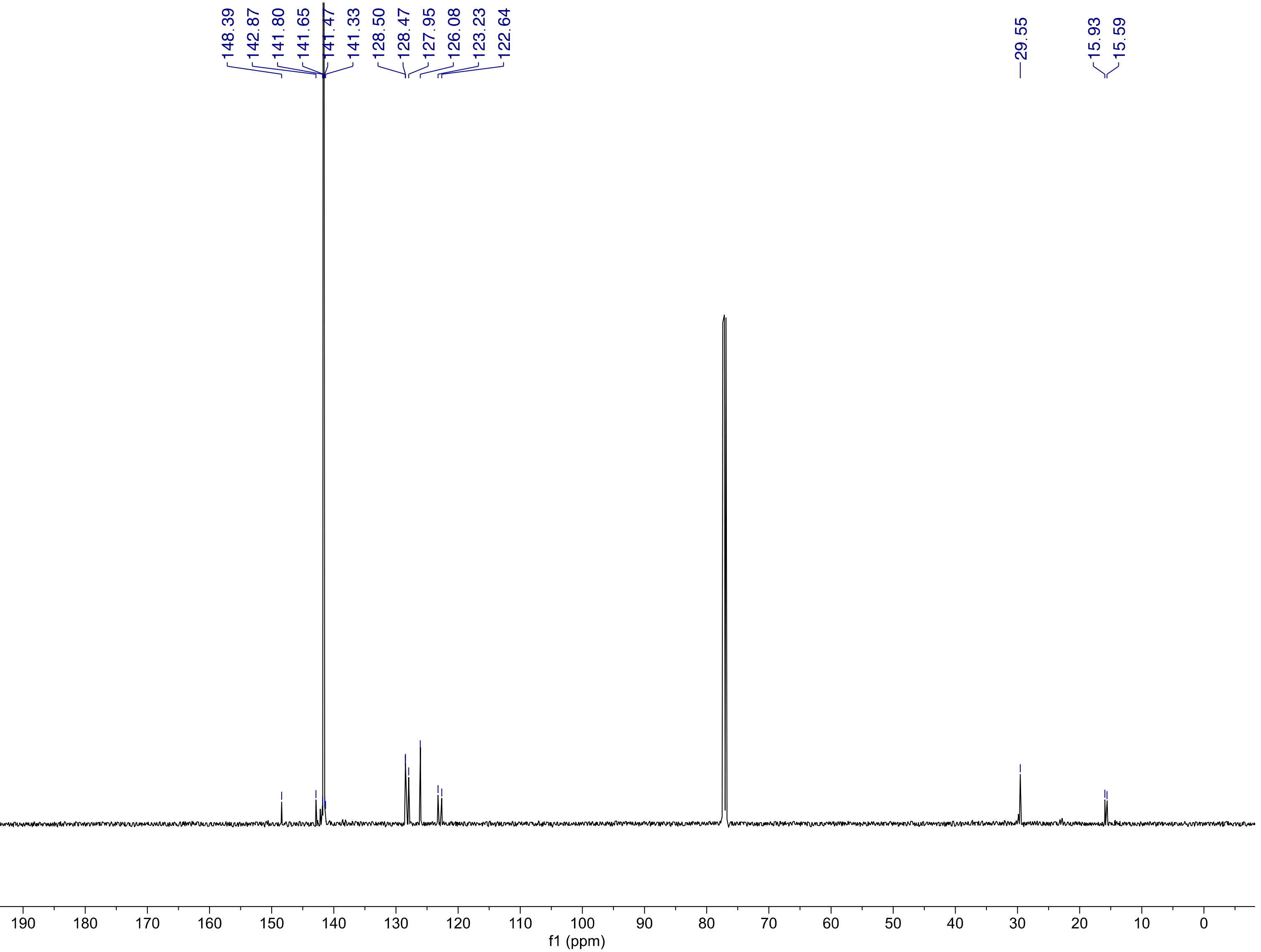

S71 – ^{1}H NMR (400 MHz, $CDCl_3$)

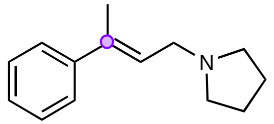

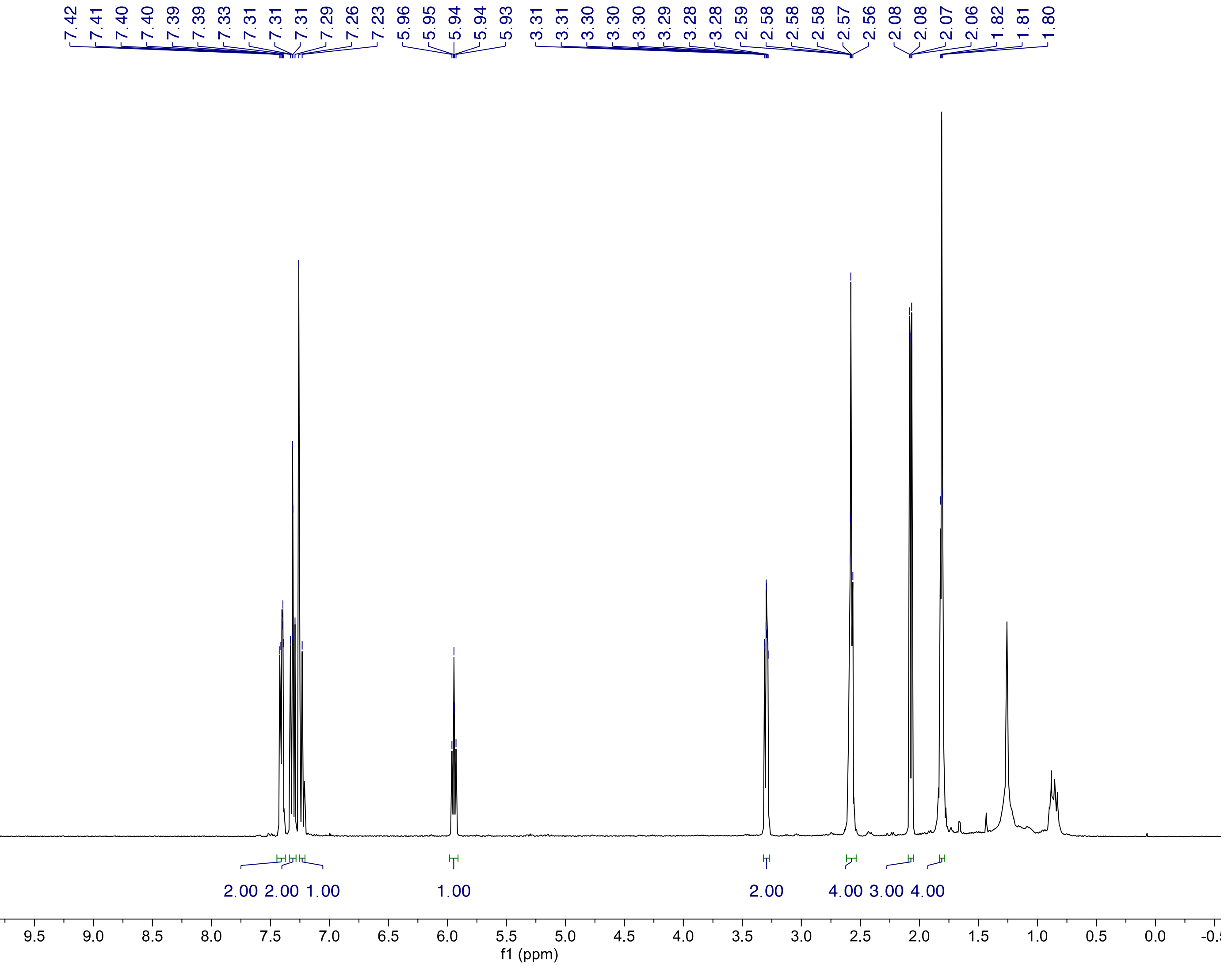

S71 – ^{13}C NMR (126 MHz, $CDCl_3$)

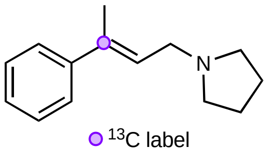

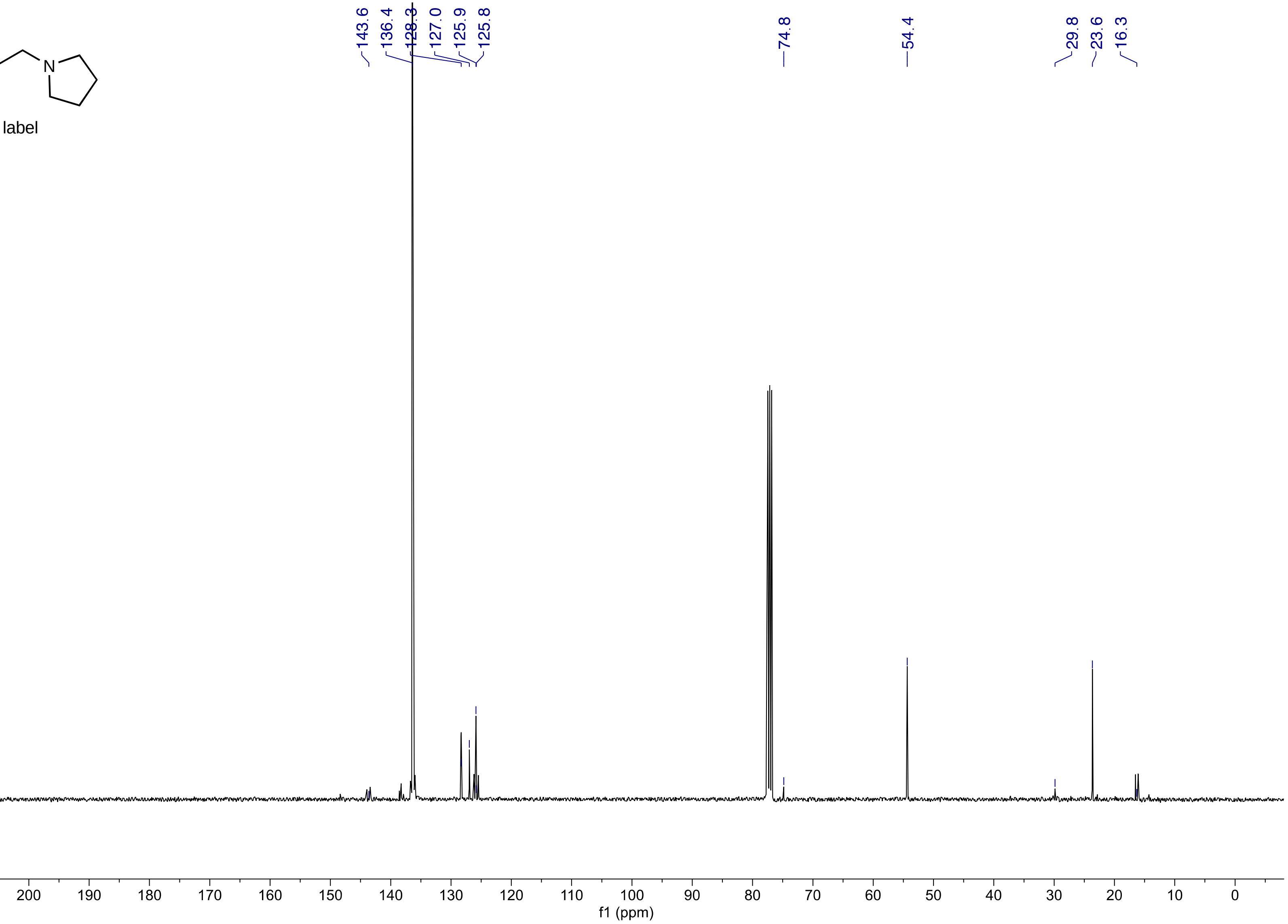

4’-[$^{13}C_1$]-**2a** - ^{1}H NMR (400 MHz, d6-DMSO)

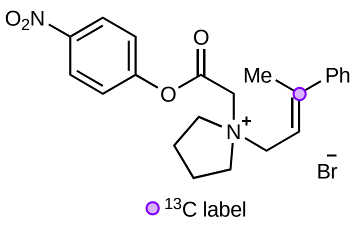

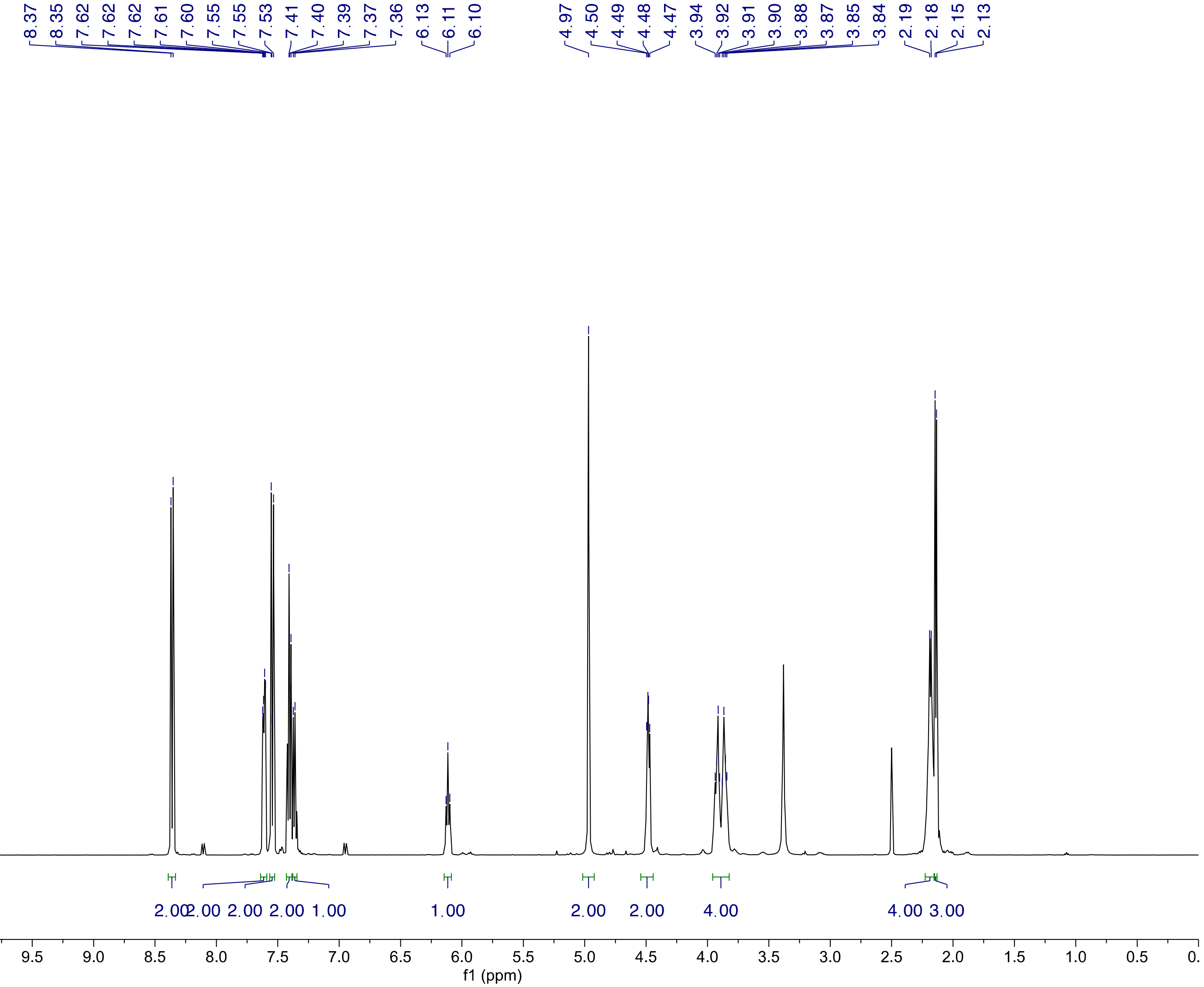

4’-[$^{13}C_1$]-**2a** – ^{13}C NMR (126 MHz, d6-DMSO)

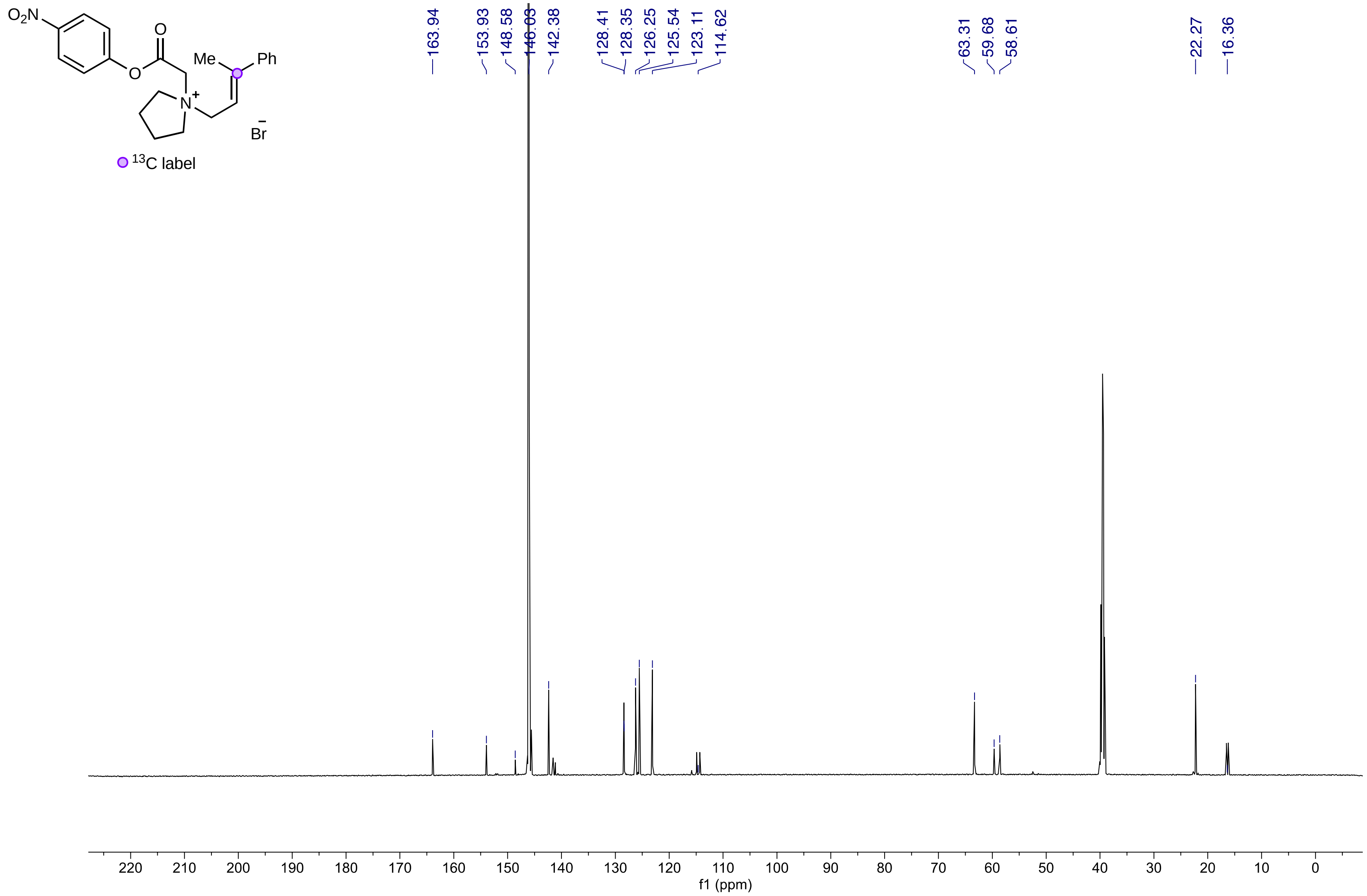

2,4'-[$^{13}C_2$]-**2a** – 1H NMR (400 MHz, d6-DMSO)

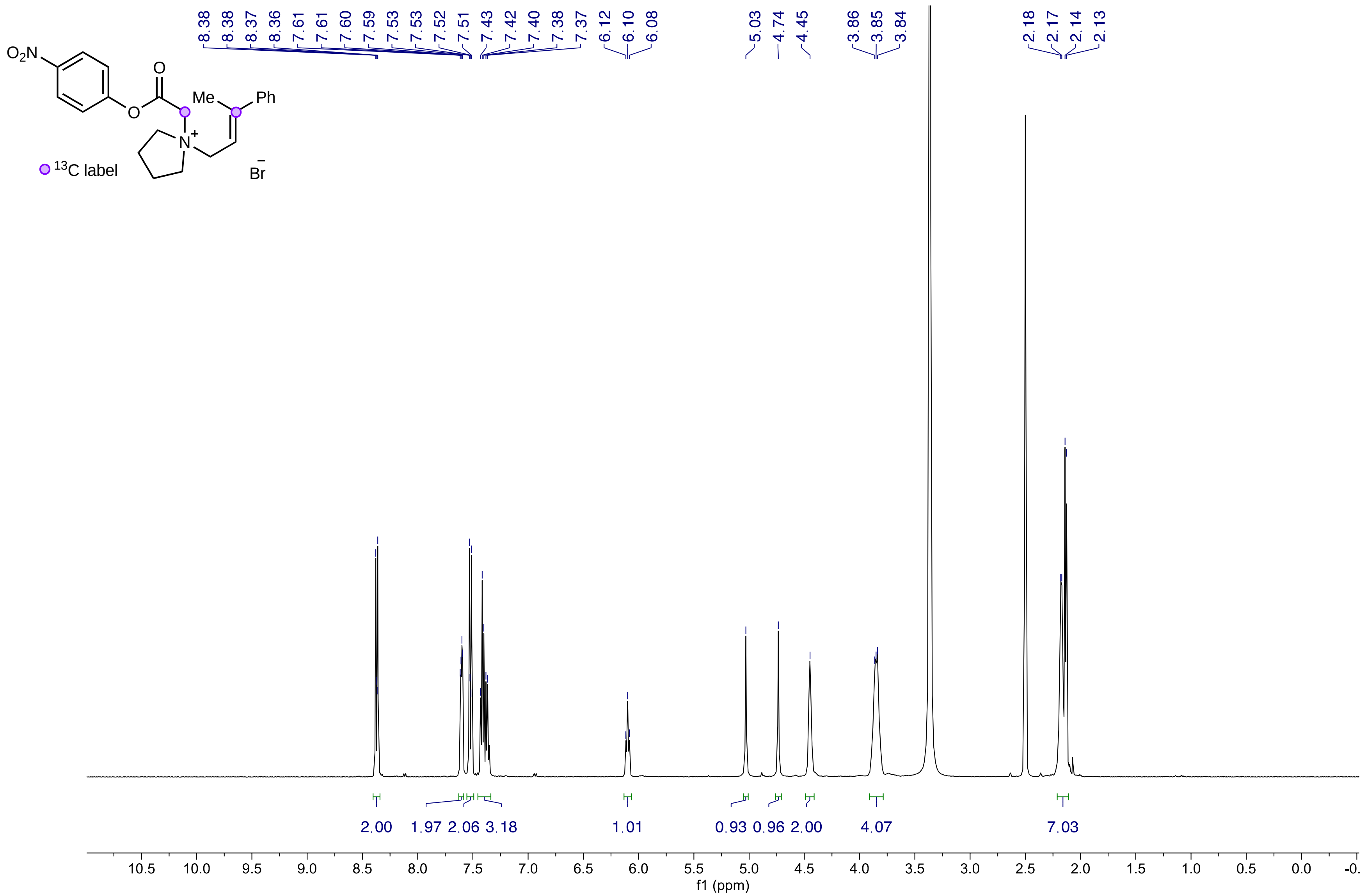

2,4’-[$^{13}C_2$]-**2a** – ^{13}C NMR (126 MHz, d6-DMSO)

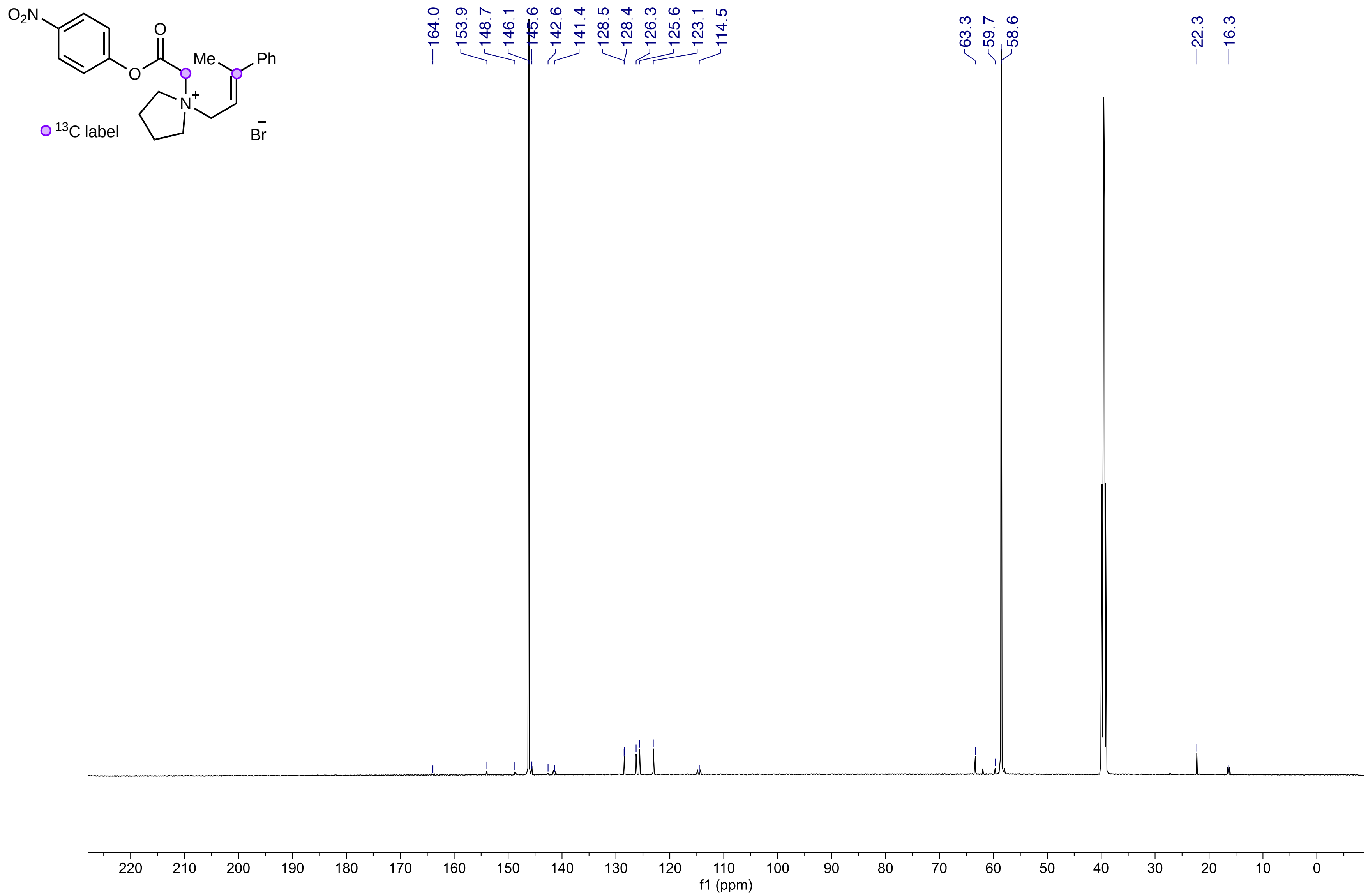

S72 – ^{1}H NMR (500 MHz, $CDCl_3$)

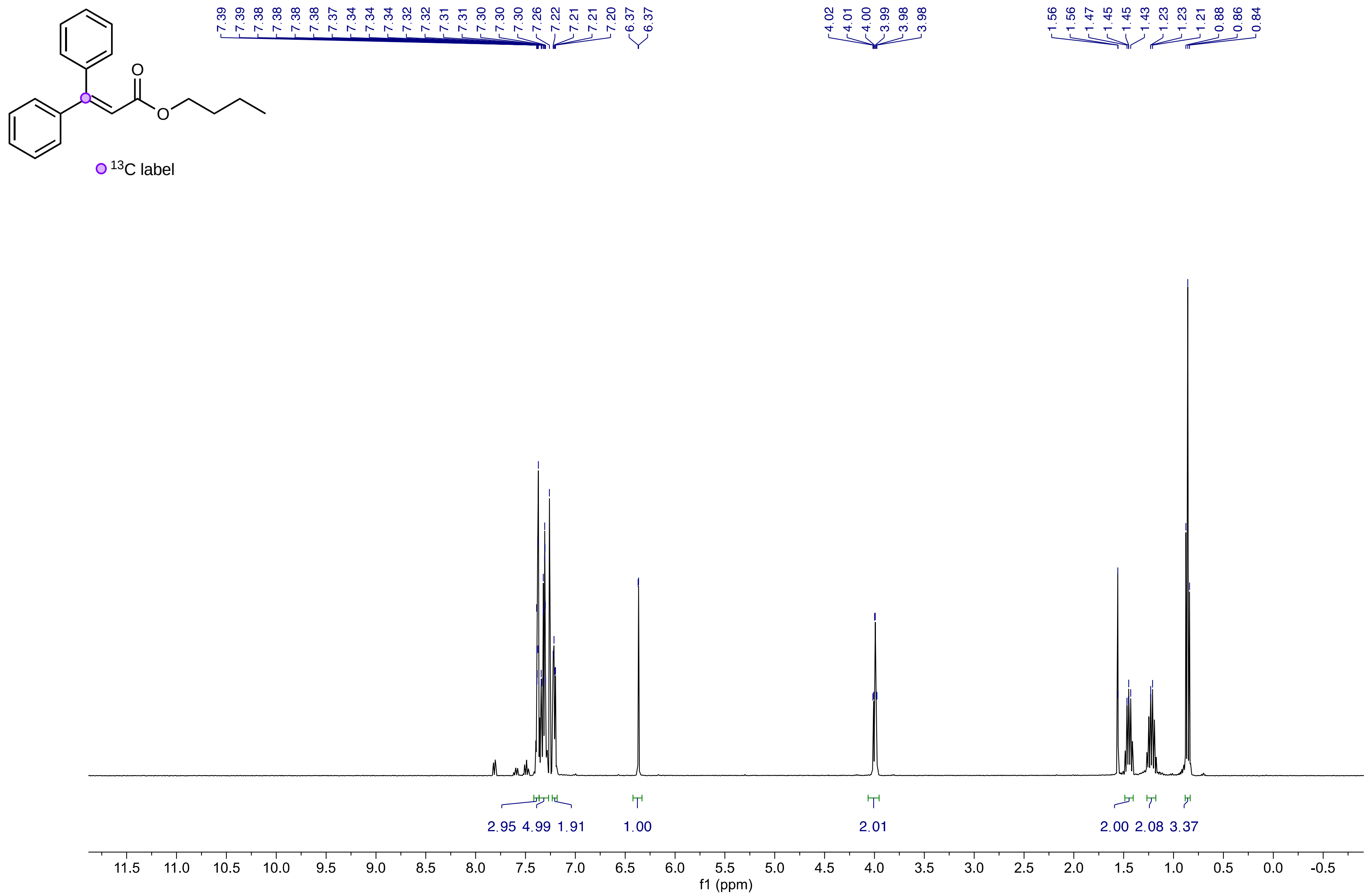

S73 – 1H NMR (400 MHz, $CDCl_3$)

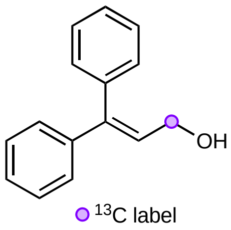

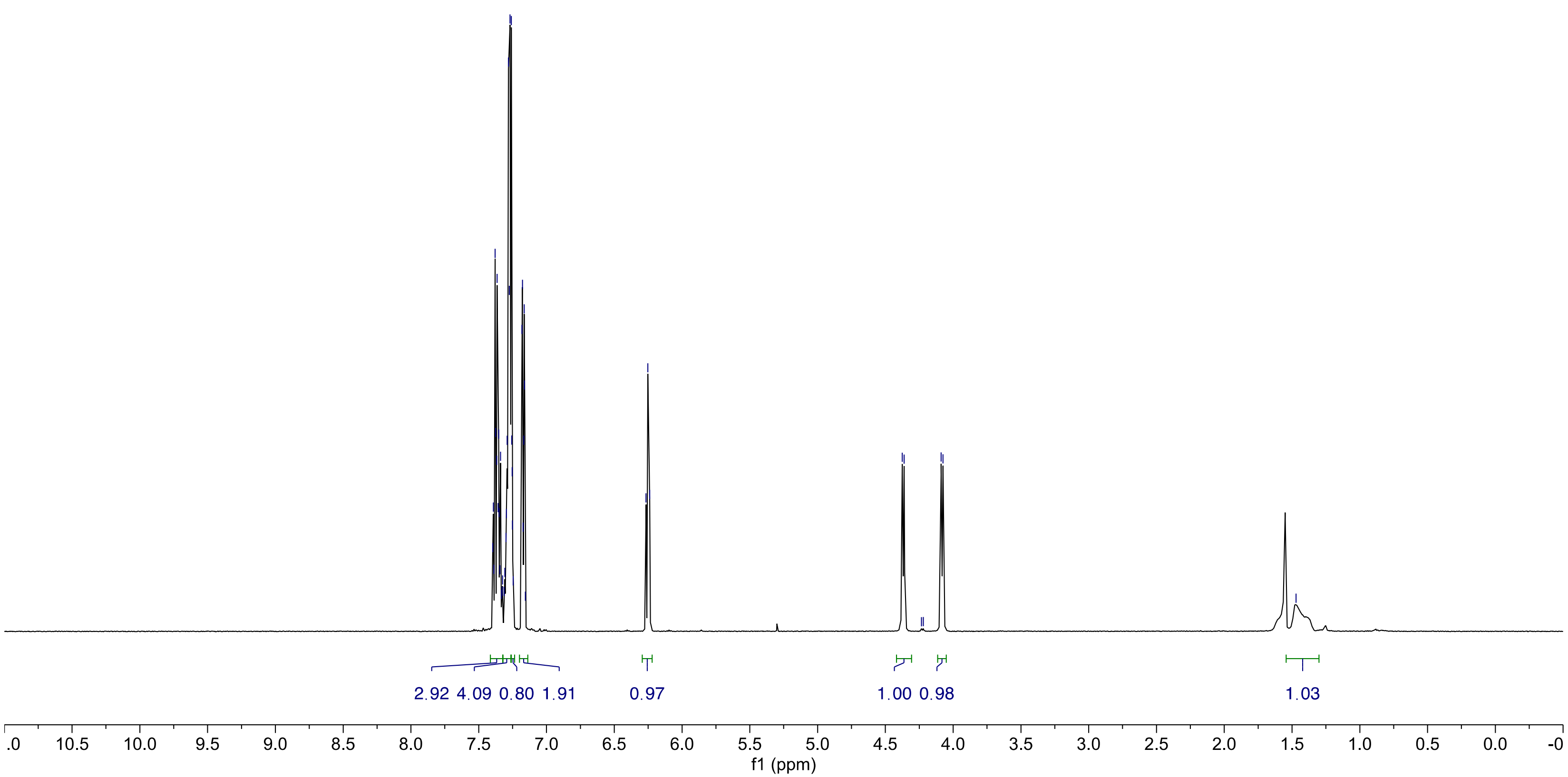

S74 – ^{1}H NMR (400 MHz, $CDCl_3$)

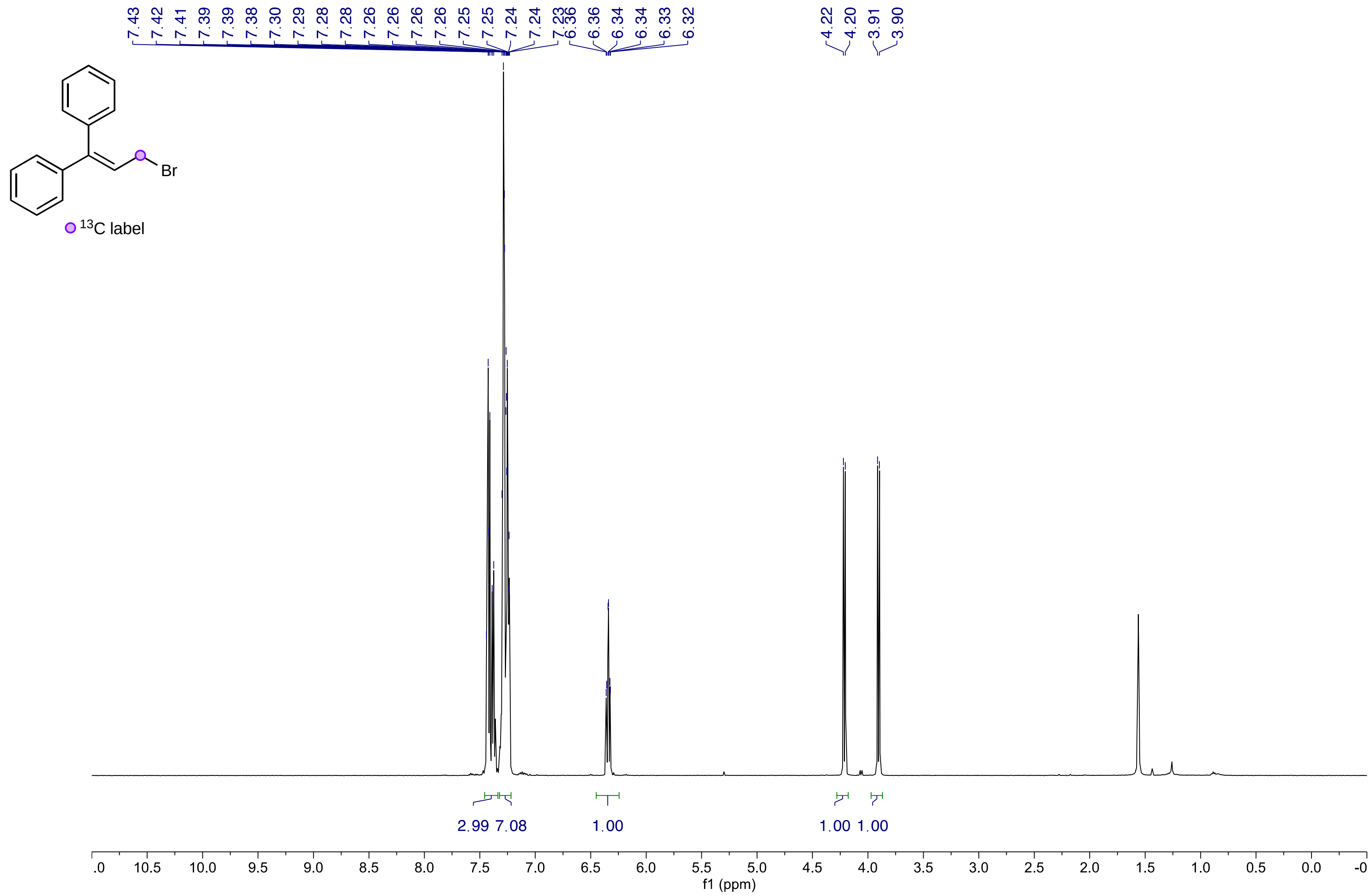

S74 – ^{13}C NMR (126 MHz, $CDCl_3$)

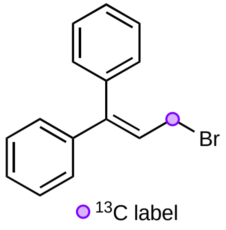

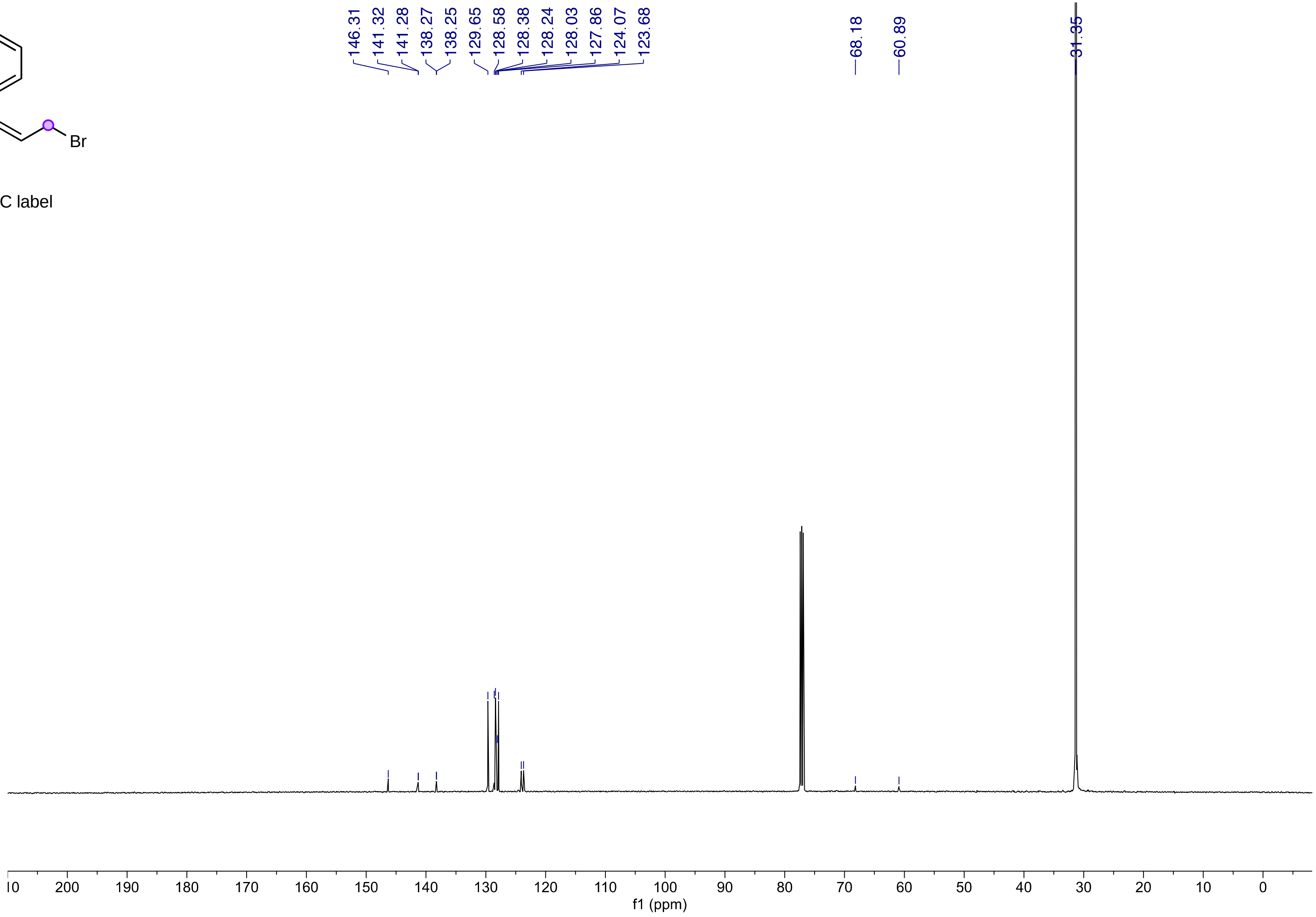

S75 – ^{1}H NMR (400 MHz, $CDCl_3$)

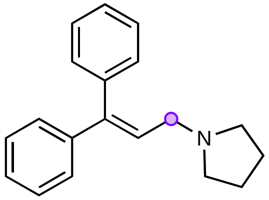

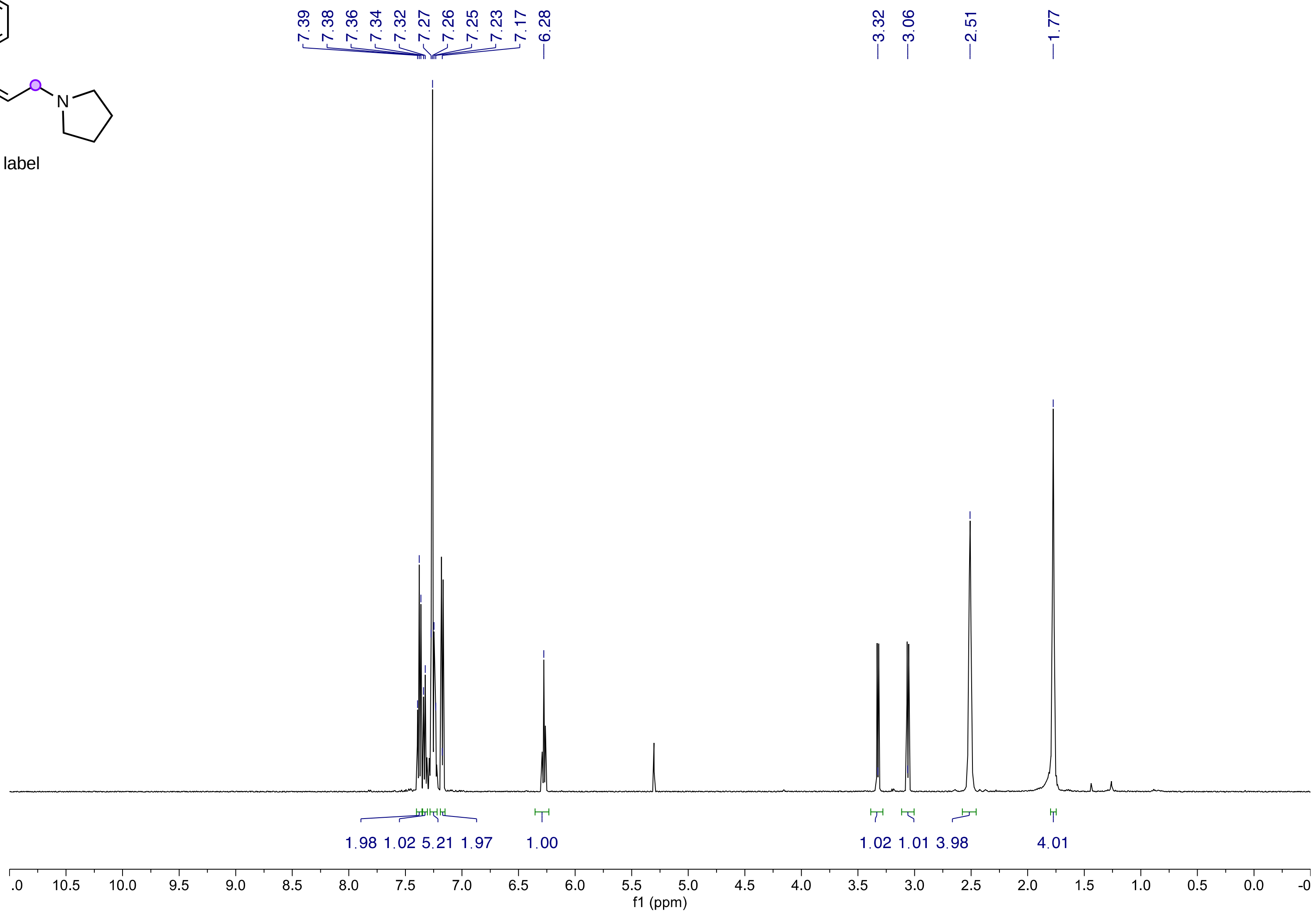

S75 – ^{13}C NMR (126 MHz, $CDCl_3$)

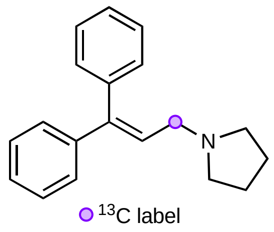

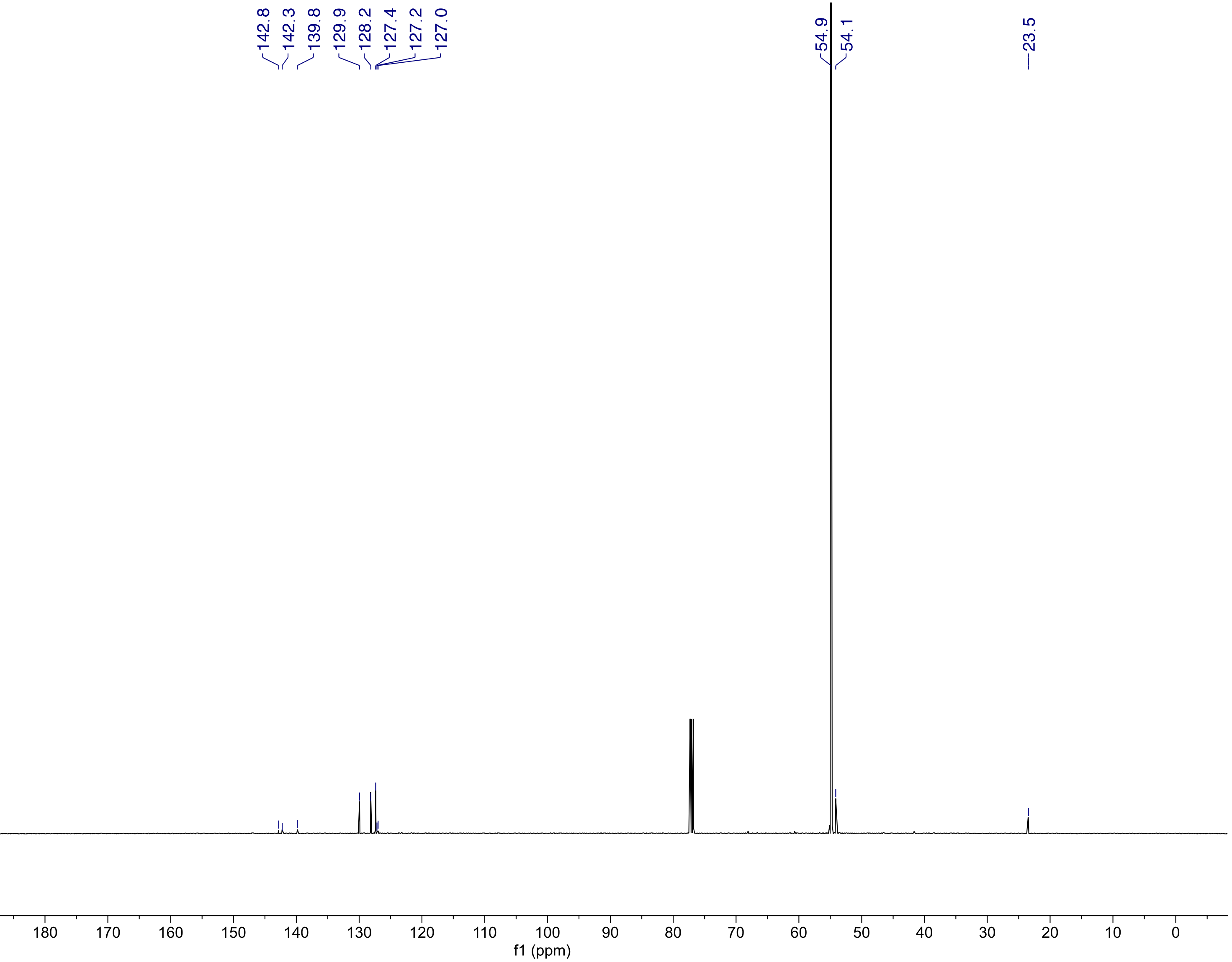

2-[$^{13}C_1$]-**2e** – ^{1}H NMR (400 MHz, d6-DMSO)

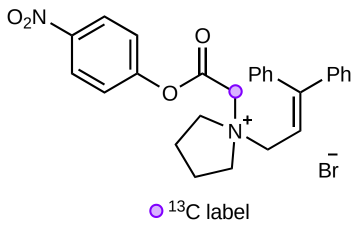

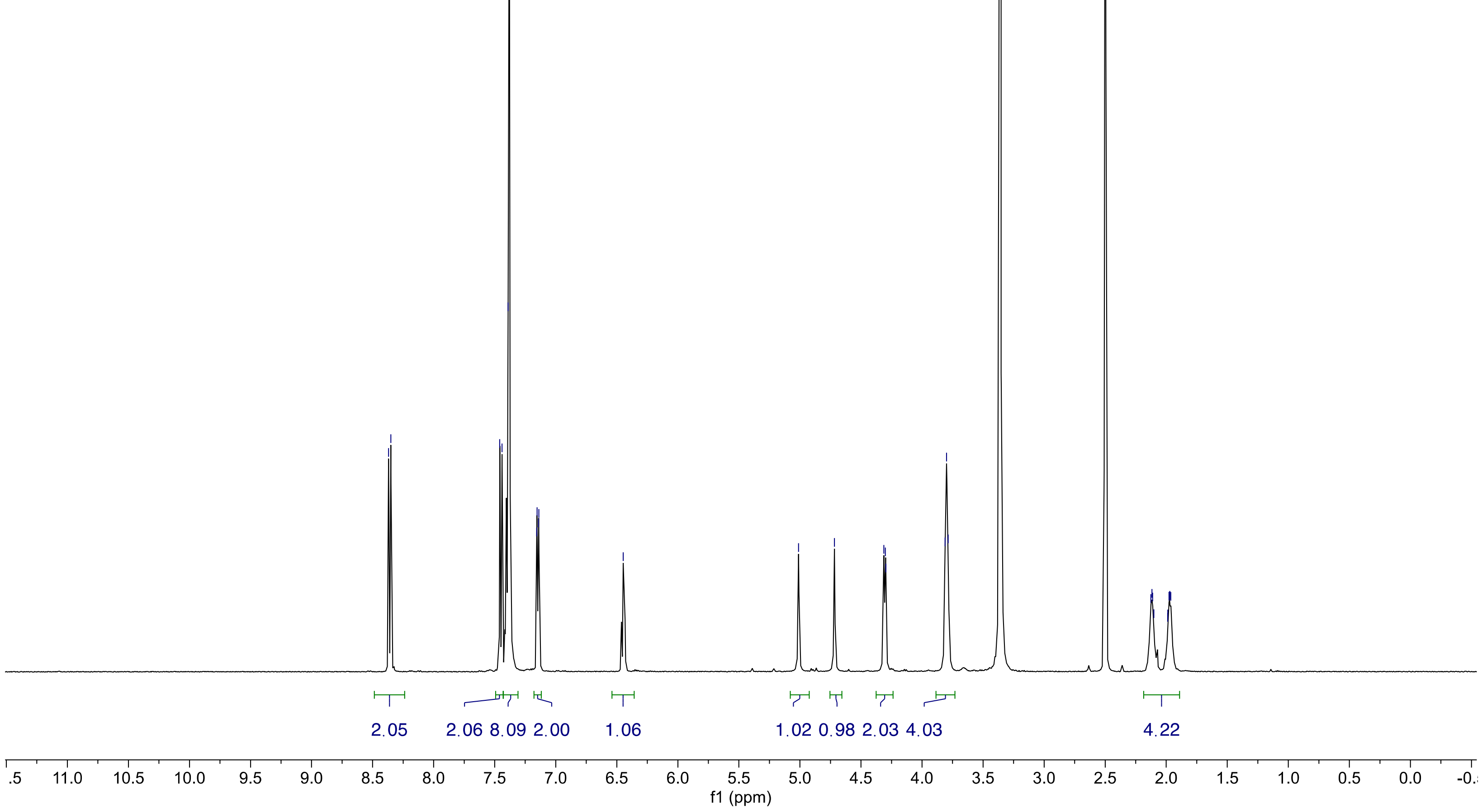

2-[$^{13}C_1$]-**2e** – ^{13}C NMR (126 MHz, d6-DMSO)

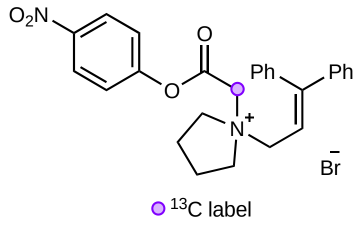

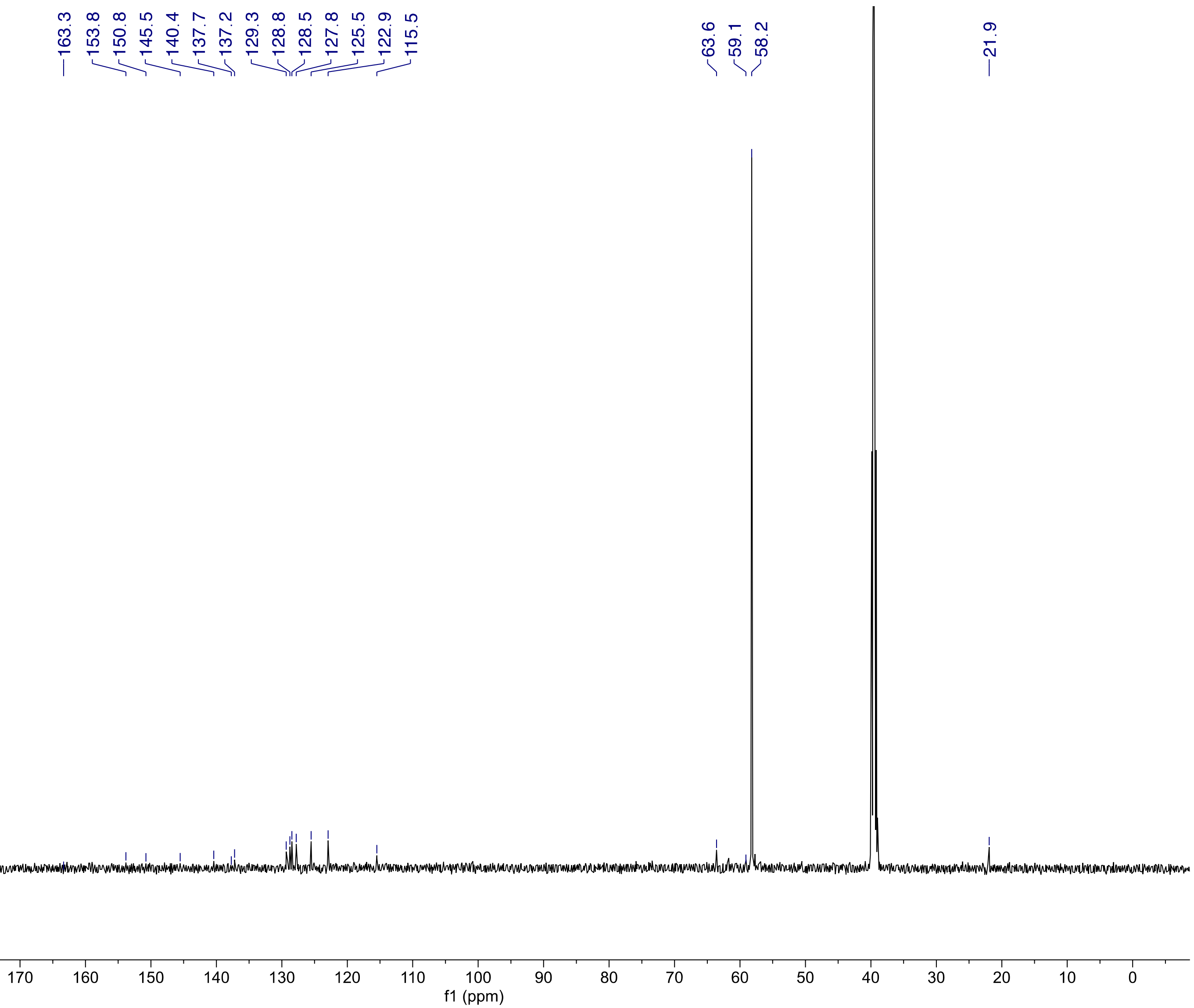

2’-[$^{13}C_1$]-**2e** – ^{1}H NMR (400 MHz, d6-DMSO)

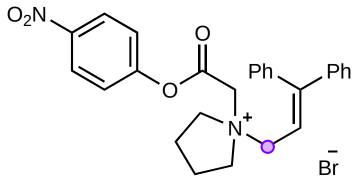

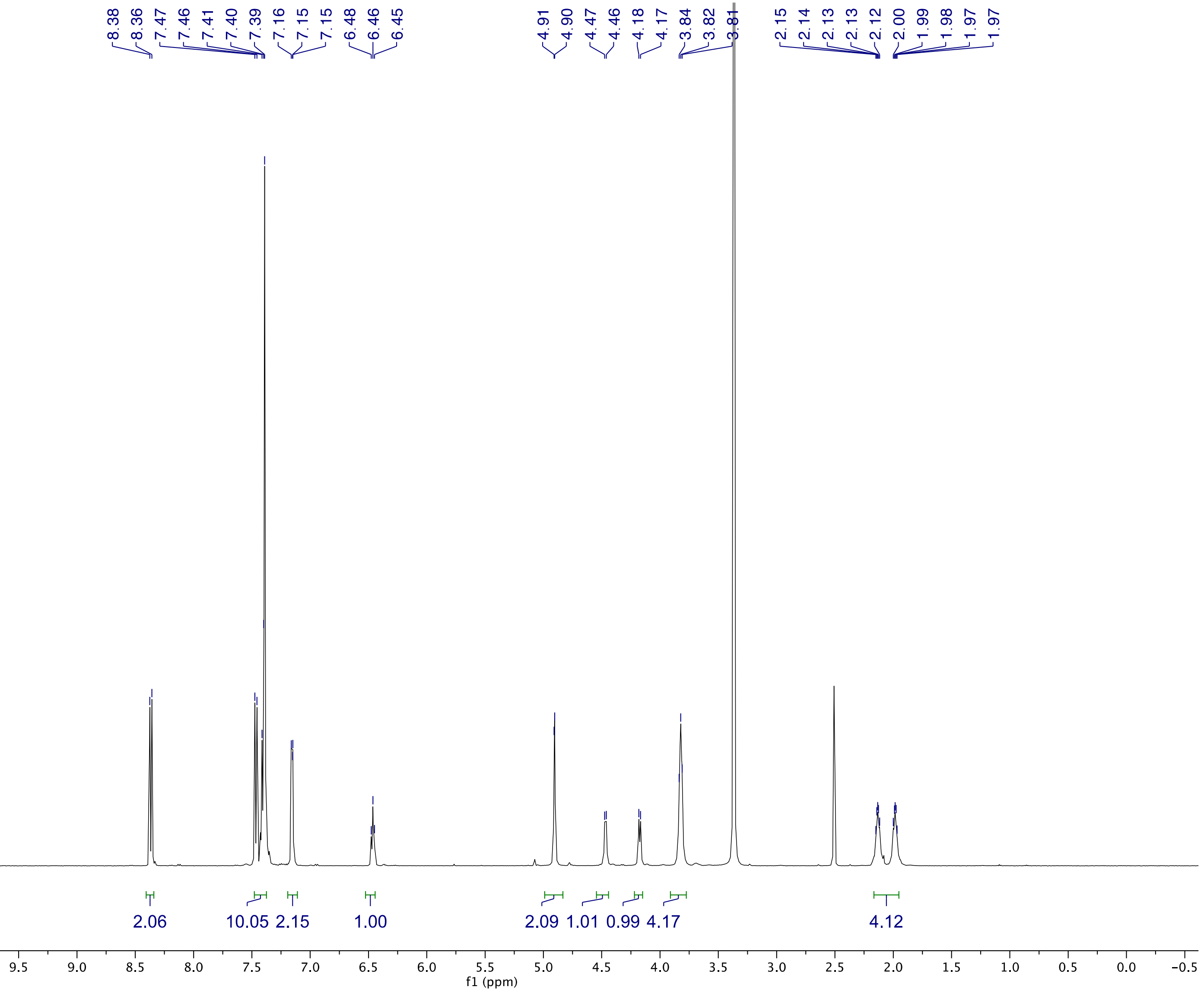

2’-[$^{13}C_1$]-**2e** – ^{13}C NMR (126 MHz, d6-DMSO)

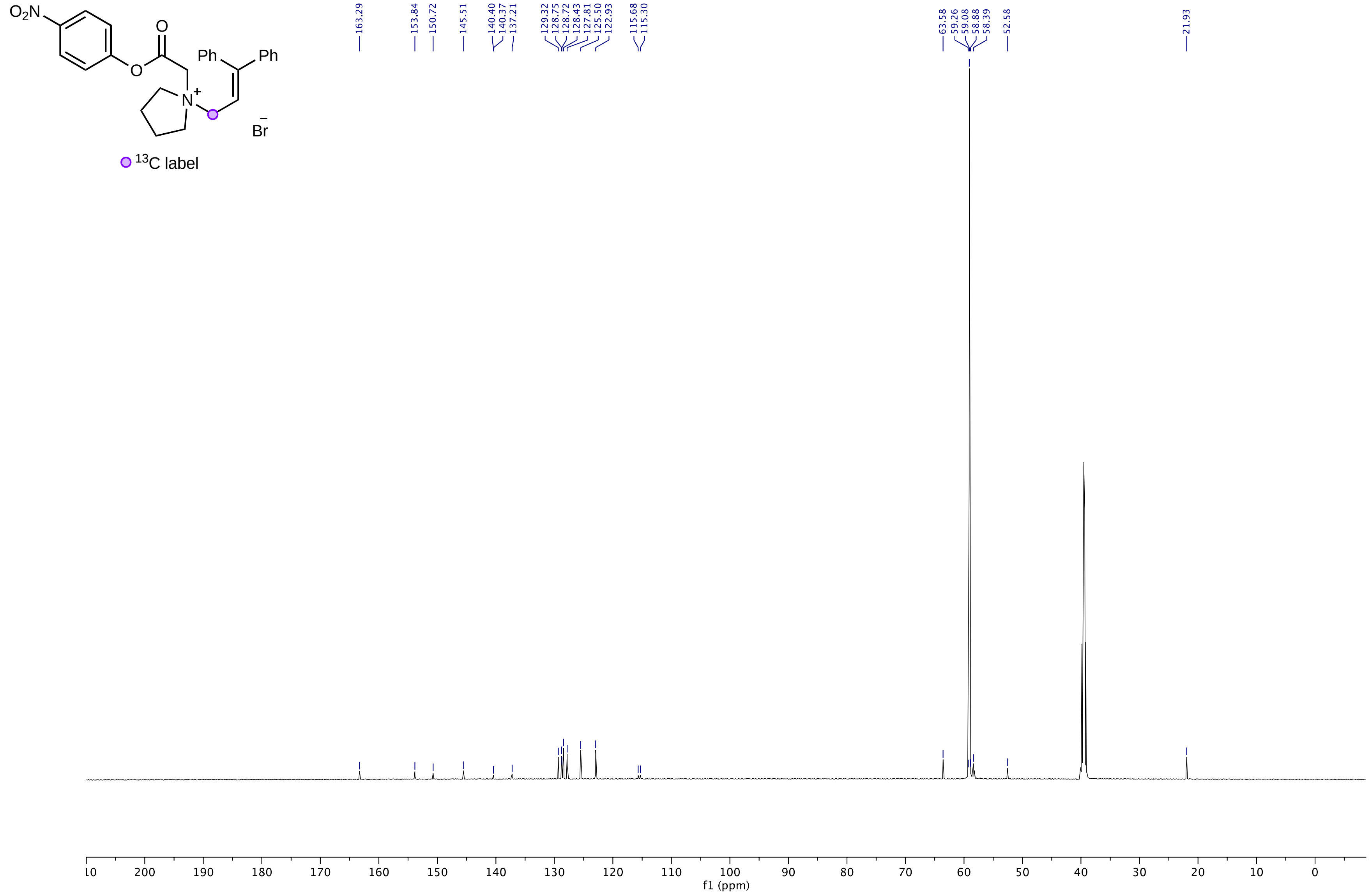

2,2'-[$^{13}C_2$]-**2e** – ^{1}H NMR (500 MHz, d6-DMSO)

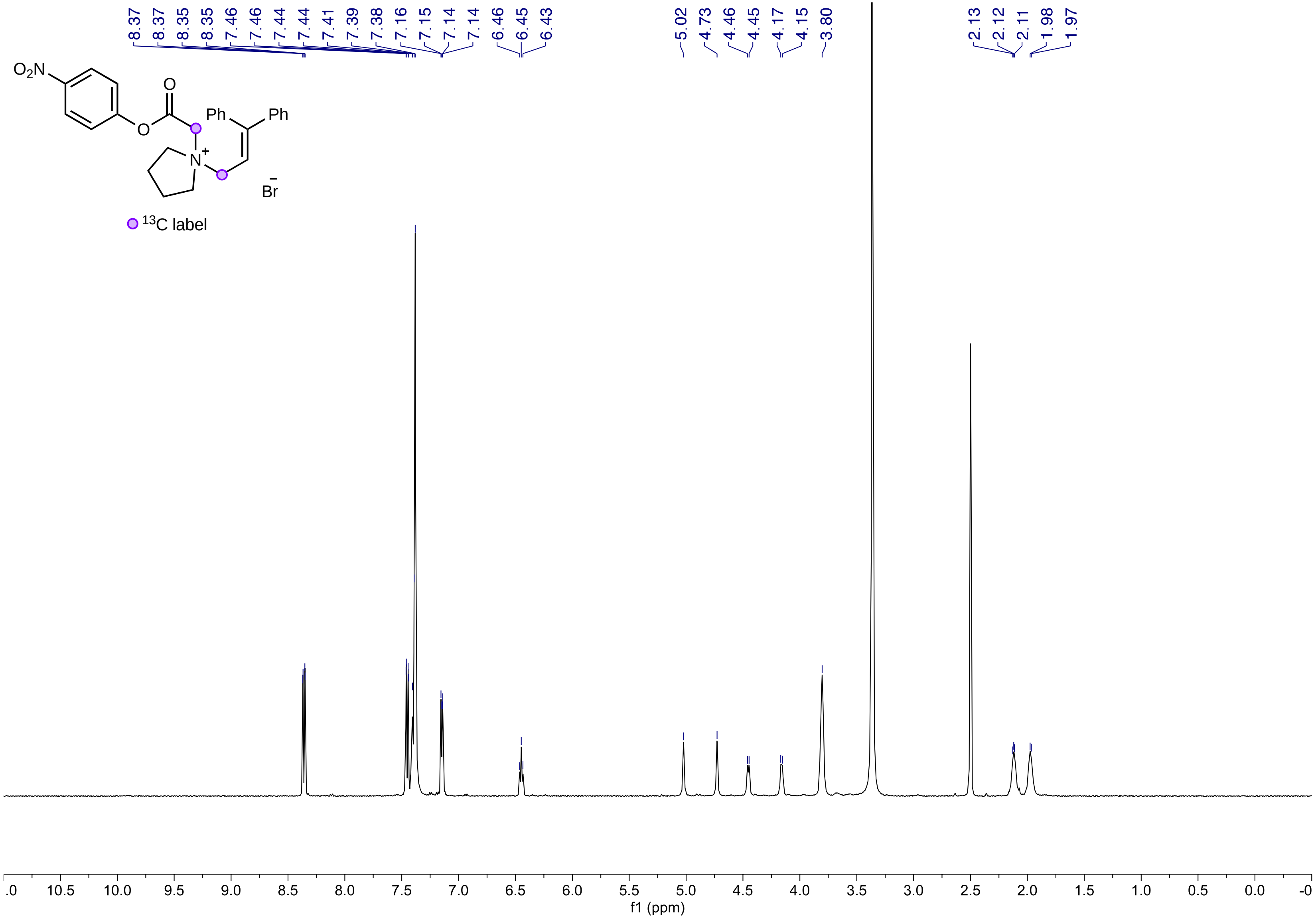

2,2'-[$^{13}C_2$]-**2e** – ^{13}C NMR (126 MHz, d6-DMSO)

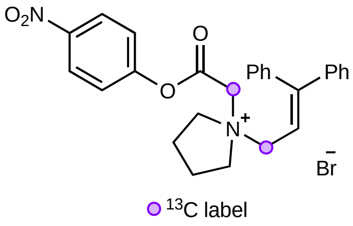

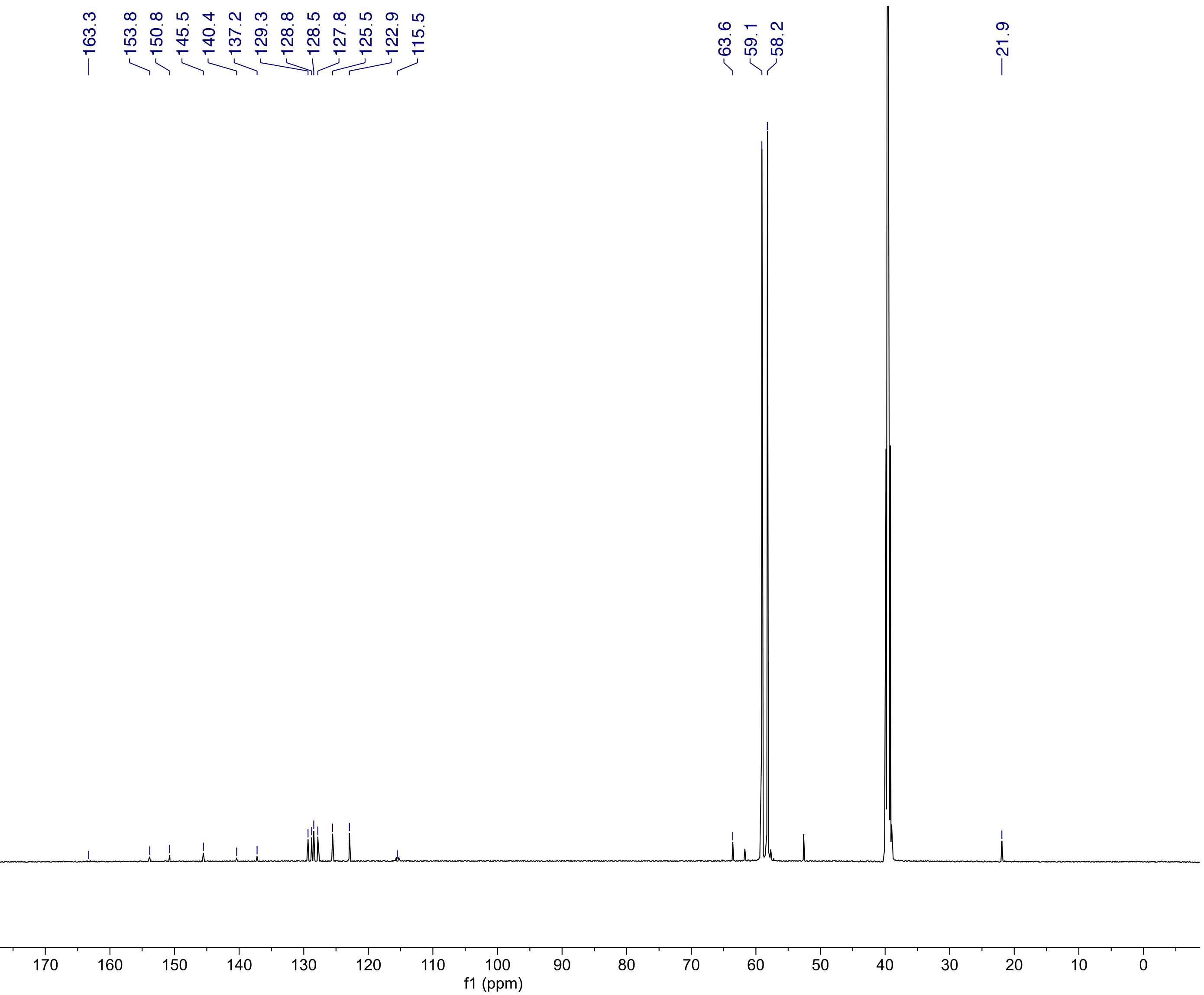

2,3-[$^{13}C_2$]-**3e** – ^{1}H NMR (500 MHz, $CDCl_3$)

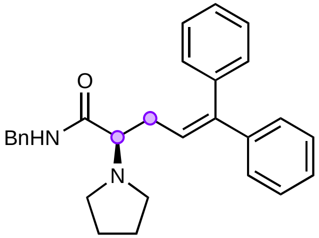

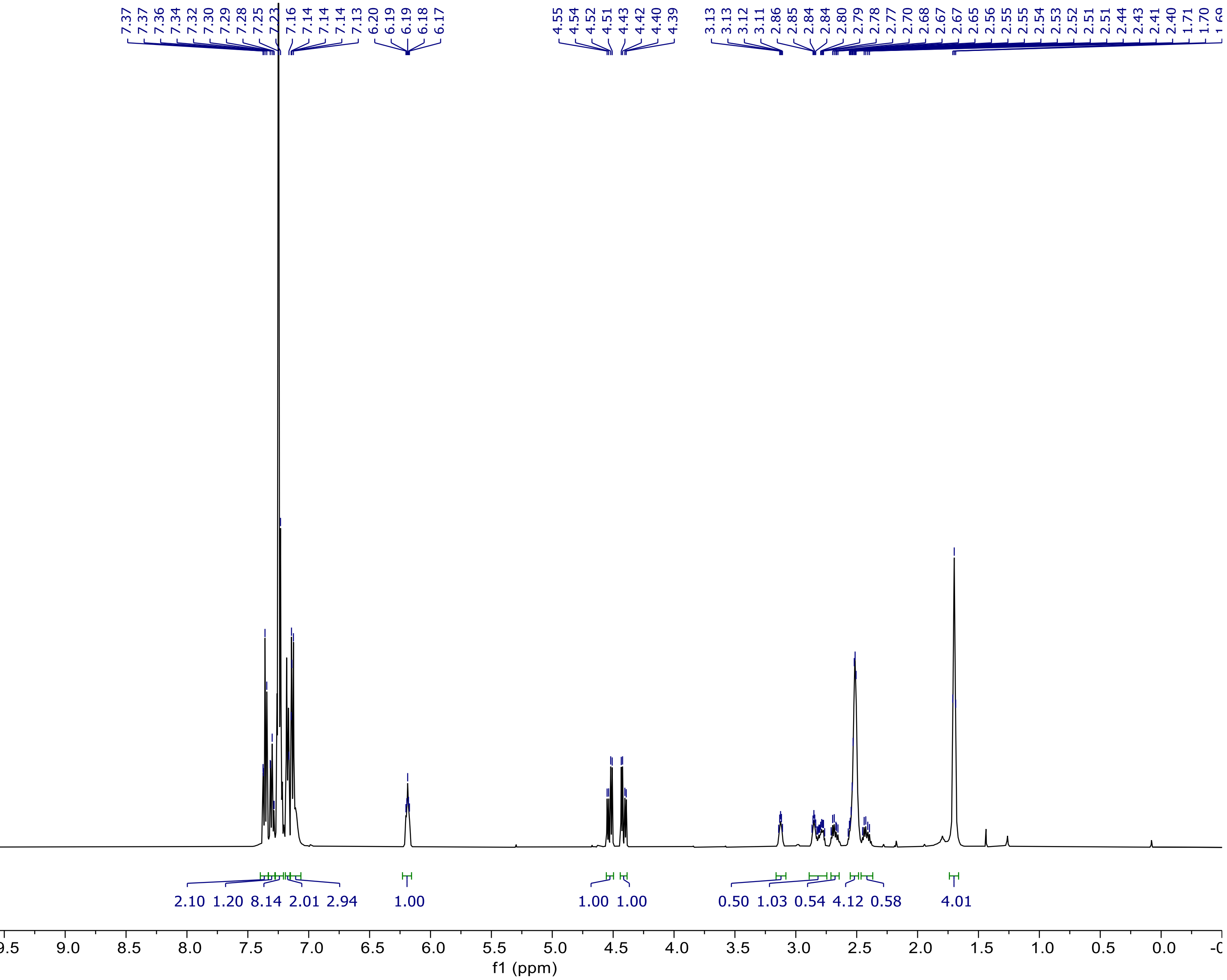

2,3-[$^{13}C_2$]-**3e** – ^{13}C NMR (126 MHz, $CDCl_3$)

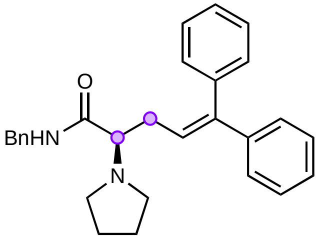

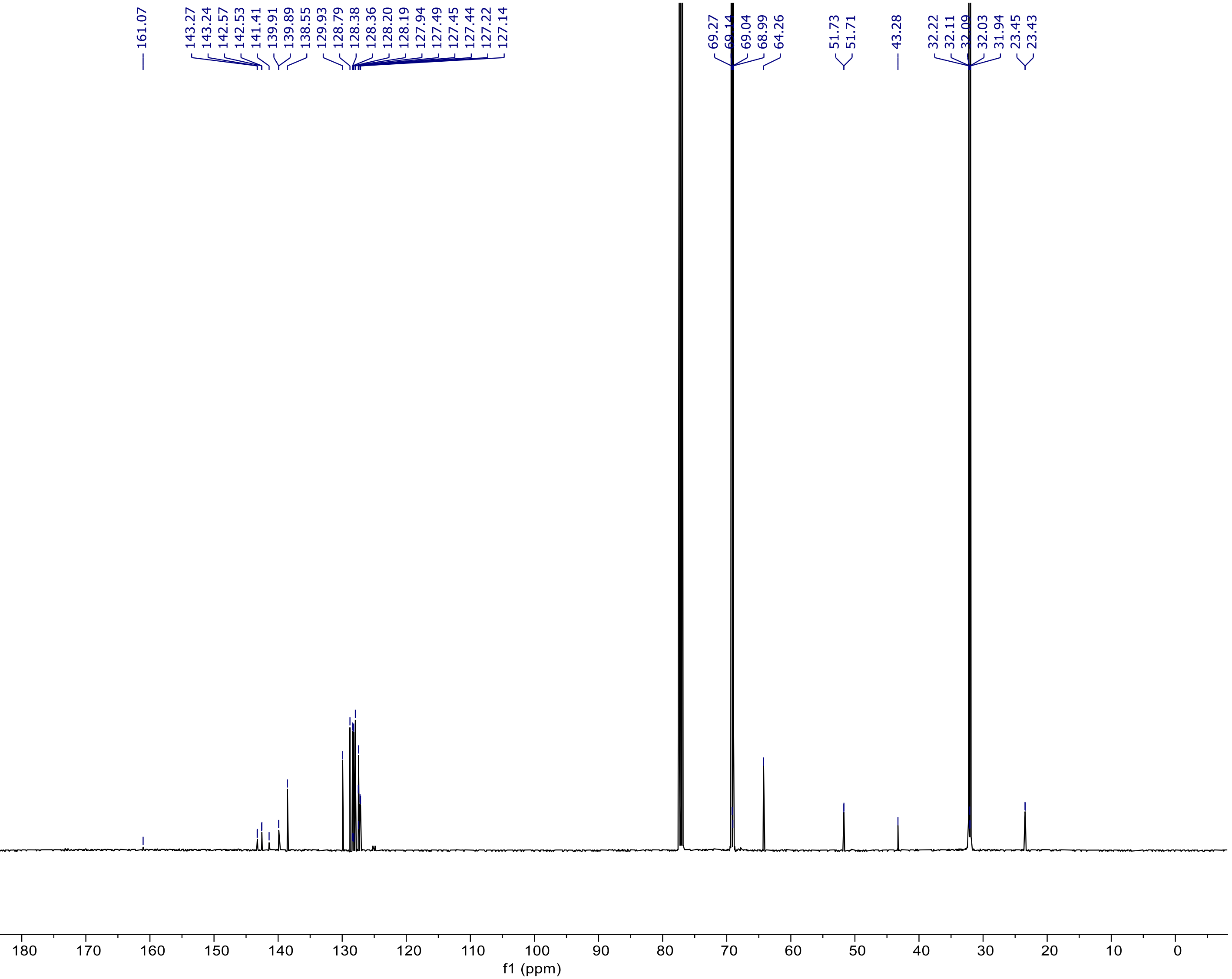

2,3-[$^{13}C_2$]-**4a** – ^{1}H NMR (500 MHz, $CDCl_3$)

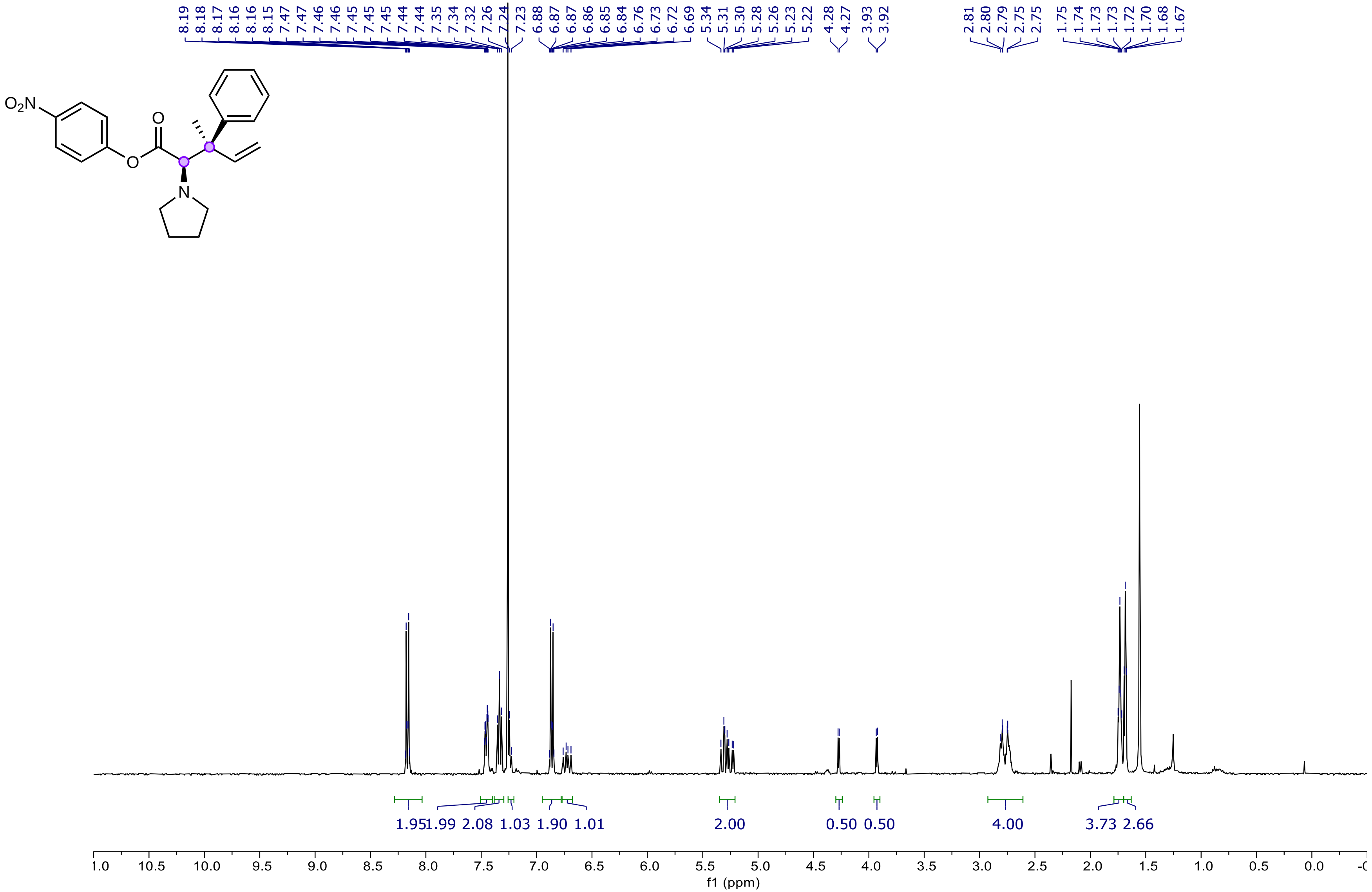

2,3-[$^{13}C_2$]-**4a** – ^{13}C NMR (126 MHz, $CDCl_3$)

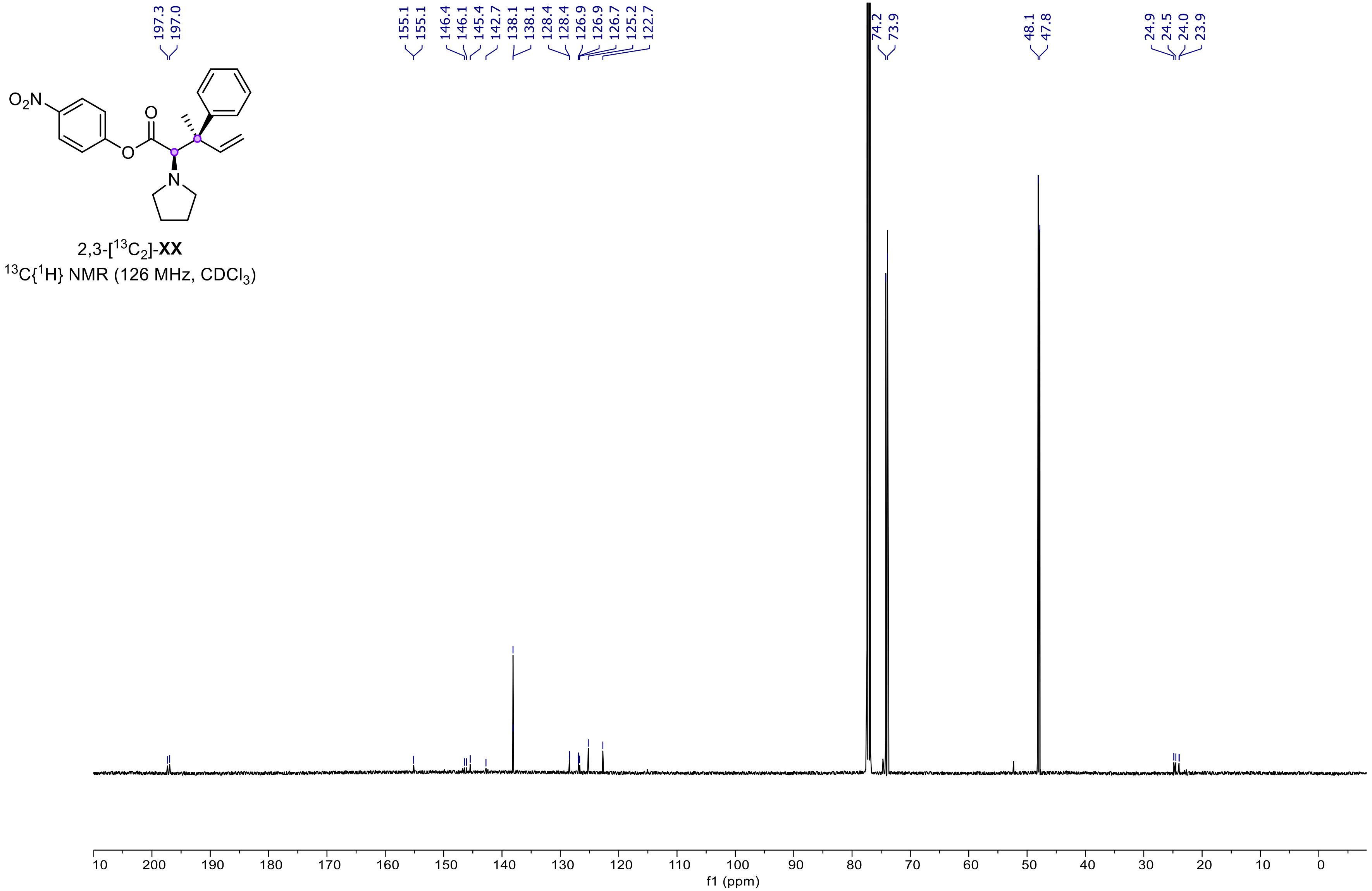

5 – ^{1}H NMR (500 MHz, $CDCl_3$)

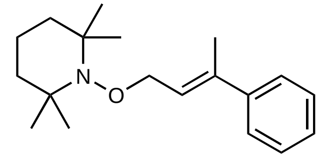

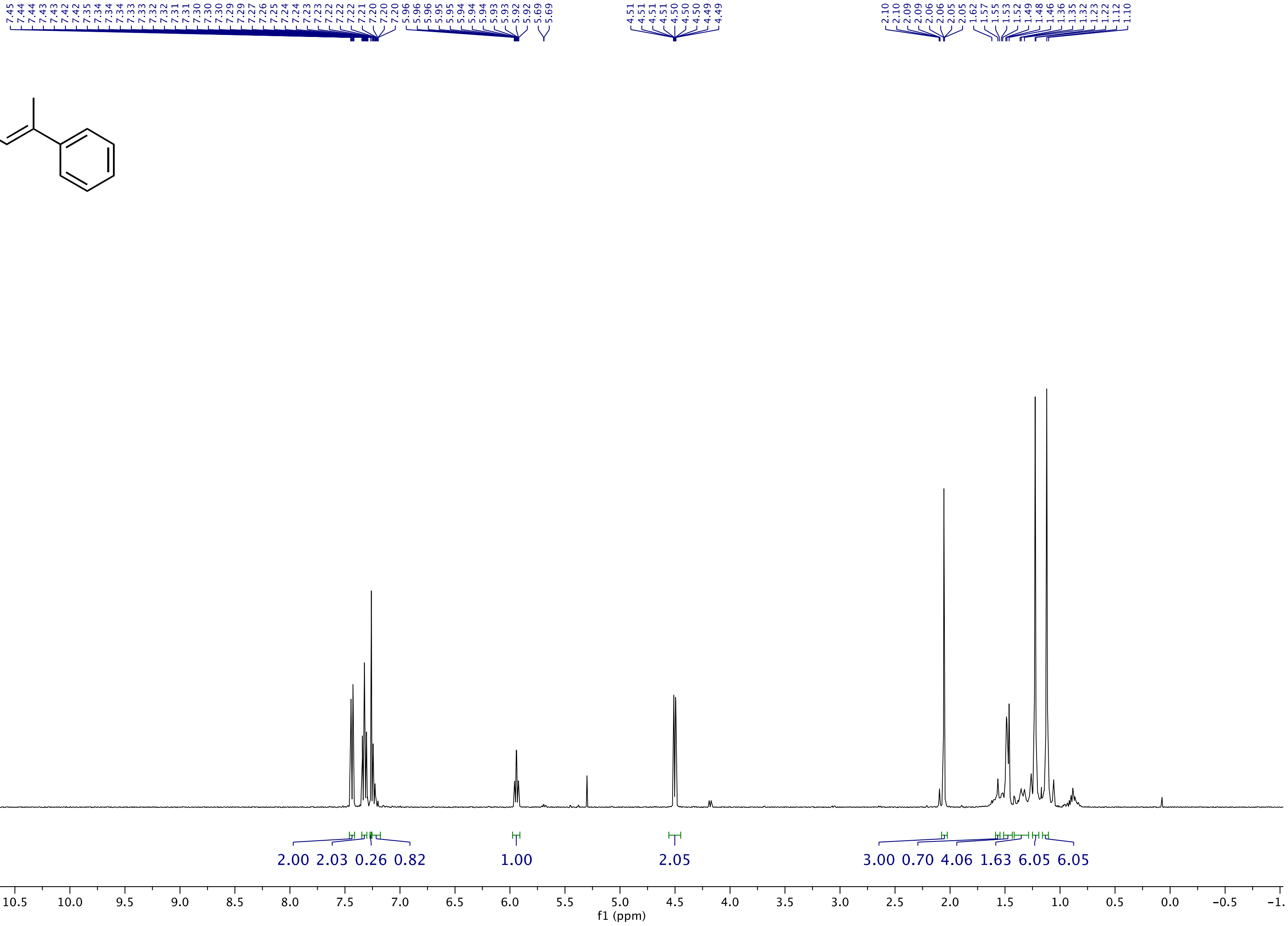

5 – ^{13}C NMR (126 MHz, $CDCl_3$)

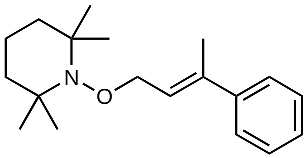

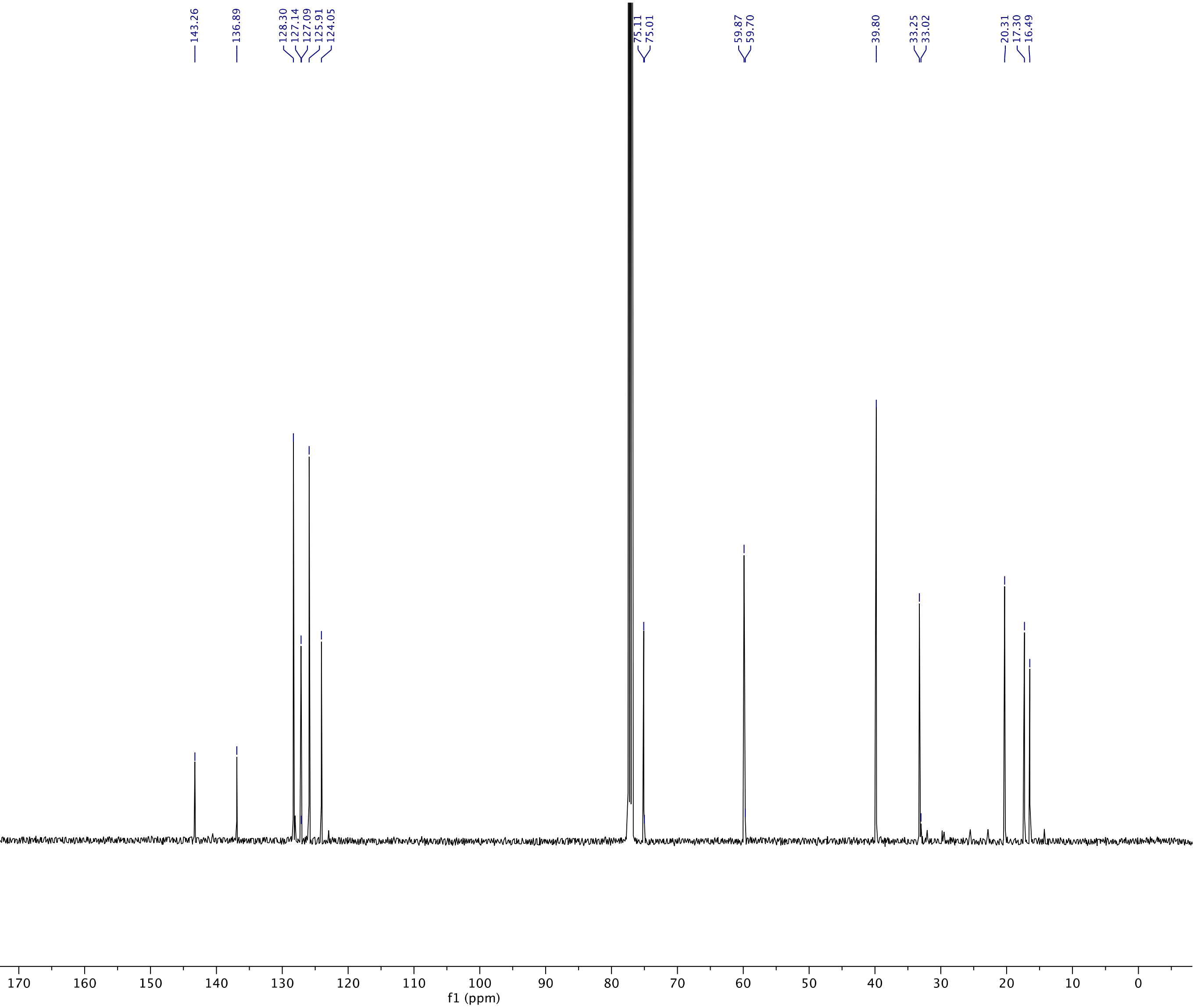

6 – ^{1}H NMR (500 MHz, $CDCl_3$)

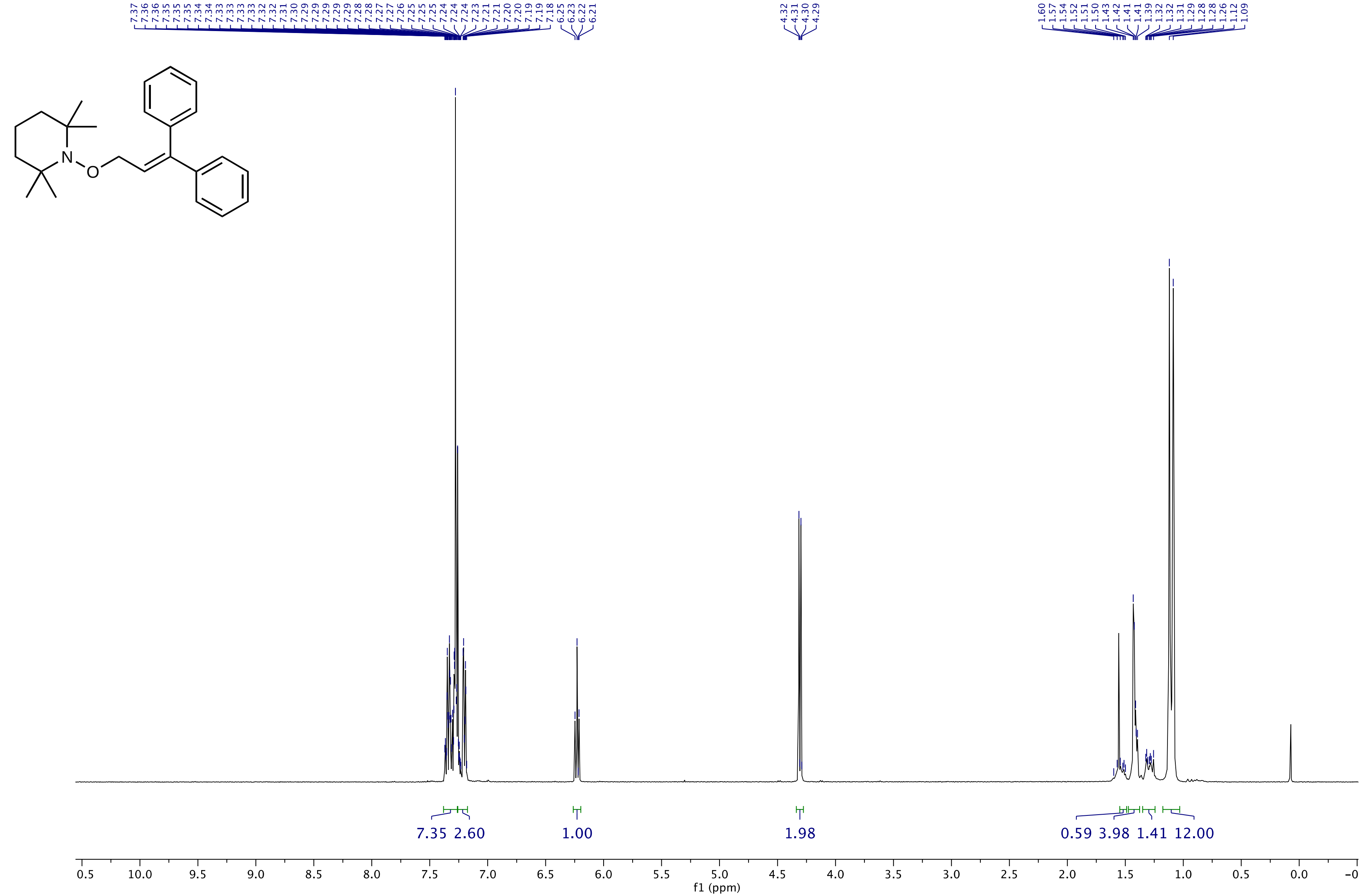

6 – ^{13}C NMR (126 MHz, $CDCl_3$)

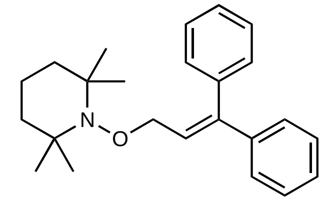

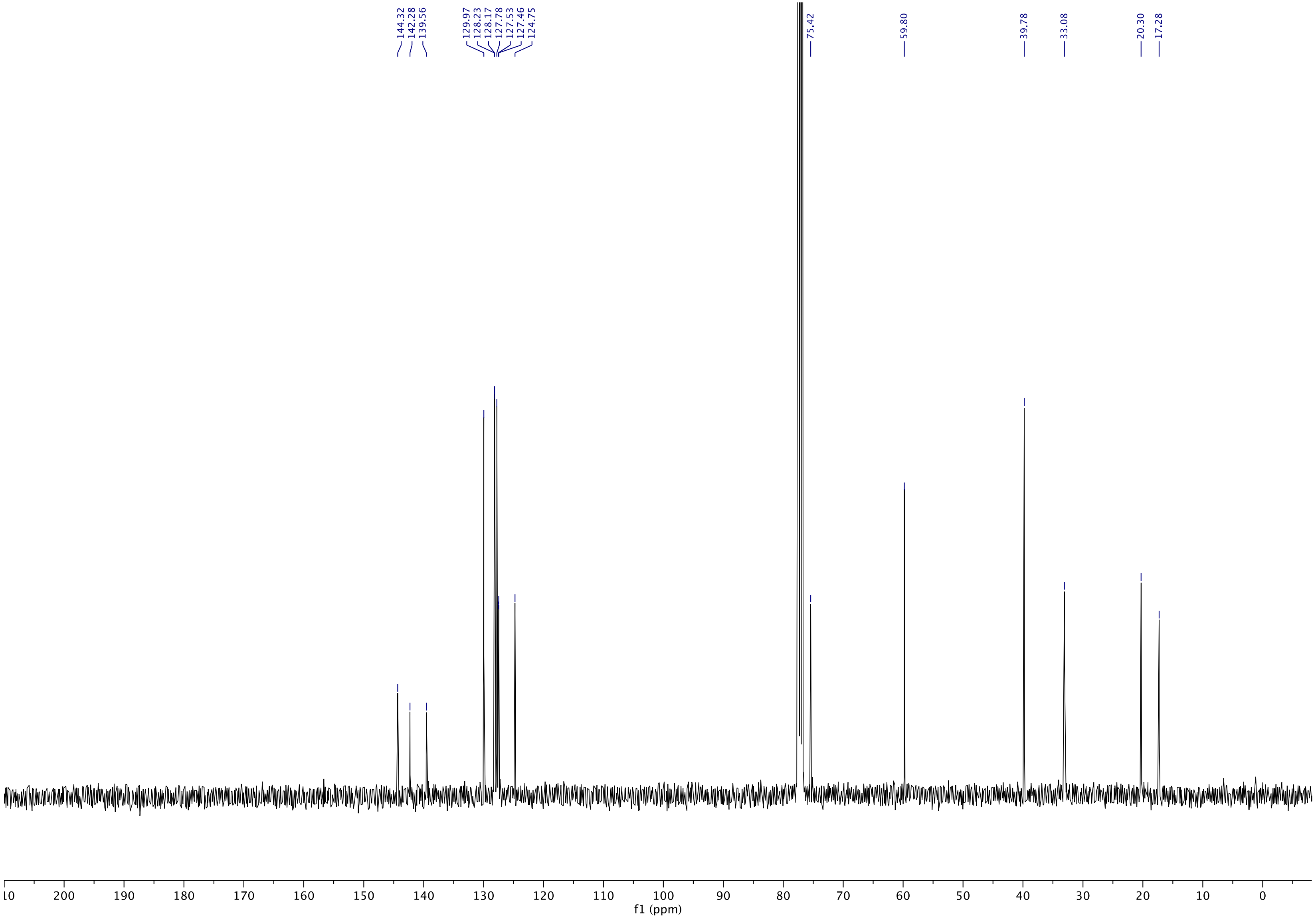
144.32
142.28
139.56
129.97
128.23
128.17
127.78
127.53
127.46
124.75
75.42
59.80
39.78
33.08
20.30
17.28
f1 (ppm)

K. HPLC traces

3a - Chiracel OJ-H (98:2 hexane/IPA, flow rate 2mLmin^{-1}, 211 nm, 40 °C) t_R (*R*): 14.7 min, t_R (*S*): 23.5 min, 91:9 er.

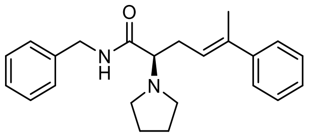

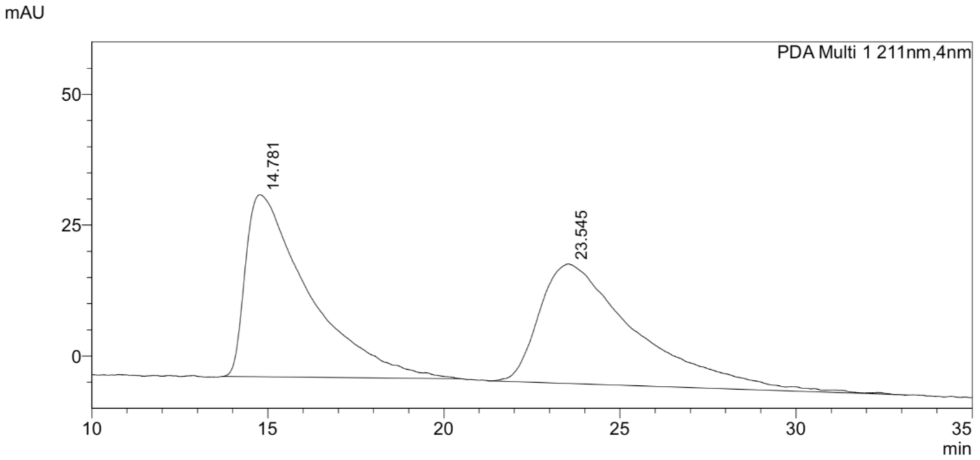

<Peak Table>

PDA Ch1 211nm

Peak#	Ret. Time	Area%
1	14.781	50.004
2	23.545	49.996
Total		100.000

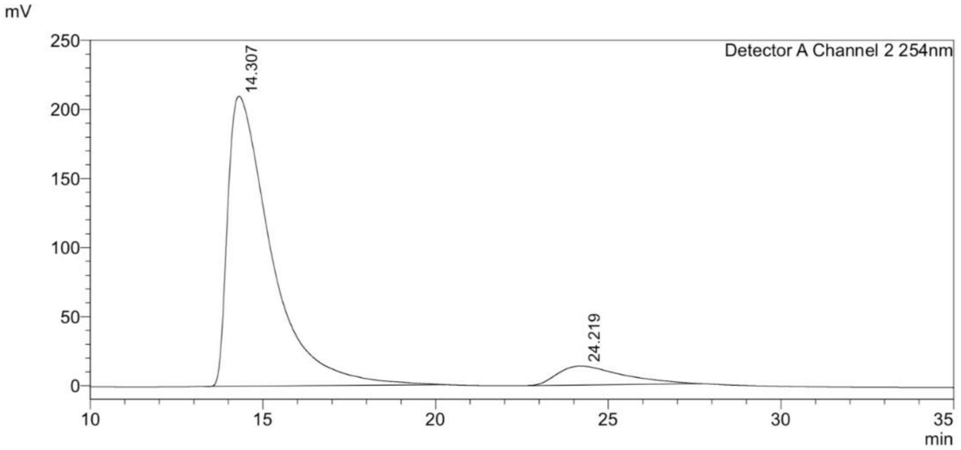

<Peak Table>

Detector A Channel 2 254nm

Peak#	Ret. Time	Area%
1	14.307	91.030
2	24.219	8.970
Total		100.000

syn-**4a** - Chiralcel OJ-H (99.6:0.4 hexane : IPA, flow rate 2 mLmin^{-1}, 254 nm, 40 °C) t_R (2*R*,3*R*): 17.8 min, t_R (2*S*,3*S*): 23.5 min, 22:78 er.

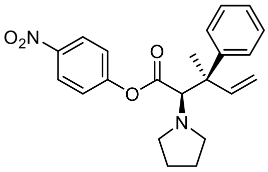

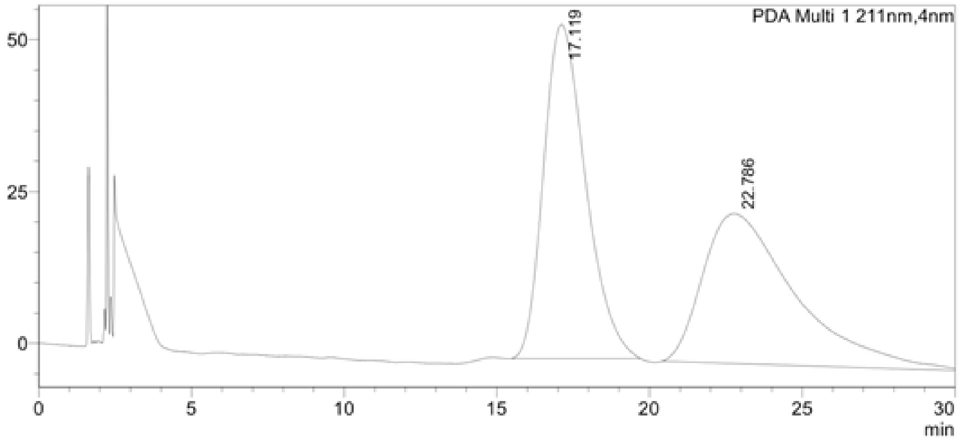

<Peak Table>

PDA Ch1 211nm

Peak#	Ret. Time	Area%
1	17.119	50.419
2	22.786	49.581
Total		100.000

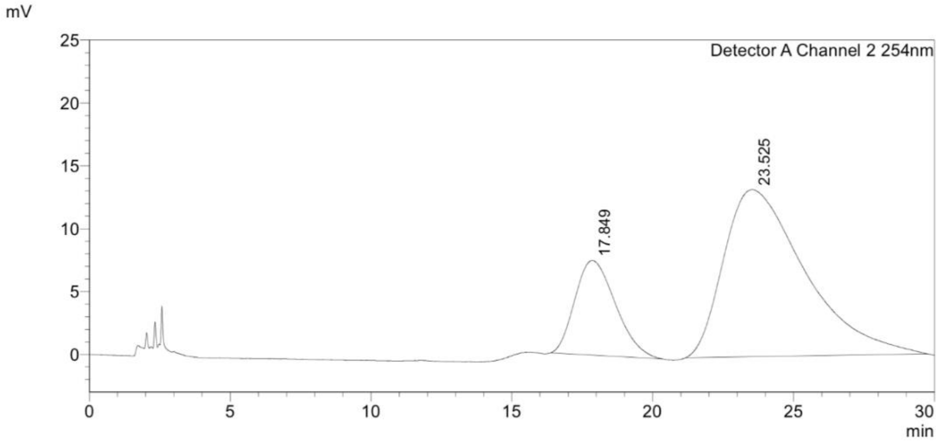

<Peak Table>

Detector A Channel 2 254nm

Peak#	Ret. Time	Area%
1	17.849	22.092
2	23.525	77.908
Total		100.000

3b - Chiracel OJ-H (95.5:2.5:2 hexane/IPA/Et_3N, flow rate 1.5 $mLmin^{-1}$, 30 °C) t_R (*R*): 13.3 min, t_R (*S*): 18.2 min, 89:11 er.

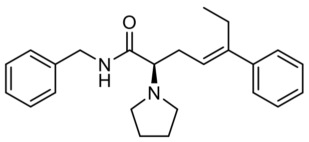

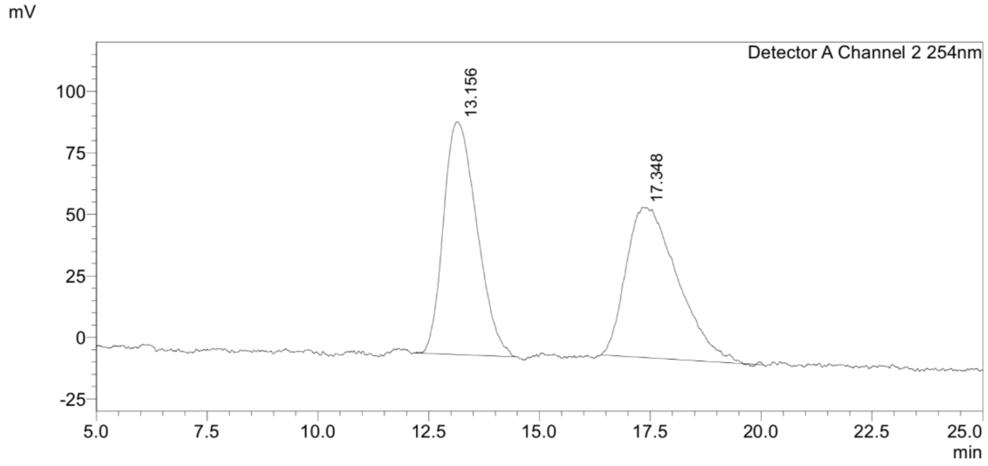

<Peak Table>

Detector A Channel 2 254nm

Peak#	Ret. Time	Area%
1	13.156	49.995
2	17.348	50.005
Total		100.000

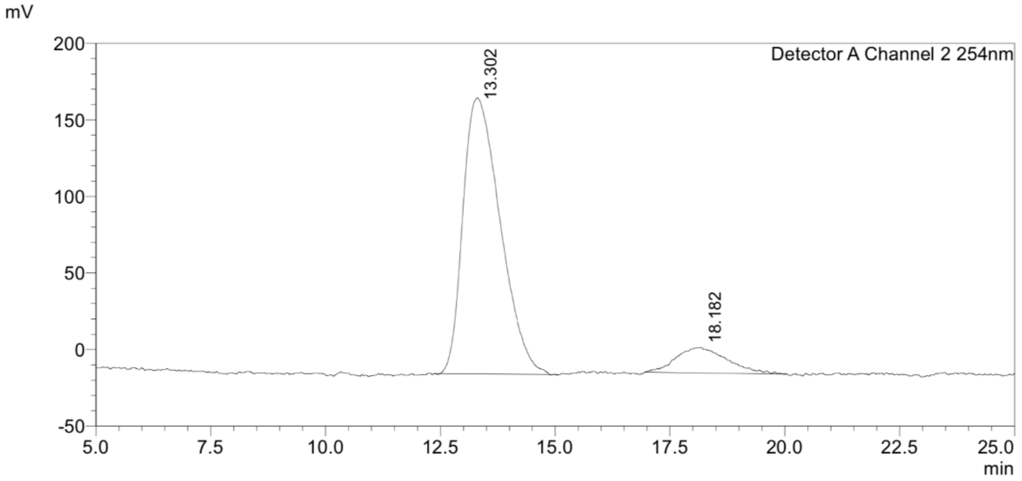

<Peak Table>

Detector A Channel 2 254nm

Peak#	Ret. Time	Area%
1	13.302	88.798
2	18.182	11.202
Total		100.000

3c - Chiralpak IC (98:2 hexane/IPA, flow rate 2.0 mLmin^{-1}, 40 °C) t_R (*S*): 26.4 min, t_R (*R*): 30.8 min, 87:13 er.

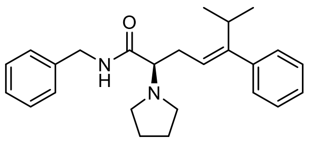

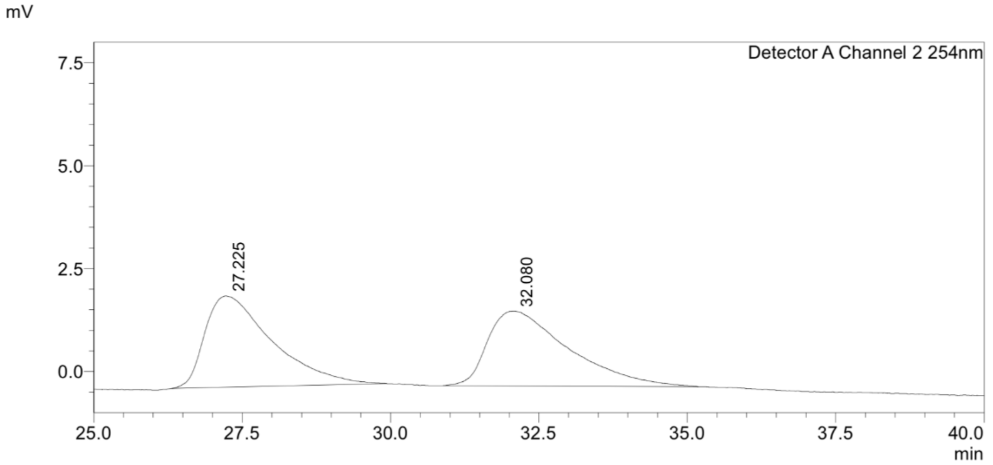

<Peak Table>

Detector A Channel 2 254nm

Peak#	Ret. Time	Area%
1	27.225	50.222
2	32.080	49.778
Total		100.000

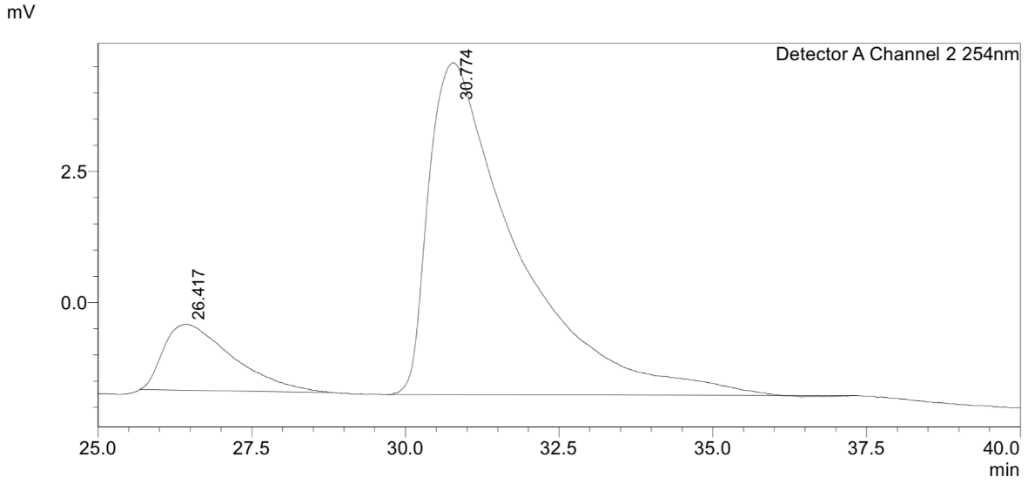

<Peak Table>

Detector A Channel 2 254nm

Peak#	Ret. Time	Area%
1	26.417	13.357
2	30.774	86.643
Total		100.000

3d – Chiral ID_(95:5 hexane/IPA, flow rate 1.5 mLmin^{-1}, 40 °C): t_R (*S*): 26.5 min, t_R (*R*): 30.0 min, 83:17 er.

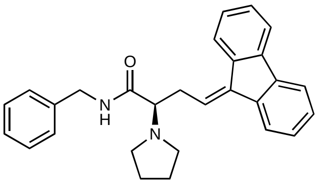

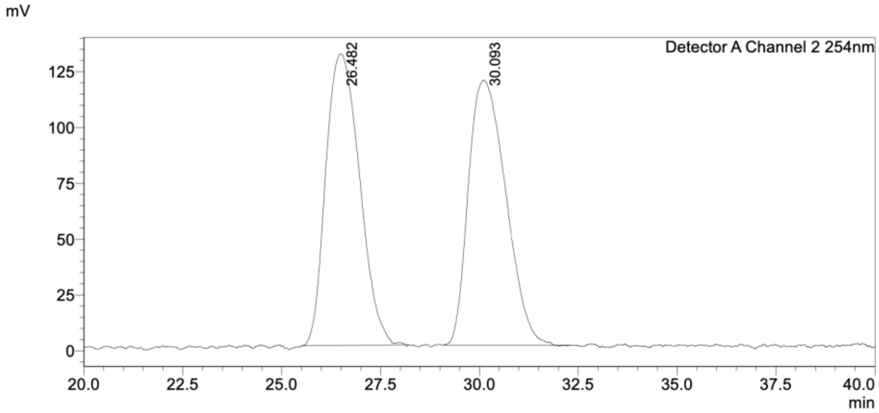

<Peak Table>

Detector A Channel 2 254nm

Peak#	Ret. Time	Area%
1	26.482	49.859
2	30.093	50.141
Total		100.000

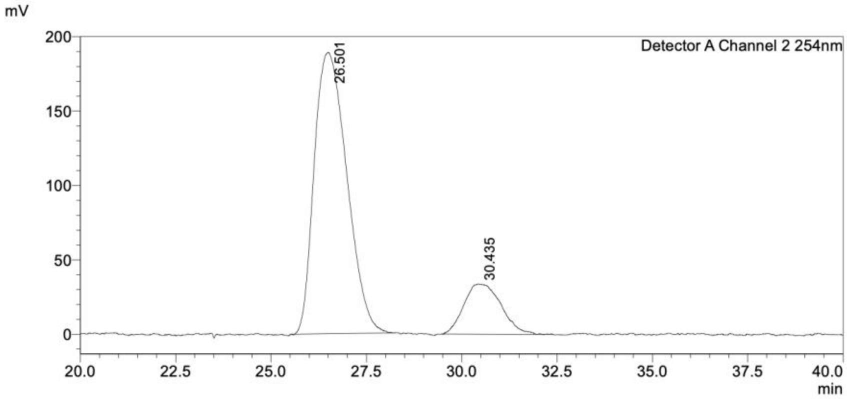

<Peak Table>

Detector A Channel 2 254nm

Peak#	Ret. Time	Area%
1	26.501	83.134
2	30.435	16.866
Total		100.000

3e - Chiracel AD-H (98.5:1.5 hexane/IPA, flow rate 2.0 mLmin^{-1}, 40 °C) t_R (*S*): 27.0 min, t_R (*R*): 33.8 min, 77:23 er.

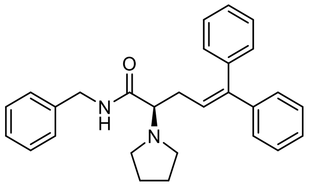

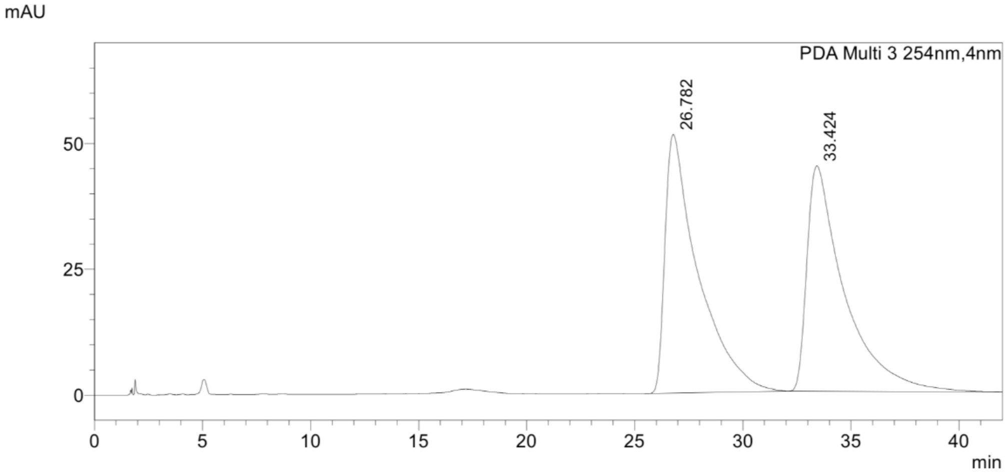

<Peak Table>

PDA Ch3 254nm

Peak#	Ret. Time	Area%
1	26.782	50.485
2	33.424	49.515
Total		100.000

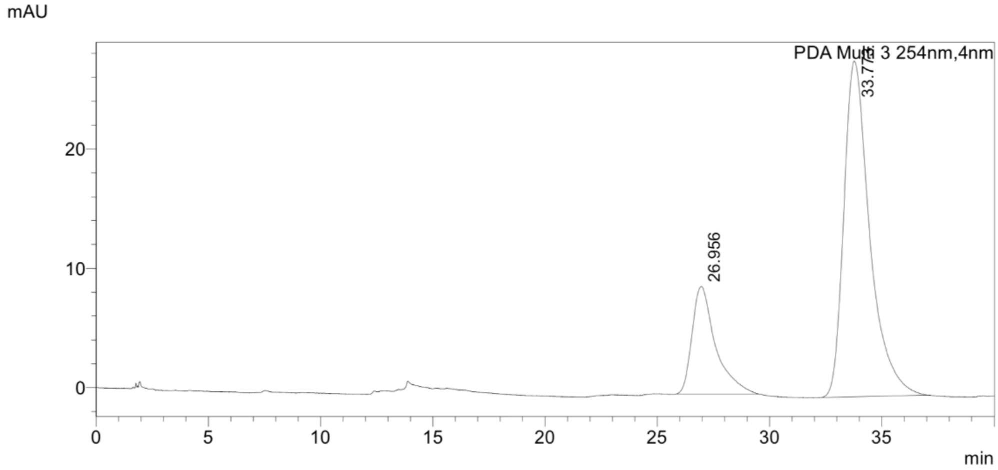

<Peak Table>

PDA Ch3 254nm

Peak#	Ret. Time	Area%
1	26.956	22.589
2	33.773	77.411
Total		100.000

3f - Chiracel OJ-H (98:2 hexane/IPA, flow rate 2.0 mLmin^{-1}, 40 °C) t_R (major): 10.2 min, t_S (minor): 14.5 min, 93:7 er.

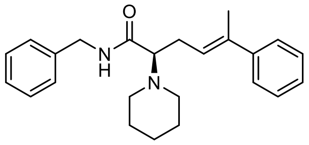

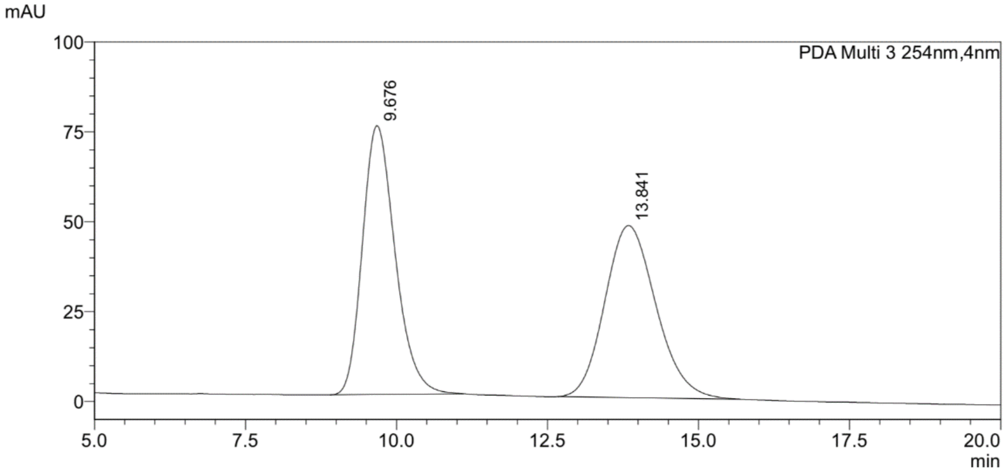

<Peak Table>

PDA Ch3 254nm

Peak#	Ret. Time	Area%
1	9.676	49.986
2	13.841	50.014
Total		100.000

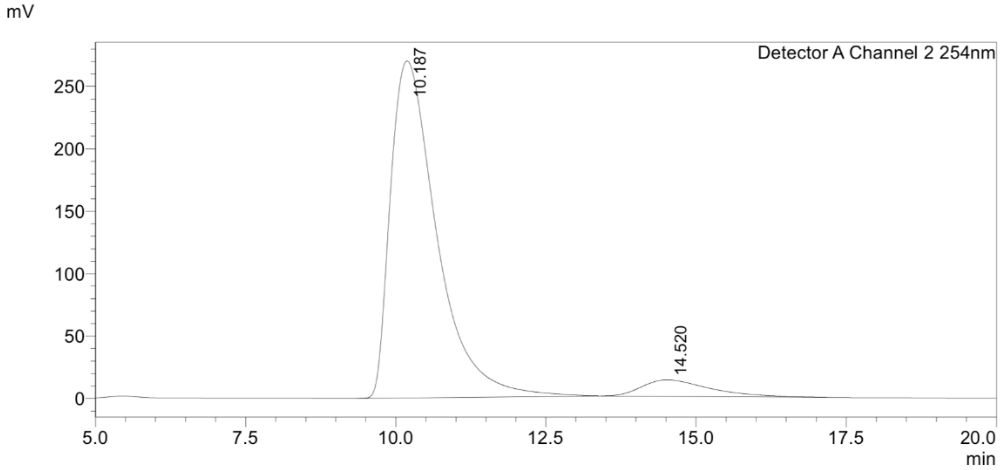

<Peak Table>

Detector A Channel 2 254nm

Peak#	Ret. Time	Area%
1	10.187	93.375
2	14.520	6.625
Total		100.000

3g - Chiracel OJ-H (98:2 hexane/IPA, flow rate 2.0 mLmin^{-1}, 40 °C) t_R (*R*): 9.4 min, t_R (*S*): 15.1 min, 92:8 er.

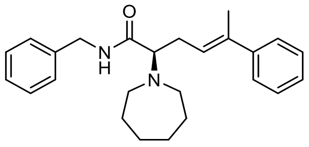

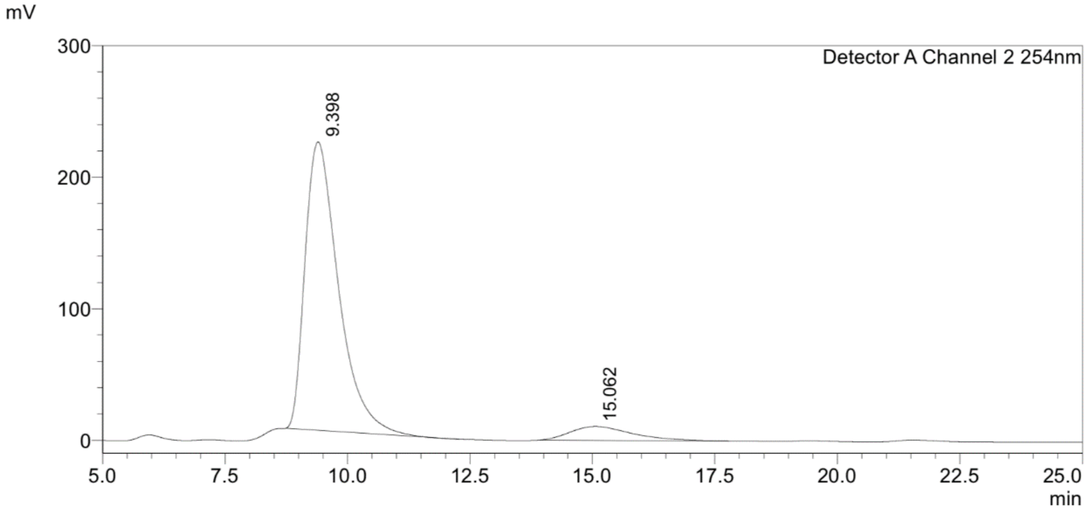

<Peak Table>

Detector A Channel 2 254nm

Peak#	Ret. Time	Area%
1	9.398	91.864
2	15.062	8.136
Total		100.000

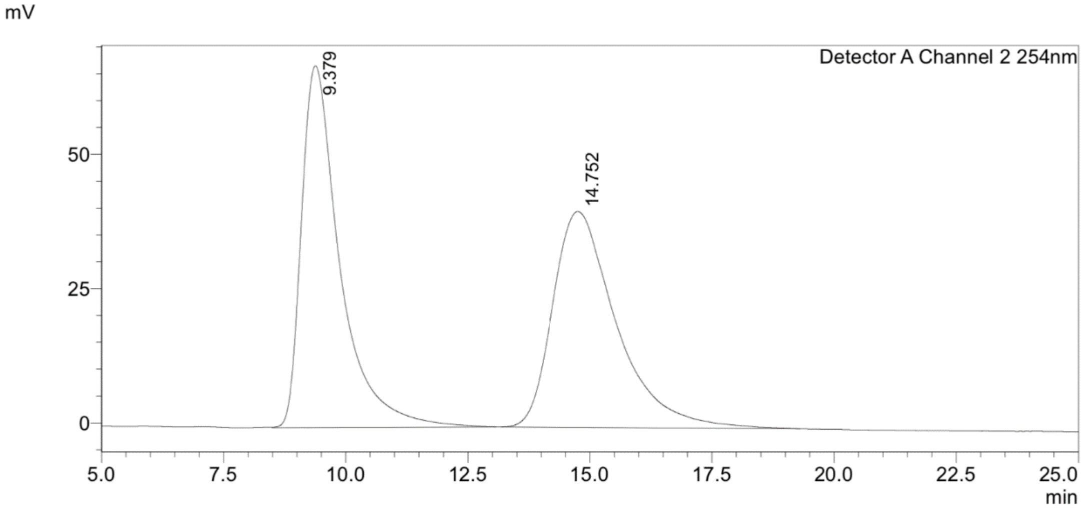

<Peak Table>

Detector A Channel 2 254nm

Peak#	Ret. Time	Area%
1	9.379	50.286
2	14.752	49.714
Total		100.000

3h - Chiralpak ID (98:2 hexane/IPA, flow rate 2.0 mLmin^{-1}, 40 °C) t_R (major): 21.0 min, t_S (minor): 23.8 min, 71:29 er.

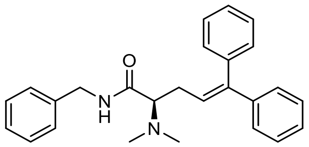

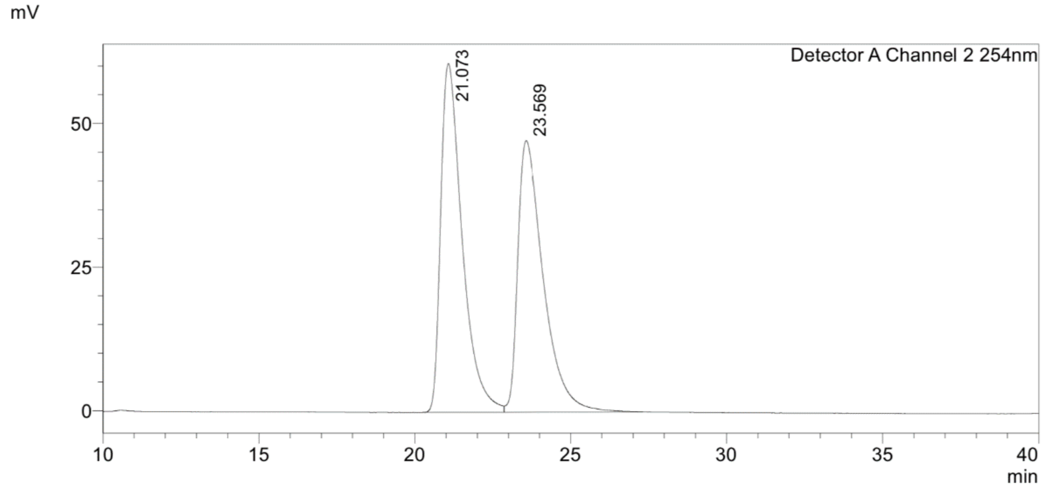

<Peak Table>

Detector A Channel 2 254nm

Peak#	Ret. Time	Area%
1	21.073	51.824
2	23.569	48.176
Total		100.000

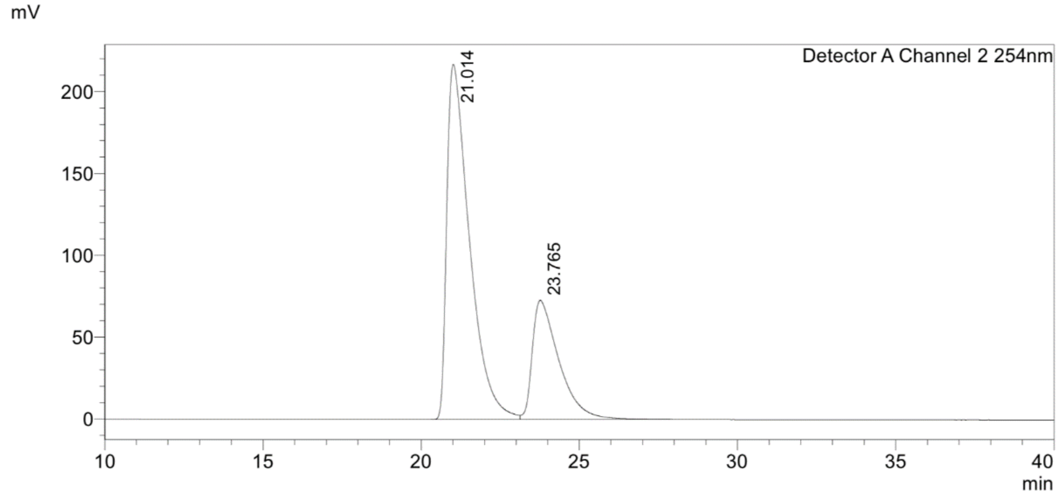

<Peak Table>

Detector A Channel 2 254nm

Peak#	Ret. Time	Area%
1	21.014	71.484
2	23.765	28.516
Total		100.000

3i - Chiracel OJ-H (98:2 hexane/IPA, flow rate 2.0 mLmin^{-1}, 40 °C) t_R (major): 19.3 min, t_S (minor): 31.1 min, 90:10 er.

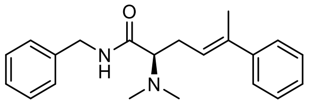

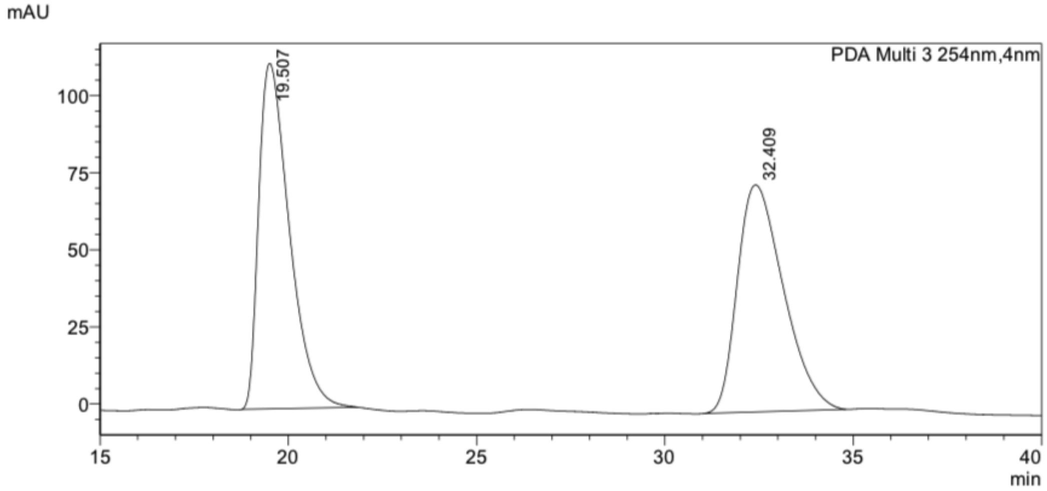

<Peak Table>

PDA Ch3 254nm

Peak#	Ret. Time	Area%
1	19.507	50.318
2	32.409	49.682
Total		100.000

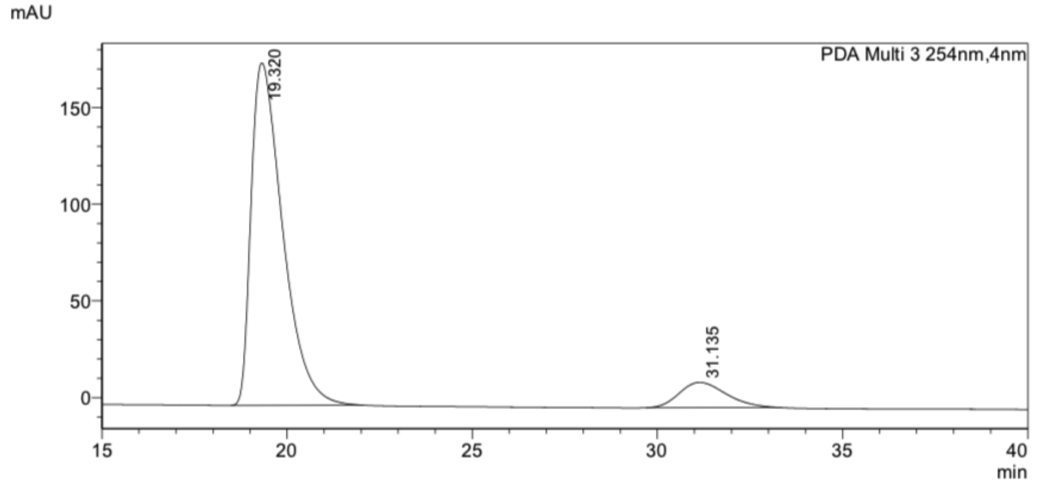

<Peak Table>

PDA Ch3 254nm

Peak#	Ret. Time	Area%
1	19.320	90.051
2	31.135	9.949
Total		100.000

4i - Chiracel OJ-H (99.5:0.5 hexane/IPA, flow rate 1.0 mLmin^{-1}, 30 °C) t_S (minor): 37.5 min, t_R (,ajor): 43.6 min, 90:10 er.

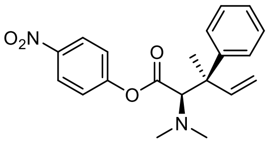

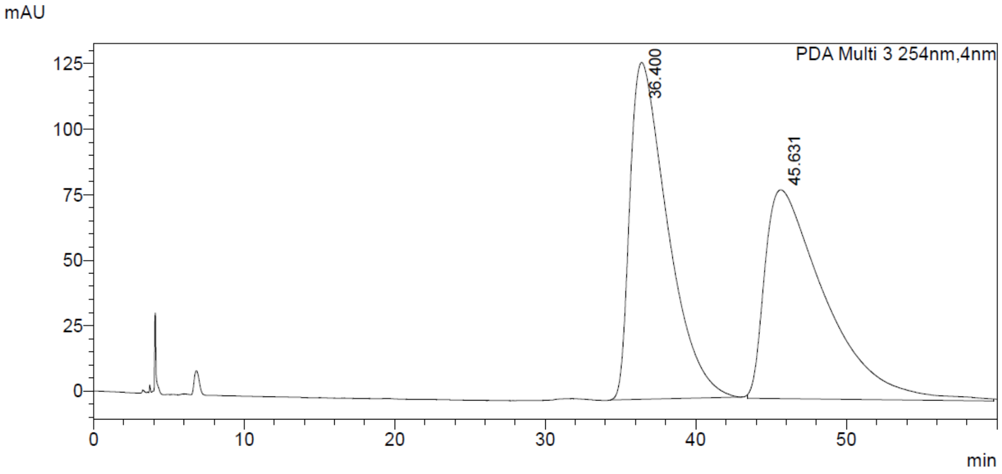

<Peak Table>

PDA Ch3 254nm

Peak#	Ret. Time	Area%
1	36.400	50.103
2	45.631	49.897
Total		100.000

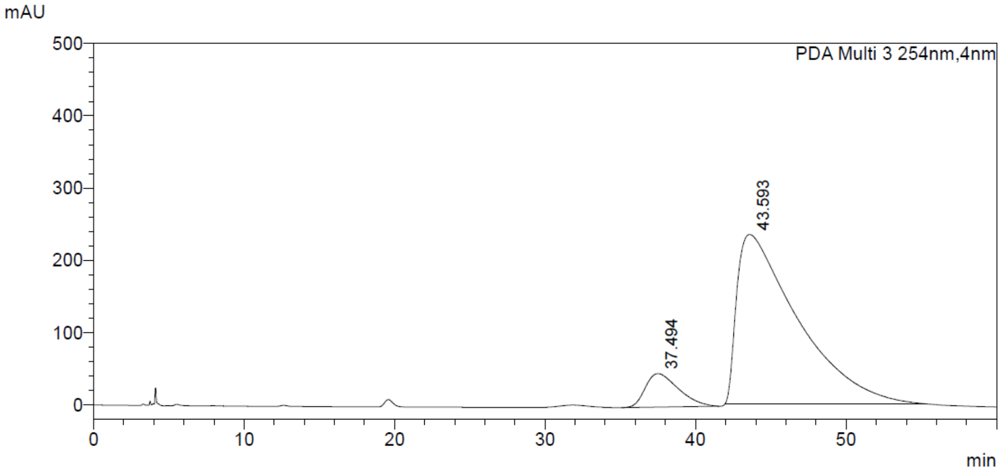

<Peak Table>

PDA Ch3 254nm

Peak#	Ret. Time	Area%
1	37.494	9.714
2	43.593	90.286
Total		100.000

4j - Chiracel OJ-H (99:1 hexane/IPA, flow rate 2 mLmin^{-1}, 40 °C) t_R (2*R*,3*R*): 33.7 min, t_R (2*S*,3*S*): 52.3 min, 77:23 er.

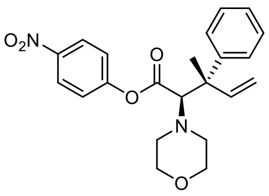

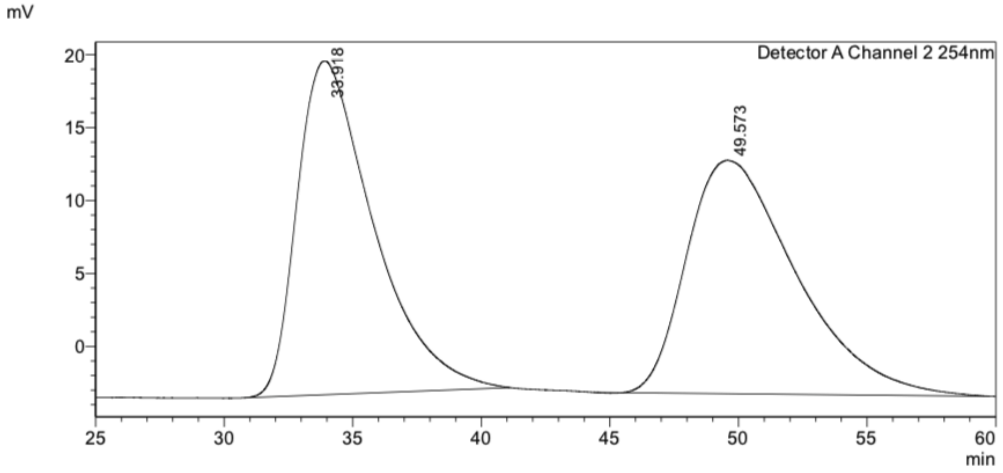

<Peak Table>

Detector A Channel 2 254nm

Peak#	Ret. Time	Area%
1	33.918	49.923
2	49.573	50.077
Total		100.000

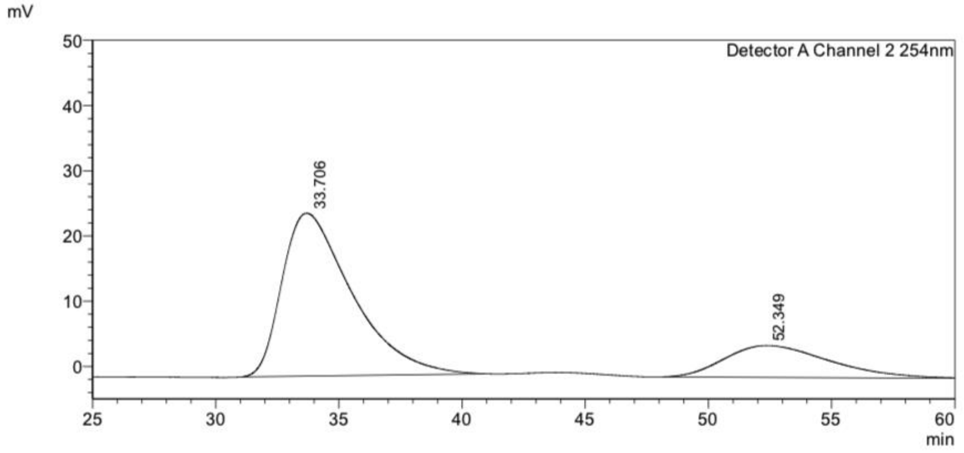

<Peak Table>

Detector A Channel 2 254nm

Peak#	Ret. Time	Area%
1	33.706	77.478
2	52.349	22.522
Total		100.000

3k - Chiracel OJ-H (95.5:2.5:2 hexane/IPA/Et_3N, flow rate 1.5 mLmin^{-1}, 30 °C) t_R (*R*): 15.3 min, t_R (*S*): 19.1 min, 91:9 er.

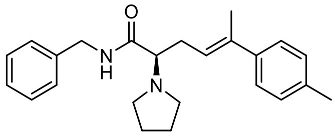

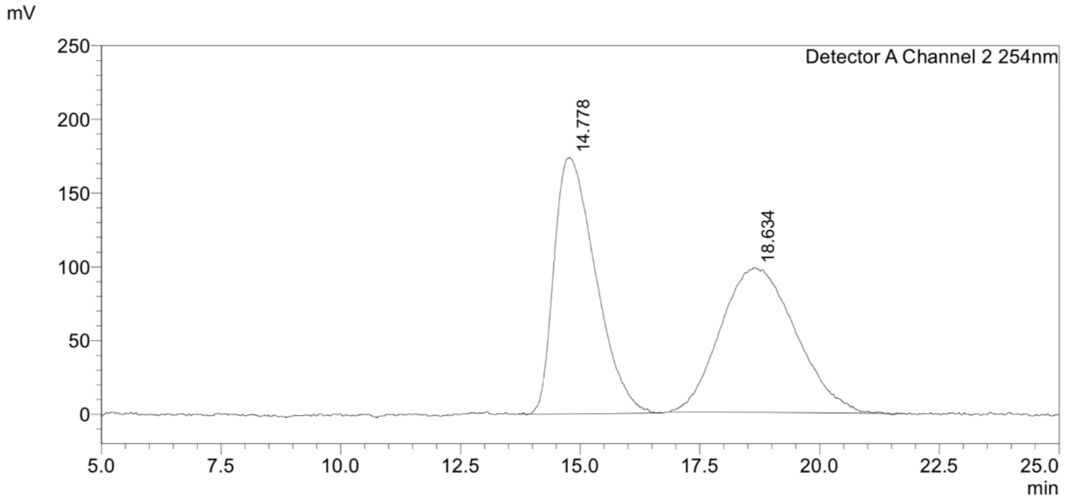

<Peak Table>

Detector A Channel 2 254nm

Peak#	Ret. Time	Area%
1	14.778	49.658
2	18.634	50.342
Total		100.000

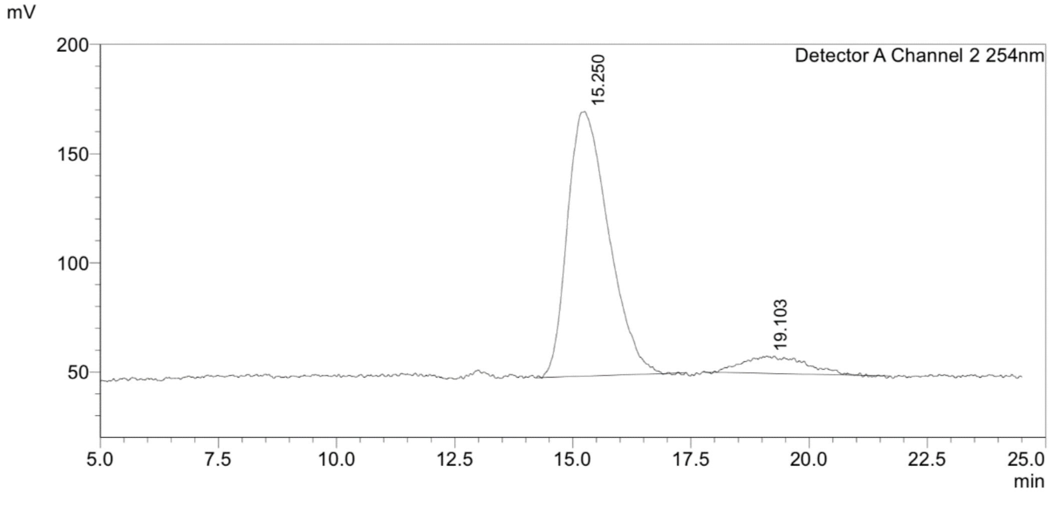

<Peak Table>

Detector A Channel 2 254nm

Peak#	Ret. Time	Area%
1	15.250	90.950
2	19.103	9.050
Total		100.000

3l - Chiracel OJ-H (98:2 hexane/IPA, flow rate 2.0 mLmin^{-1}, 40 °C) t_R (*R*): 11.7 min, t_R (*S*): 15.9 min, 91:9 er.

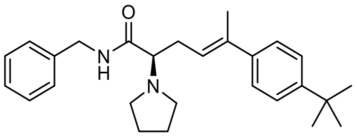

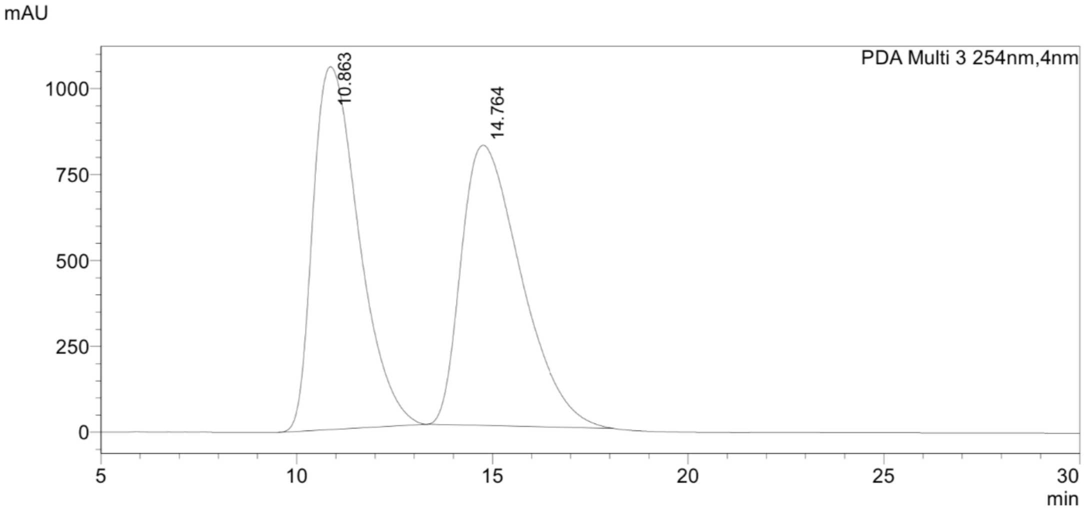

<Peak Table>

PDA Ch3 254nm

Peak#	Ret. Time	Area%
1	10.863	49.776
2	14.764	50.224
Total		100.000

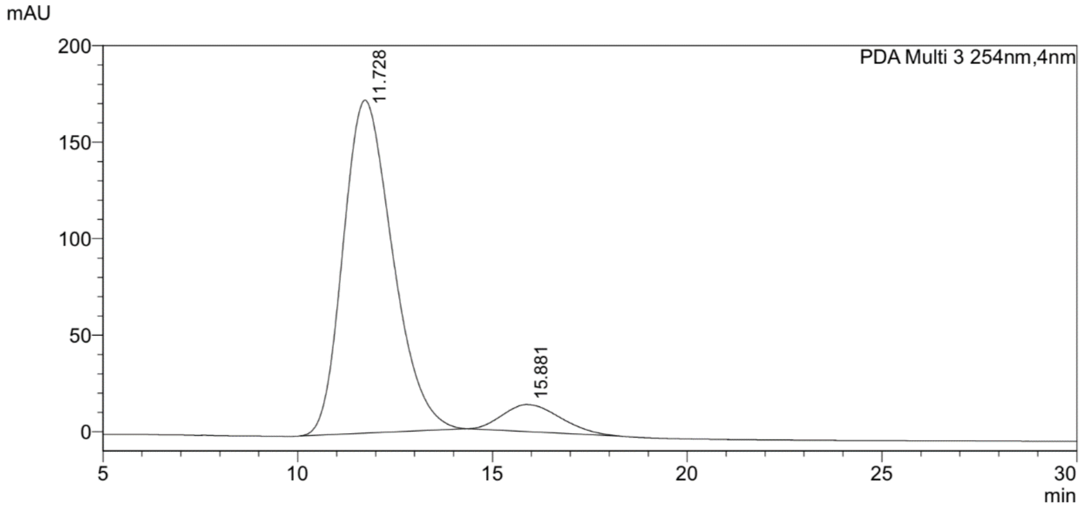

<Peak Table>

PDA Ch3 254nm

Peak#	Ret. Time	Area%
1	11.728	91.247
2	15.881	8.753
Total		100.000

3m - Chiracel AD-H (95:5 hexane/IPA, flow rate 2.0 mLmin^{-1}, 40 °C) t_R (*R*): 21.8 min, t_R (*S*): 32.0 min, 85:15 er.

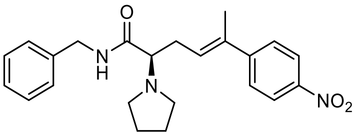

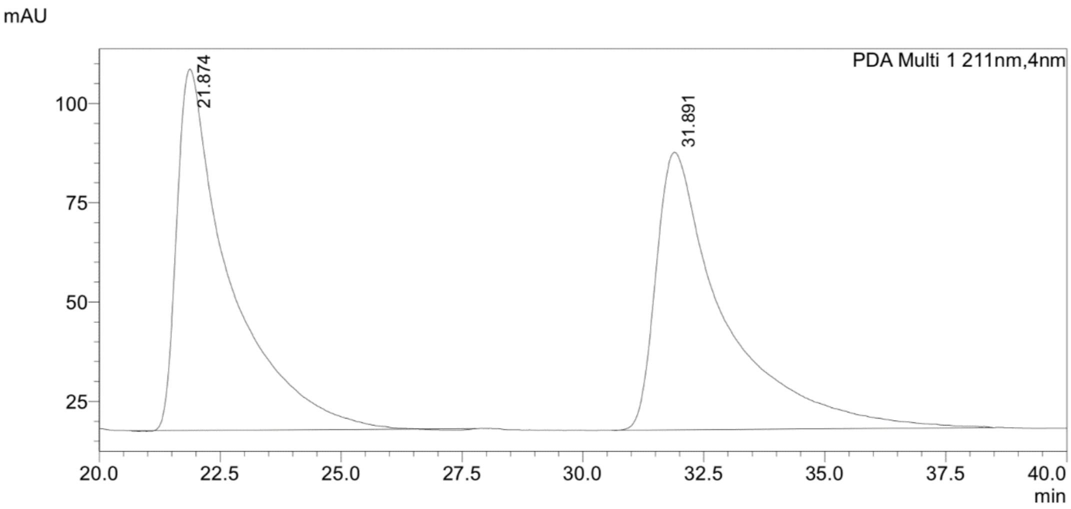

<Peak Table>

PDA Ch1 211nm

Peak#	Ret. Time	Area%
1	21.874	49.928
2	31.891	50.072
Total		100.000

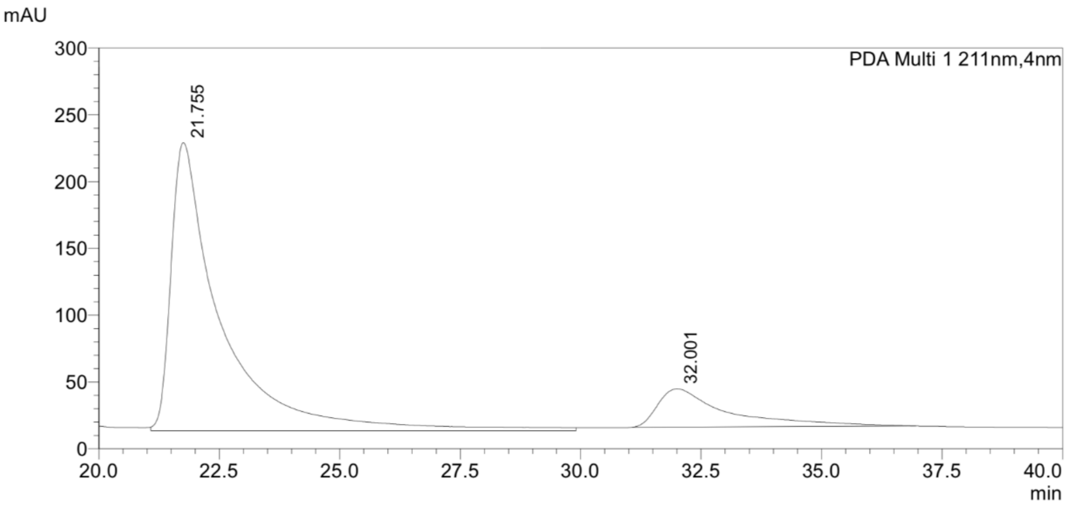

<Peak Table>

PDA Ch1 211nm

Peak#	Ret. Time	Area%
1	21.755	84.566
2	32.001	15.434
Total		100.000

3n - Chiracel OJ-H (99:1 hexane/IPA, flow rate 2.0 mLmin^{-1}, 40 °C) t_R (*R*): 31.8 min, t_R (*S*): 39.9 min, 87:13 er.

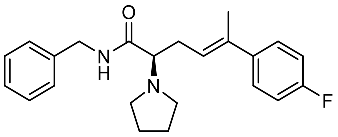

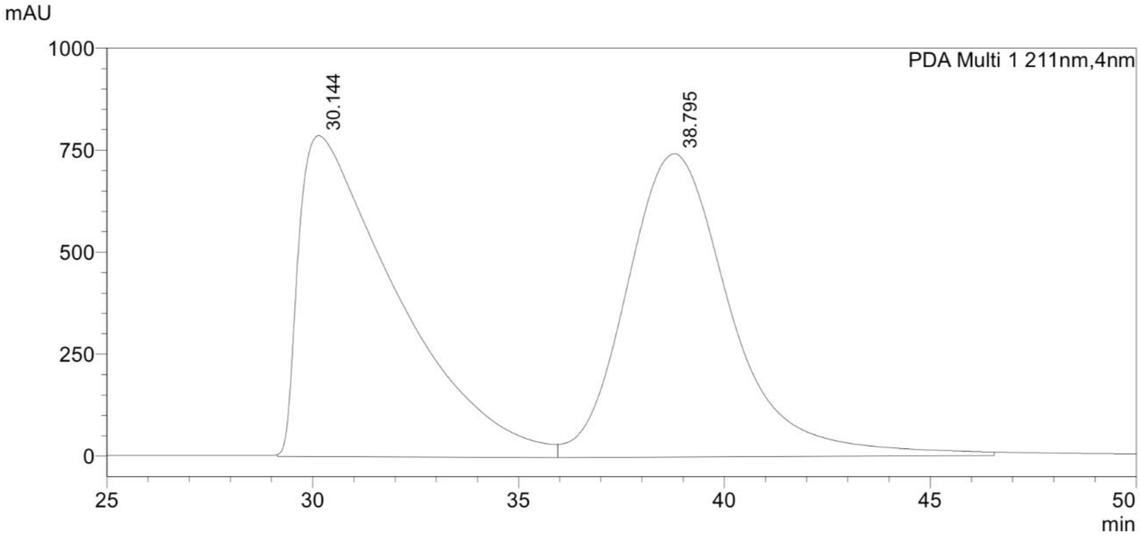

<Peak Table>

PDA Ch1 211nm

Peak#	Ret. Time	Area%
1	30.144	49.939
2	38.795	50.061
Total		100.000

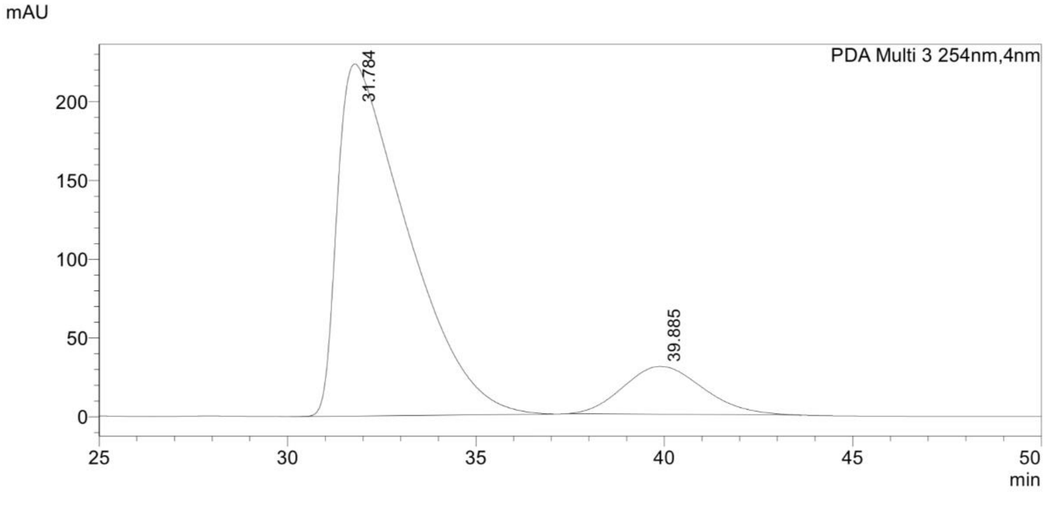

<Peak Table>

PDA Ch3 254nm

Peak#	Ret. Time	Area%
1	31.784	86.624
2	39.885	13.376
Total		100.000

3o - Chiracel OJ-H (98.5:1.5 hexane/IPA, flow rate 2.0mlmin^{-1}, 40 °C) t_R (*R*): 28.0 min, t_R (*S*): 35.1 min, 88:12 er.

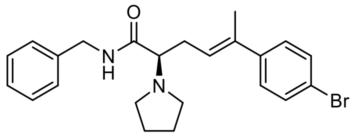

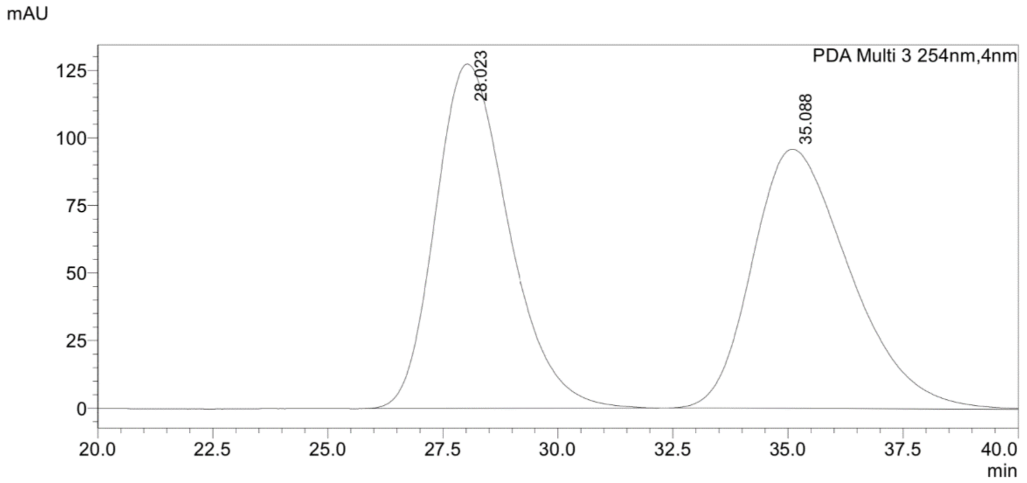

<Peak Table>

PDA Ch3 254nm

Peak#	Ret. Time	Area%
1	28.023	50.032
2	35.088	49.968
Total		100.000

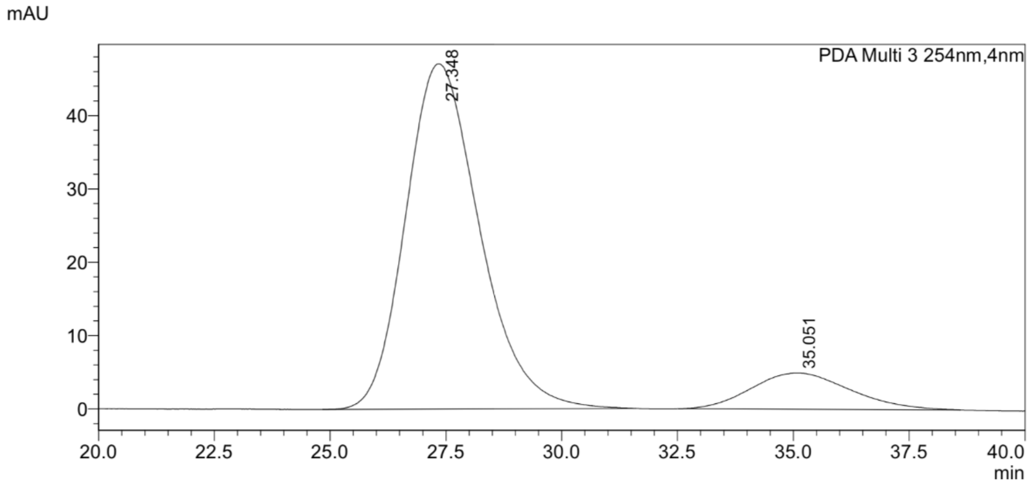

<Peak Table>

PDA Ch3 254nm

Peak#	Ret. Time	Area%
1	27.348	87.730
2	35.051	12.270
Total		100.000

3p - Chiracel OJ-H (97.2:1.8:1 hexane/IPA/Et_3N, flow rate 1.5 $mLmin^{-1}$, 30 °C) t_R (*R*): 27.1 min, t_R (*S*): 42.8 min, 91:9 er. While poor noise-to-signal ratio was observed when using Et_3N in the three-solvent eluent system, analytical separation of the enantiomers was unsuccessful without the use of Et_3N.

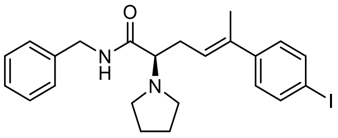

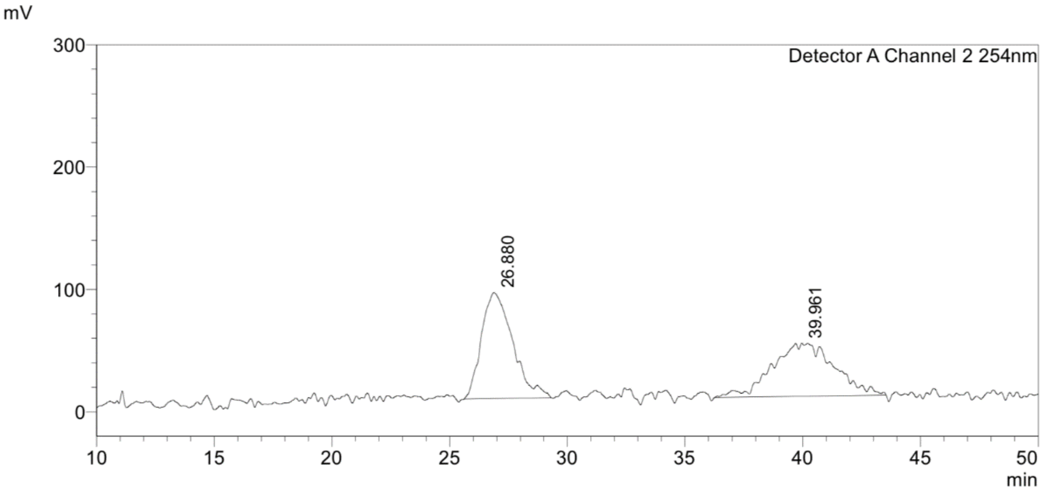

<Peak Table>

Detector A Channel 2 254nm

Peak#	Ret. Time	Area%
1	26.880	49.971
2	39.961	50.029
Total		100.000

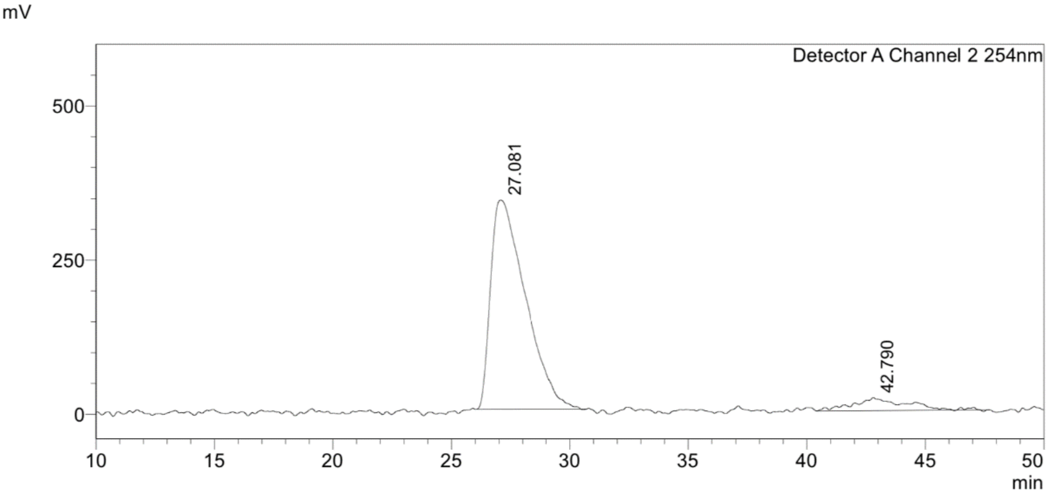

<Peak Table>

Detector A Channel 2 254nm

Peak#	Ret. Time	Area%
1	27.081	91.336
2	42.790	8.664
Total		100.000

3q - Chiracel AD-H (98:2 hexane/IPA, flow rate 2 mLmin^{-1}, 40 °C) t_R (*S*): 21.7 min, t_R: (*R*) 31.0 min, 85:15 er.

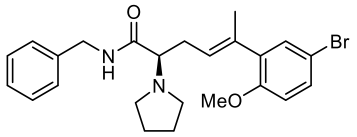

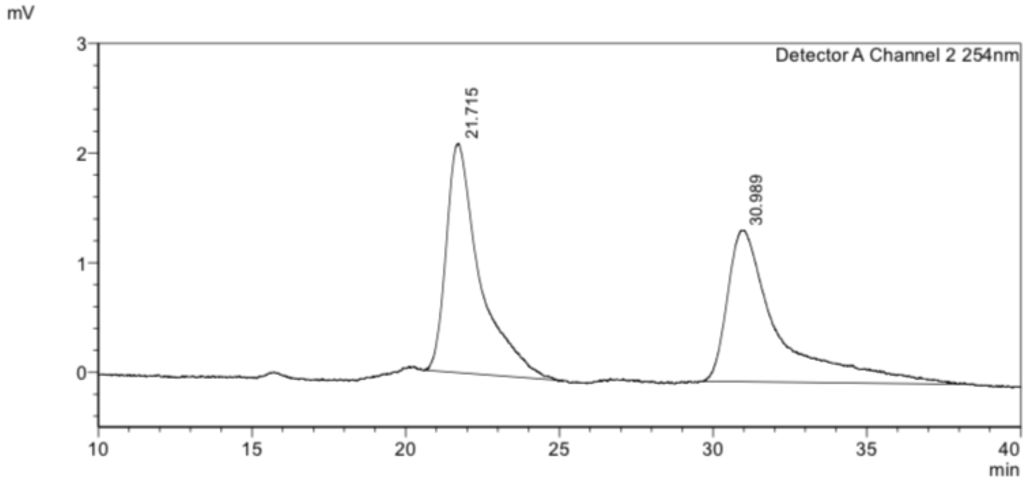

<Peak Table>

Detector A Channel 2 254nm

Peak#	Ret. Time	Area%
1	21.715	50.090
2	30.989	49.910
Total		100.000

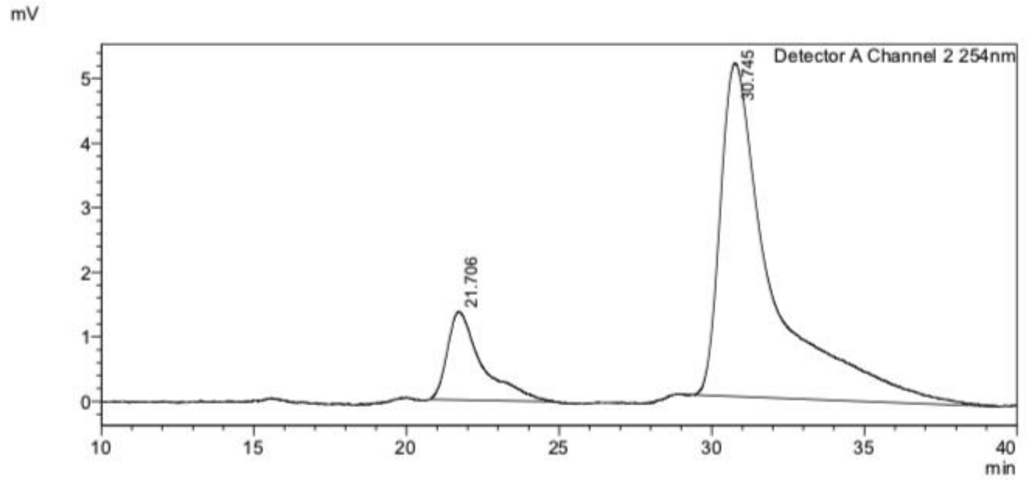

<Peak Table>

Detector A Channel 2 254nm

Peak#	Ret. Time	Area%
1	21.706	15.060
2	30.745	84.940
Total		100.000

3r - Chiracel OD-H (99:1 hexane/IPA, flow rate 1.0 mLmin^{-1}, 30 °C) t_R (*S*): 42.6 min, t_R (*R*): 51.1 min, 86:14 er.

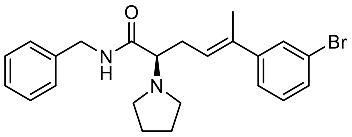

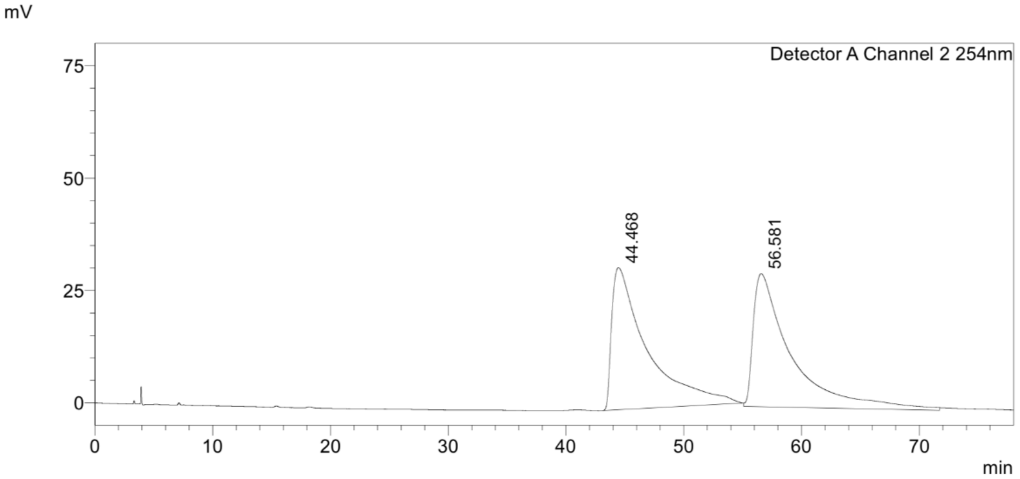

<Peak Table>

Detector A Channel 2 254nm

Peak#	Ret. Time	Area%
1	44.468	49.888
2	56.581	50.112
Total		100.000

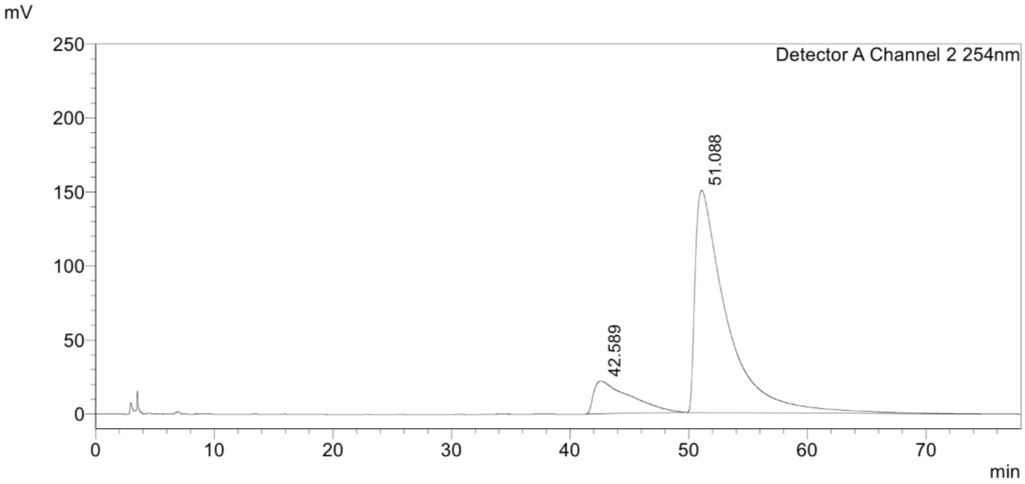

<Peak Table>

Detector A Channel 2 254nm

Peak#	Ret. Time	Area%
1	42.589	14.383
2	51.088	85.617
Total		100.000

3s - Chiracel OJ-H (96:3:1 hexane/IPA/Et_3N, flow rate 1.5 $mLmin^{-1}$, 254 nm, 40 °C, t_R (*R*): 13.7 min, t_R (*S*): 19.6 min, 91:9 er.

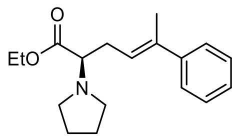

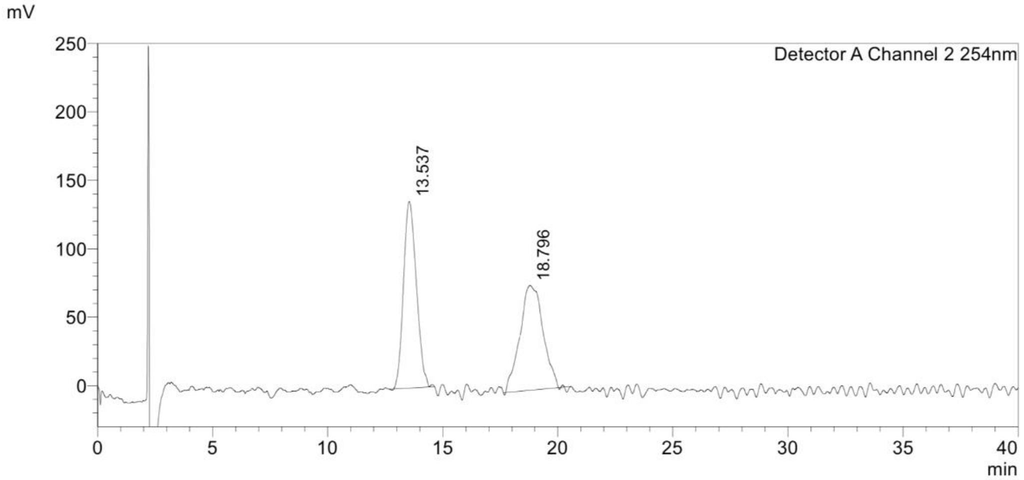

<Peak Table>

Detector A Channel 2 254nm

Peak#	Ret. Time	Area%
1	13.537	49.807
2	18.796	50.193
Total		100.000

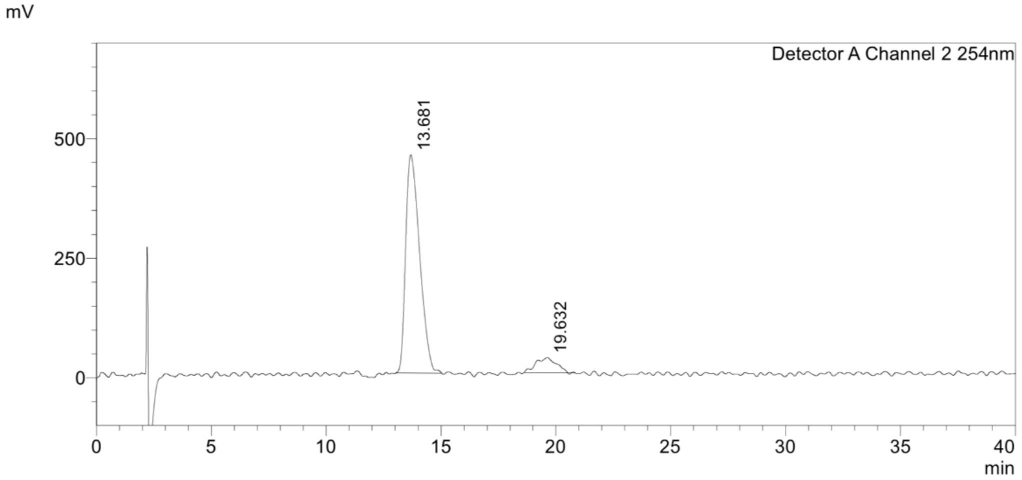

<Peak Table>

Detector A Channel 2 254nm

Peak#	Ret. Time	Area%
1	13.681	90.625
2	19.632	9.375
Total		100.000

3t - Chiralpak ID (92:8 hexane/IPA, flow rate 1.2 mLmin^{-1}, 254 nm, 40 °C, t_R (*S*): 18.0 min, t_R (*R*): 19.8 min, 91:9 er.

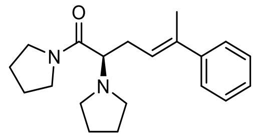

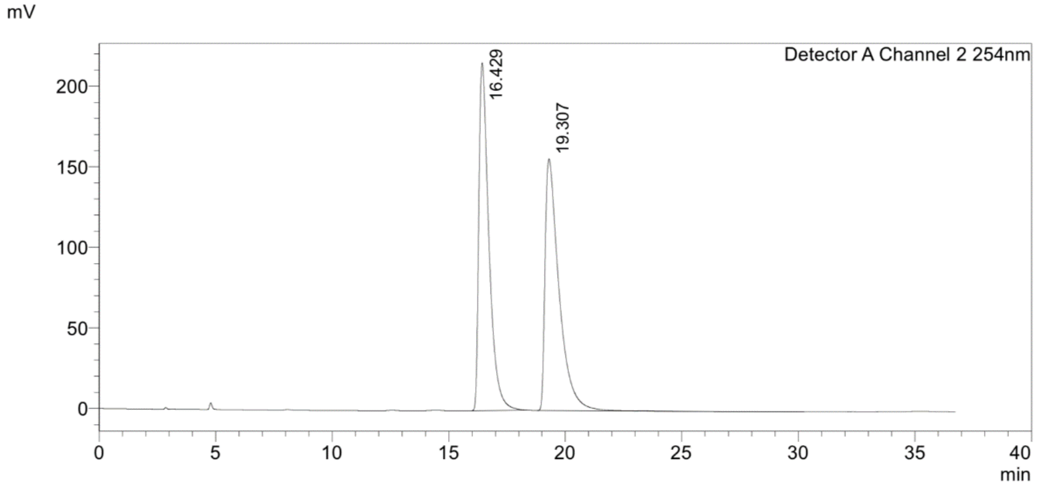

<Peak Table>

Detector A Channel 2 254nm

Peak#	Ret. Time	Area%
1	16.429	50.110
2	19.307	49.890
Total		100.000

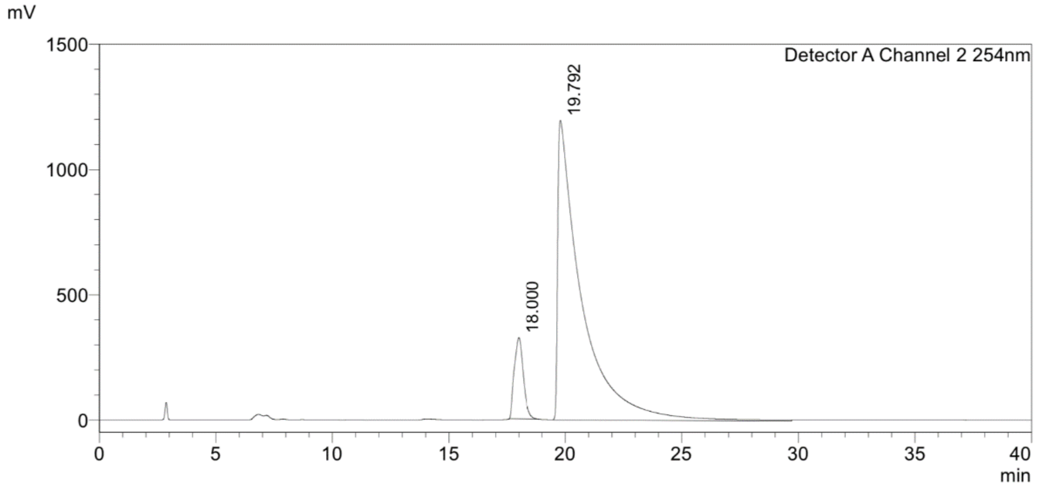

<Peak Table>

Detector A Channel 2 254nm

Peak#	Ret. Time	Area%
1	18.000	9.436
2	19.792	90.564
Total		100.000

3u - Chiralcel OJ-H (96:3.7:0.3 hexane/IPA/Et_3N, flow rate 2 $mLmin^{-1}$, 254 nm, 30 °C, t_R (*S*): 14.7 min, t_R (*R*): 16.8 min, 91:9 er.

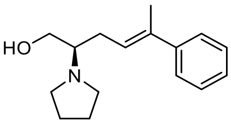

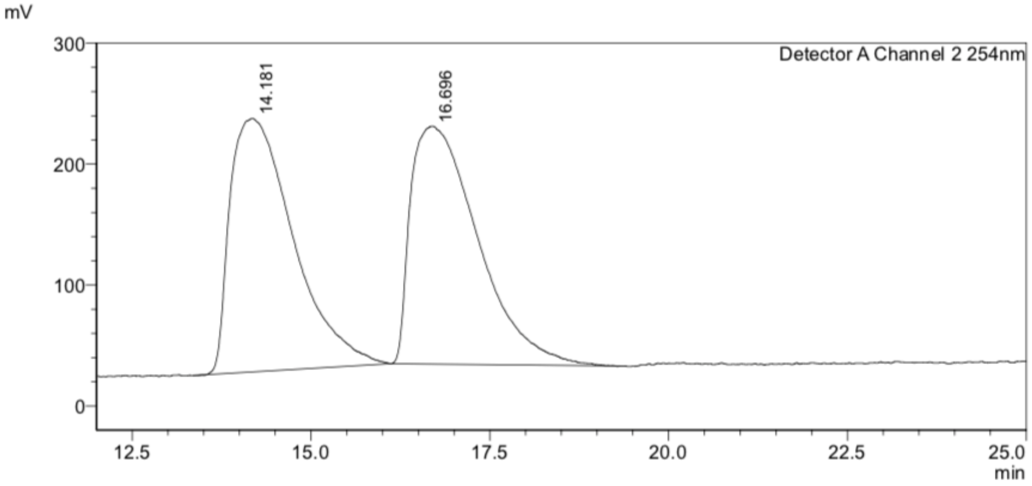

<Peak Table>

Detector A Channel 2 254nm

Peak#	Ret. Time	Area%
1	14.181	50.046
2	16.696	49.954
Total		100.000

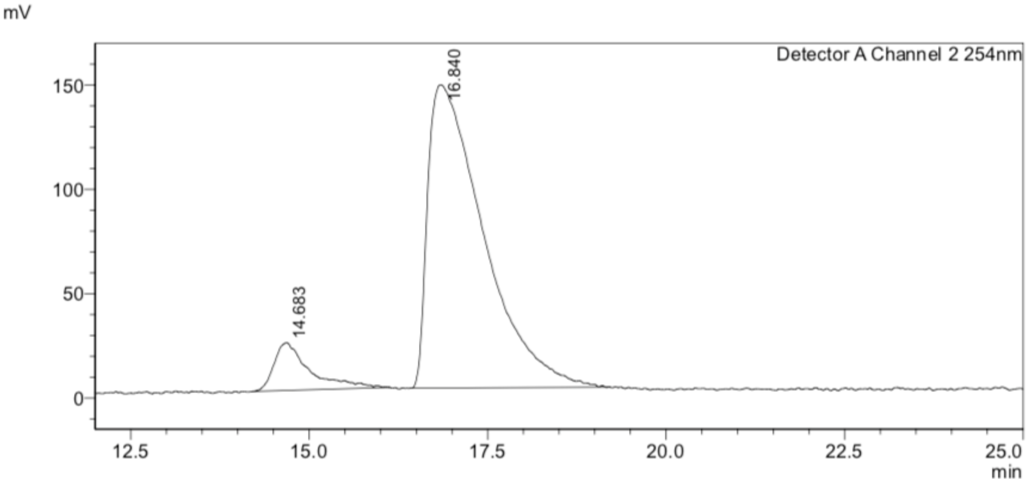

<Peak Table>

Detector A Channel 2 254nm

Peak#	Ret. Time	Area%
1	14.683	9.165
2	16.840	90.835
Total		100.000

3v - Chiracel AS-H (99.5:0.5 hexane/IPA, flow rate 1 mLmin^{-1}, 40 °C) t_R (major): 32.0 min, t_S (minor): 39.9 min, 88:12 er.

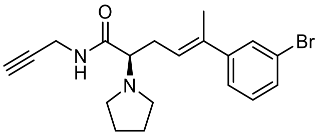

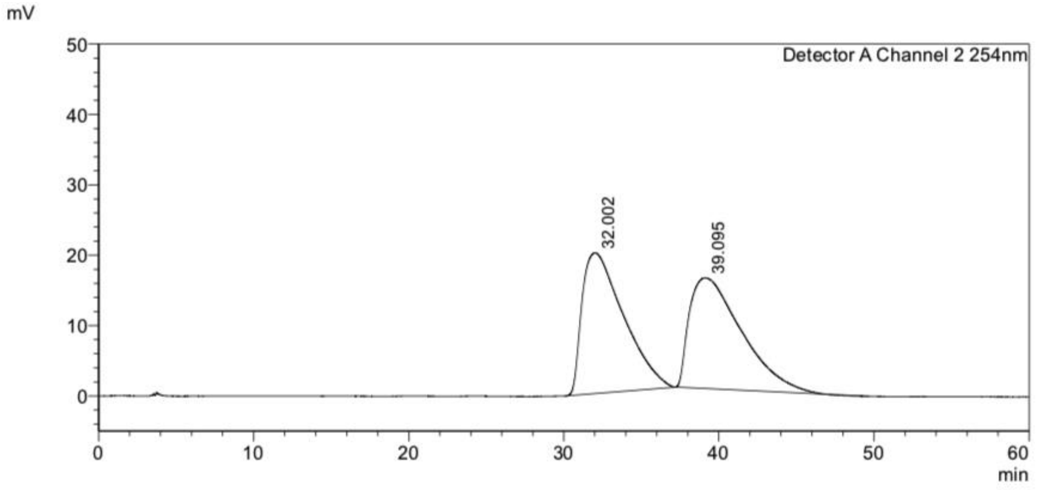

<Peak Table>

Detector A Channel 2 254nm

Peak#	Ret. Time	Area%
1	32.002	50.281
2	39.095	49.719
Total		100.000

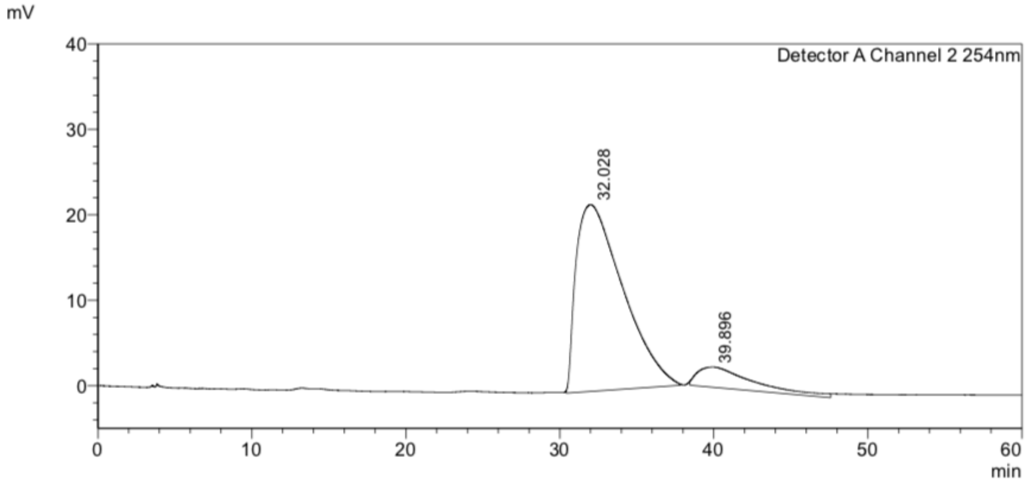

<Peak Table>

Detector A Channel 2 254nm

Peak#	Ret. Time	Area%
1	32.028	88.409
2	39.896	11.591
Total		100.000
